# Organocatalytic skeletal reorganization for enantioselective synthesis of *S*-stereogenic sulfinamides

**DOI:** 10.1038/s41467-024-48727-x

**Published:** 2024-05-22

**Authors:** Zanjiao Liu, Siqiang Fang, Haoze Li, Chunxiu Xiao, Kai Xiao, Zhishan Su, Tianli Wang

**Affiliations:** 1 Key Laboratory of Green Chemistry & Technology of Ministry of Education, College of Chemistry, Sichuan University, 610041 Chengdu, P. R. China; 2 Precision Medicine Research Center & Sichuan Provincial Key Laboratory of Precision Medicine, West China Hospital, Sichuan University, 610041 Chengdu, P. R. China; 3 Beijing National Laboratory for Molecular Sciences, 100190 Beijing, China

**Keywords:** Synthetic chemistry methodology, Organocatalysis, Homogeneous catalysis

## Abstract

The enantioselective synthesis of *S*-stereogenic sulfinamides has garnered considerable attention due to their structural and physicochemical properties. However, catalytic asymmetric synthesis of sulfinamides still remains daunting challenges, impeding their broad application in drug discovery and development. Here, we present an approach for the synthesis of *S*-stereogenic sulfinamides through peptide-mimic phosphonium salt-catalyzed asymmetric skeletal reorganization of simple prochiral and/or racemic sulfoximines. This methodology allows for the facile access to a diverse array of substituted sulfinamides with excellent enantioselectivities, accommodating various substituent patterns through desymmetrization or parallel kinetic resolution process. Mechanistic experiments, coupled with density functional theory calculations, clarify a stepwise pathway involving ring-opening and ring-closing processes, with the ring-opening step identified as crucial for achieving stereoselective control. Given the prevalence of *S*-stereogenic centers in pharmaceuticals, we anticipate that this protocol will enhance the efficient and precise synthesis of relevant chiral molecules and their analogs, thereby contributing to advancements in drug discovery.

## Abstract (web summary)

The enantioselective synthesis of *S*-stereogenic sulfinamides has garnered considerable attention due to their unique structural and physicochemical properties but catalytic asymmetric synthesis of sulfinamides still remains challenging. Here, the authors present the synthesis of *S*-stereogenic sulfinamides through the peptide-mimic phosphonium salt-catalyzed asymmetric skeletal reorganization of simple prochiral and racemic sulfoximines.

## Introduction

**Fig. 1:**
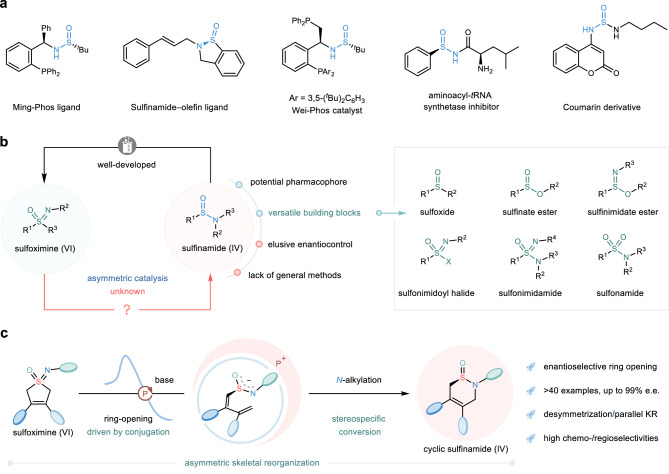
Motivation and design of PPS-catalyzed enantioselective transformation of sulfoximines to sulfinamides. **a** Importance of chiral/achiral sulfinamide compounds. **b** Challenge for sulfinamide chemistry: no precedent for transformation of sulfoximines to sulfinamides in a catalytic enantioselective manner. **c** This work: PPS-catalyzed enantioselective skeletal reorganization of sulfoximines for unified access to chiral sulfinamides.

Over the past century, innovations of catalytic asymmetric synthesis have rapidly expanded the realm of accessible chiral chemical entities for pharmaceutical research^1–3^. Despite some impressive advances have been achieved, the breadth and depth of chiral skeletal diversification in discovery and development of new drugs are still constrained due to the unsolved problems in particularly important catalytic asymmetric transformations^4^. In this scenario, a conspicuous example is that the sporadic occurrence of catalytic protocols towards chiral sulfinamides, a class of stereogenic-at-S(IV) scaffolds, which is thus difficult to fulfill the demands of systematically biological screening (Fig. 1a)^5–14^. To date, the most conventional and widely employed approach for synthesizing chiral sulfinamides primarily relies on using chiral starting materials^15–17^ or employing stoichiometric amounts of chiral auxiliaries^18,19^. Furthermore, catalytic strategies involve kinetic resolution enabled by enzyme^9^ or transition metal^20^ and asymmetric oxidation^10^. As such, a reliable and efficient method that will enable the construction of structurally and stereochemically diverse sulfinamide targets to meet diversified demands^21,22^ from multiple disciplines is highly desirable and urgently required.

Organocatalytic asymmetric reduction of hexavalent sulfoximines to sulfinamides represents an attractive and direct solution towards above target-specific synthesis. However, despite the considerable research efforts that have been focused on this area, compared to the well-established and widely-applied transformations of sulfinamides to sulfoximines, the concurrent S = O and S = N bonds in sulfoximine skeletons typically pose an inherent chemoselectivity challenge for such reduction process, let along the challenging ambiguous stereo-control, and thus no successful example has been developed so far (Fig. 1b, left)^23^. Of note, such type of reduction process was pioneered almost 30 years ago by Mock^24^, Gais^25^ and Pyne^26^, respectively via a skeletal reorganization strategy. However, their approaches had limited substrate scope and yielded moderate chemical yields. Despite the intriguing mechanism and high atom-economy^27,28^ associated with skeletal reorganization, it is surprising that a catalytic asymmetric version of this reaction has not been realized to date. The major challenges encountered in such asymmetric skeletal reorganizations not only lie in the high barrier in the S-C bond cleavage, which generally necessitates expensive transition-metal catalysts together with high reaction temperatures, undoubtedly limiting its broad application and functional group tolerance^29^, but also come from the formidable stereo-differentiation in the indistinguishable S-, O-, and N-nucleophilic centers of the in situ generated sulfinamide anions^30–36^. In addition, such process towards the target products might proceed with unpopular desulfurization reaction^26^.

Recognizing the notable obstacles presented in the aforementioned protocol, and to unlock the synthetic and biological potential of yet underutilized sulfinamide scaffolds, we sought to realize the catalytic enantioselective skeletal reorganization of achiral sulfoximines, thus producing the important chiral sulfinamide scaffolds. If successful, this proposed approach would establish a versatile molecular platform for the synthesis of libraries containing enantioenriched sulfinamide compounds, and potentially, for the generation of value-added downstream products (Fig. 1b, right)^7,37–39^. Inspired by our recent disclosure of peptide-mimic phosphonium salt (PPS) catalysts and their wide applications in asymmetric synthesis^40–45^, we anticipated that the enhanced ion-pairing induction and multiple hydrogen-bonding interaction of PPS catalyst system could potentially provide synergistic activation for promoting this transformation reaction bearing much less reactivity of substrates. Furthermore, this class of conformationally flexible and highly structural tunable catalysts might be also suitable candidates for addressing the major stereo-control challenge of such enantioselective skeletal reorganization reaction.

Herein, we disclose a PPS-catalyzed enantioselective skeletal reorganization of sulfoximines, guiding the precise and highly efficient construction of optically pure *S*-stereogenic sulfinamides (Fig. 1c). Crucially, the use of PPS catalysts bypass both the transition-metal catalysts and the previous high reaction temperatures required by the in-situ generation of conjugated diene-tethered sulfinamide intermediates, in which the existence of conjugation element may ensure an effective thermodynamic driving force for the initial enantioselective S-C bond cleavage. Moreover, this protocol employing prochiral and/or racemic cyclic sulfoximines as starting reagents and harnessing one-pot operations with cascade process leads to a broad range of enantioenriched cyclic sulfinamides with an assortment of functional groups in high yields with excellent enantioselectivities.

## Results

### Reaction development

**Fig. 2:**
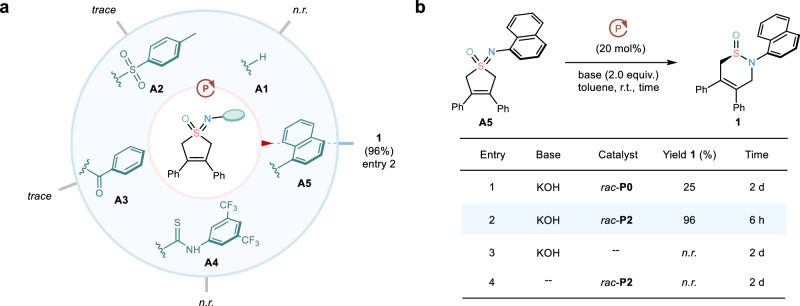
Development of asymmetric skeletal reorganization reaction. **a** Preliminary experiments. **b** Control experiments. *rac*-**P0** = Me_2_P^+^Ph_2_I^-^. Reactions were conducted by using different *N*-protected sulfoximines (0.1 mmol) in the presence of racemic catalyst **P0**/**P2** (20 mol%), KOH (2.0 equiv.) in toluene (2.5 ml) at r.t. The isolated yields were given.

Given the aforementioned challenges, we initiated our investigations by testing the skeletal reorganization of prochiral cyclic sulfoximine **A1** with a free NH unit as a benchmark reaction in the presence of a racemic PPS catalyst **P2** (Fig. 2a). However, no formation of the desired sulfinamide was observed, which might be attributed to the NH-involving hydrogen transfer process. Thus, we prepared a series of *N*-protected cyclic sulfoximines and tested the validity of our hypothesis. Encouragingly, the use of the naphthyl protecting group could afford the desired skeletal reorganization product with a nearly quantitative yield, while the other *N*-protected sulfoximines **A2**-**A4** suffered from the instability problem. Control experiments uncovered that the nature of ion-pairing and H-bonding effects appeared to play a pivotal role in realizing this reaction, wherein the lack of H-bonding donors (Fig. 2b, entry 1), let alone the lack of PPS catalysts (Fig. 2b, entry 3), would result in a sluggish reaction under otherwise identical conditions. Additionally, the loss of reactivity without the base also indicated an ionic reaction pathway (Fig. 2b, entry 4).

**Fig. 3:**
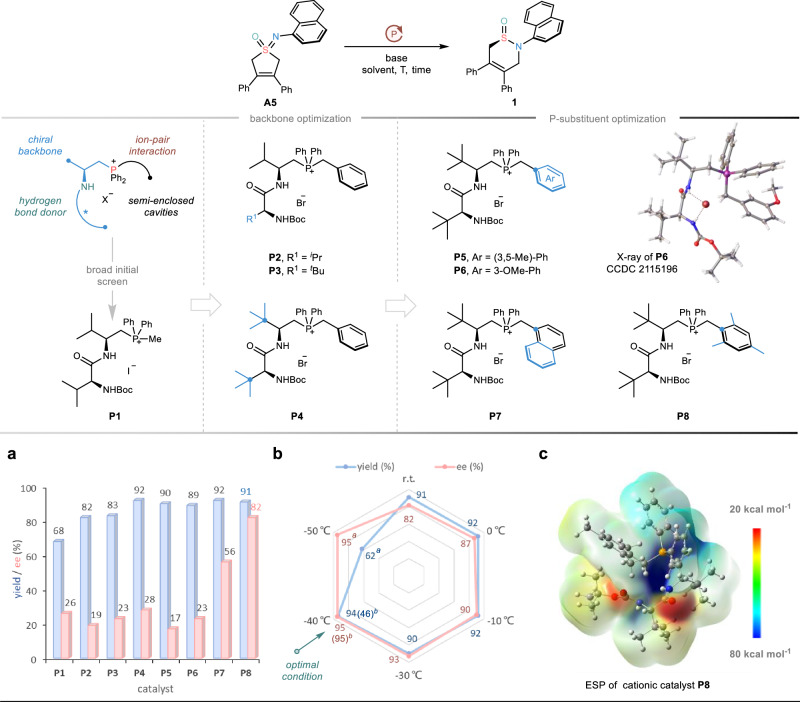
Reaction optimization. **a** Catalyst screening. **b** Effects of other reaction parameters including temperature and catalyst loading. **c** Electrostatic potential surface (ESP) of cationic catalyst **P8**. Unless indicated, all the reactions were performed with **A5** (0.1 mmol), **P** (20 mol%), KOH (2.0 equiv.), in toluene (2.5 ml) for 6 hours. ^*a*^The reaction was stirred for 24 h. ^*b*^**P8** (5 mol%) was used and the reaction was stirred for 4 days. The isolated yields were given. All the e.e. values were determined by HPLC analysis.

Encouraged by the preliminary success, we turned our attention to the sought-after catalytic enantioselective skeletal reorganization of prochiral cyclic sulfoximines. As shown in Fig. 3a, initial extensive evaluation of chiral PPS catalysts revealed that the (*L*, *L*)-dipeptide-based phosphonium salt catalyst (**P1**) led to inspiring enantioinduction, albeit with a decrease in the reactivity. Exchanging the methyl substituent of the P(V) atom to a benzyl group could enhance the reactivity. As such, further skeleton optimization of PPS catalysts was performed, wherein *L*-*tert*-Leu-*L*-*tert*-Leu-derived phosphonium salt **P4** furnished the product **1** in 92% yield with 28% e.e. In view of the density functional theory (DFT) calculated electrostatic potential (ESP) of chiral PPS cationic catalyst **P8** (Fig. 3c), we speculated that multiple hydrogen-bonding donors with an improved electropositive region could offer better recognition of the transition state structures. Thus, the substituent effects of the active P(V)-center were investigated, and surprisingly, catalyst **P8** led to a significant increase in enantioselectivity (82% e.e.). Then, the catalyst **P8** was used for further screening of other parameters (see the Supplementary Tables 3–8 for details). Remarkably, an obvious increase in enantioselectivity (i.e. 95% e.e.) coupled with a satisfactory yield was observed while lowering the reaction temperature to −40 °C, even under a very low catalyst loading of 5 mol% (Fig. 3b).

### Reaction scope

**Fig. 4:**
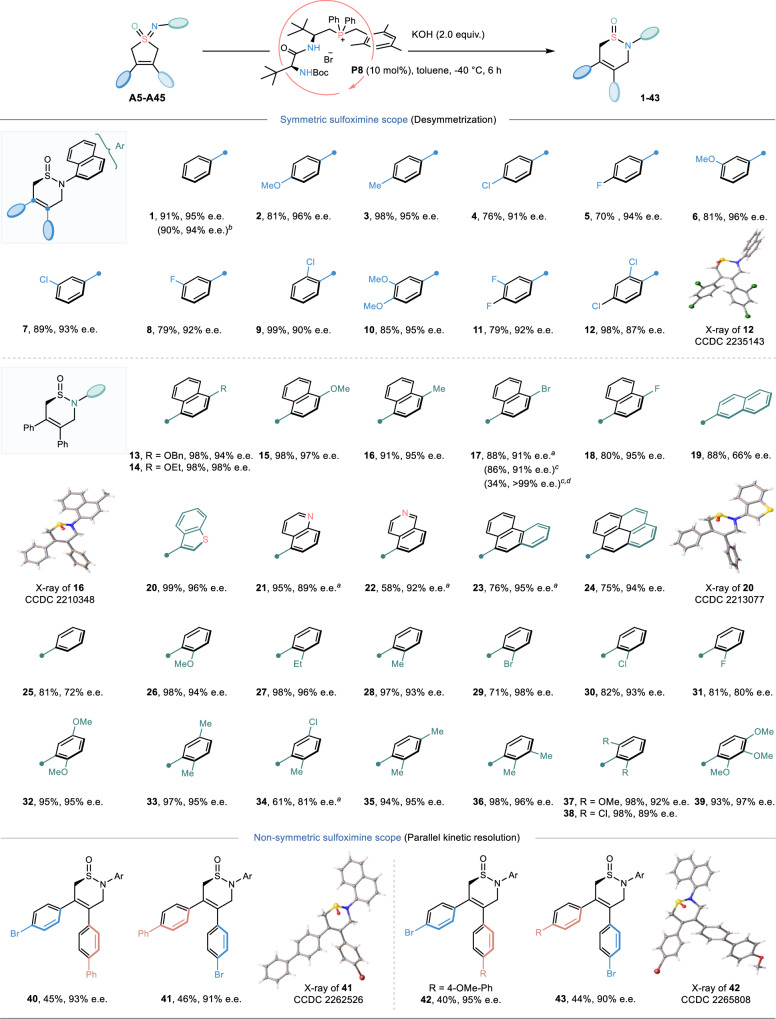
Substrate scope and Gram-scale synthesis. Standard reaction conditions: **A** (0.1 mmol), **P8** (10 mol%), KOH (2.0 equiv.) in toluene (1.0 ml) at −40 ^o^C for 6 hours. ^*a*^The reaction was stirred for 48 hours. ^*b*^Reaction was performed on a 2.5 mmol scale. ^*c*^Reaction was performed on a 1.1 mmol scale. The isolated yields were given. ^*d*^After once recrystallization. The e.e. values were determined by HPLC analysis.

With the optimal catalyst and other reaction conditions in hand, the substrate scope of this catalytic asymmetric skeletal reorganization was systematically explored. As shown in Fig. 4, a variety of symmetric cyclic sulfoximines with aryl substitutions on the alkene moiety were firstly prepared and subjected to this transformation. Obviously, both electron-donating and electron-withdrawing groups at different positions (i.e. *para-, meta-* or *ortho*-position) were tolerated well, affording the corresponding products (**1−9**) in good to excellent yields (70**−**99%) with good enantioselectivities (90**−**96%). Likewise, disubstituted substrates(**A14**–**A16**) were also suitable for this transformation. To further expand the generality of this protocol, other orthogonal *N*-protecting groups were examined. Good functional group compatibility was, in most cases, observed with tolerance of a series of sterically and electronically differentiated aromatics and heteroaromatics (**A17−A43**). Besides, a decrease in stereocontrol of sulfinamides **19** and **25** indicated the importance of steric hindrance of the aromatic ring bonded to nitrogen atom at the *ortho-*position. Of note, this reaction could be scalable without any loss of efficiency (eg. **1** and **17**). Additionally, the absolute configurations of **12, 16** and **20** were determined by X-ray diffraction analysis and those of other products were assigned by analogy. Remarkably, beyond symmetric cyclic sulfoximines, this catalytic system was also effective for non-symmetric counterparts. For example, the racemic sulfoximines bearing different aryl groups on the C = C moiety could be engaged to this skeletal reorganization reaction, simultaneously yielding two classes of expected enantioenriched products (such as **40/41** and **42**/**43**), absolute configurations of which were confirmed by X-ray crystallographic analysis. This sophisticated parallel kinetic resolution^46^ emphasized the practicability of this construction strategy towards chiral sulfinamide compounds.

### Synthetic applications

**Fig. 5:**
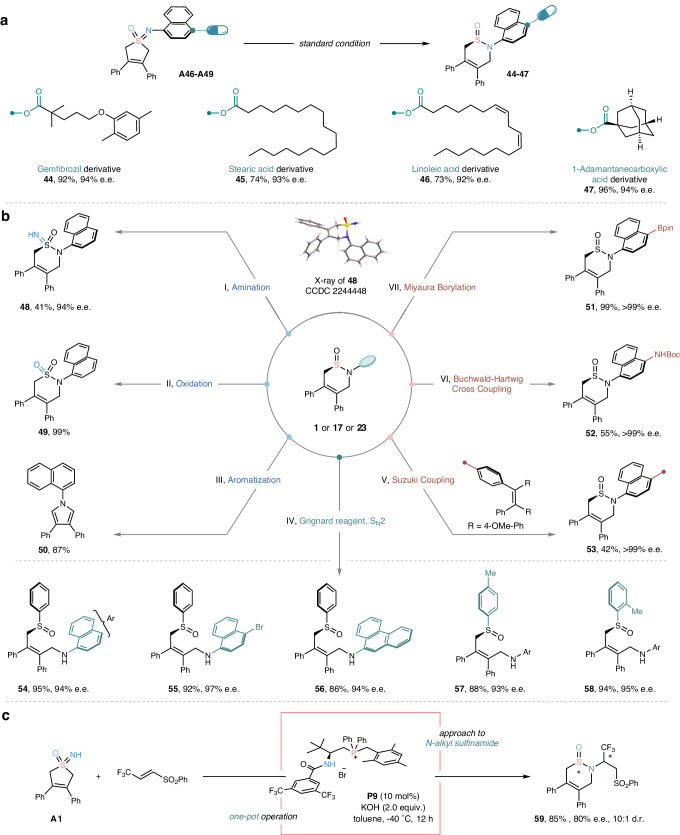
Applications of chiral cyclic sulfinamides. **a** Late-stage diversification of bioactive molecules and material building blocks. **b** Derivatization of the chiral sulfinamide products. I, Amination. Sulfinamide **1** (0.1 mmol), ammonium carbamate (2.0 equiv.) and iodobenzene diacetate (2.5 equiv.) in MeOH at r.t. II, Oxidation. Sulfinamide **1** (0.1 mmol), mCPBA (1.4 equiv.) and NaHCO_3_ (2.5 equiv.) in CH_2_Cl_2_ at r.t. III, Aromatization. Sulfinamide **1** (0.1 mmol), Pd(OAc)_2_ (10 mol%), XPhos (30 mol%) and Cs_2_CO_3_ (1.4 equiv.) in dioxane at 100 ^o^C. IV, S_N_2 substitution. Sulfinamide (0.1 mmol) and ArMgCl (1.2 equiv.) in CPME solvent at −80 ^o^C. V, Suzuki Coupling. Sulfinamide **17** (0.1 mmol), aryl boronic acid pinacol ester (1.2 equiv.), Pd(PPh_3_)_4_ (5 mol%) and K_2_CO_3_ (2.0 equiv.) in THF/H_2_O (v/v = 3/1) at 90 ^o^C. VI, Buchwald-Hartwig Cross Coupling. Sulfinamide **17** (0.1 mmol), BocNH_2_ (1.5 equiv.), Pd(OAc)_2_ (10 mol%), XPhos (30 mol%) and Cs_2_CO_3_ (1.4 equiv.) in dioxane at 100 ^o^C. VII, Miyaura Borylation. Sulfinamide **17** (0.1 mmol), Pd(dppf)Cl_2_ (10 mol%), B_2_pin_2_ (4.0 equiv.) and KOAc (5.0 equiv.) in dioxane at 90 ^o^C. Also see the synthetic application section in Supplementary Information for more condition details. **c** Approach to *N*-alkyl sulfinamide via one-pot operation.

To investigate the versatility of this catalytic system, we subsequently examined its application in the late-stage functionalization of complex skeletons. To our delight, all of the elaborate sulfoximines derived from bioactive molecules or material building blocks, such as Gemfibrozil, Stearic acid, Linoleic acid etc., could serve as effective substrates, furnishing the corresponding skeletal reorganization products (**44**−**47**) in good yields with excellent enantioselectivities (Fig. 5a). Furthermore, a wide variety of powerful synthetic transformations of the resulting chiral sulfinamides were also demonstrated. For instance, the sulfinamide **1** could be readily converted into sulfonimidamide **48** and sulfonamide **49** by simple amination and oxidation. Of note, an unexpected desulfurized aromatization occurred towards the facile assembly of the pyrrole skeleton **50** under the transition metal catalytic condition (Fig. 5b, left). Another interesting and important application would be the expedient installation of various types of functionalities via a series of cross-coupling (i.e. Miyaura borylation, Buchwald-Hartwig coupling and Suzuki coupling) of brominated chiral sulfinamides, affording the corresponding products (**51**−**53**) without any loss of enantiopurity (Fig. 5b, right). Notably, the vulnerable nature of the S−N bond in sulfinamides rendered them versatile building blocks for further elaborations towards chiral sulfoxide compounds (**54**−**58**), many of which have been previously deemed as synthetically challenging (Fig. 5b, bottom)^47,48^. Notably, though a striking steric-hindrance for better asymmetric induction has been demanded, the challenging *N*-alkyl-substituted sulfoximine also gave access to the corresponding skeletal reorganization product in 85% isolated yield with 80% e.e. and 10:1 *d.r*. via one-pot operation, employing an improved phosphonium salt catalyst (Fig. 5c).

### Mechanistic investigations

**Fig. 6:**
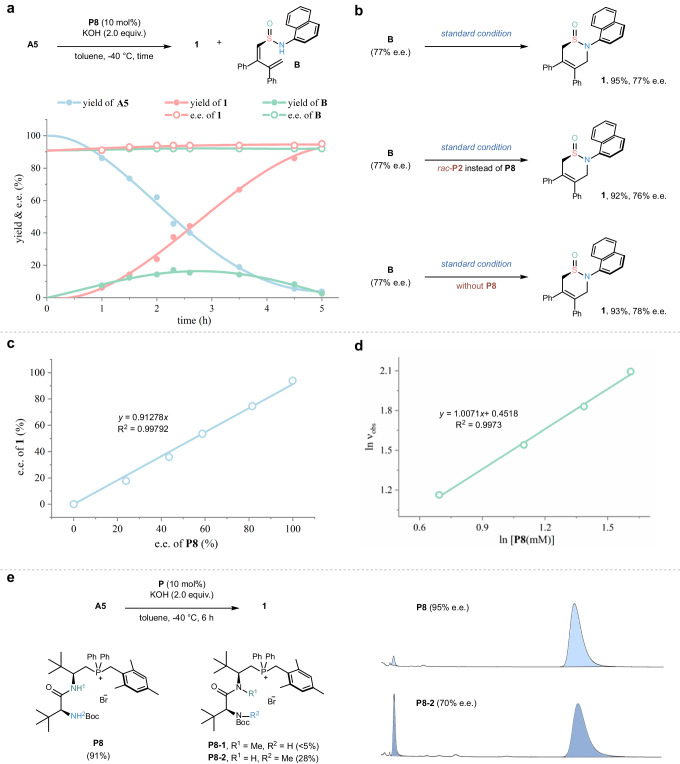
Mechanistic studies. **a** Monitoring the reaction process over time. **b** Control experiments to illustrate the transformation from the species **B**. **c** Non-linear study. **d** Reaction kinetics to determine the reaction order of catalyst **P8**. **e** Investigation of weak interactions.

To shed light on this underlying reaction mechanism, a series of kinetic and spectroscopic studies were performed. Our initial interest was focused on the identification of the key intermediate in this organocatalytic skeletal reorganization of sulfoximines. Thus, a time-course study for this reaction process with substrate **A5** was conducted (Fig. 6a). The kinetic profile indicated the presence of a short-lived enantioenriched species, which was confirmed as the conjugated diene-tethered sulfinamide **B**. Additionally, we performed several elaborate control experiments. As shown in Fig. 6b, such chiral specie **B** could be smoothly converted into the desired chiral product **1** without erosion of enantioselectivity, irrespective of harnessing either chiral catalyst **P8** or racemic catalyst **P2**. Based on these results, we suggested an enantioselective ring-opening and subsequent ring-closing step-wise pathway towards the facile access to these chiral sulfinamide products.

Subsequently, we turned our interest in stereochemical probing to the significant ring-opening step. The nonlinear effect experiment between chiral catalyst **P8** and product **1** was firstly conducted (Fig. 6c), and at last a linear relationship was observed, which uncovered a monomeric catalytic mode in the stereo-determining step, in correspondence with the Job Plot analysis of the ^1^H NMR titration experiments (see the Supplementary Figs. 16–18 for details). Further kinetic studies also illustrated that the overall reaction is the first order relationship with the catalyst **P8** (Fig. 6d). Then, to investigate the nature of multiple weak-bonding activations, the methylated phosphonium salts **P8-1** and **P8-2** were prepared and subjected to the standard reaction (Fig. 6e). As a result, both of the reactivity and enantioselectivity suffered from a dramatical decline, thus indicating the indispensability of H-bonding interactions in the asymmetric induction.

**Fig. 7:**
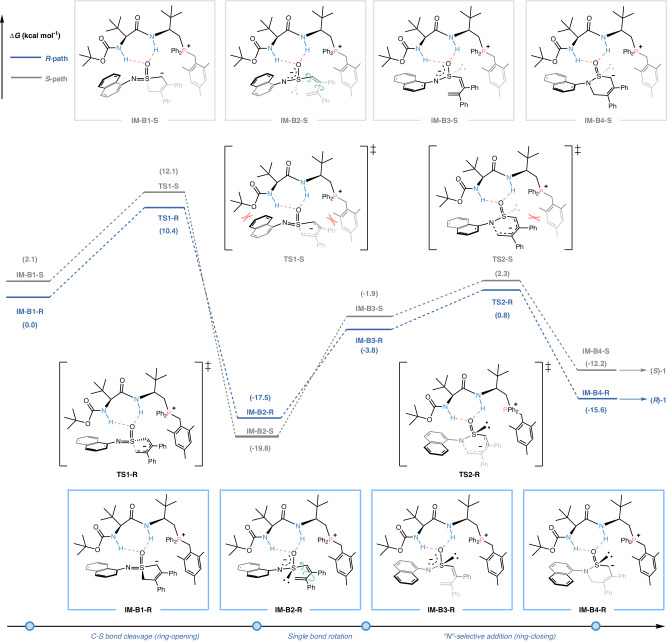
Energy profiles for enantioselective skeletal reorganization reaction under phosphonium salt catalyst P8 system. All the structures were calculated at the M06-2X-D3/6-311 G(d, p)(SMD, toluene)// M06-2X/6-31 G(d)(SMD, toluene) theoretical level, employing Gaussian 09 program package.

To provide further molecular-level insight into this process, DFT calculations were carried out to understand the reaction pathway and the origin of stereoselectivity by employing Gaussian 09 program package (see the Supplementary Information for more details)^49^. As shown in Fig. 7, the catalyst cation **P8** firstly interacted with the oxygen atom of the deprotonated substrate via dual H-bonding interactions, forming the intermediates **IM-B1-R** and **IM-B1-S**, alternatively. The NH···O(S) distances in **IM-B1-R** were 1.96 and 1.86 Å, which were slightly shorter than those in **IM-B1-S** (2.10 and 1.87 Å). Accordingly, **IM-B1-R** was more stable than **IM-B1-S** by 2.1 kcal mol^−1^. Then, **IM-B1-R** and **IM-B1-S** underwent the ring-opening process, affording **IM-B2-R** and **IM-B2-S** via transition states **TS1-R** and **TS1-S**, respectively. Due to significant repulsion between *ortho*-CH_3_ group in the Bn unit of catalyst and the Ph group of sulfoximine substrate, as well as unfavorable steric effect between the two *t-*Bu groups and the naphthyl group, the relative Gibbs free energy (∆*G*) of **TS1-R** was lower than that of **TS1-S** by 1.7 kcal mol^−1^. Next, **IM-B2-R** and **IM-B2-S** underwent C−S single bond rotation, followed by ring-closing process to construct the C−N bonds in the **IM-B4-R** and **IM-B4-S**. This step could occur easily, with the Δ*G*^≠^ as low as 4.6 and 4.2 kcal mol^−1^, respectively. Otherwise, the energy profile for transformation of conjugated diene-tethered anion to sulfinamide anion along *R*-path without catalyst has also been calculated, which was highly consistent with the experimental results (Fig. 6) and reasonably illustrated that the “*N*”-selective ring-closing might be a stereospecific process to form the product with *R*-configuration eventually. In the view point of energy, the ring-opening of the sulfoximine was the key step of stereocontrol with the ΔΔ*G* of 1.7 kcal mol^−1^. The theoretical stereoselectivity was predicted to be 94% e.e. at 233 K, which was close to experimental observation (95% e.e.).

## Discussion

In summary, we have successfully developed the catalytic enantioselective skeletal reorganization of sulfoximines, facilitating the creation of enantioenriched sulfinamides featuring a *S*-stereocenter. Amid this process, peptide-mimic phosphonium salts, which serve as multifunctional weak-bonding catalysts to not only activate C−S bonds but also afford efficient asymmetric induction, are crucial for this formal metal-free reduction. Remarkably, leveraging the versatility of this class of cyclic sulfinamides, this current methodology could provide a powerful platform to allow access to a wide variety of sought-after stereogenic-at-sulfur scaffolds, such as chiral sulfonimidamides and sulfoxides etc. We anticipate that this general and efficient method for the synthesis of enantioenriched sulfinamide compounds would open up an avenue for related drug discovery and development.

## Methods

### Procedure for the synthesis of chiral sulfinamides 1−47

Sulfoximines **A** (0.1 mmol), KOH (0.2 mmol, 2.0 equiv.) and catalyst **P8** (0.01 mmol, 10 mol%) were added to a dried reaction tube with a magnetic stirring bar under air, followed by the addition of precooling toluene (1.0 mL). The reaction mixture was stirred at −40 °C until completion determined by TLC. Then, the reaction mixture was directly purified by column chromatography on silica gel (petroleum ether/ethyl acetate = 5:1) to afford the desired products.

### Reporting summary

Further information on research design is available in the Nature Portfolio Reporting Summary linked to this article.

### Supplementary information

Supplementary Information

Peer Review File

Reporting Summary

### Source data

Source Data

## Data Availability

Crystallographic data for the structures reported in this article have been deposited at the Cambridge Crystallographic Data Centre, under deposition numbers CCDC 2115196 (**P6**), CCDC 2235143 (**12**), CCDC 2210348 (**16**), CCDC 2213077 (**20**), CCDC 2262526 (**41**), CCDC 2265808 (**42**) and CCDC 2244448 (**48**). Copies of the data can be obtained free of charge via https://www.ccdc.cam.ac.uk/structures/. All information relating to initial studies, optimization studies, experimental procedures, mechanistic studies, DFT calculations, high-performance liquid chromatography spectra, NMR spectra, high-resolution mass spectrometry and optical rotation data are available in Supplementary Information. All other data are present and available from the corresponding authors upon request. Source data are provided with this paper.
