# Supplementary material for: Organocatalytic skeletal reorganization for enantioselective synthesis of S-stereogenic sulfinamides
Source: Nat Commun. 2024 May 22;15:4348. doi: 10.1038/s41467-024-48727-x (PMC11111665; doi:10.1038/s41467-024-48727-x)
Supplement: Supplementary file 1 — Supplementary Information [file 41467_2024_48727_MOESM1_ESM.pdf]

Supplementary Information

Organocatalytic skeletal reorganization for enantioselective synthesis of *S*-stereogenic sulfinamides

Zanjiao Liu,[1,4] Siqiang Fang,[1,4] Haoze Li,[1,4] Chunxiu Xiao,[2] Kai Xiao,[2] Zhishan Su,*[,1] Tianli Wang, *[,1,3]

[1]*Key Laboratory of Green Chemistry & Technology of Ministry of Education, College of Chemistry, Sichuan University, 29 Wangjiang Road, Chengdu 610064, P. R. China.*

[2]*Precision Medicine Research Center & Sichuan Provincial Key Laboratory of Precision Medicine, West China Hospital, Sichuan University, Chengdu 610041, P. R. China.*

[3]*Beijing National Laboratory for Molecular Sciences, Beijing 100190, China.*

[4]*These authors contributed equally to this work.*

e-mail: suzhishan@scu.edu.cn; wangtl@scu.edu.cn

Supplementary Methods--2

1. General Information--2
2. Initial Studies of Skeletal Reorganization Reaction--------------------------------3
3. Optimization of Reaction Conditions---4
4. Preparation of Chiral Phosphonium Salt Catalysts--------------------------------8
5. Preparation of Cyclic Sulfoximines---13
6. General Procedure for Enantioselective Skeletal Reorganization----------------41
7. Applications of Chiral Cyclic Sulfinamides-------------------------------------100
8. X-Ray Data of Chiral Compounds---124
9. Mechanistic Investigations---132
10. NMR Spectra of New Compounds---176

Supplementary References---302

Supplementary Methods

1. General Information

All the starting materials were obtained from commercial sources and used without further purification unless otherwise stated. ^{1}H and ^{13}C NMR spectra were recorded at ambient temperature in $CDCl_3$ or DMSO-*d6* on a Brüker Advance 400 spectrometer. Chemical shifts (δ) were given in parts per million (ppm), and the residual solvent peak was used as an internal reference ($CDCl_3$: δ 7.26 ppm ^{1}H; δ 77.16 ppm ^{13}C; DMSO-*d6*: δ 2.50 ppm ^{1}H; δ 39.51 ppm ^{13}C). Multiplicity was indicated as follows: s (singlet), d (doublet), t (triplet), q (quartet), m (multiplet), dd (doublet of doublet), br s (broad singlet). Coupling constants (*J*) were reported in Hertz (Hz). High resolution mass spectra were obtained on a Thermo LTQ mass spectrometer. For thin layer chromatography (TLC), Merck pre-coated TLC plates (Merck 60 F254) were used, and compounds were visualized with a UV light at 254 nm. Flash chromatographic separations were performed on Merck 60 (0.040-0.063 mm) mesh silica gel. Enantiomeric excess was determined by HPLC analysis using chiral column described below in detail. Optical rotations were measured with polarimeter.

2. Initial Studies of Skeletal Reorganization Reaction

Supplementary Table 1. Design of sulfoximines as substratese [a,b]

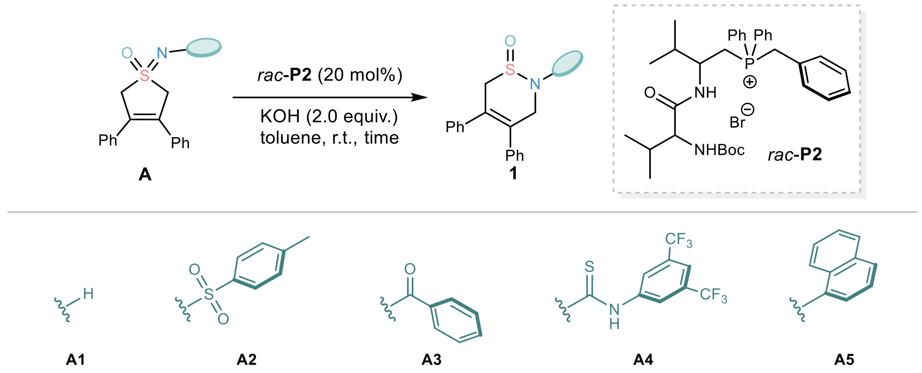

Entry	A	Yield (%)	Time
1	**A1**	*n.r.*	2 d
2	**A2**	trace	2 d
3	**A3**	trace	2 d
4	**A4**	*n.r.*	2 d
5	**A5**	96%	6 h

[a] Reaction conditions: **A** (0.1 mmol), KOH (0.2 mmol) and the catalyst *rac*-**P2** (20 mol%) in toluene (2.5 mL) at room temperature. [b] All yields were isolated yields.

Supplementary Table 2. Feasibility analysis of the reaction [a,b]

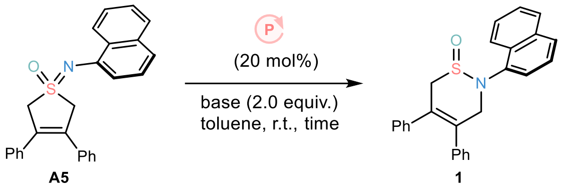

Entry	Base	Catalyst	Yield (%)	Time
1	KOH	*rac*-**P0**	25	2 d
2	KOH	*rac*-**P2**	96	6 h
3	KOH	--	*n.r.*	2 d
4	--	*rac*-**P2**	*n.r.*	2 d

[a] Reaction conditions: **A** (0.1 mmol), KOH (0.2 mmol) and the catalyst (20 mol%) in toluene (2.5 mL) at room temperature. [b] All yields were isolated yields. *rac*-**P0**: $Me_2P^+Ph_2I^-$

3. Optimization of Reaction Conditions

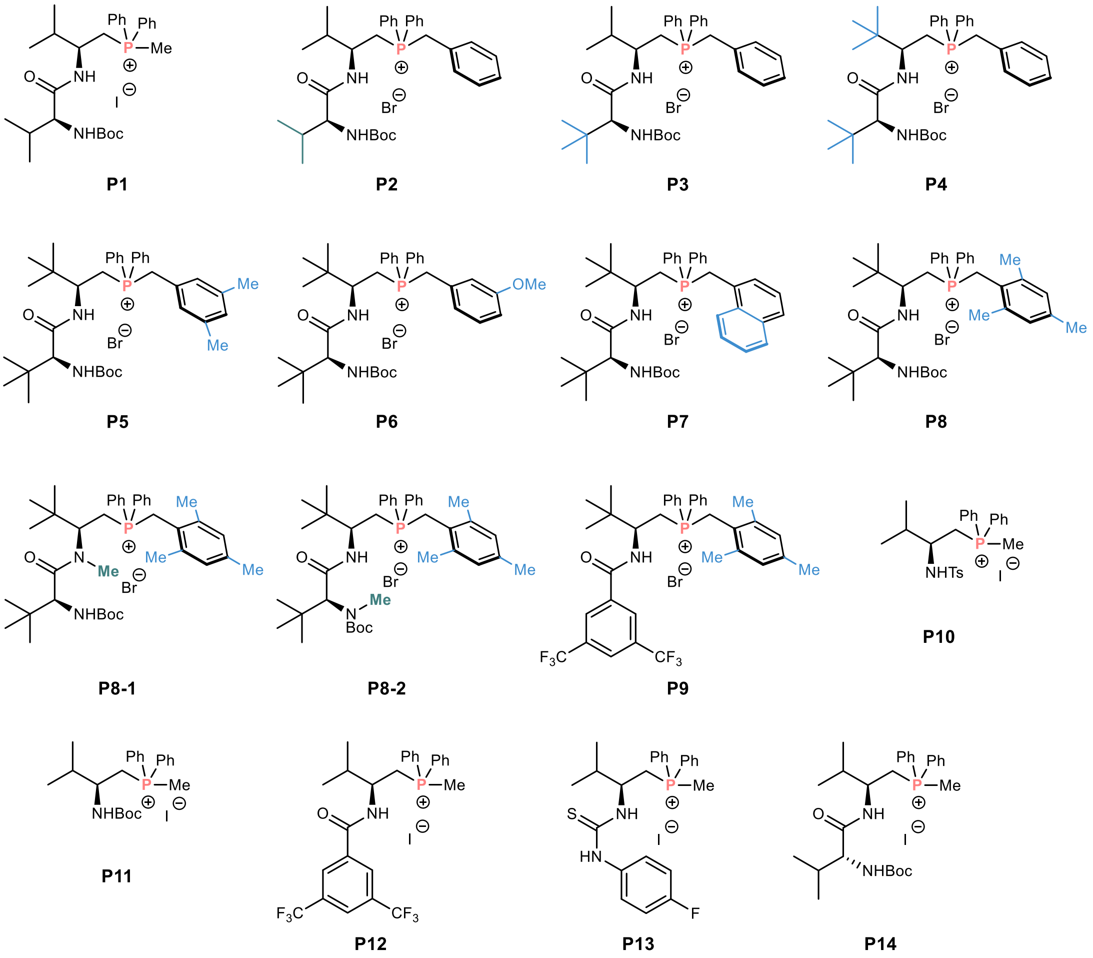

Supplementary Fig. 1. Chiral phosphonium salt catalysts examined in this study.

Supplementary Table 3. Optimization of the catalysts[a,b]

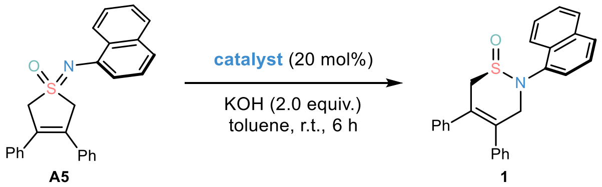

Entry	Catalyst	Yield (%)	e.e. (%)
1	**P1**	68	26
2	**P2**	82	19
3	**P3**	83	23
4	**P4**	92	28

5	**P5**	90	17
6	**P6**	89	23
7	**P7**	92	56
**8**	**P8**	**91**	**82**
9	**P9**	85	20
10	**P10**	88	4
11	**P11**	72	6
12	**P12**	85	11
13	**P13**	64	-19
14	**P14**	76	10

[a] Reaction conditions: **A5** (0.1 mmol), KOH (0.2 mmol) and the catalyst (20 mol%) in toluene (2.5 mL) at room temperature for 6 hours. [b] All e.e. values were determined by HPLC analysis on a chiral stationary phase, and all yields were isolated yields.

Supplementary Table 4. Optimization of the solvents[a,b]

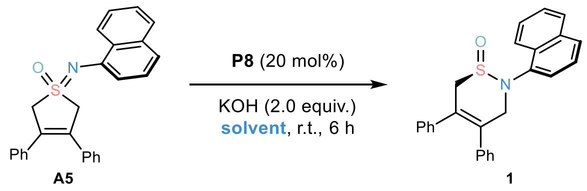

Entry	Solvent	Yield (%)	e.e. (%)
1	Et_2O	92	74
2	Hexane	87	50
**3**	**Toluene**	**91**	**82**
4	DCM	90	58
5	EA	88	39
6	PE (b.p. 60-90)	76	57
7	THF	94	63
8	$CHCl_3$	52	62

[a] Reaction conditions: **A5** (0.1 mmol), KOH (0.2 mmol) and **P8** (20 mol%) in solvent (2.5 mL) at room temperature for 6 hours. [b] All e.e. values were determined by HPLC analysis on a chiral stationary phase, and all yields were isolated yields.

Supplementary Table 5. Optimization of the bases[a,b]

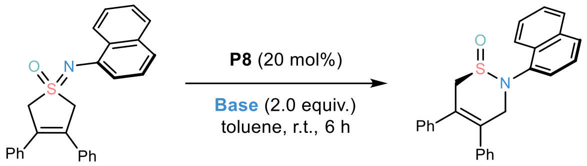

Entry	Base	Yield (%)	e.e. (%)
1	PhONa	32	81
**2**	**KOH**	**91**	**82**
3	$CsOH•H_2O$	75	79
4	Cs_2CO_3	92	81
5	Na_2CO_3	*n.r.*	--
6	K_2CO_3	*n.r.*	--
7	K_3PO_4	83	78
8	$K_3PO_4•3H_2O$	64	81
9	TEA	*n.r.*	--
10	*t*-BuOK	86	61

[a] Reaction conditions: **A5** (0.1 mmol), Base (0.2 mmol) and **P8** (20 mol%) in toluene (2.5 mL) at room temperature for 6 hours. [b] All e.e. values were determined by HPLC analysis on a chiral stationary phase, and all yields were isolated yields.

Supplementary Table 6. Optimization of the temperatures[a,b]

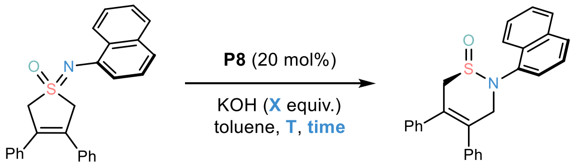

Entry	T (℃)	KOH (X equiv.)	Yield (%)	e.e. (%)	Time (h)
1	r.t.		91	82	6
2	0		92	87	6
3	-10	**2.0**	92	90	6
4	-30		90	93	6
**5**	**-40**		**94**	**95**	**6**

6	-50		62	95	24
7	-60		*n.r.*	--	24
8	-40	1.0	18	95	6
9		4.0	88	93	6

[a] Reaction conditions: **A5** (0.1 mmol), KOH (X equiv.) and **P8** (20 mol%) in toluene (2.5 mL) at T. [b] All e.e. values were determined by HPLC analysis on a chiral stationary phase, and all yields were isolated yields.

Supplementary Table 7. Optimization of the loading of catalysts[a,b]

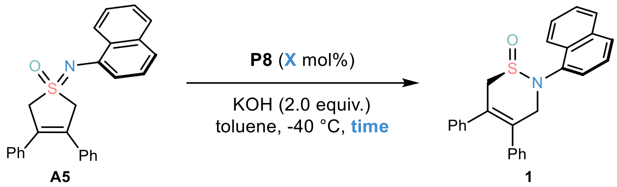

Entry	**P8** (mol%)	Yield (%)	e.e. (%)	Time
1	20	94	95	6 h
**2**	**10**	**90**	**95**	**6 h**
3	5	46	95	4 d

[a] Reaction conditions: **A5** (0.1 mmol), KOH (0.2 mmol) and **P8** (X mol%) in toluene (2.5 mL) at -40 °C. [b] All e.e. values were determined by HPLC analysis on a chiral stationary phase, and all yields were isolated yields.

Supplementary Table 8. Optimization of reaction concentration [a,b]

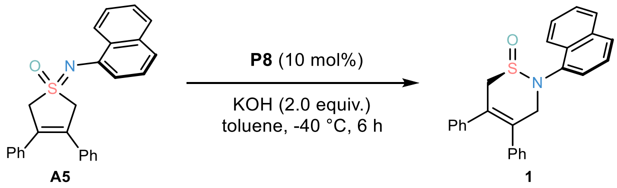

Entry	C (mol/L)	Yield (%)	e.e. (%)
1	0.01	82	95
2	0.04	90	95
**3**	**0.1**	**91**	**95**

[a] Reaction conditions: **A5** (0.1 mmol), KOH (0.2 mmol) and **P8** (10 mol%) in toluene (X mL) at -40 °C for 6 hours. [b] All e.e. values were determined by HPLC analysis on a chiral stationary phase, and all yields were isolated yields.

4. Preparation of Chiral Phosphonium Salt Catalysts

All bifunctional phosphonium salt catalysts **P0-P14** used in this study were prepared via a P-alkylation reaction of our previously reported organophosphines according to the known procedures.[1-6] Except for **P5**, **P6**, **P7**, **P8, P8-1, P8-2** and **P14**, other catalysts are known compounds, and their characterization data were in agreement with those reported in the literature[7-9]. Unknown compounds **P5**, **P6**, **P7**, **P8, P8-1, P8-2** and **P14** were fully characterized.

((*S*)-2-((*S*)-2-((tert-butoxycarbonyl)amino)-3,3-dimethylbutanamido)-3,3-dimethylbutyl)(3,5-dimethylbenzyl)diphenylphosphonium bromide (P5)

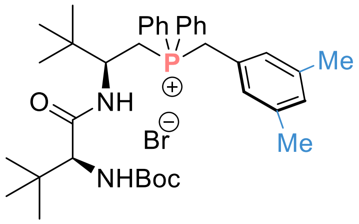

P5

White powder; m.p. = 235.2-236.7 °C; ^{1}H NMR (400 MHz, $CDCl_3$) δ 8.59 (d, *J* = 9.4 Hz, 1H), 7.83-7.76 (m, 3H), 7.71 (m, 2H), 7.65 (td, *J* = 7.2, 1.0 Hz, 1H), 7.61-7.54 (m, 2H), 7.47 (td, *J* = 7.9, 3.4 Hz, 2H), 7.07 (d, *J* = 11.2 Hz, 1H), 6.66 (s, 1H), 6.34 (s, 2H), 5.15 (dt, *J* = 14.7, 11.5 Hz, 1H), 4.97 (t, *J* = 15.0 Hz, 1H), 4.30 (d, *J* = 11.2 Hz, 1H), 4.03 (dd, *J* = 15.2, 12.9 Hz, 1H), 4.01-3.91 (ddd, *J* = 9.4, 9.9, 15.2 Hz, 1H), 2.40 (t, *J* = 14.7 Hz, 1H), 1.96 (s, 6H), 1.06 (s, 9H), 1.02 (s, 9H), 0.84 (s, 9H); ^{13}C NMR (100 MHz, $CDCl_3$) δ 171.67, 156.18, 138.26 (d, *J* = 3.5 Hz), 135.12 (d, *J* = 2.8 Hz), 134.46 (d, *J* = 2.7 Hz), 133.93 (d, *J* = 8.7 Hz), 133.66 (d, *J* = 8.9 Hz), 130.36 (d, *J* = 12.0 Hz), 129.56 (d, *J* = 12.3 Hz), 129.55 (d, *J* = 4.0 Hz), 128.61 (d, *J* = 5.8 Hz), 127.54 (d, *J* = 9.6 Hz), 118.21 (d, *J* = 86.4 Hz), 116.61 (d, *J* = 81.4 Hz), 79.00, 65.93, 52.11 (d, *J* = 4.7 Hz), 36.89 (d, *J* = 12.4 Hz), 34.69, 27.89, 27.87 (d, *J* = 42.2 Hz), 27.10, 26.59, 26.00 (d, *J* = 47.9 Hz), 20.94. ^{31}P NMR (162 MHz, $CDCl_3$) δ 29.32; HRMS (ESI) *m/z* calcd for $C_{38}H_{54}BrN_2O_3P$ $[M-Br]^+$ =617.3872, found = 617.3872.

((*S*)-2-((*S*)-2-((tert-butoxycarbonyl)amino)-3,3-dimethylbutanamido)-3,3-dimethylbutyl)(3-methoxybenzyl)diphenylphosphonium bromide (P6)

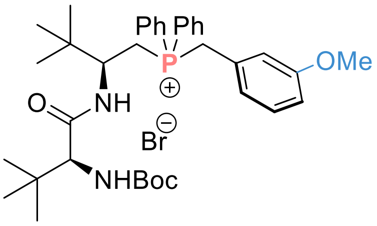

P6

White powder; m.p. = 203.5-204.6 °C; ^{1}H NMR (400 MHz, $CDCl_3$) δ 8.53 (d, *J* = 9.2 Hz, 1H), 7.84-7.75 (m, 3H), 7.70 (td, *J* = 7.7, 3.3 Hz, 2H), 7.67-7.55 (m, 3H), 7.45 (td, *J* = 7.7, 3.3 Hz, 2H), 7.00 (d, *J* = 11.1 Hz, 1H), 6.88 (t, *J* = 7.9 Hz, 1H), 6.58 (d, *J* = 8.2 Hz, 1H), 6.45 (d, *J* = 7.4 Hz, 1H), 6.27 (d, *J* = 1.5 Hz, 1H), 5.10 (dt, *J* = 14.3, 11.9 Hz, 1H), 5.07 (t, *J* = 15.0 Hz, 1H), 4.27 (d, *J* = 11.1 Hz, 1H), 4.11 (dd, *J* = 15.0, 13.3 Hz, 1H), 3.96 (dt, *J* = 15.3, 10.2 Hz, 1H), 3.42 (s, 3H), 2.42 (t, *J* = 14.7 Hz, 1H), 1.03 (s, 18H), 0.82 (s, 9H); ^{13}C NMR (100 MHz, $CDCl_3$) δ 171.58, 159.57 (d, *J* = 3.5 Hz), 156.13, 135.19 (d, *J* = 2.8 Hz), 134.55 (d, *J* = 2.6 Hz), 133.77 (d, *J* = 8.8 Hz), 133.58 (d, *J* = 8.9 Hz), 130.41 (d, *J* = 12.1 Hz), 129.73 (d, *J* = 5.0 Hz), 129.70 (d, *J* = 12.2 Hz), 129.36 (d, *J* = 9.6 Hz), 122.98 (d, *J* = 5.9 Hz), 118.41 (s), 116.36 (d, *J* = 81.7 Hz), 115.51 (d, *J* = 5.6 Hz), 114.53 (d, *J* = 3.8 Hz), 79.04, 65.74, 55.17, 52.05 (d, *J* = 4.7 Hz), 36.87 (d, *J* = 12.4 Hz), 34,63, 27.94 (d, *J* = 43.0 Hz), 27.92, 27.03, 26.52, 25.94 (d, *J* = 48.3 Hz); ^{31}P NMR (162 MHz, $CDCl_3$) δ 29.33; HRMS (ESI) *m/z* calcd for $C_{37}H_{52}BrN_2O_4P$ $[M-Br]^+$ = 619.3665, found = 619.3667.

((*S*)-2-((*S*)-2-((tert-butoxycarbonyl)amino)-3,3-dimethylbutanamido)-3,3-dimethylbutyl)(naphthalen-1-ylmethyl)diphenylphosphoniumbromide (P7)

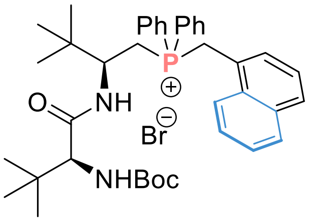

P7

White powder; m.p. = 244.4-245.7 °C; ^{1}H NMR (400 MHz, $CDCl_3$) δ 8.89 (d, *J* = 9.2 Hz, 1H), 8.04 (d, *J* = 7.9 Hz, 1H), 7.85-7.78 (m, 3H), 7.69 (td, *J* = 7.9, 3.2 Hz, 2H), 7.59 (dd, *J* = 8.1, 2.4 Hz, 1H), 7.55-7.51 (m, 1H), 7.25-7.11 (m, 8H), 7.02 (td, *J* = 7.9, 3.5 Hz, 2H), 5.67 (dd, *J* = 15.3, 13.7 Hz, 1H), 5.36 (dt, *J* = 14.4, 11.7 Hz, 1H), 4.38 (d, *J* = 11.2 Hz, 1H), 4.37 (t, *J* = 14.7 Hz, 1H), 4.12 (ddd, *J* = 14.7, 10.2, 10.1 Hz, 1H),

2.39 (t, J = 14.5 Hz, 1H), 1.07 (s, 9H), 0.87 (s, 9H), 0.61 (s, 9H); ^{13}C NMR (100 MHz, $CDCl_3$) δ 171.86, 156.11, 135.07 (d, J = 2.9 Hz), 134.28 (d, J = 2.9 Hz), 133.70 (d, J = 9.2 Hz), 133.59 (d, J = 8.6 Hz), 133.40 (d, J = 3.0 Hz), 132.35 (d, J = 4.4 Hz), 130.30 (d, J = 12.1 Hz), 129.16 (d, J = 4.5 Hz), 128.93 (d, J = 12.1 Hz), 128.78, 127.82, 126.84, 125.87, 125.21 (d, J = 1.5 Hz), 124.74 (d, J = 5.1 Hz), 124.71, 117.03 (d, J = 82.0 Hz), 116.83 (d, J = 85.4 Hz), 78.87, 66.07, 52.10 (d, J = 4.7 Hz), 36.91 (d, J = 12.3 Hz), 34.69, 27.42, 27.12, 26.63, 25.64 (d, J = 46.9 Hz), 24.57 (d, J = 43.0 Hz); ^{31}P NMR (162 MHz, $CDCl_3$) δ 27.55; HRMS (ESI) *m/z* calcd for $C_{40}H_{52}BrN_2O_3P$ $[M\text{-}Br]^+$ = 639.3716, found = 6339.3720.

((*S*)-2-((*S*)-2-((tert-butoxycarbonyl)amino)-3,3-dimethylbutanamido)-3,3-dimethylbutyl)diphenyl(2,4,6-trimethylbenzyl)phosphonium bromide (P8)

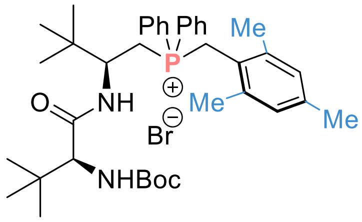

P8

White powder; m.p. = 213.2-214.9 °C; ^{1}H NMR (400 MHz, $CDCl_3$) δ 9.08 (d, J = 9.1 Hz, 1H), 7.76-7.69 (m, 1H), 7.68-7.61 (m, 1H), 7.59-7.56 (m, 4H), 7.44-7.39 (m, 4H), 7.23 (d, J = 11.4 Hz, 1H), 6.54 (s, 2H), 4.84 (t, J = 15.0 Hz, 1H), 4.77 (dt, J = 14.6, 11.5 Hz, 1H), 4.42 – 4.36 (m, 1H), 4.33 (d, J = 11.4 Hz, 1H), 4.18 (t, J = 14.2 Hz 1H), 2.52 (t, J = 14.6 Hz, 1H), 2.09 (s, 3H), 1.79 (s, 6H), 1.04 (s, 9H), 0.92 (s, 9H), 0.91 (s, 9H); ^{13}C NMR (100 MHz, $CDCl_3$) δ 171.71, 156.01, 138.57, 137.98 (d, J = 4.4 Hz), 135.00 (d, J = 2.8 Hz), 134.64 (d, J = 2.8 Hz), 133.91 (d, J = 9.0 Hz), 133.72 (d, J = 8.5 Hz), 129.82 (d, J = 11.9 Hz), 129.57 (d, J = 3.7 Hz), 129.36 (d, J = 11.9 Hz), 122.85 (d, J = 10.5 Hz), 118.07 (d, J = 84.1 Hz), 117.86 (d, J = 78.2 Hz), 78.95, 65.93, 52.15 (d, J = 5.2 Hz), 37.37 (d, J = 12.1 Hz), 34.76, 27.64, 27.16, 26.81, 25.56, 24.90 (d, J = 40.6 Hz), 21.80, 20.73 (d, J = 1.3 Hz); ^{31}P NMR (162 MHz, $CDCl_3$) δ 21.44; HRMS (ESI) *m/z* calcd for $C_{39}H_{56}BrN_2O_3P$ $[M\text{-}Br]^+$ = 631.4029, found = 631.4023.

((*S*)-2-((*S*)-2-((tert-butoxycarbonyl)amino)-N,3,3-trimethylbutanamido)-3,3-dimethylbutyl)diphenyl(2,4,6-trimethylbenzyl)phosphonium bromide (P8-1)

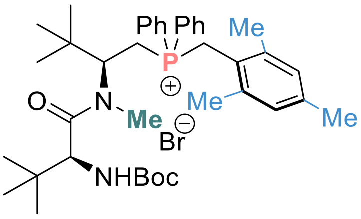

P8-1

White solid; m.p. = 126.8-127.5 °C; ^{1}H NMR (400 MHz, $CDCl_3$) δ 7.94-7.85 (m, 2H), 7.79-7.74 (m, 2H), 7.68-7.66 (m, 2H), 7.55-7.43 (m, 5H), 6.54 (s, 2H), 4.98 (d, *J* = 10.4 Hz, 1H), 4.84-4.71 (m, 1H), 4.69-4.56 (m, 1H), 4.43-4.28 (m, 2H), 3.34 (s, 3H), 3.15 (t, *J* = 15.8 Hz, 1H), 2.08 (s, 3H), 1.85 (s, 6H), 1.31 (s, 9H), 0.75 (s, 9H), 0.71 (s, 9H); ^{13}C NMR (100 MHz, $CDCl_3$) δ 175.09, 155.47, 137.87 (d, *J* = 5.5 Hz), 134.92 (d, *J* = 2.7 Hz), 134.86 (d, *J* = 2.1 Hz), 134.03 (d, *J* = 9.2 Hz), 129.68 (d, *J* = 12.0 Hz), 129.56, 129.49 (d, *J* = 10.0 Hz), 122.59 (d, *J* = 9.5 Hz), 117.82 (d, *J* = 79.6 Hz), 116.02 (d, *J* = 82.6 Hz), 79.73, 56.58, 54.69 (d, *J* = 4.5 Hz), 37.84 (d, *J* = 11.4 Hz), 34.29 (d, *J* = 29.1 Hz), 28.24, 27.26, 27.02, 26.40 (d, *J* = 33.8 Hz), 26.38, 24.07 (d, *J* = 46.6 Hz), 21.61 (s), 20.75 (s); ^{31}P NMR (162 MHz, $CDCl_3$) δ 26.58; HRMS (ESI) *m/z* calcd for $C_{40}H_{58}BrN_2O_3P$ $[M-Br]^+$ = 645.4185, found = 645.4188.

((*S*)-2-((*S*)-2-((tert-butoxycarbonyl)(methyl)amino)-3,3-dimethylbutanamido)-3,3-dimethylbutyl)diphenyl(2,4,6-trimethylbenzyl)phosphonium bromide (P8-2)

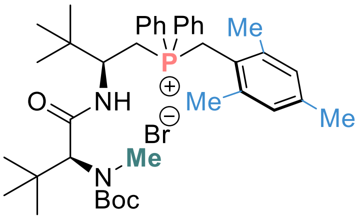

P8-2

White solid; m.p. = 148.3-149.5 °C; 2:1 *d.r.*; ^{1}H NMR (400 MHz, $CDCl_3$) δ 7.78-7.62 (m, 5H), 7.62-7.50 (m, 4H), 7.50-7.37 (m, 2H), 6.57 (d, *J* = 16.9 Hz, 2H), 5.22- 4.93 (m, 1H), 4.88-4.72 (m, 1H), 4.72-4.52 (m, 1H), 4.51-4.30 (m, 1H), 4.20-4.07 (m, 1H), 3.06 (s, 3H), 2.50 (t, *J* = 15.0 Hz, 1H), 2.10 (s, 3H), 1.80 (s, 6H), 1.49 (s, 9H), 0.98 (s, 9H), 0.85 (s, 9H); ^{13}C NMR (100 MHz, $CDCl_3$) δ 171.13, 156.29, 138.40 (d, *J* = 4.3 Hz), 137.87 (d, *J* = 4.5 Hz), 134.67, 134.17, 133.80 (d, *J* = 9.8 Hz), 133.69, 129.63 (d,

J = 3.1 Hz), 129.54, 129.42, 121.96 (d, J = 10.4 Hz), 118.10 (d, J = 78.7 Hz), 117.68 (d, J = 83.1 Hz), 80.91, 65.49, 50.66 (d, J = 5.0 Hz), 37.37 (d, J = 11.9 Hz), 35.94, 32.61, 28.67, 28.37, 27.77, 25.97, 25.05 (d, J = 21.8 Hz), 24.41 (d, J = 22.5 Hz), 21.46; ^{31}P NMR (162 MHz, $CDCl_3$) δ 21.89, 21.65; HRMS (ESI) *m/z* calcd for $C_{40}H_{58}BrN_2O_3P$ [M-Br]$^+$ = 645.4185, found = 645.4180.

(*S*)-(2-(3,5-bis(trifluoromethyl)benzamido)-3,3-dimethylbutyl)diphenyl(2,4,6-trimethylbenzyl)phosphonium bromide (P9)

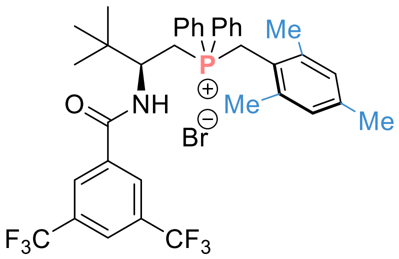

P9

White solid; m.p. = 134.0-135.5 °C; ^{1}H NMR (400 MHz, $CDCl_3$) δ 9.32 (d, J = 9.2 Hz, 1H), 8.49 (s, 2H), 7.97-7.90 (m, 2H), 7.87 (s, 1H), 7.67 (td, J = 7.1, 1.2 Hz, 1H), 7.59-7.52 (m, 2H), 7.48 (td, J = 7.8, 3.5 Hz, 2H), 7.33-7.27 (m, 3H), 6.60 (s, 2H), 5.25 (dt, J = 15.6, 11.5 Hz, 1H), 4.56-4.45 (m, 1H), 4.47-4.28 (m, 2H), 2.95 (dd, J = 15.2, 13.5 Hz, 1H), 2.10 (d, J = 2.6 Hz, 3H), 1.82 (s, 6H), 1.03 (s, 9H); ^{13}C NMR (100 MHz, $CDCl_3$) δ 164.34, 138.24 (d, J = 4.2 Hz), 137.75 (d, J = 5.4 Hz), 134.93 (d, J = 2.9 Hz), 134.87, 134.47 (d, J = 9.7 Hz), 134.29 (d, J = 2.9 Hz), 133.86 (d, J = 8.5 Hz), 131.34 (q, J = 33.8 Hz), 129.77, 129.70 (d, J = 9.4 Hz), 129.42 (d, J = 12.3 Hz), 128.90 (d, J = 2.3 Hz), 124.82 (dt, J = 7.6, 3.7 Hz), 123.14 (q, J = 273.1 Hz), 122.23 (d, J = 9.2 Hz), 117.14 (d, J = 80.0 Hz), 116.58 (d, J = 81.9 Hz), 52.69 (d, J = 6.1 Hz), 37.76 (d, J = 11.6 Hz), 27.18 (d, J = 46.3 Hz), 26.63, 24.06 (d, J = 47.3 Hz), 21.34 (d, J = 1.4 Hz), 20.74; ^{31}P NMR (162 MHz, $CDCl_3$) δ 22.87; ^{19}F NMR (376 MHz, $CDCl_3$) δ -62.45; HRMS (ESI) *m/z* calcd for $C_{37}H_{39}BrF_6NOP$ [M-Br]$^+$ = 658.2674, found = 658.2671.

5. Preparation of Cyclic Sulfoximines

General procedure A : the synthesis of **A1-A4**

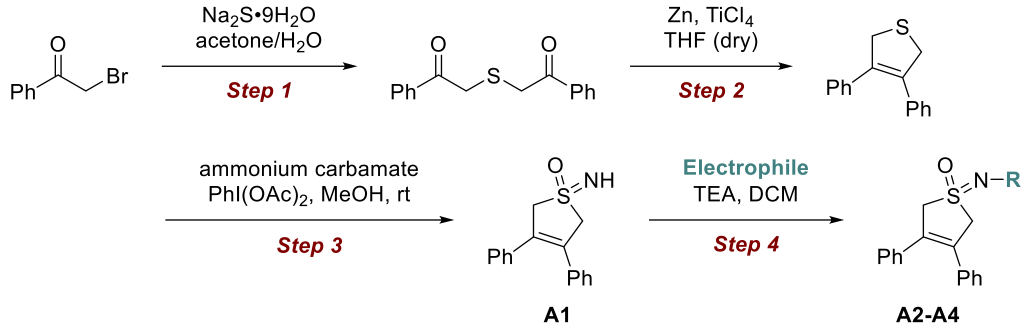

Supplementary Fig. 2. General procedure A for synthesis of **A1-A4**

Step 1: According to modified literature procedure[10], to a stirred solution of bromoketone (2.0 equiv.) in acetone (200 mL) was added a solution of Na_2S•H_2O (20 mmol) in H_2O (50 mL) at 0 °C. After completion of the addition, the mixture was warmed to room temperature and stirred for 2.5 h. The majority of the acetone was removed under reduced pressure, and the remaining aqueous mixture was extracted three times with dichloromethane. The combined organic extracts were dried over anhydrous magnesium sulfate, filtered and concentrated under reduced pressure. The corresponding open-chain thioether was used in the next synthetic step without further purification.

Step 2: To a suspension of Zn powder (8.0 equiv.) in dry THF (150 mL) was dropwise added $TiCl_4$ (4.0 equiv.) at -30 ℃, the mixture was refluxed for 2.5 h. A solution of open-chain thioether (19 mmol) in dry THF (80 mL) was slowly added to the reaction mixture at 0 ℃. The reaction was refluxed for 2 h and quenched with 10% K_2CO_3 aqueous solution (40 mL) at 0 ℃. Then five spoons of anhydrous magnesium sulfate were added to the mixture to stir for 10 min. The insoluble materials were filtered through Celite. The majority of the THF was removed under reduced pressure and then the residues were extracted three times with CH_2Cl_2. The combined organic extracts were dried over anhydrous magnesium sulfate, filtered and concentrated under reduced pressure. The crude cyclic thioether was obtained after once recrystallization under the solution of hexane and ethyl acetate.

Step 3: To a 250 mL round-bottom flask with a magnetic stirring bar were added the crude cyclic thioether (10 mmol), ammonium carbamate (2.0 equiv.) and iodobenzene diacetate (2.5 equiv.). The mixture was dissolved in methanol (100 mL), and then stirred at r.t. for 6 h. The solvent was removed in vacuo, and the residue was purified by column chromatography on silica gel (PE/EA= 1/1) to afford cyclic sulfoximine **A1** (46% yield) as a yellow solid[11].

Step 4: To a stirred solution of **A1** (5 mmol) in CH_2Cl_2 (20 mL) was added TEA (3.0 equiv.) and electrophile (2.0 equiv.) at 0 °C. The reaction was removed to r.t. and stirred for 2 h. The solvent was removed in vacuo, and the residue was purified by column chromatography on silica gel (PE/EA= 5/1) to afford cyclic sulfoximine **A2-A4**.

General procedure B: the synthesis of **A5-A43**

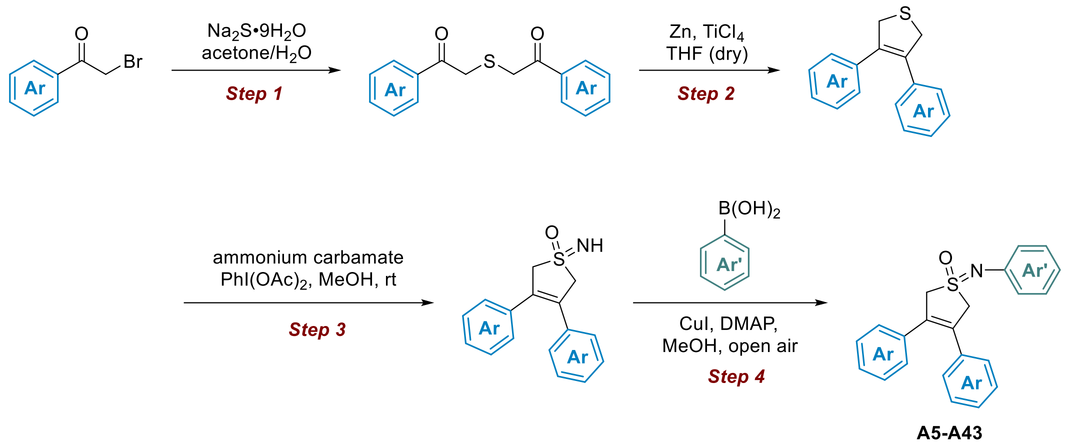

Supplementary Fig. 3. General procedure B for synthesis of **A5-A43**

The **Step 1**, **Step 2** were the same as **General procedure A**.

Step 3: To a 250 mL round-bottom flask with a magnetic stirring bar were added the crude cyclic thioether (1.0 equiv.), ammonium carbamate (2.0 equiv.) and iodobenzene diacetate (2.5 equiv.). The mixture was dissolved in methanol (100 mL), and then stirred at r.t. for 6 h. The solvent was removed in vacuo, and the residue was purified by a flash column chromatography on silica gel (PE/EA= 1/1) to afford crude cyclic sulfoximine, which were directly used in the next synthetic step.

Step 4: According to modified literature procedure[12], a mixture of the crude cyclic sulfoximines (1.0 equiv.), CuI (10 mol%) and DMAP (1.0 equiv.) was stirred in meOH

(5 mL) under open air at r.t. for 5 min. The arylboronic acid (1.5 equiv.) was added to the reaction mixture and allowed to stir at r.t.. The progress of reaction was monitored by TLC. After completion, the solvent was removed in vacuo, and the residue was purified by column chromatography on silica gel (PE/EA= 5/1) to afford N-protected cyclic sulfoximines **A5-A43**.

General procedure C : the synthesis of **A44-A45**

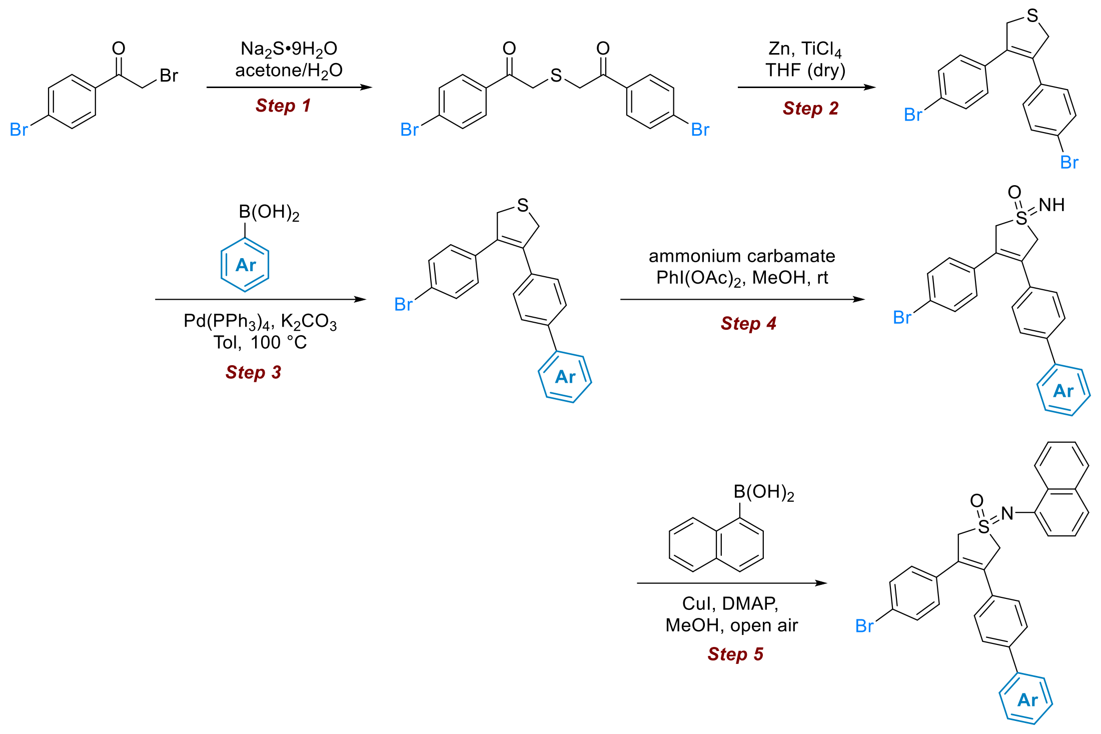

A44, A45

Supplementary Fig. 4. General procedure C for synthesis of **A44-A45**

The **Step 1**, **Step 2** were the same as **General procedure A**.

Step 3: To a 50 mL Schlenk tube with a magnetic stirring bar were added the crude symmetric cyclic thioether (1.0 equiv.), arylboronic acid (1.1 equiv.) and $Pd(PPh_3)_4$ (10 mol%), K_2CO_3 (2.0 equiv.). The mixture was dissolved in toluene (10 mL), and then the reaction was refluxed overnight under N_2 atmosphere. After completion, the reaction mixture was diluted with CH_2Cl_2 and washed with H_2O. The organic layer was dried over anhydrous magnesium sulfate and the solvent was removed in vacuo. The residue was purified by a flash column chromatography on silica gel (PE/EA = 50/1) to

afford the crude non-symmetric sulfoximines, which weredirectly used in the next synthetic step.

The **Step 4** , **Step 5** were the same as **General procedure B**. Finally, the racemic cyclic sulfoximines **A44**, **A45** were obtained.

General procedure D : the synthesis of **A46-A49**

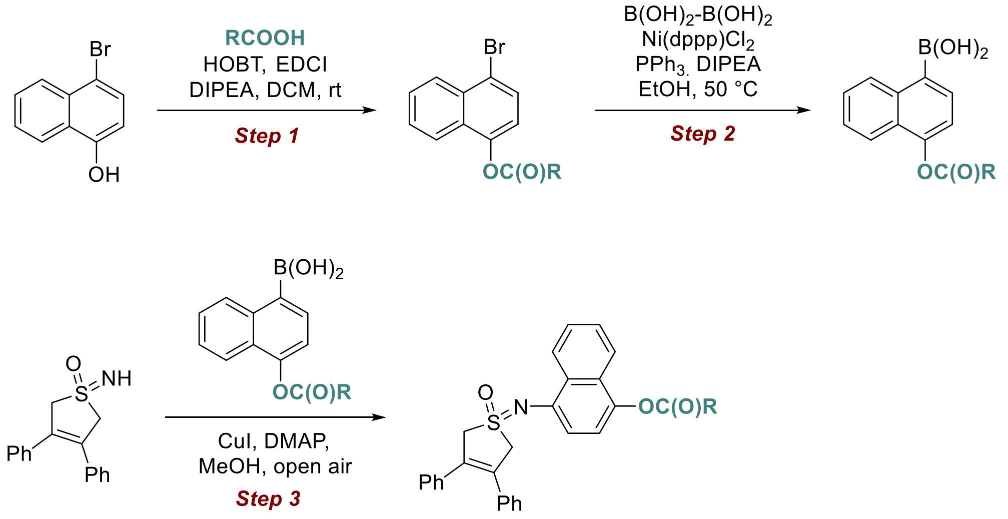

A1 **A46-A49**

Supplementary Fig. 5. General procedure D for synthesis of **A46-A49**

Step 1: To a 250 mL round-bottom flask with a magnetic stirring bar were added 4-bromo-L-naphthol (1.0 equiv.), indicated acid (1.2 equiv.), HOBT (1.2 equiv.), EDCI (1.2 equiv.), DIPEA (2.4 equiv.). The mixture was dissolved in CH_2Cl_2 (100 mL), and then stirred at r.t. for 4 h. The solvent was removed in vacuo, and the residue was purified by a flash column chromatography on silica gel (PE/EA= 5/1) to afford corresponding aryl carboxylate to be directly used in the next synthetic step.

Step 2: According to modified literature procedure[13], to a 50 mL Schlenk tube with a magnetic stirring bar were added aryl carboxylate (1.0 equiv.), Ni(dppp)Cl_2 (3 mol%), PPh_3 (6 mol%), and $(OH)_2B-B(OH)_2$ (1.5 equiv.). The Schlenk tube was capped and then evacuated and backfilled with N_2. EtOH (10 mL) was added via syringe, which was followed by the addition of DIPEA (3.0 equiv.). The reaction was then heated at 50 ℃ overnight. After completion, the reaction was quenched with 1M HCl (20 mL), and extracted with CH_2Cl_2. The solvent was removed in vacuo, and the residue was

purified by a flash column chromatography on silica gel (EA/MeOH = 50/1) to afford corresponding arylboronic acid, which was directly used in the next synthetic step. The **Step 3** was the same as **General procedure B**. Finally, the cyclic sulfoximines bearng bioactive molecules or material building blocks **A46-A49** were obtained.

All the unknown compounds **A1-A49** were fully characterized.

1-imino-3,4-diphenyl-2,5-dihydro-1*H*-1λ⁶-thiophene 1-oxide (A1)

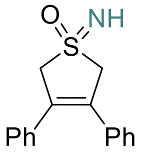

A1

Light yellow solid; 2.3 g, 42% total yield; m.p. = 123.4-125.8 °C; ^{1}H NMR (400 MHz, $CDCl_3$) δ 7.25-7.20 (m, 6H), 7.13-7.05 (m, 4H), 4.41 (s, 4H), 3.01 (br, 1H); ^{13}C NMR (100 MHz, $CDCl_3$) δ 135.09, 131.42, 128.68, 128.58, 128.37, 65.36; HRMS (ESI) *m/z* calcd for $C_{16}H_{15}NOS$ $[M+H]^+$ = 270.0952, found = 270.0953.

4-methyl-*N*-(1-oxido-3,4-diphenyl-2,5-dihydro-1λ⁶-thiophen-1-ylidene)benzenesulfonamide (A2)

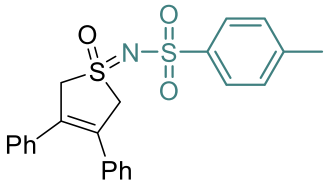

A2

White solid; 3.2 g, 38% total yield; m.p. = 53.5-54.0 °C; ^{1}H NMR (400 MHz, $CDCl_3$) δ 7.91 (d, *J* = 8.2 Hz, 2H), 7.30 (d, *J* = 8.2 Hz, 2H), 7.28-7.22 (m, 6H), 7.11-7.06 (m, 4H), 4.99 (d, *J* = 15.7 Hz, 2H), 4.55 (d, *J* = 15.7 Hz, 2H), 2.42 (s, 3H); ^{13}C NMR (100 MHz, $CDCl_3$) δ 143.35, 140.32, 133.82, 130.52, 129.55, 128.95, 128.89, 128.57, 126.88, 63.96, 21.70; HRMS (ESI) *m/z* calcd for $C_{23}H_{21}NO_3S_2$ $[M+Na]^+$ = 446.0861, found = 446.0860.

***N*-(1-oxido-3,4-diphenyl-2,5-dihydro-1λ⁶-thiophen-1-ylidene)benzamide (A3)**

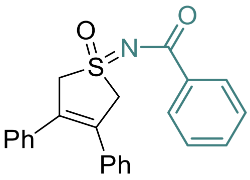

A3

White solid; 2.9 g, 39% total yield; m.p. = 100.3-101.9 °C; ^{1}H NMR (400 MHz, $CDCl_3$) δ 8.22-8.17 (m, 2H), 7.54 (t, *J* = 7.3 Hz, 1H), 7.44 (t, *J* = 7.6 Hz, 2H), 7.29-7.23 (m, 6H), 7.18-7.11 (m, 4H), 5.00 (d, *J* = 15.8 Hz, 2H), 4.61 (d, *J* = 15.8 Hz, 2H); ^{13}C NMR (100 MHz, $CDCl_3$) δ 175.33, 135.01, 134.29, 132.52, 130.66, 129.55, 128.81, 128.74, 128.62, 128.24, 62.25; HRMS (ESI) *m/z* calcd for $C_{23}H_{19}NO_2S$ $[M+Na]^+$ = 396.1034, found = 396.1033.

1-(3,5-bis(trifluoromethyl)phenyl)-3-(1-oxido-3,4-diphenyl-2,5-dihydro-1λ^6-thiophen-1-ylidene)thiourea (A4)

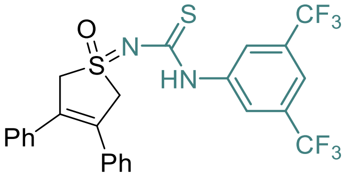

A4

White solid; 3.7 g, 34% total yield; m.p. = 184.2-184.5 °C; ^{1}H NMR (400 MHz, $CDCl_3$) δ 8.91 (br, 1H), 8.01 (s, 2H), 7.61 (s, 1H), 7.38-7.19 (m, 6H), 7.18-7.05 (m, 4H), 5.16 (d, *J* = 16.6 Hz, 2H), 4.73 (d, *J* = 16.6 Hz, 2H); ^{13}C NMR (100 MHz, $CDCl_3$) δ 186.99, 139.96, 133.94, 132.20 (q, *J* = 33.5 Hz), 130.45, 128.95, 128.91, 128.57, 123.19 (q, *J* = 272.8 Hz), 62.79. ^{19}F NMR (376 MHz, $CDCl_3$) δ -62.97; HRMS (ESI) *m/z* calcd for $C_{25}H_{18}F_6N_2OS_2$ $[M+Na]^+$ = 563.0663, found = 563.0665.

1-(naphthalen-1-ylimino)-3,4-diphenyl-2,5-dihydro-1*H*-1λ^6-thiophene 1-oxide (A5)

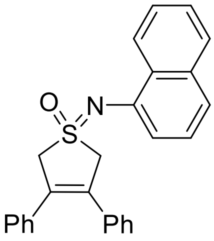

A5

Yellow solid; 2.0 g, 25% total yield; m.p. = 69.2-70.7 °C; ^{1}H NMR (400 MHz, $CDCl_3$) δ 8.44-8.37 (m, 1H), 7.81 (m, 1H), 7.53 (d, *J* = 8.2 Hz, 1H), 7.50-7.43 (m, 2H), 7.38 (t, *J* = 7.7 Hz, 1H), 7.31 (dd, *J* = 7.3, 0.9 Hz, 1H), 7.26-7.20 (m, 6H), 7.14-7.08 (m, 4H), 4.76-4.60 (m, 2H), 4.60-4.49 (m, 2H); ^{13}C NMR (100 MHz, $CDCl_3$) δ 141.77, 135.02, 134.86, 131.55, 130.32, 128.75, 128.63, 128.50, 127.93, 126.27, 126.20, 125.35, 124.08, 122.47, 116.44, 62.51; HRMS (APCI) *m/z* calcd for $C_{26}H_{21}NOS$ $[M+H]^+$ = 396.1422, found = 396.1417.

3,4-bis(4-methoxyphenyl)-1-(naphthalen-1-ylimino)-2,5-dihydro-1*H*-1λ^6-thiophene 1-oxide (A6)

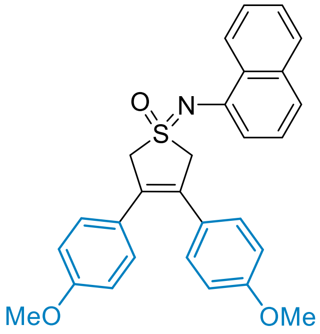

A6

Yellow solid; 1.8 g, 20% total yield; m.p. = 50.0-51.9 °C; ^{1}H NMR (400 MHz, $CDCl_3$) δ 8.44-8.35 (m, 1H), 7.84-7.77 (m, 1H), 7.52 (d, *J* = 8.2 Hz, 1H), 7.49-7.41 (m, 2H), 7.37 (t, *J* = 7.7 Hz, 1H), 7.30 (dd, *J* = 7.3, 0.8 Hz, 1H), 7.10-7.01 (m, 4H), 6.79-6.74 (m, 4H), 4.77-4.58 (m, 2H), 4.58-4.38 (m, 2H), 3.78 (s, 6H); ^{13}C NMR (100 MHz, $CDCl_3$) δ 159.48, 141.93, 134.81, 130.27, 129.91, 129.71, 127.88, 127.46, 126.24, 126.20, 125.29, 124.10, 122.30, 116.28, 114.12, 62.46, 55.36; HRMS (ESI) *m/z* calcd for $C_{28}H_{25}NO_3S$ $[M+H]^+$ = 456.1633, found = 456.1630.

1-(naphthalen-1-ylimino)-3,4-di-p-tolyl-2,5-dihydro-1*H*-1λ^6-thiophene 1-oxide (A7)

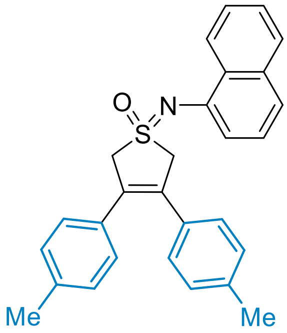

A7

Light yellow solid; 3.0 g, 36% total yield; m.p. = 132.4-133.6 °C; ^{1}H NMR (400 MHz, $CDCl_3$) δ 8.46-8.36 (m, 1H), 7.81 (dd, *J* = 6.8, 2.5 Hz, 1H), 7.53 (d, *J* = 8.1 Hz, 1H), 7.50-7.42 (m, 2H), 7.38 (t, *J* = 7.7 Hz, 1H), 7.31 (dd, *J* = 7.3, 1.0 Hz, 1H), 7.07-6.98 (m, 8H), 4.73-4.63 (m, 2H), 4.55-4.46 (m, 2H), 2.31 (s, 6H); ^{13}C NMR (100 MHz, $CDCl_3$) δ 141.88, 138.35, 134.81, 132.19, 130.72, 130.28, 129.40, 128.47, 127.88, 126.23, 126.18, 125.30, 124.10, 122.33, 116.30, 62.52, 21.36; HRMS (APCI) *m/z* calcd for $C_{28}H_{25}NOS$ $[M+H]^+$ = 424.1735, found = 424.1734.

<u>3,4-bis(4-chlorophenyl)-1-(naphthalen-1-ylimino)-2,5-dihydro-1*H*-1λ^6-thiophene 1-oxide (A8)</u>

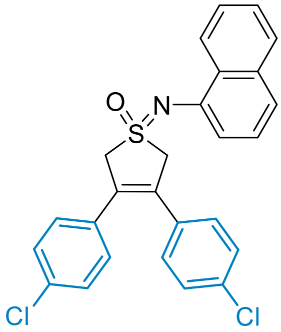

A8

Yellow solid; 1.2 g, 13% total yield; m.p. = 42.2-43.1 °C; ^{1}H NMR (400 MHz, $CDCl_3$) δ 8.35 (d, *J* = 7.9 Hz, 1H), 7.85-7.77 (m, 1H), 7.54 (d, *J* = 8.2 Hz, 1H), 7.51-7.41 (m, 2H), 7.37 (t, *J* = 7.8 Hz, 1H), 7.30-7.27 (m, 1H), 7.25-7.20 (m, 4H), 7.05-6.97 (m, 4H), 4.71-4.56 (m, 2H), 4.56-4.42 (m, 2H); ^{13}C NMR (100 MHz, $CDCl_3$) δ 141.46, 134.83, 134.74, 133.04, 131.14, 130.18, 129.90, 129.21, 128.00, 126.34, 126.19, 125.42, 123.91, 122.66, 116.46, 62.19; HRMS (APCI) *m/z* calcd for $C_{26}H_{19}Cl_2NOS$ $[M+H]^+$ = 464.0642, found = 464.0637.

3,4-bis(4-fluorophenyl)-1-(naphthalen-1-ylimino)-2,5-dihydro-1*H*-1λ^6-thiophene 1-oxide (A9)

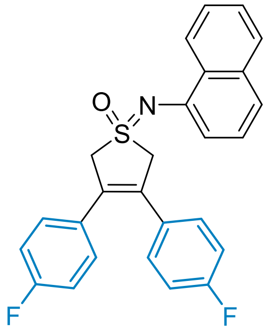

A9

Yellow oil; 603 mg, 7% total yield; ^{1}H NMR (400 MHz, $CDCl_3$) δ 8.42-8.33 (m, 1H), 7.85-7.76 (m, 1H), 7.54 (d, *J* = 8.2 Hz, 1H), 7.51-7.42 (m, 2H), 7.38 (t, *J* = 7.8 Hz, 1H), 7.30 (d, *J* = 6.9 Hz, 1H), 7.10-7.03 (m, 4H), 6.99-6.90 (m, 4H), 4.69-4.61 (m, 2H), 4.54-4.46 (m, 2H); ^{13}C NMR (100 MHz, $CDCl_3$) δ 162.55 (d, *J* = 249.6 Hz), 141.57, 134.83, 130.75, 130.71, 130.48, 130.40, 130.20, 127.98, 126.26 (d, *J* = 12.5 Hz), 125.38, 123.95, 122.58, 116.40, 116.00 (d, *J* = 21.7 Hz), 62.33; ^{19}F NMR (376 MHz, $CDCl_3$) δ -111.81; HRMS (ESI) *m/z* calcd for $C_{26}H_{19}F_2NOS$ $[M+H]^+$ = 432.1233 found = 432.1231.

3,4-bis(3-methoxyphenyl)-1-(naphthalen-1-ylimino)-2,5-dihydro-1*H*-1λ^6-thiophene 1-oxide (A10)

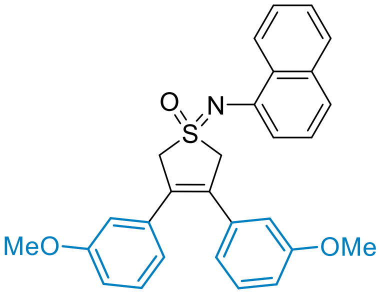

A10

Yellow oil; 455 mg, 5% total yield; ^{1}H NMR (400 MHz, $CDCl_3$) δ 8.43-8.35 (m, 1H), 7.84-7.77 (m, 1H), 7.53 (d, *J* = 8.2 Hz, 1H), 7.51-7.42 (m, 2H), 7.38 (t, *J* = 7.8 Hz, 1H), 7.30 (dd, *J* = 7.3, 0.7 Hz, 1H), 7.16 (t, *J* = 8.1 Hz, 2H), 6.79 (dd, *J* = 8.1, 2.2 Hz, 2H), 6.72 (d, *J* = 7.7 Hz, 2H), 6.66-6.61 (m, 2H), 4.79-4.58 (m, 2H), 4.58-4.46 (m, 2H), 3.62 (s, 6H); ^{13}C NMR (100 MHz, $CDCl_3$) δ 159.66, 141.74, 136.25, 134.83, 131.49, 130.26,

129.83, 127.93, 126.27, 126.19, 125.35, 124.05, 122.46, 120.88, 116.38, 114.44, 113.91, 62.42, 55.27; HRMS (ESI) *m/z* calcd for $C_{28}H_{25}NO_3S$ $[M+H]^+$ = 456.1633, found = 456.1630.

3,4-bis(3-chlorophenyl)-1-(naphthalen-1-ylimino)-2,5-dihydro-1*H*-1λ^6-thiophene 1-oxide (A11)

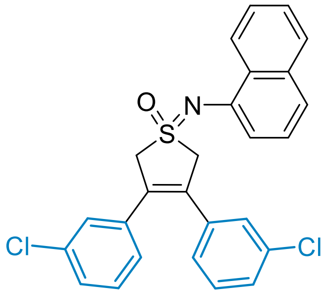

A11

Yellow oil; 833 mg, 9% total yield; ^{1}H NMR (400 MHz, $CDCl_3$) δ 8.40-8.32 (m, 1H), 7.84-7.78 (m, 1H), 7.55 (d, *J* = 8.2 Hz, 1H), 7.51-7.44 (m, 2H), 7.38 (t, *J* = 7.8 Hz, 1H), 7.31-7.28 (m, 1H), 7.25-7.23 (m, 2H), 7.17 (t, *J* = 7.9 Hz, 2H), 7.12-7.08 (m, 2H), 6.98-6.91 (m, 2H), 4.68-4.60 (m, 2H), 4.53-4.45 (m, 2H); ^{13}C NMR (100 MHz, $CDCl_3$) δ 141.34, 136.23, 134.85, 134.83, 131.50, 130.18, 130.16, 128.99, 128.48, 127.99, 126.86, 126.36, 126.18, 125.48, 123.92, 122.75, 116.57, 62.14; HRMS (APCI) *m/z* calcd for $C_{26}H_{19}Cl_2NOS$ $[M+H]^+$ = 464.0642, found = 464.0637.

3,4-bis(3-fluorophenyl)-1-(naphthalen-1-ylimino)-2,5-dihydro-1*H*-1λ^6-thiophene 1-oxide (A12)

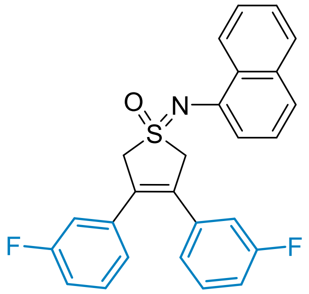

A12

Yellow oil; 603 mg, 7% total yield; ^{1}H NMR (400 MHz, $CDCl_3$) δ 8.42-8.33 (m, 1H), 7.86-7.77 (m, 1H), 7.54 (d, *J* = 8.2 Hz, 1H), 7.51 – 7.43 (m, 2H), 7.38 (t, *J* = 7.3 Hz, 1H), 7.29 (dd, *J* = 7.3, 1.0 Hz, 1H), 7.26-7.19 (m, 2H), 6.97 (tdd, *J* = 8.4, 2.5, 0.8 Hz,

2H), 6.92-6.85 (m, 2H), 6.83-6.76 (m, 2H), 4.70-4.60 (m, 2H), 4.55-4.46 (m, 2H); ^{13}C NMR (100 MHz, $CDCl_3$) δ 162.77 (d, *J* = 247.7 Hz), 141.41, 136.61 (d, *J* = 7.8 Hz), 134.84, 131.51 (d, *J* = 2.2 Hz), 130.59 (d, *J* = 8.4 Hz), 130.20, 127.99, 126.27 (d, *J* = 17.2 Hz), 125.45, 124.32 (d, *J* = 3.0 Hz), 123.92, 122.70, 116.50, 115.99, 115.74 (d, *J* = 8.5 Hz), 115.47, 62.23; ^{19}F NMR (376 MHz, $CDCl_3$) δ -111.53; HRMS (ESI) *m/z* calcd for $C_{26}H_{19}F_2NOS$ $[M+H]^+$ = 432.1233 found = 432.1232.

3,4-bis(2-chlorophenyl)-1-(naphthalen-1-ylimino)-2,5-dihydro-1*H*-1λ^6-thiophene 1-oxide (A13)

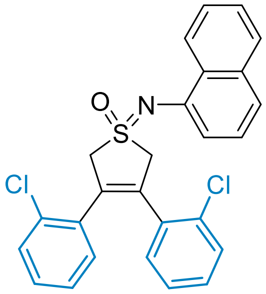

A13

Yellow oil; 2.4 g, 26% total yield; ^{1}H NMR (400 MHz, $CDCl_3$) δ 8.54-8.45 (m, 1H), 7.84-7.76 (m, 1H), 7.52 (d, *J* = 7.3 Hz, 1H), 7.50-7.47 (m, 2H), 7.40-7.33 (m, 2H), 7.32-7.28 (m, 2H), 7.18-7.13 (m, 2H), 7.11-7.03 (m, 4H), 4.66 (d, *J* = 15.4 Hz, 2H), 4.51 (d, *J* = 15.4 Hz, 2H); ^{13}C NMR (100 MHz, $CDCl_3$) δ 141.71, 134.79, 134.01, 133.69, 133.16, 130.53, 130.35, 130.01, 129.75, 127.85, 127.17, 126.23, 126.16, 125.28, 124.32, 122.52, 116.62, 60.17; HRMS (APCI) *m/z* calcd for $C_{26}H_{19}Cl_2NOS$ $[M+H]^+$ = 464.0642, found = 464.0637.

3,4-bis(3,4-dimethoxyphenyl)-1-(naphthalen-1-ylimino)-2,5-dihydro-1*H*-1λ^6-thiophene 1-oxide (A14)

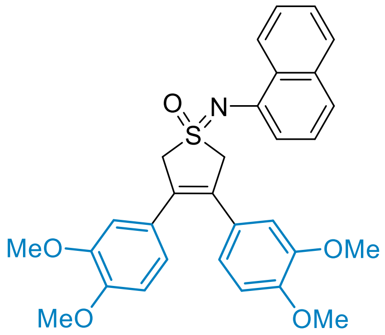

A14

Yellow solid; 1.1 g, 11% total yield; m.p. = 54.3-56.0 °C; ^{1}H NMR (400 MHz, $CDCl_3$) δ 8.46-8.39 (m, 1H), 7.82 (dd, *J* = 7.1, 2.0 Hz, 1H), 7.55 (d, *J* = 8.1 Hz, 1H), 7.51-7.43 (m, 2H), 7.40 (t, *J* = 7.7 Hz, 1H), 7.33 (dd, *J* = 7.3, 0.9 Hz, 1H), 6.80-6.70 (m, 4H), 6.63 (d, *J* = 1.8 Hz, 2H), 4.73-4.65 (m, 2H), 4.57-4.49 (m, 2H), 3.87 (s, 6H), 3.62 (s, 6H); ^{13}C NMR (100 MHz, $CDCl_3$) δ 149.13, 148.84, 141.90, 134.82, 130.24, 130.05, 127.93, 127.69, 126.24, 126.22, 125.26, 124.07, 122.36, 121.21, 116.30, 111.77, 111.14, 62.39, 56.00, 55.83; HRMS (ESI) *m/z* calcd for $C_{30}H_{29}NO_5S$ $[M+H]^+$ = 516.1844, found = 516.1855.

3,4-bis(3,4-difluorophenyl)-1-(naphthalen-1-ylimino)-2,5-dihydro-1*H*-1λ^6-thiophene 1-oxide (A15)

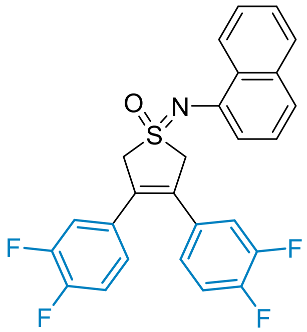

A15

Yellow oil; 560 mg, 6% total yield; ^{1}H NMR (400 MHz, $CDCl_3$) δ 8.37-8.29 (m, 1H), 7.85-7.77 (m, 1H), 7.54 (d, *J* = 8.2 Hz, 1H), 7.51-7.42 (m, 2H), 7.37 (t, *J* = 7.4 Hz,1H), 7.29-7.27 (m, 1H), 7.11-7.02 (m, 2H), 6.93-6.85 (m, 2H), 6.85-6.76 (m, 2H), 4.66-4.57 (m, 2H), 4.50-4.41 (m, 2H); ^{13}C NMR (100 MHz, $CDCl_3$) δ 150.52 (d, *J* = 252.5 Hz), 150.37 (d, *J* = 253.1 Hz), 141.24, 134.84, 131.18 (dd, *J* = 5.8, 4.4 Hz), 130.90, 130.13, 128.05, 126.40, 126.18, 125.48, 125.01 (dd, *J* = 6.3, 3.7 Hz), 123.82, 122.83, 118.15

(d, J = 18.1 Hz), 117.73 (d, J = 17.9 Hz), 116.52, 62.09; ^{19}F NMR (376 MHz, $CDCl_3$) δ -135.45 (d, J = 21.3 Hz), -135.66 (d, J = 21.4 Hz); HRMS (APCI) m/z calcd for $C_{26}H_{17}F_4NOS$ $[M+H]^+$ = 468.1045, found = 468.1040.

3,4-bis(2,4-dichlorophenyl)-1-(naphthalen-1-ylimino)-2,5-dihydro-1*H*-1λ^6-thiophene 1-oxide (A16)

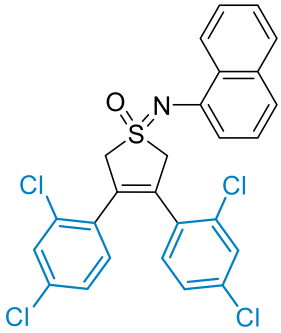

A16

Light yellow solid; 1.4 g, 13% total yield; m.p. = 120.0-121.9 °C; ^{1}H NMR (400 MHz, $CDCl_3$) δ 8.45-8.39 (m, 1H), 7.84-7.77 (m, 1H), 7.53 (d, J = 8.1 Hz, 1H), 7.50-7.45 (m, 2H), 7.39-7.29 (m, 4H), 7.09 (dd, J = 8.3, 2.0 Hz, 2H), 6.99 (d, J = 8.3 Hz, 2H), 4.60 (d, J = 15.4 Hz, 2H), 4.46 (d, J = 15.4 Hz, 2H); ^{13}C NMR (100 MHz, $CDCl_3$) δ 141.40, 135.61, 134.80, 133.87, 133.80, 131.95, 131.14, 130.26, 129.88, 127.94, 127.81, 126.30, 126.16, 125.34, 124.13, 122.73, 116.68, 60.01; HRMS (ESI) m/z calcd for $C_{26}H_{17}Cl_4NOS$ $[M+H]^+$ = 531.9863, found = 531.9864.

1-((4-(benzyloxy)naphthalen-1-yl)imino)-3,4-diphenyl-2,5-dihydro-1*H*-1λ^6-thiophene 1-oxide (A17)

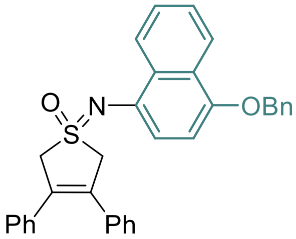

A17

Yellow solid; 802 mg, 8% total yield; m.p. = 112.9-113.9 °C; ^{1}H NMR (400 MHz, $CDCl_3$) δ 8.36 (s, 2H), 7.63-7.34 (m, 7H), 7.33-7.20 (m, 7H), 7.13 (s, 4H), 6.84 (d, J = 7.1 Hz, 1H), 5.26 (s, 2H), 4.66 (d, J = 15.8 Hz, 2H), 4.50 (d, J = 15.8 Hz, 2H); ^{13}C

NMR (100 MHz, $CDCl_3$) δ 150.63, 137.55, 135.08, 134.64, 131.66, 131.20, 128.72, 128.69, 128.62, 128.45, 127.98, 127.54, 126.84, 126.12, 125.70, 123.87, 122.30, 116.96, 105.60, 70.44, 62.02; HRMS (ESI) *m/z* calcd for $C_{33}H_{27}NO_2S$ $[M+H]^+$ = 502.1840, found = 502.1835.

1-((4-ethoxynaphthalen-1-yl)imino)-3,4-diphenyl-2,5-dihydro-1*H*-1λ^6-thiophene 1-oxide (A18)

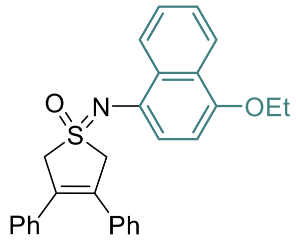

A18

Yellow solid; 1.6 g, 18% total yield; m.p. = 118.5-119.2°C; ^{1}H NMR (400 MHz, $CDCl_3$) δ 8.41-8.25 (m, 2H), 7.54-7.43 (m, 2H), 7.26-7.17 (m, 7H), 7.15-7.05 (m, 4H), 6.73 (d, *J* = 8.1 Hz, 1H), 4.63 (d, *J* = 15.3 Hz, 2H), 4.47 (d, *J* = 15.3 Hz, 2H), 4.19 (q, *J* = 6.9 Hz, 2H), 1.54 (t, *J* = 6.9 Hz, 3H); ^{13}C NMR (100 MHz, $CDCl_3$) δ 150.88, 135.10, 134.16, 131.68, 131.15, 128.70, 128.60, 128.41, 126.79, 126.01, 125.52, 123.79, 122.19, 117.18, 105.04, 64.01, 61.95, 15.08; HRMS (ESI) *m/z* calcd for $C_{28}H_{25}NO_2S$ $[M+H]^+$ = 440.1684, found = 440.1676.

1-((4-methoxynaphthalen-1-yl)imino)-3,4-diphenyl-2,5-dihydro-1*H*-1λ^6-thiophene 1-oxide (A19)

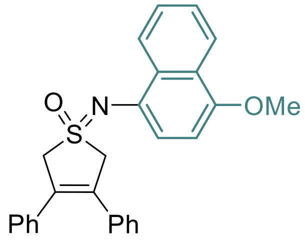

A19

White solid; 2.2 g, 26% total yield; m.p. = 135.5-137.6 °C; ^{1}H NMR (400 MHz, $CDCl_3$) δ 8.37-8.29 (m, 1H), 8.28-8.21 (m, 1H), 7.53-7.44 (m, 2H), 7.26-7.19 (m, 7H), 7.13-7.06 (m, 4H), 6.74 (d, *J* = 8.1 Hz, 1H), 4.67-4.59 (m, 2H), 4.51-4.43 (m, 2H), 3.98 (s,

3H); ^{13}C NMR (100 MHz, $CDCl_3$) δ 151.55, 135.09, 134.33, 131.68, 131.13, 128.71, 128.61, 128.43, 126.61, 126.08, 125.63, 123.85, 122.06, 117.08, 104.04, 61.98, 55.76; HRMS (ESI) *m/z* calcd for $C_{27}H_{23}NO_2S$ $[M+H]^+$ = 426.1527, found = 426.1517.

<u>1-((4-methylnaphthalen-1-yl)imino)-3,4-diphenyl-2,5-dihydro-1*H*-1λ^6-thiophene 1-oxide (A20)</u>

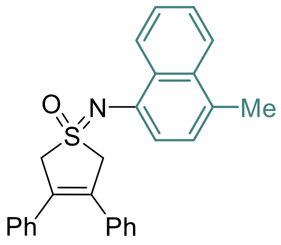

A20

Yellow solid; 1.7 g, 21% total yield; m.p. = 120.3-121.7 °C; ^{1}H NMR (400 MHz, $CDCl_3$) δ 8.43 (dd, *J* = 8.3, 0.8 Hz, 1H), 7.95 (d, *J* = 7.9 Hz, 1H), 7.7.56-7.50 (m, 1H), 7.50-7.43 (m, 1H), 7.26-7.17 (m, 8H), 7.15-7.06 (m, 4H), 4.73-4.62 (m, 2H), 4.55-4.46 (m, 2H), 2.64 (s, 3H); ^{13}C NMR (100 MHz, $CDCl_3$) δ 139.97, 135.04, 133.74, 131.57, 130.37, 128.73, 128.62, 128.47, 126.73, 126.09, 125.07, 124.56, 124.30, 116.43, 62.28, 19.21; HRMS (ESI) *m/z* calcd for $C_{27}H_{23}NOS$ $[M+H]^+$ = 410.1578, found = 410.1574.

<u>1-((4-bromonaphthalen-1-yl)imino)-3,4-diphenyl-2,5-dihydro-1*H*-1λ^6-thiophene 1-oxide (A2)</u>

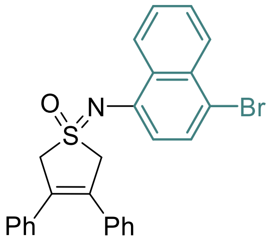

A21

Yellow solid; 1.2 g, 13% total yield; m.p. = 126.2-128.0 °C; ^{1}H NMR (400 MHz, $CDCl_3$) δ 8.42 (d, *J* = 8.1 Hz, 1H), 8.19 (d, *J* = 8.1 Hz, 1H), 7.65 (d, *J* = 8.0 Hz, 1H), 7.62-7.56 (m, 1H), 7.54-7.47 (m, 1H), 7.26-7.20 (m, 6H), 7.18 (d, *J* = 8.0 Hz, 1H), 7.14-7.06 (m, 4H), 4.72-4.63 (m, 2H), 4.59-4.50 (m, 2H); ^{13}C NMR (100 MHz, $CDCl_3$) δ 141.98, 134.81, 132.93, 131.48, 131.42, 129.96, 128.78, 128.59, 127.68, 127.22, 126.07,

124.61, 116.55, 115.80, 62.46; HRMS (ESI) *m/z* calcd for $C_{26}H_{20}BrNOS$ $[M+H]^+$ = 474.0527, found = 474.0526.

1-((4-fluoronaphthalen-1-yl)imino)-3,4-diphenyl-2,5-dihydro-1*H*-1λ^6-thiophene 1-oxide (A22)

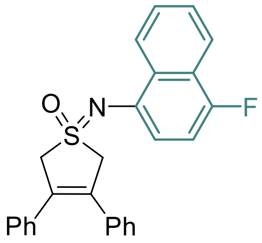

A22

Yellow solid; 2.4 g, 29% total yield; m.p. = 96.4-97.6 °C; ^{1}H NMR (400 MHz, $CDCl_3$) δ 8.36 (d, *J* = 8.0 Hz, 1H), 8.06 (d, *J* = 7.7 Hz, 1H), 7.57-7.48 (m, 2H), 7.26-7.18 (m, 7H), 7.16-7.08 (m, 4H), 7.08-7.01 (m, 1H), 4.70-4.60 (m, 2H), 4.56-4.45 (m, 2H); ^{13}C NMR (100 MHz, $CDCl_3$) δ 154.88 (d, *J* = 246.6 Hz), 137.64 (d, *J* = 3.9 Hz), 134.93, 131.55, 131.16 (d, *J* = 4.7 Hz), 128.77, 128.60, 128.55, 126.55 (d, *J* = 1.6 Hz), 126.31, 124.71 (d, *J* = 17.3 Hz), 124.22 (d, *J* = 2.5 Hz), 120.62 (d, *J* = 4.8 Hz), 115.92 (d, *J* = 7.7 Hz), 109.38 (d, *J* = 20.6 Hz), 62.24; ^{19}F NMR (376 MHz, $CDCl_3$) δ -130.15; HRMS (ESI) *m/z* calcd for $C_{26}H_{20}FNOS$ $[M+H]^+$ = 414.1328, found = 414.1324.

1-(naphthalen-2-ylimino)-3,4-diphenyl-2,5-dihydro-1*H*-1λ^6-thiophene 1-oxide (A23)

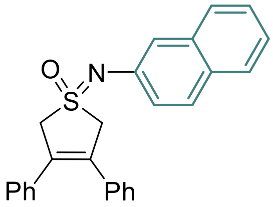

A23

Yellow solid; 1.9 g, 24% total yield; m.p. = 128.6-130.3 °C; ^{1}H NMR (400 MHz, DMSO-*d6*) δ 7.79 (d, *J* = 8.6 Hz, 2H), 7.76 (d, *J* = 8.3 Hz, 1H), 7.45 (d, *J* = 1.9 Hz, 1H), 7.44-7.39 (m, 1H), 7.33-7.29 (m, 1H), 7.28-7.24 (m, 6H), 7.24-7.16 (m, 5H), 4.85 (d, *J* = 15.8 Hz, 2H), 4.79 (d, *J* = 15.8 Hz, 2H); ^{13}C NMR (100 MHz, DMSO-*d6*) δ 143.66, 134.95, 134.22, 130.83, 128.80, 128.59, 128.43, 128.12, 127.37, 126.50,

126.08, 124.41, 123.55, 115.90, 61.89; HRMS (APCI) *m/z* calcd for $C_{26}H_{21}NOS$ $[M+H]^+$ = 396.1422, found = 396.1417.

1-(benzo*[b]*thiophen-3-ylimino)-3,4-diphenyl-2,5-dihydro-1*H*-1λ^6-thiophene 1-oxide (A24)

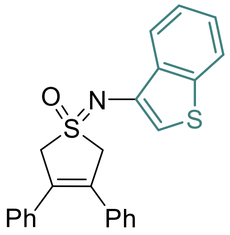

A24

White solid; 1.1 g, 14% total yield; m.p. = 113.7-114.9 °C; ^{1}H NMR (400 MHz, $CDCl_3$) δ 7.95-7.87 (m, 1H), 7.83-7.77 (m, 1H), 7.39-7.33 (m, 2H), 7.26-7.20 (m, 6H), 7.14-7.08 (m, 4H), 6.88 (s, 1H), 4.72-4.64 (m, 2H), 4.55-4.47 (m, 2H); ^{13}C NMR (100 MHz, $CDCl_3$) δ 138.56, 137.72, 137.00, 134.86, 131.36, 128.76, 128.61, 128.56, 124.89, 123.89, 122.85, 121.97, 107.93, 62.05; HRMS (ESI) *m/z* calcd for $C_{24}H_{19}NOS_2$ $[M+H]^+$ = 402.0986, found = 402.0985.

3,4-diphenyl-1-(quinolin-5-ylimino)-2,5-dihydro-1*H*-1λ^6-thiophene 1-oxide (A25)

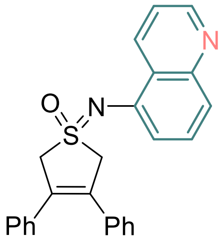

A25

Yellow solid; 1,8 g, 23% total yield; m.p. = 129.2-130.5 °C; ^{1}H NMR (400 MHz, $CDCl_3$) δ 8.96-8.23 (m, 1H), 8.73 (d, *J* = 8.4 Hz, 1H), 7.78 (d, *J* = 8.5 Hz, 1H), 7.64-7.55 (m, 1H), 7.36 (dd, *J* = 8.5, 4.2 Hz, 1H), 7.32 (d, *J* = 7.4 Hz, 1H), 7.26-7.19 (m, 6H), 7.14-7.06 (m, 4H), 4.74-4.65 (m, 2H), 4.61-4.53 (m, 2H); ^{13}C NMR (100 MHz, $CDCl_3$) δ 150.57, 149.48, 142.11, 134.76, 132.90, 131.37, 129.76, 128.80, 128.64, 128.58, 125.45, 123.42, 120.37, 115.96, 62.51; HRMS (ESI) *m/z* calcd for $C_{25}H_{20}N_2OS$ $[M+H]^+$ = 397.1374, found = 397.1374.

1-(isoquinolin-5-ylimino)-3,4-diphenyl-2,5-dihydro-1*H*-1λ^6-thiophene 1-oxide (A26)

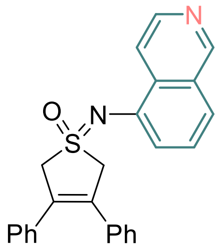

A26

Yellow solid; 1.1 g, 14% total yield; m.p. = 105.8-106.7 °C; ^{1}H NMR (400 MHz, $CDCl_3$) δ 9.19 (s, 1H), 8.50 (d, *J* = 5.8 Hz, 1H), 8.14 (d, *J* = 5.8 Hz, 1H), 7.61 (dd, *J* = 6.4, 2.5 Hz, 1H), 7.52-7.45 (m, 2H), 7.26-7.19 (m, 6H), 7.15-7.07 (m, 4H), 4.69 (d, *J* = 15.4 Hz, 2H), 4.57 (d, *J* = 15.4 Hz, 2H); ^{13}C NMR (100 MHz, $CDCl_3$) δ 152.23, 142.62, 141.24, 134.70, 133.01, 131.34, 129.92, 128.78, 128.62, 128.56, 127.62, 121.32, 119.56, 116.95, 62.56; HRMS (ESI) *m/z* calcd for $C_{25}H_{20}N_2OS$ $[M+H]^+$ = 397.1374, found = 397.1371.

1-(phenanthren-9-ylimino)-3,4-diphenyl-2,5-dihydro-1*H*-1λ^6-thiophene 1-oxide (A27)

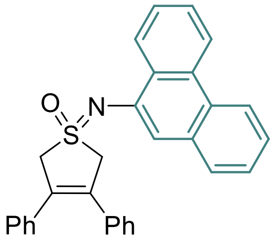

A27

White solid; 1.7 g, 19% total yield; m.p. = 152.1-153.4°C; ^{1}H NMR (400 MHz, $CDCl_3$) δ 8.69 (d, *J* = 8.0 Hz, 1H), 8.66-8.59 (m, 1H), 8.55 (dd, *J* = 8.0, 1.1 Hz, 1H), 7.85-7.78 (m, 1H), 7.71-7.65 (m, 1H), 7.64-7.59 (m, 1H), 7.58-7.49 (m, 3H), 7.26-7.19 (m, 6H), 7.15-7.07 (m, 4H), 4.80-4.72 (m, 2H), 4.65-4.56 (m, 2H); ^{13}C NMR (100 MHz, $CDCl_3$) δ 140.25, 134.90, 132.94, 131.49, 131.46, 130.44, 128.73, 128.60, 128.50, 127.63, 127.60, 126.99, 126.89, 126.42, 124.83, 124.66, 122.72, 122.52, 114.55, 62.43; HRMS (ESI) *m/z* calcd for $C_{30}H_{23}NOS$ $[M+H]^+$ = 446.1578, found = 446.1576.

3,4-diphenyl-1-(pyren-4-ylimino)-2,5-dihydro-1*H*-1λ⁶-thiophene 1-oxide (A28)

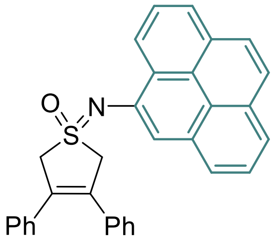

A28

Yellow solid; 1.5 g, 16% total yield; m.p. = 116.8-117.9 °C; ^{1}H NMR (400 MHz, $CDCl_3$) δ 8.60 (d, *J* = 9.2 Hz, 1H), 8.10 (dd, *J* = 7.8, 5.9 Hz, 3H), 8.03 (d, *J* = 9.2 Hz, 1H), 8.00 (d, *J* = 9.0 Hz, 1H), 7.96 (t, *J* = 6.6 Hz, 2H), 7.93 (d, *J* = 6.6 Hz, 1H), 7.26-7.20 (m, 6H), 7.17-7.11 (m, 4H), 4.80-4.71 (m, 2H), 4.64-4.55 (m, 2H); ^{13}C NMR (100 MHz, $CDCl_3$) δ 139.75, 134.93, 131.83, 131.79, 131.59, 128.76, 128.62, 128.52, 127.51, 127.00, 126.62, 126.11, 126.09, 125.78, 125.60, 125.59, 125.14, 124.46, 124.32, 123.38, 119.80, 62.54; HRMS (ESI) *m/z* calcd for $C_{32}H_{23}NOS$ $[M+H]^+$ = 470.1578, found = 470.1573.

3,4-diphenyl-1-(phenylimino)-2,5-dihydro-1*H*-1λ⁶-thiophene 1-oxide (A29)

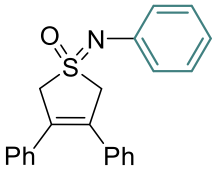

A29

Yellow solid; 2.1 g, 30% total yield; m.p. = 107.2-108.7 °C; ^{1}H NMR (400 MHz, $CDCl_3$) δ 7.31-7.22 (m, 8H), 7.17-7.08 (m, 6H), 7.01 (t, *J* = 7.3 Hz, 1H), 4.64 (d, *J* = 15.4 Hz, 2H), 4.48 (d, *J* = 15.4 Hz, 2H); ^{13}C NMR (100 MHz, $CDCl_3$) δ 145.33, 134.90, 131.17, 129.50, 128.71, 128.62, 128.48, 122.90, 122.32, 62.64; HRMS (ESI) *m/z* calcd for $C_{22}H_{19}NOS$ $[M+H]^+$ = 346.1265, found = 346.1261.

1-((2-methoxyphenyl)imino)-3,4-diphenyl-2,5-dihydro-1*H*-1λ⁶-thiophene 1-oxide (A30)

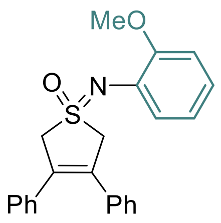

A30

Yellow solid; 1.4 g, 19% total yield; m.p. = 86.1-87.0 °C; ^{1}H NMR (400 MHz, $CDCl_3$) δ 7.26-7.18 (m, 6H), 7.18-7.14 (m, 1H), 7.14-7.06 (m, 4H), 7.02-6.96 (m, 1H), 6.94-6.87 (m, 2H), 4.78-4.68 (m, 2H), 4.52-4.43 (m, 2H), 3.88 (s, 3H); ^{13}C NMR (100 MHz, $CDCl_3$) δ 152.27, 135.20, 133.79, 131.14, 128.67, 128.64, 128.32, 124.69, 123.09, 121.58, 111.94, 64.56, 56.03; HRMS (ESI) *m/z* calcd for $C_{23}H_{21}NO_2S$ $[M+H]^+$ = 376.1371, found = 376.1367.

1-((2-ethylphenyl)imino)-3,4-diphenyl-2,5-dihydro-1*H*-1λ^6-thiophene 1-oxide (A31)

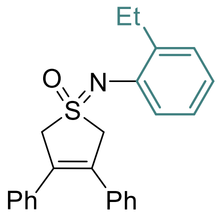

A31

Yellow oil; 1.8 g, 24% total yield; ^{1}H NMR (400 MHz, $CDCl_3$) δ 7.26-7.16 (m, 8H), 7.14-7.06 (m, 5H), 6.97 (td, *J* = 7.4, 1.1 Hz, 1H), 4.64-4.54 (m, 2H), 4.51-4.43 (m, 2H), 2.71 (q, *J* = 7.5 Hz, 2H), 1.20 (t, *J* = 7.5 Hz, 3H); ^{13}C NMR (100 MHz, $CDCl_3$) δ 143.19, 138.68, 135.09, 131.63, 129.20, 128.72, 128.57, 128.42, 126.62, 122.58, 121.47, 62.30, 25.16, 14.68; HRMS (ESI) *m/z* calcd for $C_{24}H_{23}NOS$ $[M+H]^+$ = 374.1578, found = 374.1571.

3,4-diphenyl-1-(o-tolylimino)-2,5-dihydro-1*H*-1λ^6-thiophene 1-oxide (A32)

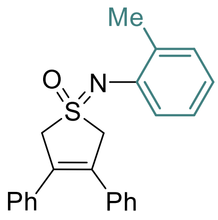

A32

Yellow oil; 718 mg, 10% total yield; ^{1}H NMR (400 MHz, $CDCl_3$) δ 7.26-7.22 (m, 6H), 7.21-7.16 (m, 2H), 7.14-7.08 (m, 5H), 6.94 (td, J = 7.4, 0.9 Hz, 1H), 4.62-4.55 (m, 2H), 4.50-4.42 (m, 2H), 2.29 (s, 3H); ^{13}C NMR (100 MHz, $CDCl_3$) δ 143.58, 135.09, 132.82, 131.53, 130.79, 128.73, 128.59, 128.44, 126.64, 122.51, 121.62, 62.44, 18.49; HRMS (ESI) *m/z* calcd for $C_{23}H_{21}NOS$ $[M+H]^+$ = 360.1422, found = 360.1425.

1-((2-bromophenyl)imino)-3,4-diphenyl-2,5-dihydro-1*H*-1λ^6-thiophene 1-oxide (A33)

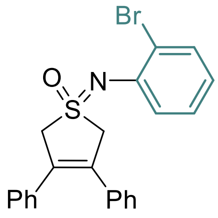

A33

Yellow oil; 1.4 g, 17% total yield; ^{1}H NMR (400 MHz, $CDCl_3$) δ 7.57 (dd, J = 8.0, 1.4 Hz, 1H), 7.32 (dd, J = 7.9, 1.5 Hz, 1H), 7.26-7.18 (m, 7H), 7.15-7.10 (m, 4H), 6.89 (td, J = 7.9, 1.5 Hz, 1H), 4.69-4.58 (m, 2H), 4.51-4.39 (m, 2H); ^{13}C NMR (100 MHz, $CDCl_3$) δ 143.31, 134.94, 133.44, 131.35, 128.74, 128.65, 128.49, 128.26, 123.93, 123.91, 119.69, 62.74; HRMS (ESI) *m/z* calcd for $C_{22}H_{18}BrNOS$ $[M+H]^+$ = 424.0370, found = 424.0369.

1-((2-chlorophenyl)imino)-3,4-diphenyl-2,5-dihydro-1*H*-1λ^6-thiophene 1-oxide (A34)

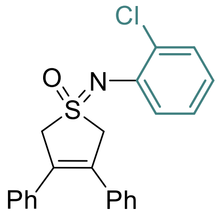

A34

Yellow oil; 2.2 g, 29% total yield; ^{1}H NMR (400 MHz, $CDCl_3$) δ 7.39 (dd, *J* = 8.0, 1.4 Hz, 1H), 7.31 (dd, *J* = 8.0, 1.4 Hz, 1H), 7.26-7.20 (m, 6H), 7.17 (td, *J* = 7.7, 1.5 Hz, 1H), 7.15-7.07 (m, 4H), 6.96 (td, *J* = 7.7, 1.4 Hz, 1H), 4.69-4.60 (m, 2H), 4.52-4.42 (m, 2H); ^{13}C NMR (100 MHz, $CDCl_3$) δ 141.93, 134.91, 131.28, 130.33, 128.99, 128.73, 128.63, 128.49, 127.54, 124.14, 123.54, 62.91; HRMS (ESI) *m/z* calcd for $C_{22}H_{18}ClNOS$ $[M+H]^+$ = 380.0876, found = 380.0872

1-((2-fluorophenyl)imino)-3,4-diphenyl-2,5-dihydro-1*H*-1λ^6-thiophene 1-oxide (A35)

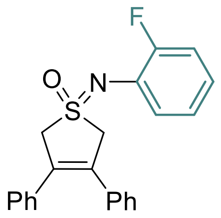

A35

Yellow solid; 2.1 g, 29% total yield; m.p. = 84.3-85.4 °C; ^{1}H NMR (400 MHz, $CDCl_3$) δ 7.26-7.20 (m, 7H), 7.16-7.10 (m, 4H), 7.10-7.01 (m, 2H), 7.00-6.92 (m, 1H), 4.73-4.65 (m, 2H), 4.50 (d, *J* = 15.2 Hz, 2H); ^{13}C NMR (100 MHz, $CDCl_3$) δ 156.06 (d, *J* = 242.7 Hz), 134.89, 132.44 (d, *J* = 13.0 Hz), 131.10, 128.73, 128.64, 128.49, 125.51 (d, *J* = 2.3 Hz), 124.70 (d, *J* = 3.6 Hz), 123.19 (d, *J* = 7.5 Hz), 116.15 (d, *J* = 20.8 Hz), 63.75 (d, *J* = 1.7 Hz); ^{19}F NMR (376 MHz, $CDCl_3$) δ -125.41; HRMS (ESI) *m/z* calcd for $C_{22}H_{18}FNOS$ $[M+H]^+$ = 364.1171, found = 364.1173.

1-((2,5-dimethoxyphenyl)imino)-3,4-diphenyl-2,5-dihydro-1*H*-1λ^6-thiophene 1-oxide (A36)

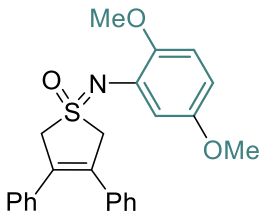

A36

Yellow solid; 2.0 g, 25% total yield; m.p. = 119.6-120.5°C; ^{1}H NMR (400 MHz, $CDCl_3$) δ 7.25-7.19 (m, 6H), 7.15-7.08 (m, 4H), 6.83 (d, *J* = 8.8 Hz, 1H), 6.75 (d, *J* = 3.0 Hz, 1H), 6.52 (dd, *J* = 8.8, 3.0 Hz, 1H), 4.77-4.68 (m, 2H), 4.53-4.44 (m, 2H), 3.84 (s, 3H),

3.76 (s, 3H); ^{13}C NMR (100 MHz, $CDCl_3$) δ 154.53, 146.45, 135.16, 134.78, 131.10, 128.67, 128.63, 128.32, 113.09, 110.89, 107.63, 64.72, 56.88, 55.80; HRMS (ESI) *m/z* calcd for $C_{24}H_{23}NO3S$ $[M+H]^+$ = 406.1477, found = 406.1477.

1-((2,5-dimethylphenyl)imino)-3,4-diphenyl-2,5-dihydro-1*H*-1λ^6-thiophene 1-oxide (A37)

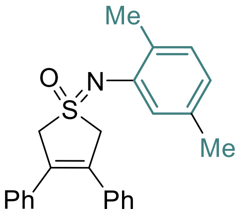

A37

Yellow solid; 1.6 g, 22% total yield; m.p. = 124.5-126.8 °C; ^{1}H NMR (400 MHz, $CDCl_3$) δ 7.26-7.20 (m, 6H), 7.15-7.08 (m, 4H), 7.06 (d, J = 7.5 Hz, 1H), 7.02 (s, 1H), 6.76 (d, J = 7.5 Hz, 1H), 4.63-4.53 (m, 2H), 4.51-4.41 (m, 2H), 2.30 (s, 3H), 2.24 (s, 3H); ^{13}C NMR (100 MHz, $CDCl_3$) δ 143.35, 136.25, 135.14, 131.58, 130.52, 129.61, 128.72, 128.59, 128.42, 123.27, 122.43, 62.41, 21.23, 18.02; HRMS (ESI) *m/z* calcd for $C_{24}H_{23}NOS$ $[M+H]^+$ = 374.1578, found = 374.1572.

1-((5-chloro-2-methylphenyl)imino)-3,4-diphenyl-2,5-dihydro-1*H*-1λ^6-thiophene 1-oxide (A38)

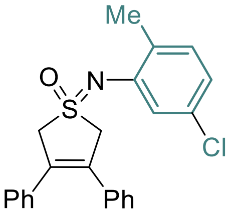

A38

Yellow solid; 1.3 g, 16% total yield; m.p. = 128.5-130.5 °C; ^{1}H NMR (400 MHz, $CDCl_3$) δ 7.27-7.23 (m, 6H), 7.20 (d, J = 2.1 Hz, 1H), 7.16-7.09 (m, 4H), 7.08 (d, J = 8.0 Hz, 1H), 6.90 (dd, J = 8.0, 2.1 Hz, 1H), 4.58 (d, J = 15.6 Hz, 2H), 4.49 (d, J = 15.6 Hz, 2H), 2.23 (s, 3H); ^{13}C NMR (100 MHz, $CDCl_3$) δ 144.80, 134.91, 131.58, 131.44, 131.25, 128.78, 128.58, 128.55, 122.24, 121.08, 62.56, 18.00; HRMS (ESI) *m/z* calcd for $C_{23}H_{20}ClNOS$ $[M+H]^+$ = 394.1032, found = 394.1029.

1-((2,4-dimethylphenyl)imino)-3,4-diphenyl-2,5-dihydro-1*H*-1λ^6-thiophene 1-oxide (A39)

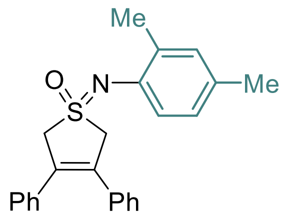

A39

Yellow solid; 671 mg, 9% total yield; m.p. = 95.0-96.9 ^{o}C; ^{1}H NMR (400 MHz, $CDCl_3$) δ 7.25-7.20 (m, 6H), 7.12-7.06 (m, 5H), 7.00 (s, 1H), 6.92 (d, J = 7.9 Hz, 1H), 4.56 (d, J = 14.8 Hz, 2H), 4.43 (d, J = 14.8 Hz, 2H), 2.27 (s, 3H), 2.26 (s, 3H); ^{13}C NMR (100 MHz, $CDCl_3$) δ 140.82, 135.17, 132.63, 131.96, 131.60, 128.72, 128.59, 128.41, 127.11, 121.80, 62.23, 20.85, 18.41; HRMS (ESI) *m/z* calcd for $C_{24}H_{23}NOS$ $[M+H]^+$ = 374.1578, found = 374.1577.

1-((2,3-dimethylphenyl)imino)-3,4-diphenyl-2,5-dihydro-1*H*-1λ^6-thiophene 1-oxide (A40)

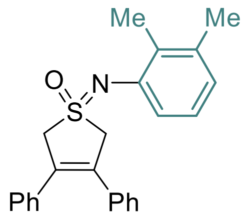

A40

Yellow oil; 1.9 g, 25% total yield; ^{1}H NMR (400 MHz, $CDCl_3$) δ 7.26-7.20 (m, 6H), 7.15-7.06 (m, 5H), 7.01 (t, J = 7.5 Hz, 1H), 6.85 (d, J = 7.5 Hz, 1H), 4.58 (d, J = 14.9 Hz, 2H), 4.45 (d, J = 14.9 Hz, 2H), 2.28 (s, 3H), 2.24 (s, 3H); ^{13}C NMR (100 MHz, $CDCl_3$) δ 143.26, 138.00, 135.15, 131.54, 131.29, 128.71, 128.60, 128.41, 125.78, 124.35, 119.62, 62.37, 20.81, 14.16; HRMS (ESI) *m/z* calcd for $C_{24}H_{23}NOS$ $[M+H]^+$ = 374.1578, found = 374.1578.

1-((2,6-dimethoxyphenyl)imino)-3,4-diphenyl-2,5-dihydro-1*H*-1λ^6-thiophene 1-oxide (A41)

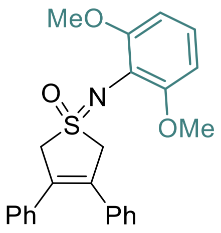

A41

Yellow solid; 1.3 g, 16% total yield; m.p. = 119.5-121.5 °C; ^{1}H NMR (400 MHz, $CDCl_3$) δ 7.25-7.18 (m, 6H), 7.14-7.08 (m, 4H), 6.93 (t, *J* = 8.3 Hz, 1H), 6.61 (d, *J* = 8.3 Hz, 2H), 4.78 (d, *J* = 15.4 Hz, 2H), 4.47 (d, *J* = 15.4 Hz, 2H), 3.88 (s, 6H); ^{13}C NMR (100 MHz, $CDCl_3$) δ 153.32, 135.43, 131.14, 128.62, 128.60, 128.15, 122.53, 122.24, 105.33, 65.94, 56.41; HRMS (ESI) *m/z* calcd for $C_{24}H_{23}NO3S$ $[M+H]^+$ = 406.1477, found = 406.1475.

<u>1-((2,6-dichlorophenyl)imino)-3,4-diphenyl-2,5-dihydro-1*H*-1λ^6-thiophene 1-oxide (A42)</u>

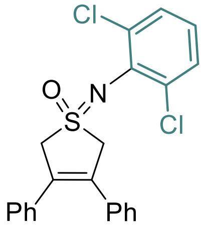

A42

Yellow solid; 1.8 g, 22% total yield; m.p. = 119.5-120.3 °C; ^{1}H NMR (400 MHz, $CDCl_3$) δ 7.32 (d, *J* = 8.0 Hz, 2H), 7.26-7.20 (m, 6H), 7.16-7.09 (m, 4H), 6.93 (t, *J* = 8.0 Hz, 1H), 4.77 (d, *J* = 15.2 Hz, 2H), 4.46 (d, *J* = 15.2 Hz, 2H); ^{13}C NMR (100 MHz, $CDCl_3$) δ 138.75, 135.01, 132.20, 131.25, 128.71, 128.64, 128.53, 128.42, 124.37, 64.20; HRMS (ESI) *m/z* calcd for $C_{22}H_{17}Cl_2NOS$ $[M+H]^+$ = 414.0486, found = 414.0482.

<u>3,4-diphenyl-1-((2,3,4-trimethoxyphenyl)imino)-2,5-dihydro-1*H*-1λ^6-thiophene 1-oxide (A43)</u>

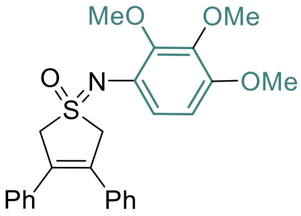

A43

Yellow solid; 2.3 g, 27% total yield; m.p. = 93.9-94.8 °C; ^{1}H NMR (400 MHz, $CDCl_3$) δ 7.25-7.19 (m, 6H), 7.15-7.08 (m, 4H), 6.84 (d, *J* = 8.8 Hz, 1H), 6.59 (d, *J* = 8.8 Hz, 1H), 4.73-4.61 (m, 2H), 4.51-4.39 (m, 2H), 3.95 (s, 3H), 3.90 (s, 3H), 3.83 (s, 3H); ^{13}C NMR (100 MHz, $CDCl_3$) δ 149.26, 146.71, 143.05, 135.22, 131.59, 131.30, 128.67, 128.62, 128.32, 119.12, 107.78, 63.94, 61.13, 60.93, 56.39; HRMS (ESI) *m/z* calcd for $C_{25}H_{25}NO_4S$ $[M+H]^+$ = 436.1582, found = 436.1591.

3-([1,1'-biphenyl]-4-yl)-4-(4-bromophenyl)-1-(naphthalen-1-ylimino)-2,5-dihydro-1*H*-1λ^6-thiophene 1-oxide (A44)

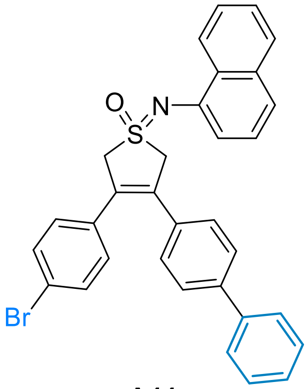

A44

Yellow solid; 769 mg, 7% total yield; m.p. = 76.4-78.1 °C; ^{1}H NMR (400 MHz, $CDCl_3$) δ 8.45-8.37 (m, 1H), 7.84-7.80 (m, 1H), 7.59-7.54 (m, 3H), 7.50-7.42 (m, 6H), 7.41-7.35 (m, 4H), 7.32 (dd, *J* = 7.3, 0.8 Hz, 1H), 7.16 (d, *J* = 8.3 Hz, 2H), 7.05-6.99 (m, 2H), 4.75-4.65 (m, 2H), 4.53 (t, *J* = 16.1 Hz, 2H); ^{13}C NMR (100 MHz, $CDCl_3$) δ 141.59, 141.44, 140.00, 134.83, 133.93, 133.41, 132.04, 131.97, 130.36, 130.26, 130.24, 129.00, 127.96, 127.91, 127.43, 127.06, 126.31, 126.20, 125.40, 123.99, 122.70, 122.58, 116.46, 62.38, 62.14; HRMS (ESI) *m/z* calcd for $C_{32}H_{24}BrNOS$ $[M+H]^+$ = 550.0840, found = 550.0836.

3-(4-bromophenyl)-4-(4'-methoxy-[1,1'-biphenyl]-4-yl)-1-(naphthalen-1-ylimino)-2,5-dihydro-1*H*-1λ^6-thiophene 1-oxide (A45)

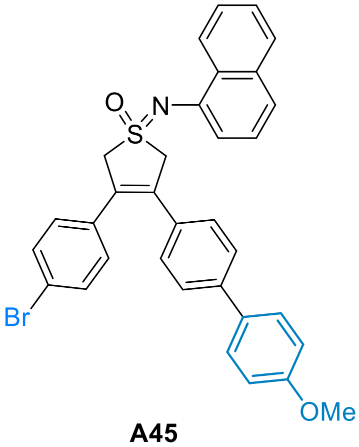

A45

Yellow solid; 1.2 g, 10% total yield; m.p. = 64.3-65.9 °C; ^{1}H NMR (400 MHz, $CDCl_3$) δ 8.45-8.34 (m, 1H), 7.85-7.78 (m, 1H), 7.54 (d, *J* = 8.2 Hz, 1H), 7.52-7.42 (m, 6H), 7.41-7.34 (m, 3H), 7.31 (dd, *J* = 7.3, 0.9 Hz, 1H), 7.13 (d, *J* = 8.4 Hz, 2H), 7.06-6.99 (m, 2H), 6.99-6.92 (m, 2H), 4.75-4.61 (m, 2H), 4.53 (t, *J* = 15.5 Hz, 2H), 3.85 (s, 3H); ^{13}C NMR (100 MHz, $CDCl_3$) δ 159.66, 141.63, 141.05, 134.83, 134.02, 132.72, 132.47, 132.04, 130.27, 130.23, 130.10, 128.97, 128.12, 127.95, 126.91, 126.31, 126.20, 125.38, 124.00, 122.65, 122.54, 116.42, 114.45, 62.41, 62.15, 55.48; HRMS (ESI) *m/z* calcd for $C_{33}H_{26}BrNO_2S$ $[M+H]^+$ = 580.0946, found = 580.0943.

4-((1-oxido-3,4-diphenyl-2,5-dihydro-1λ^6-thiophen-1-ylidene)amino)naphthalen-1-yl 5-(2,5-dimethylphenoxy)-2,2-dimethylpentanoate (A46)

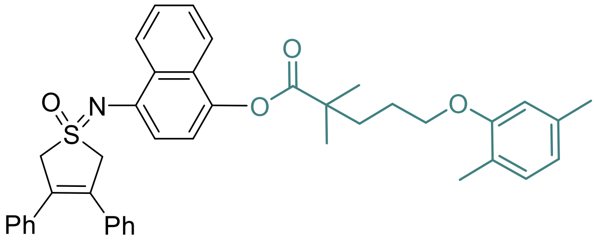

A46

Yellow oil; 3.3 g, 26% total yield; ^{1}H NMR (400 MHz, $CDCl_3$) δ 8.49-8.36 (m, 1H), 7.92-7.79 (m, 1H), 7.54-7.46 (m, 2H), 7.29-7.23 (m, 7H), 7.17-7.08 (m, 5H), 7.03 (d, *J* = 7.4 Hz, 1H), 6.73-6.64 (m, 2H), 4.76-4.65 (m, 2H), 4.60-4.48 (m, 2H), 4.05 (t, *J* = 5.5 Hz, 2H), 2.33 (s, 3H), 2.21 (s, 3H), 2.06-1.96 (m, 4H), 1.53 (s, 6H); ^{13}C NMR (100 MHz, $CDCl_3$) δ 176.74, 157.06, 142.12, 139.75, 136.61, 134.91, 131.48, 131.01, 130.46, 128.74, 128.61, 128.51, 128.04, 126.78, 125.78, 124.54, 123.78, 121.05, 120.88,

118.05, 115.59, 112.13, 68.03, 62.35, 42.92, 37.39, 25.52, 25.49, 21.54, 15.97; HRMS (ESI) *m/z* calcd for $C_{41}H_{41}NO_4S$ $[M+H]^+$ = 644.2834, found = 644.2838.

4-((1-oxido-3,4-diphenyl-2,5-dihydro-1λ^6-thiophen-1-ylidene)amino)naphthalen-1-yl stearate (A47)

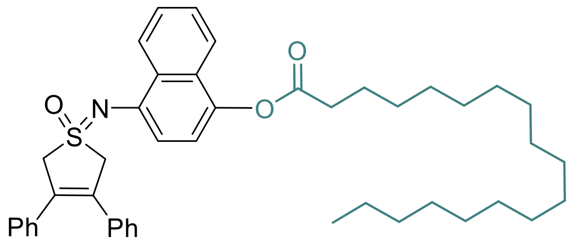

A47

Yellow solid; 2.2 g, 16% total yield; m.p. = 105.1-106.8 °C; ^{1}H NMR (400 MHz, $CDCl_3$) δ 8.41 (d, *J* = 7.9 Hz, 1H), 7.81 (d, *J* = 7.9 Hz, 1H), 7.55-7.45 (m, 2H), 7.27-7.21 (m, 7H), 7.16-7.07 (m, 5H), 4.69 (d, *J* = 15.1 Hz, 2H), 4.53 (d, *J* = 15.1 Hz, 2H), 2.73 (t, *J* = 7.5 Hz, 2H), 1.92-1.81 (m, 2H), 1.41-1.23 (m, 28H), 0.89 (t, *J* = 6.7 Hz, 3H); ^{13}C NMR (100 MHz, $CDCl_3$) δ 172.79, 141.92, 139.87, 134.92, 131.47, 131.00, 128.74, 128.62, 128.51, 127.93, 126.74, 125.79, 124.55, 121.18, 118.23, 115.48, 62.44, 34.57, 32.06, 29.84, 29.80, 29.76, 29.65, 29.50, 29.45, 29.40, 25.29, 22.83, 14.26; HRMS (ESI) *m/z* calcd for $C_{44}H_{55}NO_3S$ $[M+H]^+$ = 678.3981, found = 678.3990.

4-((1-oxido-3,4-diphenyl-2,5-dihydro-1λ^6-thiophen-1-ylidene)amino)naphthalen-1-yl (7Z,10Z)-octadeca-7,10-dienoate (A48)

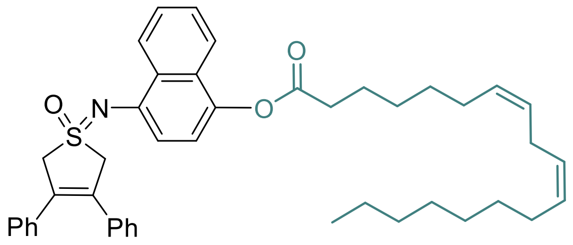

A48

Yellow solid; 2.2 g, 16% total yield; m.p. = 85.3-86.9 °C; ^{1}H NMR (400 MHz, $CDCl_3$) δ 8.40 (d, *J* = 8.1 Hz, 1H), 7.80 (d, *J* = 8.1 Hz, 1H), 7.54-7.45 (m, 2H), 7.28-7.20 (m, 7H), 7.15-7.09 (m, 5H), 5.56-5.26 (m, 4H), 4.69 (d, *J* = 15.3 Hz, 2H), 4.53 (d, *J* = 15.3 Hz, 2H), 2.76 (dt, *J* = 15.0, 7.2 Hz, 4H), 2.15-2.01 (m, 4H), 1.87 (dt, *J* = 15.0, 7.2 Hz,

2H), 1.42-1.27 (m, 14H), 0.93-0.88 (m, 3H); ^{13}C NMR (100 MHz, $CDCl_3$) δ 172.75, 141.91, 139.87, 134.91, 131.46, 130.99, 130.36, 130.16, 128.74, 128.61, 128.51, 128.22, 128.04, 127.92, 126.74, 125.79, 124.55, 121.17, 118.22, 115.47, 62.44, 34.55, 31.65, 29.75, 29.47, 29.35, 29.27, 27.34, 25.78, 25.26, 22.70, 14.21; HRMS (ESI) *m/z* calcd for $C_{44}H_{51}NO_3S$ $[M+H]^+$ = 674.3668, found = 674.3662.

<u>4-((1-oxido-3,4-diphenyl-2,5-dihydro-1λ^6-thiophen-1-ylidene)amino)naphthalen-1-yl (3*r*,5*r*,7*r*)-adamantane-1-carboxylate (A49)</u>

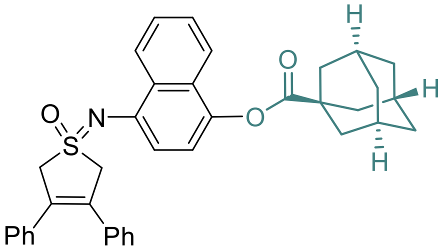

A49

Yellow solid; 3.0 g, 26% total yield; m.p. = 99.7-101.2°C; ^{1}H NMR (400 MHz, $CDCl_3$) δ 8.41-8.33 (m, 1H), 7.77 (d, *J* = 7.7 Hz, 1H), 7.53-7.41 (m, 2H), 7.25-7.17 (m, 7H), 7.13-7.02 (m, 5H), 4.72-4.61 (m, 2H), 4.55-4.46 (m, 2H), 2.24-2.15 (m, 6H), 2.13 (s, 3H), 1.80 (s, 6H); ^{13}C NMR (100 MHz, $CDCl_3$) δ 176.57, 142.12, 139.65, 134.92, 131.48, 131.01, 128.75, 128.62, 128.51, 128.08, 126.71, 125.75, 124.50, 121.15, 118.14, 115.64, 62.36, 41.52, 39.17, 36.65, 28.13; HRMS (ESI) *m/z* calcd for $C_{37}H_{35}NO_3S$ $[M+H]^+$ = 574.2416, found = 574.2423.

6. General Procedure for Enantioselective Skeletal Reorganization

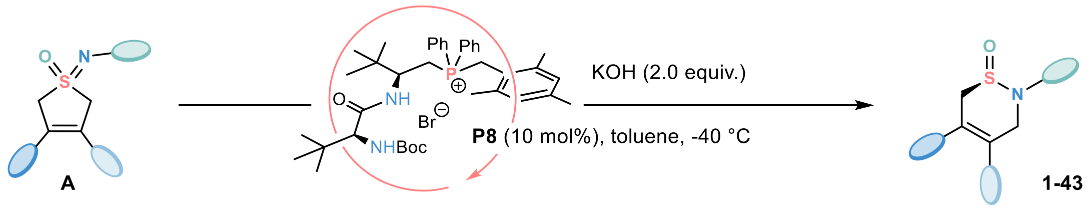

To a tube (20 mL) with a magnetic stirring bar, the sulfoximines **A** (0.10 mmol), catalyst **P8** (10 mol%) and KOH (0.20 mmol) were added, followed by the addition of

toluene (1.0 mL). The reaction mixture was stirred at -40 °C. After completion, the reaction mixture was transferred to room temperature, and diluted with dichloromethane. Purification by column chromatography on silica gel (petroleum ether/ethyl acetate = 10:1 to 5:1) afforded the prodcut **1-43**.

(*R*)-2-(naphthalen-1-yl)-4,5-diphenyl-3,6-dihydro-2*H*-1,2-thiazine 1-oxide (1)

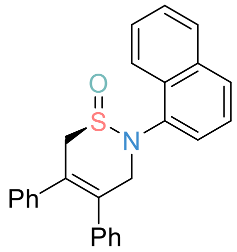

1

White solid; 40 mg, 91% yield; m.p. = 69.2-70.7 °C; ^{1}H NMR (400 MHz, $CDCl_3$) δ 7.87 (d, *J* = 8.9 Hz, 1H), 7.83 (d, *J* = 7.9 Hz, 1H), 7.79 (d, *J* = 7.9 Hz, 1H), 7.63 (d, *J* = 2.0 Hz, 1H), 7.54-7.42 (m, 3H), 7.26-7.07 (m, 10H), 4.74 (ddd, *J* = 16.8, 4.2, 2.4 Hz, 1H), 4.26 (dd, *J* = 16.8, 2.4 Hz, 1H), 4.14 (ddd, *J* = 16.6, 4.2, 2.4 Hz, 1H), 3.82 (dd, *J* = 16.6, 2.4 Hz, 1H); ^{13}C NMR (100 MHz, $CDCl_3$) δ 143.34, 140.55, 139.29, 133.89, 133.58, 131.10, 129.51, 129.38, 129.22, 128.31, 128.27, 127.73, 127.66, 127.45, 127.12, 126.85, 125.60, 124.13, 121.83, 118.05, 55.39, 46.86; HRMS (APCI) *m/z* calcd for $C_{26}H_{21}NOS$ $[M+H]^+$ = 396.1422, found = 396.1417.

Optical Rotation: $[\alpha]^{25}_D$ = 146.38 (*c* = 0.58, $CHCl_3$). 95% e.e. (HPLC condition: Chiralpak ODH column, *n*-Hexane/*i*-PrOH = 70:30, flow rate = 1.0 mL/min,

wavelength = 254 nm, t_R = 7.15 min for minor isomer, t_R = 38.23 min for major isomer).

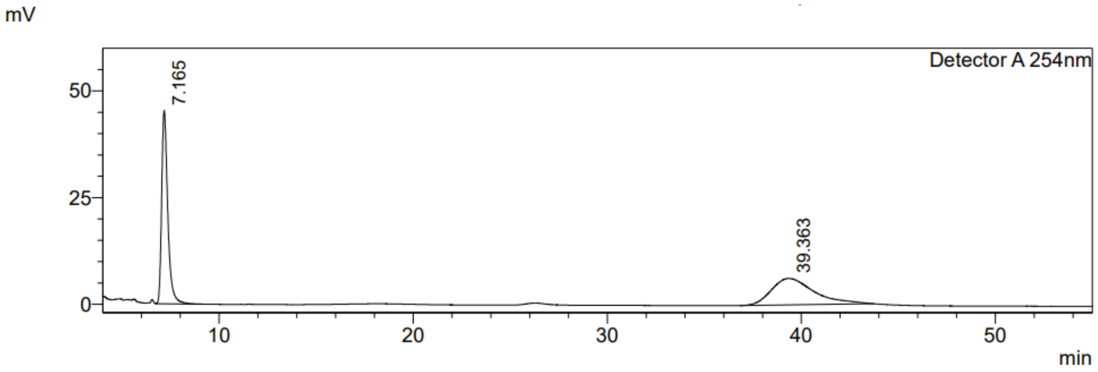

Detector A 254nm

Peak#	Ret. Time	Height	Height%	Area	Area%
1	7.165	45275	87.951	989721	50.939
2	39.363	6202	12.049	953241	49.061
Total		51477	100.000	1942962	100.000

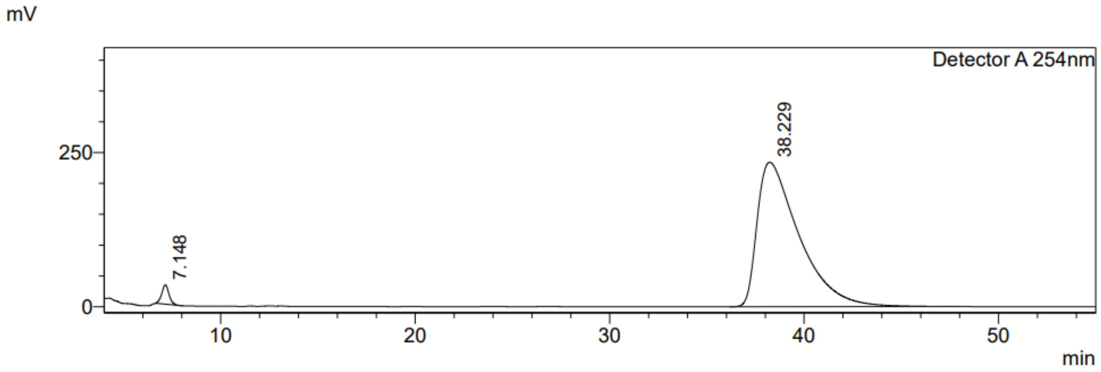

Detector A 254nm

Peak#	Ret. Time	Height	Height%	Area	Area%
1	7.148	31490	11.845	814500	2.266
2	38.229	234371	88.155	35128222	97.734
Total		265862	100.000	35942722	100.000

(*R*)-4,5-bis(4-methoxyphenyl)-2-(naphthalen-1-yl)-3,6-dihydro-2*H*-1,2-thiazine 1-oxide (2)

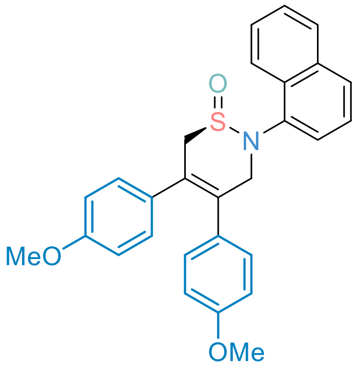

2

White solid; 37 mg, 81% yield; m.p. = 71.0-72.6 °C; ^{1}H NMR (400 MHz, $CDCl_3$) δ 8.20 (d, J = 8.1 Hz, 1H), 7.90 (d, J = 7.6 Hz, 1H), 7.81 (d, J = 8.3 Hz, 1H), 7.72 (d, J = 7.0 Hz, 1H), 7.63-7.52 (m, 2H), 7.52-7.46 (m, 1H), 7.11-7.06 (m, 2H), 7.07-7.00 (m, 2H), 6.77-6.73 (m, 2H), 6.73-6.65 (m, 2H), 4.68 (ddd, J = 17.1, 4.1, 2.4 Hz, 1H), 4.25 (ddd, J = 16.7, 4.1, 2.2 Hz, 1H), 4.09 (dd, J = 17.1, 2.2 Hz, 1H), 3.86-3.78 (m, 1H), 3.76 (s, 3H), 3.72 (s, 3H); ^{13}C NMR (100 MHz, $CDCl_3$) δ 158.61, 158.47, 142.84, 134.73, 133.14, 131.60, 130.51, 128.60, 127.79, 126.81, 126.60, 125.99, 123.08, 122.87, 113.72, 113.64, 55.92, 55.26, 55.21, 49.48; HRMS (ESI) *m/z* calcd for $C_{28}H_{25}NO_3S$ $[M+H]^+$ = 456.1633, found = 456.1630.

Optical Rotation: $[\alpha]^{25}_{D}$ = 191.43 (c = 0.49, $CHCl_3$). 96% e.e. (HPLC condition: Chiralpak IE column, n-Hexane/i-PrOH = 70:30, flow rate = 1.0 mL/min, wavelength = 254 nm, t_R = 29.83 min for major isomer, t_R = 33.42 min for minor isomer).

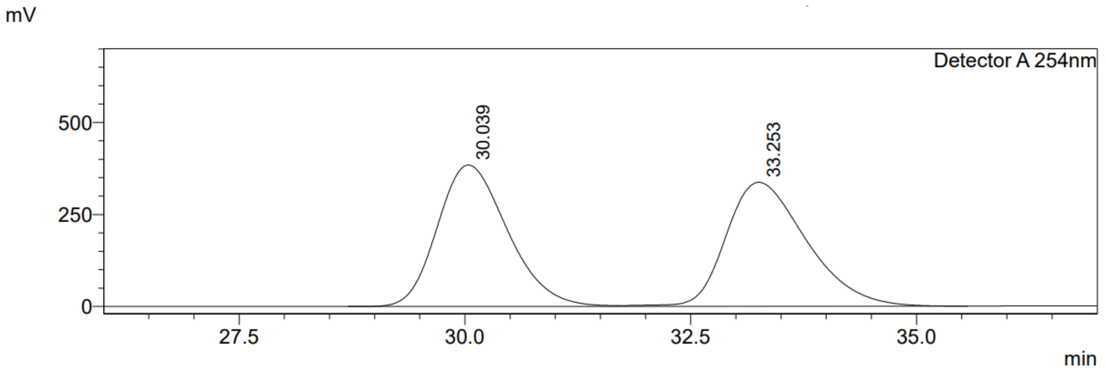

Detector A 254nm

Peak#	Ret. Time	Height	Height%	Area	Area%
1	30.039	384128	53.295	20787257	49.915
2	33.253	336623	46.705	20858174	50.085
Total		720751	100.000	41645431	100.000

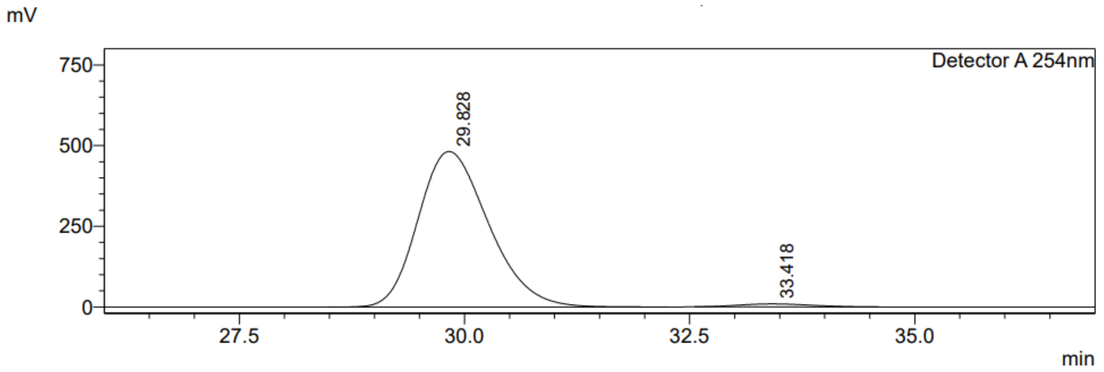

Detector A 254nm

Peak#	Ret. Time	Height	Height%	Area	Area%
1	29.828	482160	98.132	26268271	98.081
2	33.418	9180	1.868	513862	1.919
Total		491340	100.000	26782133	100.000

(R)-2-(naphthalen-1-yl)-4,5-di-p-tolyl-3,6-dihydro-2H-1,2-thiazine 1-oxide (3)

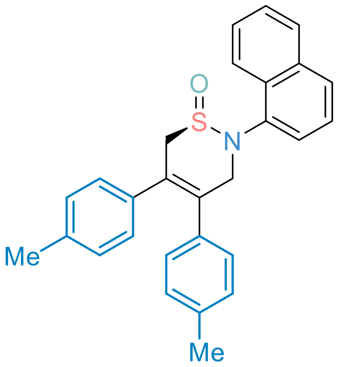

3

White solid; 41 mg, 98% yield; m.p. = 73.6-74.2 ºC; ^{1}H NMR (400 MHz, $CDCl_3$) δ 8.21 (d, J = 8.2 Hz, 1H), 7.91 (d, J = 7.8 Hz, 1H), 7.82 (d, J = 8.2 Hz, 1H), 7.72 (d, J = 7.0 Hz, 1H), 7.63-7.53 (m, 2H), 7.49 (t, J = 7.8 Hz, 1H), 7.10-6.94 (m, 8H), 4.70 (ddd, J = 17.1, 3.9, 2.5 Hz, 1H), 4.26 (ddd, J = 16.7, 3.9, 2.8 Hz, 1H), 4.10 (dd, J = 17.1, 2.1 Hz, 1H), 3.79 (dd, J = 16.7, 1.9 Hz, 1H), 2.29 (s, 3H), 2.25 (s, 3H); ^{13}C NMR (100 MHz, $CDCl_3$) δ 142.83, 137.88, 136.89, 136.68, 136.30, 134.73, 133.73, 130.47, 129.19, 129.17, 129.00, 128.90, 128.59, 127.79, 126.80, 126.60, 125.98, 123.69, 123.07, 122.88, 55.92, 49.45, 21.26, 21.24; HRMS (APCI) m/z calcd for $C_{28}H_{25}NOS$ $[M+H]^+$ = 424.1735, found = 424.1735.

Optical Rotation: $[\alpha]^{25}_{D}$ = 1184.53 (c = 0.53, $CHCl_3$). 95% e.e. (HPLC condition: Chiralpak IC column, n-Hexane/i-PrOH = 70:30, flow rate = 1.0 mL/min, wavelength = 254 nm, t_R = 17.19 min for minor isomer, t_R = 27.47 min for major isomer).

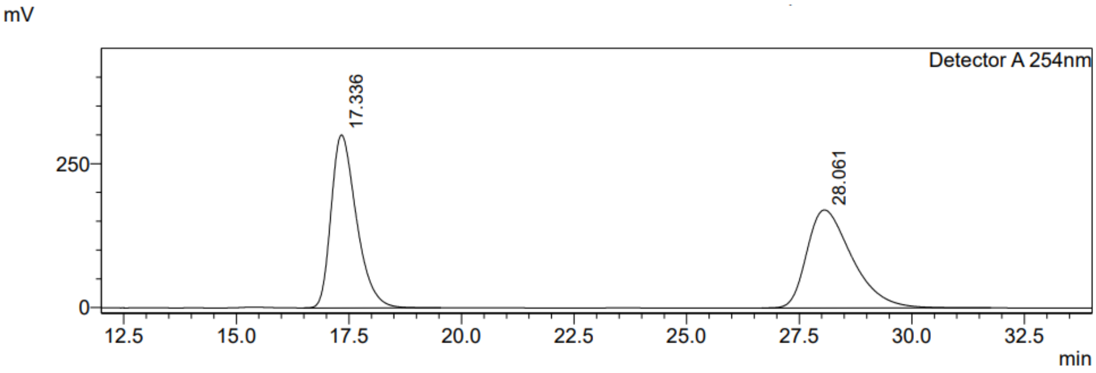

Detector A 254nm

Peak#	Ret. Time	Height	Height%	Area	Area%
1	17.336	300396	63.838	11764690	49.747
2	28.061	170166	36.162	11884201	50.253
Total		470563	100.000	23648892	100.000

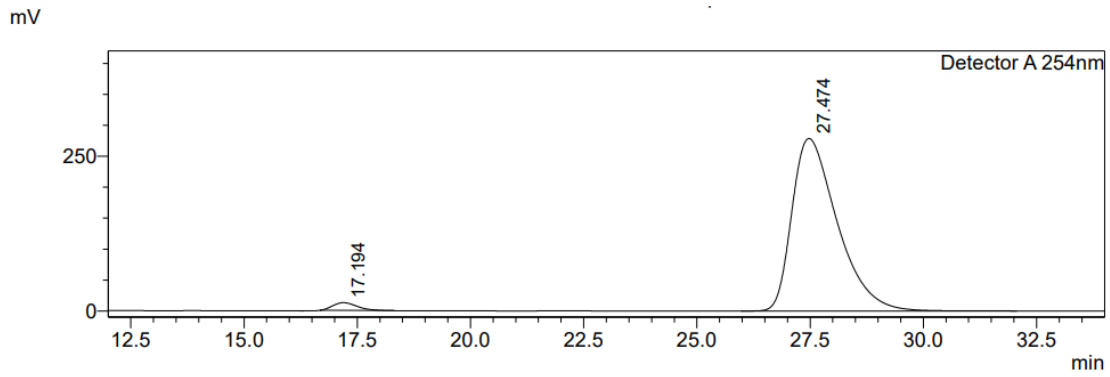

Detector A 254nm

Peak#	Ret. Time	Height	Height%	Area	Area%
1	17.194	12555	4.310	471650	2.371
2	27.474	278748	95.690	19418149	97.629
Total		291304	100.000	19889799	100.000

(*R*)-4,5-bis(4-chlorophenyl)-2-(naphthalen-1-yl)-3,6-dihydro-2*H*-1,2-thiazine 1-oxide (4)

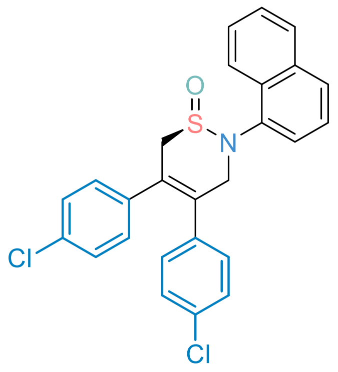

4

White solid; 35 mg, 76% yield; m.p. = 82.4-84.3 °C; ^{1}H NMR (400 MHz, $CDCl_3$) δ 8.16 (d, *J* = 8.2 Hz, 1H), 7.91 (d, *J* = 7.7 Hz, 1H), 7.83 (d, *J* = 8.2 Hz, 1H), 7.71 (d, *J* = 7.1 Hz, 1H), 7.63-7.53 (m, 2H), 7.49 (t, *J* = 7.7 Hz, 1H), 7.20 (d, *J* = 8.5 Hz, 2H), 7.14 (d, *J* = 8.5 Hz, 2H), 7.11-7.00 (m, 4H), 4.67 (ddd, *J* = 17.4, 4.2, 2.4 Hz, 1H), 4.24 (ddd, *J* = 16.7, 4.2, 2.9 Hz, 1H), 4.05 (dd, *J* = 17.4, 2.3 Hz, 1H), 3.74 (dd, *J* = 16.7, 2.0 Hz, 1H); ^{13}C NMR (100 MHz, $CDCl_3$) δ 142.47, 138.84, 137.16, 134.75, 133.83, 133.53, 133.29, 130.59, 130.39, 128.74, 128.69, 128.67, 128.08, 126.94, 126.71, 126.00, 123.92, 123.15, 122.64, 55.65, 49.27; HRMS (APCI) *m/z* calcd for $C_{26}H_{19}Cl_2NOS$ $[M+H]^+$ = 464.0642, found = 464.0637.

Optical Rotation: $[\alpha]^{25}_{D}$ = 145.74 (c = 0.54, $CHCl_3$). 91% e.e. (HPLC condition: Chiralpak IC column, n-Hexane/i-PrOH = 70:30, flow rate = 1.0 mL/min, wavelength = 254 nm, t_R = 9.21 min for minor isomer, t_R = 25.31 min for major isomer).

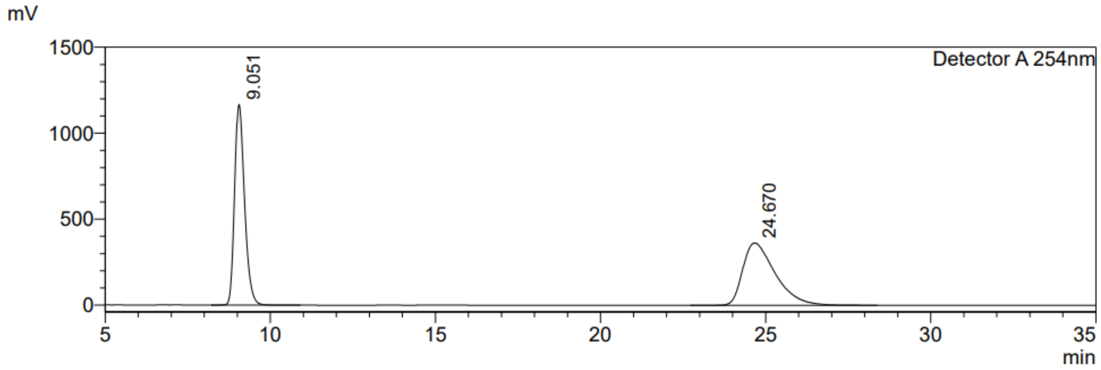

Detector A 254nm

Peak#	Ret. Time	Height	Height%	Area	Area%
1	9.051	1167068	76.297	24438582	49.676
2	24.670	362567	23.703	24757674	50.324
Total		1529635	100.000	49196256	100.000

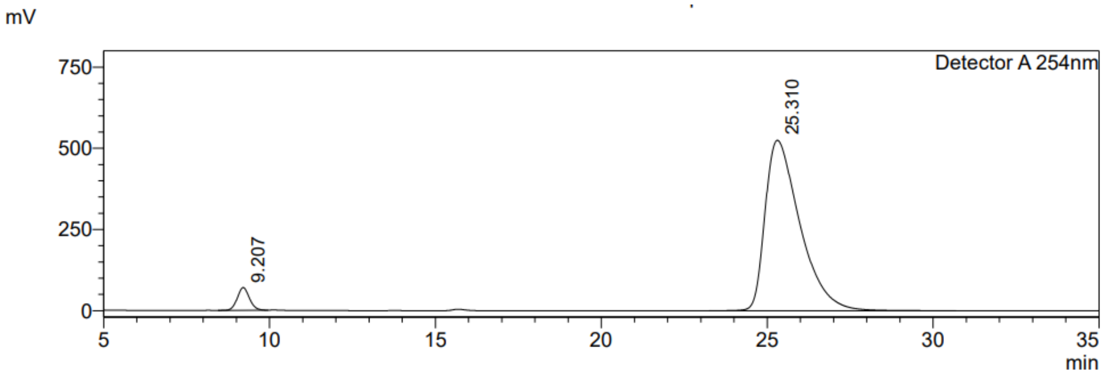

Detector A 254nm

Peak#	Ret. Time	Height	Height%	Area	Area%
1	9.207	70007	11.768	1775482	4.423
2	25.310	524908	88.232	38364089	95.577
Total		594915	100.000	40139571	100.000

(*R*)-4,5-bis(4-fluorophenyl)-2-(naphthalen-1-yl)-3,6-dihydro-2*H*-1,2-thiazine 1-oxide (5)

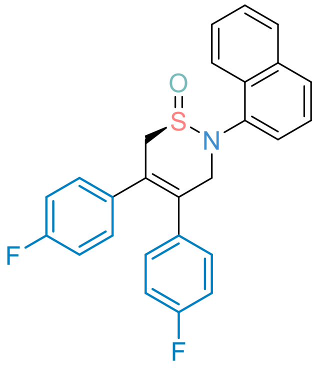

5

White solid; 30 mg, 70% yield; m.p. = 144.4-145.8 °C; ^{1}H NMR (400 MHz, $CDCl_3$) δ 8.17 (d, *J* = 8.2 Hz, 1H), 7.91 (d, *J* = 7.5 Hz, 1H), 7.83 (d, *J* = 8.2 Hz, 1H), 7.72 (d, *J* = 7.1 Hz, 1H), 7.64-7.53 (m, 2H), 7.53-7.43 (m, 1H), 7.19-7.02 (m, 4H), 6.97-6.80 (m, 4H), 4.68 (ddd, *J* = 17.3, 4.3, 2.5 Hz, 1H), 4.26 (ddd, *J* = 16.7, 4.3, 2.8 Hz, 1H), 4.07 (dd, *J* = 17.3, 2.4 Hz, 1H), 3.75 (dd, *J* = 16.7, 2.1 Hz, 1H); ^{13}C NMR (100 MHz, $CDCl_3$) δ 161.96 (d, *J* = 247.4 Hz), 161.82 (d, *J* = 247.3 Hz), 142.59, 136.45 (d, *J* = 3.4 Hz), 134.83 (d, *J* = 3.5 Hz), 134.77, 133.71, 131.02 (d, *J* = 2.6 Hz), 130.94 (d, *J* = 2.6 Hz), 130.44, 128.70, 128.03, 126.92, 126.70, 126.02, 123.76, 123.14, 122.69, 115.53 (d, *J* = 7.2 Hz), 115.32 (d, *J* = 7.2 Hz), 55.82, 49.46; ^{19}F NMR (376 MHz, $CDCl_3$) δ -114.04, -114.35; HRMS (ESI) *m/z* calcd for $C_{26}H_{19}F_2NOS$ $[M+H]^+$ = 432.1233 found = 432.1236.

Optical Rotation: $[\alpha]^{25}_{D}$ = 157.60 (*c* = 0.50, $CHCl_3$). 94% e.e. (HPLC condition: Chiralpak IC column, *n*-Hexane/*i*-PrOH = 70:30, flow rate = 1.0 mL/min, wavelength = 254 nm, t_R = 9.21 min for minor isomer, t_R = 25.76 min for major isomer).

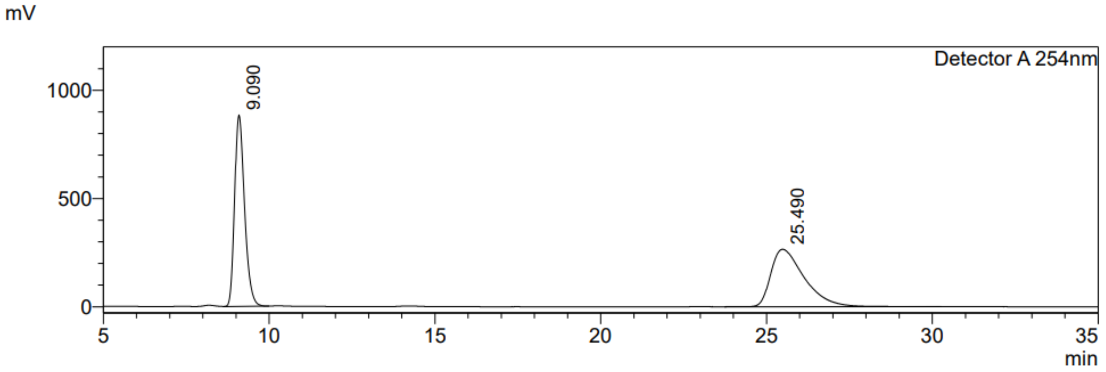

Detector A 254nm

Peak#	Ret. Time	Height	Height%	Area	Area%
1	9.090	882431	76.874	18393294	49.627
2	25.490	265459	23.126	18669837	50.373
Total		1147890	100.000	37063131	100.000

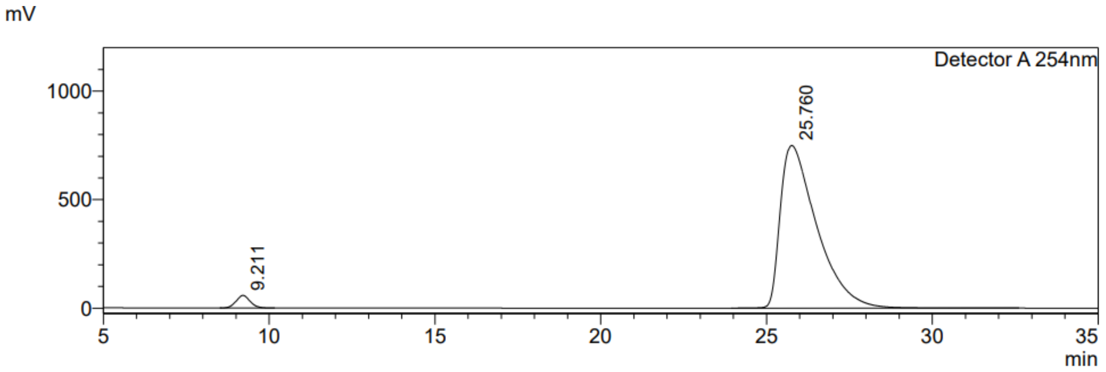

Detector A 254nm

Peak#	Ret. Time	Height	Height%	Area	Area%
1	9.211	58074	7.191	1700780	2.872
2	25.760	749555	92.809	57510593	97.128
Total		807629	100.000	59211373	100.000

(*R*)-4,5-bis(3-methoxyphenyl)-2-(naphthalen-1-yl)-3,6-dihydro-2*H*-1,2-thiazine 1-oxide (6)

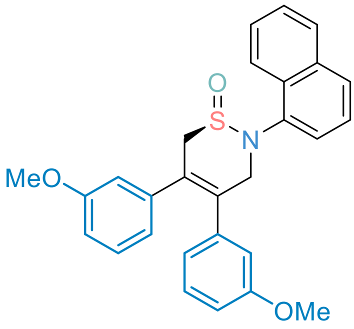

6

White solid; 37 mg, 81% yield; m.p. = 117.8-119.0 °C; ^{1}H NMR (400 MHz, $CDCl_3$) δ 8.20 (d, *J* = 8.3 Hz, 1H), 7.91 (d, *J* = 7.6 Hz, 1H), 7.82 (d, *J* = 8.3 Hz, 1H), 7.72 (d, *J* = 7.1 Hz, 1H), 7.63-7.53 (m, 2H), 7.53-7.45 (m, 1H), 7.14 (t, *J* = 7.9 Hz, 1H), 7.08 (t, *J*

= 7.9 Hz, 1H), 6.80 (d, *J* = 7.6 Hz, 1H), 6.75 (d, *J* = 7.6 Hz, 1H), 6.74-6.61 (m, 4H), 4.73 (ddd, *J* = 17.3, 4.3, 2.5 Hz, 1H), 4.29 (ddd, *J* = 16.8, 4.3, 2.7 Hz, 1H), 4.11 (dd, *J* = 17.3, 2.3 Hz, 1H), 3.80 (dd, *J* = 16.8, 2.1 Hz, 1H), 3.65 (s, 3H), 3.59 (s, 3H); ^{13}C NMR (100 MHz, $CDCl_3$) δ 159.41, 159.33, 142.70, 142.09, 140.48, 134.74, 134.16, 130.43, 129.32, 129.22, 128.62, 127.88, 126.85, 126.64, 125.98, 124.13, 123.06, 122.81, 121.52, 121.47, 114.61, 113.52, 113.31, 55.67, 55.29, 55.23, 49.20; HRMS (ESI) *m/z* calcd for $C_{28}H_{25}NO_3S$ $[M+H]^+$ = 456.1633, found = 456.1630.

Optical Rotation: $[\alpha]^{25}_D$ = 168.63 (*c* = 0.51, $CHCl_3$). 96% e.e. (HPLC condition: Chiralpak IE column, *n*-Hexane/*i*-PrOH = 70:30, flow rate = 1.0 mL/min, wavelength = 254 nm, t_R = 19.61 min for major isomer, t_R = 24.99 min for minor isomer).

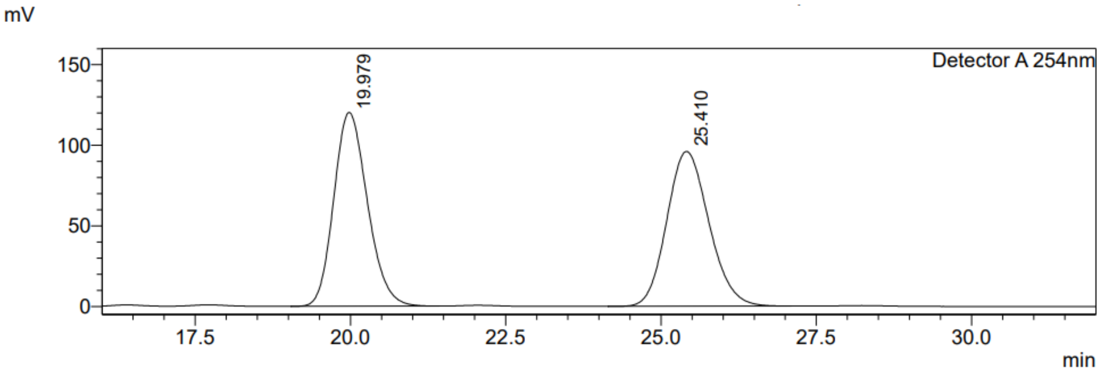

Detector A 254nm

Peak#	Ret. Time	Height	Height%	Area	Area%
1	19.979	120213	55.595	4533376	49.992
2	25.410	96016	44.405	4534870	50.008
Total		216229	100.000	9068247	100.000

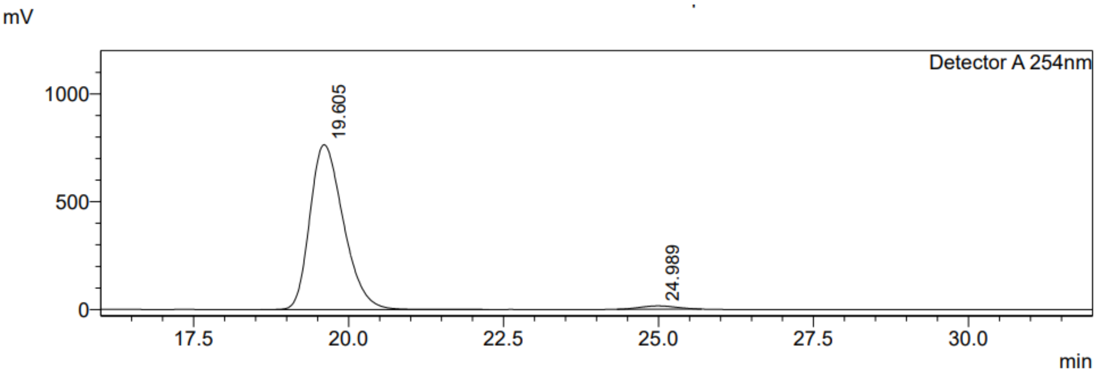

Detector A 254nm

Peak#	Ret. Time	Height	Height%	Area	Area%
1	19.605	764414	98.051	28759709	97.903
2	24.989	15193	1.949	615869	2.097
Total		779607	100.000	29375578	100.000

(*R*)-4,5-bis(3-chlorophenyl)-2-(naphthalen-1-yl)-3,6-dihydro-2*H*-1,2-thiazine 1-oxide (7)

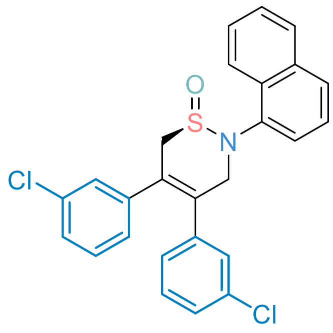

7

White solid; 41 mg, 89% yield; m.p. = 142.5-143.6 °C; ^{1}H NMR (400 MHz, $CDCl_3$) δ 8.15 (d, J = 8.3 Hz, 1H), 7.91 (d, J = 7.5 Hz, 1H), 7.83 (d, J = 8.3 Hz, 1H), 7.71 (d, J = 7.2 Hz, 1H), 7.65-7.54 (m, 2H), 7.54-7.45 (m, 1H), 7.24-7.12 (m, 5H), 7.09 (t, J = 7.7 Hz, 1H), 7.02 (dt, J = 6.9, 1.7 Hz, 1H), 7.01-6.95 (m, 1H), 4.68 (ddd, J = 17.4, 4.5, 2.5 Hz, 1H), 4.25 (ddd, J = 16.7, 4.5, 2.9 Hz, 1H), 4.06 (dd, J = 17.4, 2.4 Hz, 1H), 3.74 (dd, J = 16.7, 2.1 Hz, 1H); ^{13}C NMR (100 MHz, $CDCl_3$) δ 142.43, 142.11, 140.38, 134.76, 134.36, 134.26, 134.05, 130.41, 129.75, 129.69, 129.14, 129.07, 128.70, 128.13, 127.89, 127.68, 127.67, 127.61, 126.99, 126.74, 126.00, 124.13, 123.18, 122.63, 55.61, 49.25; HRMS (APCI) m/z calcd for $C_{26}H_{19}Cl_2NOS$ $[M+H]^+$ = 464.0642, found = 464.0637.

Optical Rotation: $[\alpha]^{25}_{D}$ = 152.86 (c = 0.49, $CHCl_3$). 93% e.e. (HPLC condition: Chiralpak IC column, n-Hexane/i-PrOH = 70:30, flow rate = 1.0 mL/min, wavelength = 254 nm, t_R = 10.96 min for minor isomer, t_R = 23.84 min for major isomer).

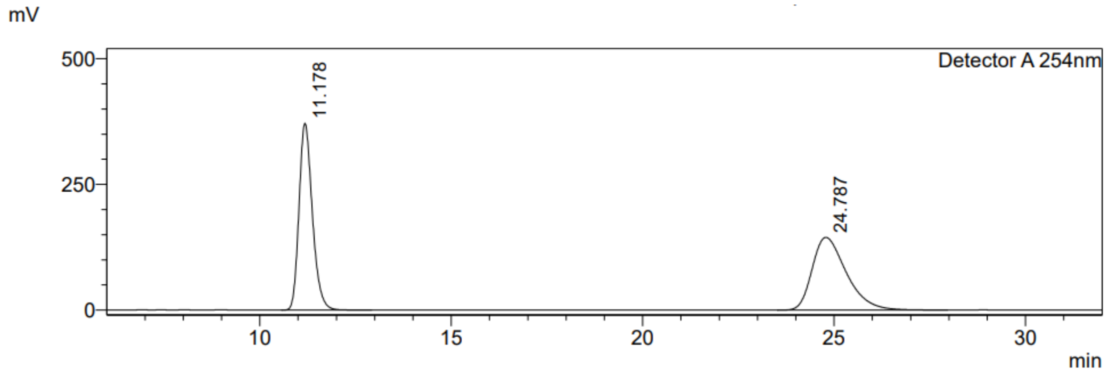

Detector A 254nm

Peak#	Ret. Time	Height	Height%	Area	Area%
1	11.178	371399	71.968	8986303	49.988
2	24.787	144661	28.032	8990526	50.012
Total		516060	100.000	17976829	100.000

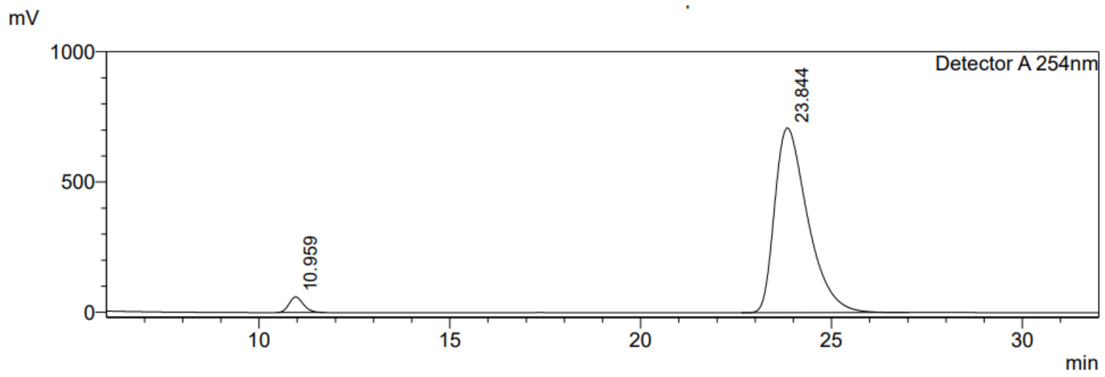

Detector A 254nm

Peak#	Ret. Time	Height	Height%	Area	Area%
1	10.959	59879	7.786	1559149	3.514
2	23.844	709153	92.214	42806292	96.486
Total		769032	100.000	44365441	100.000

(*R*)-4,5-bis(3-fluorophenyl)-2-(naphthalen-1-yl)-3,6-dihydro-2*H*-1,2-thiazine 1-oxide (8)

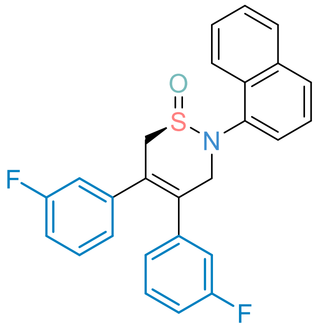

8

White solid; 34 mg, 79% yield; m.p. = 61.2-62.7 °C; ^{1}H NMR (400 MHz, $CDCl_3$) δ 8.17 (d, *J* = 8.3 Hz, 1H), 7.91 (d, *J* = 7.7 Hz, 1H), 7.83 (d, *J* = 8.3 Hz, 1H), 7.72 (d, *J* = 7.1 Hz, 1H), 7.65-7.54 (m, 2H), 7.54-7.43 (m, 1H), 7.23-7.08 (m, 2H), 7.02-6.81 (m, 6H), 4.70 (ddd, *J* = 17.4, 4.4, 2.5 Hz, 1H), 4.26 (ddd, *J* = 16.7, 4.4, 2.9 Hz, 1H), 4.07 (dd, *J* = 17.4, 2.4 Hz, 1H), 3.75 (dd, *J* = 16.7, 2.1 Hz, 1H); ^{13}C NMR (100 MHz, $CDCl_3$) δ 162.66 (d, *J* = 246.9 Hz), 162.57 (d, *J* = 246.8 Hz), 142.61, 142.50 (d, *J* = 6.1 Hz), 140.84 (d, *J* = 7.6 Hz), 134.76, 134.03, 130.41, 130.04 (d, *J* = 7.1 Hz), 129.96 (d, *J* = 7.1 Hz), 128.70, 128.09, 126.97, 126.72, 126.00, 125.02 (d, *J* = 3.3 Hz), 124.99 (d, *J* = 3.4 Hz), 124.13, 123.15, 122.65, 116.17 (d, *J* = 21.9 Hz), 114.78, 114.57, 114.36, 55.60, 49.19; ^{19}F NMR (376 MHz, $CDCl_3$) δ -112.63, -112.71; HRMS (ESI) *m/z* calcd for $C_{26}H_{19}F_2NOS$ $[M+H]^+$ = 432.1233 found = 432.1236.

Optical Rotation: $[\alpha]^{25}_{D}$ = 143.08 (*c* = 0.52, $CHCl_3$). 92% e.e. (HPLC condition: Chiralpak IC column, *n*-Hexane/*i*-PrOH = 70:30, flow rate = 1.0 mL/min, wavelength = 254 nm, t_R = 9.60 min for minor isomer, t_R = 21.59 min for major isomer).

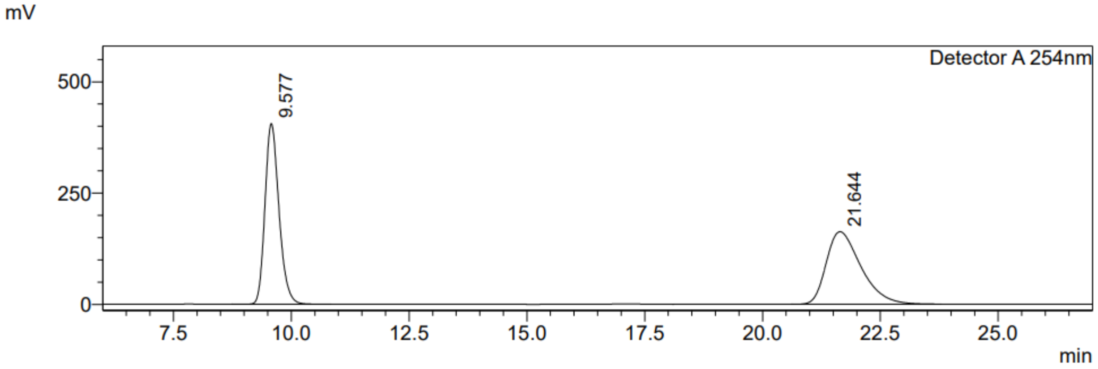

Detector A 254nm

Peak#	Ret. Time	Height	Height%	Area	Area%
1	9.577	406153	71.353	8382571	50.079
2	21.644	163065	28.647	8356059	49.921
Total		569218	100.000	16738630	100.000

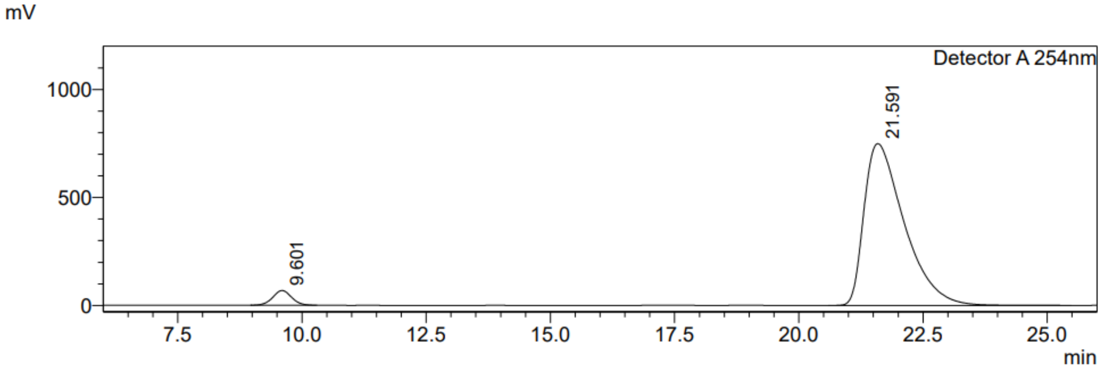

Detector A 254nm

Peak#	Ret. Time	Height	Height%	Area	Area%
1	9.601	68334	8.358	1807617	4.114
2	21.591	749285	91.642	42127323	95.886
Total		817619	100.000	43934940	100.000

(*R*)-4,5-bis(2-chlorophenyl)-2-(naphthalen-1-yl)-3,6-dihydro-2*H*-1,2-thiazine 1-oxide (9)

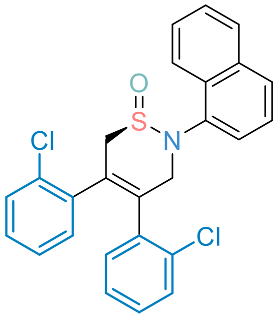

9

White solid; 46 mg, 99% yield; m.p. = 78.9-79.9 °C; ^{1}H NMR (400 MHz, $CDCl_3$) δ 8.36 (d, *J* = 8.0 Hz, 1H), 7.89 (d, *J* = 8.0 Hz, 1H), 7.82-7.76 (m, 2H), 7.62 (t, *J* = 7.3 Hz, 1H), 7.57-7.53 (m, 1H), 7.48 (t, *J* = 7.8 Hz, 1H), 7.35-7.28 (m, 3H), 7.23 (d, *J* = 7.1 Hz, 1H), 7.10-7.03 (m, 4H), 4.57 (d, *J* = 16.1 Hz, 1H), 4.17 (d, *J* = 17.1 Hz, 1H), 4.05 (d, *J* = 16.3 Hz, 1H), 3.48 (d, *J* = 16.3 Hz, 1H); ^{13}C NMR (100 MHz, $CDCl_3$) δ 143.03, 138.29, 137.10, 134.63, 134.35, 133.09, 132.40, 130.43, 130.15, 129.93, 129.87, 129.68, 129.28, 129.08, 128.50, 128.43, 127.85, 127.26, 126.88, 126.62, 125.92, 124.98, 123.45, 123.03, 54.65, 53.31; HRMS (APCI) *m/z* calcd for $C_{26}H_{19}Cl_2NOS$ $[M+H]^+$ = 464.0642, found = 464.0637.

Optical Rotation: $[\alpha]^{25}_D$ = 149.55 (*c* = 0.44, $CHCl_3$). 90% e.e. (HPLC condition: Chiralpak IC column, *n*-Hexane/*i*-PrOH = 70:30, flow rate = 1.0 mL/min, wavelength = 254 nm, t_R = 10.58 min for minor isomer, t_R = 21.61 min for major isomer).

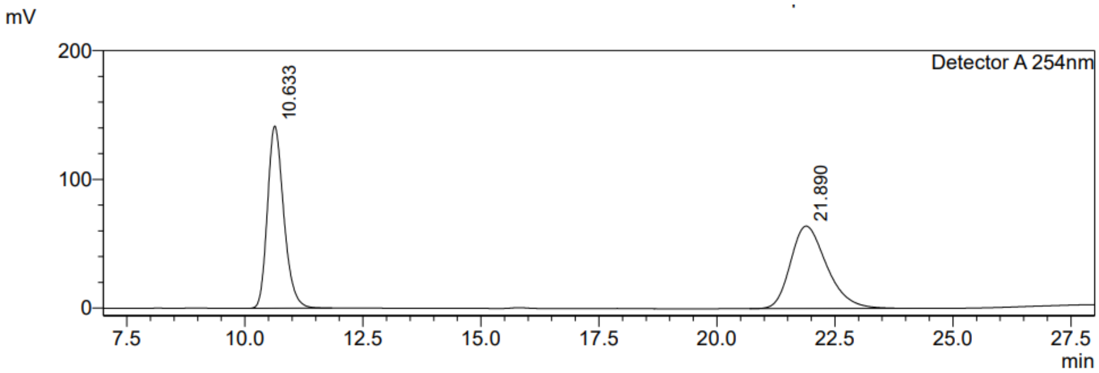

Detector A 254nm

Peak#	Ret. Time	Height	Height%	Area	Area%
1	10.633	141579	68.841	3407039	49.832
2	21.890	64082	31.159	3430004	50.168
Total		205661	100.000	6837043	100.000

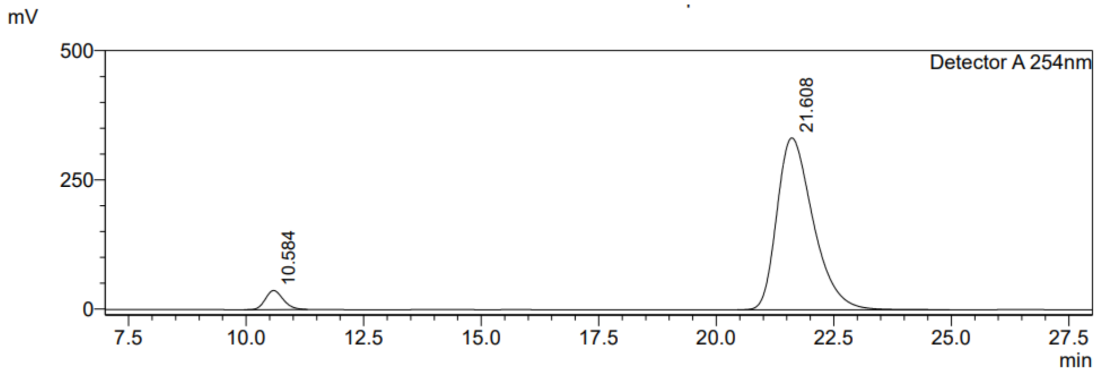

Detector A 254nm

Peak#	Ret. Time	Height	Height%	Area	Area%
1	10.584	36858	9.974	972212	5.166
2	21.608	332684	90.026	17848927	94.834
Total		369542	100.000	18821139	100.000

(*R*)-4,5-bis(3,4-dimethoxyphenyl)-2-(naphthalen-1-yl)-3,6-dihydro-2*H*-1,2-thiazine 1-oxide (10)

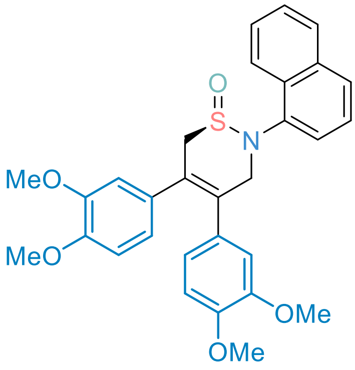

10

White solid; 44 mg, 85% yield; m.p. = 78.6-79.8 °C; ^{1}H NMR (400 MHz, $CDCl_3$) δ 8.19 (d, *J* = 8.1 Hz, 1H), 7.90 (d, *J* = 7.8 Hz, 1H), 7.82 (d, *J* = 8.1 Hz, 1H), 7.71 (d, *J* = 5.8 Hz, 1H), 7.62-7.52 (m, 2H), 7.49 (t, *J* = 7.8 Hz, 1H), 6.78-6.65 (m, 4H), 6.64 (s, 1H), 6.58 (s, 1H), 4.70 (d, *J* = 17.1 Hz, 1H), 4.27 (d, *J* = 16.7 Hz, 1H), 4.12 (d, *J* = 17.1 Hz, 1H), 3.83 (s, 3H), 3.83-3.80 (m, 1H), 3.79 (s, 3H), 3.65 (s, 3H), 3.58 (s, 3H); ^{13}C NMR (100 MHz, $CDCl_3$) δ 148.53, 148.49, 148.17, 148.04, 142.70, 134.71, 133.41, 131.87, 130.42, 128.60, 127.84, 126.77, 126.61, 125.97, 123.05, 122.82, 121.35, 121.23, 112.97, 112.87, 110.87, 110.74, 55.90, 55.86, 55.81, 55.66, 49.24; HRMS (ESI) *m/z* calcd for $C_{30}H_{29}NO_5S$ $[M+H]^+$ = 516.1844, found = 516.1836.

Optical Rotation: $[\alpha]^{25}_{D}$ = 168.63 (*c* = 0.51, $CHCl_3$). 95% e.e. (HPLC condition: Chiralpak IE column, *n*-Hexane/*i*-PrOH = 70:30, flow rate = 1.0 mL/min, wavelength = 254 nm, t_R = 63.97 min for minor isomer, t_R = 74.85 min for major isomer).

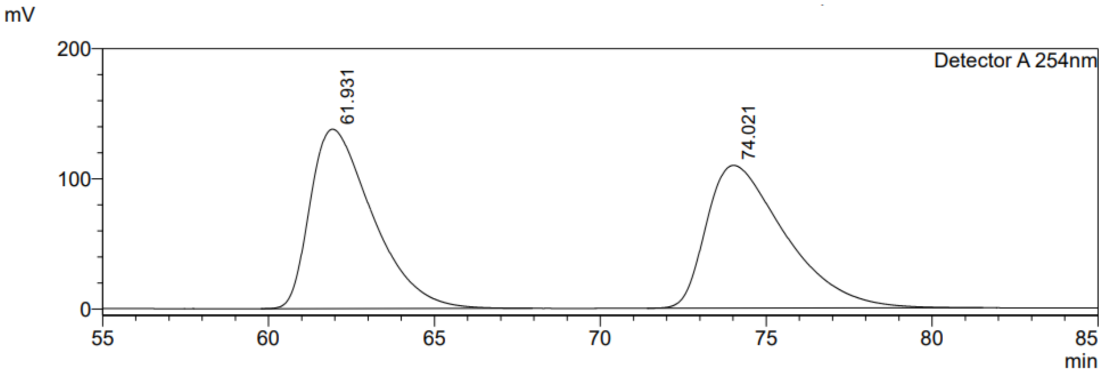

Detector A 254nm

Peak#	Ret. Time	Height	Height%	Area	Area%
1	61.931	137862	55.690	18071482	49.883
2	74.021	109690	44.310	18156489	50.117
Total		247552	100.000	36227971	100.000

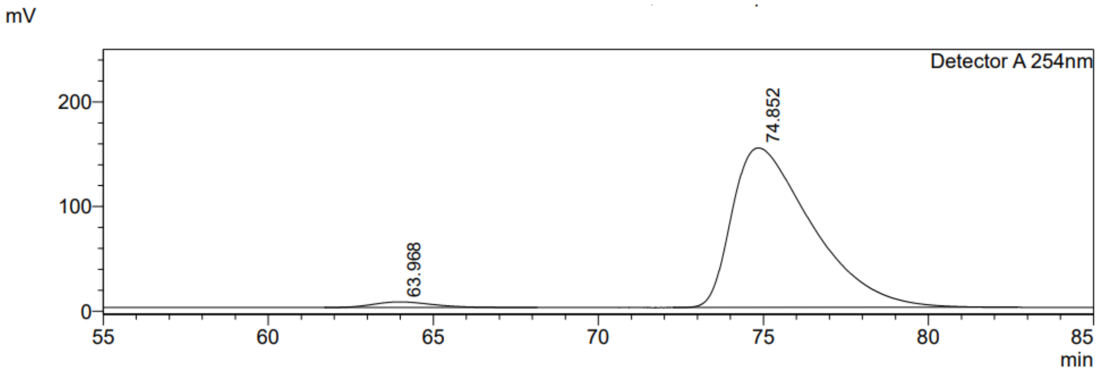

Detector A 254nm

Peak#	Ret. Time	Height	Height%	Area	Area%
1	63.968	5399	3.420	689800	2.609
2	74.852	152473	96.580	25749127	97.391
Total		157871	100.000	26438927	100.000

(*R*)-4,5-bis(3,4-difluorophenyl)-2-(naphthalen-1-yl)-3,6-dihydro-2*H*-1,2-thiazine 1-oxide (11)

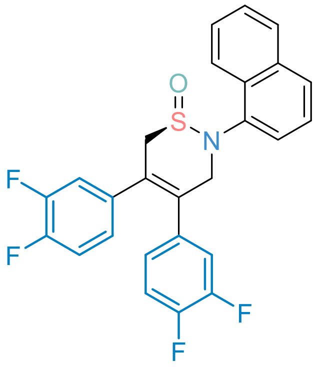

11

White solid; 37 mg, 79% yield; m.p. = 65.6-67.2 ºC; ^{1}H NMR (400 MHz, $CDCl_3$) δ 8.13 (d, *J* = 8.2 Hz, 1H), 7.92 (dd, *J* = 8.2, 1.0 Hz, 1H), 7.84 (d, *J* = 8.2 Hz, 1H), 7.70

(d, J = 7.2 Hz, 1H), 7.64-7.53 (m, 2H), 7.50 (dd, J = 8.2, 7.5 Hz, 1H), 7.07-6.92 (m, 4H), 6.90-6.80 (m, 2H), 4.65 (ddd, J = 17.5, 4.6, 2.5 Hz, 1H), 4.23 (ddd, J = 16.7, 4.6, 2.9 Hz, 1H), 4.03 (dd, J = 17.5, 2.4 Hz, 1H), 3.70 (dd, J = 16.7, 2.0 Hz, 1H); ^{13}C NMR (100 MHz, $CDCl_3$) δ 150.20 (ddd, J = 249.6, 12.6, 6.1 Hz), 149.74 (dt, J = 250.0, 12.8 Hz), 142.29, 137.11 (dd, J = 5.6, 4.3 Hz), 135.36 (dd, J = 5.8, 4.2 Hz), 134.79, 133.67, 130.37, 128.76, 128.23, 127.03, 126.78, 126.00, 125.56 (dd, J = 5.5, 2.6 Hz), 125.47 (dd, J = 6.1, 3.6 Hz), 123.86, 123.16, 122.53, 118.31 (d, J = 2.0 Hz), 118.13 (d, J = 2.0 Hz), 117.66 (d, J = 6.2 Hz), 117.49 (d, J = 6.1 Hz), 55.62, 49.29; ^{19}F NMR (376 MHz, $CDCl_3$) δ -136.67 (d, J = 21.3 Hz), -136.77 (d, J = 21.3 Hz), -137.96 (d, J = 21.3 Hz), -138.28 (d, J = 21.3 Hz); HRMS (APCI) *m/z* calcd for $C_{26}H_{17}F_4NOS$ $[M+H]^+$ = 468.1045, found = 468.1040.

Optical Rotation: $[\alpha]^{25}_D$ = 139.40 (c = 0.50, $CHCl_3$). 92% e.e. (HPLC condition: Chiralpak IC column, n-Hexane/i-PrOH = 70:30, flow rate = 1.0 mL/min, wavelength = 254 nm, t_R = 7.40 min for minor isomer, t_R = 16.11 min for major isomer).

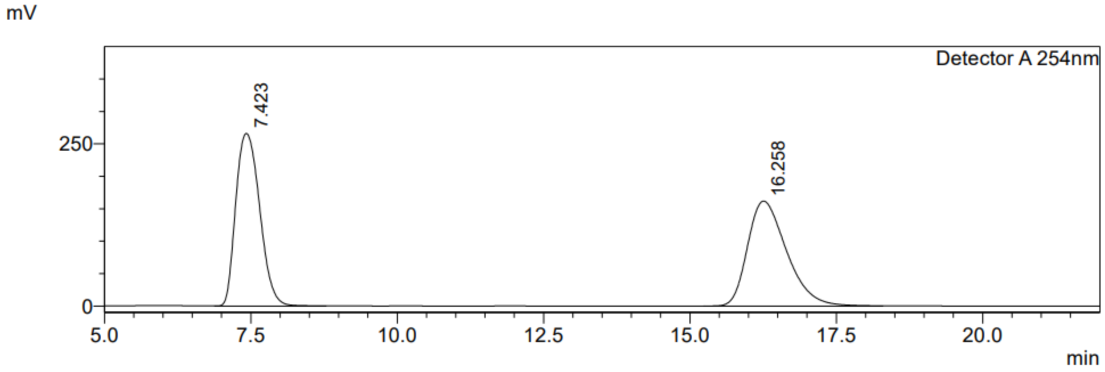

Detector A 254nm

Peak#	Ret. Time	Height	Height%	Area	Area%
1	7.423	266193	62.198	7596257	50.247
2	16.258	161787	37.802	7521521	49.753
Total		427981	100.000	15117777	100.000

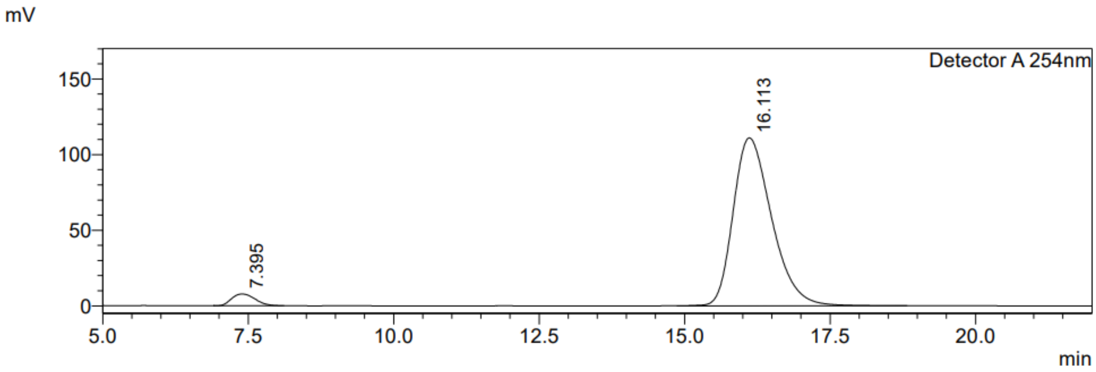

Detector A 254nm

Peak#	Ret. Time	Height	Height%	Area	Area%
1	7.395	7772	6.541	220556	4.139
2	16.113	111047	93.459	5108262	95.861
Total		118819	100.000	5328818	100.000

(*R*)-4,5-bis(2,4-dichlorophenyl)-2-(naphthalen-1-yl)-3,6-dihydro-2*H*-1,2-thiazine 1-oxide (12)

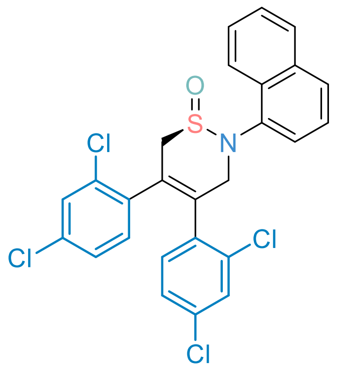

12

White solid; 52 mg, 98% yield; m.p. = 138.8-139.5 ºC; ^{1}H NMR (400 MHz, $CDCl_3$) δ 8.28 (d, *J* = 7.8 Hz, 1H), 7.89 (d, *J* = 8.0 Hz, 1H), 7.81 (d, *J* = 8.2 Hz, 1H), 7.74 (d, *J* = 7.1 Hz, 1H), 7.63-7.52 (m, 2H), 7.47 (t, *J* = 7.8 Hz, 1H), 7.32 (s, 1H), 7.25-7.01 (m, 5H), 4.52 (d, *J* = 15.9 Hz, 1H), 4.43 (d, *J* = 17.2 Hz, 1H), 4.01 (d, *J* = 17.2 Hz, 1H), 3.78 (d, *J* = 16.8 Hz, 1H); ^{13}C NMR (100 MHz, $CDCl_3$) δ 142.74, 136.59, 135.37, 134.64, 134.14, 133.79, 133.04, 132.54, 131.03, 130.78, 130.56, 130.32, 129.75, 129.30, 129.14, 128.52, 128.03, 127.49, 126.78, 126.68, 125.93, 124.82, 123.39, 122.81, 54.47, 53.24; HRMS (ESI) *m/z* calcd for $C_{26}H_{17}Cl_4NOS$ $[M+H]^+$ = 531.9863, found = 531.9864.

Optical Rotation: $[\alpha]^{25}_{D}$ = 124.81 (c = 0.52, $CHCl_3$). 87% e.e. (HPLC condition: Chiralpak IC column, n-Hexane/i-PrOH = 70:30, flow rate = 1.0 mL/min, wavelength = 254 nm, t_R = 6.33 min for minor isomer, t_R = 93.67 min for major isomer).

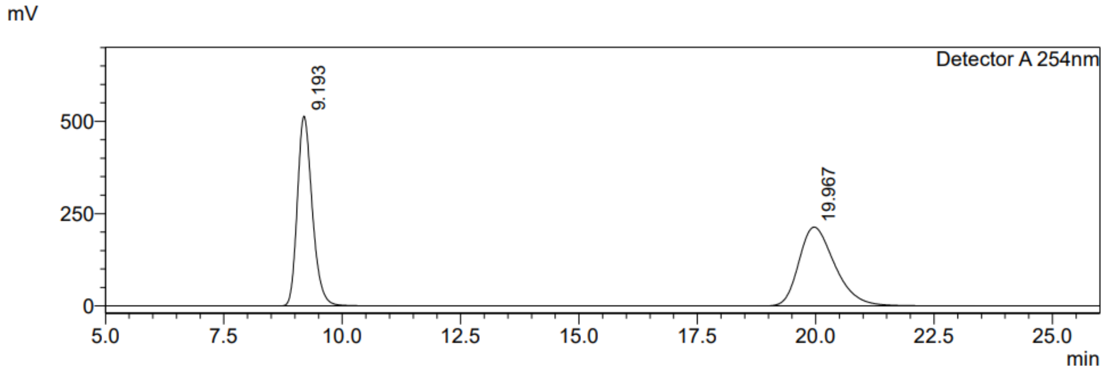

Detector A 254nm

Peak#	Ret. Time	Height	Height%	Area	Area%
1	9.193	513686	70.657	11263080	49.927
2	19.967	213331	29.343	11296191	50.073
Total		727017	100.000	22559270	100.000

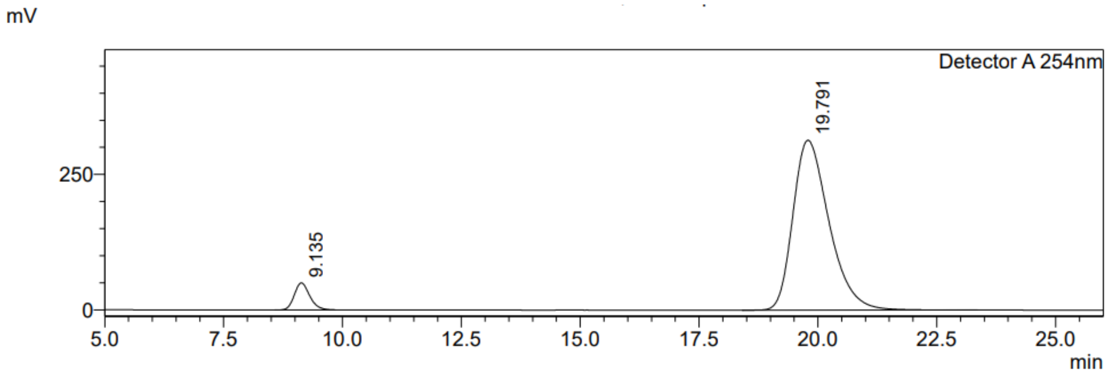

Detector A 254nm

Peak#	Ret. Time	Height	Height%	Area	Area%
1	9.135	49944	13.740	1121216	6.332
2	19.791	313547	86.260	16584534	93.668
Total		363491	100.000	17705750	100.000

(*R*)-2-(4-(benzyloxy)naphthalen-1-yl)-4,5-diphenyl-3,6-dihydro-2*H*-1,2-thiazine 1-oxide (13)

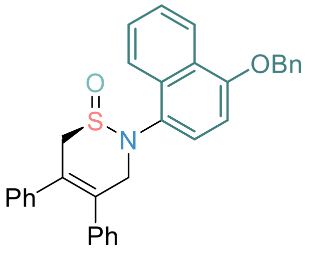

13

White solid; 49 mg, 98% yield; m.p. = 212.8-214.5 °C; ^{1}H NMR (400 MHz, $CDCl_3$) δ 8.43 (d, J = 8.3 Hz, 1H), 8.17 (d, J = 7.0 Hz, 1H), 7.79-7.60 (m, 2H), 7.60-7.51 (m, 3H), 7.44 (t, J = 7.4 Hz, 2H), 7.37 (t, J = 7.2 Hz, 1H), 7.24-7.07 (m, 10H), 6.88 (d, J = 8.2 Hz, 1H), 5.27 (s, 2H), 4.68 (d, J = 17.2 Hz, 1H), 4.35-4.20 (m, 1H), 4.08 (dd, J = 17.2, 1.7 Hz, 1H), 3.80 (dd, J = 16.7, 1.3 Hz, 1H); ^{13}C NMR (100 MHz, $CDCl_3$) δ 154.10, 140.83, 139.18, 136.86, 135.78, 134.29, 131.59, 129.36, 129.34, 128.76, 128.26, 128.16, 127.51, 127.44, 127.28, 127.07, 126.66, 126.01, 123.83, 122.99, 122.51, 104.89, 70.41, 56.01, 49.84; HRMS (ESI) m/z calcd for $C_{33}H_{27}NO_2S$ $[M+H]^+$ = 502.1840, found = 502.1835.

Optical Rotation: $[\alpha]^{25}_{D}$ = 142.29 (c = 0.48, $CHCl_3$). 94% e.e. (HPLC condition: Chiralpak IG column, n-Hexane/i-PrOH = 90:10, flow rate = 1.0 mL/min, wavelength = 254 nm, t_R = 70.89 min for major isomer, t_R = 81.21 min for minor isomer).

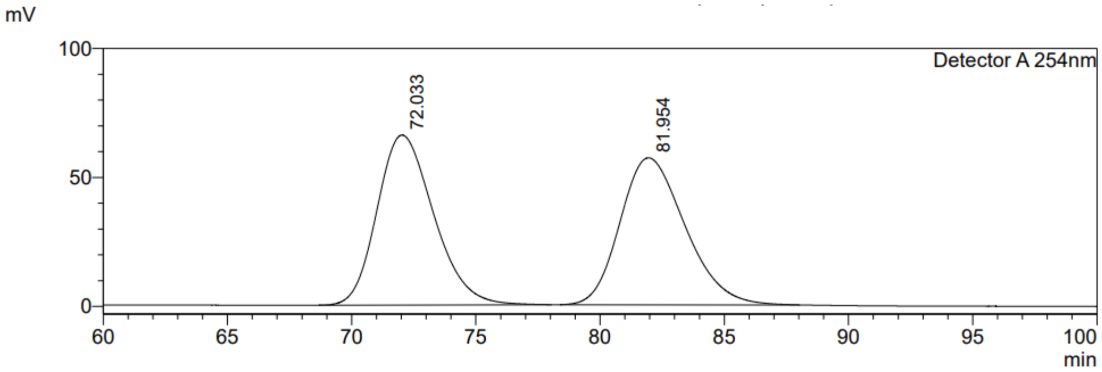

Detector A 254nm

Peak#	Ret. Time	Height	Height%	Area	Area%
1	72.033	65911	53.636	10449999	50.185
2	81.954	56974	46.364	10372940	49.815
Total		122885	100.000	20822939	100.000

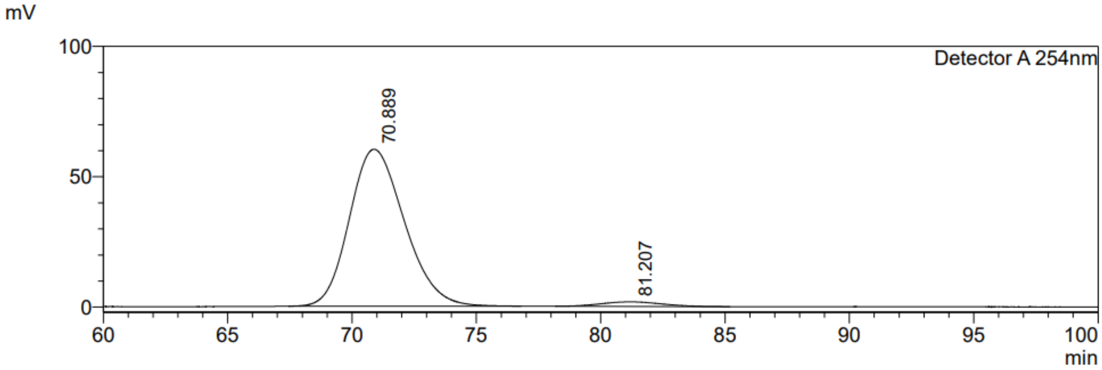

Detector A 254nm

Peak#	Ret. Time	Height	Height%	Area	Area%
1	70.889	60199	97.144	9273065	96.863
2	81.207	1770	2.856	300351	3.137
Total		61969	100.000	9573416	100.000

(*R*)-2-(4-ethoxynaphthalen-1-yl)-4,5-diphenyl-3,6-dihydro-2*H*-1,2-thiazine 1-oxide (14)

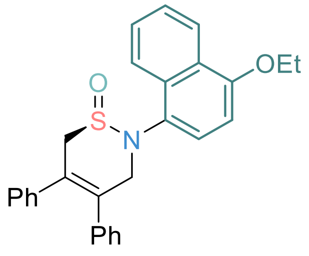

14

White solid; 43 mg, 98% yield; m.p. = 173.4-174.8 °C; ^{1}H NMR (400 MHz, $CDCl_3$) δ 8.37 (d, *J* = 8.0 Hz, 1H), 8.15 (d, *J* = 7.0 Hz, 1H), 7.90-7.64 (m, 1H), 7.62 (ddd, *J* = 8.3, 6.9, 1.3 Hz, 1H), 7.55 (ddd, *J* = 8.1, 6.9, 1.2 Hz, 1H), 7.23-7.10 (m, 10H), 6.79 (d, *J* = 8.2 Hz, 1H), 4.67 (d, *J* = 17.1 Hz, 1H), 4.31-4.25 (m, 1H), 4.23 (q, *J* = 7.0 Hz, 2H), 4.08 (dd, *J* = 17.1, 2.3 Hz, 1H), 3.80 (dd, *J* = 16.7, 2.0 Hz, 1H), 1.56 (t, *J* = 7.0 Hz, 3H); ^{13}C NMR (100 MHz, $CDCl_3$) δ 154.42, 140.86, 139.20, 135.31, 134.30, 131.52, 129.36, 129.34, 128.25, 128.15, 127.31, 127.26, 127.05, 126.58, 125.81, 123.96, 122.90, 122.44, 104.23, 64.08, 56.02, 49.89, 14.89; HRMS (ESI) *m/z* calcd for $C_{28}H_{25}NO_2S$ $[M+H]^+$ = 440.1684, found = 440.1676.

Optical Rotation: $[\alpha]^{25}_D$ = 146.33 (*c* = 0.49, $CHCl_3$). 98% e.e. (HPLC condition: Chiralpak ID column, *n*-Hexane/*i*-PrOH = 70:30, flow rate = 1.0 mL/min, wavelength = 254 nm, t_R = 12.92 min for major isomer, t_R = 14.31 min for minor isomer).

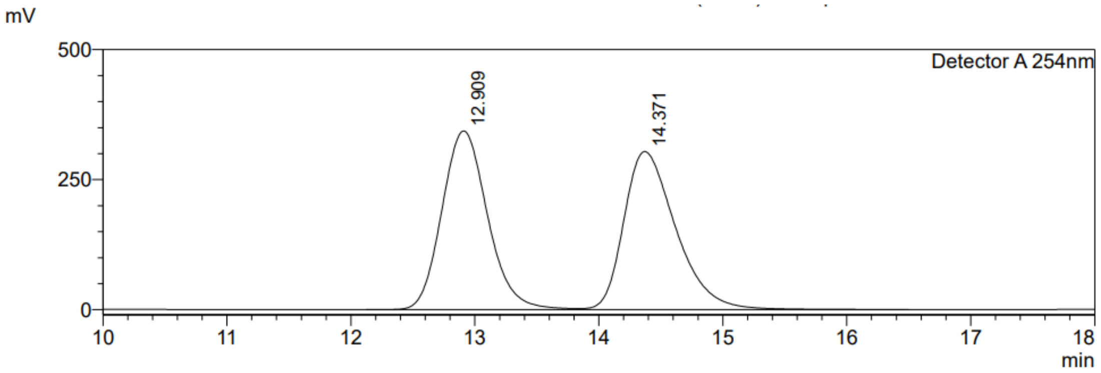

Detector A 254nm

Peak#	Ret. Time	Height	Height%	Area	Area%
1	12.909	343535	53.077	8785766	49.825
2	14.371	303699	46.923	8847401	50.175
Total		647234	100.000	17633167	100.000

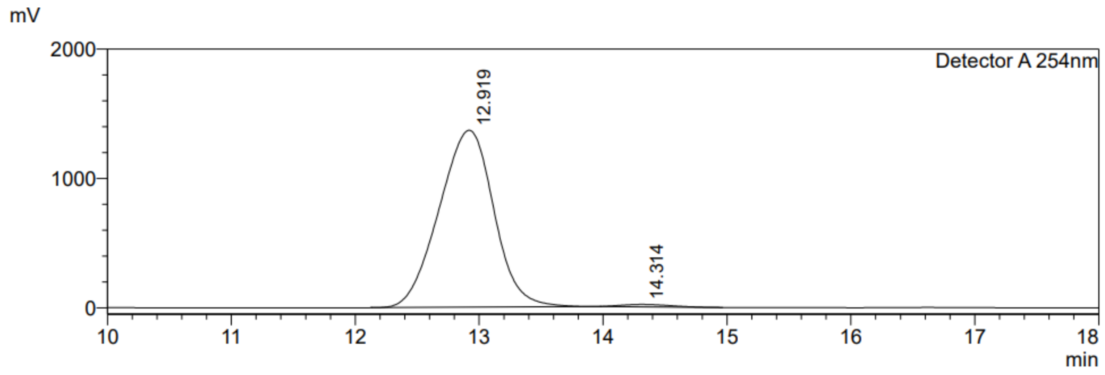

Detector A 254nm

Peak#	Ret. Time	Height	Height%	Area	Area%
1	12.919	1368064	98.684	42113682	98.832
2	14.314	18245	1.316	497630	1.168
Total		1386309	100.000	42611312	100.000

(*R*)-2-(4-methoxynaphthalen-1-yl)-4,5-diphenyl-3,6-dihydro-2*H*-1,2-thiazine 1-oxide (15)

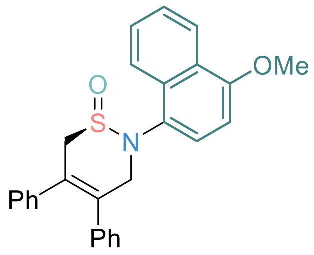

15

White solid; 42 mg, 98% yield; m.p. = 172.7-173.8 ºC; ^{1}H NMR (400 MHz, $CDCl_3$) δ 8.33 (d, *J* = 8.0 Hz, 1H), 8.15 (d, *J* = 7.0 Hz, 1H), 7.87-7.64 (m, 1H), 7.63 (ddd, *J* = 8.3, 7.0, 1.3 Hz, 1H), 7.55 (ddd, *J* = 8.1, 7.0, 1.2 Hz, 1H), 7.24-7.08 (m, 10H), 6.80 (d, *J* = 8.2 Hz, 1H), 4.67 (d, *J* = 17.2 Hz, 1H), 4.27 (ddd, *J* = 16.7, 4.3, 2.9 Hz, 1H), 4.07 (dd, *J* = 17.2, 2.3 Hz, 1H), 4.02 (s, 3H), 3.80 (dd, *J* = 16.7, 2.0 Hz, 1H); ^{13}C NMR (100 MHz, $CDCl_3$) δ 155.09, 140.85, 139.20, 135.57, 134.31, 131.49, 129.36, 129.35, 128.26, 128.16, 127.39, 127.28, 127.06, 126.46, 125.94, 123.94, 122.79, 122.50, 103.48, 56.04, 55.83, 49.89; HRMS (ESI) *m/z* calcd for $C_{27}H_{23}NO_2S$ $[M+H]^+$ = 426.1527, found = 426.1516.

Optical Rotation: $[\alpha]^{25}_D$ = 217.31 (*c* = 0.52, $CHCl_3$). 97% e.e. (HPLC condition: Chiralpak IF column, *n*-Hexane/*i*-PrOH =70:30, flow rate = 1.0 mL/min, wavelength = 254 nm, t_R = 9.80 min for major isomer, t_R = 13.01 min for minor isomer).

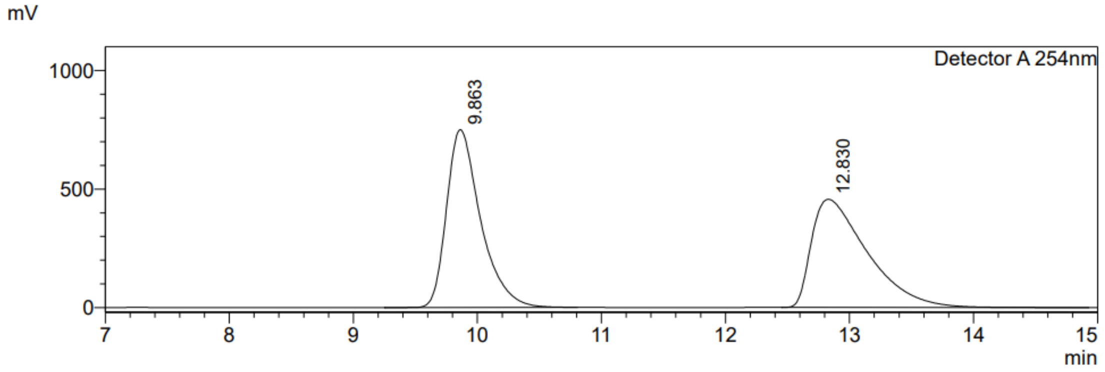

Detector A 254nm

Peak#	Ret. Time	Height	Height%	Area	Area%
1	9.863	749937	62.167	14301851	49.979
2	12.830	456396	37.833	14313775	50.021
Total		1206333	100.000	28615626	100.000

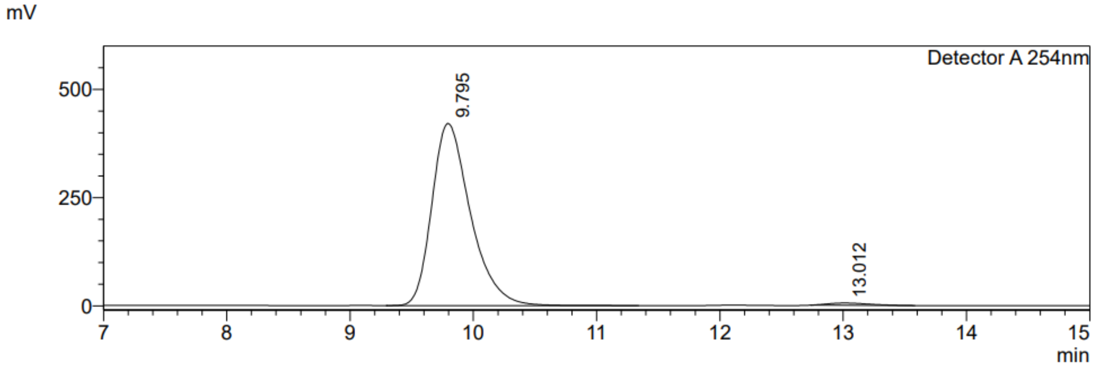

Detector A 254nm

Peak#	Ret. Time	Height	Height%	Area	Area%
1	9.795	420789	98.777	9160969	98.667
2	13.012	5211	1.223	123735	1.333
Total		426000	100.000	9284704	100.000

(*R*)-2-(4-methylnaphthalen-1-yl)-4,5-diphenyl-3,6-dihydro-2*H*-1,2-thiazine 1-oxide (16)

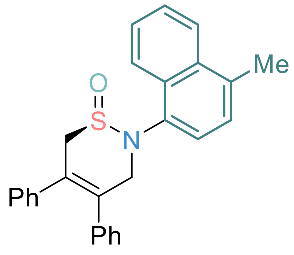

16

White solid; 37 mg, 91% yield; m.p. = 186.5-187.2 ºC; ^{1}H NMR (400 MHz, $CDCl_3$) δ 8.24 (d, *J* = 6.3 Hz, 1H), 8.12-7.93 (m, 1H), 7.76-7.55 (m, 3H), 7.34 (d, *J* = 7.5 Hz, 1H), 7.23-7.09 (m, 10H), 4.70 (dd, *J* = 17.3, 1.3 Hz, 1H), 4.28 (ddd, *J* = 16.7, 4.2, 2.8 Hz, 1H), 4.09 (dd, *J* = 17.3, 2.3 Hz, 1H), 3.80 (dd, *J* = 16.7, 2.0 Hz, 1H), 2.71 (s, 3H); ^{13}C

NMR (100 MHz, $CDCl_3$) δ 141.18, 140.80, 139.18, 134.51, 134.34, 133.71, 130.52, 129.37, 129.35, 128.28, 128.18, 127.31, 127.10, 126.62, 126.50, 126.47, 124.97, 123.32, 55.92, 49.61, 19.54; HRMS (ESI) *m/z* calcd for $C_{27}H_{23}NOS$ $[M+H]^+$ = 410.1578, found = 410.1576.

Optical Rotation: $[\alpha]^{25}_D$ = 115.09 (c = 0.55, $CHCl_3$). 95% e.e. (HPLC condition: Chiralpak IF column, *n*-Hexane/*i*-PrOH = 80:20, flow rate = 1.0 mL/min, wavelength = 254 nm, t_R = 12.74 min for major isomer, t_R = 16.47 min for minor isomer).

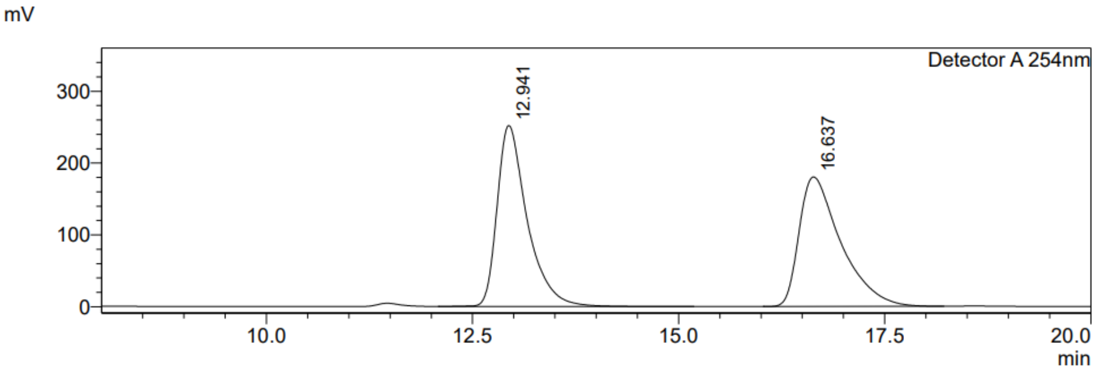

Detector A 254nm

Peak#	Ret. Time	Height	Height%	Area	Area%
1	12.941	251983	58.286	6394365	50.165
2	16.637	180337	41.714	6352404	49.835
Total		432321	100.000	12746769	100.000

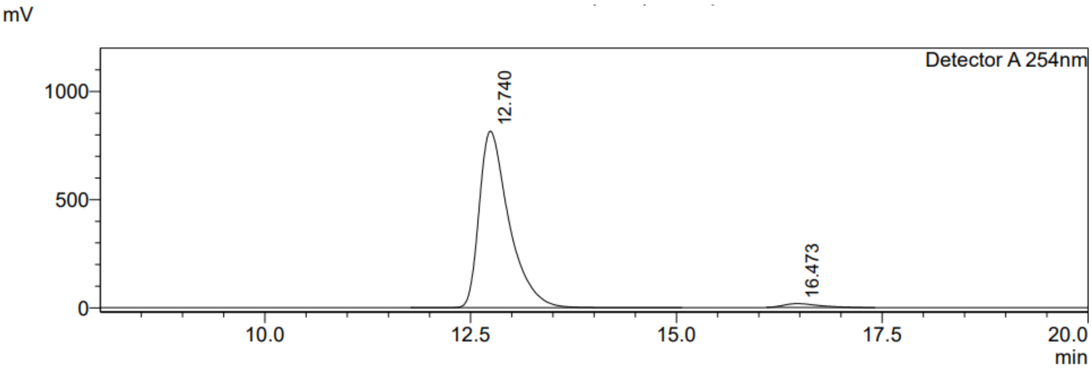

Detector A 254nm

Peak#	Ret. Time	Height	Height%	Area	Area%
1	12.740	816505	97.834	20608138	97.385
2	16.473	18079	2.166	553355	2.615
Total		834584	100.000	21161493	100.000

(*R*)-2-(4-bromonaphthalen-1-yl)-4,5-diphenyl-3,6-dihydro-2*H*-1,2-thiazine 1-oxide (17)

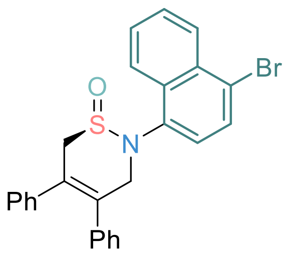

17

White solid; 42 mg, 88% yield; m.p. = 207.0-208.9 °C; ^{1}H NMR (400 MHz, $CDCl_3$) δ 8.37 – 8.26 (m, 1H), 8.23 (dd, J = 16.8, 10.3 Hz, 1H), 7.80 (d, J = 7.9 Hz, 1H), 7.73 – 7.63 (m, 2H), 7.61 (t, J = 11.7 Hz, 1H), 7.26 – 7.00 (m, 10H), 4.68 (ddd, J = 17.2, 4.2, 2.5 Hz, 1H), 4.29 (ddd, J = 16.8, 4.2, 2.7 Hz, 1H), 4.08 (dd, J = 17.2, 2.3 Hz, 1H), 3.81 (dd, J = 16.8, 2.1 Hz, 1H); ^{13}C NMR (100 MHz, $CDCl_3$) δ 142.74, 140.58, 138.98, 133.08, 131.73, 129.95, 129.33, 129.32, 128.33, 128.26, 128.11, 128.09, 127.69, 127.44, 127.21, 123.59, 123.36, 122.36, 55.85, 49.44; HRMS (ESI) *m/z* calcd for $C_{26}H_{20}BrNOS$ $[M+H]^+$ = 474.0527, found = 474.0520.

Optical Rotation: $[\alpha]^{25}_{D}$ = 156.94 (c = 0.49, $CHCl_3$). 91% e.e. (HPLC condition: Chiralpak IF column, *n*-Hexane/*i*-PrOH = 70:30, flow rate = 1.0 mL/min, wavelength = 254 nm, t_R = 9.50 min for major isomer, t_R = 12.56 min for minor isomer).

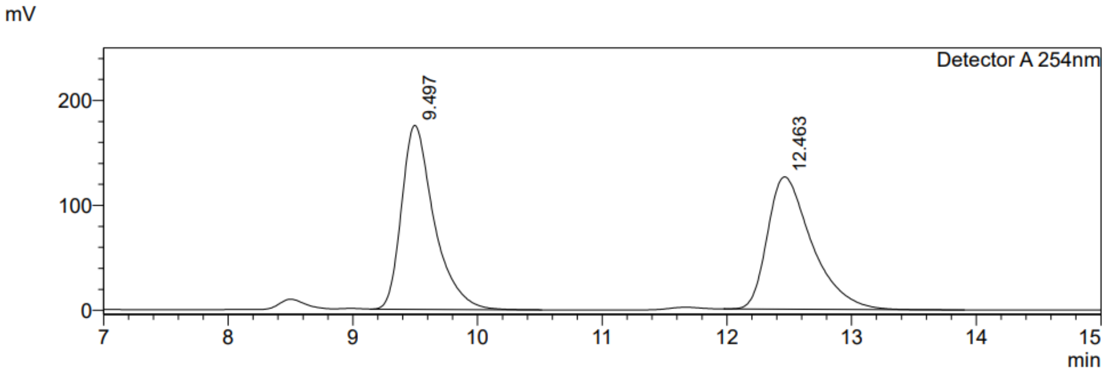

Detector A 254nm

Peak#	Ret. Time	Height	Height%	Area	Area%
1	9.497	175349	58.169	3179144	50.371
2	12.463	126098	41.831	3132360	49.629
Total		301447	100.000	6311504	100.000

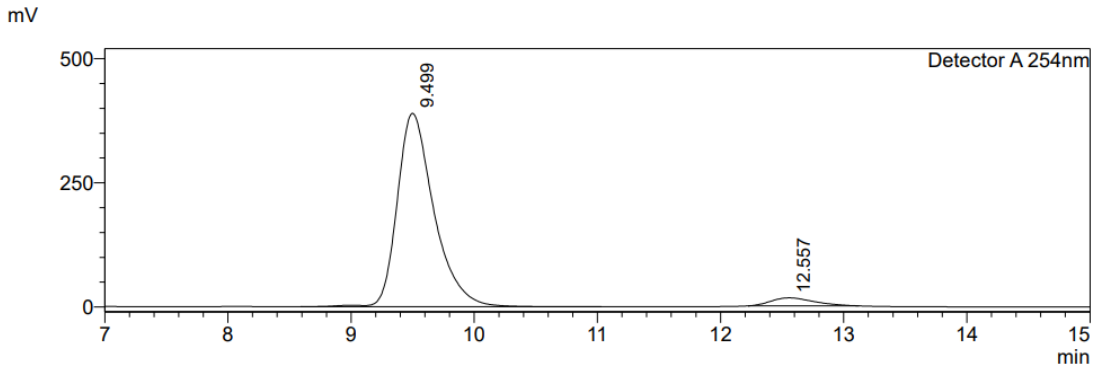

Detector A 254nm

Peak#	Ret. Time	Height	Height%	Area	Area%
1	9.499	389561	96.028	8063962	95.376
2	12.557	16115	3.972	390977	4.624
Total		405676	100.000	8454939	100.000

(*R*)-2-(4-fluoronaphthalen-1-yl)-4,5-diphenyl-3,6-dihydro-2*H*-1,2-thiazine 1-oxide (18)

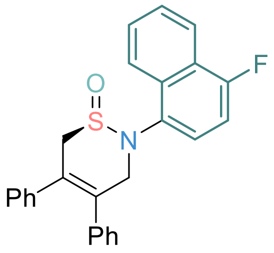

18

White solid; 33 mg, 80% yield; m.p. = 167.9-168.4 °C; ^{1}H NMR (400 MHz, $CDCl_3$) δ 8.20 (d, *J* = 7.9 Hz, 1H), 8.16 (d, *J* = 7.8 Hz, 1H), 7.78-7.55 (m, 3H), 7.24-7.06 (m, 11H), 4.68 (ddd, *J* = 17.2, 3.1, 2.4 Hz, 1H), 4.28 (ddd, *J* = 16.7, 4.1, 2.8 Hz, 1H), 4.07 (dd, *J* = 17.2, 2.3 Hz, 1H), 3.81 (dd, *J* = 16.7, 2.0 Hz, 1H); ^{13}C NMR (100 MHz, $CDCl_3$) δ 157.96 (d, *J* = 253.3 Hz), 140.65, 139.03, 138.81 (d, *J* = 3.8 Hz), 134.20, 131.89 (d, *J* = 5.4 Hz), 129.33, 129.31, 128.30, 128.23, 127.87, 127.39, 127.16, 126.99 (d, *J* = 1.8 Hz), 124.68 (d, *J* = 17.3 Hz), 123.50, 122.96, 121.37 (d, *J* = 5.1 Hz), 109.45 (d, *J* = 21.2 Hz), 55.93, 49.75; ^{19}F NMR (376 MHz, $CDCl_3$) δ -122.15; HRMS (ESI) *m/z* calcd for $C_{26}H_{20}FNOS$ $[M+H]^+$ = 414.1328, found = 414.1326.

Optical Rotation: $[\alpha]^{25}_D$ = 122.08 (*c* = 0.53, $CHCl_3$). 95% e.e. (HPLC condition: Chiralpak IF column, *n*-Hexane/*i*-PrOH = 80:20, flow rate = 1.0 mL/min, wavelength = 254 nm, t_R = 10.38 min for major isomer, t_R = 12.29 min for minor isomer).

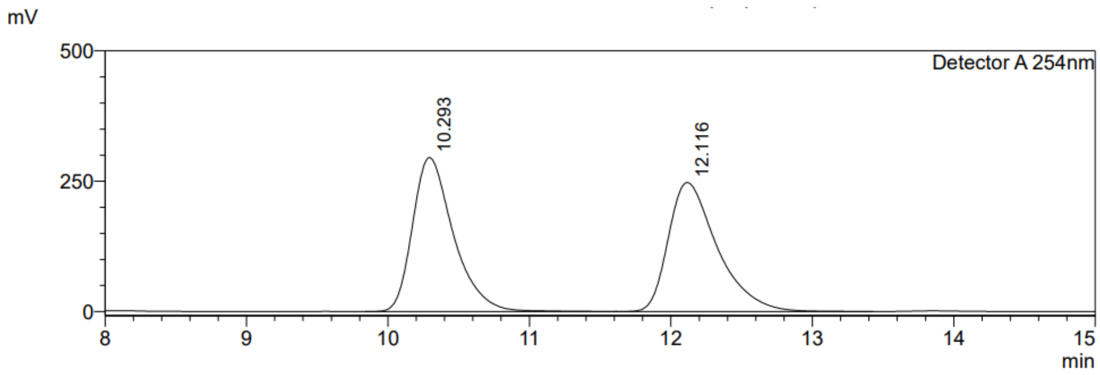

Detector A 254nm

Peak#	Ret. Time	Height	Height%	Area	Area%
1	10.293	295233	54.393	5945492	49.948
2	12.116	247543	45.607	5957773	50.052
Total		542776	100.000	11903265	100.000

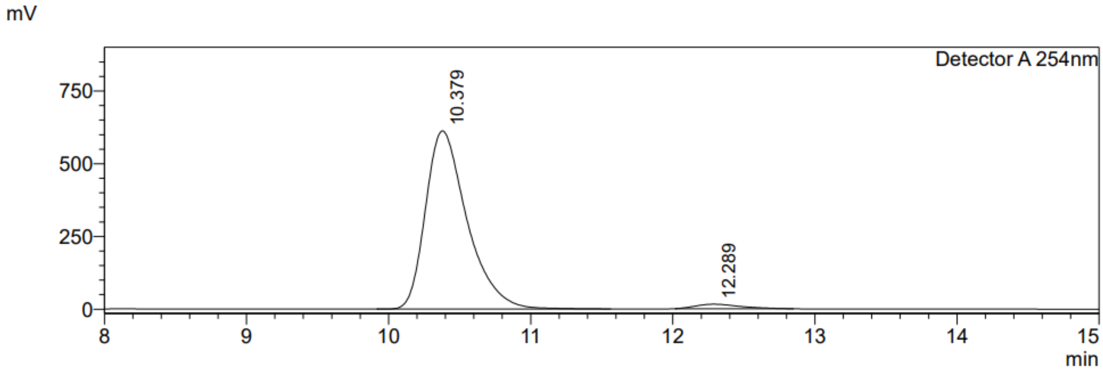

Detector A 254nm

Peak#	Ret. Time	Height	Height%	Area	Area%
1	10.379	612185	97.542	12442833	97.360
2	12.289	15425	2.458	337343	2.640
Total		627609	100.000	12780176	100.000

(*R*)-2-(naphthalen-2-yl)-4,5-diphenyl-3,6-dihydro-2*H*-1,2-thiazine 1-oxide (19)

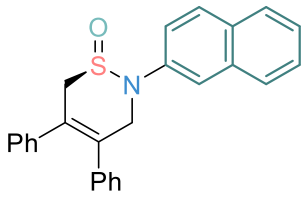

19

White solid; 35 mg, 88% yield; m.p. = 176.9-177.7 °C; ^{1}H NMR (400 MHz, $CDCl_3$) δ 7.87 (d, *J* = 8.9 Hz, 1H), 7.83 (d, *J* = 7.9 Hz, 1H), 7.79 (d, *J* = 7.9 Hz, 1H), 7.63 (d, *J* = 2.0 Hz, 1H), 7.54-7.42 (m, 3H), 7.26-7.07 (m, 10H), 4.74 (ddd, *J* = 16.8, 4.2, 2.4 Hz, 1H), 4.26 (dd, *J* = 16.8, 2.4 Hz, 1H), 4.14 (ddd, *J* = 16.6, 4.2, 2.8 Hz, 1H), 3.82 (dd, *J* = 16.6, 2.0 Hz, 1H); ^{13}C NMR (100 MHz, $CDCl_3$) δ 143.34, 140.55, 139.29, 133.89, 133.58, 131.10, 129.51, 129.38, 129.22, 128.31, 128.27, 127.73, 127.66, 127.45,

127.12, 126.85, 125.60, 124.13, 121.83, 118.05, 55.39, 46.86; HRMS (APCI) *m/z* calcd for $C_{26}H_{21}NOS$ $[M+H]^+$ = 396.1422, found = 396.1417

Optical Rotation: $[\alpha]^{25}_D$ = 31.43 (*c* = 0.49, $CHCl_3$). 66% e.e. (HPLC condition: Chiralpak IF column, *n*-Hexane/*i*-PrOH = 80:20, flow rate = 1.0 mL/min, wavelength = 254 nm, t_R = 19.42 min for major isomer, t_R = 21.49 min for minor isomer).

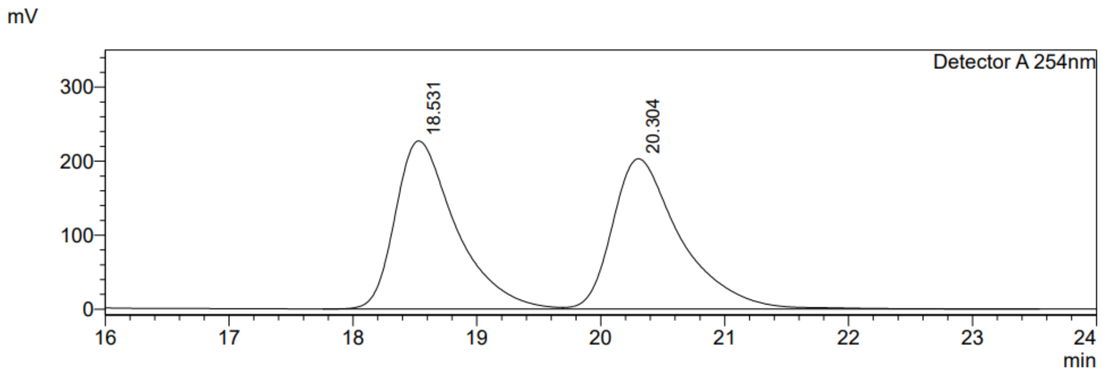

Detector A 254nm

Peak#	Ret. Time	Height	Height%	Area	Area%
1	18.531	227073	52.803	7723604	49.844
2	20.304	202963	47.197	7771821	50.156
Total		430037	100.000	15495425	100.000

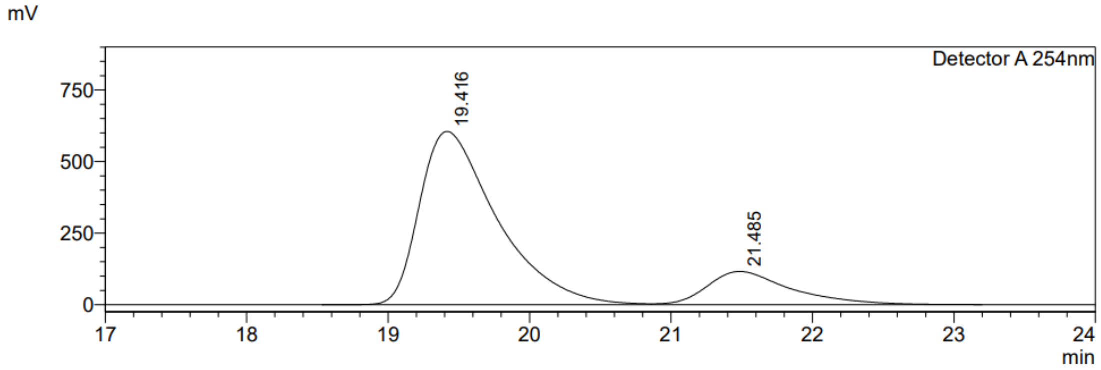

Detector A 254nm

Peak#	Ret. Time	Height	Height%	Area	Area%
1	19.416	604900	83.922	23058329	82.876
2	21.485	115889	16.078	4764448	17.124
Total		720789	100.000	27822777	100.000

(*R*)-2-(benzo[*b*]thiophen-3-yl)-4,5-diphenyl-3,6-dihydro-2*H*-1,2-thiazine 1-oxide (20)

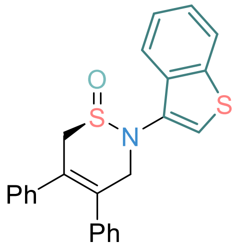

20

White solid; 40 mg, 99% yield; m.p. = 188.4-190.0 °C; ^{1}H NMR (400 MHz, $CDCl_3$) δ 7.86 (dd, J = 6.8, 1.8 Hz, 2H), 7.48-7.39 (m, 2H), 7.38 (s, 1H), 7.22-7.11 (m, 10H), 4.69 (ddd, J = 17.0, 4.3, 2.4 Hz, 1H), 4.23 (ddd, J = 16.7, 4.3, 2.7 Hz, 1H), 4.14 (dd, J = 17.0, 2.4 Hz, 1H), 3.79 (dd, J = 16.7, 2.1 Hz, 1H); ^{13}C NMR (100 MHz, $CDCl_3$) δ 140.53, 139.06, 139.02, 138.86, 134.95, 133.88, 129.33, 129.28, 128.30, 128.27, 127.43, 127.17, 125.36, 124.48, 123.99, 123.34, 121.72, 118.12, 55.39, 48.41; HRMS (ESI) m/z calcd for $C_{24}H_{19}NOS_2$ $[M+H]^+$ = 402.0986, found = 402.0988.

Optical Rotation: $[\alpha]^{25}_{D}$ = 105.58 (c = 0.52, $CHCl_3$). 96% e.e. (HPLC condition: Chiralpak IF column, n-Hexane/i-PrOH = 70:30, flow rate = 1.0 mL/min, wavelength = 254 nm, t_R = 9.95 min for minor isomer, t_R = 11.04 min for major isomer).

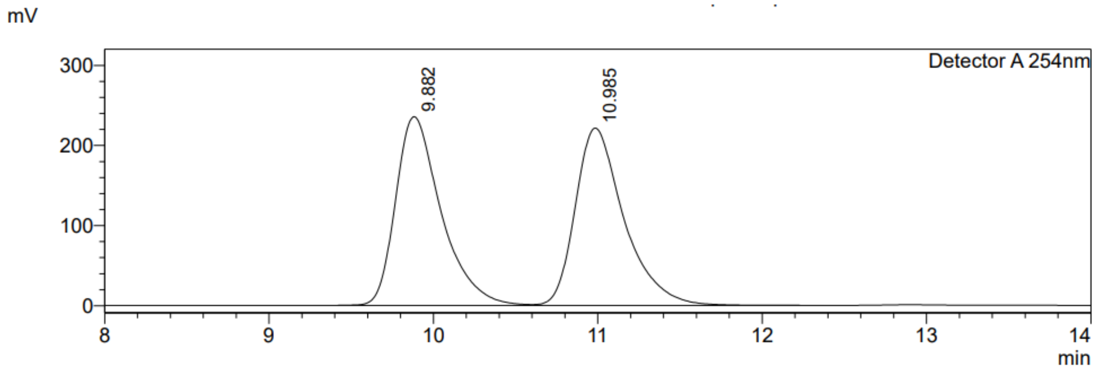

Detector A 254nm

Peak#	Ret. Time	Height	Height%	Area	Area%
1	9.882	235682	51.551	4548931	49.896
2	10.985	221504	48.449	4567979	50.104
Total		457185	100.000	9116910	100.000

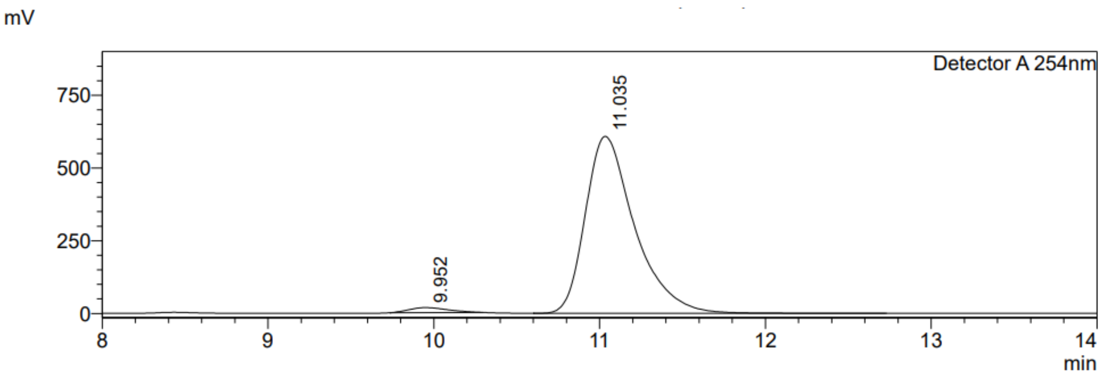

Detector A 254nm

Peak#	Ret. Time	Height	Height%	Area	Area%
1	9.952	17243	2.757	284723	2.210
2	11.035	608168	97.243	12598487	97.790
Total		625411	100.000	12883211	100.000

(*R*)-4,5-diphenyl-2-(quinolin-5-yl)-3,6-dihydro-2*H*-1,2-thiazine 1-oxide (21)

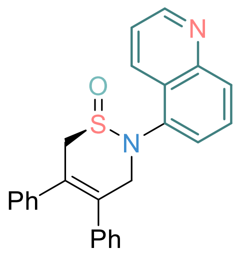

21

Light yellow solid; 38 mg, 95% yield; m.p. = 182.2-183.7 °C; ^{1}H NMR (400 MHz, $CDCl_3$) δ 8.98 (d, *J* = 2.8 Hz, 1H), 8.53 (d, *J* = 8.3 Hz, 1H), 8.10 (dd, *J* = 7.4, 1.6 Hz, 1H), 7.80-7.68 (m, 2H), 7.51 (dd, *J* = 8.5, 4.2 Hz, 1H), 7.23-7.04 (m, 10H), 4.75 (ddd, *J* = 17.1, 4.1, 2.5 Hz, 1H), 4.28 (ddd, *J* = 16.8, 4.1, 2.7 Hz, 1H), 4.06 (dd, *J* = 17.1, 2.2 Hz, 1H), 3.82 (dd, *J* = 16.8, 2.0 Hz, 1H); ^{13}C NMR (100 MHz, $CDCl_3$) δ 150.99, 149.28, 142.58, 140.50, 138.95, 134.16, 131.56, 129.44, 129.33, 129.29, 128.34, 128.28, 127.47, 127.24, 125.79, 124.26, 123.45, 121.71, 55.74, 49.44; HRMS (ESI) *m/z* calcd for $C_{25}H_{20}N_2OS$ $[M+H]^+$ = 397.1374, found = 397.1368.

Optical Rotation: $[\alpha]^{25}_{D}$ = 151.00 (*c* = 0.50, $CHCl_3$). 89% e.e. (HPLC condition: Chiralpak ODH column, *n*-Hexane/*i*-PrOH = 70:30, flow rate = 1.0 mL/min, wavelength = 254 nm, t_R = 10.41 min for minor isomer, t_R = 37.78 min for major isomer).

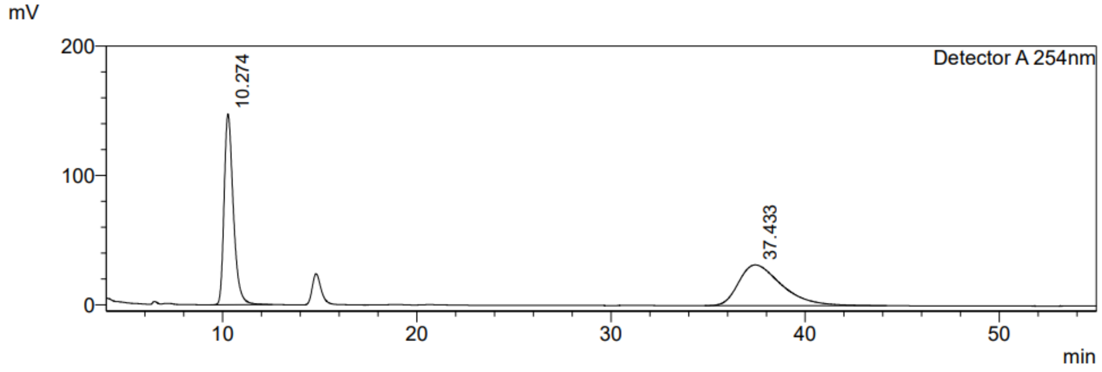

Detector A 254nm

Peak#	Ret. Time	Height	Height%	Area	Area%
1	10.274	147516	82.390	4859407	49.997
2	37.433	31531	17.610	4860065	50.003
Total		179046	100.000	9719472	100.000

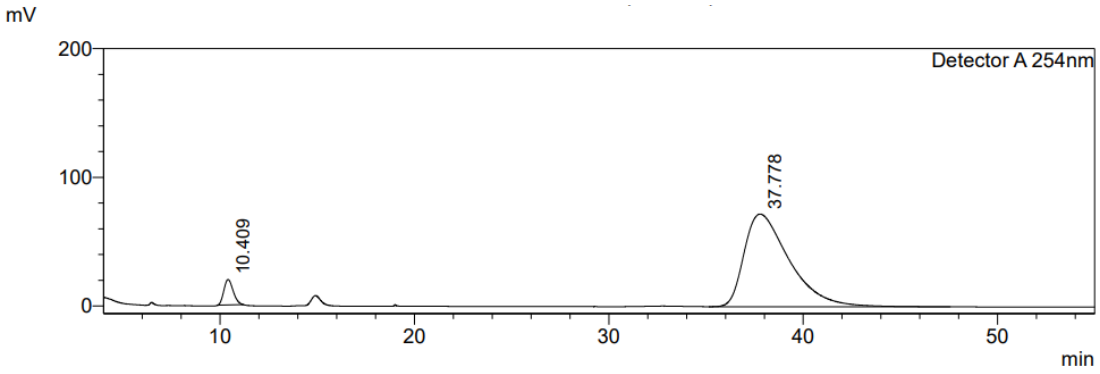

Detector A 254nm

Peak#	Ret. Time	Height	Height%	Area	Area%
1	10.409	19862	21.624	658400	5.348
2	37.778	71989	78.376	11652995	94.652
Total		91851	100.000	12311395	100.000

(*R*)-2-(isoquinolin-5-yl)-4,5-diphenyl-3,6-dihydro-2*H*-1,2-thiazine 1-oxide (22)

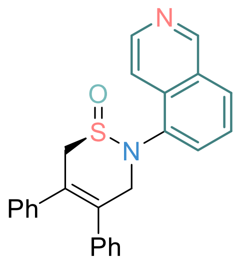

22

White solid; 23 mg, 58% yield; m.p. = 64.0-65.2 °C; ^{1}H NMR (400 MHz, $CDCl_3$) δ 9.31 (s, 1H), 8.65 (d, *J* = 5.0 Hz, 1H), 8.02-7.92 (m, 2H), 7.90 (d, *J* = 7.2 Hz, 1H), 7.63 (t, *J* = 7.8 Hz, 1H), 7.24-7.10 (m, 10H), 4.73 (ddd, *J* = 17.1, 4.3, 2.5 Hz, 1H), 4.30 (ddd, *J* = 16.8, 4.3, 2.7 Hz, 1H), 4.07 (dd, *J* = 17.1, 2.3 Hz, 1H), 3.82 (dd, *J* = 16.8, 2.1 Hz, 1H); ^{13}C NMR (100 MHz, $CDCl_3$) δ 152.97, 144.02, 141.94, 140.45, 138.93, 134.01,

133.25, 129.30, 128.35, 128.29, 127.50, 127.49, 127.25, 127.22, 126.90, 124.22, 115.79, 55.74, 49.09; HRMS (ESI) *m/z* calcd for $C_{25}H_{20}N_2OS$ $[M+H]^+$ = 397.1374, found = 397.1370.

Optical Rotation: $[\alpha]^{25}_{D}$ = 71.82 (*c* = 0.44, $CHCl_3$). 92% e.e. (HPLC condition: Chiralpak IG column, *n*-Hexane/*i*-PrOH = 70:30, flow rate = 1.0 mL/min, wavelength = 254 nm, t_R = 28.79 min for minor isomer, t_R = 43.42 min for major isomer).

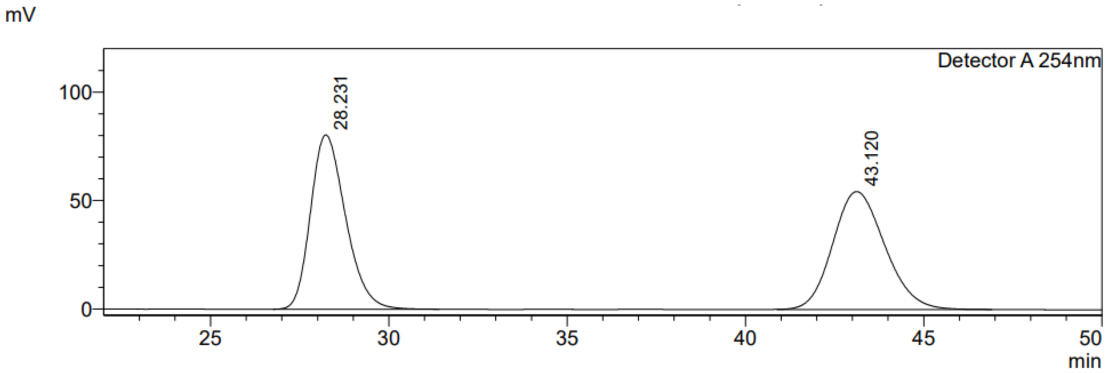

Detector A 254nm

Peak#	Ret. Time	Height	Height%	Area	Area%
1	28.231	80398	59.659	5494504	49.834
2	43.120	54364	40.341	5531206	50.166
Total		134761	100.000	11025710	100.000

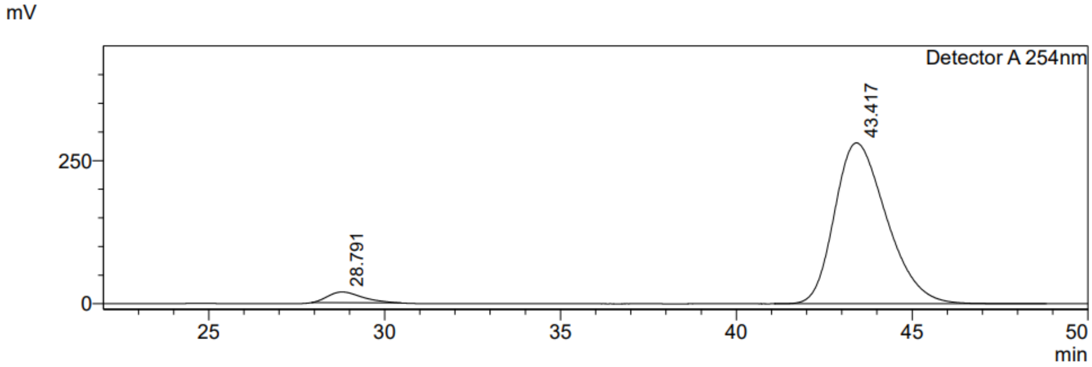

Detector A 254nm

Peak#	Ret. Time	Height	Height%	Area	Area%
1	28.791	18714	6.246	1274486	4.216
2	43.417	280918	93.754	28954246	95.784
Total		299631	100.000	30228732	100.000

<u>(*R*)-2-(phenanthren-9-yl)-4,5-diphenyl-3,6-dihydro-2*H*-1,2-thiazine 1-oxide (23)</u>

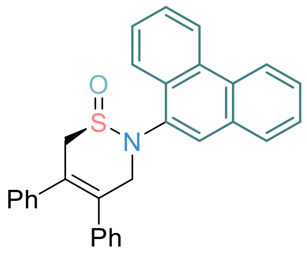

23

White solid; 34 mg, 76% yield; m.p. = 182.8-183.7 °C; ^{1}H NMR (400 MHz, $CDCl_3$) δ 8.81-8.73 (m, 1H), 8.67 (d, J = 8.2 Hz, 1H), 8.29 (d, J = 5.4 Hz, 1H), 8.05 (s, 1H), 7.91 (d, J = 7.4 Hz, 1H), 7.78-7.70 (m, 2H), 7.70-7.65 (m, 1H), 7.65-7.59 (m, 1H), 7.25-7.01 (m, 10H), 4.77 (ddd, J = 17.3, 4.2, 2.4 Hz, 1H), 4.33 (ddd, J = 16.8, 4.2, 2.7 Hz, 1H), 4.20 (dd, J = 17.3, 2.2 Hz, 1H), 3.84 (dd, J = 16.8, 2.1 Hz, 1H); ^{13}C NMR (100 MHz, $CDCl_3$) δ 141.29, 140.70, 139.12, 134.23, 131.74, 131.70, 129.76, 129.46, 129.37, 129.35, 129.01, 128.30, 128.23, 127.40, 127.37, 127.28, 127.25, 127.20, 127.15, 124.19, 123.59, 123.47, 123.41, 122.64, 55.95, 49.27; HRMS (ESI) *m/z* calcd for $C_{30}H_{23}NOS$ $[M+H]^+$ = 446.1578, found = 446.1572.

Optical Rotation: $[\alpha]^{25}_D$ = 121.13 (c = 0.53, $CHCl_3$). 95% e.e. (HPLC condition: Chiralpak IF column, *n*-Hexane/*i*-PrOH = 90:10, flow rate = 1.0 mL/min, wavelength = 254 nm, t_R = 26.65 min for major isomer, t_R = 29.56 min for minor isomer).

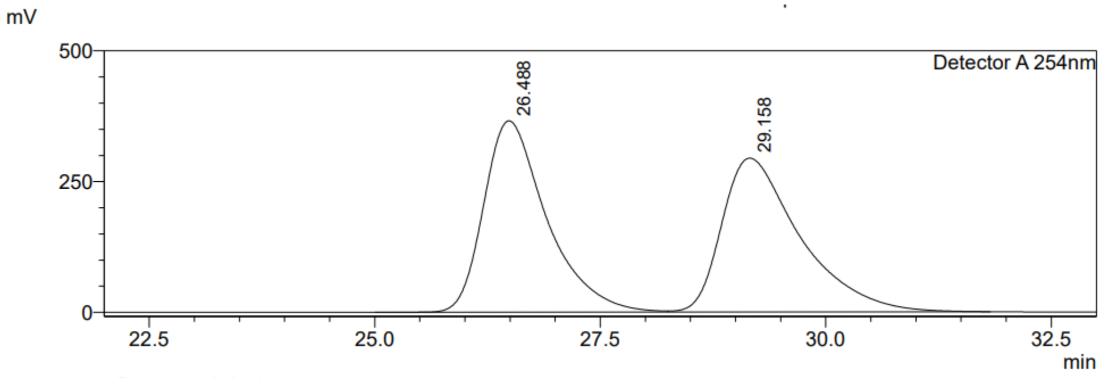

Detector A 254nm

Peak#	Ret. Time	Height	Height%	Area	Area%
1	26.488	365825	55.429	17792576	50.078
2	29.158	294164	44.571	17737371	49.922
Total		659989	100.000	35529947	100.000

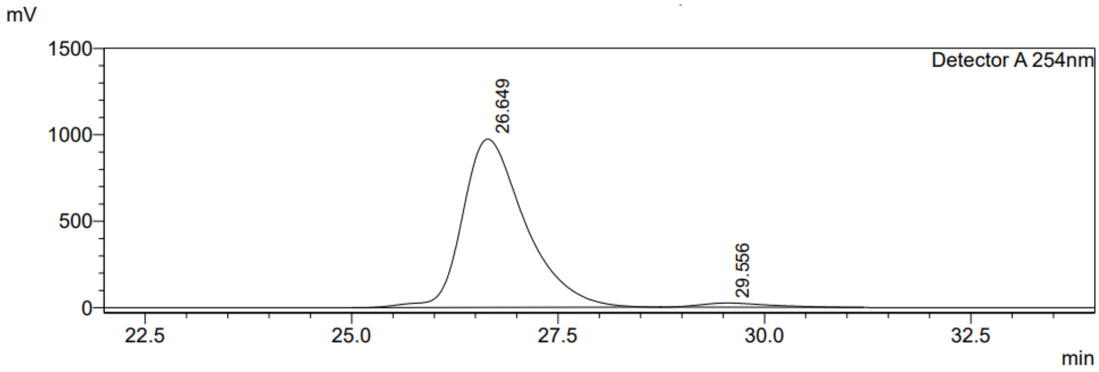

Detector A 254nm

Peak#	Ret. Time	Height	Height%	Area	Area%
1	26.649	973274	97.595	51554466	97.331
2	29.556	23982	2.405	1413906	2.669
Total		997256	100.000	52968371	100.000

(*R*)-4,5-diphenyl-2-(pyren-4-yl)-3,6-dihydro-2*H*-1,2-thiazine 1-oxide (24)

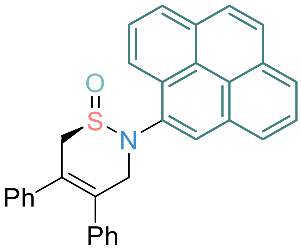

24

Light yellow solid; 35 mg, 75% yield; m.p. = 199.3-200.6 °C; ^{1}H NMR (400 MHz, $CDCl_3$) δ 8.44 (d, *J* = 9.0 Hz, 1H), 8.34-8.11 (m, 5H), 8.11-7.98 (m, 3H), 7.26-7.02 (m, 10H), 4.91 (ddd, *J* = 17.2, 3.7, 2.4 Hz, 1H), 4.40 (dd, *J* = 16.7, 3.7 Hz, 1H), 4.24 (dd, *J* = 17.2, 1.9 Hz, 1H), 3.89 (dd, *J* = 16.7, 1.8 Hz, 1H); ^{13}C NMR (100 MHz, $CDCl_3$) δ 140.80, 139.89, 139.16, 134.38, 131.32, 131.07, 130.63, 129.39, 128.50, 128.32, 128.23, 127.87, 127.37, 127.28, 127.16, 126.48, 125.75, 125.66, 125.62, 125.53, 124.77, 123.67, 122.04, 55.96, 49.86; HRMS (ESI) *m/z* calcd for $C_{32}H_{23}NOS$ $[M+H]^+$ = 470.1578, found = 470.1573.

Optical Rotation: $[\alpha]^{25}_{D}$ = 185.82 (*c* = 0.55, $CHCl_3$). 94% e.e. (HPLC condition: Chiralpak IF column, *n*-Hexane/*i*-PrOH = 70:30, flow rate = 1.0 mL/min, wavelength = 254 nm, t_R = 15.33 min for major isomer, t_R = 19.52 min for minor isomer).

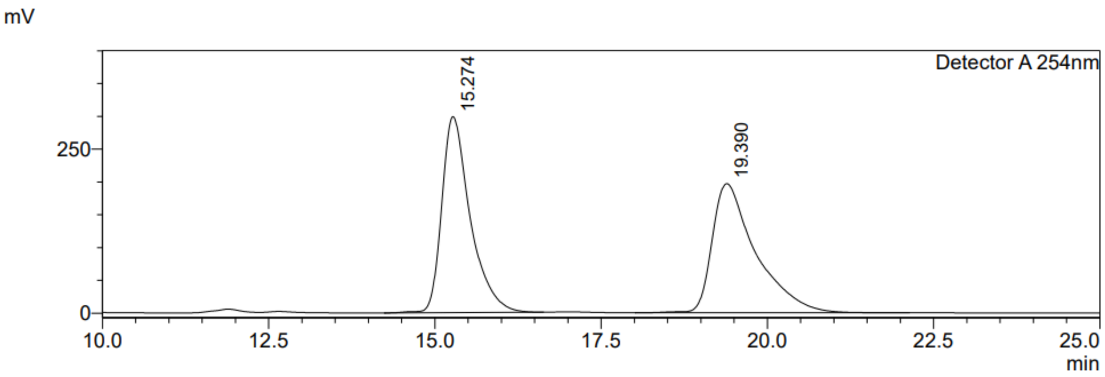

Detector A 254nm

Peak#	Ret. Time	Height	Height%	Area	Area%
1	15.274	298659	60.282	8907352	49.809
2	19.390	196776	39.718	8975827	50.191
Total		495435	100.000	17883179	100.000

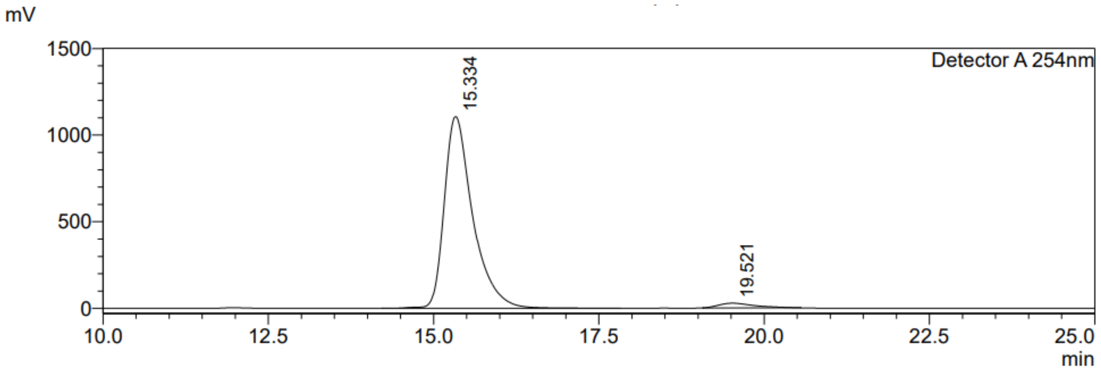

Detector A 254nm

Peak#	Ret. Time	Height	Height%	Area	Area%
1	15.334	1106327	97.604	33170640	96.985
2	19.521	27161	2.396	1031135	3.015
Total		1133489	100.000	34201775	100.000

(*R*)-2,4,5-triphenyl-3,6-dihydro-2*H*-1,2-thiazine 1-oxide (25)

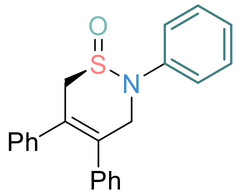

25

White solid; 28 mg, 81% yield; m.p. = 145.6-146.5 °C; ^{1}H NMR (400 MHz, $CDCl_3$) δ 7.43-7.36 (m, 2H), 7.29 (d, *J* = 7.7 Hz, 2H), 7.25-7.12 (m, 9H), 7.12-7.03 (m, 2H), 4.70-4.56 (m, 1H), 4.16-4.10 (m, 1H), 4.10-4.03 (m, 1H), 3.77 (dd, *J* = 16.1, 2.1 Hz, 1H); ^{13}C NMR (100 MHz, $CDCl_3$) δ 145.94, 140.60, 139.28, 133.60, 129.55, 129.35, 129.22, 128.27, 128.26, 127.41, 127.10, 125.33, 124.14, 121.97, 55.35, 46.83; HRMS (ESI) *m/z* calcd for $C_{22}H_{19}NOS$ $[M+H]^+$ = 346.1265, found = 346.1264.

Optical Rotation: $[\alpha]^{25}_{D}$ = 51.54 (c = 0.52, $CHCl_3$). 72% e.e. (HPLC condition: Chiralpak IF column, *n*-Hexane/*i*-PrOH = 70:30, flow rate = 1.0 mL/min, wavelength = 254 nm, t_R = 11.19 min for minor isomer, t_R = 13.14 min for major isomer).

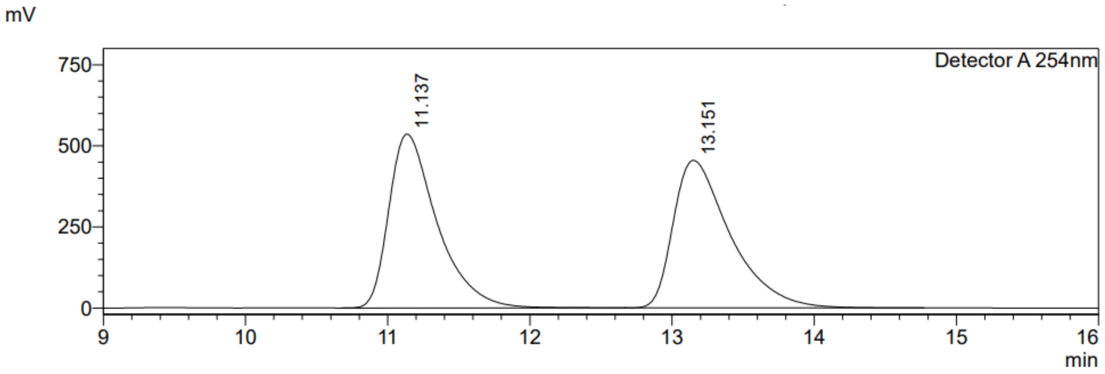

Detector A 254nm

Peak#	Ret. Time	Height	Height%	Area	Area%
1	11.137	536007	54.079	12657553	49.892
2	13.151	455141	45.921	12712320	50.108
Total		991149	100.000	25369873	100.000

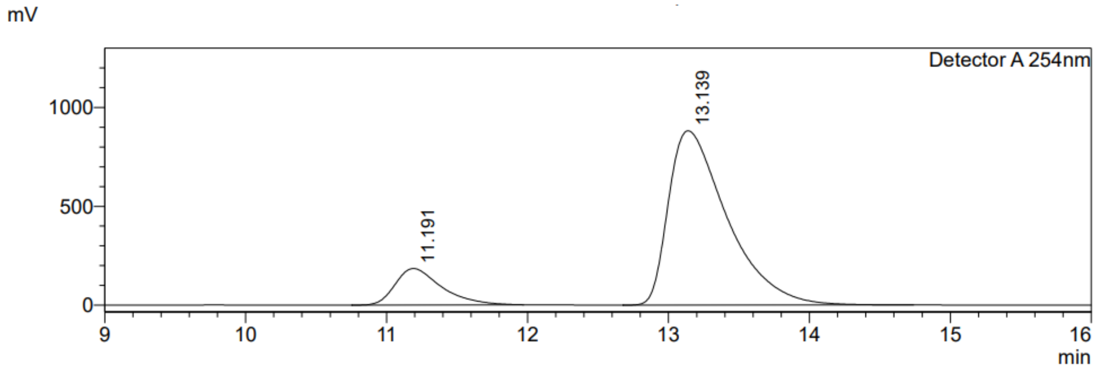

Detector A 254nm

Peak#	Ret. Time	Height	Height%	Area	Area%
1	11.191	184400	17.284	4214018	14.159
2	13.139	882465	82.716	25547052	85.841
Total		1066865	100.000	29761070	100.000

(*R*)-2-(2-methoxyphenyl)-4,5-diphenyl-3,6-dihydro-2*H*-1,2-thiazine 1-oxide (26)

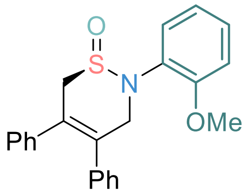

26

White solid; 37 mg, 98% yield; m.p. = 96.2-98.2 °C; ^{1}H NMR (400 MHz, $CDCl_3$) δ 7.40 (dd, J = 7.7, 1.5 Hz, 1H), 7.23 (td, J = 7.9, 1.6 Hz, 1H), 7.19-7.08 (m, 10H), 7.01-6.94 (m, 2H), 4.58 (ddd, J = 16.9, 4.1, 2.4 Hz, 1H), 4.15 (ddd, J = 16.3, 4.1, 2.9 Hz, 1H), 4.06 (dd, J = 16.9, 2.5 Hz, 1H), 3.89 (s, 3H), 3.69 (dd, J = 16.3, 2.1 Hz, 1H); ^{13}C

NMR (100 MHz, $CDCl_3$) δ 154.24, 140.99, 139.45, 134.73, 134.19, 129.37, 129.31, 128.17, 128.13, 127.57, 127.20, 126.92, 125.21, 124.15, 121.25, 112.03, 55.94, 55.42, 47.51; HRMS (ESI) *m/z* calcd for $C_{23}H_{21}NO_2S$ $[M+H]^+$ = 376.1371, found = 376.1379.

Optical Rotation: $[\alpha]^{25}_D$ = 192.08 (*c* = 0.53, $CHCl_3$). 94% e.e. (HPLC condition: Chiralpak IG column, *n*-Hexane/*i*-PrOH =70:30, flow rate = 1.0 mL/min, wavelength = 254 nm, t_R = 19.49 min for major isomer, t_R = 22.51 min for minor isomer).

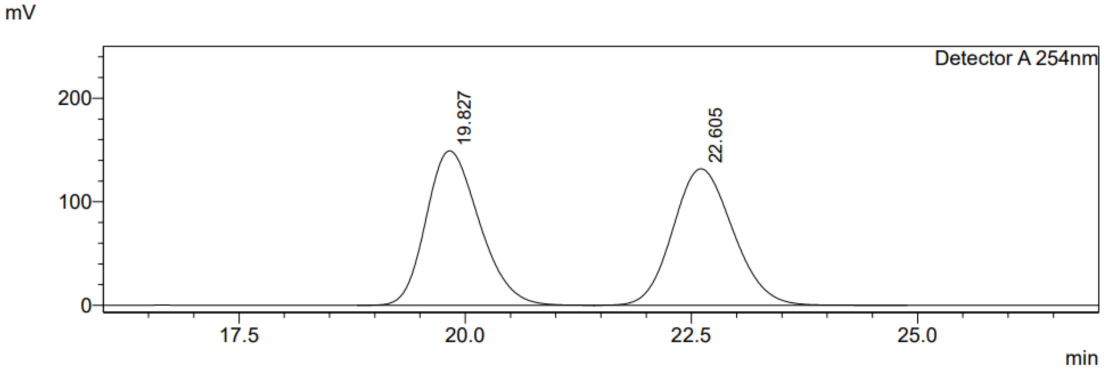

Detector A 254nm

Peak#	Ret. Time	Height	Height%	Area	Area%
1	19.827	149120	53.071	6110534	50.001
2	22.605	131860	46.929	6110346	49.999
Total		280980	100.000	12220880	100.000

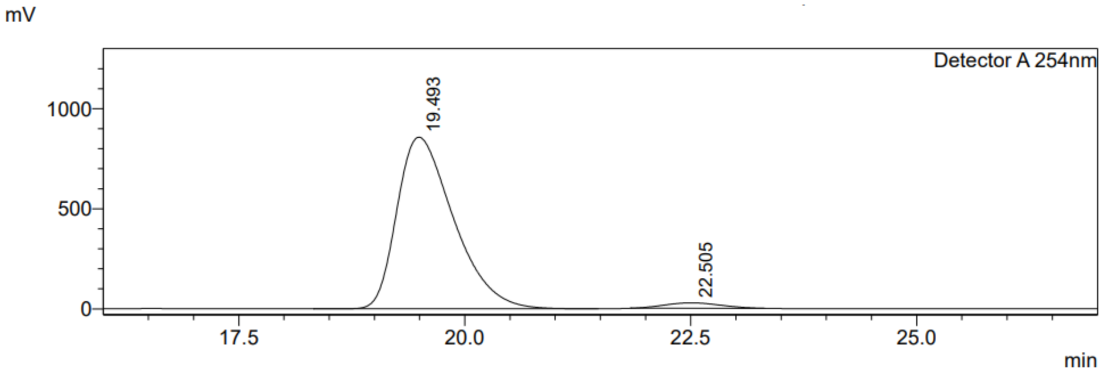

Detector A 254nm

Peak#	Ret. Time	Height	Height%	Area	Area%
1	19.493	857258	96.920	37405761	96.924
2	22.505	27242	3.080	1187008	3.076
Total		884500	100.000	38592769	100.000

(*R*)-2-(2-ethylphenyl)-4,5-diphenyl-3,6-dihydro-2*H*-1,2-thiazine 1-oxide (27)

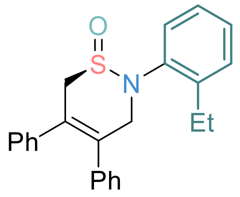

27

White solid; 37 mg, 98% yield; m.p. = 107.5-108.2 °C; ^{1}H NMR (400 MHz, $CDCl_3$) δ 7.57-7.46 (m, 1H), 7.32-7.24 (m, 3H), 7.23-6.98 (m, 10H), 4.61 (ddd, J = 17.3, 4.4, 2.6 Hz, 1H), 4.09 (ddd, J = 16.6, 4.4, 2.8 Hz, 1H), 3.89 (dd, J = 17.3, 2.4 Hz, 1H), 3.72 (dd, J = 16.6, 2.1 Hz, 1H), 2.85-2.65 (m, 2H), 1.28 (t, J = 7.5 Hz, 3H); ^{13}C NMR (100 MHz, $CDCl_3$) δ 144.35, 141.59, 140.77, 139.21, 134.16, 129.58, 129.31, 128.22, 128.18, 128.02, 127.35, 127.29, 127.04, 126.76, 124.12, 55.55, 49.31, 24.56, 15.23; HRMS (ESI) m/z calcd for $C_{24}H_{23}NOS$ $[M+H]^+$ = 374.1578, found = 374.1573.

Optical Rotation: $[\alpha]^{25}_D$ = 189.17 (c = 0.48, $CHCl_3$). 96% e.e. (HPLC condition: Chiralpak IF column, n-Hexane/i-PrOH 90:10, flow rate = 1.0 mL/min, wavelength = 254 nm, t_R = 18.93 min for major isomer, t_R = 20.39 min for minor isomer).

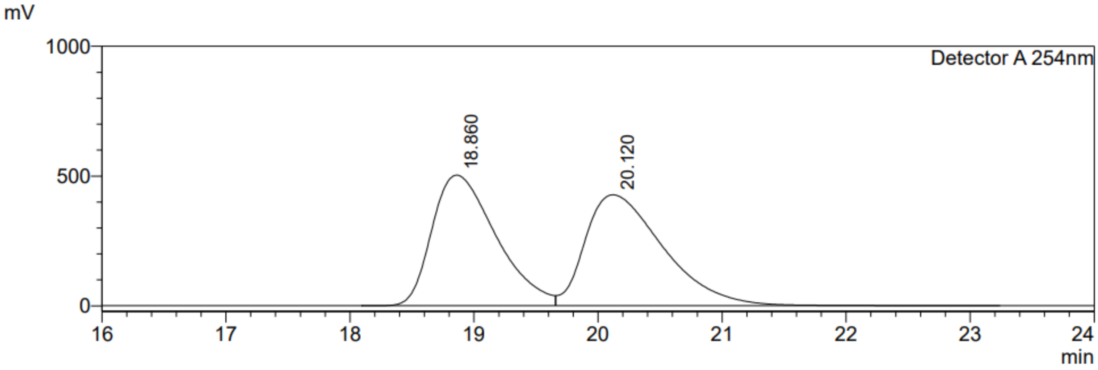

Detector A 254nm

Peak#	Ret. Time	Height	Height%	Area	Area%
1	18.860	503265	54.080	18122368	49.128
2	20.120	427327	45.920	18765782	50.872
Total		930593	100.000	36888150	100.000

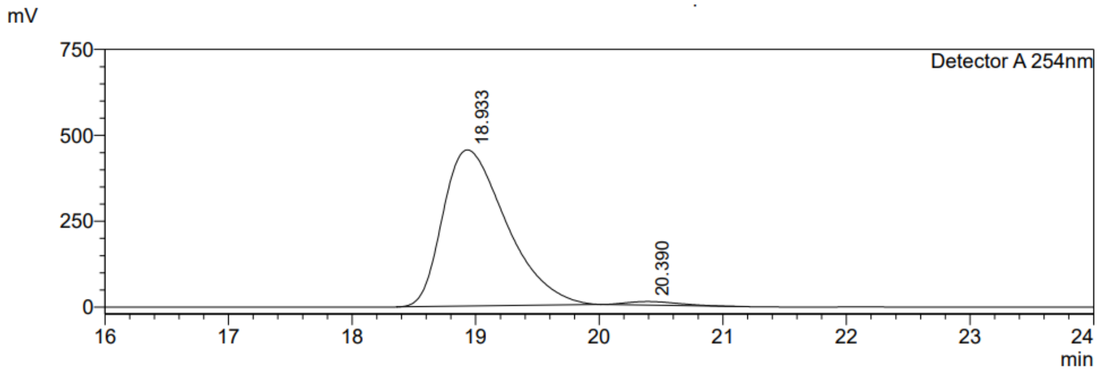

Detector A 254nm

Peak#	Ret. Time	Height	Height%	Area	Area%
1	18.933	454498	97.731	16281777	98.089
2	20.390	10550	2.269	317189	1.911
Total		465048	100.000	16598966	100.000

(*R*)-4,5-diphenyl-2-(o-tolyl)-3,6-dihydro-2*H*-1,2-thiazine 1-oxide (28)

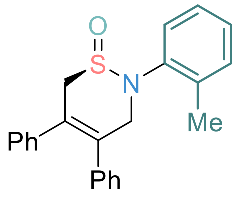

28

White solid; 35 mg, 97% yield; m.p. = 117.4-119.2 °C; ^{1}H NMR (400 MHz, $CDCl_3$) δ 7.55-7.44 (m, 1H), 7.30-7.20 (m, 3H), 7.20-7.07 (m, 10H), 4.61 (ddd, J = 17.2, 4.4, 2.5 Hz, 1H), 4.10 (ddd, J = 16.6, 4.4, 2.8 Hz, 1H), 3.89 (dd, J = 17.2, 2.4 Hz, 1H), 3.71 (dd, J = 16.6, 2.1 Hz, 1H), 2.39 (s, 3H); ^{13}C NMR (100 MHz, $CDCl_3$) δ 144.73, 140.76, 139.24, 135.24, 134.12, 131.29, 129.31, 129.29, 128.22, 128.19, 127.47, 127.36, 127.30, 127.04, 125.85, 124.12, 55.49, 48.45, 18.27; HRMS (ESI) m/z calcd for $C_{23}H_{21}NOS$ $[M+H]^+$ = 360.1422, found = 360.1430.

Optical Rotation: $[\alpha]^{25}_{D}$ = 134.29 (c = 0.49, $CHCl_3$). 93% e.e. (HPLC condition: Chiralpak IF column, n-Hexane/i-PrOH = 90:10, flow rate = 1.0 mL/min, wavelength = 254 nm, t_R = 18.92 min for major isomer, t_R = 21.06 min for minor isomer).

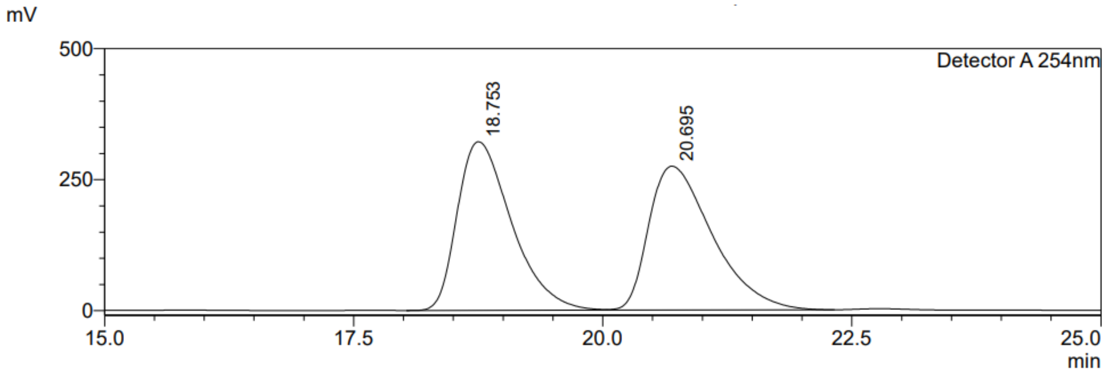

Detector A 254nm

Peak#	Ret. Time	Height	Height%	Area	Area%
1	18.753	321931	53.968	12208823	49.981
2	20.695	274594	46.032	12218210	50.019
Total		596525	100.000	24427033	100.000

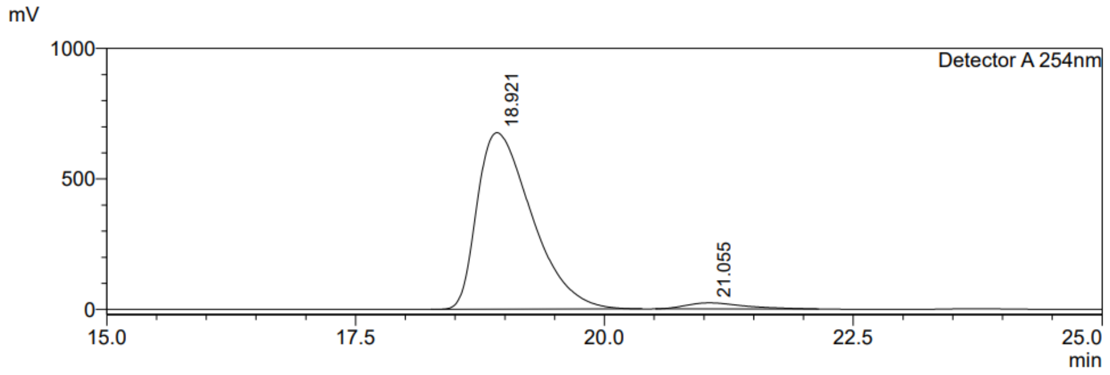

Detector A 254nm

Peak#	Ret. Time	Height	Height%	Area	Area%
1	18.921	676330	96.695	25942468	96.500
2	21.055	23113	3.305	940908	3.500
Total		699444	100.000	26883376	100.000

(*R*)-2-(2-bromophenyl)-4,5-diphenyl-3,6-dihydro-2*H*-1,2-thiazine 1-oxide (29)

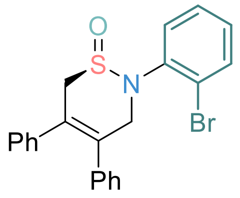

29

White solid; 30 mg, 71% yield; m.p. = 44.8-45.6 °C; ^{1}H NMR (400 MHz, $CDCl_3$) δ 7.67 (dd, *J* = 8.0, 1.3 Hz, 1H), 7.55 (dd, *J* = 7.9, 1.3 Hz, 1H), 7.36 (td, *J* = 7.8, 1.3 Hz, 1H), 7.19-7.09 (m, 11H), 4.54 (ddd, *J* = 16.9, 4.3, 2.4 Hz, 1H), 4.23 (ddd, *J* = 16.5, 4.3, 2.8 Hz, 1H), 4.03 (dd, *J* = 16.9, 2.4 Hz, 1H), 3.72 (dd, *J* = 16.5, 2.0 Hz, 1H); ^{13}C NMR (100 MHz, $CDCl_3$) δ 144.40, 140.72, 139.13, 133.81, 133.63, 129.41, 129.33, 128.91, 128.59, 128.22, 128.20, 127.35, 127.06, 126.81, 124.12, 122.41, 55.19, 47.84; HRMS (ESI) *m/z* calcd for $C_{22}H_{18}BrNOS$ $[M+H]^+$ = 424.0370, found = 424.0367.

Optical Rotation: $[\alpha]^{25}_D$ = 74.52 (*c* = 0.42, $CHCl_3$). 98% e.e. (HPLC condition: Chiralpak ODH column, *n*-Hexane/*i*-PrOH = 80:20, flow rate = 1.0 mL/min, wavelength = 254 nm, t_R = 7.80 min for minor isomer, t_R = 15.84 min for major isomer).

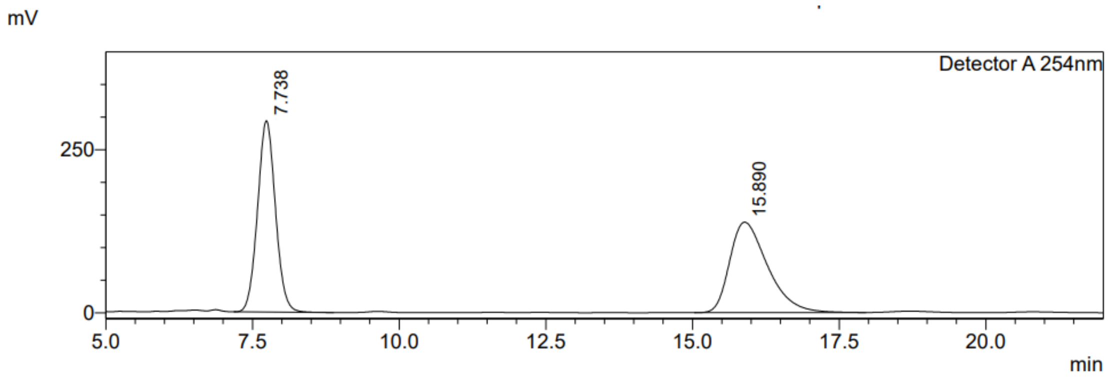

Detector A 254nm

Peak#	Ret. Time	Height	Height%	Area	Area%
1	7.738	293396	67.897	6230033	49.967
2	15.890	138722	32.103	6238307	50.033
Total		432118	100.000	12468341	100.000

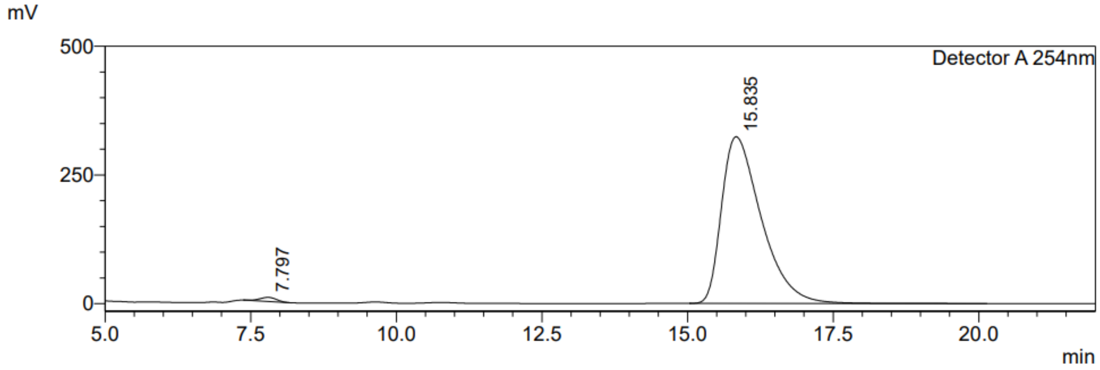

Detector A 254nm

Peak#	Ret. Time	Height	Height%	Area	Area%
1	7.797	8040	2.422	160778	1.032
2	15.835	323950	97.578	15422219	98.968
Total		331990	100.000	15582997	100.000

(*R*)-2-(2-chlorophenyl)-4,5-diphenyl-3,6-dihydro-2*H*-1,2-thiazine 1-oxide (30)

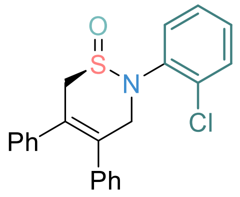

30

White oil; 31 mg, 82% yield; ^{1}H NMR (400 MHz, $CDCl_3$) δ 7.53 (dd, J = 7.9, 1.4 Hz, 1H), 7.48 (dd, J = 7.9, 1.4 Hz, 1H), 7.31 (td, J = 7.7, 1.5 Hz, 1H), 7.23 (td, J = 7.7, 1.5 Hz, 1H), 7.21-7.06 (m, 10H), 4.56 (ddd, J = 16.9, 4.3, 2.4 Hz, 1H), 4.19 (ddd, J = 16.5, 4.3, 2.8 Hz, 1H), 4.03 (dd, J = 16.9, 2.5 Hz, 1H), 3.72 (dd, J = 16.5, 2.0 Hz, 1H); ^{13}C NMR (100 MHz, $CDCl_3$) δ 143.04, 140.70, 139.13, 133.65, 131.54, 130.67, 129.39, 129.31, 128.23, 128.21, 128.17, 128.08, 127.35, 127.06, 126.42, 124.11, 55.23, 47.61; HRMS (ESI) *m/z* calcd for $C_{22}H_{18}ClNOS$ $[M+H]^+$ = 380.0876, found = 380.0876

Optical Rotation: $[\alpha]^{25}_{D}$ = 17.19 (c = 0.32, $CHCl_3$). 93% e.e. (HPLC condition: Chiralpak IC column, n-Hexane/i-PrOH = 70:30, flow rate = 1.0 mL/min, wavelength = 254 nm, t_R = 12.62 min for minor isomer, t_R = 16.83 min for major isomer).

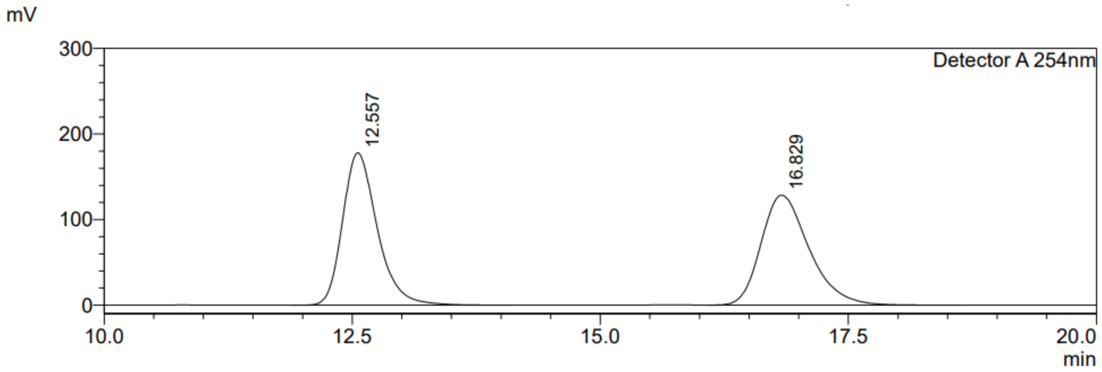

Detector A 254nm

Peak#	Ret. Time	Height	Height%	Area	Area%
1	12.557	177990	58.082	4333520	50.012
2	16.829	128455	41.918	4331467	49.988
Total		306445	100.000	8664987	100.000

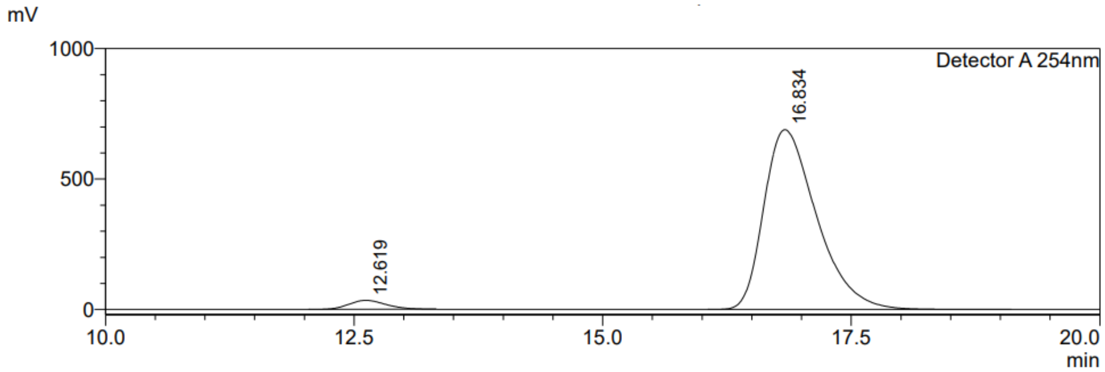

Detector A 254nm

Peak#	Ret. Time	Height	Height%	Area	Area%
1	12.619	34592	4.780	910244	3.451
2	16.834	689027	95.220	25466155	96.549
Total		723619	100.000	26376399	100.000

(*R*)-2-(2-fluorophenyl)-4,5-diphenyl-3,6-dihydro-2*H*-1,2-thiazine 1-oxide (31)

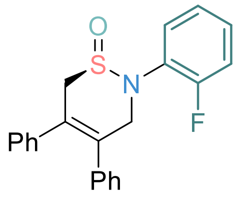

31

White solid; 29 mg, 81% yield; m.p. = 107.4-105.9 ºC; ^{1}H NMR (400 MHz, $CDCl_3$) δ 7.50-7.40 (m, 1H), 7.25 – 7.20 (m, 1H), 7.20-7.03 (m, 12H), 4.62 (ddd, J = 16.8, 4.01 2.4 Hz, 1H), 4.14 (ddd, J = 16.5, 4.1, 2.9 Hz, 1H), 4.05 (dd, J = 16.8, 2.5 Hz, 1H), 3.72 (dd, J = 16.5, 2.1 Hz, 1H); ^{13}C NMR (100 MHz, $CDCl_3$) δ 157.22 (d, J = 248.6 Hz),

140.66, 139.13, 133.89 (d, J = 10.6 Hz), 133.66, 129.35, 129.26, 128.25, 128.23, 127.76 (d, J = 7.9 Hz), 127.38, 127.08, 125.61, 124.96 (d, J = 3.9 Hz), 124.10, 116.74 (d, J = 20.3 Hz), 55.49, 47.53; ^{19}F NMR (376 MHz, $CDCl_3$) δ -122.44; HRMS (ESI) *m/z* calcd for $C_{22}H_{18}FNOS$ $[M+H]^+$ = 364.1171, found = 364.1169.

Optical Rotation: $[\alpha]^{25}_D$ = 109.25 (c = 0.53, $CHCl_3$). 80% e.e. (HPLC condition: Chiralpak IE column, *n*-Hexane/*i*-PrOH = 70:30, flow rate = 1.0 mL/min, wavelength = 254 nm, t_R = 11.46 min for minor isomer, t_R = 12.29 min for major isomer).

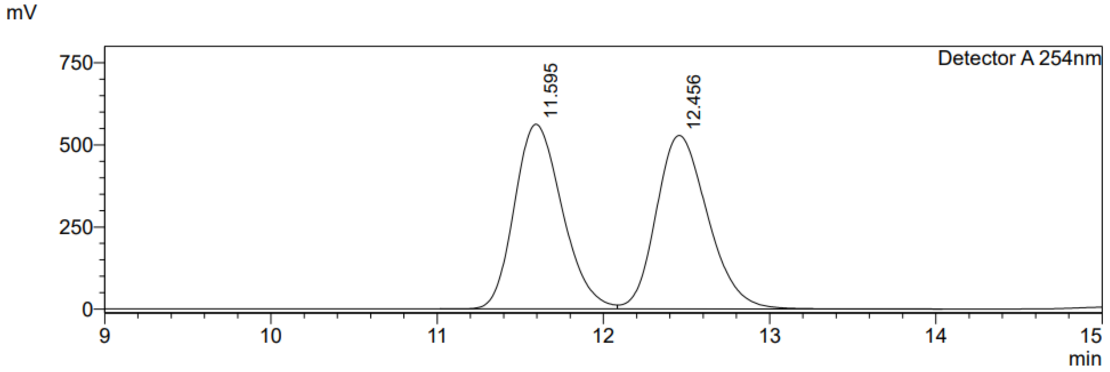

Detector A 254nm

Peak#	Ret. Time	Height	Height%	Area	Area%
1	11.595	562560	51.565	11245013	49.686
2	12.456	528403	48.435	11387341	50.314
Total		1090963	100.000	22632353	100.000

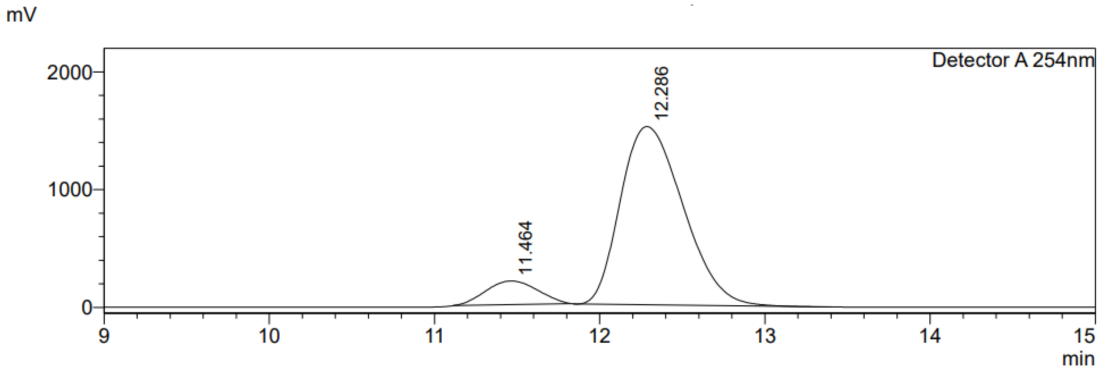

Detector A 254nm

Peak#	Ret. Time	Height	Height%	Area	Area%
1	11.464	200830	11.712	4385080	9.877
2	12.286	1513953	88.288	40009886	90.123
Total		1714783	100.000	44394966	100.000

(*R*)-2-(2,5-dimethoxyphenyl)-4,5-diphenyl-3,6-dihydro-2*H*-1,2-thiazine 1-oxide (32)

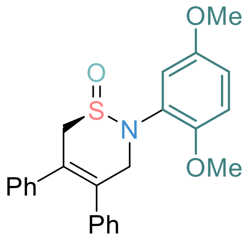

32

White solid; 39 mg, 95% yield; m.p. = 44.6-45.5 ºC; ^{1}H NMR (400 MHz, $CDCl_3$) δ 7.18-7.08 (m, 10H), 6.96 (d, J = 3.0 Hz, 1H), 6.88 (d, J = 8.9 Hz, 1H), 6.75 (dd, J = 8.9, 3.0 Hz, 1H), 4.57 (ddd, J = 16.8, 4.1, 2.4 Hz, 1H), 4.14 (ddd, J = 16.3, 4.1, 2.9 Hz, 1H), 4.06 (dd, J = 16.8, 2.5 Hz, 1H), 3.84 (s, 3H), 3.77 (s, 3H), 3.69 (dd, J = 16.4, 2.1 Hz, 1H); ^{13}C NMR (100 MHz, $CDCl_3$) δ 154.08, 148.34, 140.94, 139.41, 135.37, 134.11, 129.39, 129.30, 128.18, 128.15, 127.23, 126.94, 124.23, 113.11, 111.86, 111.31, 56.59, 55.95, 55.37, 47.29; HRMS (ESI) *m/z* calcd for $C_{24}H_{23}NO3S$ $[M+H]^+$ = 406.1477, found = 406.1472.

Optical Rotation: $[\alpha]^{25}_{D}$ = 93.67 (c = 0.49, $CHCl_3$). 95% e.e. (HPLC condition: Chiralpak ODH column, *n*-Hexane/*i*-PrOH = 70:30, flow rate = 1.0 mL/min, wavelength = 254 nm, t_R = 7.76 min for minor isomer, t_R = 13.28 min for major isomer).

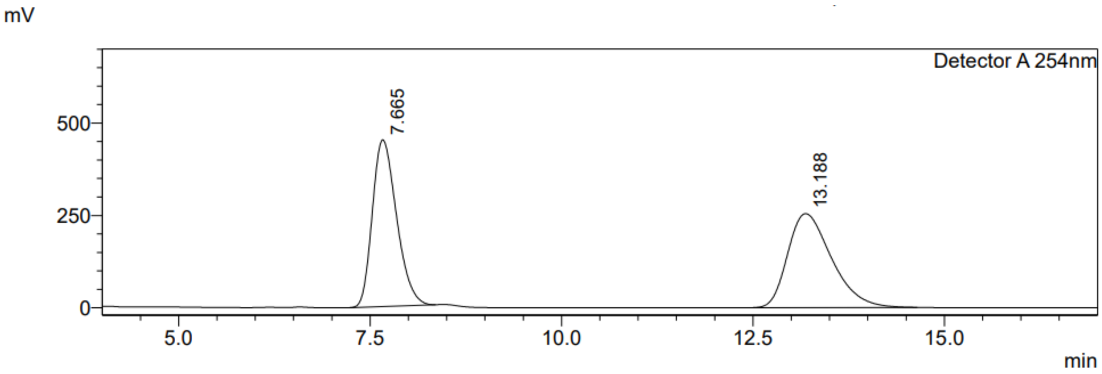

Detector A 254nm

Peak#	Ret. Time	Height	Height%	Area	Area%
1	7.665	451115	63.928	9942357	49.484
2	13.188	254546	36.072	10149583	50.516
Total		705661	100.000	20091940	100.000

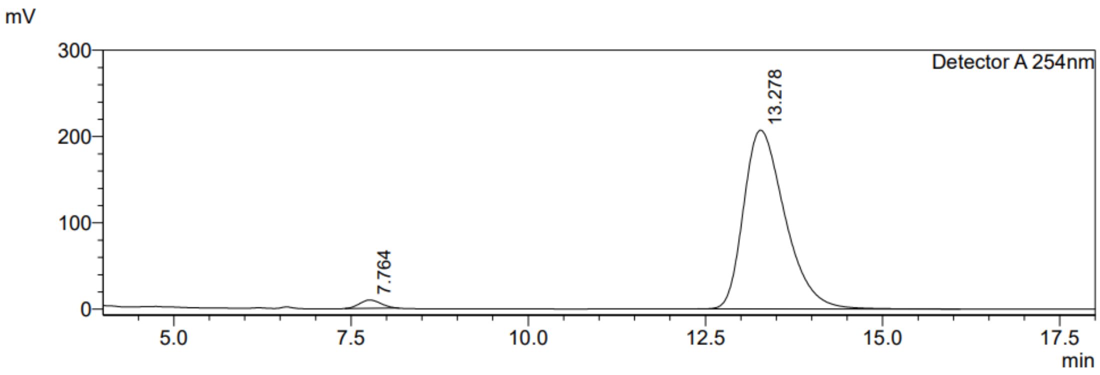

Detector A 254nm

Peak#	Ret. Time	Height	Height%	Area	Area%
1	7.764	9598	4.429	200988	2.331
2	13.278	207131	95.571	8421156	97.669
Total		216729	100.000	8622144	100.000

(*R*)-2-(2,5-dimethylphenyl)-4,5-diphenyl-3,6-dihydro-2*H*-1,2-thiazine 1-oxide (33)

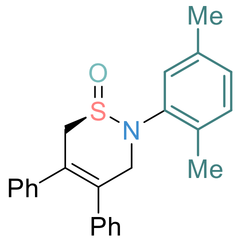

33

White solid; 36 mg, 97% yield; m.p. = 133.7-135.0 °C; ^{1}H NMR (400 MHz, $CDCl_3$) δ 7.29 (s, 1H), 7.21-7.08 (m, 11H), 7.02 (d, *J* = 7.6 Hz, 1H), 4.60 (ddd, *J* = 17.2, 4.4, 2.6 Hz, 1H), 4.09 (ddd, *J* = 16.6, 4.4, 2.8 Hz, 1H), 3.88 (dd, *J* = 17.2, 2.4 Hz, 1H), 3.71 (dd, *J* = 16.6, 2.1 Hz, 1H), 2.34 (s, 3H), 2.32 (s, 3H); ^{13}C NMR (100 MHz, $CDCl_3$) δ 144.49, 140.79, 139.28, 137.16, 134.12, 131.85, 131.04, 129.32, 129.30, 128.21, 128.17, 127.27, 127.01, 126.35, 124.08, 55.51, 48.40, 21.02, 17.82; HRMS (ESI) *m/z* calcd for $C_{24}H_{23}NOS$ $[M+H]^+$ = 374.1578, found = 374.1574.

Optical Rotation: $[\alpha]^{25}_D$ = 98.75 (*c* = 0.56, $CHCl_3$). 95% e.e. (HPLC condition: Chiralpak IG column, *n*-Hexane/*i*-PrOH = 90:10, flow rate = 1.0 mL/min, wavelength = 254 nm, t_R = 22.58 min for minor isomer, t_R = 24.15 min for major isomer).

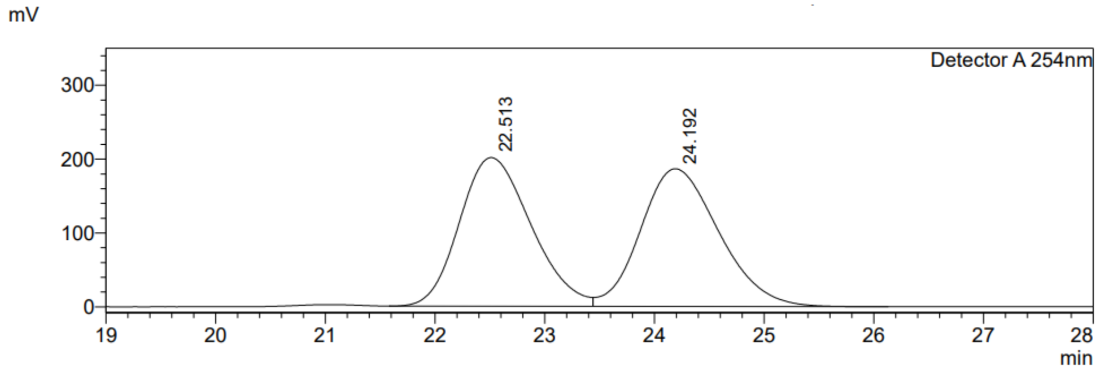

Detector A 254nm

Peak#	Ret. Time	Height	Height%	Area	Area%
1	22.513	201298	51.924	9136498	49.681
2	24.192	186381	48.076	9253883	50.319
Total		387679	100.000	18390381	100.000

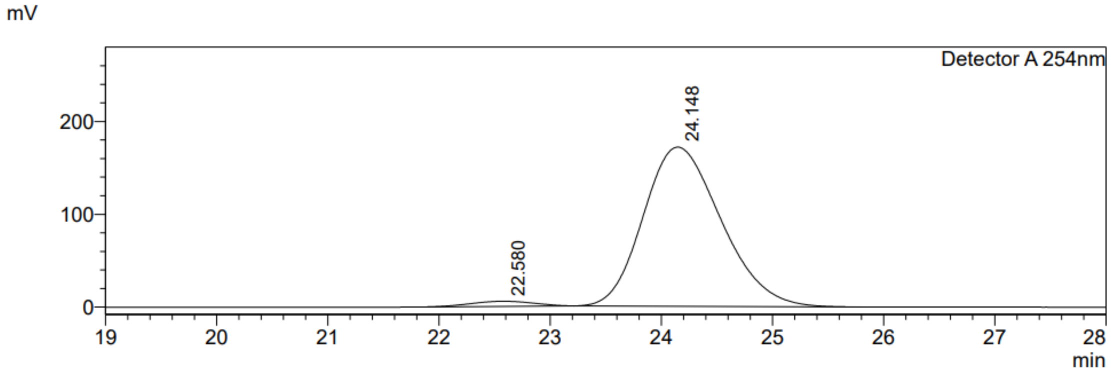

Detector A 254nm

Peak#	Ret. Time	Height	Height%	Area	Area%
1	22.580	5524	3.121	213821	2.497
2	24.148	171458	96.879	8350290	97.503
Total		176982	100.000	8564111	100.000

(*R*)-2-(5-chloro-2-methylphenyl)-4,5-diphenyl-3,6-dihydro-2*H*-1,2-thiazine 1-oxide (34)

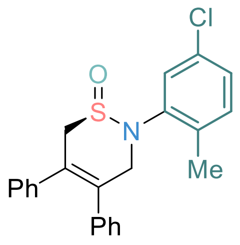

34

White solid; 24 mg, 61% yield; m.p. = 41.8-43.6 °C; ^{1}H NMR (400 MHz, $CDCl_3$) δ 7.44 (d, *J* = 1.4 Hz, 1H), 7.25-7.03 (m, 12H), 4.59 (ddd, *J* = 17.0, 4.3, 2.5 Hz, 1H), 4.10 (ddd, *J* = 16.7, 4.3, 2.7 Hz, 1H), 3.86 (dd, *J* = 17.0, 2.4 Hz, 1H), 3.72 (dd, *J* = 16.7, 2.2 Hz, 1H), 2.35 (s, 3H); ^{13}C NMR (100 MHz, $CDCl_3$) δ 145.56, 140.55, 139.06, 133.90,

133.57, 132.40, 132.21, 129.30, 129.26, 128.27, 127.45, 127.43, 127.15, 125.75, 124.07, 55.39, 48.06, 17.94; HRMS (ESI) *m/z* calcd for $C_{23}H_{20}ClNOS$ $[M+H]^+$ = 394.1032, found = 394.1027.

Optical Rotation: $[\alpha]^{25}_{D}$ = 48.78 (*c* = 0.49, $CHCl_3$). 81% e.e. (HPLC condition: Chiralpak IE column, *n*-Hexane/*i*-PrOH = 70:30, flow rate = 1.0 mL/min, wavelength = 254 nm, t_R = 21.57 min for minor isomer, t_R = 23.80 min for major isomer).

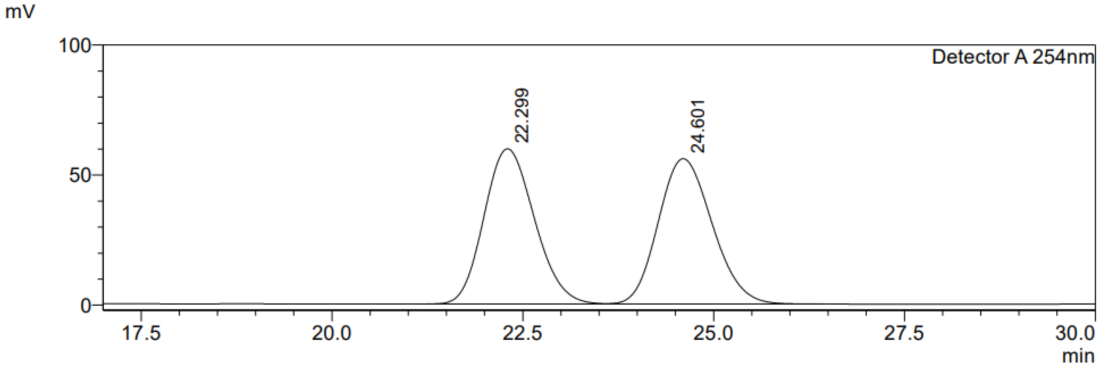

Detector A 254nm

Peak#	Ret. Time	Height	Height%	Area	Area%
1	22.299	59666	51.639	2743365	49.931
2	24.601	55879	48.361	2750969	50.069
Total		115545	100.000	5494334	100.000

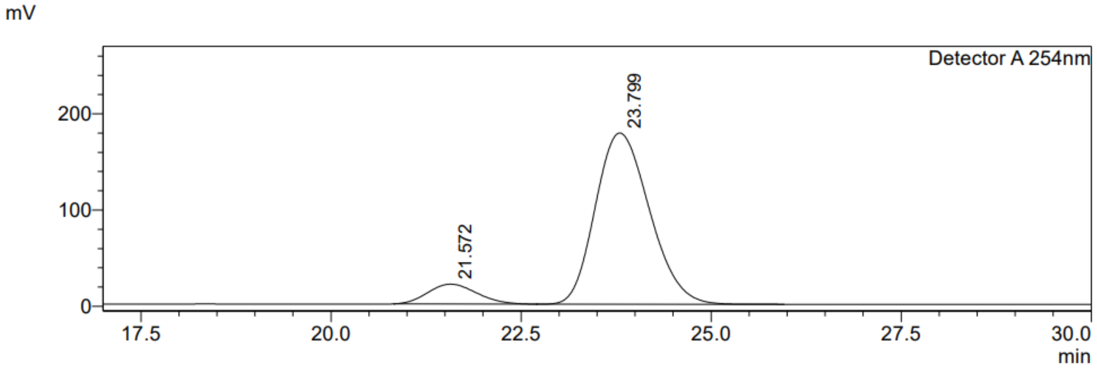

Detector A 254nm

Peak#	Ret. Time	Height	Height%	Area	Area%
1	21.572	20658	10.401	925573	9.502
2	23.799	177952	89.599	8815460	90.498
Total		198611	100.000	9741033	100.000

(*R*)-2-(2,4-dimethylphenyl)-4,5-diphenyl-3,6-dihydro-2*H*-1,2-thiazine 1-oxide (35)

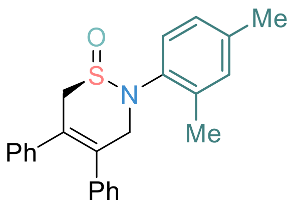

35

White solid; 35 mg, 94% yield; m.p. = 88.3-89.9 ºC; ^{1}H NMR (400 MHz, $CDCl_3$) δ 7.37 (d, *J* = 8.0 Hz, 1H), 7.19-7.09 (m, 11H), 7.05 (d, *J* = 8.0 Hz, 1H), 4.57 (ddd, *J* = 17.2, 4.4, 2.5 Hz, 1H), 4.08 (ddd, *J* = 16.6, 4.4, 2.8 Hz, 1H), 3.87 (dd, *J* = 17.2, 2.4 Hz, 1H), 3.70 (dd, *J* = 16.6, 2.0 Hz, 1H), 2.35 (s, 3H), 2.33 (s, 3H); ^{13}C NMR (100 MHz, $CDCl_3$) δ 142.20, 140.84, 139.28, 137.30, 135.03, 134.18, 131.94, 129.32, 129.30, 128.21, 128.17, 127.91, 127.27, 127.01, 125.83, 124.11, 55.57, 48.69, 21.09, 18.16; HRMS (ESI) *m/z* calcd for $C_{24}H_{23}NOS$ $[M+H]^+$ = 374.1578, found = 374.1573.

Optical Rotation: $[\alpha]^{25}_D$ = 100.38 (*c* = 0.52, $CHCl_3$). 95% e.e. (HPLC condition: Chiralpak IF column, *n*-Hexane/*i*-PrOH = 70:30, flow rate = 1.0 mL/min, wavelength = 254 nm, t_R = 9.60 min for major isomer, t_R = 12.04 min for minor isomer).

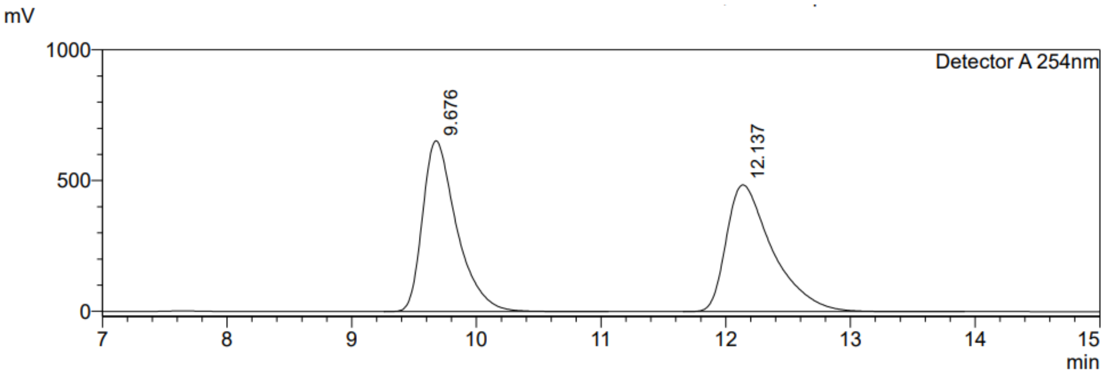

Detector A 254nm

Peak#	Ret. Time	Height	Height%	Area	Area%
1	9.676	653415	57.435	12414406	49.914
2	12.137	484250	42.565	12457351	50.086
Total		1137664	100.000	24871756	100.000

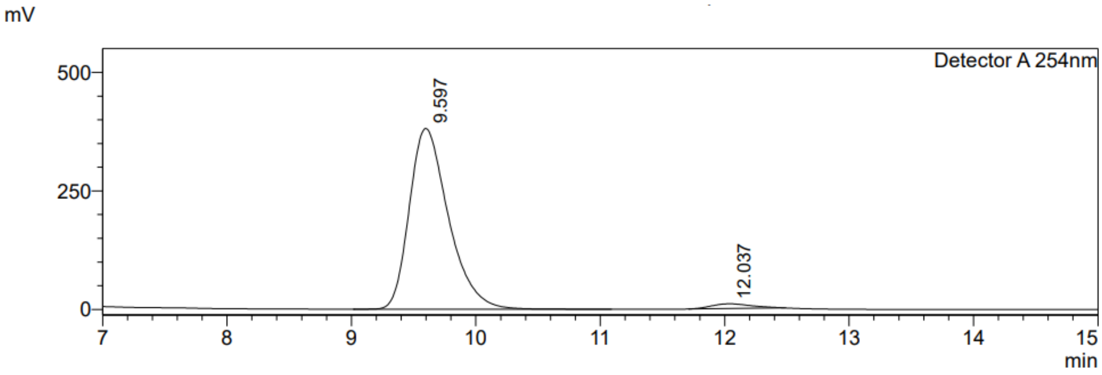

Detector A 254nm

Peak#	Ret. Time	Height	Height%	Area	Area%
1	9.597	381197	97.439	8340511	97.361
2	12.037	10018	2.561	226029	2.639
Total		391215	100.000	8566540	100.000

(*R*)-2-(2,3-dimethylphenyl)-4,5-diphenyl-3,6-dihydro-2*H*-1,2-thiazine 1-oxide (36)

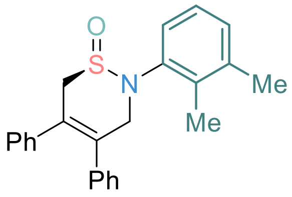

36

White solid; 37 mg, 98% yield; m.p. = 129.7-131.5 °C; ^{1}H NMR (400 MHz, $CDCl_3$) δ 7.38 (d, *J* = 6.8 Hz, 1H), 7.21-7.09 (m, 12H), 4.56 (ddd, *J* = 17.3, 4.4, 2.6 Hz, 1H), 4.11 (ddd, *J* = 16.6, 4.4, 2.8 Hz, 1H), 3.88 (dd, *J* = 17.3, 2.4 Hz, 1H), 3.71 (dd, *J* = 16.6, 2.1 Hz, 1H), 2.33 (s, 3H), 2.30 (s, 3H); ^{13}C NMR (100 MHz, $CDCl_3$) δ 144.83, 140.82, 139.26, 138.54, 134.17, 133.94, 129.31, 129.05, 128.22, 128.17, 127.27, 127.02, 126.60, 124.17, 123.66, 55.57, 48.97, 20.74, 14.35; HRMS (ESI) *m/z* calcd for $C_{24}H_{23}NOS$ $[M+H]^+$ = 374.1578, found = 374.1574.

Optical Rotation: $[\alpha]^{25}_{D}$ = 1124.07 (*c* = 0.54, $CHCl_3$). 96% e.e. (HPLC condition: Chiralpak IG column, *n*-Hexane/*i*-PrOH = 70:30, flow rate = 1.0 mL/min, wavelength = 254 nm, t_R = 14.38 min for major isomer, t_R = 16.40 min for minor isomer).

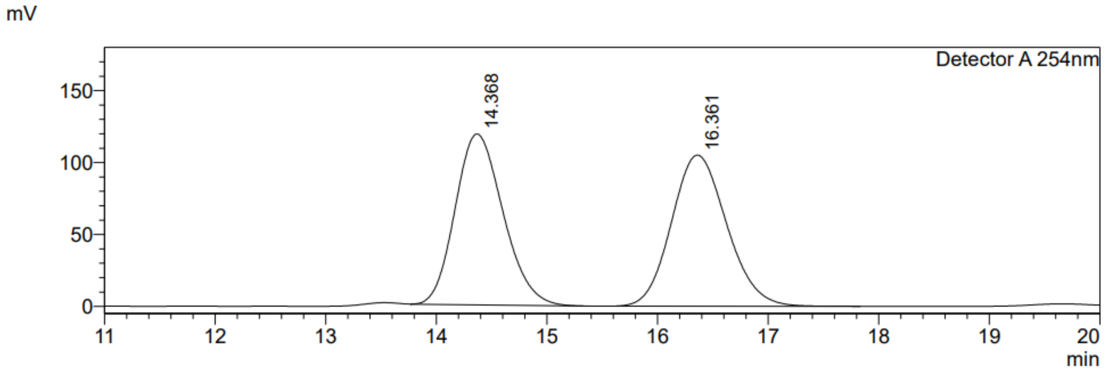

Detector A 254nm

Peak#	Ret. Time	Height	Height%	Area	Area%
1	14.368	118755	53.067	3581134	49.516
2	16.361	105028	46.933	3651097	50.484
Total		223783	100.000	7232230	100.000

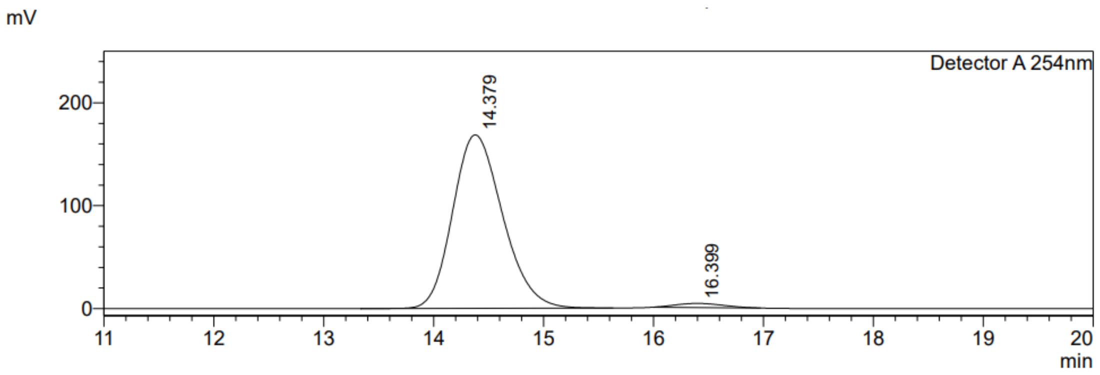

Detector A 254nm

Peak#	Ret. Time	Height	Height%	Area	Area%
1	14.379	168581	97.694	5327338	97.812
2	16.399	3980	2.306	119189	2.188
Total		172561	100.000	5446528	100.000

(*R*)-2-(2,6-dimethoxyphenyl)-4,5-diphenyl-3,6-dihydro-2*H*-1,2-thiazine 1-oxide (37)

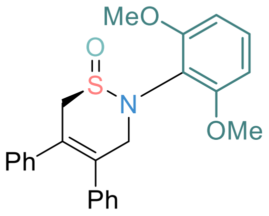

37

White solid; 40 mg, 98% yield; m.p. = 58.4-60.0 °C; ^{1}H NMR (400 MHz, $CDCl_3$) δ 7.20 (t, J = 8.4 Hz, 1H), 7.17-7.08 (m, 10H), 6.59 (d, J = 8.4 Hz, 2H), 4.77 (ddd, J = 17.1, 3.6, 2.2 Hz, 1H), 4.15-4.05 (m, 1H), 3.88-3.81 (m, 1H), 3.84 (s, 6H), 3.70 (dd, J = 15.5, 1.7 Hz, 1H); ^{13}C NMR (100 MHz, $CDCl_3$) δ 141.71, 139.79, 134.85, 129.30, 129.26, 128.29, 127.99, 127.89, 126.85, 126.59, 125.12, 122.05, 56.19, 55.55, 48.75; HRMS (ESI) *m/z* calcd for $C_{24}H_{23}NO3S$ $[M+H]^+$ = 406.1477, found = 406.1475.

Optical Rotation: $[\alpha]^{25}_{D}$ = 113.96 (c = 0.53, $CHCl_3$). 92% e.e. (HPLC condition: Chiralpak IG column, *n*-Hexane/*i*-PrOH =70:30, flow rate = 1.0 mL/min, wavelength = 254 nm, t_R = 17.22 min for minor isomer, t_R = 28.01 min for major isomer).

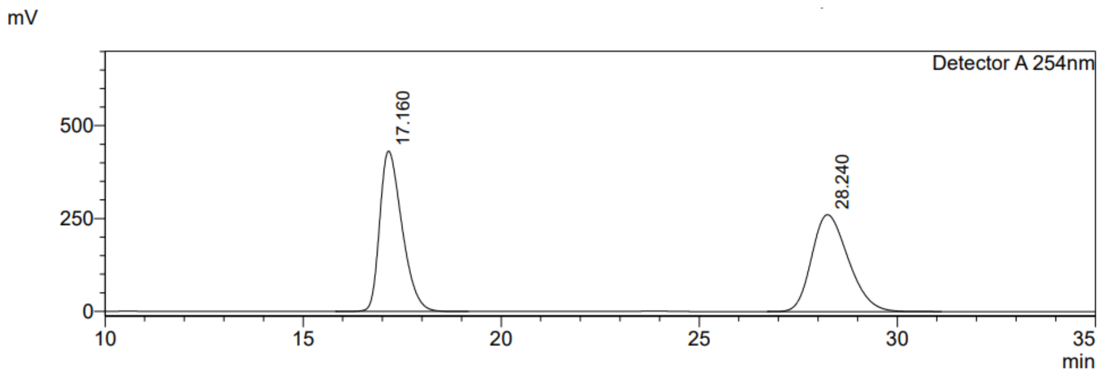

Detector A 254nm

Peak#	Ret. Time	Height	Height%	Area	Area%
1	17.160	432138	62.373	16933461	50.191
2	28.240	260689	37.627	16804713	49.809
Total		692827	100.000	33738173	100.000

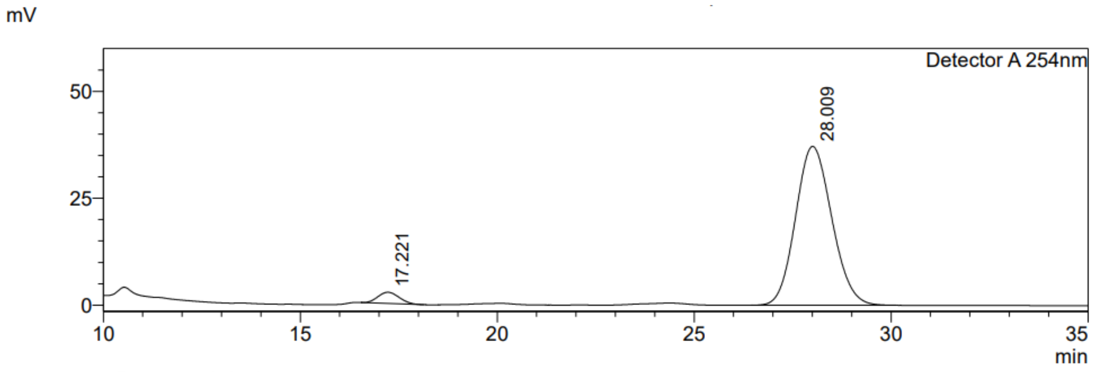

Detector A 254nm

Peak#	Ret. Time	Height	Height%	Area	Area%
1	17.221	2618	6.583	98967	3.991
2	28.009	37157	93.417	2380819	96.009
Total		39776	100.000	2479786	100.000

(*R*)-2-(2,6-dichlorophenyl)-4,5-diphenyl-3,6-dihydro-2*H*-1,2-thiazine 1-oxide (38)

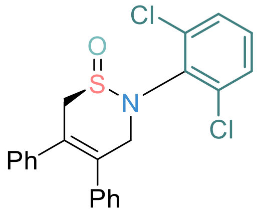

38

White solid; 40 mg, 98% yield; m.p. = 119.2-121.0 ºC; ^{1}H NMR (400 MHz, $CDCl_3$) δ 7.42 (d, *J* = 8.2 Hz, 2H), 7.22 (t， *J* = 8.2 Hz, 1H), 7.19-7.04 (m, 10H), 4.74 (ddd, *J* = 16.3, 2.9, 1.9 Hz, 1H), 4.20 (dt, *J* = 15.3, 3.1 Hz, 1H), 3.93 (dd, *J* = 16.3, 3.1 Hz, 1H), 3.85 (dd, *J* = 15.3, 1.5 Hz, 1H); ^{13}C NMR (100 MHz, $CDCl_3$) δ 141.22, 139.58, 139.34, 136.90, 136.69, 134.77, 129.57, 129.24, 128.88, 128.23, 128.19, 127.31, 127.01,

125.90, 56.41, 49.31; HRMS (ESI) *m/z* calcd for $C_{22}H_{17}Cl_2NOS$ $[M+H]^+$ = 414.0486, found = 414.0482.

Optical Rotation: $[\alpha]^{25}_{D}$ = 31.51 (*c* = 0.53, $CHCl_3$). 89% e.e. (HPLC condition: Chiralpak IC column, *n*-Hexane/*i*-PrOH = 70:30, flow rate = 1.0 mL/min, wavelength = 254 nm, t_R = 15.11 min for minor isomer, t_R = 20.20 min for major isomer).

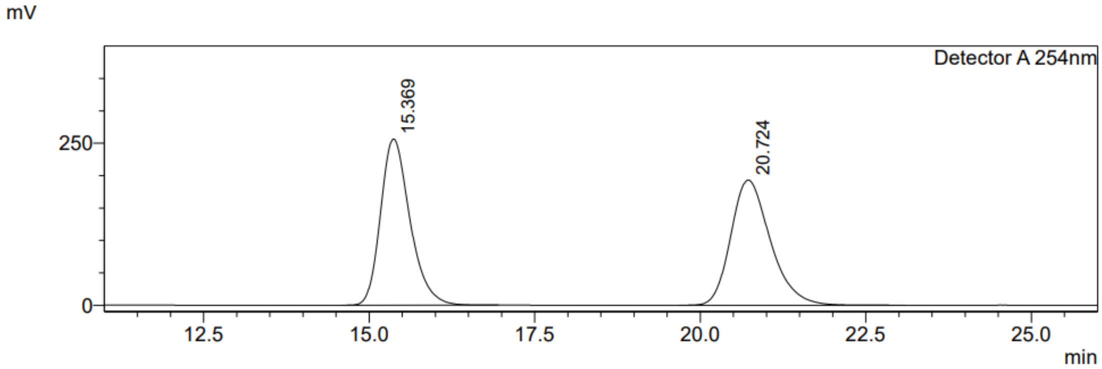

Detector A 254nm

Peak#	Ret. Time	Height	Height%	Area	Area%
1	15.369	256620	57.058	7938372	49.682
2	20.724	193132	42.942	8040070	50.318
Total		449752	100.000	15978442	100.000

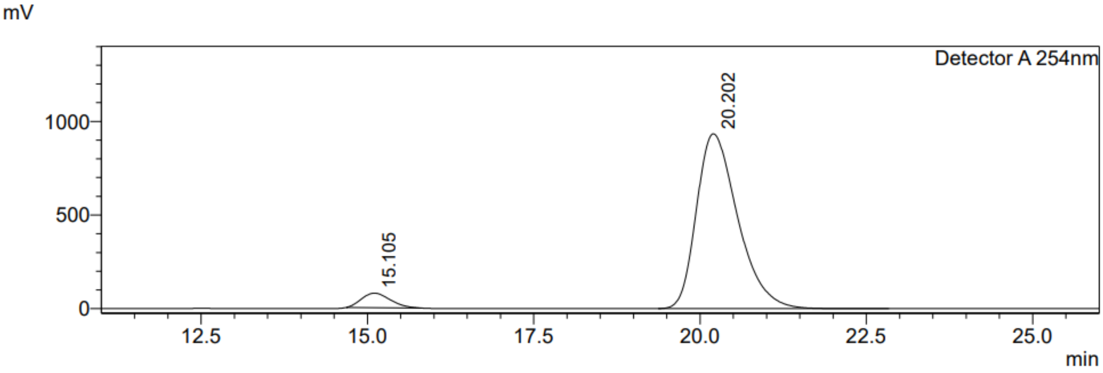

Detector A 254nm

Peak#	Ret. Time	Height	Height%	Area	Area%
1	15.105	77375	7.657	2313833	5.430
2	20.202	933118	92.343	40301253	94.570
Total		1010493	100.000	42615086	100.000

(*R*)-4,5-diphenyl-2-(2,3,4-trimethoxyphenyl)-3,6-dihydro-2*H*-1,2-thiazine 1-oxide (39)

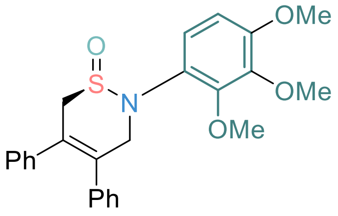

39

White solid; 40 mg, 93% yield; m.p. = 43.8-45.7 °C; ^{1}H NMR (400 MHz, $CDCl_3$) δ 7.18-7.07 (m, 11H), 6.65 (d, *J* = 8.9 Hz, 1H), 4.57 (ddd, *J* = 16.9, 4.1, 2.5 Hz, 1H), 4.13-4.06 (m, 1H), 4.01 (dd, *J* = 16.9, 2.5 Hz, 1H), 3.96 (s, 3H), 3.90 (s, 3H), 3.86 (s, 3H), 3.71 (dd, *J* = 16.4, 2.0 Hz, 1H); ^{13}C NMR (100 MHz, $CDCl_3$) δ 152.74, 149.49, 143.11, 140.88, 139.32, 134.28, 132.89, 129.36, 129.28, 128.20, 128.14, 127.24, 126.97, 124.14, 120.22, 107.03, 61.55, 61.14, 56.23, 55.66, 48.38; HRMS (ESI) *m/z* calcd for $C_{25}H_{25}NO_4S$ $[M+Na]^+$ = 458.1402, found = 458.1410.

Optical Rotation: $[\alpha]^{25}_{D}$ = 90.85 (*c* = 0.47, $CHCl_3$). 97% e.e. (HPLC condition: Chiralpak IG column, *n*-Hexane/*i*-PrOH = 70:30, flow rate = 1.0 mL/min, wavelength = 254 nm, t_R = 24.05 min for major isomer, t_R = 28.48 min for minor isomer).

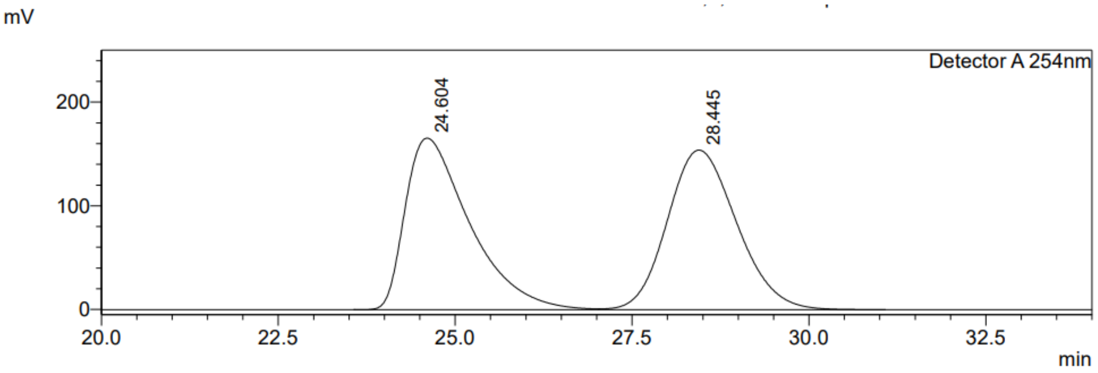

Detector A 254nm

Peak#	Ret. Time	Height	Height%	Area	Area%
1	24.604	165528	51.822	10490926	49.969
2	28.445	153892	48.178	10503979	50.031
Total		319420	100.000	20994905	100.000

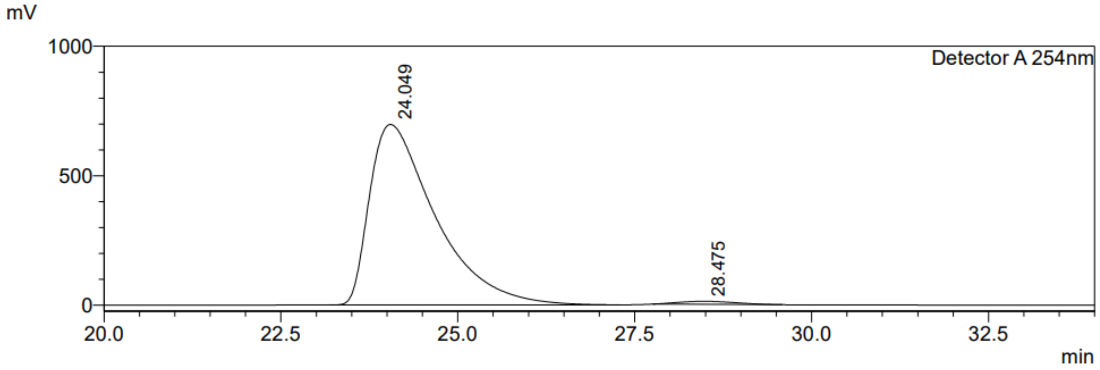

Detector A 254nm

Peak#	Ret. Time	Height	Height%	Area	Area%
1	24.049	697592	98.379	45114265	98.579
2	28.475	11492	1.621	650327	1.421
Total		709085	100.000	45764592	100.000

(*R*)-4-([1,1'-biphenyl]-4-yl)-5-(4-bromophenyl)-2-(naphthalen-1-yl)-3,6-dihydro-2*H*-1,2-thiazine 1-oxide (40)

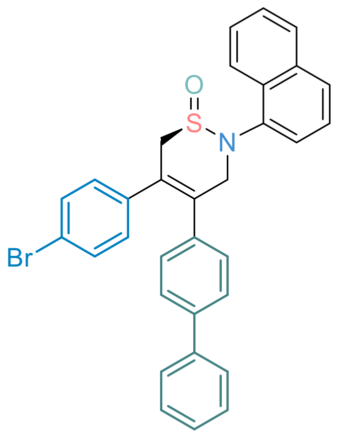

40

Light yellow solid; 25 mg, 45% yield; m.p. = 194.3-195.2 °C; ^{1}H NMR (400 MHz, $CDCl_3$) δ 8.20 (d, *J* = 8.2 Hz, 1H), 7.92 (d, *J* = 7.5 Hz, 1H), 7.84 (d, *J* = 8.3 Hz, 1H), 7.73 (d, *J* = 7.1 Hz, 1H), 7.65-7.60 (m, 1H), 7.59-7.55 (m, 1H), 7.54-7.48 (m, 3H), 7.43-7.31 (m, 7H), 7.18 (d, *J* = 8.3 Hz, 2H), 7.12-7.06 (m, 2H), 4.74 (ddd, *J* = 17.3, 4.3, 2.4 Hz, 1H), 4.28 (ddd, *J* = 16.7, 4.3, 2.8 Hz, 1H), 4.14 (dd, *J* = 17.3, 2.3 Hz, 1H), 3.77 (dd, *J* = 16.7, 2.0 Hz, 1H); ^{13}C NMR (100 MHz, $CDCl_3$) δ 142.61, 140.31, 140.25, 139.74, 137.66, 134.77, 134.66, 131.60, 131.04, 130.46, 129.68, 128.88, 128.68, 128.02, 127.58, 127.02, 127.01, 126.92, 126.69, 126.02, 123.29, 123.17, 122.76, 121.30, 55.67, 49.44; HRMS (ESI) *m/z* calcd for $C_{32}H_{24}BrNOS$ $[M+H]^+$ = 550.0840, found = 550.0838.

Optical Rotation: $[\alpha]^{25}_D$ = 150.87 (*c* = 0.46, $CHCl_3$). 93% e.e. (HPLC condition: Chiralpak IC column, *n*-Hexane/*i*-PrOH = 70:30, flow rate = 1.0 mL/min, wavelength = 254 nm, t_R = 12.43 min for minor isomer, t_R = 36.12 min for major isomer).

(*R*)-5-([1,1'-biphenyl]-4-yl)-4-(4-bromophenyl)-2-(naphthalen-1-yl)-3,6-dihydro-2*H*-1,2-thiazine 1-oxide (41)

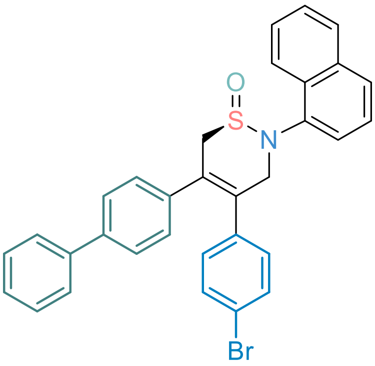

41

Light yellow solid; 25 mg, 46% yield; m.p. = 172.4-174.1 °C; ^{1}H NMR (400 MHz, $CDCl_3$) δ 8.20 (d, J = 8.1 Hz, 1H), 7.92 (d, J = 7.8 Hz, 1H), 7.84 (d, J = 8.2 Hz, 1H), 7.73 (d, J = 6.7 Hz, 1H), 7.65-7.55 (m, 4H), 7.53-7.41 (m, 5H), 7.35 (d, J = 7.3 Hz, 1H), 7.29 (d, J = 8.4 Hz, 2H), 7.22 (d, J = 8.2 Hz, 2H), 7.04 (d, J = 8.4 Hz, 2H), 4.77-4.65 (m, 1H), 4.35-4.24 (m, 1H), 4.09 (dd, J = 17.3, 2.0 Hz, 1H), 3.84 (dd, J = 16.8, 1.7 Hz, 1H); ^{13}C NMR (100 MHz, $CDCl_3$) δ 142.61, 140.33, 140.04, 139.28, 138.05, 134.77, 133.25, 131.55, 131.04, 130.46, 129.72, 128.92, 128.70, 128.03, 127.61, 127.09, 127.06, 126.94, 126.71, 126.03, 124.71, 123.16, 122.74, 121.56, 55.80, 49.22; HRMS (ESI) m/z calcd for $C_{32}H_{24}BrNOS$ $[M+H]^+$ = 550.0840, found = 550.0832.

Optical Rotation: $[\alpha]^{25}_{D}$ = 141.84 (c = 0.49, $CHCl_3$). 91% e.e. (HPLC condition: Chiralpak IC column, n-Hexane/i-PrOH = 70:30, flow rate = 1.0 mL/min, wavelength = 254 nm, t_R = 11.35 min for minor isomer, t_R = 28.75 min for major isomer).

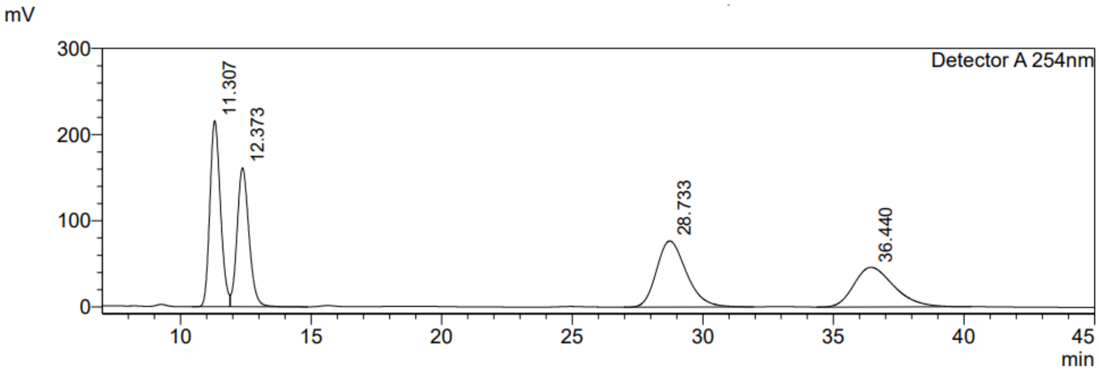

Detector A 254nm

Peak#	Ret. Time	Height	Height%	Area	Area%
1	11.307	215923	43.191	6098127	27.425
2	12.373	161302	32.265	5118084	23.018
3	28.733	76716	15.345	6106459	27.463
4	36.440	45987	9.199	4912902	22.095
Total		499928	100.000	22235571	100.000

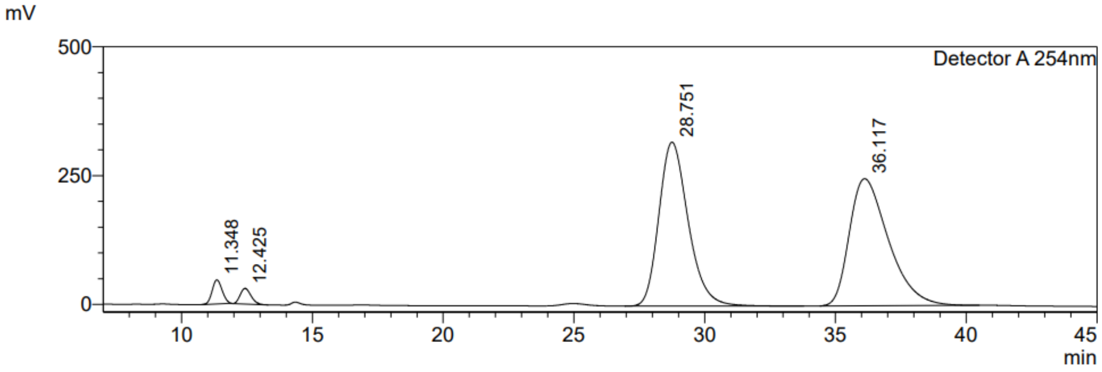

Detector A 254nm

Peak#	Ret. Time	Height	Height%	Area	Area%
1	11.348	46620	7.263	1227309	2.336
2	12.425	30373	4.732	871392	1.659
3	28.751	318299	49.590	24835232	47.270
4	36.117	246571	38.415	25605569	48.736
Total		641863	100.000	52539502	100.000

(*R*)-5-(4-bromophenyl)-4-(4'-methoxy-[1,1'-biphenyl]-4-yl)-2-(naphthalen-1-yl)-3,6-dihydro-2*H*-1,2-thiazine 1-oxide (42)

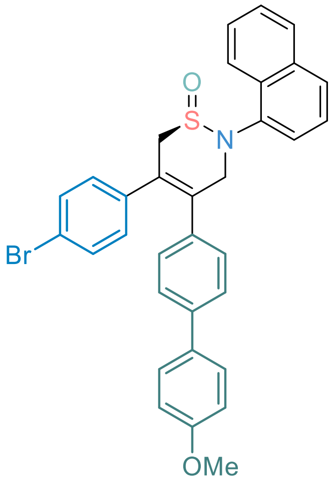

42

Light yellow solid; 23 mg, 40% yield; m.p. = 187.4-188.4 °C; ^{1}H NMR (400 MHz, $CDCl_3$) δ 8.20 (d, *J* = 8.2 Hz, 1H), 7.92 (d, *J* = 7.7 Hz, 1H), 7.83 (d, *J* = 8.3 Hz, 1H), 7.73 (d, *J* = 7.1 Hz, 1H), 7.63 – 7.54 (m, 2H), 7.53 – 7.48 (m, 1H), 7.48 – 7.43 (m, 2H), 7.36 (t, *J* = 8.8 Hz, 4H), 7.15 (d, *J* = 8.3 Hz, 2H), 7.08 (d, *J* = 8.5 Hz, 2H), 6.99-6.89 (m, 2H), 4.73 (ddd, *J* = 17.3, 4.1, 2.3 Hz, 1H), 4.28 (ddd, *J* = 16.7, 4.1, 2.8 Hz, 1H), 4.14 (dd, *J* = 17.3, 2.2 Hz, 1H), 3.82 (s, 3H), 3.77 (dd, *J* = 16.7, 1.9 Hz, 1H); ^{13}C NMR (100 MHz, $CDCl_3$) δ 159.30, 142.52, 139.74, 139.70, 136.88, 134.67, 134.60, 132.72, 131.49, 130.95, 130.36, 129.55, 128.58, 127.95, 127.90, 126.81, 126.59, 126.41,

125.92, 123.04, 122.68, 121.16, 114.23, 55.57, 55.35, 49.35; HRMS (ESI) *m/z* calcd for $C_{33}H_{26}BrNO_2S$ $[M+H]^+$ = 580.0946, found = 580.0940.

Optical Rotation: $[\alpha]^{25}_D$ = 155.49 (*c* = 0.51, $CHCl_3$). 95% e.e. (HPLC condition: Chiralpak ODH column, *n*-Hexane/*i*-PrOH = 70:30, flow rate = 1.0 mL/min, wavelength = 254 nm, t_R = 55.93 min for minor isomer, t_R = 140.169 min for major isomer).

(*R*)-4-(4-bromophenyl)-5-(4'-methoxy-[1,1'-biphenyl]-4-yl)-2-(naphthalen-1-yl)-3,6-dihydro-2*H*-1,2-thiazine 1-oxide (43)

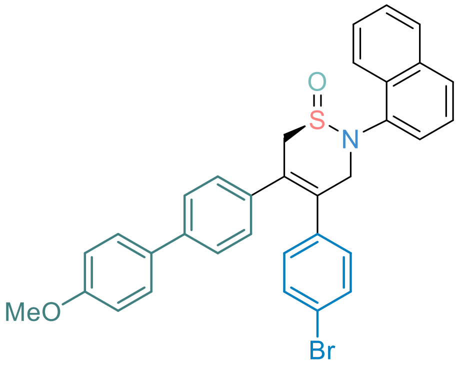

43

Light yellow solid; 25 mg, 44% yield; m.p. = 102.5-103.9 °C; ^1H NMR (400 MHz, $CDCl_3$) δ 8.19 (d, *J* = 8.1 Hz, 1H), 7.92 (d, *J* = 7.7 Hz, 1H), 7.84 (d, *J* = 8.3 Hz, 1H), 7.72 (d, *J* = 6.8 Hz, 1H), 7.63 – 7.55 (m, 2H), 7.54-7.48 (m, 3H), 7.43 (d, *J* = 8.3 Hz, 2H), 7.29 (d, *J* = 8.4 Hz, 2H), 7.19 (d, *J* = 8.2 Hz, 2H), 7.03 (d, *J* = 8.4 Hz, 2H), 6.96 (d, *J* = 8.7 Hz, 2H), 4.74-4.64 (m, 1H), 4.34-4.23 (m, 1H), 4.08 (dd, *J* = 17.2, 2.0 Hz, 1H), 3.85 (s, 3H), 3.84-3.72 (m, 1H); ^{13}C NMR (100 MHz, $CDCl_3$) δ 159.42, 142.62, 139.64, 138.58, 138.11, 134.77, 133.07, 132.84, 131.53, 131.04, 130.45, 129.68, 128.68, 128.08, 128.01, 126.93, 126.70, 126.58, 126.02, 124.74, 123.17, 122.74, 121.51, 114.36, 55.80, 55.47, 49.22; HRMS (ESI) *m/z* calcd for $C_{33}H_{26}BrNO_2S$ $[M+H]^+$ = 580.0946, found = 580.0939.

Optical Rotation: $[\alpha]^{25}_D$ = 26.98 (*c* = 0.43, $CHCl_3$). 90% e.e. (HPLC condition: Chiralpak ODH column, *n*-Hexane/*i*-PrOH = 70:30, flow rate = 1.0 mL/min, wavelength = 254 nm, t_R = 14.57 min for minor isomer, t_R = 73.60 min for major isomer).

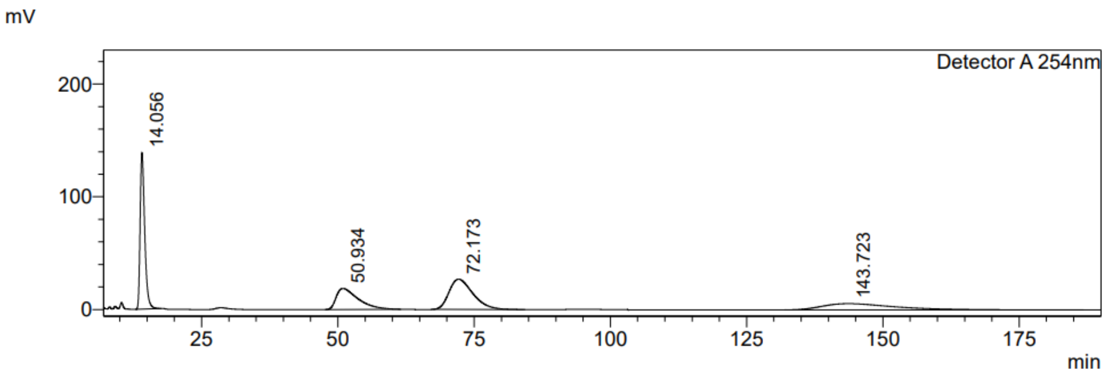

Detector A 254nm

Peak#	Ret. Time	Height	Height%	Area	Area%
1	14.056	138919	73.101	8099324	30.216
2	50.934	18687	9.833	5208631	19.432
3	72.173	26862	14.135	8230701	30.706
4	143.723	5569	2.931	5265831	19.645
Total		190038	100.000	26804487	100.000

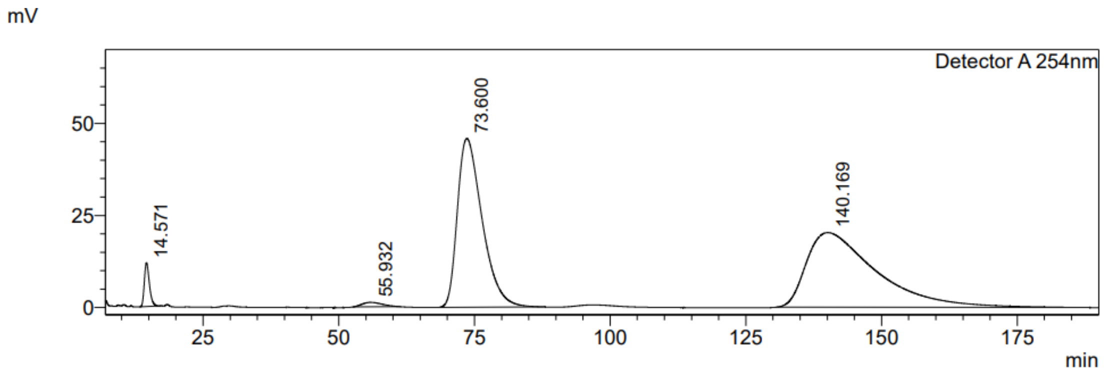

Detector A 254nm

Peak#	Ret. Time	Height	Height%	Area	Area%
1	14.571	11907	15.037	770067	2.284
2	55.932	1221	1.542	318826	0.946
3	73.600	45810	57.852	14553438	43.173
4	140.169	20246	25.568	18067619	53.597
Total		79184	100.000	33709950	100.000

Approach to *N*-alkyl sulfinamide by one-pot operation

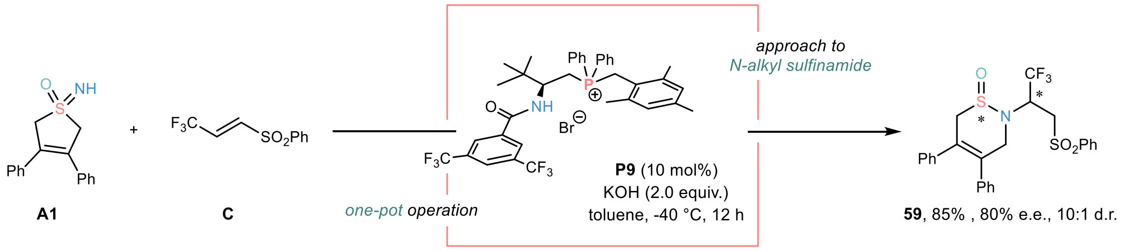

To a tube (20 mL) with a magnetic stirring bar, the sulfoximines **A1** (0.10 mmol), **C** (0.12 mmol), catalyst **P9** (10 mol%) and KOH (0.20 mmol) were added, followed by the addition of toluene (1.0 mL). The reaction mixture was stirred at -40 ^{o}C. After completion, the reaction mixture was transfered to room temperature, and diluted with

dichloromethane. Purification by column chromatography on silica gel (petroleum ether/ethyl acetate = 2:1) afforded the prodcut **59**.

<u>4,5-diphenyl-2-(1,1,1-trifluoro-3-(phenylsulfonyl)-2λ^3-propan-2-yl)-3,6-dihydro-2H-1,2-thiazine 1-oxide (59)</u>

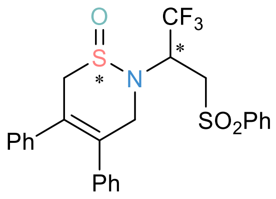

59

White solid; 43 mg, 85% yield; m.p. = 141.0-142.2 °C; ^{1}H NMR (400 MHz, $CDCl_3$) δ 8.07-7.86 (m, 2H), 7.79-7.69 (m, 1H), 7.64 (t, J = 7.6 Hz, 2H), 7.24-7.04 (m, 8H), 7.06-76.96 (m, 2H), 4.76-4.61 (m, 1H), 4.19 (d, J = 16.6 Hz, 1H), 4.04-3.94 (m, 1H), 3.90-3.68 (m, 2H), 3.59 (dd, J = 16.0, 1.8 Hz, 1H), 3.39 (dd, J = 14.8, 2.8 Hz, 1H); ^{13}C NMR (100 MHz, $CDCl_3$) δ 140.64, 139.20 (d, J = 6.6 Hz), 134.68, 131.89, 129.91, 129.24, 129.10, 128.29, 128.18, 127.99, 127.46, 127.07, 124.46, 60.64 (q, J = 31.7 Hz), 55.24, 51.61, 43.09, 29.82; ^{19}F NMR (376 MHz, $CDCl_3$) δ -70.92; HRMS (ESI) *m/z* calcd for $C_{25}H_{22}F_3NO_3S_2$ $[M+H]^+$ = 506.1071, found = 506.1068.

Optical Rotation: $[\alpha]^{25}_D$ = 69.81 (c = 0.54, $CHCl_3$). 80% e.e. (HPLC condition: Chiralpak ODH column, *n*-Hexane/*i*-PrOH = 70:30, flow rate = 1.0 mL/min, wavelength = 254 nm, t_R = 9.56 min for major isomer, t_R = 13.37 min for minor isomer).

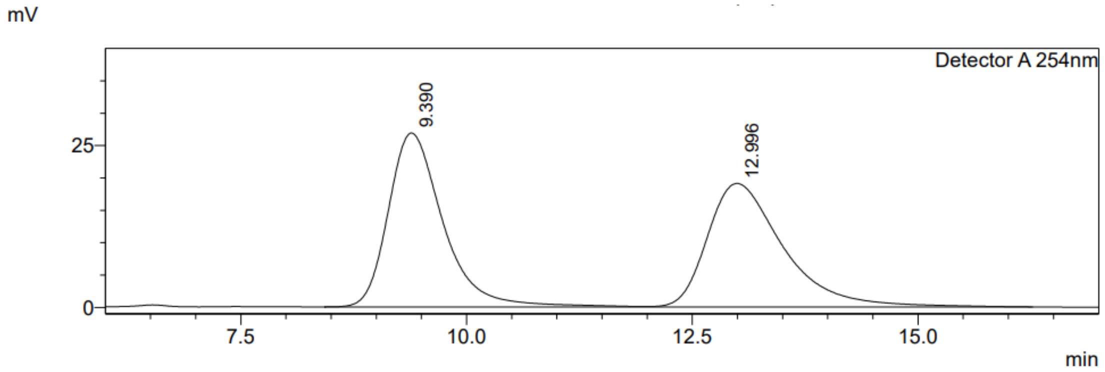

Detector A 254nm

Peak#	Ret. Time	Height	Height%	Area	Area%
1	9.390	26857	58.480	1133510	50.434
2	12.996	19068	41.520	1114008	49.566
Total		45925	100.000	2247518	100.000

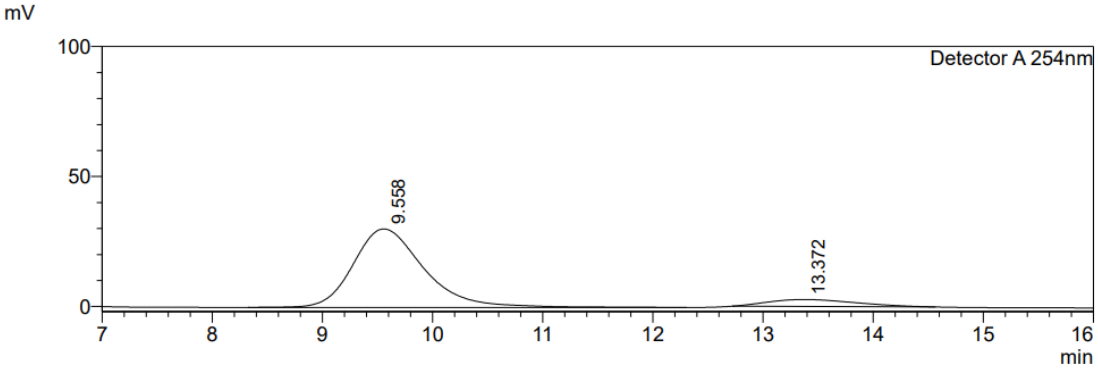

Detector A 254nm

Peak#	Ret. Time	Height	Height%	Area	Area%
1	9.558	30217	91.870	1351726	90.129
2	13.372	2674	8.130	148043	9.871
Total		32891	100.000	1499769	100.000

7. Applications of Chiral Cyclic Sulfinamides

Scaled-up preparation

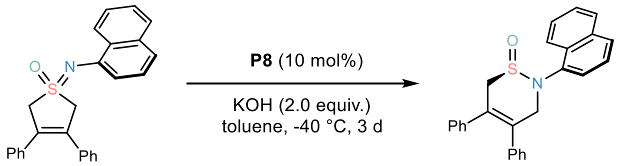

A5 1

To a flame-dried round bottle flask with a magnetic stirring bar were added **A5** (1.0 g, 2.5 mmol), **P8** (10 mol%) and KOH (2.0 equiv.), followed by the addition of toluene (25 mL). The reaction mixture was stirred at -40 ºC for 3 d. Until completion, the mixture was extracted with CH_2Cl_2 (50 mL x 3) and water (50 mL). The combined organic extracts were dried over anhydrous magnesium sulfate, filtered and concentrated under reduced pressure, and the residue was purified by column chromatography on silica gel (petroleum ether/ethyl acetate = 5:1) to afford the prodcut **1** (0.89 g, 90% yield, 94% e.e.) as a white solid.

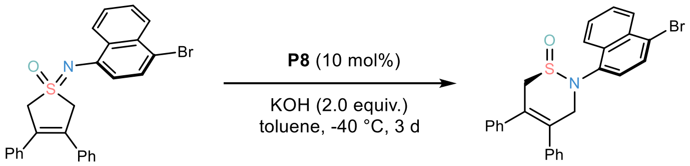

A21 17

To a flame-dried round bottle flask with a magnetic stirring bar were added **A21** (0.5 g, 1.1 mmol), **P8** (10 mol%) and KOH (2.0 equiv.), followed by the addition of

toluene (11 mL). The reaction mixture was stirred at -40 ºC for 3 d. Until completion, the mixture was extracted with CH_2Cl_2 (50 mL x 3) and water (50 mL). The combined organic extracts were dried over anhydrous magnesium sulfate, filtered and concentrated under reduced pressure, and the residue was purified by column chromatography on silica gel (petroleum ether/ethyl acetate = 5:1) to afford the prodcut **17** (0.45 g, 86% yield, 91% e.e.) , The enantiomeric excess can be increased up to >99% through a recrystallization in CH_2Cl_2/hexane. The crystals was easily afforded from solution with 34% yield.

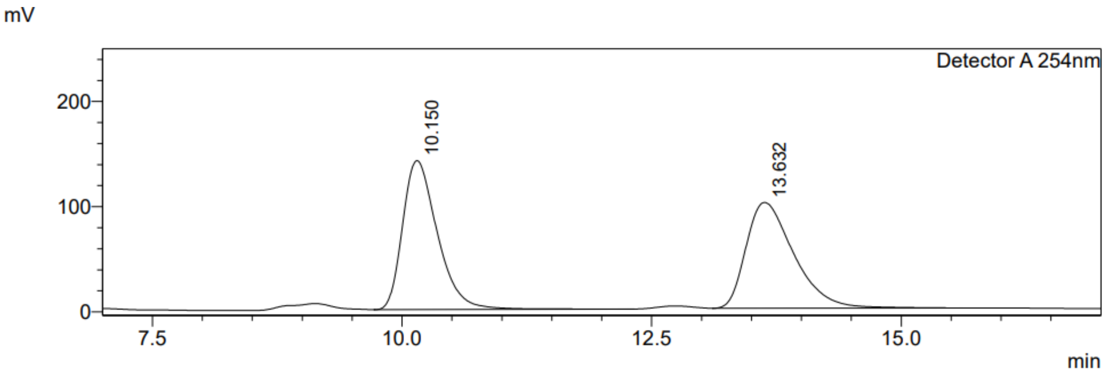

Detector A 254nm

Peak#	Ret. Time	Height	Height%	Area	Area%
1	10.150	141732	58.484	3439267	50.651
2	13.632	100613	41.516	3350793	49.349
Total		242345	100.000	6790060	100.000

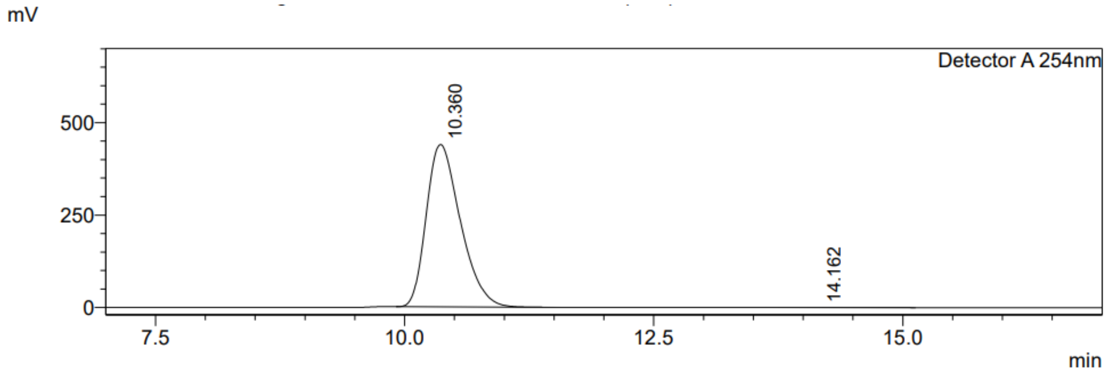

Detector A 254nm

Peak#	Ret. Time	Height	Height%	Area	Area%
1	10.360	439236	99.842	10472111	99.775
2	14.162	694	0.158	23572	0.225
Total		439929	100.000	10495683	100.000

Late-stage diversification

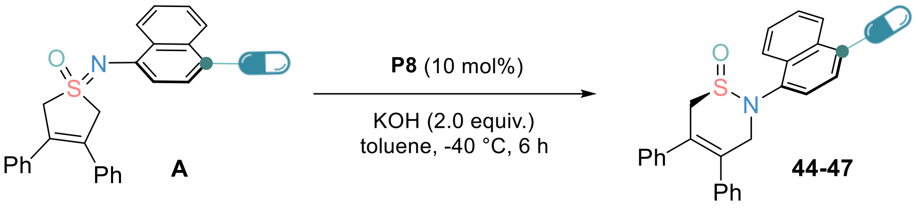

To a tube (20 mL) with a magnetic stirring bar, the sulfoximines **A** (0.10 mmol), catalyst **P8** (10 mol%) and KOH (0.20 mmol) were added, followed by the addition of toluene (1.0 mL). The reaction mixture was stirred at -40 °C. After completion, the reaction mixture was transfered to room temperature, and diluted with dichloromethane. Purification by column chromatography on silica gel (petroleum ether/ethyl acetate = 10:1 to 5:1) afforded the prodcut **44-47**.

(*R*)-4-(1-oxido-4,5-diphenyl-3,6-dihydro-2*H*-1,2-thiazin-2-yl)naphthalen-1-yl 5-(2,5-dimethylphenoxy)-2,2-dimethylpentanoate (44)

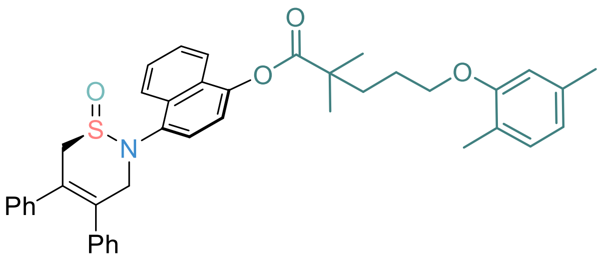

44

White solid; 59 mg, 92% yield; m.p. = 54.2-55.1 °C; ^{1}H NMR (400 MHz, $CDCl_3$) δ 8.24 (d, *J* = 8.2 Hz, 1H), 8.02-7.92 (m, 1H), 7.74 (d, *J* = 7.2 Hz, 1H), 7.69-7.55 (m, 2H), 7.25-7.11 (m, 11H), 7.03 (d, *J* = 7.4 Hz, 1H), 6.73-6.64 (m, 2H), 4.72 (ddd, *J* = 17.2, 4.2, 2.4 Hz, 1H), 4.29 (ddd, *J* = 16.7, 4.2, 2.7 Hz, 1H), 4.12 (dd, *J* = 17.2, 2.3 Hz, 1H), 4.06 (t, *J* = 5.8 Hz, 2H), 3.82 (dd, *J* = 16.7, 2.0 Hz, 1H), 2.33 (s, 3H), 2.21 (s, 3H), 2.09-1.96 (m, 4H), 1.54 (d, *J* = 1.8 Hz, 6H); ^{13}C NMR (100 MHz, $CDCl_3$) δ 176.25, 156.99, 146.30, 140.71, 140.52, 139.03, 136.61, 134.27, 131.64, 130.48, 129.33, 129.32, 128.29, 128.21, 128.13, 127.36, 127.16, 127.13, 124.22, 123.72, 123.28, 123.04, 121.88, 120.94, 118.02, 112.12, 67.91, 55.89, 43.01, 37.34, 25.48, 25.46, 25.43, 21.53, 15.94; HRMS (ESI) *m/z* calcd for $C_{41}H_{41}NO_4S$ $[M+H]^+$ = 644.2834, found = 644.2838.

Optical Rotation: $[\alpha]^{25}_{D}$ = 95.10 (c = 0.49, $CHCl_3$). 94% e.e. (HPLC condition: Chiralpak IC column, n-Hexane/i-PrOH = 70:30, flow rate = 1.0 mL/min, wavelength = 254 nm, t_R = 18.78 min for minor isomer, t_R = 22.13 min for major isomer).

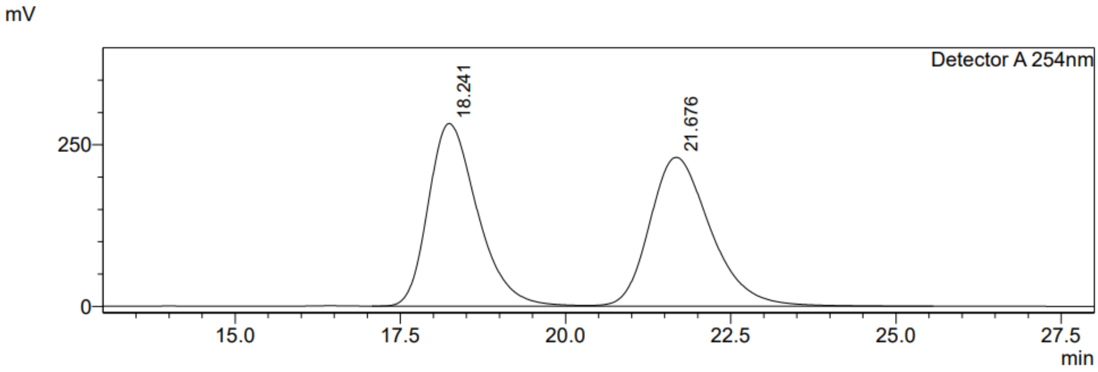

Detector A 254nm

Peak#	Ret. Time	Height	Height%	Area	Area%
1	18.241	282608	55.103	14657200	49.750
2	21.676	230267	44.897	14804464	50.250
Total		512875	100.000	29461664	100.000

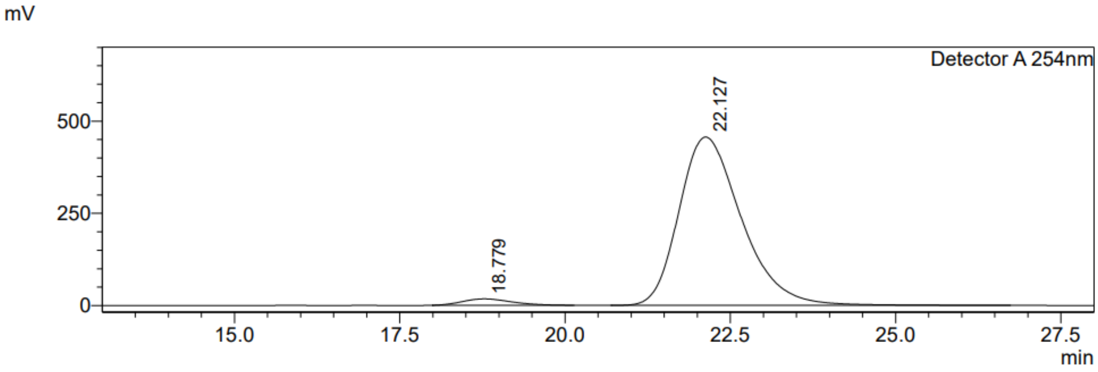

Detector A 254nm

Peak#	Ret. Time	Height	Height%	Area	Area%
1	18.779	17321	3.658	896403	2.895
2	22.127	456224	96.342	30063584	97.105
Total		473546	100.000	30959986	100.000

(*R*)-4-(1-oxido-4,5-diphenyl-3,6-dihydro-2*H*-1,2-thiazin-2-yl)naphthalen-1-yl stearate (45)

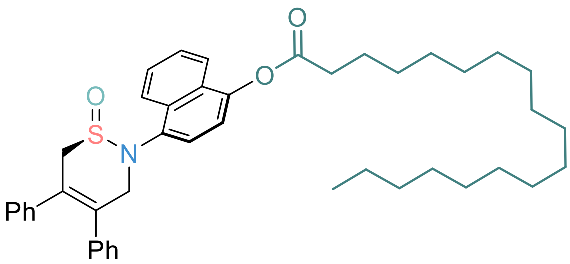

45

White solid; 50 mg, 74% yield; m.p. = 73.5-74.8 °C; ^{1}H NMR (400 MHz, $CDCl_3$) δ 8.22 (d, J = 7.9 Hz, 1H), 7.93 (d, J = 7.9 Hz, 1H), 7.74 (d, J = 6.9 Hz, 1H), 7.67-7.57 (m, 2H), 7.29-7.26 (m, 1H), 7.21-7.10 (m, 10H), 4.71 (d, J = 17.2 Hz, 1H), 4.34-4.23 (m, 1H), 4.11 (dd, J = 17.2, 2.0 Hz, 1H), 3.82 (dd, J = 16.7, 1.9 Hz, 1H), 2.75 (t, J = 7.5 Hz, 2H), 1.93-1.84 (m, 2H), 1.39-1.24 (m, 28H), 0.89 (t, J = 6.8 Hz, 3H); ^{13}C NMR (100 MHz, $CDCl_3$) δ 172.25, 146.07, 140.68, 140.60, 139.01, 134.22, 131.62, 129.33, 129.31, 128.28, 128.20, 127.99, 127.36, 127.12, 124.13, 123.25, 123.08, 122.01, 118.20, 55.87, 49.54, 34.52, 32.04, 29.82, 29.78, 29.74, 29.61, 29.48, 29.41, 29.34, 25.19, 22.81, 14.25; HRMS (ESI) m/z calcd for $C_{44}H_{55}NO_3S$ $[M+H]^+$ = 678.3981, found = 678.3989.

Optical Rotation: $[\alpha]^{25}_D$ = 86.80 (c = 0.50, $CHCl_3$). 93% e.e. (HPLC condition: Chiralpak IC column, n-Hexane/i-PrOH = 70:30, flow rate = 1.0 mL/min, wavelength = 254 nm, t_R = 13.43 min for minor isomer, t_R = 18.87 min for major isomer).

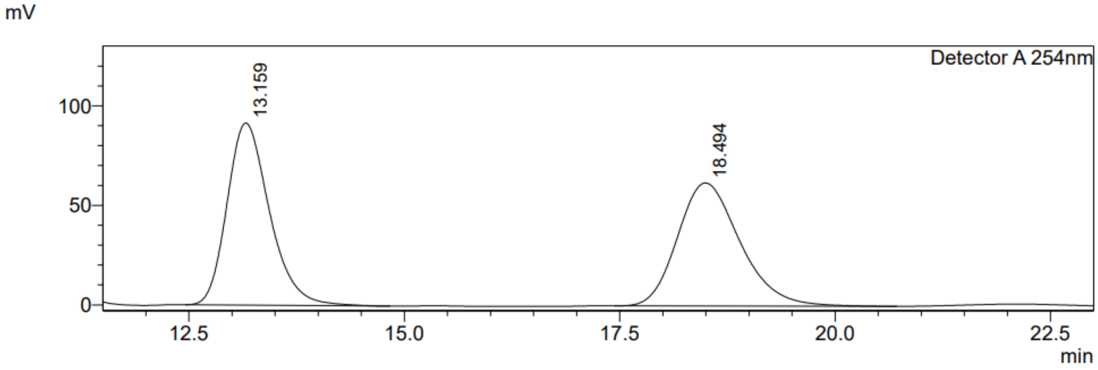

Detector A 254nm

Peak#	Ret. Time	Height	Height%	Area	Area%
1	13.159	91501	59.672	3138424	50.091
2	18.494	61837	40.328	3126997	49.909
Total		153338	100.000	6265421	100.000

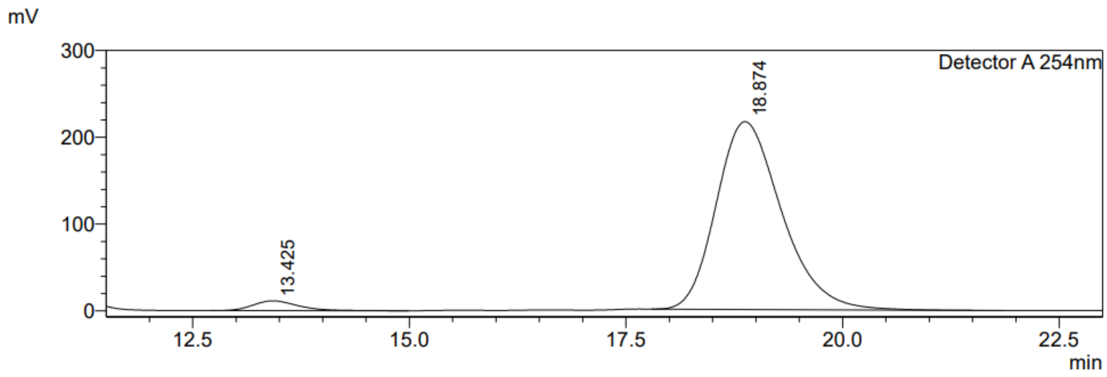

Detector A 254nm

Peak#	Ret. Time	Height	Height%	Area	Area%
1	13.425	11289	4.959	409178	3.471
2	18.874	216360	95.041	11377678	96.529
Total		227649	100.000	11786856	100.000

4-((*R*)-1-oxido-4,5-diphenyl-3,6-dihydro-2*H*-1,2-thiazin-2-yl)naphthalen-1-yl (7*Z*,10*Z*)-octadeca-7,10-dienoate (46)

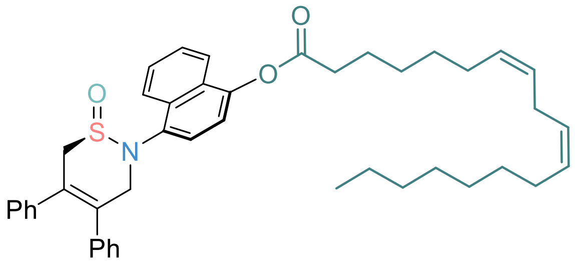

46

White solid; 49 mg, 73% yield; m.p. = 110.8-111.4 °C; ^{1}H NMR (400 MHz, $CDCl_3$) δ 8.22 (d, *J* = 7.9 Hz, 1H), 7.93 (d, *J* = 8.0 Hz, 1H), 7.74 (d, *J* = 6.8 Hz, 1H), 7.68-7.56 (m, 2H), 7.28 (s, 1H), 7.23-7.09 (m, 10H), 5.50-5.26 (m, 4H), 4.71 (d, *J* = 16.5 Hz, 1H), 4.34-4.23 (m, 1H), 4.11 (dd, *J* = 17.2, 2.0 Hz, 1H), 3.82 (dd, *J* = 16.5, 1.9 Hz, 1H), 2.87-2.67 (m, 4H), 2.16-1.99 (m, 4H), 1.93-1.80 (m, 2H), 1.55-1.23 (m, 14H), 0.92-0.88 (m, 3H); ^{13}C NMR (100 MHz, $CDCl_3$) δ 172.22, 146.06, 140.68, 140.61, 139.01, 134.23, 131.62, 130.34, 130.10, 129.34, 129.31, 128.28, 128.22, 128.20, 128.00, 127.98, 127.36, 127.12, 123.27, 123.07, 122.01, 118.20, 55.86, 49.53, 34.50, 31.64, 29.72, 29.46, 29.31, 29.24, 27.32, 27.31, 25.76, 25.16, 22.69, 14.21; HRMS (ESI) *m/z* calcd for $C_{44}H_{51}NO_3S$ $[M+H]^+$ = 674.3688, found = 674.3662.

Optical Rotation: $[\alpha]^{25}_D$ = 16.17 (*c* = 0.47, $CHCl_3$). 92% e.e. (HPLC condition: Chiralpak ODH column, *n*-Hexane/*i*-PrOH = 60:40, flow rate = 1.0 mL/min, wavelength = 254 nm, t_R = 5.69 min for minor isomer, t_R = 11.80 min for major isomer).

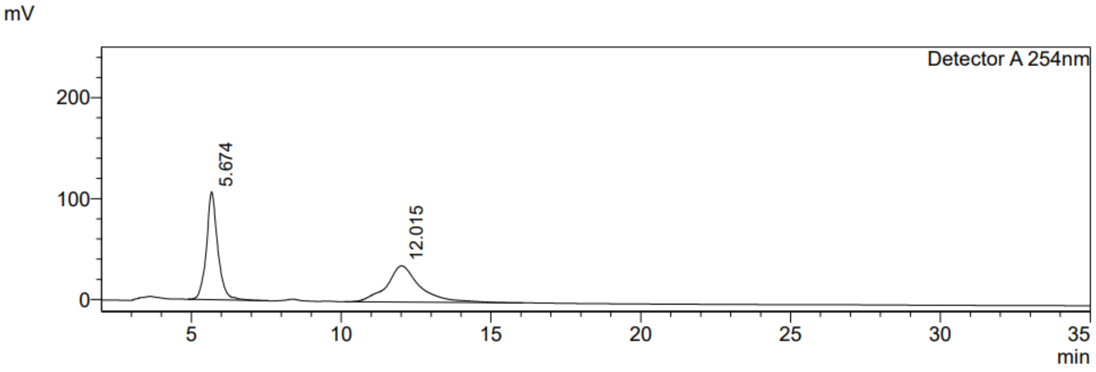

Detector A 254nm

Peak#	Ret. Time	Height	Height%	Area	Area%
1	5.674	106771	74.846	2824406	50.366
2	12.015	35883	25.154	2783388	49.634
Total		142654	100.000	5607794	100.000

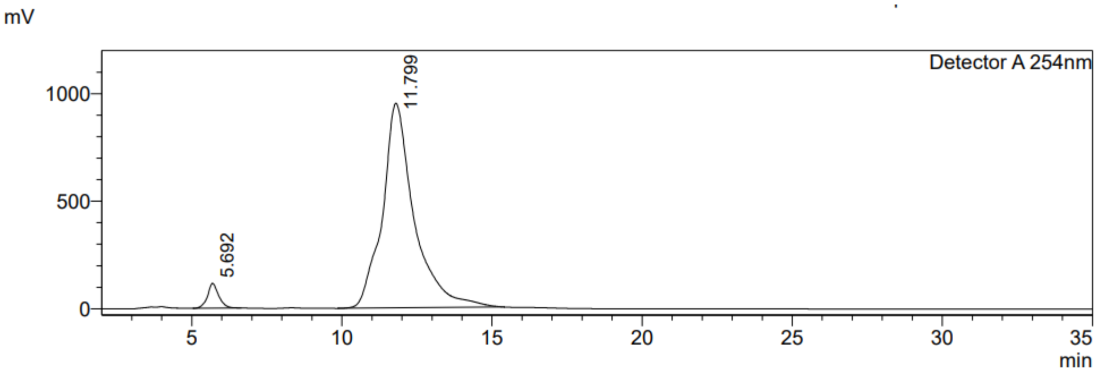

Detector A 254nm

Peak#	Ret. Time	Height	Height%	Area	Area%
1	5.692	115429	10.823	2983567	4.074
2	11.799	951106	89.177	70248161	95.926
Total		1066535	100.000	73231728	100.000

4-((*R*)-1-oxido-4,5-diphenyl-3,6-dihydro-2*H*-1,2-thiazin-2-yl)naphthalen-1-yl (3*R*,5*R*,7*R*)-adamantane-1-carboxylate (47)

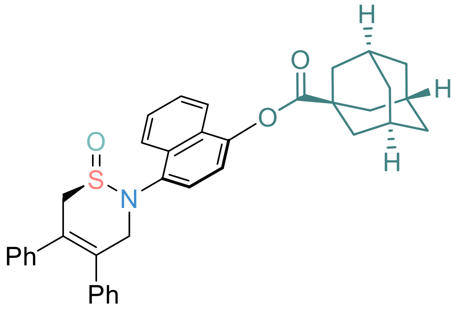

47

White solid; 55 mg, 96% yield; m.p. = 210.3-211.7 °C; ^{1}H NMR (400 MHz, $CDCl_3$) δ 8.21 (d, *J* = 7.9 Hz, 1H), 7.96-7.88 (m, 1H), 7.73 (d, *J* = 7.1 Hz, 1H), 7.67-7.55 (m, 2H), 7.24-7.06 (m, 11H), 4.70 (ddd, *J* = 17.2, 4.0, 2.4 Hz, 1H), 4.28 (ddd, *J* = 16.7, 4.0, 2.8 Hz, 1H), 4.10 (dd, *J* = 17.2, 2.3 Hz, 1H), 3.81 (dd, *J* = 16.7, 2.0 Hz, 1H), 2.28-2.19 (m,

6H), 2.16 (s, 3H), 1.89-1.79 (m, 6H);^{13}C NMR (100 MHz, $CDCl_3$) δ 176.04, 146.35, 140.72, 140.43, 139.04, 134.24, 131.63, 129.34, 129.32, 128.28, 128.21, 128.16, 127.36, 127.31, 127.12, 127.08, 124.18, 123.23, 123.09, 121.98, 118.13, 55.91, 49.58, 41.60, 39.11, 36.58, 28.07; HRMS (ESI) *m/z* calcd for $C_{37}H_{35}NO_3S$ $[M+H]^+$ = 574.2416, found = 574.2423.

Optical Rotation: $[\alpha]^{25}_D$ = 105.77 (*c* = 0.52, $CHCl_3$). 94% e.e. (HPLC condition: Chiralpak IC column, *n*-Hexane/*i*-PrOH = 70:30, flow rate = 1.0 mL/min, wavelength = 254 nm, t_R = 23.54 min for minor isomer, t_R = 35.76 min for major isomer).

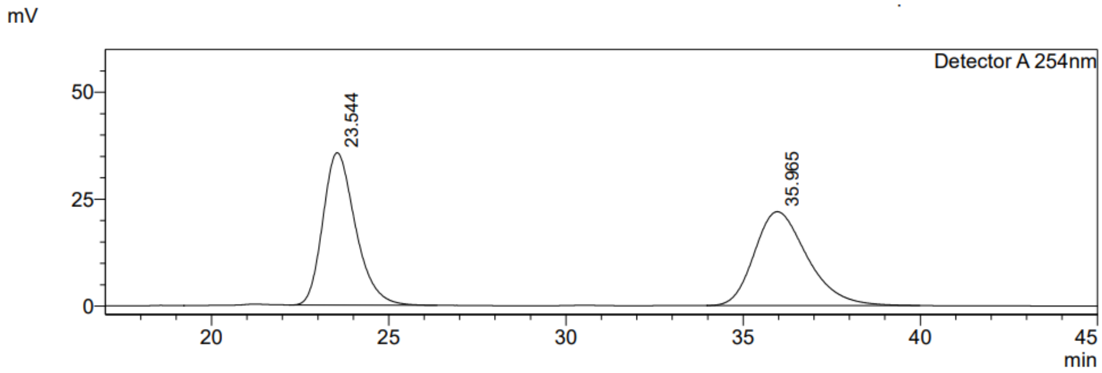

Detector A 254nm

Peak#	Ret. Time	Height	Height%	Area	Area%
1	23.544	35630	61.874	2282188	50.003
2	35.965	21955	38.126	2281904	49.997
Total		57585	100.000	4564092	100.000

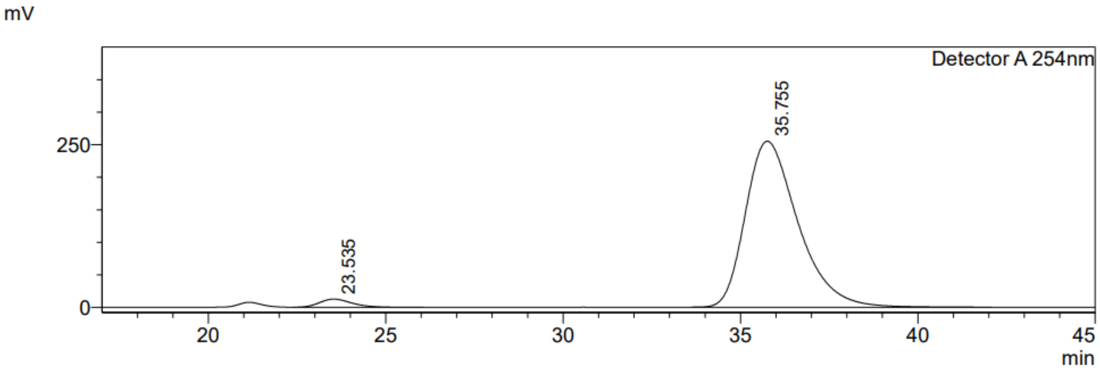

Detector A 254nm

Peak#	Ret. Time	Height	Height%	Area	Area%
1	23.535	12621	4.716	808877	2.980
2	35.755	255021	95.284	26333710	97.020
Total		267642	100.000	27142588	100.000

Derivatization of the chiral products

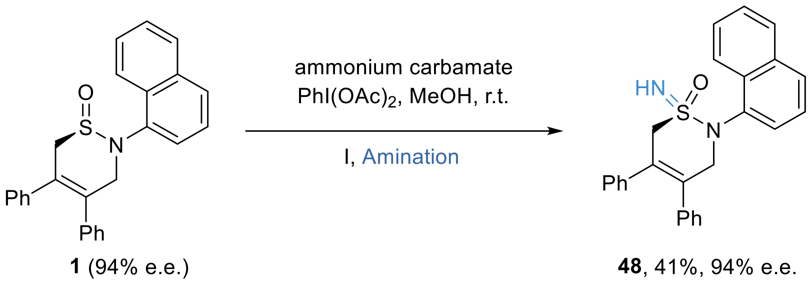

I, Amination : To a 25 mL round-bottom flask with a magnetic stirring bar were added the sulfinamide **1** (0.1 mmol), ammonium carbamate (2.0 equiv.) and iodobenzene diacetate (2.5 equiv.). The mixture was dissolved in methanol (5.0 mL), and then stirred at r.t. for 12 h. The solvent was removed in vacuo, and the residue was purified by a column chromatography on silica gel (PE/EA= 1/2) to afford the product **48** as a light yellow solid.

(*S*)-1-imino-2-(naphthalen-1-yl)-4,5-diphenyl-1,2,3,6-tetrahydro-1λ^6,2-thiazine 1-oxide (48)

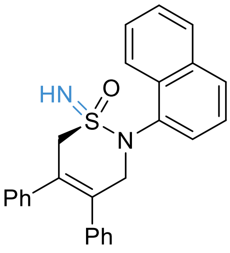

48

White solid; 17 mg, 41% yield; m.p. = 1159.1-160.2 °C; ^{1}H NMR (400 MHz, $CDCl_3$) δ 8.40 (d, *J* = 8.4 Hz, 1H), 7.90-7.87 (m, 2H), 7.85-7.78 (m, 1H), 7.58-7.50 (m, 3H), 7.21-7.14 (m, 10H), 4.67-4.65 (m, 1H), 4.59 (dt, *J* = 17.0, 2.9 Hz, 1H), 4.56-4.40 (m, 1H), 4.20-4.15 (m, 1H), 2.53 (s, 1H); ^{13}C NMR (100 MHz, $CDCl_3$) δ 139.83, 139.61, 138.21, 134.79, 134.35, 132.09, 129.19, 129.10, 128.86, 128.71, 128.41, 128.29, 127.53, 127.50, 127.12, 126.75, 125.63, 124.12, 123.58, 122.23, 54.73, 51.12; HRMS (APCI) *m/z* calcd for $C_{26}H_{22}N_2OS$ $[M+H]^+$ = 411.1531, found = 411.1526.

Optical Rotation: $[\alpha]^{25}_D$ = 72.96 (*c* = 0.46, $CHCl_3$). 94% e.e. (HPLC condition: Chiralpak IG column, *n*-Hexane/*i*-PrOH = 70:30, flow rate = 1.0 mL/min, wavelength = 254 nm, t_R = 18.20 min for major isomer, t_R = 22.56 min for minor isomer).

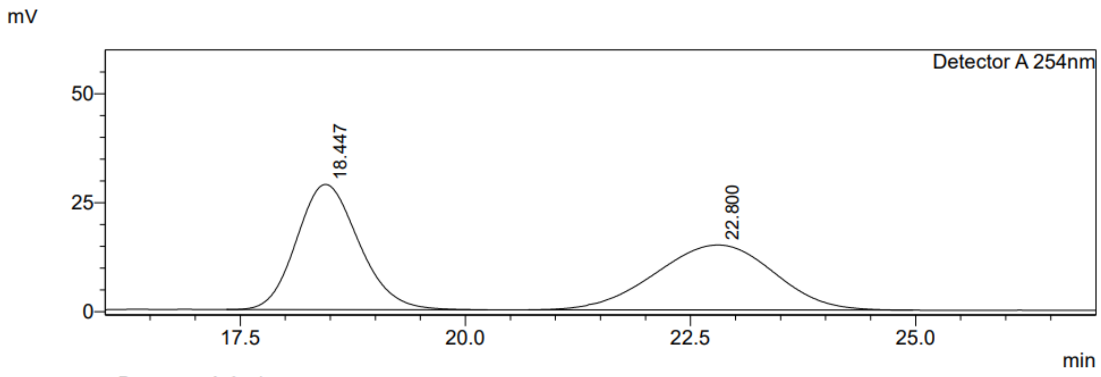

Detector A 254nm

Peak#	Ret. Time	Height	Height%	Area	Area%
1	18.447	28741	65.848	1387069	50.312
2	22.800	14907	34.152	1369858	49.688
Total		43648	100.000	2756927	100.000

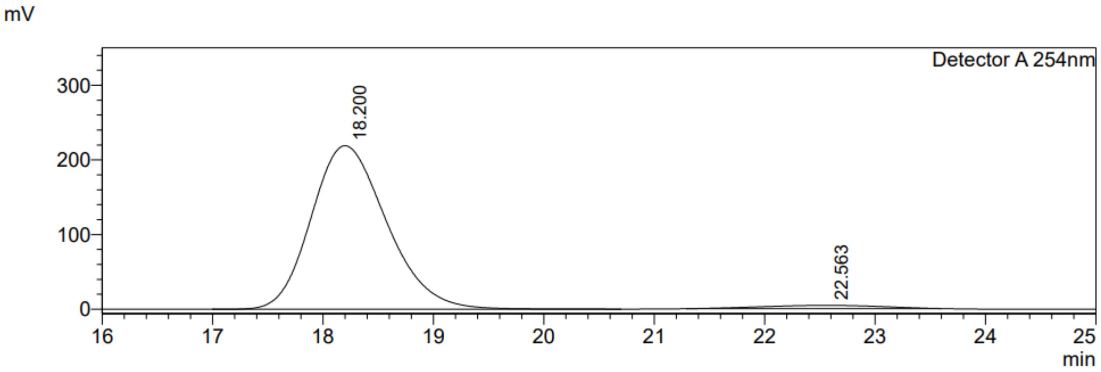

Detector A 254nm

Peak#	Ret. Time	Height	Height%	Area	Area%
1	18.200	218971	98.039	10409609	96.910
2	22.563	4380	1.961	331888	3.090
Total		223350	100.000	10741498	100.000

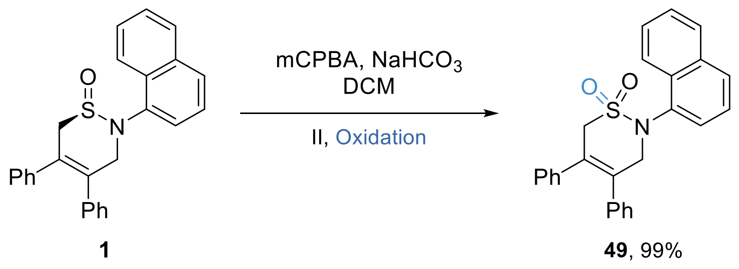

II, Oxidation: To a 25 mL round-bottom flask with a magnetic stirring bar were added the sulfinamide **1** (0.1 mmol), 3-chloroperbenzoic acid (1.4 equiv.) and $NaHCO_3$ (2.5 equiv.). The mixture was dissolved in CH_2Cl_2 (5.0 mL), and then stirred at r.t. for 2 h. After completion, the mixture was quenched with aq. $Na_2S_2O_3$ (10 mL), and washed with aq. $NaHCO_3$. The organic layer was dried over anhydrous magnesium sulfate and the solvent was removed in vacuo. The residue was purified by a column chromatography on silica gel (PE/EA= 5/1) to afford the product **49** as a white solid.

2-(naphthalen-1-yl)-4,5-diphenyl-3,6-dihydro-2*H*-1,2-thiazine 1,1-dioxide (49)

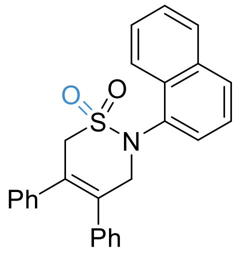

49

White solid; 41 mg, 99% yield; m.p. = 173.2-174.9 °C; ^{1}H NMR (400 MHz, $CDCl_3$) δ 8.43 (d, *J* = 8.5 Hz, 1H), 7.91 (d, *J* = 8.3 Hz, 2H), 7.77 (dd, *J* = 7.3, 0.8 Hz, 1H), 7.70-7.62 (m, 1H), 7.61-7.51 (m, 2H), 7.25-7.12 (m, 8H), 7.12-7.06 (m, 2H), 4.88 (dt, *J* = 18.0, 2.7 Hz, 1H), 4.61 (dt, *J* = 18.0, 2.2 Hz, 1H), 4.32 (dt, *J* = 17.1, 2.4 Hz, 1H), 4.25 (dt, *J* = 17.1, 2.4 Hz, 1H); ^{13}C NMR (100 MHz, $CDCl_3$) δ 139.51, 137.86, 137.62, 134.79, 134.46, 132.16, 129.20, 129.15, 129.03, 128.52, 128.49, 128.44, 128.26, 127.76, 127.70, 127.24, 126.91, 125.65, 123.81, 123.14, 59.69, 51.11; HRMS (APCI) *m/z* calcd for $C_{26}H_{21}NO_2S$ $[M+H]^+$ = 412.1371, found = 412.1366.

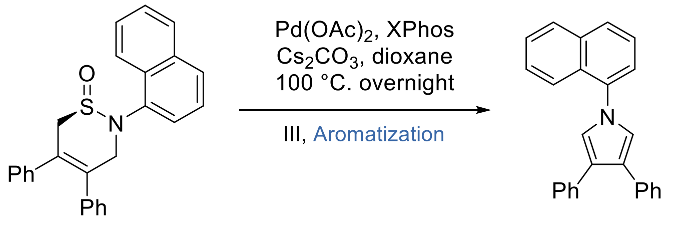

III, Aromatization: To a 25 mL Schlenk tube with a magnetic stirring bar were added the sulfinamide **1** (0.1 mmol), $Pd(OAc)_2$ (10 mol%) and XPhos (30 mol%) and Cs_2CO_3 (1.4 equiv.). The mixture was dissolved in dioxane (4.0 mL), and then the reaction was heated at 100 ℃ overnight under N_2 atmosphere. After completion, the mixture was quenched with H_2O (5 mL), and extracted with CH_2Cl_2 (10mL x 3). The organic layer was dried over anhydrous magnesium sulfate and the solvent was removed in vacuo. The residue was purified by a column chromatography on silica gel (PE/EA= 20/1) to afford the product **50** as a white solid.

1-(naphthalen-1-yl)-3,4-diphenyl-1*H*-pyrrole (50)

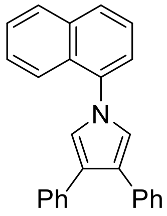

50

White solid; 30 mg, 87% yield; m.p. = 127.7-129.0 °C; ^{1}H NMR (400 MHz, $CDCl_3$) δ 8.09-8.02 (m, 1H), 8.00-7.89 (m, 2H), 7.62-7.54 (m, 4H), 7.45-7.40 (m, 4H), 7.36-7.31 (m, 4H), 7.29-7.25 (m, 2H), 7.16 (s, 2H); ^{13}C NMR (100 MHz, $CDCl_3$) δ 137.84, 135.66, 134.48, 129.59, 128.67, 128.40, 128.34, 128.17, 127.22, 126.80, 126.00, 125.49, 124.42, 123.40, 123.23, 122.71; HRMS (ESI) *m/z* calcd for $C_{26}H_{19}N$ $[M+H]^+$ = 346.1595, found = 346.1594.

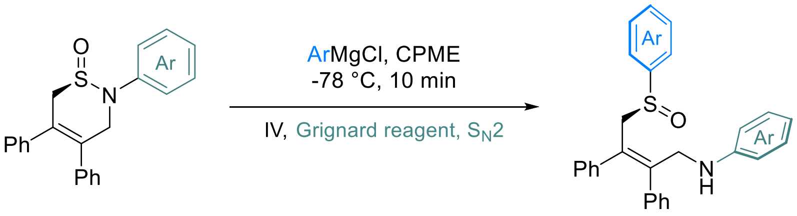

54-58

IV, S_N2 substitution: To a 25 mL Schlenk tube with a magnetic stirring bar were added the sulfinamide (0.1 mmol). The mixture was dissolved in CPME (2.0 mL), and then the reaction was moved to-78 ℃. ArMgCl (1.2 equiv.) was added dropwise under N_2 atmoephere, and the reaction was stirred at -78 ℃ for 10 min. After completion, the mixture was quenched with H_2O (5 mL), and extracted with CH_2Cl_2 (10mL x 3). The organic layer was dried over anhydrous magnesium sulfate and the solvent was removed in vacuo. The residue was purified by a column chromatography on silica gel (PE/EA= 10/1) to afford the product **54-58.**

(*S*,*Z*)-*N*-(2,3-diphenyl-4-(phenylsulfinyl)but-2-en-1-yl)naphthalen-1-amine (54)

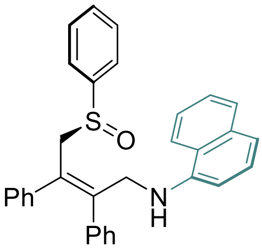

54

White solid; 45 mg, 95% yield; m.p. = 148.7-150.4 °C; ^{1}H NMR (400 MHz, $CDCl_3$) δ 8.43-8.33 (m, 1H), 7.87-7.76 (m, 1H), 7.53-7.45 (m, 4H), 7.44-7.28 (m, 5H), 7.25-7.16 (m, 7H), 7.14-7.03 (m, 3H), 6.54 (d, J = 7.3 Hz, 1H), 6.23 (s, 1H), 4.30 (d, J = 12.4 Hz, 1H), 4.20 (d, J = 12.1 Hz, 1H), 4.13 (d, J = 12.1 Hz, 1H), 3.94 (d, J = 12.4 Hz, 1H); ^{13}C NMR (100 MHz, $CDCl_3$) δ 144.13, 143.85, 143.80, 141.87, 140.08, 134.49, 132.08, 131.21, 129.83, 129.43, 128.92, 128.51, 128.28, 127.98, 127.46, 126.96, 126.60, 125.96, 124.85, 124.17, 124.02, 121.89, 117.29, 104.09, 65.33, 47.41; HRMS (APCI) m/z calcd for $C_{32}H_{27}NOS$ $[M+H]^+$ = 474.1891, found = 474.1886.

Optical Rotation: $[\alpha]^{25}_D$ = -191.40 (c = 0.50, $CHCl_3$). 94% e.e. (HPLC condition: Chiralpak ADH column, n-Hexane/i-PrOH = 70:30, flow rate = 1.0 mL/min, wavelength = 254 nm, t_R = 7.07 min for major isomer, t_R = 7.76 min for minor isomer).

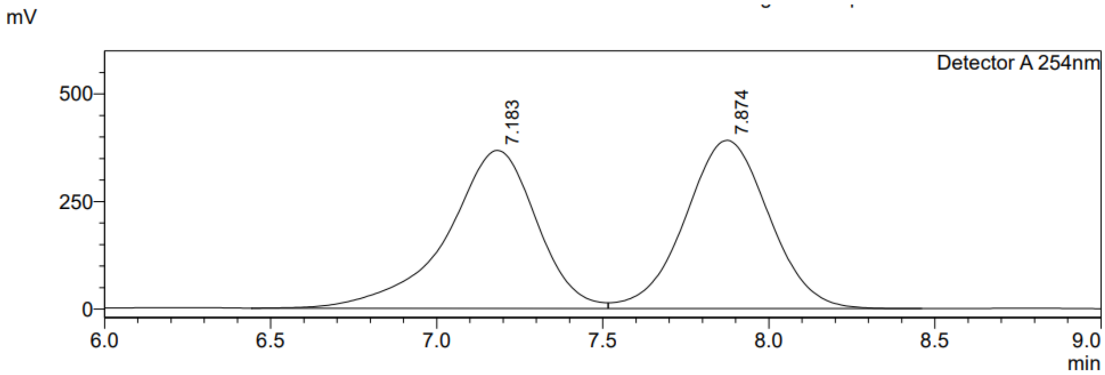

Detector A 254nm

Peak#	Ret. Time	Height	Height%	Area	Area%
1	7.183	367281	48.438	6901375	50.080
2	7.874	390966	51.562	6879463	49.920
Total		758247	100.000	13780839	100.000

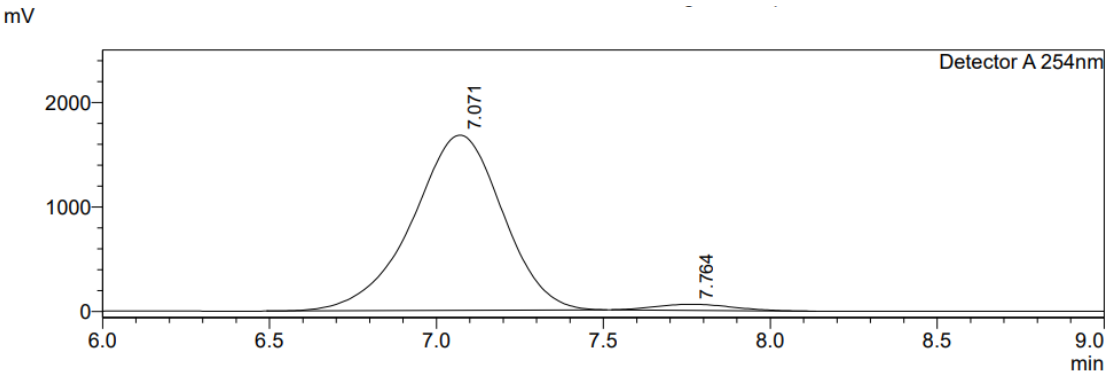

Detector A 254nm

Peak#	Ret. Time	Height	Height%	Area	Area%
1	7.071	1676425	96.653	31602004	97.189
2	7.764	58061	3.347	914037	2.811
Total		1734486	100.000	32516041	100.000

<u>(*S*,*Z*)-4-bromo-*N*-(2,3-diphenyl-4-(phenylsulfinyl)but-2-en-1-yl)naphthalen-1-amine (55)</u>

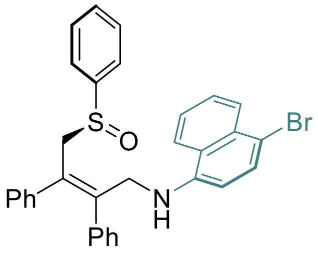

55

White solid; 48 mg, 92% yield; m.p. = 158.5-159.2 °C; ^{1}H NMR (400 MHz, $CDCl_3$) δ 8.51-8.43 (m, 1H), 8.27-8.20 (m, 1H), 7.64-7.55 (m, 3H), 7.45-7.41 (m, 2H), 7.40-7.36 (m, 1H), 7.35-7.30 (m, 2H), 7.25-7.14 (m, 7H), 7.11-7.04 (m, 3H), 6.50 (d, J = 6.7 Hz, 1H), 6.40 (d, J = 8.3 Hz, 1H), 4.26 (d, J = 12.5 Hz, 1H), 4.18 (d, J = 12.2 Hz, 1H), 4.09 (dd, J = 12.2, 7.3 Hz, 1H), 3.93 (d, J = 12.5 Hz, 1H); ^{13}C NMR (100 MHz, $CDCl_3$) δ 144.22, 143.75, 143.62, 141.88, 139.93, 132.35, 132.33, 131.26, 130.39, 129.78, 129.48, 128.82, 128.55, 128.01, 127.54, 127.43, 127.34, 127.02, 125.58, 125.47, 123.90, 122.44, 109.77, 104.73, 65.39, 47.42; HRMS (ESI) *m/z* calcd for $C_{32}H_{26}BrNOS$ $[M+H]^+$ = 522.0996, found = 522.0992.

Optical Rotation: $[\alpha]^{25}_{D}$ = -228.37 (c = 0.49, $CHCl_3$). 97% e.e. (HPLC condition: Chiralpak IC column, *n*-Hexane/*i*-PrOH = 90:10, flow rate = 1.0 mL/min, wavelength = 254 nm, t_R = 15.02 min for minor isomer, t_R = 18.04 min for major isomer).

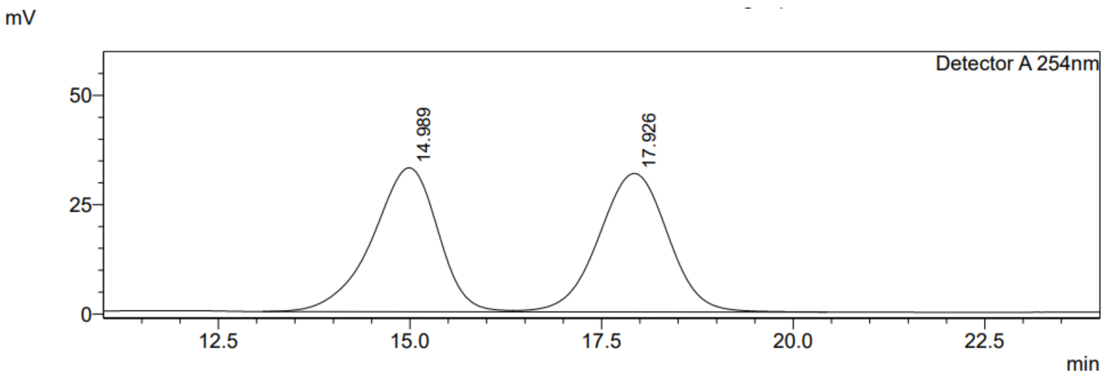

Detector A 254nm

Peak#	Ret. Time	Height	Height%	Area	Area%
1	14.989	32905	50.956	1963260	49.659
2	17.926	31670	49.044	1990237	50.341
Total		64575	100.000	3953497	100.000

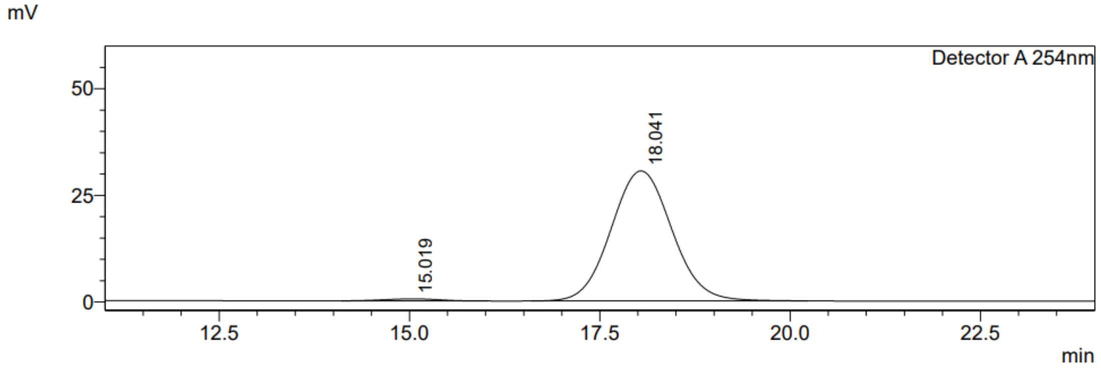

Detector A 254nm

Peak#	Ret. Time	Height	Height%	Area	Area%
1	15.019	473	1.527	23405	1.360
2	18.041	30505	98.473	1696946	98.640
Total		30978	100.000	1720351	100.000

(*S*,*Z*)-*N*-(2,3-diphenyl-4-(phenylsulfinyl)but-2-en-1-yl)phenanthren-9-amine (56)

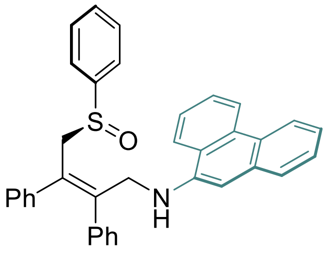

56

White solid; 45 mg, 86% yield; m.p. = 159.0-160.2 °C; ^{1}H NMR (400 MHz, $CDCl_3$) δ 8.78-8.68 (m, 1H), 8.57 (d, *J* = 8.2 Hz, 1H), 8.53-8.46 (m, 1H), 7.72-7.64 (m, 3H), 7.56-7.33 (m, 6H), 7.33-7.28 (m, 2H), 7.25-7.20 (m, 6H), 7.11-7.05 (m, 3H), 6.71 (s, 1H), 6.30 (s, 1H), 4.30 (d, *J* = 12.5 Hz, 1H), 4.29 (d, *J* = 12.0 Hz, 1H), 4.21 (d, *J* = 12.0 Hz, 1H), 3.95 (d, *J* = 12.5 Hz, 1H); ^{13}C NMR (100 MHz, $CDCl_3$) δ 143.93, 143.86, 142.10,

141.95, 140.09, 134.00, 132.22, 131.24, 131.18, 129.87, 129.47, 128.96, 128.55, 128.02, 127.51, 127.01, 126.93, 126.73, 126.55, 126.52, 126.07, 125.48, 124.00, 123.15, 122.81, 122.53, 122.37, 101.91, 65.42, 47.60; HRMS (APCI) *m/z* calcd for $C_{36}H_{29}NOS$ $[M+H]^+$ = 524.2048, found = 524.2048.

Optical Rotation: $[\alpha]^{25}_{D}$ = -131.49 (*c* = 0.43, $CHCl_3$). 94% e.e. (HPLC condition: Chiralpak ADH column, *n*-Hexane/*i*-PrOH = 70:30, flow rate = 1.0 mL/min, wavelength = 254 nm, t_R = 10.16 min for major isomer, t_R = 12.31 min for minor isomer).

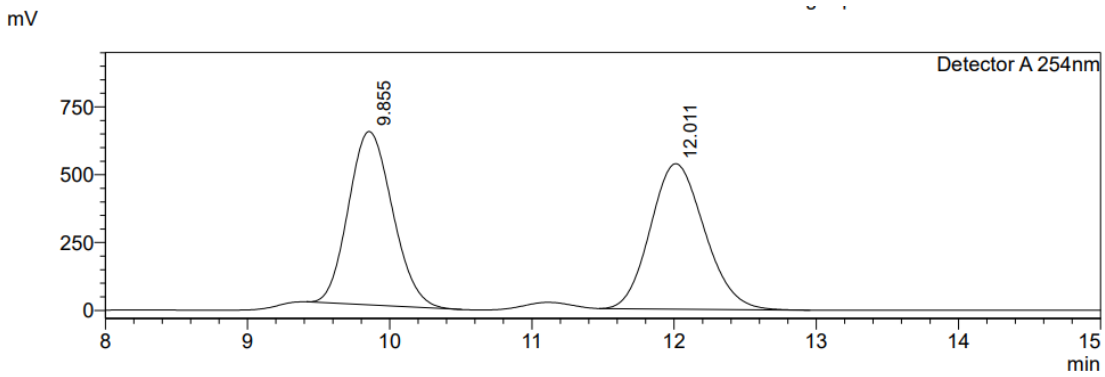

Detector A 254nm

Peak#	Ret. Time	Height	Height%	Area	Area%
1	9.855	639340	54.408	13781918	49.285
2	12.011	535735	45.592	14181767	50.715
Total		1175074	100.000	27963685	100.000

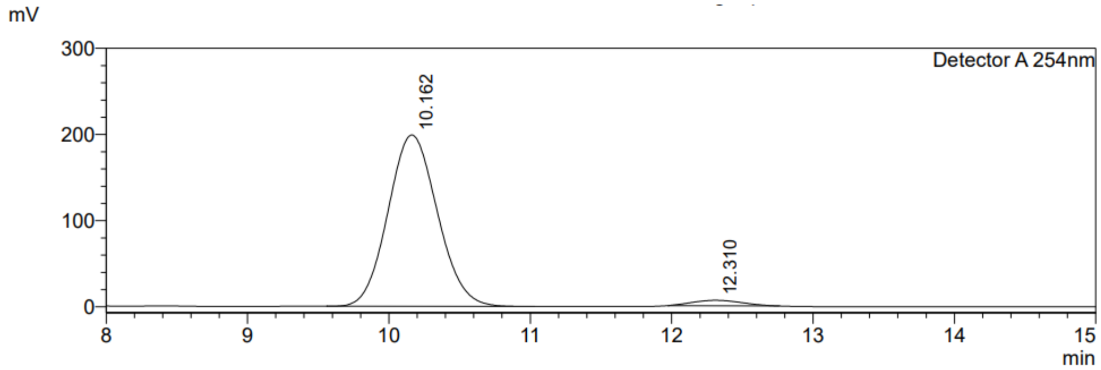

Detector A 254nm

Peak#	Ret. Time	Height	Height%	Area	Area%
1	10.162	198962	96.930	4839524	96.922
2	12.310	6301	3.070	153666	3.078
Total		205264	100.000	4993190	100.000

(*S*,*Z*)-*N*-(2,3-diphenyl-4-(*p*-tolylsulfinyl)but-2-en-1-yl)naphthalen-1-amine (57)

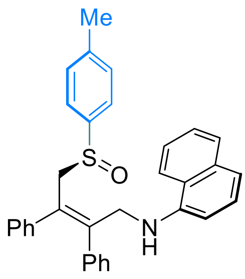

57

White solid; 43 mg, 88% yield; m.p. = 115.5-116.6 °C; ^{1}H NMR (400 MHz, $CDCl_3$) δ 8.45-8.33 (m, 1H), 7.89-7.78 (m, 1H), 7.52-7.46 (m, 2H), 7.38-7.33 (m, 3H), 7.29-7.26 (m, 1H), 7.25-7.17 (m, 7H), 7.13 (d, *J* = 7.9 Hz, 2H), 7.10-7.05 (m, 3H), 6.55 (d, *J* = 7.4 Hz, 1H), 6.23 (s, 1H), 4.29 (d, *J* = 12.5 Hz, 1H), 4.21 (d, *J* = 12.3 Hz, 1H), 4.13 (d, *J* = 12.3 Hz, 1H), 3.92 (d, *J* = 12.5 Hz, 1H), 2.32 (s, 3H); ^{13}C NMR (100 MHz, $CDCl_3$) δ 144.17, 143.67, 141.90, 141.74, 140.68, 140.18, 134.52, 132.21, 130.11, 129.83, 128.95, 128.46, 128.26, 127.97, 127.38, 126.93, 126.60, 125.94, 124.83, 124.19, 124.08, 121.93, 117.27, 104.10, 65.39, 47.46, 21.46; HRMS (ESI) *m/z* calcd for $C_{33}H_{29}NOS$ $[M+H]^+$ = 488.2048, found = 488.2043.

Optical Rotation: $[\alpha]^{25}_{D}$ = -257.31 (*c* = 0.52, $CHCl_3$). 93% e.e. (HPLC condition: Chiralpak ADH column, *n*-Hexane/*i*-PrOH = 90:10, flow rate = 1.0 mL/min, wavelength = 254 nm, t_R = 12.04 min for minor isomer, t_R = 15.25 min for major isomer).

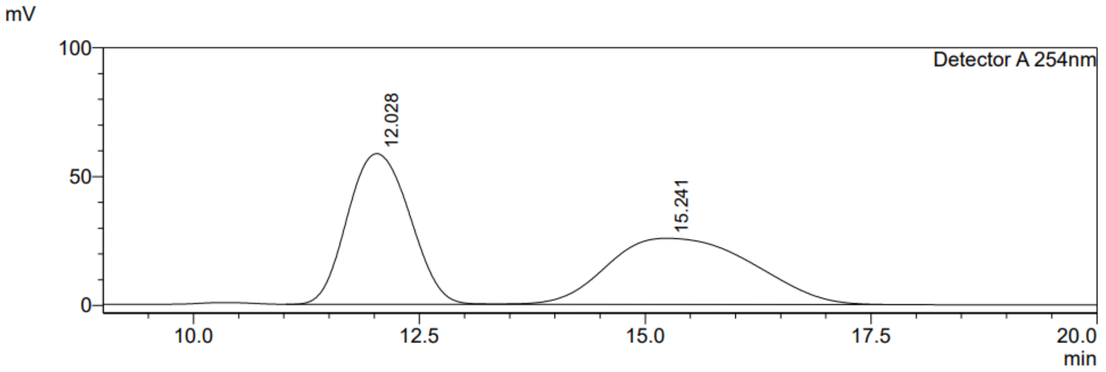

Detector A 254nm

Peak#	Ret. Time	Height	Height%	Area	Area%
1	12.028	58512	69.450	2813158	49.440
2	15.241	25739	30.550	2876858	50.560
Total		84251	100.000	5690017	100.000

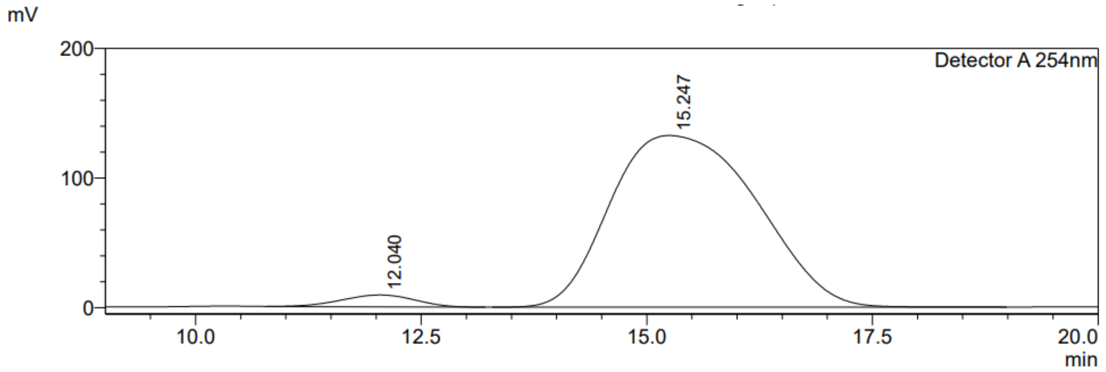

Detector A 254nm

Peak#	Ret. Time	Height	Height%	Area	Area%
1	12.040	9151	6.462	519497	3.359
2	15.247	132456	93.538	14946334	96.641
Total		141607	100.000	15465830	100.000

(*S*,*Z*)-*N*-(2,3-diphenyl-4-(o-tolylsulfinyl)but-2-en-1-yl)naphthalen-1-amine (58)

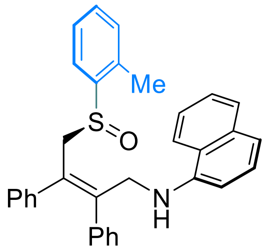

58

White solid; 46 mg, 94% yield; m.p. = 158.1-159.6 °C; ^{1}H NMR (400 MHz, $CDCl_3$) δ 8.51-8.36 (m, 1H), 7.88-7.77 (m, 1H), 7.69 (dd, *J* = 7.8, 0.9 Hz, 1H), 7.54-7.47 (m, 2H), 7.37-7.33 (m, 1H), 7.29-7.26 (m, 2H), 7.24-7.16 (m, 7H), 7.15-7.06 (m, 5H), 6.58 (d, *J* = 7.3 Hz, 1H), 6.29 (d, *J* = 5.7 Hz, 1H), 4.28-4.19 (m, 2H), 4.14 (d, *J* = 12.6 Hz, 1H), 4.00 (d, *J* = 12.6 Hz, 1H), 2.15 (s, 3H); ^{13}C NMR (100 MHz, $CDCl_3$) δ 144.25, 143.61, 142.17, 142.04, 140.18, 134.54, 134.22, 132.28, 130.86, 130.67, 129.73, 128.98, 128.46, 128.27, 127.99, 127.60, 127.40, 126.94, 126.62, 125.96, 124.86, 124.29, 123.93, 121.98, 117.29, 104.19, 63.04, 47.55, 17.98; HRMS (ESI) *m/z* calcd for $C_{33}H_{29}NOS$ $[M+H]^+$ = 488.2048, found = 488.2041.

Optical Rotation: $[\alpha]^{25}_D$ = -136.98 (*c* = 0.53, $CHCl_3$). 95% e.e. (HPLC condition: Chiralpak ODH column, *n*-Hexane/*i*-PrOH = 95:5, flow rate = 1.0 mL/min, wavelength = 254 nm, t_R = 18.92 min for major isomer, t_R = 24.93 min for minor isomer).

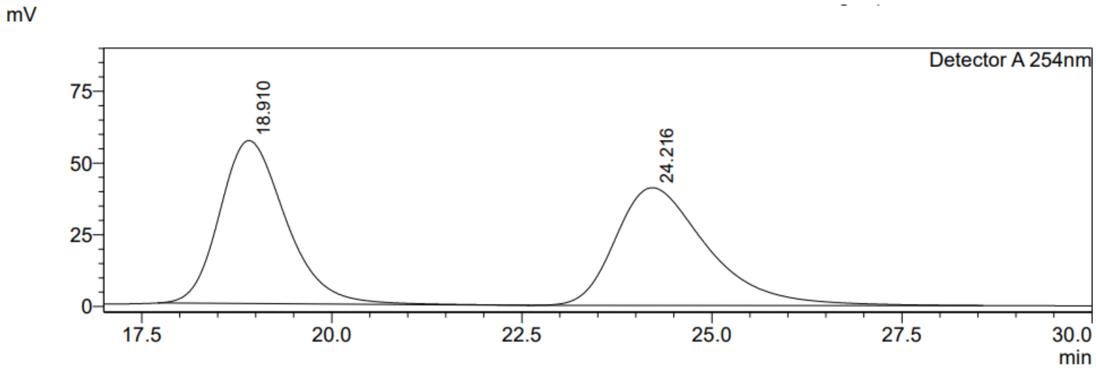

Detector A 254nm

Peak#	Ret. Time	Height	Height%	Area	Area%
1	18.910	56771	58.083	3442342	49.944
2	24.216	40971	41.917	3450096	50.056
Total		97741	100.000	6892438	100.000

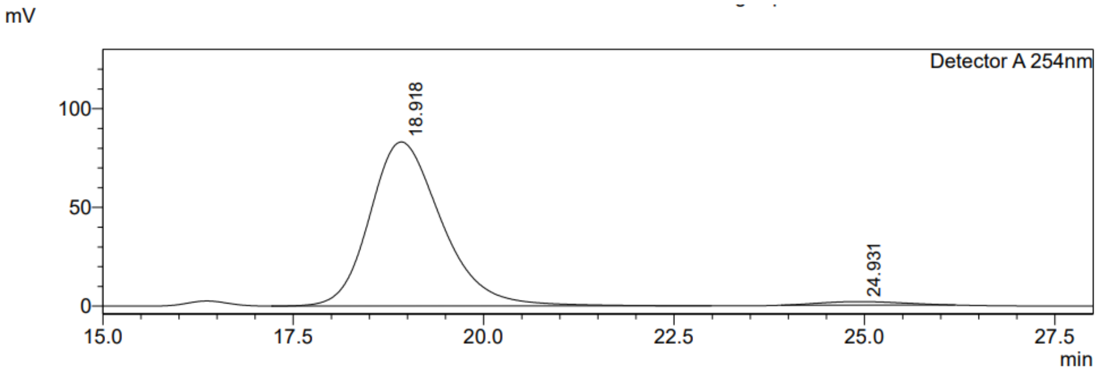

Detector A 254nm

Peak#	Ret. Time	Height	Height%	Area	Area%
1	18.918	83119	97.872	5482408	97.598
2	24.931	1808	2.128	134938	2.402
Total		84927	100.000	5617346	100.000

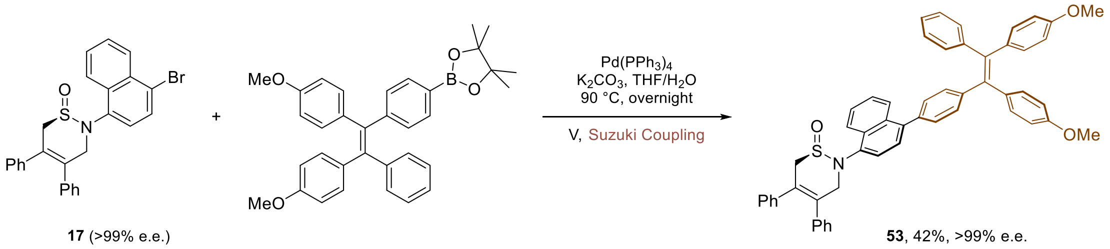

17 (>99% e.e.) **53**, 42%, >99% e.e.

V, Suzuki Coupling : To a 25 mL Schlenk tube with a magnetic stirring bar were added the sulfinamide **17** (0.1 mmol), aryl boronic acid pinacol ester (1.2 equiv.), $Pd(PPh_3)_4$ (5 mol%), K_2CO_3 (2.0 equiv.). The mixture was dissolved in THF (3.0 mL) and H_2O (1 mL), and then the reaction was heated to 90 ℃ overnight under N_2 atmosphere. After completion, the mixture was quenched with H_2O (5 mL), and extracted with CH_2Cl_2 (10mL x 3). The organic layer was dried over anhydrous magnesium sulfate and the solvent was removed in vacuo. The residue was purified by a column chromatography

on silica gel (PE/EA= 1/1) to afford the product **53.**

(*R*,*Z*)-2-(4-(4-(1,2-bis(4-methoxyphenyl)-2-phenylvinyl)phenyl)naphthalen-1-yl)-4,5-diphenyl-3,6-dihydro-2*H*-1,2-thiazine 1-oxide (53)

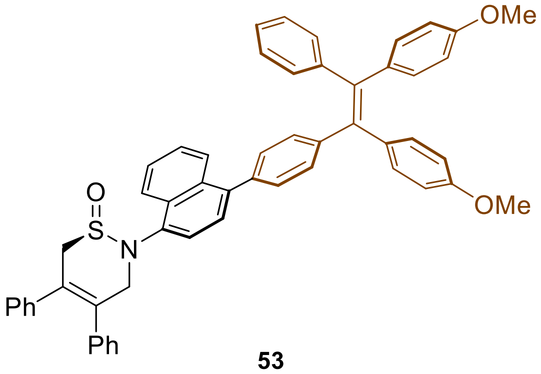

53

Yellow solid; 33 mg, 42% yield; m.p. = 97.3-98.4 °C; ^{1}H NMR (400 MHz, $CDCl_3$) δ 8.27 (d, *J* = 8.2 Hz, 1H), 7.91 (d, *J* = 8.4 Hz, 1H), 7.76 (d, *J* = 6.4 Hz, 1H), 7.63-7.59 (m, 1H), 7.53-7.48 (m, 1H), 7.42 (d, *J* = 7.6 Hz, 1H), 7.25-7.10 (m, 19H), 7.07-6.98 (m, 4H), 6.75-6.64 (m, 4H), 4.75 (d, *J* = 17.3 Hz, 1H), 4.31 (ddd, *J* = 16.7, 4.0, 2.8 Hz, 1H), 4.15 (dd, *J* = 17.3, 2.1 Hz, 1H), 3.83 (dd, *J* = 16.7, 2.1 Hz, 1H), 3.78 (s, 3H), 3.76 (s, 3H); ^{13}C NMR (100 MHz, $CDCl_3$) δ 158.36, 158.26, 144.24, 143.73, 142.01, 140.76, 140.70, 140.18, 139.15, 139.01, 137.82, 136.50, 136.36, 132.89, 132.83, 132.74, 131.58, 131.41, 130.72, 129.48, 129.37, 128.30, 128.21, 127.91, 127.35, 127.14, 127.01, 126.87, 126.64, 126.58, 126.33, 123.04, 113.16, 55.90, 55.28, 55.22, 49.45; HRMS (ESI) *m/z* calcd for $C_{54}H_{43}NO_3S$ $[M+H]^+$ = 786.3042, found = 786.3051.

Optical Rotation: $[\alpha]^{25}_{D}$ = 50.14 (*c* = 0.42, $CHCl_3$). >99% e.e. (HPLC condition: Chiralpak IG column, *n*-Hexane/*i*-PrOH = 70:30, flow rate = 1.0 mL/min, wavelength = 254 nm, t_R = 18.75 min for major isomer, t_R = 31.14 min for minor isomer).

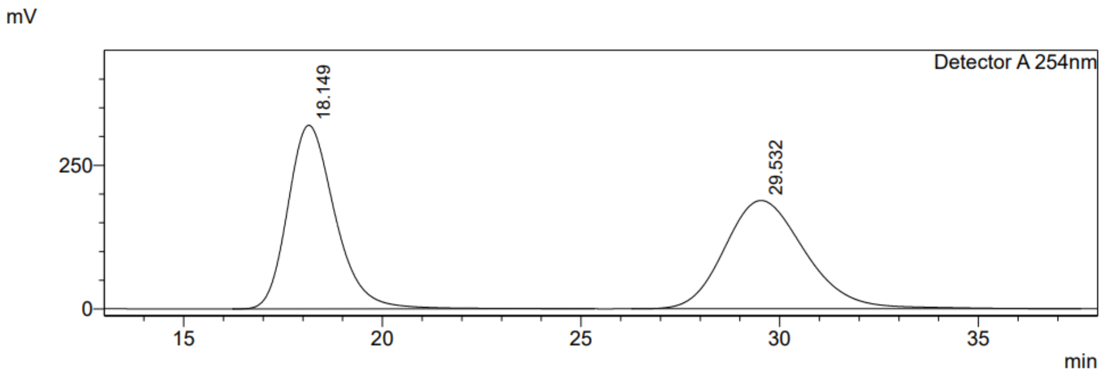

Detector A 254nm

Peak#	Ret. Time	Height	Height%	Area	Area%
1	18.149	319512	62.906	26527582	49.853
2	29.532	188405	37.094	26683844	50.147
Total		507917	100.000	53211426	100.000

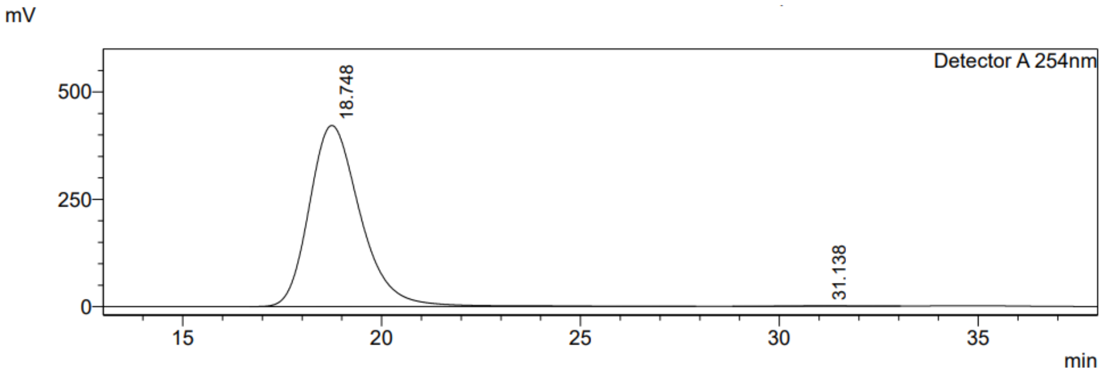

Detector A 254nm

Peak#	Ret. Time	Height	Height%	Area	Area%
1	18.748	421907	99.773	37739445	99.689
2	31.138	961	0.227	117917	0.311
Total		422868	100.000	37857362	100.000

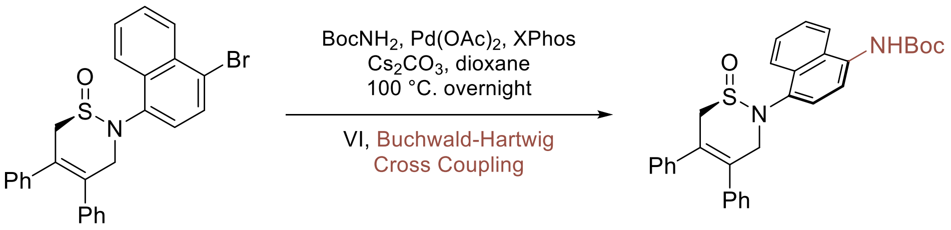

17 (>99% e.e.) **52**, 55%, >99% e.e.

VI, Buchwald-Hartwig Cross Coupling: To a 25 mL Schlenk tube with a magnetic stirring bar were added the sulfinamide **17** (0.1 mmol), $BocNH_2$ (1.5 equiv.), $Pd(OAc)_2$ (10 mol%) and XPhos (30 mol%) and Cs_2CO_3 (1.4 equiv.). The mixture was dissolved in dioxane (4.0 mL), and then the reaction was heated at 100 ℃ overnight under N_2 atmosphere. After completion, the mixture was quenched with H_2O (5 mL), and extracted with CH_2Cl_2 (10mL x 3). The organic layer was dried over anhydrous

magnesium sulfate and the solvent was removed in vacuo. The residue was purified by a column chromatography on silica gel (PE/EA= 20/1) to afford the product **52** as a light yellow solid.

tert-butyl(*R*)-(4-(1-oxido-4,5-diphenyl-3,6-dihydro-2H-1,2-thiazin-2-yl)naphthalen-1-yl)carbamate (52)

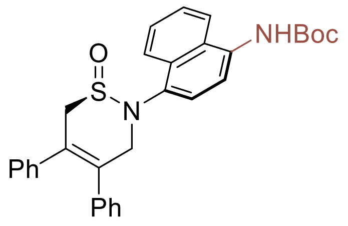

52

Yellow solid; 28 mg, 55% yield; m.p. = 110.8-112.3 °C; ^{1}H NMR (400 MHz, $CDCl_3$) δ 8.23 (d, *J* = 4.9 Hz, 1H), 7.99-7.92 (m, 1H), 7.89 (d, *J* = 7.7 Hz, 1H), 7.72 (s, 1H), 7.65-7.57 (m, 2H), 7.21-7.08 (m, 10H), 6.91 (s, 1H), 4.69 (d, *J* = 17.3 Hz, 1H), 4.30-4.23 (m, 1H), 4.05 (dd, *J* = 17.3, 1.8 Hz, 1H), 3.79 (dd, *J* = 16.7, 1.9 Hz, 1H), 1.56 (s, 9H); ^{13}C NMR (100 MHz, $CDCl_3$) δ 153.50, 140.79, 139.12, 132.88, 131.15, 129.35, 129.33, 128.29, 128.22, 127.35, 127.12, 126.90, 126.77, 123.66, 121.39, 81.11, 55.94, 49.59, 28.50; HRMS (ESI) *m/z* calcd for $C_{31}H_{30}N_2O_3S$ $[M+H]^+$ = 511.2055, found = 511.2050.

Optical Rotation: $[\alpha]^{25}_{D}$ = 135.31 (*c* = 0.49, $CHCl_3$). >99% e.e. (HPLC condition: Chiralpak IF column, *n*-Hexane/*i*-PrOH = 70:30, flow rate = 1.0 mL/min, wavelength = 254 nm, t_R = 11.12 min for major isomer, t_R = 13.30 min for minor isomer).

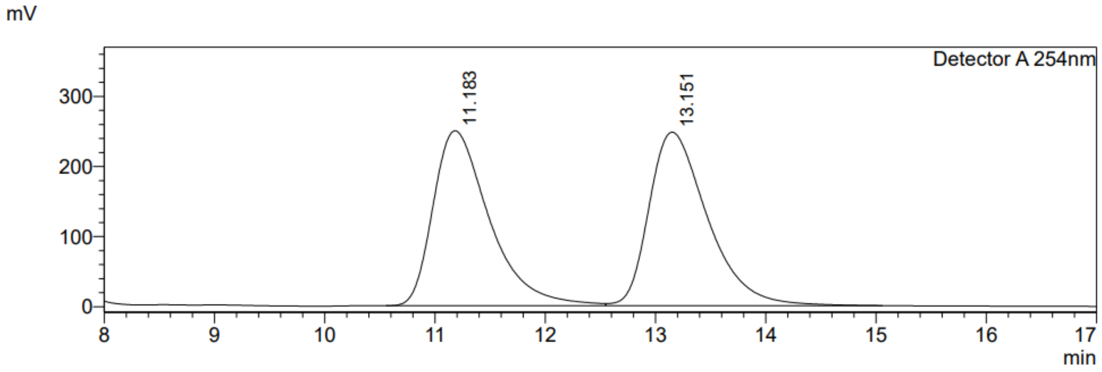

Detector A 254nm

Peak#	Ret. Time	Height	Height%	Area	Area%
1	11.183	249501	50.217	8879129	49.324
2	13.151	247348	49.783	9122467	50.676
Total		496849	100.000	18001597	100.000

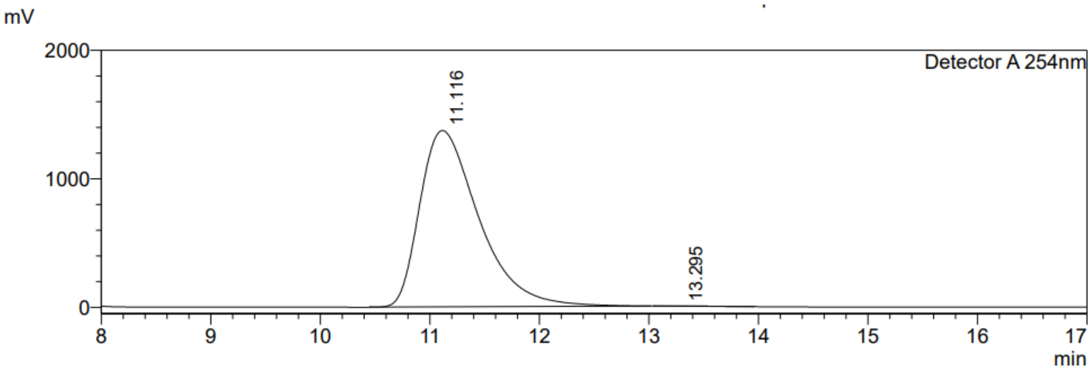

Detector A 254nm

Peak#	Ret. Time	Height	Height%	Area	Area%
1	11.116	1373261	99.824	51559086	99.873
2	13.295	2416	0.176	65566	0.127
Total		1375677	100.000	51624652	100.000

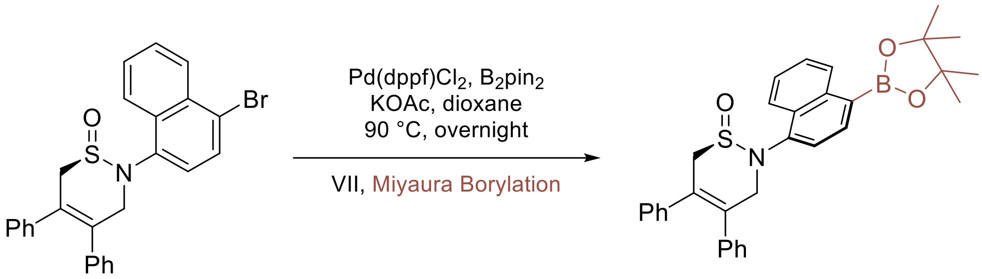

17 (>99% e.e.) **51**, 99%, >99% e.e.

VII, Miyaura Borylation : To a 25 mL Schlenk tube with a magnetic stirring bar were added the sulfinamide **17** (0.1 mmol), Pd(dppf)Cl_2 (10 mol%) and B_2pin_2 (4.0 equiv.) and KOAc (5.0 equiv.). The mixture was dissolved in dioxane (4.0 mL), and then the reaction was heated at 90 ℃ overnight under N_2 atmosphere. After completion, the mixture was quenched with H_2O (5 mL), and extracted with CH_2Cl_2 (10mL x 3). The organic layer was dried over anhydrous magnesium sulfate and the solvent was removed in vacuo. The residue was purified by a column chromatography on silica gel (PE/EA= 5/1) to afford the product **51**.

(*R*)-4,5-diphenyl-2-(4-(4,4,5,5-tetramethyl-1,3,2-dioxaborolan-2-yl)naphthalen-1-yl)-3,6-dihydro-2*H*-1,2-thiazine 1-oxide (51)

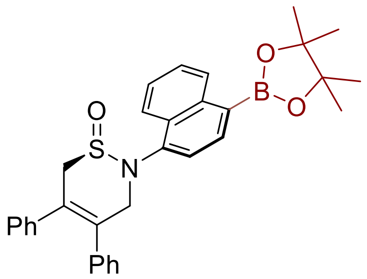

51

Light yellow oil; 52 mg, 99% yield; ^{1}H NMR (400 MHz, $CDCl_3$) δ 8.87 (dd, *J* = 5.4, 4.0 Hz, 1H), 8.23 (d, *J* = 5.4 Hz, 1H), 8.11 (d, *J* = 7.4 Hz, 1H), 7.70 (d, *J* = 6.9 Hz, 1H), 7.67-7.56 (m, 2H), 7.24-7.10 (m, 10H), 4.73 (d, *J* = 17.2 Hz, 1H), 4.30 (d, *J* = 16.7 Hz, 1H), 4.12 (d, *J* = 17.2 Hz, 1H), 3.80 (d, *J* = 16.7 Hz, 1H), 1.44 (s, 12H); ^{13}C NMR (100 MHz, $CDCl_3$) δ 145.63, 140.73, 139.13, 138.40, 135.94, 134.26, 129.97, 129.35, 129.27, 128.28, 128.21, 127.34, 127.12, 127.03, 126.40, 124.36, 122.92, 121.71, 84.01, 55.76, 49.05, 25.09, 25.05; HRMS (ESI) *m/z* calcd for $C_{32}H_{32}BNOS_3$ $[M+H]^+$ = 522.2274, found = 522.2278.

Optical Rotation: $[\alpha]^{25}_D$ = 1.52 (*c* = 0.50, $CHCl_3$). >99% e.e. (HPLC condition: Chiralpak IE column, *n*-Hexane/*i*-PrOH = 70:30, flow rate = 1.0 mL/min, wavelength = 254 nm, t_R = 11.67 min for major isomer, t_R = 13.69 min for minor isomer).

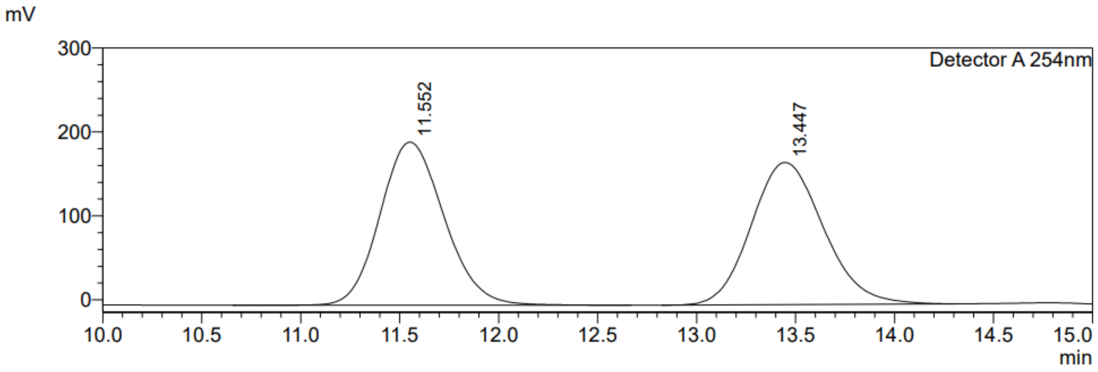

Detector A 254nm

Peak#	Ret. Time	Height	Height%	Area	Area%
1	11.552	194310	53.427	4388745	50.429
2	13.447	169382	46.573	4314013	49.571
Total		363692	100.000	8702759	100.000

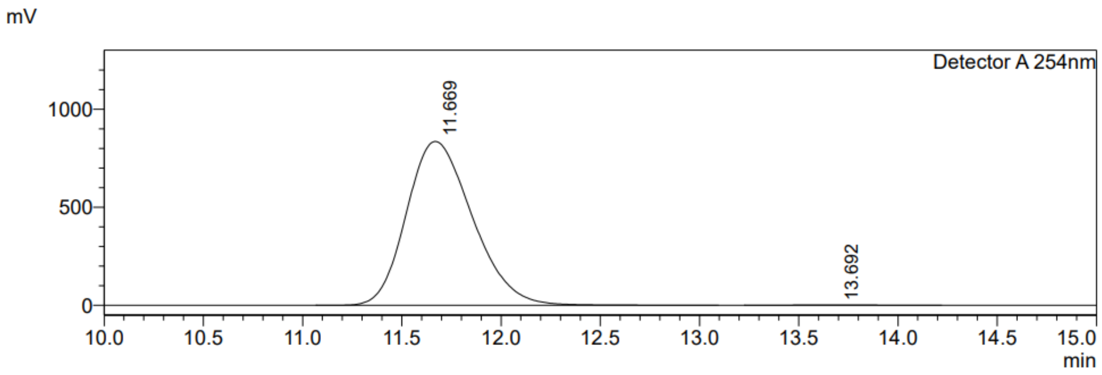

Detector A 254nm

Peak#	Ret. Time	Height	Height%	Area	Area%
1	11.669	835052	99.786	19340134	99.777
2	13.692	1787	0.214	43209	0.223
Total		836839	100.000	19383343	100.000

8. X-Ray Data of Chiral Compounds

(1) The absolute configurations of catalyst **P6** were displayed by X-ray crystallographic analysis of a single crystal (**Supplementary Fig. 6**). CCDC 2115196, contains the supplementary crystallographic data for this paper. This data can be obtained free of charge from The Cambridge Crystallographic Data Centre via www.ccdc.cam.ac.uk/data_request/cif.

(2) The absolute configurations of products **1-47** were assigned as *R* by X-ray crystallographic analysis of a single crystal of enantiopure **12, 16, 20, 41, 42** (**Supplementary Fig. 7-11**). CCDC 2235143, CCDC 2210348, CCDC 2213077, CCDC 2262526, CCDC 2265808 contains the supplementary crystallographic data for this paper. These data can be obtained free of charge from The Cambridge Crystallographic Data Centre via www.ccdc.cam.ac.uk/data_request/cif.

(3) The absolute configurations of the amination product **48** were assigned as *S* by X-ray crystallographic analysis of a single crystal of enantiopure **48** (**Supplementary Fig. 12**). CCDC 2244448, contains the supplementary crystallographic data for this paper. This data can be obtained free of charge from The Cambridge Crystallographic Data Centre via www.ccdc.cam.ac.uk/data_request/cif.

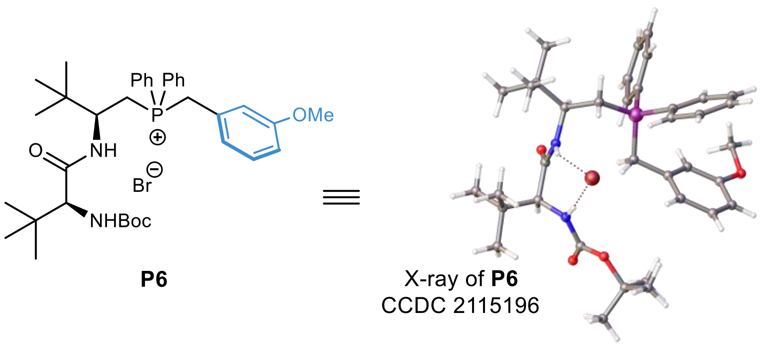

Supplementary Fig. 6. X-ray structure of catalyst **P6**.

Supplementary Table 9. Crystal data and structure refinement for **P6**.

Identification code	**P6**
Empirical formula	$C_{37}H_{52}BrN_2O_4P$
Formula weight	699.68
Temperature/K	150.0
Crystal system	triclinic
Space group	P1
a/Å	11.810(2)
b/Å	14.819(3)
c/Å	15.001(3)
α/°	113.633(8)
β/°	92.252(7)
γ/°	113.189(7)
Volume/Å^3	2149.6(8)
Z	2
ρ_{calc}g/cm^3	1.081
μ/mm^{-1}	1.027
F(000)	740.0
Crystal size/mm^3	0.32 × 0.12 × 0.04
Radiation	MoKα (λ = 0.71073)
2Θ range for data collection/°	3.816 to 50
Index ranges	$-14 \leq h \leq 14$, $-17 \leq k \leq 17$, $-17 \leq l \leq 17$
Reflections collected	47055
Independent reflections	15064 [R_{int} = 0.0889, R_{sigma} = 0.1013]
Data/restraints/parameters	15064/3/831
Goodness-of-fit on F^2	0.952
Final R indexes [I>=2σ (I)]	R_1 = 0.0428, wR_2 = 0.0920
Final R indexes [all data]	R_1 = 0.0572, wR_2 = 0.0993
Largest diff. peak/hole / e Å^{-3}	0.36/-0.25
Flack parameter	0.036(6)

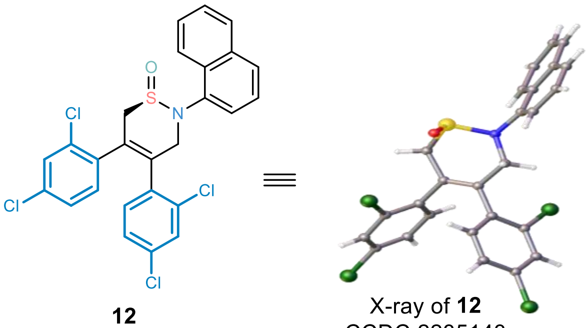

12

X-ray of **12**
CCDC 2235143

Supplementary Fig. 7. X-ray structure of compound **12**.

Supplementary Table 10. Crystal data and structure refinement for compound **12**.

Identification code	**12**
Empirical formula	$C_{26}H_{17}Cl_4NOS$
Formula weight	533.27
Temperature/K	156.0
Crystal system	tetragonal
Space group	$P4_12_12$
a/Å	9.6781(4)
b/Å	9.6781(4)
c/Å	50.076(3)
α/°	90
β/°	90
γ/°	90
Volume/Å^3	4690.4(5)
Z	8
ρ_{calc}g/cm^3	1.510
μ/mm^{-1}	0.615
F(000)	2176.0
Crystal size/mm^3	0.39 × 0.12 × 0.08
Radiation	MoKα (λ = 0.71073)
2Θ range for data collection/°	4.286 to 55.09
Index ranges	$-12 \le h \le 12$, $-10 \le k \le 12$, $-65 \le l \le 65$
Reflections collected	33818
Independent reflections	5416 [R_{int} = 0.1085, R_{sigma} = 0.0654]
Data/restraints/parameters	5416/0/298
Goodness-of-fit on F^2	1.049
Final R indexes [I>=2σ (I)]	R_1 = 0.0566, wR_2 = 0.1044
Final R indexes [all data]	R_1 = 0.0864, wR_2 = 0.1166
Largest diff. peak/hole / e Å^{-3}	0.40/-0.33
Flack parameter	0.03(6)

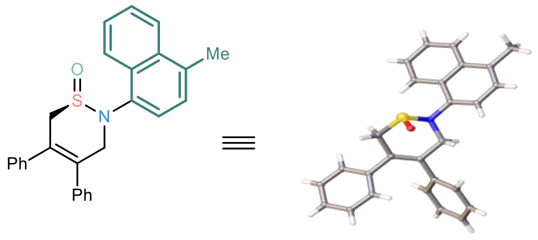

16 X-ray of **16**
CCDC 2210348

Supplementary Fig. 8. X-ray structure of compound **12**.

Supplementary Table 11. Crystal data and structure refinement for compound **12**.

Identification code	**12**
Empirical formula	$C_{27}H_{23}NOS$
Formula weight	409.52
Temperature/K	256.0
Crystal system	monoclinic
Space group	$P2_1$
a/Å	8.5176(6)
b/Å	10.5650(6)
c/Å	12.0057(8)
α/°	90
β/°	96.097(3)
γ/°	90
Volume/Å^3	1074.26(12)
Z	2
ρ_{calc}g/cm^3	1.266
μ/mm^{-1}	0.169
F(000)	432.0
Crystal size/mm^3	0.45 × 0.27 × 0.09
Radiation	MoKα (λ = 0.71073)
2Θ range for data collection/°	4.81 to 55.074
Index ranges	$-10 \le h \le 11, -13 \le k \le 13, -15 \le l \le 14$
Reflections collected	13544
Independent reflections	4913 [R_{int} = 0.0529, R_{sigma} = 0.0601]
Data/restraints/parameters	4913/1/272
Goodness-of-fit on F^2	1.052
Final R indexes [I>=2σ (I)]	R_1 = 0.0450, wR_2 = 0.0939
Final R indexes [all data]	R_1 = 0.0590, wR_2 = 0.1023
Largest diff. peak/hole / e Å^{-3}	0.15/-0.20
Flack parameter	0.03(5)

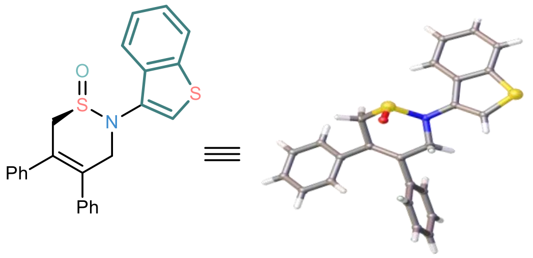

20

X-ray of **20**
CCDC 2213077

Supplementary Fig. 9. X-ray structure of compound **20**.

Supplementary Table 12. Crystal data and structure refinement for compound **20**.

Identification code	**20**
Empirical formula	$C_{24}H_{19}NOS_2$
Formula weight	401.52
Temperature/K	192.0
Crystal system	monoclinic
Space group	C2
a/Å	31.2777(7)
b/Å	5.81410(10)
c/Å	10.8231(3)
α/°	90
β/°	92.7200(10)
γ/°	90
Volume/Å^3	1965.98(8)
Z	4
ρ_{calc}g/cm^3	1.357
μ/mm^{-1}	0.286
F(000)	840.0
Crystal size/mm^3	0.48 × 0.12 × 0.11
Radiation	MoKα (λ = 0.71073)
2Θ range for data collection/°	4.48 to 54.998
Index ranges	$-40 \leq h \leq 40$, $-7 \leq k \leq 7$, $-14 \leq l \leq 14$
Reflections collected	27192
Independent reflections	4504 [R_{int} = 0.0483, R_{sigma} = 0.0308]
Data/restraints/parameters	4504/1/253
Goodness-of-fit on F^2	1.064
Final R indexes [I>=2σ (I)]	R_1 = 0.0297, wR_2 = 0.0704
Final R indexes [all data]	R_1 = 0.0340, wR_2 = 0.0730
Largest diff. peak/hole / e Å^{-3}	0.21/-0.28
Flack parameter	-0.01(2)

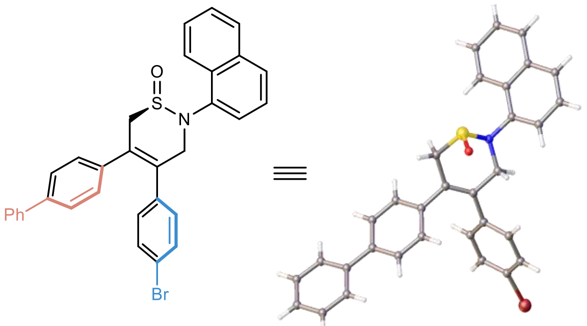

Supplementary Fig. 10. X-ray structure of compound **41**.

Supplementary Table 13. Crystal data and structure refinement for compound **41**.

Identification code	**41**
Empirical formula	$C_{32}H_{24}BrNOS$
Formula weight	550.49
Temperature/K	150.0
Crystal system	monoclinic
Space group	$P2_1$
a/Å	13.433(4)
b/Å	5.5074(15)
c/Å	17.022(6)
α/°	90
β/°	91.707(14)
γ/°	90
Volume/Å^3	1258.7(7)
Z	2
ρ_{calc}g/cm^3	1.452
μ/mm^{-1}	1.743
F(000)	564.0
Crystal size/mm^3	0.43 × 0.07 × 0.03
Radiation	MoKα (λ = 0.71073)
2Θ range for data collection/ °	3.808 to 54.942
Index ranges	-17 ≤ h ≤ 17, -6 ≤ k ≤ 7, -22 ≤ l ≤ 21
Reflections collected	11962
Independent reflections	5598 [R_{int} = 0.0584, R_{sigma} t= 0.1007]
Data/restraints/parameters	5598/1/325
Goodness-of-fit on F^2	0.893
Final R indexes [I>=2σ (I)]	R_1 = 0.0406, wR_2 = 0.0779
Final R indexes [all data]	R_1 = 0.0669, wR_2 = 0.0897
Largest diff. peak/hole / e Å^{-3}	0.29/-0.36
Flack parameter	0.023(8)

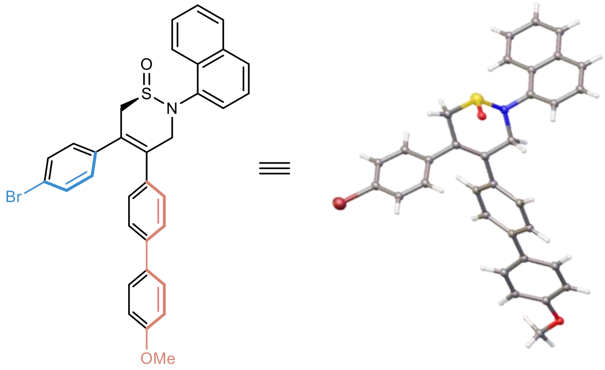

42

X-ray of **42**
CCDC 2265808

Supplementary Fig. 11. X-ray structure of compound **42**.

Supplementary Table 14. Crystal data and structure refinement for compound **42**.

Identification code	**42**
Empirical formula	$C_{33}H_{26}BrNO_2S$
Formula weight	580.52
Temperature/K	241.0
Crystal system	orthorhombic
Space group	$P2_12_12_1$
a/Å	5.5518(8)
b/Å	13.212(2)
c/Å	36.922(6)
α/°	90
β/°	90
γ/°	90
Volume/Å^3	2708.3(8)
Z	4
ρ_{calc}g/cm^3	1.424
μ/mm^{-1}	1.626
F(000)	1192.0
Crystal size/mm^3	0.48 × 0.25 × 0.13
Radiation	MoKα (λ = 0.71073)
2Θ range for data collection/°	4.412 to 54.666
Index ranges	$-6 \leq h \leq 6$, $-17 \leq k \leq 14$, $-47 \leq l \leq 30$
Reflections collected	15242
Independent reflections	5821 [R_{int} = 0.0466, R_{sigma} = 0.0882]
Data/restraints/parameters	5821/0/344
Goodness-of-fit on F^2	0.966
Final R indexes [I>=2σ (I)]	R_1 = 0.0403, wR_2 = 0.0740
Final R indexes [all data]	R_1 = 0.0852, wR_2 = 0.0871
Largest diff. peak/hole / e Å^{-3}	0.29/-0.41

Flack parameter	0.009(7)

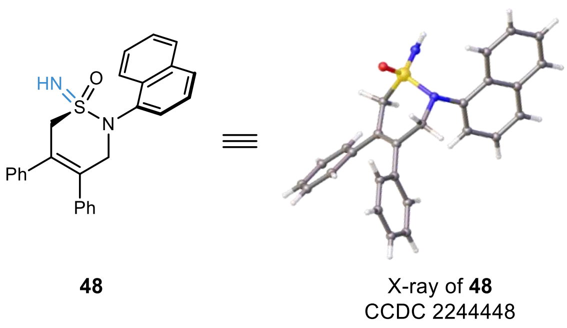

Supplementary Fig. 12. X-ray structure of compound **48**.

Supplementary Table 15. Crystal data and structure refinement for compound **48**.

Identification code	**48**
Empirical formula	$C_{26}H_{22}N_2OS$
Formula weight	410.51
Temperature/K	182.0
Crystal system	orthorhombic
Space group	$P2_12_12_1$
a/Å	5.7902(4)
b/Å	11.0819(8)
c/Å	31.634(2)
α/°	90
β/°	90
γ/°	90
Volume/Å^3	2029.9(2)
Z	4
ρ_{calc}g/cm^3	1.343
μ/mm^{-1}	0.181
F(000)	864.0
Crystal size/mm^3	0.43 × 0.05 × 0.05
Radiation	MoKα (λ = 0.71073)
2Θ range for data collection/°	3.894 to 55.046
Index ranges	$-7 \leq h \leq 7, -14 \leq k \leq 14, -35 \leq l \leq 40$
Reflections collected	18298
Independent reflections	4586 [R_{int} = 0.0859, R_{sigma} = 0.0746]
Data/restraints/parameters	4586/0/274
Goodness-of-fit on F^2	1.045
Final R indexes [I>=2σ (I)]	R_1 = 0.0527, wR_2 = 0.1035
Final R indexes [all data]	R_1 = 0.0719, wR_2 = 0.1130
Largest diff. peak/hole / e Å^{-3}	0.40/-0.33

Flack parameter	0.05(6)

9. Mechanistic Investigations

Identification of the Key Intermediate

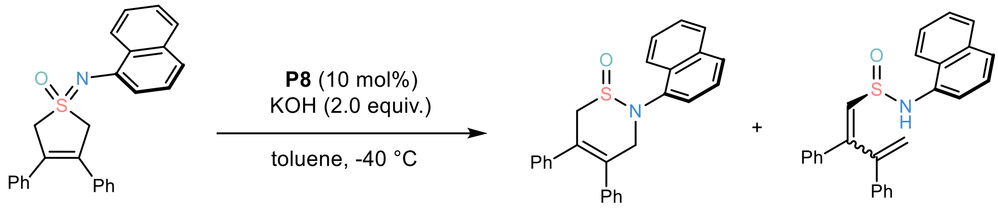

A5 **1** **B**

To a tube (20 mL) with a magnetic stirring bar, the sulfoximine **A5** (0.20 mmol), catalyst **P8** (10 mol%) and KOH (2.0 equiv.) were added, followed by the addition of toluene (5.0 mL). The reaction mixture was stirred at -40 °C. The progress of reaction was monitored by TLC. Purification by column chromatography on silica gel (petroleum ether/ethyl acetate = 2:1) at -40 ℃ afforded the key intermediate **B**.

<u>(*R*,*Z*)-*N*-(naphthalen-1-yl)-2,3-diphenylbuta-1,3-diene-1-sulfinamide (B)</u>

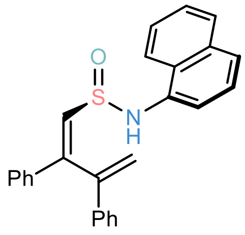

B

Yellow solid; 10 mg, 13% yield; m.p. = 141.6-142.9 °C; ^{1}H NMR (400 MHz, DMSO-*d6*) δ 9.42 (s, 1H), 8.26-8.12 (m, 1H), 8.00-7.85 (m, 1H), 7.66-7.60 (m, 2H), 7.56-7.48 (m, 4H), 7.42-7.36 (m, 5H), 7.32 (d, *J* = 7.3 Hz, 1H), 7.28-7.20 (m, 3H), 6.14 (s, 1H), 5.43 (s, 1H), 3.43 (s, 1H); ^{13}C NMR (100 MHz, DMSO-*d6*) δ 146.17, 143.19, 137.29, 137.29, 136.34, 135.55, 134.03, 129.54, 128.93, 128.65, 128.29, 128.17, 126.94, 126.56, 126.30, 126.27, 125.98, 125.64, 123.45, 122.51, 118.84, 115.62.; HRMS (ESI) *m/z* calcd for $C_{26}H_{21}NOS$ $[M+Na]^+$ = 418.1242, found = 418.1239.

Time-Course Study

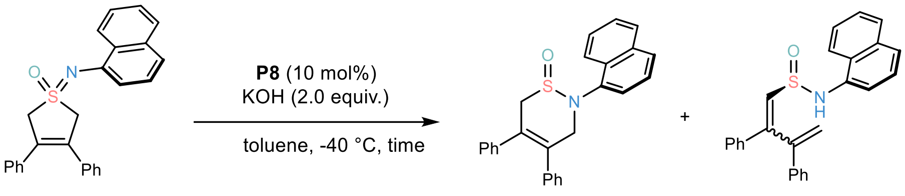

A5 1 B

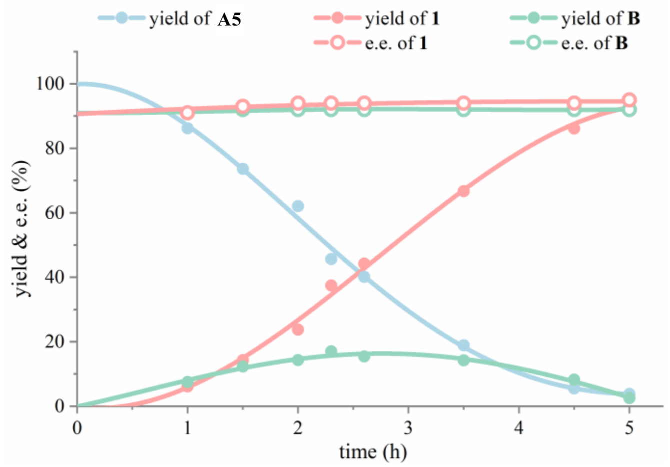

Supplementary Fig. 13. Monitoring the reaction process over time.

To a tube (20 mL) with a magnetic stirring bar, the sulfoximine **A5** (0.10 mmol), catalyst **P8** (10 mol%) and KOH (2.0 equiv.) were added, followed by the addition of toluene (5.0 mL). The reaction mixture was stirred at -40 °C. The progress of reaction was monitored by TLC. The e.e. values was determined by HPLC analysis. The yield was determined by ^{1}H NMR analysis.

Control Experiments

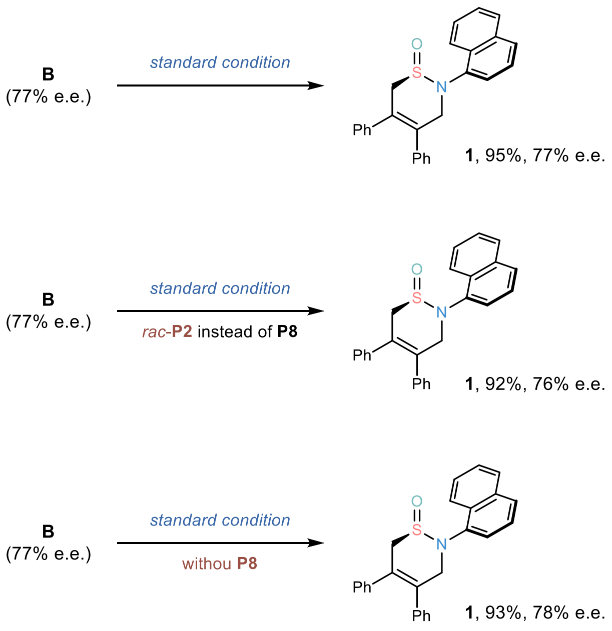

Supplementary Fig. 14. Control experiments.

First, the intermediate **B** with 77% e.e. was prepared for the control experiments. Next, to a tube (20 mL) with a magnetic stirring bar, the intermediate **B** (0.10 mmol), catalyst (10 mol%) and KOH (2.0 equiv.) were added, followed by the addition of toluene (1.0 mL). The reaction mixture was stirred at -40 ^{o}C. The progress of reaction was monitored by TLC. The e.e. values was determined by HPLC analysis. Isolated yield was reported.

Investigation of Nonlinear Effect

Supplementary Table 16. Enantioselective skeletal reorganization catalyzed by **P8**[a,b]

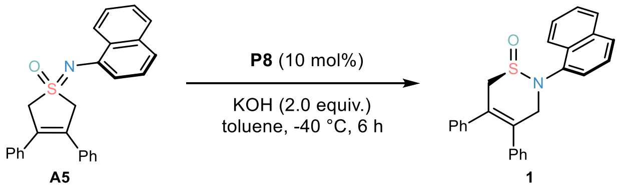

ee of **P8** (%)	ee of **1** (%)
0	0
24	18
44	36

59	54
82	74
100	95

[a] Reaction conditions: **A5** (0.1 mmol), KOH (2.0 equiv.) and **P8** (10 mol%) in toluene (1.0 mL) at -40 °C for 6 h. [b] All ee values were determined by HPLC analysis on a chiral stationary phase.

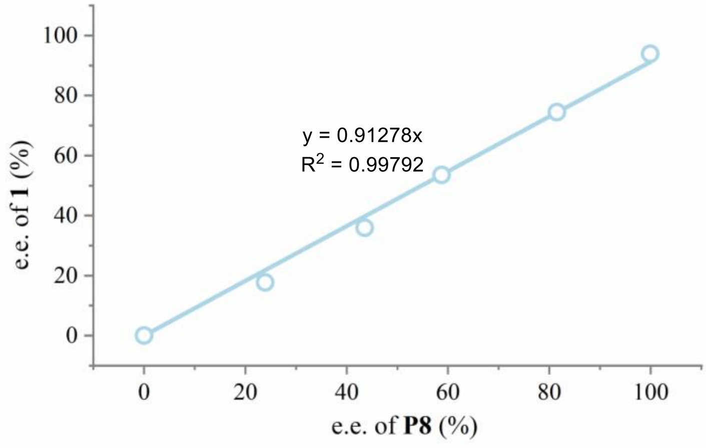

Supplementary Fig. 15. Nonlinear Effect.

^{1}H NMR Titration Experiments

^{1}H NMR titration was conducted in $CDCl_3$. The total concentration of the host (**P8**) and the guest (**A5**) was 0.08 M. The proportion of the concentration of the host vs the total concentration varied from 0.1 equivalent to 1.0. A ^{1}H NMR spectrum was recorded after each addition. ^{1}H NMR spectra were calibrated to the residual $CDCl_3$ solvent peak.

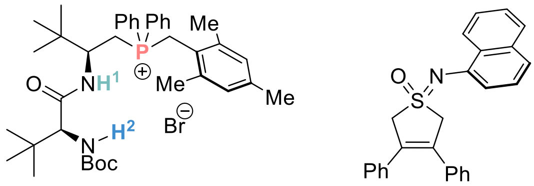

P8 **A5**

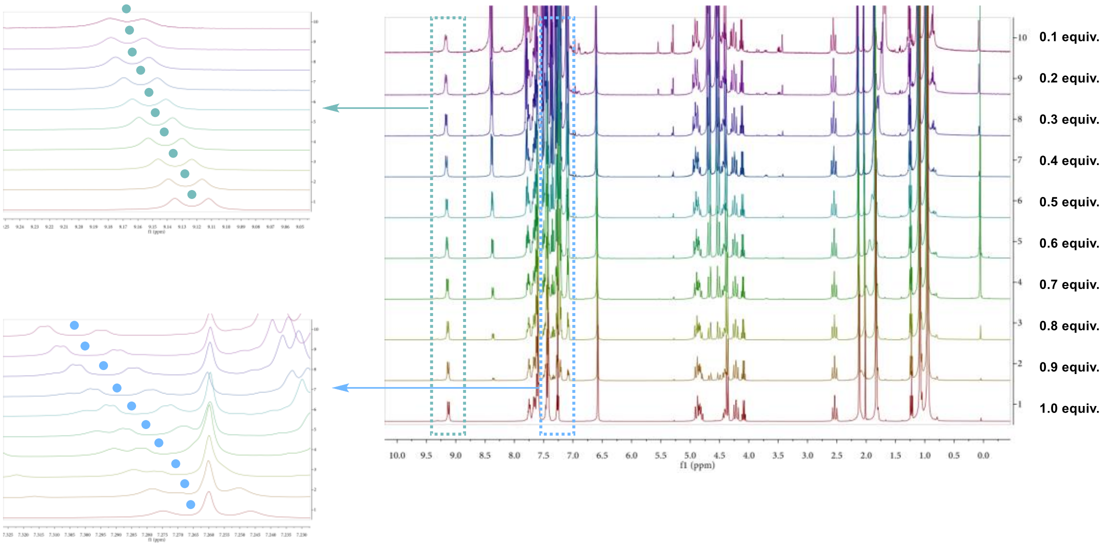

Supplementary Fig. 16. ^{1}H NMR spectrum of P**8** with **A5**

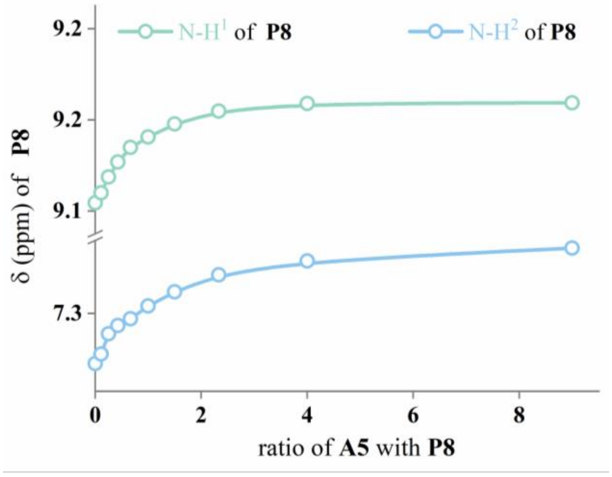

Supplementary Fig. 17. ^{1}H NMR titration of **A5** with **P8.**

As shown in Supplementary Fig. 17, ^{1}H NMR titration of substrate **A5** to catalyst **P8** led to obvious changes in the positions of both NH^{1} and NH^{2} of the catalyst. Then, we undertook the Job plot analyses, which suggests a 1 : 1 binding pattern between catalyst **P8** and **A5**.

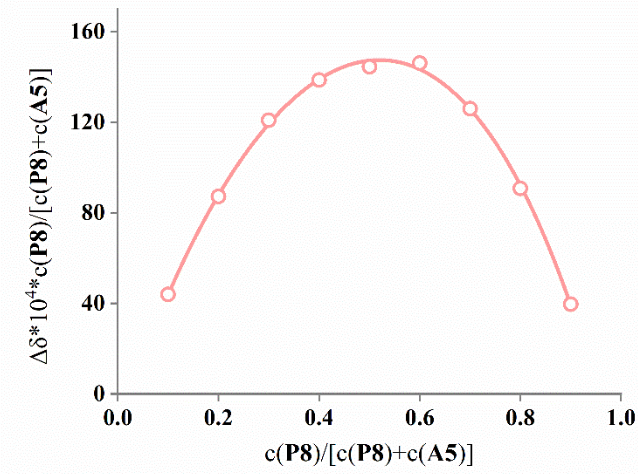

Supplementary Fig. 18. Job plot analyses

Kinetic Study of Catalyst P8

2 mM/[P8] concentration: To a tube (20 mL) with a magnetic stirring bar, the sulfoximine **A5** (0.10 mmol), catalyst **P8** (10 mol%) and KOH (0.20 mmol) were added, followed by the addition of toluene (5.0 mL). The reaction mixture was stirred at -40 ^{o}C. After stirring for the indicated period, small amount of reaction mixture was sampled via syringe, which was directly used for NMR analysis to measure the conversion yield.

3 mM/[P8] concentration: **A5** (0.10 mmol), catalyst **P8** (15 mol%), KOH (0.20 mmol) and toluene (5.0 mL) were used.

4 mM/[P8] concentration: **A5** (0.10 mmol), catalyst **P8** (20 mol%), KOH (0.20 mmol) and toluene (5.0 mL) were used.

5 mM/[P8] concentration: **A5** (0.10 mmol), catalyst **P8** (25 mol%), KOH (0.20 mmol) and toluene (5.0 mL) were used.

A **P8** (*mM*) vs. time (h) plot is shown in Supplementary Fig. 20. As a result, first-order dependency (1.0073) was observed by plotting ln v_{obs} (*mM*/h) versus ln[**P8** (*mM*)].

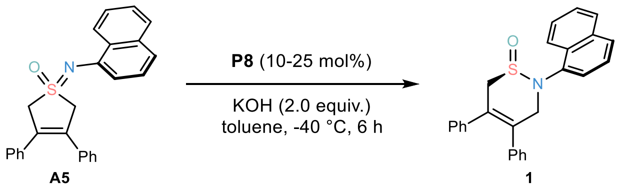

Supplementary Table 17. Kinetic study of catalyst **P8**

**P8** concentration (*mM*)	v_{obs} (*mM*/h)
2	3.2026
3	4.6678
4	6.2317
5	8.1176

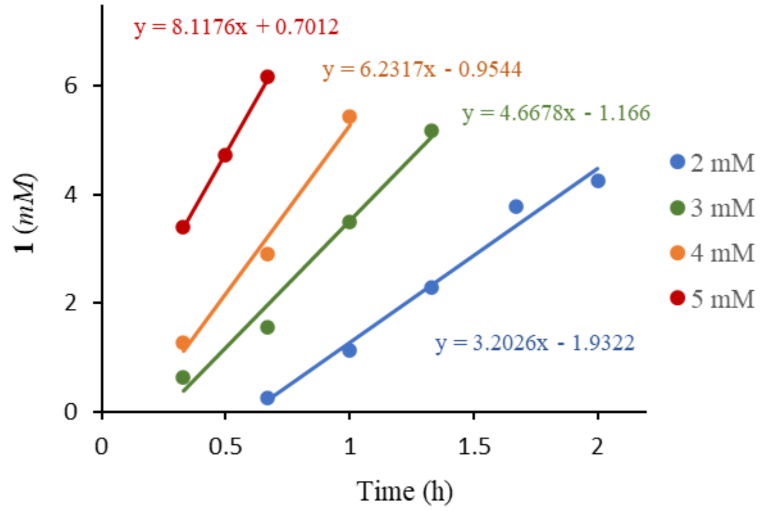

Supplementary Fig. 19. Plot of **1** (*mM*) versus time (h) with variable **P8** concentration.

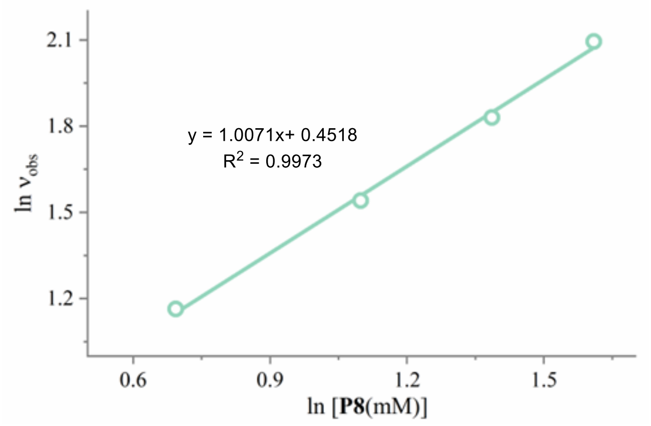

Supplementary Fig. 20. Plot of ln v_{obs} (*mM*/h) versus ln[**P8** (*mM*)].

Kinetic Study of sulfoximine A5

16 mM/[A5] concentration: To a tube (20 mL) with a magnetic stirring bar, the sulfoximine **A5** (0.08 mmol), catalyst **P8** (15 mol%) and KOH (0.20 mmol) were added, followed by the addition of toluene (5.0 mL). The reaction mixture was stirred at -40

°C. After stirring for the indicated period, small amount of reaction mixture was sampled via syringe, which was directly used for NMR analysis to measure the conversion yield.

20 mM/[A5] concentration: **A5** (0.10 mmol), catalyst **P8** (15 mol%), KOH (0.20 mmol) and toluene (5.0 mL) were used.

24 mM/[A5] concentration: **A5** (0.12 mmol), catalyst **P8** (15 mol%), KOH (0.20 mmol) and toluene (5.0 mL) were used.

A **A5** (*mM*) vs. time (h) plot is shown in Supplementary Fig. 20. As a result, zero-order dependency (0.1355) was observed by plotting ln v_{obs} (*mM*/h) versus ln[**A5** (*mM*)].

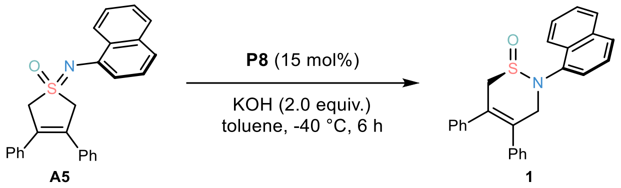

Supplementary Table 18. Kinetic study of sulfoximine **A5**

**A5** concentration (*mM*)	v_{obs} (*mM*/h)
16	4.472
20	4.6678
24	4.7208

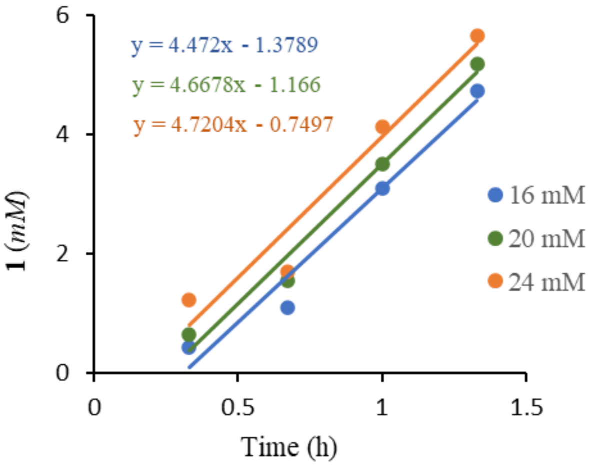

Supplementary Fig. 21. Plot of **1** (*mM*) versus time (h) with variable **A5** concentration.

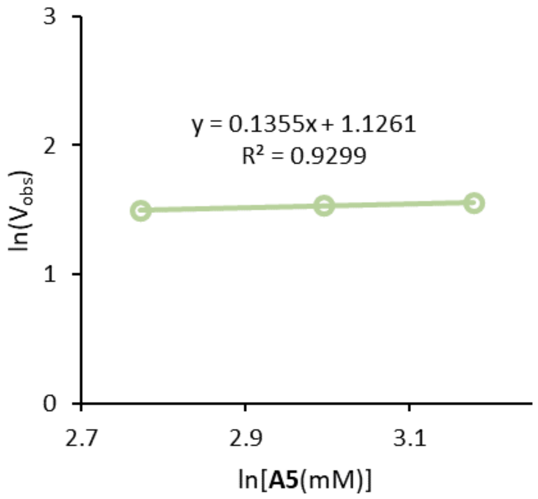

Supplementary Fig. 22. Plot of ln ν_{obs} (*mM*/h) versus ln[**A5** (*mM*)].

Kinetic Study of KOH

20 mM/[KOH] concentration: To a tube (20 mL) with a magnetic stirring bar, the sulfoximine **A5** (0.10 mmol), catalyst **P8** (15 mol%) and KOH (0.10 mmol) were added, followed by the addition of toluene (5.0 mL). The reaction mixture was stirred at -40 °C. After stirring for the indicated period, small amount of reaction mixture was sampled via syringe, which was directly used for NMR analysis to measure the conversion yield.

40 mM/[KOH] concentration: **A5** (0.10 mmol), catalyst **P8** (15 mol%), KOH (0.20 mmol) and toluene (5.0 mL) were used.

80 mM/[KOH] concentration: **A5** (0.10 mmol), catalyst **P8** (15 mol%), KOH (0.40 mmol) and toluene (5.0 mL) were used.

A **KOH** (*mM*) vs. time (h) plot is shown in Supplementary Fig. 24. As a result, zero-order dependency (0.0405) was observed by plotting ln ν_{obs} (*mM*/h) versus ln[**KOH** (*mM*)].

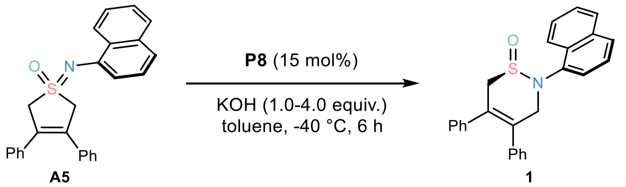

Supplementary Table 19. Kinetic study of KOH

**KOH** concentration (*mM*)	ν_{obs} (*mM*/h)
20	4.5152
40	4.6678
80	4.7758

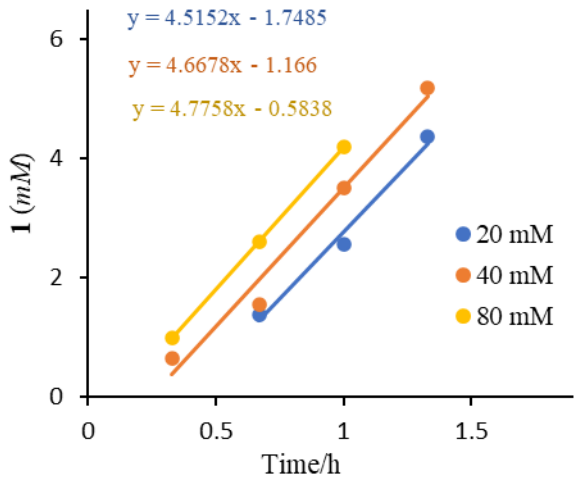

Supplementary Fig. 23. Plot of **1** (*mM*) versus time (h) with variable KOH concentration.

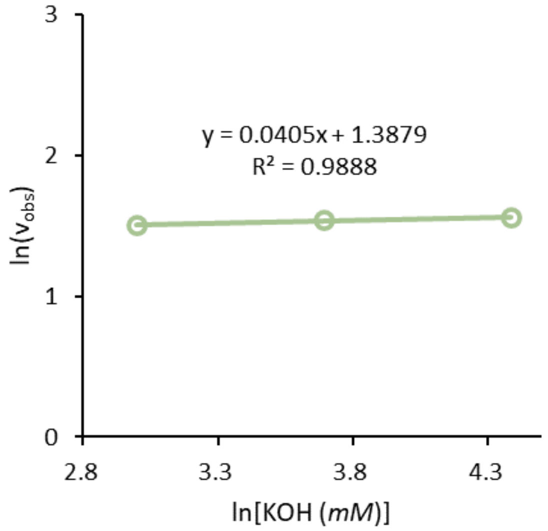

Supplementary Fig. 24. Plot of ln ν_{obs} (*mM*/h) versus ln[KOH (*mM*)].

Investigation of Weak Interactions

Supplementary Table 20. Enantioselective skeletal reorganization catalyzed by different catalysts[a,b]

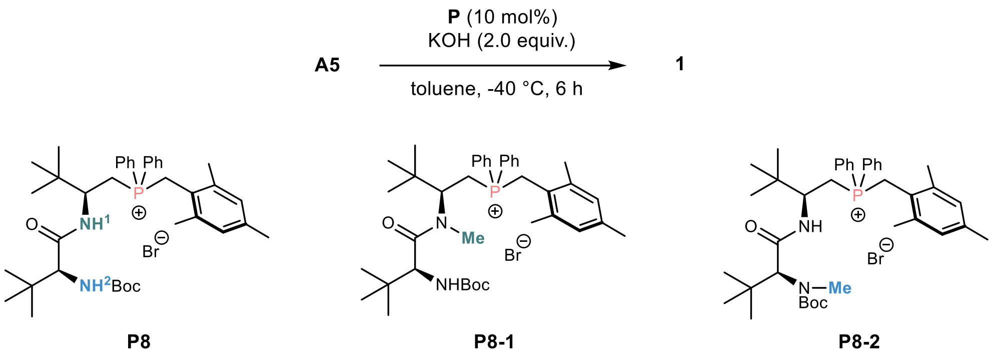

Entry	**catalyst**	e.e. of **1** (%)	yield of **1** (%)
1	**P8**	95	91
2	**P8-1**	--	<5%
3	**P8-2**	70	28

[a] Reaction conditions: **A5** (0.1 mmol) , KOH (2.0 equiv.) and catalyst (10 mol%) in toluene (1.0 mL) at -40 °C for 6 h. [b] All e.e. values were determined by HPLC analysis on a chiral stationary phase, and all yields were isolated yields.

DFT Calculations

Computational details: All calculations were performed using Gaussian 09 program package[14]. Geometries were optimized in toluene solvent and characterized by frequency analysis at 233 K, using M062X functional[15] with 6-31G(d) basis set. The self-consistent reaction field (SCRF) and SMD solvation model[16] were adopted to evaluate the effect of solvent. Single-point energies were obtained by using M062X-D3[17,18]/6-311G(d,p) (SMD, toluene) level of theory. The intrinsic reaction coordinate (IRC) path was traced to check the energy profile connecting each transition state to two associated minima of the proposed mechanism[19]. The optimized geometries of all stationary points were visualized using CYLView software[20].

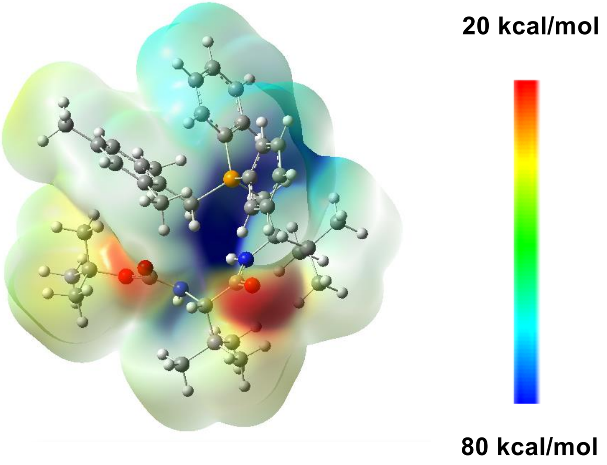

Supplementary Fig. 25. Electrostatic potential (ESP) mapped molecular van der Waals surface of cationic catalyst **P8**.

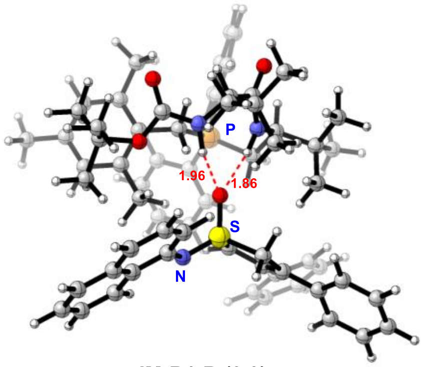

IM-B1-R (0.0)

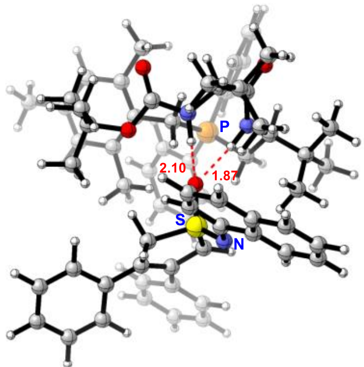

IM-B1-S (2.1)

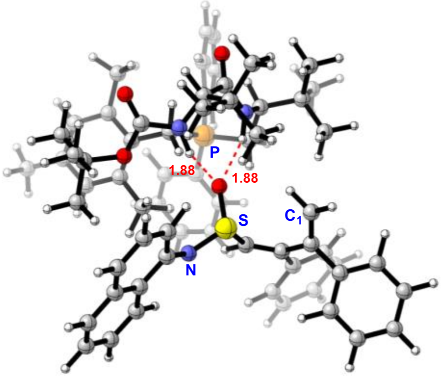

IM-B2-R (-17.5)

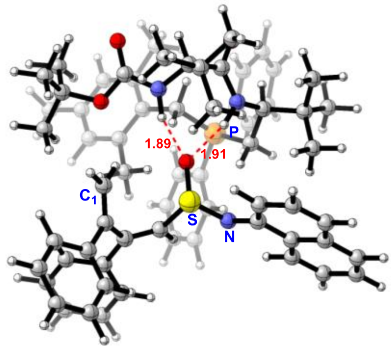

IM-B2-S (-19.8)

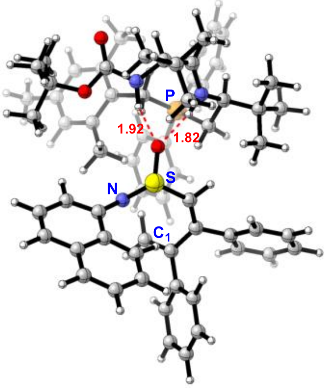

IM-B3-R (-3.8)

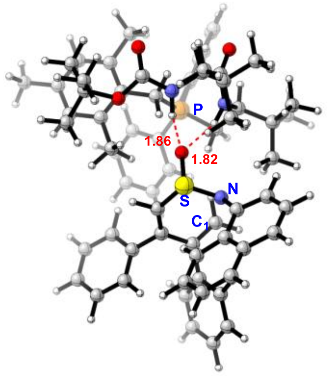

IM-B3-S (-1.9)

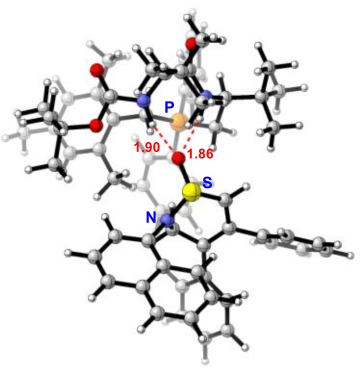

IM-B4-R (-15.6)

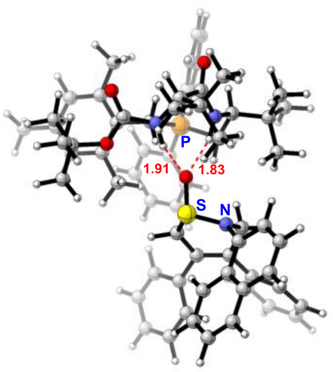

IM-B4-S (-12.2)

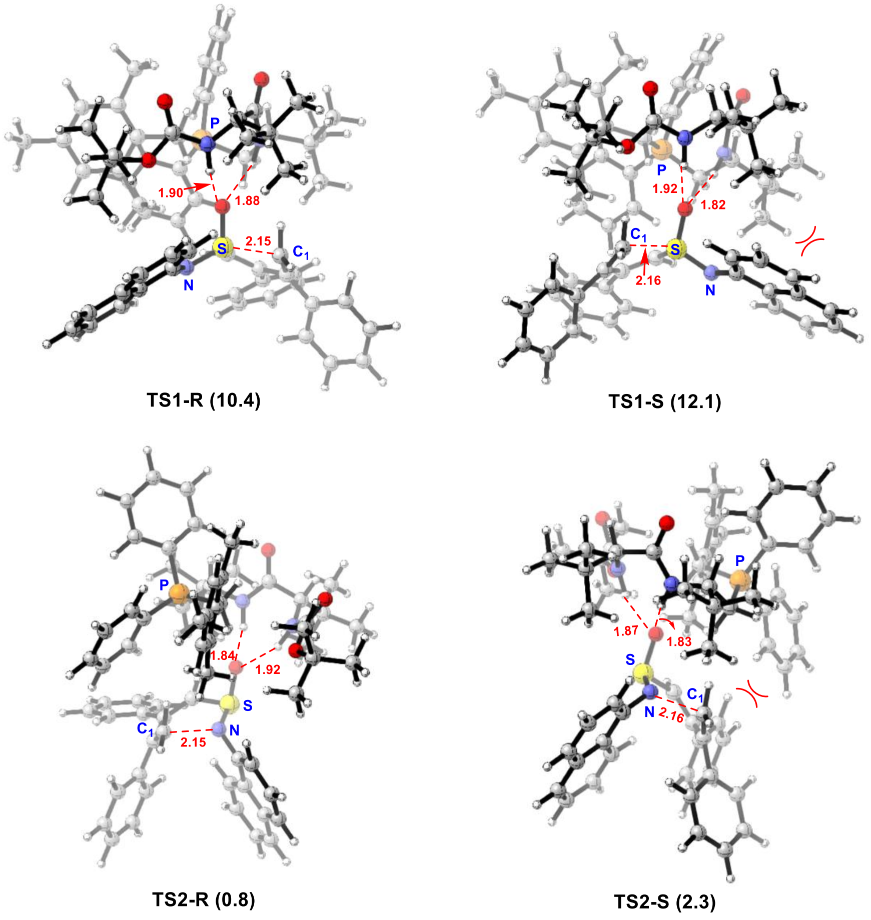

Supplementary Fig. 26. Optimized geometries of **IM-B1-R, IM-B1-S, IM-B2-R, IM-B2-S, IM-B3-R, IM-B3-S, IM-B4-R, IM-B4-S, TS1-R, TS1-S, TS2-R** and **TS2-S**. The relative Gibbs free energy was in kcal mol^{-1}, and the distance was in Å.

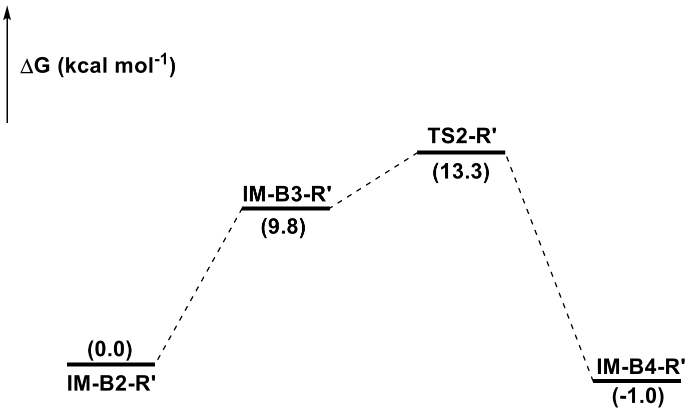

Supplementary Fig. 27. Energy profile for enantioselective transformation of sulfoximine anion to sulfinamide anion along *R*-path without catalyst. The relative free energies (in kcal/mol) are obtained at 233K.

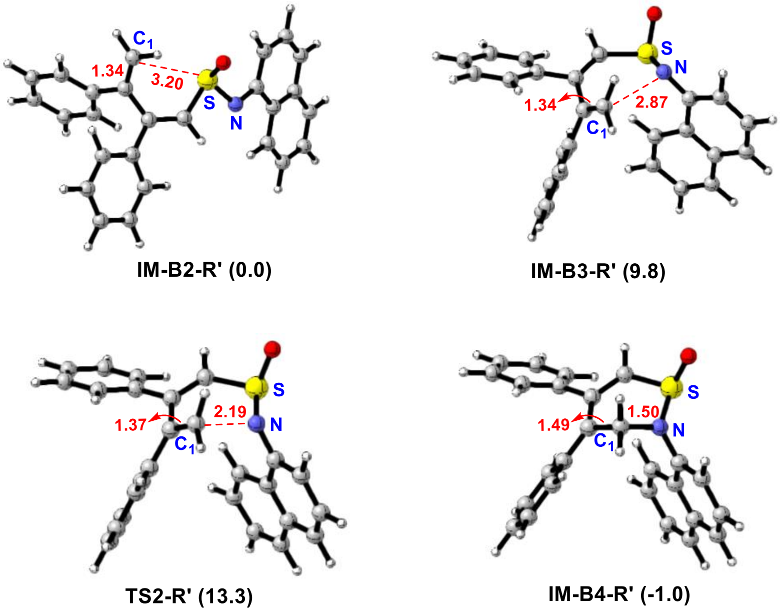

Supplementary Fig. 28. Optimized geometries of transition states and intermediates for enantioselective transformation of sulfoximine anion to sulfinamide anion along *R*-path without catalyst. The relative Gibbs free energy was in kcal mol^{-1}, and the distance was in Å.

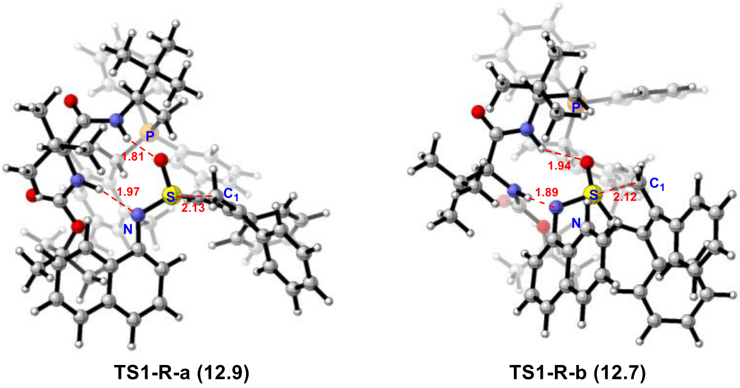

Supplementary Fig. 29. Optimized geometries of **TS1-R-a** and **TS1-R-b** involving the hydrogen bonding (HB) between the NH groups of catalyst and the N and O atoms of the substrate. The relative Gibbs free energy was in kcal mol^{-1}, and the distance was in Å.

Supplementary Table 21. The corrected electronic energies (E_Z), enthalpies (H), and Gibbs free energies (G) for all stationary points (in Hartree) at 233 K, obtained at M062X/6-31G(d)(SMD, toluene) theoretical level.

Structures	^{a}ZPE	$^{b}H_c$	$^{c}G_c$	E_z	H	G	^{d}G

**IM-B1-R**	1.28292	1.32922	1.20996	-3724.79702	-3724.75072	-3724.86997	-3725.73690
**TS1-R**	1.28209	1.32885	1.20787	-3724.77882	-3724.73206	-3724.85303	-3725.72025
**IM-B2-R**	1.28330	1.33068	1.20745	-3724.82179	-3724.77441	-3724.89765	-3725.76484
**IM-B3-R**	1.28252	1.32882	1.20958	-3724.80403	-3724.75773	-3724.87697	-3725.74295
**TS2-R**	1.28285	1.32847	1.21151	-3724.79897	-3724.75335	-3724.87031	-3725.73562
**IM-B4-R**	1.28485	1.33066	1.21284	-3724.82425	-3724.77843	-3724.89626	-3725.76180
**IM-B1-S**	1.28361	1.32945	1.21354	-3724.79713	-3724.75129	-3724.86720	-3725.73359
**TS1-S**	1.28224	1.32866	1.21003	-3724.77905	-3724.73263	-3724.85126	-3725.71762
**IM-B2-S**	1.28368	1.33096	1.20987	-3724.82738	-3724.78011	-3724.90119	-3725.76852
**IM-B3-S**	1.28198	1.32824	1.20842	-3724.79861	-3724.75235	-3724.87217	-3725.73993
**TS2-S**	1.28234	1.32877	1.20846	-3724.79252	-3724.74609	-3724.86639	-3725.73327
**IM-B4-S**	1.28573	1.33157	1.21424	-3724.81971	-3724.77387	-3724.89120	-3725.75638
**IM-B1-R'**	0.39059	0.40571	0.35273	-1530.08383	-1530.06872	-1530.12170	-1530.43887
**TS1-R'**	0.38882	0.40378	0.35061	-1530.06702	-1530.05206	-1530.10523	-1530.42221
**IM-B2-R'**	0.38878	0.40391	0.35073	-1530.10641	-1530.09128	-1530.14446	-1530.46128
**IM-B3-R'**	0.38889	0.40453	0.35006	-1530.08704	-1530.07140	-1530.12587	-1530.44567
**TS2-R'**	0.38913	0.40398	0.35229	-1530.08346	-1530.06860	-1530.12030	-1530.44008
**IM-B4-R'**	0.39161	0.40647	0.35465	-1530.10738	-1530.09252	-1530.14434	-1530.46292
**TS1-R-a**	1.28264	1.32875	1.21178	-3724.77821	-3724.73210	-3724.84908	-3725.71630
**TS1-R-b**	1.28349	1.32934	1.21236	-3724.78030	-3724.73445	-3724.85142	-3725.71660

[a] Zero-point energy;

[b] Thermal correction to enthalpy;

[c] Thermal correction to Gibbs free energy;

[d] Gibbs free energy at the M062X-D3/6-311G(d,p)(SMD, toluene)//M062X/6-31G(d)(SMD, toluene) theoretical level.

Cartesian coordinates of DFT-computed structures.

IM-B1-R

P	0.478409	-2.728287	-0.440172
O	3.163641	1.482788	1.063854
N	-0.378381	-1.269423	2.331539
H	-0.477494	-0.368001	1.860865
H	3.717287	0.509144	-3.629777
O	0.691401	-2.479443	3.927386
O	4.004945	0.289561	2.820750
N	1.756492	0.568192	2.500527
H	1.080542	1.076159	1.931770
C	1.782704	-1.525973	0.070617
H	1.252859	-0.578296	0.217190
H	2.128198	-1.855981	1.055918
C	-2.664254	-2.278647	2.365168
C	5.472684	1.072155	0.342552
H	5.766213	0.480746	1.211067
H	6.358448	1.574908	-0.061669
H	5.076432	0.404594	-0.428813
C	4.427170	2.118717	0.708923
C	3.069684	0.746770	2.187014
C	1.364730	-0.180489	3.676721
H	2.289804	-0.580783	4.099516
C	0.527086	-1.419580	3.327897
C	-1.205664	-2.352759	1.826868
H	-0.776807	-3.286059	2.207391
C	-1.143593	-2.325035	0.291089
H	-1.418654	-1.331254	-0.099151
H	-1.829578	-3.061708	-0.140208
C	2.916565	-1.375992	-0.916939
C	2.803905	-0.384158	-1.911882
C	3.814345	-0.256860	-2.862675
C	0.264305	-2.718048	-2.234613
C	1.345594	-3.064890	-3.058429
H	2.297919	-3.360050	-2.626144

C	1.202661	-3.027333	-4.440471	C	-3.302829	-0.932451	2.017344
H	2.045643	-3.289433	-5.071559	H	-4.342051	-0.900892	2.365811
C	-0.012913	-2.649242	-5.007168	H	-3.311279	-0.750653	0.937313
H	-0.120296	-2.617093	-6.087091	H	-2.767433	-0.103593	2.495197
C	-1.090306	-2.313842	-4.192454	C	-3.503244	-3.415808	1.769327
H	-2.041667	-2.021953	-4.626319	H	-2.986976	-4.380911	1.849778
C	-0.958569	-2.346402	-2.806645	H	-3.750748	-3.241314	0.716255
H	-1.807486	-2.059745	-2.193315	H	-4.451108	-3.500320	2.311483
C	0.944240	-4.396454	0.087635	C	4.062340	-2.188030	-0.861456
C	1.354778	-4.596340	1.413783	C	5.052445	-2.025032	-1.835959
H	1.419313	-3.770158	2.119603	H	5.939736	-2.653291	-1.789659
C	1.680844	-5.876112	1.848472	C	4.944350	-1.075728	-2.849320
H	1.996667	-6.025275	2.875928	C	1.633156	0.560232	-1.938574
C	1.606591	-6.954621	0.968777	H	1.608852	1.177461	-1.032664
H	1.870449	-7.950911	1.310094	H	1.688418	1.233768	-2.797705
C	1.191635	-6.758144	-0.345416	H	0.674245	0.028667	-1.991568
H	1.129501	-7.597494	-1.030685	C	4.273514	-3.223902	0.215567
C	0.854063	-5.482261	-0.789075	H	4.046626	-2.829934	1.212387
H	0.527935	-5.337749	-1.814433	H	3.654767	-4.114489	0.060910
C	0.703963	0.718906	4.760903	H	5.317212	-3.548419	0.223933
C	0.473646	-0.105367	6.030880	C	6.037001	-0.893709	-3.870118
H	1.416992	-0.507999	6.416578	H	6.700477	-1.762185	-3.904176
H	-0.198916	-0.950531	5.852486	H	5.622998	-0.737004	-4.870926
H	0.028745	0.524655	6.809277	H	6.648182	-0.016482	-3.628920
C	1.671637	1.867375	5.067561	S	-1.277161	1.761595	-0.371069
H	1.293366	2.463941	5.905213	O	-0.440067	1.077152	0.694832
H	1.789705	2.531736	4.204697	N	-0.503752	2.871469	-1.171830
H	2.662888	1.486311	5.338586	C	-5.724944	-2.051635	-3.537598
C	-0.630819	1.294220	4.273862	C	-5.858095	-2.011276	-2.150786
H	-1.398588	0.516814	4.195242	C	-5.148146	-1.074501	-1.405654
H	-0.535293	1.777288	3.292791	C	-4.287851	-0.165966	-2.035175
H	-0.993849	2.048721	4.980704	C	-3.468557	0.780746	-1.229634
C	4.053393	2.958231	-0.505159	C	-2.053898	0.725607	-1.443404
H	3.742244	2.318561	-1.337493	C	-4.874377	-1.150502	-4.173529
H	4.912823	3.554262	-0.828426	C	-4.152571	-0.220741	-3.426962
H	3.231091	3.634463	-0.254191	C	-3.932207	1.602700	-0.232487
C	4.879895	3.021065	1.852491	C	-5.312113	1.860760	0.182007
H	4.091316	3.743711	2.090007	C	-6.397438	1.793862	-0.714517
H	5.771370	3.577983	1.546534	C	-7.695222	2.054523	-0.295723
H	5.121819	2.441549	2.745590	C	-7.958817	2.415916	1.025721
C	-2.614929	-2.451043	3.886830	C	-6.897523	2.515172	1.920562
H	-2.004912	-1.673968	4.358390	C	-5.597483	2.241620	1.506460
H	-2.190407	-3.422069	4.164829	C	-2.803848	2.310030	0.485304
H	-3.625745	-2.384817	4.304114	H	-6.284212	-2.778515	-4.119068

H	-6.521024	-2.708696	-1.646742
H	-5.265027	-1.032422	-0.325919
H	-1.554628	0.445750	-2.360441
H	-4.772786	-1.167711	-5.255259
H	-3.489740	0.483725	-3.923513
H	-6.211518	1.555787	-1.756873
H	-8.507657	1.994301	-1.014921
H	-8.973854	2.628787	1.347025
H	-7.079860	2.803830	2.952254
H	-4.788030	2.315136	2.228041
H	-2.662597	2.007704	1.529216
H	-2.859067	3.404065	0.435853
C	0.241176	3.845667	-0.517604
C	0.951915	4.755738	-1.387089
C	0.351843	4.032844	0.854278
C	0.897972	4.615363	-2.796747
C	1.729463	5.809012	-0.829685
C	1.129492	5.082867	1.390809
H	-0.156108	3.358680	1.536951
C	1.588201	5.472670	-3.619018
H	0.291990	3.814999	-3.207404
C	2.429620	6.679420	-1.706626
C	1.807582	5.957605	0.581585
H	1.183560	5.190124	2.471382
C	2.364435	6.517485	-3.067510
H	1.536797	5.351338	-4.697189
H	3.023603	7.480811	-1.273346
H	2.406519	6.762343	0.999030
H	2.907939	7.191563	-3.723614

TS1-R

P	0.95320200	-2.54013700	-0.71647500
O	2.94807600	1.88007100	1.08515800
N	0.07034900	-1.63551900	2.34797200
H	-0.27447800	-0.74174200	1.98982900
H	3.27958700	1.54101200	-3.68352200
O	1.66052300	-2.69332400	3.57566600
O	4.10987800	0.76760800	2.70647200
N	1.83297700	0.63015000	2.52925100
H	1.03204700	1.00201500	2.01756600
C	2.05764600	-1.18895700	-0.12849900
H	1.37360100	-0.38373200	0.16448800
H	2.53386100	-1.56011200	0.78610100
C	-1.74303200	-3.38386700	2.39323100
C	5.25135000	1.98028000	0.23254500
H	5.69848000	1.39400000	1.03642700
H	6.00070800	2.67750900	-0.15885500
H	4.95348200	1.31039500	-0.58068200
C	4.04410700	2.77236400	0.72144000
C	3.06555300	1.07299800	2.15683800
C	1.67625800	-0.28639200	3.63988600
H	2.68682500	-0.49935300	3.99685300
C	1.13621600	-1.65193600	3.18929500
C	-0.44434100	-2.84588900	1.72786200
H	0.30903400	-3.62244900	1.89210200
C	-0.61101600	-2.57610400	0.22333600
H	-1.11287300	-1.61288600	0.04893700
H	-1.20485700	-3.36259300	-0.25357800
C	3.06066200	-0.72254000	-1.15845200
C	2.68929200	0.32731900	-2.02035300
C	3.57500600	0.73094800	-3.01957100
C	0.53115200	-2.32384100	-2.45943100
C	1.55109300	-2.34662500	-3.42220800
H	2.58467500	-2.51247900	-3.13010700
C	1.23917600	-2.14974500	-4.76224600
H	2.03284400	-2.15991300	-5.50250300
C	-0.08224600	-1.93484200	-5.14933800
H	-0.31969500	-1.77772100	-6.19708300
C	-1.09769200	-1.92241900	-4.19762300
H	-2.13009900	-1.76006400	-4.49174700
C	-0.79643200	-2.11636400	-2.85218300
H	-1.60214300	-2.08496800	-2.12508000
C	1.75430400	-4.15352200	-0.51518400
C	2.39042600	-4.46538400	0.69570200
H	2.45161600	-3.74155400	1.50659700
C	2.95088000	-5.72550600	0.87506500
H	3.44222900	-5.96114900	1.81346100
C	2.88588100	-6.67309000	-0.14489200
H	3.33059400	-7.65316700	-0.00220300
C	2.25005000	-6.36691100	-1.34454400
H	2.19437600	-7.10448000	-2.13880500
C	1.67949900	-5.11086000	-1.53218700
H	1.18314900	-4.88073400	-2.46982600
C	0.88480700	0.33341800	4.82557800
C	0.90974300	-0.64776200	6.00115700
H	1.93798800	-0.85367600	6.31963300
H	0.44495900	-1.60566800	5.74352000
H	0.36652800	-0.22678200	6.85458300

C	1.59325800	1.63120900	5.22889100	S	-1.60514400	1.50225100	0.12142100
H	1.13507400	2.04536100	6.13394400	O	-0.49352300	0.78175000	0.90453500
H	1.52313700	2.38459800	4.43714900	N	-1.06300600	2.73374200	-0.72856300
H	2.65537600	1.45400300	5.43296400	C	-5.88314400	-1.98901600	-3.77298100
C	-0.56734500	0.64770200	4.44649400	C	-6.03040300	-2.30796200	-2.42454500
H	-1.15339700	-0.26649200	4.29665500	C	-5.31951500	-1.60450600	-1.45764900
H	-0.63080000	1.24598000	3.52859600	C	-4.44608600	-0.57317700	-1.82314000
H	-1.04568900	1.22043600	5.24882000	C	-3.66940200	0.14492500	-0.77534900
C	3.45023200	3.59613900	-0.41269100	C	-2.34573500	0.47655900	-1.04633100
H	3.26123500	2.96791600	-1.28895400	C	-5.02064100	-0.96083100	-4.14750200
H	4.14304100	4.39315800	-0.70178200	C	-4.30272100	-0.26098000	-3.18054900
H	2.50838400	4.04747200	-0.09117600	C	-4.23062100	0.55275900	0.46107700
C	4.37514900	3.67168400	1.90822200	C	-5.64984700	0.89941200	0.65633400
H	3.47539700	4.21002700	2.22628700	C	-6.48956200	1.31908900	-0.39123300
H	5.12685200	4.40904900	1.60867700	C	-7.81657300	1.65754100	-0.15703300
H	4.76878500	3.09420500	2.74723000	C	-8.34420100	1.60883400	1.13281300
C	-1.44035400	-3.64902200	3.87199900	C	-7.52530600	1.20922300	2.18571900
H	-1.15239700	-2.72718000	4.38773000	C	-6.20270000	0.84952400	1.94791500
H	-0.62316300	-4.36851000	3.99123800	C	-3.25369700	0.99138800	1.40432900
H	-2.33078800	-4.04838400	4.37001800	H	-6.44229800	-2.53364600	-4.52792900
C	-2.89130900	-2.38072200	2.28544600	H	-6.70294700	-3.10577400	-2.12366800
H	-3.81736900	-2.82110700	2.67258300	H	-5.43971900	-1.84380900	-0.40438100
H	-3.08498800	-2.06972300	1.25063000	H	-1.88104700	0.40752700	-2.02332900
H	-2.68363600	-1.48485300	2.88030500	H	-4.91299400	-0.69451500	-5.19536200
C	-2.14139100	-4.70917400	1.73008000	H	-3.64978100	0.55706500	-3.47384000
H	-1.28124000	-5.38480200	1.63903200	H	-6.08802900	1.40273100	-1.39636100
H	-2.57546100	-4.56713100	0.73457400	H	-8.43878000	1.98062800	-0.98707600
H	-2.89897100	-5.21522300	2.33764300	H	-9.37970200	1.88085300	1.31424800
C	4.33347100	-1.31017500	-1.27392500	H	-7.92113700	1.16210300	3.19652900
C	5.18714900	-0.87545200	-2.29097300	H	-5.58128200	0.50472200	2.77123600
H	6.17203200	-1.33077300	-2.37725500	H	-2.54138600	0.27157200	1.80708600
C	4.82448500	0.13396600	-3.18109800	H	-3.52893400	1.75987400	2.12874200
C	1.37611800	1.04347400	-1.85679300	C	-0.38663800	3.76784600	-0.11505700
H	1.30919200	1.51854800	-0.87136500	C	0.01898400	4.85416300	-0.98184500
H	1.25643700	1.82500100	-2.61181300	C	-0.07736000	3.86873400	1.23954300
H	0.51806900	0.36401100	-1.94199200	C	-0.22991300	4.80721000	-2.37580900
C	4.82663900	-2.38021100	-0.33147000	C	0.68422400	5.98865800	-0.43811200
H	4.56176600	-2.16482300	0.70930500	C	0.57145000	5.00705000	1.76129900
H	4.42525300	-3.36769900	-0.58214900	H	-0.34235600	3.06168500	1.91591700
H	5.91650800	-2.44858800	-0.38194000	C	0.16816800	5.83145000	-3.20135600
C	5.78136700	0.60666900	-4.24393800	H	-0.74729800	3.93918700	-2.77148700
H	6.36421200	-0.22348100	-4.65429400	C	1.08080600	7.03140800	-1.31720700
H	5.25240800	1.09608900	-5.06630000	C	0.95065300	6.05182000	0.95631800
H	6.49136200	1.33199500	-3.82996900	H	0.77315700	5.04591200	2.82924600

C	0.83236500	6.95780600	-2.66493600
H	-0.02974200	5.77976000	-4.26813900
H	1.58935800	7.89561700	-0.89609200
H	1.45377100	6.92429700	1.36369100
H	1.14408500	7.76531400	-3.32147100

IM-B2-R

P	1.08771600	-2.44307100	-0.88643400
O	2.83481200	2.06594600	1.12810100
N	0.27841000	-1.83691000	2.33780500
H	-0.21326900	-0.98393500	2.05348100
H	3.20531600	1.85444000	-3.64528300
O	2.18550900	-2.65914300	3.25885300
O	4.04590100	1.09272100	2.79886800
N	1.82174300	0.65120100	2.50480400
H	0.99860700	0.84843200	1.92603800
C	2.14720800	-1.09821300	-0.21559000
H	1.42459100	-0.34512600	0.12389600
H	2.63858500	-1.50632500	0.67549200
C	-1.08502900	-3.96737200	2.29194100
C	5.11925700	2.30088600	0.27856700
H	5.57314100	1.70167400	1.06948100
H	5.85054000	3.03757300	-0.07247300
H	4.85892800	1.65134300	-0.56343200
C	3.87570400	3.02772900	0.77846200
C	2.99956500	1.26144200	2.19566100
C	1.75984500	-0.31231500	3.58660000
H	2.78079300	-0.40607600	3.96300700
C	1.42785700	-1.71416400	3.05557200
C	-0.02392800	-3.05985300	1.61009600
H	0.89354600	-3.65469700	1.62882500
C	-0.38737800	-2.67372400	0.16274000
H	-0.95071400	-1.73060900	0.14319000
H	-0.97667700	-3.45099000	-0.33413900
C	3.12359300	-0.52519000	-1.21867000
C	2.68899400	0.53941500	-2.03647200
C	3.54823500	1.03575300	-3.01516500
C	0.53851400	-2.06938700	-2.56293000
C	1.49028600	-1.98167700	-3.58807800
H	2.54150900	-2.16522300	-3.38131700
C	1.08576200	-1.64734600	-4.87535600
H	1.82544600	-1.56787600	-5.66544900
C	-0.26043700	-1.40828100	-5.14453800
H	-0.57098900	-1.14452300	-6.15090800
C	-1.20904000	-1.50784800	-4.13011900
H	-2.25946800	-1.32827200	-4.33643800
C	-0.81428200	-1.83593700	-2.83615500
H	-1.56568800	-1.89500100	-2.05332600
C	1.95663400	-4.03360000	-0.91548800
C	2.74702600	-4.41749900	0.17749200
H	2.92371200	-3.73871800	1.00923300
C	3.31274800	-5.68787300	0.21042900
H	3.92303700	-5.97770800	1.05951200
C	3.09643800	-6.57714300	-0.84004200
H	3.54083100	-7.56730300	-0.81099500
C	2.31046400	-6.19939700	-1.92561000
H	2.14109300	-6.89135600	-2.74433800
C	1.73651600	-4.93207300	-1.96564800
H	1.12278400	-4.64645700	-2.81470200
C	0.87373800	0.15606300	4.77340000
C	0.98774800	-0.87750100	5.89776300
H	2.02704600	-0.99079300	6.22678000
H	0.62901700	-1.86363000	5.58088600
H	0.38973100	-0.56635600	6.76150500
C	1.43136000	1.49914600	5.25849700
H	0.90337700	1.82138500	6.16284500
H	1.30869600	2.27775200	4.49809000
H	2.49927700	1.42218300	5.49204200
C	-0.59890500	0.33623500	4.38384500
H	-1.09180900	-0.62728900	4.21075500
H	-0.71216800	0.94640600	3.47878900
H	-1.13633700	0.84186700	5.19385400
C	3.24391700	3.83955700	-0.34511100
H	3.03783900	3.20457500	-1.21200300
H	3.93001800	4.63412800	-0.65631300
H	2.30799600	4.29512900	-0.00880000
C	4.16844500	3.93422500	1.97060100
H	3.23563500	4.38253300	2.32924200
H	4.83374600	4.74389100	1.65348200
H	4.64491800	3.38699700	2.78511200
C	-0.62061800	-4.24817100	3.72455700
H	-0.56895500	-3.32371200	4.30841100
H	0.37140900	-4.71172700	3.73962000
H	-1.32670600	-4.92282700	4.22151200
C	-2.46068100	-3.30529000	2.32986100
H	-3.19889800	-3.99082800	2.76150600
H	-2.81559700	-3.02322700	1.33068000
H	-2.44150000	-2.40532500	2.95325800

C	-1.15867800	-5.29987600	1.53508800
H	-0.16094900	-5.73649500	1.40043800
H	-1.62348100	-5.20181200	0.54820200
H	-1.76217500	-6.01627800	2.10257000
C	4.43360100	-1.01742100	-1.34566400
C	5.26097100	-0.48742500	-2.34081500
H	6.27496700	-0.87113300	-2.43394500
C	4.83493400	0.52580700	-3.19525300
C	1.34577600	1.18356100	-1.82636400
H	1.27732400	1.62541500	-0.82713700
H	1.17418200	1.98254200	-2.55275300
H	0.51250600	0.47661900	-1.90557500
C	5.00892900	-2.06895500	-0.42900600
H	4.71895500	-1.90877600	0.61521900
H	4.69985000	-3.07908300	-0.71430400
H	6.10096800	-2.03898700	-0.46938900
C	5.75363200	1.10622800	-4.23836400
H	6.57293500	0.42199300	-4.47569200
H	5.21327200	1.33101000	-5.16299600
H	6.19585100	2.04432400	-3.88344900
S	-1.60237200	1.55751500	0.54272100
O	-0.52500600	0.47153400	0.89723000
N	-1.03929500	2.68414900	-0.47338400
C	-5.63005100	-1.86760600	-3.82169600
C	-5.68218500	-2.42516900	-2.54579100
C	-5.08136700	-1.77451200	-1.47329600
C	-4.40125000	-0.56492700	-1.66297300
C	-3.72796800	0.10045200	-0.51117300
C	-2.53368200	0.68578000	-0.69707000
C	-4.97467900	-0.65373200	-4.01745300
C	-4.36179000	-0.00766200	-2.94649000
C	-4.42243700	0.06399900	0.80856900
C	-5.87563200	0.38405700	0.82990100
C	-6.39468000	1.36057000	-0.02854600
C	-7.74465200	1.69210400	0.01029100
C	-8.59927900	1.04572600	0.90035500
C	-8.09545000	0.06398000	1.75050700
C	-6.74451700	-0.26665900	1.71247600
C	-3.74949700	-0.22294000	1.93072400
H	-6.11112000	-2.36908900	-4.65595600
H	-6.19909000	-3.36624000	-2.38335000
H	-5.13817200	-2.20414100	-0.47604200
H	-2.02411100	0.68299600	-1.66051600
H	-4.94875200	-0.20065100	-5.00451800
H	-3.87114100	0.95075400	-3.09592400
H	-5.72990600	1.87321100	-0.71942000
H	-8.12963100	2.45779700	-0.65644500
H	-9.65433500	1.30129200	0.92683000
H	-8.75751100	-0.45281000	2.43917400
H	-6.35795700	-1.04912100	2.36029300
H	-2.69352900	-0.47348900	1.90284400
H	-4.22637300	-0.18679800	2.90690300
C	-0.48810700	3.79652100	0.12552800
C	-0.33321300	4.96747400	-0.71455500
C	-0.03470300	3.88791800	1.44185800
C	-0.72949100	4.94297300	-2.07451900
C	0.22266900	6.16428100	-0.18057900
C	0.48344900	5.09155800	1.96289900
H	-0.05175700	3.00970400	2.08312000
C	-0.57692400	6.04703200	-2.87873400
H	-1.15803900	4.02367500	-2.45977000
C	0.36514700	7.28919300	-1.03678700
C	0.62055400	6.21296400	1.18316700
H	0.79652900	5.11470000	3.00446300
C	-0.02209200	7.23570000	-2.35226000
H	-0.88502300	6.01096400	-3.91990700
H	0.79046300	8.20137900	-0.62441800
H	1.03329600	7.13393600	1.58536900
H	0.09468200	8.10693600	-2.99106300

IM-B3-R

P	2.16822400	-1.76449900	-0.83305700
O	2.21171100	3.29912300	0.51430600
N	1.67361300	-1.06296600	2.37131500
H	0.86381700	-0.47879500	2.12934300
H	1.65011200	2.57415900	-4.24227900
O	3.82829400	-1.10497200	3.09157400
O	4.06792200	2.96601100	1.79683400
N	2.14444600	1.77534400	2.12727300
H	1.18851000	1.62225300	1.79040900
C	2.62166700	-0.01007500	-0.50544400
H	1.71420100	0.41910400	-0.06242800
H	3.39224400	-0.02762200	0.27390800
C	1.00575900	-3.45036300	2.82491700
C	3.98019500	4.24568700	-0.89956800
H	4.79623100	3.90605600	-0.26005500
H	4.28990000	5.16403200	-1.41110100
H	3.77439100	3.48518000	-1.65867100

C	2.72122000	4.52611100	-0.08657700	C	0.48958300	0.93359900	4.54217100
C	2.91218400	2.70806700	1.50382600	H	0.34321900	-0.15067100	4.48369100
C	2.68796200	1.00739700	3.23022300	H	-0.02337500	1.39397800	3.68925700
H	3.72788300	1.32491700	3.33368500	H	-0.00337700	1.28589400	5.45523100
C	2.78344400	-0.48694200	2.89613600	C	1.58495300	4.95688300	-1.00478900
C	1.68383400	-2.42713300	1.87058900	H	1.39572300	4.18798100	-1.76160800
H	2.73388600	-2.73043000	1.82261500	H	1.85129900	5.88729900	-1.51615800
C	1.06005500	-2.41312100	0.46358900	H	0.66878200	5.12025300	-0.42939200
H	0.16464500	-1.77690400	0.45437800	C	2.95163200	5.58088600	0.99303400
H	0.78424100	-3.41907200	0.12981300	H	2.05354200	5.68718000	1.61127800
C	3.05160800	0.74665500	-1.74161600	H	3.15437700	6.54649700	0.51849100
C	2.06009300	1.41524700	-2.48926800	H	3.79574400	5.31954300	1.63245300
C	2.42258900	2.06683600	-3.66713700	C	1.62932100	-3.29422500	4.21541300
C	1.30337700	-1.90534400	-2.41141800	H	1.39618000	-2.31403700	4.64285500
C	1.99842700	-1.63142600	-3.59795900	H	2.71913700	-3.39532900	4.17739000
H	3.05563800	-1.37912400	-3.57315800	H	1.23253900	-4.05915600	4.89179100
C	1.32812900	-1.66667000	-4.81487900	C	-0.50211500	-3.21821600	2.91100600
H	1.86785700	-1.44497500	-5.72984800	H	-0.95416900	-3.90842000	3.63260700
C	-0.03146600	-1.96975400	-4.85495900	H	-0.99528400	-3.38827400	1.94688700
H	-0.55428700	-1.98822800	-5.80626700	H	-0.72437700	-2.19611800	3.24445800
C	-0.72356000	-2.24329600	-3.67849300	C	1.28727600	-4.87108600	2.32026800
H	-1.78575100	-2.46532100	-3.70458000	H	2.36248400	-5.03473700	2.17654100
C	-0.06190800	-2.21219000	-2.45395300	H	0.77813100	-5.09264900	1.37611400
H	-0.62501400	-2.40293700	-1.54402100	H	0.93255900	-5.60406300	3.05269100
C	3.63939500	-2.82294200	-0.86820500	C	4.39448100	0.79725600	-2.15385700
C	4.60217200	-2.68870800	0.14331700	C	4.71159200	1.46472600	-3.34091800
H	4.51111400	-1.92613700	0.91493200	H	5.75277500	1.50336200	-3.65526500
C	5.69366800	-3.54972200	0.17527200	C	3.74247400	2.09288500	-4.11898200
H	6.43527000	-3.44066600	0.95981100	C	0.63394800	1.47247800	-2.01346000
C	5.83419500	-4.53888800	-0.79636100	H	-0.00285500	1.99138600	-2.73561900
H	6.69169400	-5.20420300	-0.77120800	H	0.19618100	0.48281000	-1.84522200
C	4.87706600	-4.67669100	-1.79791000	H	0.55315900	1.99285100	-1.05259900
H	4.98414000	-5.44772700	-2.55408200	C	5.51860900	0.18216400	-1.35688500
C	3.77617200	-3.82501400	-1.83460800	H	5.41759200	0.37902700	-0.28395100
H	3.03131800	-3.94065600	-2.61596000	H	5.57641600	-0.90246000	-1.49407000
C	1.97603000	1.30516600	4.57897700	H	6.47582800	0.60272600	-1.67602900
C	2.68258700	0.52260900	5.68981800	C	4.11270200	2.83266600	-5.37790200
H	3.74075500	0.80050700	5.75632400	H	4.22479100	3.90483000	-5.17868800
H	2.63538500	-0.55881000	5.52120700	H	5.06010600	2.47209500	-5.78827100
H	2.21336000	0.73365700	6.65721800	H	3.34083800	2.72241000	-6.14547700
C	2.11058800	2.80768900	4.85048700	S	-1.75423500	0.85504100	0.96827800
H	1.70277300	3.04882400	5.83858100	O	-0.22561600	0.53660200	1.07469800
H	1.56362000	3.39569500	4.10632400	N	-2.07128600	1.65740800	-0.37045900
H	3.16056100	3.12096300	4.82668900	C	-4.30700900	-5.50503200	0.88731700

C	-4.11521300	-4.58852700	1.91869100
C	-3.79554000	-3.26567900	1.62815200
C	-3.65460500	-2.83758400	0.30222200
C	-3.26933200	-1.42996100	-0.02736200
C	-2.34711200	-0.83003500	0.75358700
C	-4.18625600	-5.08720700	-0.43634600
C	-3.86983400	-3.76400300	-0.72568800
C	-3.90321000	-0.83054900	-1.23098800
C	-5.37944900	-0.95983200	-1.37093700
C	-6.20322000	-0.99295300	-0.23861500
C	-7.58572200	-1.08192000	-0.36272900
C	-8.17295000	-1.14770900	-1.62369700
C	-7.36452200	-1.13068200	-2.75824600
C	-5.98240000	-1.04428800	-2.63182700
C	-3.15839100	-0.19271400	-2.14541400
H	-4.56015900	-6.53650200	1.11340800
H	-4.22533400	-4.90048500	2.95324700
H	-3.66910400	-2.54784200	2.43500900
H	-1.92514900	-1.41447700	1.56850900
H	-4.34351000	-5.79362200	-1.24605900
H	-3.78718000	-3.43797300	-1.75963200
H	-5.75336300	-0.94081100	0.75041000
H	-8.20539700	-1.09735300	0.52916800
H	-9.25197100	-1.21968400	-1.72214400
H	-7.81117000	-1.19674100	-3.74615500
H	-5.35733700	-1.05816400	-3.52066600
H	-2.08268200	-0.12261700	-2.04755600
H	-3.61327000	0.31249400	-2.99387200
C	-2.66589700	2.91395100	-0.24571000
C	-3.95981600	3.12819700	0.35832800
C	-2.04016200	4.00409800	-0.83372500
C	-4.74691000	2.05663800	0.85479100
C	-4.49922700	4.44874900	0.42187500
C	-2.59332400	5.29803500	-0.78911100
H	-1.08353800	3.83463900	-1.31710500
C	-5.97393400	2.27495700	1.42963600
H	-4.37734800	1.04289800	0.74045200
C	-5.76304900	4.64551700	1.04352100
C	-3.78882200	5.53175200	-0.15503100
H	-2.05172800	6.12018900	-1.25099400
C	-6.48162400	3.59029800	1.54296100
H	-6.56631500	1.43510700	1.78208400
H	-6.15618200	5.65786000	1.10316700
H	-4.21231500	6.53107900	-0.09955200
H	-7.44933000	3.75795400	2.00723700

TS2-R

P	2.25229400	-1.69748900	-0.92311600
O	2.06787700	3.31822500	0.60363400
N	1.81074500	-1.12707900	2.31731200
H	0.97676600	-0.56760900	2.10542700
H	1.57017600	2.76154100	-4.17772900
O	3.98688300	-1.09993900	2.97090900
O	3.97336300	3.07407800	1.83482700
N	2.14725600	1.73585700	2.15590200
H	1.19618200	1.53669800	1.83128700
C	2.64130300	0.06298000	-0.54314100
H	1.71808900	0.43723300	-0.08371800
H	3.41306200	0.04889500	0.23511800
C	1.24585900	-3.55344600	2.70185000
C	3.73491800	4.45281200	-0.79343100
H	4.58761400	4.18032400	-0.17031700
H	3.95021000	5.40285500	-1.29537100
H	3.58976300	3.68603100	-1.56031700
C	2.46710800	4.60162800	0.04012400
C	2.83307900	2.74419300	1.55498700
C	2.76696900	0.95744100	3.21053300
H	3.79584700	1.31657600	3.28542100
C	2.91232600	-0.52051100	2.82532200
C	1.86174600	-2.47267300	1.76873900
H	2.92200000	-2.73127300	1.68900100
C	1.20647600	-2.43685900	0.37732900
H	0.28101700	-1.84591900	0.41001300
H	0.97220000	-3.44289800	0.01279000
C	3.03703600	0.89335100	-1.74438700
C	2.02164400	1.54902000	-2.47102500
C	2.36157500	2.26778800	-3.61665300
C	1.34539000	-1.81932500	-2.47795600
C	1.98832200	-1.47239400	-3.67448100
H	3.03716500	-1.18625900	-3.67353700
C	1.27541200	-1.48125600	-4.86771400
H	1.77433900	-1.20569300	-5.79121800
C	-0.07494200	-1.82726500	-4.87275800
H	-0.63124900	-1.82177900	-5.80500800
C	-0.71469700	-2.17506600	-3.68632400
H	-1.76990200	-2.42973200	-3.68205000
C	-0.00842400	-2.17643200	-2.48639800
H	-0.53290300	-2.42730100	-1.56811200

C	3.75532000	-2.70434500	-1.02942800
C	4.73995600	-2.56509800	-0.04005800
H	4.65173500	-1.81567100	0.74443100
C	5.84858000	-3.40470600	-0.04602700
H	6.60613000	-3.29207400	0.72260700
C	5.98346600	-4.37847300	-1.03363200
H	6.85286100	-5.02860400	-1.03693900
C	5.00527400	-4.52087800	-2.01416900
H	5.10864900	-5.28008300	-2.78273600
C	3.88771600	-3.69015500	-2.01321600
H	3.12588600	-3.81064700	-2.77748900
C	2.09773000	1.17854800	4.59575500
C	2.86189500	0.36966900	5.64802700
H	3.91659700	0.66547600	5.68361700
H	2.82718900	-0.70581800	5.44271400
H	2.42716800	0.53705300	6.63973300
C	2.20285800	2.67114100	4.92791400
H	1.80330300	2.86176300	5.93030900
H	1.63242300	3.27730400	4.21651400
H	3.24499100	3.00900700	4.90423500
C	0.62053700	0.76962500	4.59473800
H	0.49559500	-0.31226400	4.47441600
H	0.06177000	1.27158500	3.79584300
H	0.15723400	1.05207200	5.54675200
C	1.28661400	4.96721000	-0.85116900
H	1.16573800	4.22435700	-1.64669100
H	1.45648300	5.94484500	-1.31305900
H	0.36376900	5.00975900	-0.26450700
C	2.62426100	5.63233200	1.15506700
H	1.71809000	5.66265300	1.76974800
H	2.77148600	6.62374300	0.71475500
H	3.47963700	5.40052800	1.79136900
C	1.89244800	-3.41330900	4.08396900
H	1.60307500	-2.47064100	4.55896600
H	2.98536800	-3.43576000	4.01675500
H	1.56655100	-4.23183900	4.73499900
C	-0.26912500	-3.39169300	2.83046500
H	-0.66898300	-4.11416800	3.55089800
H	-0.78236200	-3.56423400	1.87706400
H	-0.52667500	-2.38734800	3.19084600
C	1.57936600	-4.94324600	2.14571500
H	2.65931400	-5.05905500	1.99073600
H	1.07427500	-5.15160600	1.19642100
H	1.25837000	-5.71505400	2.85339300
C	4.37986900	1.03844500	-2.13375200
C	4.67491900	1.77190300	-3.28677300
H	5.71621600	1.88039200	-3.58366800
C	3.68237500	2.37968300	-4.05158500
C	0.59042000	1.51813800	-2.00776500
H	-0.06142500	2.04685300	-2.70970000
H	0.20702400	0.49836300	-1.90809300
H	0.47808900	1.97894400	-1.01964000
C	5.52302600	0.46435600	-1.33461300
H	5.42327000	0.68727700	-0.26614000
H	5.60106500	-0.62137600	-1.44418500
H	6.46941500	0.89597900	-1.67028800
C	4.02802500	3.19045000	-5.27324900
H	4.10712600	4.25463800	-5.02201400
H	4.98578700	2.87960900	-5.69995300
H	3.25924700	3.09327800	-6.04556200
S	-1.70591000	0.61386200	1.05148300
O	-0.16007700	0.42447500	1.05031900
N	-2.18038800	1.44461400	-0.24319600
C	-4.77741000	-5.48112900	0.86221000
C	-4.40264100	-4.63571600	1.90385100
C	-3.92863100	-3.35610600	1.63021400
C	-3.81202400	-2.90143900	0.31034700
C	-3.25603800	-1.55254500	-0.00128700
C	-2.25384400	-1.06343600	0.78508400
C	-4.67690300	-5.03610700	-0.45483700
C	-4.19923400	-3.75903600	-0.72722300
C	-3.71481700	-0.80056100	-1.15959400
C	-5.14388800	-0.75336600	-1.54165800
C	-6.16550300	-0.96629100	-0.60293700
C	-7.50509100	-0.91072000	-0.97155400
C	-7.86280100	-0.63344500	-2.28892200
C	-6.86212400	-0.42111700	-3.23510400
C	-5.52301000	-0.49098100	-2.86736300
C	-2.78262400	0.02396500	-1.73835800
H	-5.15306800	-6.47765000	1.07480100
H	-4.49096100	-4.96826300	2.93412700
H	-3.66479400	-2.69009400	2.44794200
H	-1.85852400	-1.67698800	1.58941200
H	-4.97124000	-5.68643700	-1.27343100
H	-4.12164800	-3.41235300	-1.75431900
H	-5.90298100	-1.17053500	0.43180600
H	-8.27375700	-1.07375700	-0.22135600
H	-8.90906000	-0.58600200	-2.57579000

H	-7.12478800	-0.21174400	-4.26843700
H	-4.75112700	-0.35540200	-3.62073000
H	-1.73269200	-0.23738800	-1.67857700
H	-3.05563100	0.73500200	-2.51438200
C	-2.88245500	2.64561700	-0.06147700
C	-4.13381200	2.74161700	0.64588600
C	-2.39421700	3.77793500	-0.69279600
C	-4.77111000	1.61301200	1.22332200
C	-4.77934400	4.01109000	0.75076200
C	-3.05218300	5.02063300	-0.60409600
H	-1.46353500	3.68634200	-1.24460300
C	-5.95981000	1.73366400	1.89941200
H	-4.32403600	0.63246800	1.09854700
C	-6.00029900	4.10582300	1.47210100
C	-4.21207100	5.14696900	0.11874200
H	-2.62034300	5.88563900	-1.10083600
C	-6.57651900	2.99867800	2.03886400
H	-6.43504500	0.85251800	2.32111700
H	-6.47522700	5.08043800	1.55683100
H	-4.71688100	6.10538300	0.20718100
H	-7.51340500	3.08643200	2.58143600

IM-B4-R

P	-1.93423400	-1.82562000	0.92192100
O	-2.26292000	3.28426100	-0.43475900
N	-1.97092600	-1.10042200	-2.33020400
H	-1.14768200	-0.50037100	-2.21279400
H	-1.60232000	2.61969100	4.34121300
O	-4.21649700	-1.18783900	-2.66214900
O	-4.27798800	3.01030000	-1.47059900
N	-2.46113200	1.72740500	-2.00041600
H	-1.46468000	1.58339500	-1.81453100
C	-2.50069600	-0.09532000	0.64824900
H	-1.64633800	0.36756900	0.13766100
H	-3.30931600	-0.16494700	-0.08713000
C	-1.32209700	-3.46933200	-2.88233600
C	-3.83597200	4.34250000	1.12463600
H	-4.74320900	4.12513200	0.56039200
H	-3.99119400	5.25347900	1.71366700
H	-3.63776500	3.51710300	1.81464000
C	-2.64054900	4.53950700	0.19884400
C	-3.10735900	2.70669700	-1.31473700
C	-3.16596700	0.94678400	-2.99864500
H	-4.21618600	1.23756900	-2.92185800
C	-3.16603200	-0.54833000	-2.65391100
C	-1.86952000	-2.46335400	-1.83149100
H	-2.89259500	-2.78971100	-1.62314000
C	-1.03095400	-2.43199300	-0.54155100
H	-0.15100800	-1.78353100	-0.67440800
H	-0.68265800	-3.43262800	-0.26286500
C	-2.92401800	0.68010300	1.87757100
C	-1.96050400	1.39637400	2.62131400
C	-2.35548100	2.07646700	3.77276700
C	-0.81396100	-1.91341700	2.33064600
C	-1.30408100	-1.63389700	3.61534400
H	-2.35966100	-1.42455400	3.77113500
C	-0.42833800	-1.60967100	4.69349300
H	-0.80515000	-1.38280200	5.68569100
C	0.92945900	-1.86809100	4.49809100
H	1.61230400	-1.83977600	5.34208100
C	1.41318100	-2.16395100	3.22694300
H	2.47050500	-2.35460500	3.06834200
C	0.54433300	-2.18706500	2.13775900
H	0.94993200	-2.38217000	1.14783700
C	-3.31340600	-2.96371600	1.21683400
C	-4.43986000	-2.93198100	0.38144300
H	-4.54167200	-2.17853400	-0.39799400
C	-5.44452900	-3.88140400	0.54295800
H	-6.31442000	-3.85317900	-0.10513100
C	-5.33372200	-4.85883400	1.53008800
H	-6.12143600	-5.59565500	1.65332200
C	-4.21345800	-4.89545000	2.35542200
H	-4.12213200	-5.65775400	3.12246300
C	-3.20044600	-3.95417900	2.19876400
H	-2.32618300	-3.99428500	2.84063100
C	-2.70397200	1.25879800	-4.44925000
C	-3.54566400	0.42897200	-5.42285300
H	-4.61468800	0.63242600	-5.29259800
H	-3.39297800	-0.64646800	-5.27946700
H	-3.27597200	0.67199600	-6.45659300
C	-2.95360000	2.74986300	-4.70229200
H	-2.71080900	3.00177400	-5.74072800
H	-2.33186300	3.37222800	-4.05008300
H	-4.00195500	3.01179400	-4.52171200
C	-1.21612900	0.95819000	-4.65564100
H	-1.00501200	-0.11551000	-4.59890900
H	-0.59368100	1.47045800	-3.91225000
H	-0.90017700	1.30481100	-5.64600400

C	-1.39992500	4.89793700	1.00753500
H	-1.20513000	4.12678400	1.76060800
H	-1.54823900	5.85414200	1.51873500
H	-0.52887200	4.98153200	0.35008600
C	-2.90161400	5.59887800	-0.86829700
H	-2.03883900	5.67934400	-1.53848200
H	-3.05479900	6.57123000	-0.38905600
H	-3.78794900	5.35536900	-1.45649900
C	-2.14582000	-3.31425900	-4.16509400
H	-1.96931700	-2.33960000	-4.63090300
H	-3.21907500	-3.40163400	-3.96357800
H	-1.86343800	-4.08778300	-4.88767500
C	0.15280400	-3.21216400	-3.18997700
H	0.49447400	-3.88064400	-3.98827700
H	0.79312200	-3.38351800	-2.31678400
H	0.30806400	-2.18051700	-3.52990700
C	-1.51003100	-4.89629600	-2.35390800
H	-2.55757300	-5.08386400	-2.08741900
H	-0.89102200	-5.10806800	-1.47559400
H	-1.22670400	-5.61992400	-3.12585500
C	-4.27704600	0.73906900	2.26272000
C	-4.62540300	1.43505300	3.42425000
H	-5.67367300	1.47400600	3.71265900
C	-3.68124000	2.09409600	4.20496900
C	-0.50848600	1.45366600	2.22225700
H	-0.00919100	2.27867100	2.73964400
H	0.01613400	0.53691200	2.51113000
H	-0.36717600	1.59413300	1.14389200
C	-5.38722300	0.11407200	1.45391800
H	-5.28874500	0.32620200	0.38326300
H	-5.42864200	-0.97163100	1.58004900
H	-6.35165500	0.51622800	1.77431100
C	-4.07784700	2.85934300	5.44010000
H	-5.11359500	2.65156700	5.72159100
H	-3.43503900	2.60475100	6.28894600
H	-3.98557500	3.93877500	5.27427800
S	1.60916300	0.69312800	-1.49314600
O	0.07362800	0.57900000	-1.31513100
N	2.24810000	1.35446600	-0.10754200
C	5.46003100	-4.83003500	-1.53346000
C	5.26184500	-3.81440300	-2.46502300
C	4.55982400	-2.66486200	-2.10940600
C	4.05361600	-2.50507500	-0.81451400
C	3.28063100	-1.28285200	-0.43352200
C	2.26191700	-0.91096700	-1.33901200
C	4.95194600	-4.68705700	-0.24295800
C	4.25619700	-3.53638300	0.11065400
C	3.50104500	-0.59995100	0.75804600
C	4.68984400	-0.68636300	1.61321100
C	5.97236900	-0.98555000	1.10931100
C	7.08139700	-1.06226800	1.94208200
C	6.96471600	-0.81986900	3.31069900
C	5.71354000	-0.49625700	3.82885100
C	4.59965700	-0.43462400	2.99762800
C	2.44503100	0.41818900	1.03462300
H	6.00679900	-5.72679900	-1.80975100
H	5.65736700	-3.91482100	-3.47181700
H	4.41127000	-1.86983500	-2.83573800
H	2.10386700	-1.49142200	-2.24025300
H	5.09779300	-5.47517500	0.49024900
H	3.86654100	-3.42086500	1.11872100
H	6.09756800	-1.14117200	0.04200700
H	8.05291100	-1.29355200	1.51297500
H	7.83536600	-0.87040700	3.95750100
H	5.59932000	-0.29603300	4.89125600
H	3.63011200	-0.19987500	3.43182000
H	1.46444200	-0.06348200	1.14978400
H	2.64013700	1.03138700	1.91810800
C	2.87569000	2.63301700	-0.14384500
C	4.15654800	2.81970600	-0.75303000
C	2.24375000	3.68804400	0.47267000
C	4.86721400	1.76100500	-1.37849400
C	4.74211600	4.11881900	-0.72048400
C	2.83576100	4.97011000	0.51186900
H	1.27168100	3.51394200	0.92435700
C	6.09587900	1.98564200	-1.94637300
H	4.42856300	0.76787200	-1.38677900
C	6.01333000	4.31647700	-1.32321400
C	4.05546900	5.18201400	-0.07706700
H	2.31427600	5.78485000	1.00563900
C	6.67567000	3.27645400	-1.92205900
H	6.63059800	1.16492500	-2.41559900
H	6.45212800	5.31077700	-1.29434200
H	4.51844100	6.16540800	-0.05704300
H	7.64893800	3.43745800	-2.37608600

IM-B1-S

P	0.90573500	-2.39216600	-1.23733500

O	-0.81021500	-0.16689100	3.08278500	H	-1.54706300	-6.65050200	-1.09484000
N	-2.18689800	-1.52644500	-0.98235600	C	0.29650200	-6.84536900	-2.19877900
H	-1.88381600	-0.62323100	-0.61594200	H	0.13522500	-7.89172300	-2.43940400
H	4.54458300	-0.70296200	2.60165500	C	1.44058200	-6.19556600	-2.65404400
O	-3.35800300	-3.43834600	-0.61806300	H	2.17341300	-6.73061200	-3.24952400
O	-1.78567900	-2.19798200	3.45706900	C	1.65135900	-4.85397500	-2.34850800
N	-2.37510500	-0.88314800	1.68458100	H	2.54193600	-4.35255500	-2.71406300
H	-2.22047700	0.03464500	1.27102700	C	-4.80323600	-1.22657400	1.19449500
C	0.55970600	-2.00946600	0.53094400	C	-5.81364000	-2.32653600	0.85562700
H	0.21890000	-0.96781000	0.53499200	H	-5.73254500	-3.16690100	1.55495200
H	-0.30347700	-2.62208800	0.80979600	H	-5.66853100	-2.71798900	-0.15468200
C	-2.52838300	-1.19378700	-3.43427900	H	-6.83018700	-1.92342700	0.92593400
C	0.65290600	-1.21102900	4.77882300	C	-5.06783700	-0.72599800	2.61863300
H	0.09347800	-2.14378000	4.85464700	H	-6.10890900	-0.39647200	2.70946200
H	1.13552900	-1.00678400	5.74125300	H	-4.42494300	0.12317700	2.86972400
H	1.43566000	-1.32182500	4.02322200	H	-4.89315300	-1.51920500	3.35546700
C	-0.25608900	-0.03808600	4.42546800	C	-4.95427200	-0.06645900	0.20605600
C	-1.66952500	-1.17085100	2.80713800	H	-4.89001100	-0.41043800	-0.83188800
C	-3.35822900	-1.80823900	1.15453600	H	-4.19178100	0.70627900	0.35808100
H	-3.33336200	-2.68306700	1.81027100	H	-5.93062600	0.41375400	0.33473100
C	-2.97306100	-2.33645000	-0.23449800	C	0.56901000	1.23914400	4.32642500
C	-1.69249100	-1.86749800	-2.30506900	H	1.36568800	1.10618100	3.58533700
H	-1.80678800	-2.95121600	-2.42350700	H	1.03023900	1.46715400	5.29226900
C	-0.20542800	-1.48634600	-2.37045600	H	-0.05655000	2.08759000	4.02902200
H	-0.06565700	-0.42076600	-2.14259700	C	-1.39901000	0.12400900	5.42198000
H	0.20109000	-1.68814700	-3.36681800	H	-2.03667500	0.96692700	5.13438200
C	1.72635300	-2.22570000	1.46843000	H	-0.99485400	0.32339700	6.41949400
C	2.72006500	-1.24019200	1.61220400	H	-2.00691800	-0.78274000	5.46870800
C	3.78014500	-1.47107400	2.49657000	C	-3.93166800	-1.80995800	-3.40746700
C	2.58646800	-1.90415800	-1.67441100	H	-4.40682400	-1.69680100	-2.42955400
C	3.67172100	-2.57025600	-1.08735300	H	-3.89616300	-2.88138000	-3.63455800
H	3.50495900	-3.39703400	-0.40156800	H	-4.56939900	-1.32200900	-4.15269400
C	4.96809200	-2.15870800	-1.37452900	C	-2.62474700	0.31976900	-3.21966700
H	5.80672400	-2.66787700	-0.91058100	H	-3.22702500	0.77178200	-4.01592800
C	5.18604400	-1.09243500	-2.24702500	H	-1.64174200	0.80689400	-3.24008400
H	6.19973600	-0.76912400	-2.46505000	H	-3.10019400	0.57040500	-2.26442200
C	4.10952400	-0.43529600	-2.83624000	C	-1.90438900	-1.48657200	-4.80382600
H	4.27530100	0.40054600	-3.50940600	H	-1.68347300	-2.55499200	-4.92345400
C	2.80836100	-0.83309100	-2.54604500	H	-0.98114500	-0.92144500	-4.97220500
H	1.98271800	-0.28225900	-2.98336900	H	-2.60384900	-1.20268000	-5.59729900
C	0.71575600	-4.16291600	-1.57097700	C	1.79066500	-3.40341800	2.24454500
C	-0.44123200	-4.81537000	-1.12418400	C	2.86666000	-3.59103600	3.10731200
H	-1.19886600	-4.29189600	-0.54571900	H	2.90501400	-4.49806400	3.70751000
C	-0.64621900	-6.15412100	-1.44093300	C	3.88060700	-2.63950200	3.24265000

C	2.71894000	0.07107200	0.86083000
H	2.91179800	0.90385500	1.54808600
H	3.52123700	0.08909500	0.11151200
H	1.78200700	0.28174000	0.33923300
C	0.68727600	-4.43194600	2.22442000
H	-0.26868100	-3.98443600	2.52559200
H	0.55403200	-4.88501300	1.23736700
H	0.90919800	-5.23780200	2.92866500
C	5.01791800	-2.86348000	4.20450300
H	5.79320900	-2.10161600	4.08790800
H	4.66433300	-2.82689400	5.24105000
H	5.47771100	-3.84557200	4.05343900
S	-0.41071800	2.14443300	-0.57448700
O	-0.75563800	0.76293600	-0.05109300
N	-1.70076000	2.93496300	-1.02308000
C	5.43871600	2.96398000	-3.74431800
C	4.17617200	3.23265500	-4.26821100
C	3.05276500	3.19528800	-3.44397800
C	3.17177100	2.89057900	-2.08328200
C	1.96860000	2.82248000	-1.21032800
C	0.78975200	2.20381000	-1.74388600
C	5.56777700	2.64883900	-2.39182100
C	4.44478100	2.60191700	-1.57296000
C	1.91766400	3.24921100	0.09601300
C	2.88596400	4.02846000	0.87122600
C	2.93932100	3.89465200	2.27130600
C	3.82324200	4.64545500	3.04153400
C	4.68317400	5.55777900	2.43843300
C	4.63093600	5.72126300	1.05345200
C	3.74163500	4.98230700	0.28527500
C	0.59766300	2.91710000	0.74456900
H	6.31548600	2.99967800	-4.38432700
H	4.06363400	3.48224500	-5.31937700
H	2.07396300	3.42970100	-3.85372000
H	0.54374500	2.04712600	-2.78380400
H	6.54645600	2.43085300	-1.97332500
H	4.54724300	2.34215400	-0.52347700
H	2.27841400	3.18488700	2.76417000
H	3.83783600	4.51225700	4.11991700
H	5.37249400	6.14532300	3.03718300
H	5.27529500	6.44957500	0.56842600
H	3.68866000	5.15502000	-0.78468100
H	0.64391000	2.15291400	1.53127200
H	0.06247400	3.79373700	1.12869500
C	-2.76685500	3.00620500	-0.13236300
C	-4.02810500	3.42837300	-0.69697700
C	-2.72377200	2.73613300	1.23300700
C	-4.15633000	3.69104200	-2.08430800
C	-5.17059800	3.56796300	0.14016000
C	-3.86370700	2.89797100	2.05189500
H	-1.80943700	2.37500800	1.69687700
C	-5.36242400	4.06957000	-2.62218400
H	-3.27474900	3.58539200	-2.70779900
C	-6.40201200	3.96402200	-0.44659600
C	-5.06564700	3.30069300	1.53209000
H	-3.77344200	2.68885100	3.11499300
C	-6.49928500	4.20810600	-1.79325300
H	-5.44568200	4.26575300	-3.68718900
H	-7.27139200	4.06870100	0.19835200
H	-5.94291700	3.41477400	2.16298800
H	-7.44933000	4.50966600	-2.22535000

TS1-S

P	1.59581700	-1.54959100	-1.72556400
O	0.75867400	-0.75085100	3.31107200
N	-1.23001700	-2.51222800	-0.43530400
H	-1.44593900	-1.60245000	-0.01685600
H	5.12012800	1.49115200	1.25550200
O	-0.85675700	-4.73092800	-0.11273800
O	0.96759900	-3.00169200	3.65051700
N	-0.62927900	-2.11989500	2.27003800
H	-1.02557300	-1.23493600	1.95323200
C	1.70817700	-1.50005300	0.11455900
H	0.93213600	-0.78622300	0.42374100
H	1.36744300	-2.48031700	0.45975700
C	-2.44040800	-2.45819700	-2.62151700
C	2.99664400	-0.93343900	4.31565300
H	2.99135500	-2.02344500	4.35235600
H	3.61367000	-0.55247000	5.13708700
H	3.44323600	-0.60595500	3.37284800
C	1.58760400	-0.36704500	4.44658700
C	0.41585400	-2.04470700	3.13175300
C	-1.18202200	-3.39603000	1.85796300
H	-0.53427700	-4.16330300	2.29071800
C	-1.08127500	-3.61078700	0.34127700
C	-1.07489900	-2.52223200	-1.87917700
H	-0.61456100	-3.48032200	-2.14441000
C	-0.14131500	-1.35789800	-2.26098300

H	-0.49902400	-0.42080200	-1.81202500	C	0.90793800	-0.79937000	5.74174600
H	-0.09250200	-1.22771300	-3.34731400	H	-0.11851700	-0.41858500	5.77617200
C	3.07888700	-1.15919600	0.65827400	H	1.45363400	-0.38869000	6.59729700
C	3.51149200	0.17645600	0.72946400	H	0.88737900	-1.88735500	5.83262000
C	4.79709200	0.45241300	1.20900700	C	-3.25238800	-3.69187500	-2.21036300
C	2.55165500	-0.19901000	-2.44248300	H	-3.45113000	-3.69835000	-1.13422700
C	3.95005200	-0.23221600	-2.34434100	H	-2.72506400	-4.61899600	-2.46196200
H	4.45125300	-1.08391000	-1.89122000	H	-4.21778600	-3.69190000	-2.72776100
C	4.69858400	0.83865000	-2.81836000	C	-3.21192900	-1.18956500	-2.25013800
H	5.78035900	0.81530100	-2.73413300	H	-4.17894000	-1.17424300	-2.76516200
C	4.05905200	1.94039000	-3.38606600	H	-2.67445300	-0.27704600	-2.53508500
H	4.64406000	2.78258700	-3.74392700	H	-3.41079300	-1.13074500	-1.17385200
C	2.67105900	1.97172800	-3.49086900	C	-2.21198300	-2.51084700	-4.13694700
H	2.17303100	2.83446300	-3.92340400	H	-1.53692300	-3.33095300	-4.41261100
C	1.91348900	0.90385000	-3.01858900	H	-1.79941900	-1.57612500	-4.53143200
H	0.83131800	0.95120000	-3.08304000	H	-3.16544300	-2.67804800	-4.64882500
C	2.22411400	-3.10613900	-2.40894100	C	3.92415000	-2.19109300	1.12524000
C	1.84074200	-4.32137000	-1.82668900	C	5.19652100	-1.86886500	1.58911600
H	1.21774300	-4.35061700	-0.93622700	H	5.83959800	-2.66826100	1.95219500
C	2.23361500	-5.52224100	-2.40909100	C	5.66148400	-0.55190000	1.62607200
H	1.92928200	-6.45850600	-1.95256400	C	2.66483600	1.35786700	0.32524600
C	3.00239500	-5.51810200	-3.57063800	H	2.60249400	2.07966000	1.14728400
H	3.30903700	-6.45714000	-4.02122000	H	3.11587700	1.88085000	-0.52760300
C	3.37180500	-4.31165600	-4.15958900	H	1.64269800	1.09917700	0.04193200
H	3.96497100	-4.30562100	-5.06830800	C	3.46898300	-3.62697200	1.20580000
C	2.98187900	-3.10519800	-3.58652200	H	2.55623200	-3.71909400	1.80781600
H	3.26968800	-2.17041300	-4.05704600	H	3.27621300	-4.05990500	0.21984500
C	-2.61954100	-3.62424700	2.40937200	H	4.23782500	-4.23756600	1.68593300
C	-3.12261700	-5.00725500	1.98679300	C	7.04644300	-0.24351500	2.13116200
H	-2.44110100	-5.79804800	2.32030400	H	7.22203900	0.83436100	2.17931300
H	-3.22346500	-5.09199000	0.90043500	H	7.19953700	-0.65839600	3.13292800
H	-4.10570300	-5.19268100	2.43366000	H	7.80983500	-0.68184300	1.47874900
C	-2.54568000	-3.55997900	3.93880600	S	-1.75203100	1.54017600	0.21910400
H	-3.53212300	-3.76271000	4.37045900	O	-1.40642500	0.09811900	0.62038300
H	-2.22477900	-2.57068900	4.28166600	N	-3.20030100	1.64724100	-0.43785100
H	-1.84086100	-4.30093000	4.33294900	C	2.02161200	5.62084900	-3.60633100
C	-3.57370400	-2.54271600	1.89835200	C	0.72290200	5.16176200	-3.81988900
H	-3.72160000	-2.60194700	0.81362500	C	0.07864400	4.40571100	-2.84366200
H	-3.20905500	-1.53589700	2.12718600	C	0.71945700	4.10371800	-1.63588200
H	-4.55764500	-2.64934900	2.36861000	C	0.04946300	3.27689800	-0.59528400
C	1.62328200	1.15261600	4.33777500	C	-0.72873600	2.19580800	-1.00634800
H	2.06120100	1.44826100	3.37763100	C	2.66716400	5.32690000	-2.40570300
H	2.23421900	1.57283900	5.14267800	C	2.02069500	4.57674500	-1.42840400
H	0.61382000	1.56975500	4.40987000	C	0.11128200	3.57395100	0.78681000

C	0.32429500	4.91869200	1.35371500
C	0.83822400	5.04152000	2.65638200
C	1.02118500	6.28586000	3.25153600
C	0.71381600	7.44926800	2.55142100
C	0.20716100	7.34875500	1.25603500
C	0.00558200	6.10525100	0.66928300
C	-0.42389400	2.53819400	1.60711600
H	2.52358700	6.21164700	-4.36694200
H	0.20528300	5.40009000	-4.74458100
H	-0.94338800	4.07215800	-3.00253900
H	-1.03163600	2.03491500	-2.03567300
H	3.67667600	5.68659100	-2.22831600
H	2.51895900	4.35248700	-0.48914400
H	1.11226000	4.13966200	3.19981100
H	1.41788500	6.34585900	4.26130200
H	0.86239000	8.42264500	3.00923900
H	-0.05278200	8.24722500	0.70328400
H	-0.42505900	6.04849200	-0.32562800
H	0.06427100	1.56036400	1.63999600
H	-0.88417500	2.80931200	2.55843400
C	-4.31468000	1.20024300	0.24056300
C	-5.54346100	1.14924100	-0.52100400
C	-4.36491600	0.82456400	1.57892900
C	-5.57164300	1.51704100	-1.88945900
C	-6.74835700	0.71768100	0.10172300
C	-5.57020400	0.40951400	2.18191100
H	-3.45904600	0.84546500	2.17919000
C	-6.73645700	1.45542400	-2.61610900
H	-4.64584700	1.85272900	-2.34578500
C	-7.93619200	0.66771700	-0.67627800
C	-6.74281400	0.34734300	1.47339000
H	-5.55553500	0.12995200	3.23271000
C	-7.93423300	1.02465100	-2.00103200
H	-6.74111000	1.74161100	-3.66400200
H	-8.85445700	0.33820900	-0.19544200
H	-7.66817700	0.02160500	1.94015500
H	-8.85285300	0.98055700	-2.57937600

IM-B2-S

P	2.32494500	0.90109400	-1.28298300
O	-1.27798600	2.56641900	2.28693300
N	2.42085000	-0.61043500	1.71889500
H	1.46884700	-0.94896700	1.55174500
H	-1.97753000	4.29540600	-2.04903500
O	3.66738800	0.96445800	2.77961800
O	0.25226000	3.13838800	3.87902500
N	0.27561100	1.08351400	2.88384600
H	-0.18860400	0.46526200	2.21269500
C	1.49277300	1.92671400	-0.00301600
H	0.75934100	1.24110300	0.42971400
H	2.24279200	2.11958600	0.77181200
C	4.26720100	-2.09405300	0.82416300
C	-1.26144600	5.02343000	2.23796700
H	-0.49909300	5.18413400	3.00004700
H	-1.93083400	5.89050000	2.21400900
H	-0.77819200	4.93992800	1.25874100
C	-2.07940100	3.76523100	2.51407200
C	-0.21053900	2.33641800	3.08695500
C	1.44092700	0.62595000	3.62072800
H	1.78154100	1.48672900	4.19966900
C	2.61679200	0.33513200	2.67334900
C	3.39570600	-0.81741700	0.65668500
H	4.09619000	0.02002300	0.73119600
C	2.64059700	-0.78891000	-0.68358300
H	1.67883000	-1.31025700	-0.58842000
H	3.21031200	-1.26732500	-1.48743000
C	0.84609900	3.20038300	-0.50621400
C	-0.43282000	3.16041000	-1.09191100
C	-0.99240400	4.34408400	-1.58670700
C	1.30970400	0.79760400	-2.76707700
C	1.16069700	1.92982700	-3.57939200
H	1.69923900	2.84608800	-3.34957300
C	0.30780500	1.88317600	-4.67577400
H	0.18445800	2.76346300	-5.29847700
C	-0.39241600	0.71166700	-4.96480900
H	-1.06311400	0.67997600	-5.81809200
C	-0.23806800	-0.41434800	-4.16082300
H	-0.78410100	-1.32615800	-4.38469900
C	0.60812900	-0.37811800	-3.05435500
H	0.67984200	-1.25510200	-2.41250600
C	3.94691800	1.58223500	-1.72764500
C	4.81534300	2.02800900	-0.72090900
H	4.50117600	2.06092200	0.31987600
C	6.10639500	2.43202900	-1.04754500
H	6.77170800	2.77817800	-0.26314600
C	6.53940600	2.39037500	-2.37067500
H	7.54714500	2.70668000	-2.62149600
C	5.68406000	1.93627700	-3.37085500

H	6.02033200	1.89669300	-4.40191900	C	2.81959800	4.57966700	0.36763300
C	4.39258600	1.52671700	-3.05334300	H	2.81097600	4.06678800	1.33634600
H	3.73702300	1.16495000	-3.83944800	H	3.65428800	4.18092100	-0.21635300
C	1.11142100	-0.50752100	4.62948300	H	3.03176500	5.63405800	0.56139700
C	2.39120500	-0.85639700	5.39440000	C	-0.95918700	6.83444300	-2.02251400
H	2.79864200	0.02271000	5.90680600	H	-1.86265200	6.62458600	-2.60151600
H	3.16807400	-1.24409400	4.72569700	H	-1.23579200	7.50300500	-1.19942400
H	2.18723400	-1.62504800	6.14804100	H	-0.26209400	7.38284600	-2.66458400
C	0.06339900	0.03989700	5.60580500	S	-1.16741200	-1.88402100	0.32333500
H	-0.13854800	-0.69502100	6.39298200	O	-0.23548900	-0.69715100	0.71947800
H	-0.88078400	0.25354900	5.09308200	N	-0.33665100	-2.89582400	-0.64970400
H	0.40815700	0.96527500	6.08078100	C	-5.75668900	0.56038900	-4.13513300
C	0.54987200	-1.76235000	3.95027900	C	-4.89365900	-0.52596800	-4.26344800
H	1.30847100	-2.28529300	3.35787700	C	-4.15129300	-0.96149600	-3.17122200
H	-0.29594300	-1.52065700	3.29394200	C	-4.25516000	-0.31295800	-1.93440400
H	0.18710600	-2.46413400	4.71032600	C	-3.43808900	-0.74687300	-0.76937700
C	-3.20106900	3.65225700	1.48836100	C	-2.19534000	-1.21445400	-0.96217000
H	-2.79748400	3.72522200	0.47424400	C	-5.87652400	1.20470700	-2.90577600
H	-3.91338500	4.47080200	1.63242100	C	-5.13540500	0.76859300	-1.81214900
H	-3.73739500	2.70332000	1.59279500	C	-4.03520900	-0.61333800	0.59037800
C	-2.65544700	3.73901400	3.92693300	C	-5.34780100	-1.27010900	0.82612900
H	-3.21439800	2.81066900	4.08901500	C	-6.28422900	-0.73081900	1.71611000
H	-3.34598500	4.57883600	4.05560600	C	-7.49019900	-1.38145200	1.95507500
H	-1.86823700	3.81562100	4.67828400	C	-7.78360700	-2.57581800	1.30014700
C	4.76332900	-2.15513600	2.27218000	C	-6.86488000	-3.11336000	0.40171900
H	3.94181100	-2.39723300	2.95373100	C	-5.65898100	-2.46280500	0.16299100
H	5.19792400	-1.20016000	2.58805500	C	-3.38269700	0.06673400	1.54344000
H	5.52468500	-2.93606400	2.37543700	H	-6.34264900	0.89443900	-4.98611200
C	3.47893000	-3.36332400	0.50256900	H	-4.81009600	-1.04613800	-5.21327600
H	4.08442500	-4.25095000	0.71803900	H	-3.50691500	-1.83210400	-3.26227700
H	3.18809700	-3.41519800	-0.55267300	H	-1.73293700	-1.25493100	-1.94549000
H	2.56725400	-3.43008400	1.10802200	H	-6.55339700	2.04677900	-2.79644200
C	5.48093900	-1.97134400	-0.10544600	H	-5.23153100	1.27136400	-0.85269300
H	6.08422000	-1.09017000	0.14543700	H	-6.07055400	0.21361100	2.20997800
H	5.19870600	-1.89666000	-1.16210700	H	-8.20723800	-0.95017600	2.64737100
H	6.12041200	-2.85498100	-0.00495900	H	-8.72683600	-3.08138000	1.48409800
C	1.50407800	4.44185900	-0.35801500	H	-7.08605200	-4.04242100	-0.11502300
C	0.90577200	5.59389400	-0.86330800	H	-4.94063700	-2.88949900	-0.53256300
H	1.41838600	6.54634500	-0.74255900	H	-2.44076300	0.56737000	1.33828700
C	-0.33740300	5.56654200	-1.49845900	H	-3.77085000	0.13491800	2.55731100
C	-1.25772400	1.90214100	-1.21091900	C	0.12490600	-4.05262000	-0.04655800
H	-2.24357400	2.05290400	-0.75530100	C	0.80477100	-4.99033900	-0.91778600
H	-1.43163700	1.64659300	-2.26418600	C	0.03211800	-4.39360200	1.30379100
H	-0.81369500	1.03504100	-0.72163000	C	0.93176700	-4.73507800	-2.30625500

C	1.38074100	-6.17855400	-0.38465700
C	0.59260600	-5.58308600	1.81027200
H	-0.47125600	-3.72815700	1.99905900
C	1.60330100	-5.60399700	-3.13317900
H	0.47320000	-3.83486700	-2.70187600
C	2.06824500	-7.05716900	-1.26491800
C	1.26606000	-6.46333400	1.00176200
H	0.49359200	-5.78926800	2.87342100
C	2.18195000	-6.78000500	-2.60385100
H	1.68698200	-5.39264500	-4.19551100
H	2.50521200	-7.96307500	-0.85101800
H	1.70902900	-7.37231800	1.39872100
H	2.71295700	-7.46434500	-3.25978000

IM-B3-S

P	2.42527300	0.74004400	-1.67514800
O	1.95845700	-0.95600500	3.25534300
N	1.29826900	-2.35770600	-1.25098500
H	0.51365600	-2.03006700	-0.67263200
O	3.31541900	-3.40192000	-1.29895400
O	3.53366000	-2.58783800	2.98606100
N	1.71347800	-2.41998000	1.61108500
H	0.86878500	-1.87218600	1.41185800
C	2.74770600	0.18020200	0.04709500
H	1.75818800	-0.11264400	0.41706100
H	3.34921200	-0.73238200	-0.03623500
C	0.52265000	-2.44938400	-3.64710900
C	3.88137200	-0.05122200	4.48057800
H	4.56977800	-0.86587800	4.25272600
H	4.16656600	0.40003700	5.43767600
H	3.96197600	0.71517300	3.70314100
C	2.44270100	-0.54974000	4.56703600
C	2.49775500	-2.03989400	2.65043000
C	2.08445100	-3.53618000	0.76347100
H	3.07778600	-3.85386300	1.08926000
C	2.28749600	-3.09680500	-0.69452500
C	1.42623900	-1.77326800	-2.57444500
H	2.45975100	-1.94720500	-2.88876700
C	1.12512800	-0.27005900	-2.45381400
H	0.23089500	-0.14169400	-1.82985500
H	0.95225900	0.18697800	-3.43430800
C	1.83564700	2.44913300	-1.68582000
C	2.67805100	3.47766500	-1.24105700
H	3.69584100	3.26149100	-0.92617400
C	2.20404400	4.78439100	-1.19127400
H	2.85692400	5.57650600	-0.83898700
C	0.89807700	5.07198300	-1.58399200
H	0.52958900	6.09230900	-1.53574200
C	0.06177300	4.05301000	-2.03371900
H	-0.96115100	4.26732900	-2.32826900
C	0.52574900	2.74188400	-2.08471800
H	-0.15089600	1.96133200	-2.41812000
C	3.91366700	0.62254200	-2.69830700
C	4.66143900	-0.56468600	-2.68448200
H	4.39557500	-1.39185300	-2.02703900
C	5.75949600	-0.69373000	-3.52826800
H	6.33648900	-1.61258300	-3.51600500
C	6.11686100	0.35068200	-4.37931100
H	6.97788400	0.24633200	-5.03244800
C	5.37124200	1.52610200	-4.39736200
H	5.64648800	2.33939800	-5.06107300
C	4.26617100	1.66403000	-3.56256300
H	3.68501200	2.58053100	-3.58622600
C	1.12689000	-4.75052500	0.92662900
C	1.55062800	-5.84956200	-0.05139700
H	2.60830000	-6.10771000	0.07561800
H	1.40414100	-5.54351600	-1.09308400
H	0.95583900	-6.75393200	0.11847800
C	1.27389600	-5.26180000	2.36375200
H	0.61916900	-6.12540100	2.52615000
H	0.99624900	-4.48860700	3.08840600
H	2.30500000	-5.56873000	2.57239800
C	-0.33583500	-4.36525200	0.68222000
H	-0.50332100	-3.98887700	-0.33238600
H	-0.67406200	-3.59910900	1.38994600
H	-0.98162700	-5.24019600	0.81872800
C	1.51081500	0.59675700	4.94129000
H	1.64689400	1.43869500	4.25561700
H	1.73128100	0.93999300	5.95686600
H	0.46604100	0.27276000	4.90043200
C	2.28586400	-1.70101900	5.55650500
H	1.24877300	-2.05364500	5.56327700
H	2.53499400	-1.35262000	6.56408400
H	2.94376700	-2.53370300	5.30250900
C	0.61039400	-3.96793900	-3.46567200
H	0.07554000	-4.28729400	-2.56570200
H	1.65254200	-4.29814300	-3.37900400
H	0.15730600	-4.47481200	-4.32484000

C	-0.93639700	-1.99958800	-3.52024500
H	-1.57945100	-2.63890600	-4.13611600
H	-1.07308500	-0.97036000	-3.87555400
H	-1.30317500	-2.04777300	-2.48566800
C	1.06619400	-2.09101500	-5.03531400
H	2.07909700	-2.48665800	-5.17619100
H	1.10033800	-1.00843700	-5.20609800
H	0.42524600	-2.52083200	-5.81276200
S	-1.81675500	-0.84712900	0.79173400
O	-0.30357500	-0.82214800	0.41388100
N	-2.59899900	-1.50845500	-0.43751400
C	-3.39539900	5.70845000	1.95479700
C	-4.46024300	4.81092600	1.89352500
C	-4.28865400	3.55569900	1.31730800
C	-3.04527500	3.18002700	0.79640700
C	-2.83653600	1.82529000	0.18891000
C	-2.09827500	0.94369200	0.88910400
C	-2.15409400	5.34085200	1.44358200
C	-1.98126100	4.08293500	0.86910300
C	-3.42794000	1.53908700	-1.14109200
C	-4.79451700	2.04277600	-1.47782700
C	-5.04174300	3.39544200	-1.73838200
C	-6.32189200	3.82734900	-2.07120500
C	-7.37209200	2.91383300	-2.14541400
C	-7.13490300	1.56588200	-1.89033500
C	-5.85281300	1.13226200	-1.55902400
C	-2.76036500	0.77867000	-2.01691200
C	-3.55621700	-2.48458600	-0.15649600
C	-4.69779700	-2.28181000	0.70637300
C	-3.46313100	-3.70163500	-0.82170700
C	-4.98137800	-1.02627700	1.30758000
C	-5.61095100	-3.35696100	0.93633700
C	-4.38743100	-4.74206600	-0.61210600
C	-6.06276900	-0.85252700	2.13536300
C	-6.70736100	-3.15228500	1.81872100
C	-5.43050600	-4.59348600	0.26878100
C	-6.92684200	-1.93699800	2.41318800
H	-3.53366700	6.68784400	2.40319200
H	-5.42958000	5.08844600	2.29710200
H	-5.12291000	2.86059800	1.26779400
H	-1.67255700	1.28112300	1.83470900
H	-1.31620900	6.03114900	1.49036400
H	-1.01304900	3.79282700	0.47017900
H	-4.22521200	4.11052100	-1.67852500
H	-6.49998800	4.87929400	-2.27490700
H	-8.37117100	3.25249200	-2.40374000
H	-7.94707700	0.84722100	-1.94813300
H	-5.66081700	0.08178300	-1.35600000
H	-1.77161300	0.39952400	-1.78389200
H	-3.19106800	0.50493900	-2.97681100
H	-2.63037500	-3.84472200	-1.50422000
H	-4.34955600	-0.17601900	1.07724200
H	-4.25366500	-5.68098200	-1.14326900
H	-6.25943900	0.12269700	2.57132100
H	-7.38088100	-3.98597400	2.00377600
H	-6.13418100	-5.40110100	0.45134900
H	-7.77235400	-1.79879100	3.08112200
C	3.39179000	1.22304200	0.93054600
C	4.78853000	1.31971500	1.05948300
C	2.55795900	2.10528400	1.64740800
C	3.13701200	3.10830000	2.42334300
C	4.52061200	3.25050000	2.53070800
H	2.48674300	3.78952600	2.96952600
C	5.32514400	2.33818600	1.85275100
H	6.40665000	2.40697900	1.95233100
C	1.05919100	1.95812500	1.62525400
H	0.58834300	2.73218800	2.23881500
H	0.63845200	2.02900400	0.61576300
H	0.76365300	0.97646300	2.01327000
C	5.73614500	0.34913500	0.39875600
H	5.40017900	-0.68818100	0.50625300
H	5.85780300	0.55267000	-0.66979600
H	6.72489800	0.42055800	0.85911100
C	5.12174100	4.31501000	3.41054100
H	6.13394600	4.57535100	3.08856800
H	4.51414300	5.22471100	3.40717100
H	5.18479600	3.96695800	4.44825200

TS2-S

P	2.40311300	0.73955000	-1.70906200
O	2.24744800	-0.86462400	3.26599800
N	1.33116300	-2.37862000	-1.15828600
H	0.57575200	-2.03194900	-0.55480500
O	3.33840900	-3.43805400	-1.25836000
O	3.80497400	-2.49704600	2.91003600
N	1.87669800	-2.37285500	1.68657300
H	1.01946000	-1.83004700	1.53829100
C	2.83933600	0.23218300	0.00532400

H	1.87517400	-0.05708600	0.44034200	C	1.36291900	-5.16163000	2.53151600
H	3.44185100	-0.67935000	-0.08634700	H	0.70350800	-6.01243400	2.73624100
C	0.51547600	-2.52764900	-3.53782900	H	1.10773800	-4.36131200	3.23436500
C	4.25003000	0.10081200	4.30079100	H	2.39481300	-5.47274800	2.73021500
H	4.92121200	-0.69945700	3.98643200	C	-0.26795200	-4.28891400	0.85873000
H	4.62582200	0.53534400	5.23382300	H	-0.46176100	-3.97665000	-0.17306600
H	4.24109500	0.88483100	3.53702700	H	-0.56460200	-3.46885800	1.52268600
C	2.83507400	-0.42227500	4.52294600	H	-0.92743000	-5.13733600	1.07423400
C	2.74162000	-1.96158800	2.64773400	C	1.91580600	0.71400600	4.95506300
C	2.17888800	-3.51748900	0.85021600	H	1.95920400	1.53592000	4.23388500
H	3.17523900	-3.85652200	1.14353700	H	2.23023400	1.09550400	5.93142400
C	2.33599600	-3.11371000	-0.62351600	H	0.88120700	0.36512100	5.03074900
C	1.41949400	-1.81248000	-2.49252800	C	2.78792100	-1.55454900	5.54541500
H	2.45016100	-1.96879000	-2.82489600	H	1.76656000	-1.94095800	5.63259300
C	1.08409500	-0.31547900	-2.38553200	H	3.09361600	-1.17324400	6.52505500
H	0.22356000	-0.19258100	-1.71485400	H	3.45420400	-2.37174700	5.26495500
H	0.84601000	0.11729400	-3.36345200	C	0.66338900	-4.04093400	-3.35060300
C	1.78426400	2.43614000	-1.72651300	H	0.16390300	-4.37234100	-2.43476100
C	2.64204500	3.48604300	-1.36960400	H	1.71836600	-4.33276300	-3.28833800
H	3.68448700	3.28937800	-1.13243000	H	0.20558300	-4.56960600	-4.19401000
C	2.15112900	4.78548700	-1.30758800	C	-0.95378300	-2.13068800	-3.36839600
H	2.81420000	5.59592800	-1.02264100	H	-1.59561400	-2.79952300	-3.95362300
C	0.81268000	5.04275900	-1.60049300	H	-1.14179700	-1.11048100	-3.72497100
H	0.43137000	6.05784700	-1.54297100	H	-1.27433600	-2.18232600	-2.31923100
C	-0.03842500	4.00287200	-1.96651700	C	1.00632000	-2.15674300	-4.94190300
H	-1.08295700	4.19723700	-2.18999400	H	2.03177100	-2.50873500	-5.10627600
C	0.44192700	2.69789900	-2.02762600	H	0.98861300	-1.07518200	-5.12091600
H	-0.24246200	1.89934200	-2.29600700	H	0.36515300	-2.62005400	-5.69966300
C	3.81904100	0.61652700	-2.83059500	S	-1.69348200	-0.65457000	0.90144800
C	4.60402700	-0.54612200	-2.82281900	O	-0.18578300	-0.79656000	0.55359300
H	4.42195500	-1.34354200	-2.10368600	N	-2.53724400	-1.36175000	-0.28079600
C	5.62957900	-0.68935700	-3.75145600	C	-4.06209800	5.52839900	1.94744000
H	6.23556400	-1.58934500	-3.74261500	C	-4.99872300	4.50573200	1.80519500
C	5.87535900	0.31643500	-4.68463600	C	-4.64329300	3.30677400	1.19556000
H	6.67775900	0.19995500	-5.40655100	C	-3.34116000	3.11034300	0.71879200
C	5.09196700	1.46712700	-4.69735300	C	-2.93123800	1.80225500	0.12142700
H	5.27918900	2.24901000	-5.42623400	C	-1.94625300	1.11262700	0.77505300
C	4.06064800	1.61936900	-3.77494600	C	-2.76326600	5.33990100	1.48344200
H	3.44911700	2.51615400	-3.79250100	C	-2.40563500	4.13807700	0.87613800
C	1.19312700	-4.69772900	1.08072100	C	-3.57809900	1.23412900	-1.04249000
C	1.56698200	-5.84157000	0.13385600	C	-4.96333900	1.57431800	-1.46917600
H	2.61589000	-6.13380900	0.26006800	C	-5.31549700	2.88543400	-1.81989600
H	1.42230500	-5.56425600	-0.91616500	C	-6.60060500	3.18791600	-2.25757600
H	0.94231800	-6.71841800	0.33752000	C	-7.56072100	2.18300700	-2.36493100

C	-7.22170200	0.87460200	-2.03124100
C	-5.93725500	0.57392600	-1.58400500
C	-2.89061600	0.23743800	-1.68579600
C	-3.55084700	-2.28283300	0.02936100
C	-4.67176700	-2.01361100	0.89757300
C	-3.51648100	-3.49993500	-0.63655400
C	-4.85891000	-0.76501400	1.55009700
C	-5.66238400	-3.02602200	1.09028400
C	-4.50800100	-4.48225700	-0.45143400
C	-5.93537700	-0.54035700	2.37185100
C	-6.75999300	-2.76470400	1.95579000
C	-5.55641200	-4.26300700	0.40628100
C	-6.89484300	-1.55651600	2.58762800
H	-4.34328900	6.46385700	2.42213500
H	-6.01134900	4.64023500	2.17417100
H	-5.37758800	2.51271100	1.08766500
H	-1.47165400	1.57839100	1.63688000
H	-2.02390600	6.12817300	1.59475900
H	-1.38967100	3.98707900	0.51854600
H	-4.56976500	3.67291300	-1.74895800
H	-6.85096700	4.21150800	-2.52214600
H	-8.56425800	2.41897800	-2.70674900
H	-7.96126000	0.08270300	-2.10908400
H	-5.68181700	-0.44656100	-1.30888900
H	-1.80512300	0.24149900	-1.64817100
H	-3.34030600	-0.31781400	-2.50546500
H	-2.68214600	-3.69047600	-1.30498500
H	-4.14866200	0.03583900	1.38305000
H	-4.42695300	-5.42452000	-0.98646100
H	-6.05229800	0.42563600	2.85442600
H	-7.49909700	-3.54908100	2.10021900
H	-6.32015800	-5.01959400	0.56471900
H	-7.74061400	-1.37182500	3.24363900
C	3.52472400	1.30494600	0.82080400
C	4.92781700	1.40870500	0.86835600
C	2.73071600	2.20315400	1.55991600
C	3.35061400	3.23050600	2.27373700
C	4.73539000	3.38454500	2.29052600
H	2.72994000	3.92207400	2.84024200
C	5.50449200	2.45070800	1.59712500
H	6.58981900	2.52270700	1.63614600
C	1.23292300	2.05741000	1.62503500
H	0.80045400	2.83264100	2.26493800
H	0.75733600	2.13449800	0.64057800
H	0.95447700	1.07692400	2.02917700
C	5.83734800	0.41168400	0.19437400
H	5.52317600	-0.62071400	0.38446800
H	5.87688500	0.55597200	-0.88968600
H	6.85600800	0.51705500	0.57606000
C	5.38978900	4.51105800	3.04676000
H	5.76645900	5.27812600	2.36030700
H	4.68559400	4.99193100	3.73090500
H	6.24239300	4.15185300	3.63145300

IM-B4-S

P	-2.29726100	-1.17060200	-1.51374900
O	-2.25152000	1.46799200	3.04575000
N	-1.51625600	2.06478000	-1.62333300
H	-0.72443400	1.95708700	-0.98312000
O	-3.62839200	2.85273100	-1.90722500
O	-3.93814600	2.87389600	2.42006000
N	-2.05169700	2.60752000	1.15531900
H	-1.14934800	2.12617000	1.10001300
C	-2.78842700	-0.32238400	0.04384800
H	-1.84346600	0.10073200	0.40722000
H	-3.42667500	0.51528900	-0.25809100
C	-0.66226700	1.82141100	-3.96434000
C	-4.17309300	0.65191100	4.33893100
H	-4.90766700	1.37063700	3.97512500
H	-4.46015100	0.32444000	5.34448800
H	-4.17175800	-0.22365800	3.68170400
C	-2.77550800	1.26061100	4.38947800
C	-2.84790600	2.36315900	2.22902000
C	-2.48223200	3.51239100	0.10716400
H	-3.50856400	3.79429700	0.35301400
C	-2.59656000	2.78664300	-1.24116200
C	-1.51380500	1.22472200	-2.80895200
H	-2.54467300	1.20130500	-3.17472100
C	-1.04297300	-0.18164500	-2.39164600
H	-0.19226600	-0.09519700	-1.70105500
H	-0.74800300	-0.78542500	-3.25622200
C	-1.57525700	-2.78533100	-1.16109700
C	-2.38278000	-3.79172800	-0.61182300
H	-3.44200900	-3.61723100	-0.43997200
C	-1.82209200	-5.01871300	-0.27759600
H	-2.44683600	-5.79289400	0.15611400
C	-0.46416200	-5.24845900	-0.49505600
H	-0.02779200	-6.20729500	-0.23107400

C	0.33650100	-4.25313800	-1.04836700
H	1.39504500	-4.42743300	-1.21264700
C	-0.21131800	-3.01566400	-1.37515900
H	0.43925300	-2.24187800	-1.76903300
C	-3.70128000	-1.39509000	-2.63609200
C	-4.57363200	-0.32398100	-2.88052300
H	-4.46721700	0.62259100	-2.35291000
C	-5.59005500	-0.46423100	-3.82014000
H	-6.26292400	0.36668300	-4.00581100
C	-5.74292400	-1.66269100	-4.51449100
H	-6.53962300	-1.76794000	-5.24448400
C	-4.87349900	-2.72374500	-4.27803900
H	-4.98823700	-3.65738300	-4.81929600
C	-3.84963700	-2.59235200	-3.34453500
H	-3.17058000	-3.42106400	-3.17053200
C	-1.63828300	4.81651000	0.05315600
C	-2.21511900	5.72700700	-1.03447400
H	-3.26663300	5.96299100	-0.83483100
H	-2.16102000	5.26254400	-2.02550200
H	-1.65640300	6.66872400	-1.07395000
C	-1.76677000	5.50578100	1.41641700
H	-1.25358300	6.47379300	1.39761100
H	-1.31633200	4.90108000	2.21054400
H	-2.81727900	5.67937800	1.67542500
C	-0.15567800	4.54133600	-0.22879800
H	0.00675400	4.19132400	-1.25408400
H	0.26015100	3.79154200	0.45503100
H	0.42326000	5.46282200	-0.09894400
C	-1.79296100	0.26559000	4.99574300
H	-1.86270500	-0.69975300	4.48501900
H	-2.02451100	0.11201100	6.05424100
H	-0.76699800	0.63755300	4.91196200
C	-2.74066400	2.57676700	5.16045900
H	-1.72692200	2.99170700	5.15308500
H	-3.02995000	2.39803100	6.20114800
H	-3.42771800	3.30604200	4.72801700
C	-1.09844300	3.27564500	-4.17520600
H	-0.78765400	3.90767200	-3.33794700
H	-2.18726400	3.35502600	-4.27235300
H	-0.64032200	3.67645200	-5.08597800
C	0.83006700	1.77350000	-3.62676900
H	1.40179700	2.38253400	-4.33617200
H	1.22382200	0.75288900	-3.67981200
H	1.02634900	2.15824300	-2.61607200
C	-0.94015200	1.04141300	-5.25442000
H	-2.00766800	1.06406500	-5.50543300
H	-0.62719800	-0.00648500	-5.19292700
H	-0.39077900	1.49108500	-6.08852600
S	1.61693800	0.80397400	0.89977500
O	0.20626300	1.04011800	0.30440500
N	2.71039900	1.26676000	-0.28677000
C	4.63424800	-4.85289600	2.50980100
C	5.47657600	-3.82618500	2.08577300
C	4.96890800	-2.75595200	1.35655800
C	3.60606700	-2.68933000	1.03756900
C	3.05156700	-1.51123300	0.30325800
C	1.92959700	-0.90334000	0.93690100
C	3.27627000	-4.79215000	2.20840200
C	2.76678500	-3.71632200	1.48488800
C	3.65178600	-0.97828100	-0.81983600
C	4.80285000	-1.51600900	-1.55841200
C	4.97427700	-2.89456700	-1.79077100
C	6.05223400	-3.37963800	-2.52092800
C	6.99254400	-2.50500500	-3.06438200
C	6.83791000	-1.13636400	-2.85586800
C	5.76620300	-0.65201800	-2.11341500
C	2.96260300	0.24564800	-1.33217100
C	3.68719200	2.27327000	-0.01579300
C	4.70114800	2.11976100	0.98590400
C	3.66841900	3.40293600	-0.80407900
C	4.80573900	0.96871300	1.81296500
C	5.65848800	3.16503400	1.15168500
C	4.63075200	4.42484800	-0.64417200
C	5.79751100	0.86711200	2.75567500
C	6.66910700	3.02903800	2.14154300
C	5.59974500	4.31194300	0.31822900
C	6.74018600	1.90801900	2.92636200
H	5.03329700	-5.69007000	3.07525000
H	6.53585400	-3.85757000	2.32485800
H	5.62861900	-1.95792100	1.02679300
H	1.60874500	-1.30973900	1.89124500
H	2.60847400	-5.58288700	2.53980700
H	1.70459600	-3.66489500	1.25796700
H	4.24052300	-3.59018800	-1.39401200
H	6.15125100	-4.45040100	-2.67951900
H	7.83068800	-2.88414500	-3.64148100
H	7.56416900	-0.43801200	-3.26327600
H	5.68148000	0.41785200	-1.93640800

H	1.95520000	-0.02111100	-1.69559900
H	3.48812000	0.73419300	-2.15674300
H	2.89032700	3.49515500	-1.55665800
H	4.09015700	0.16311400	1.69800600
H	4.58942500	5.30229900	-1.28227700
H	5.85787700	-0.02432900	3.37329900
H	7.38997700	3.83422400	2.25916200
H	6.34108900	5.09487800	0.45558200
H	7.51872900	1.81323300	3.67745700
C	-3.44601900	-1.20692900	1.07801300
C	-4.84705000	-1.32940000	1.15467300
C	-2.63222200	-1.89584900	1.99759400
C	-3.22792100	-2.75361900	2.92479500
C	-4.60814000	-2.93089700	2.98315500
H	-2.59073500	-3.28777200	3.62701200
C	-5.39883200	-2.19373300	2.10174500
H	-6.48223600	-2.28042800	2.16133100
C	-1.14180800	-1.68786600	2.03438300
H	-0.68265000	-2.32837500	2.79360100
H	-0.64834700	-1.89730600	1.08074200
H	-0.90641400	-0.64423800	2.27759200
C	-5.77949400	-0.52798700	0.28040400
H	-5.50188800	0.53212000	0.25321800
H	-5.79822300	-0.89582600	-0.74965400
H	-6.79955400	-0.58764000	0.66824300
C	-5.24025200	-3.86206300	3.98444900
H	-5.73336600	-4.70305000	3.48421800
H	-4.49646100	-4.26980700	4.67401600
H	-6.00320800	-3.34395700	4.57480400

IM-B1-R'

S	-0.52128200	-0.70823800	-1.45858100
O	-0.79482000	-0.76092100	-2.92059700
N	-1.76846700	-1.02658100	-0.52209800
C	5.56089500	-2.64282800	0.50357000
C	5.53231400	-1.52269400	-0.32687100
C	4.32428700	-0.90262300	-0.62445400
C	3.12270000	-1.39331000	-0.09884900
C	1.82503700	-0.76052000	-0.45931800
C	0.80995900	-1.61440700	-0.92359400
C	4.37089900	-3.14079900	1.02777200
C	3.15985300	-2.52263200	0.72466300
C	1.56424600	0.60618500	-0.43117600
C	2.30463400	1.68037100	0.19904000
C	3.27418400	1.48686200	1.21409400
C	3.94262000	2.55396600	1.79639700
C	3.66917400	3.87070600	1.42120200
C	2.69627400	4.09031600	0.44862100
C	2.03134700	3.02533300	-0.14684500
C	0.21846700	0.91612600	-1.04810800
H	6.50553000	-3.12650500	0.73573400
H	6.45560300	-1.13079300	-0.74438800
H	4.29766800	-0.02705800	-1.26672400
H	0.88126100	-2.66391700	-1.16650500
H	4.38306700	-4.01414600	1.67383500
H	2.22781000	-2.90610300	1.13053600
H	3.48048700	0.48319500	1.56846700
H	4.67636200	2.35425800	2.57396600
H	4.18788700	4.70207400	1.88894300
H	2.44940600	5.10511300	0.14525000
H	1.28348400	3.23419700	-0.90716200
H	0.26685600	1.45635700	-2.00220300
H	-0.47340600	1.44455200	-0.38138100
C	-2.88597200	-0.21969700	-0.61469200
C	-3.91310000	-0.45338100	0.38066100
C	-3.10848800	0.80188900	-1.53783800
C	-3.76357400	-1.47359100	1.35267500
C	-5.08697500	0.35109900	0.40226000
C	-4.27768200	1.58964900	-1.49752000
H	-2.39210500	0.97300300	-2.33445300
C	-4.73245400	-1.69668900	2.30199800
H	-2.85786300	-2.06990100	1.32441400
C	-6.06882500	0.09741100	1.39715900
C	-5.25500500	1.38495000	-0.55757400
H	-4.39899400	2.37207400	-2.24286000
C	-5.90032900	-0.90022500	2.32449600
H	-4.60105100	-2.48219100	3.04065800
H	-6.96375000	0.71571200	1.40878900
H	-6.15432200	1.99431200	-0.53325700
H	-6.66251800	-1.07777400	3.07848300

TS1-R'

S	-0.61241900	0.12972500	-1.65072200
O	-1.03853700	0.33435700	-3.07861400
N	-1.75213900	-0.50752400	-0.70741000
C	5.04893300	-3.12954900	0.18799700
C	5.32517300	-1.91241600	-0.43165100
C	4.28780000	-1.05598700	-0.78520200

C	2.95598400	-1.40266400	-0.53014000
C	1.85737100	-0.47947300	-0.92847100
C	0.69199000	-1.00477900	-1.43686300
C	3.72704600	-3.48335800	0.44965300
C	2.68926000	-2.62758000	0.09213500
C	1.94693400	0.94172100	-0.76219200
C	2.62899000	1.59506800	0.35923000
C	2.96058500	0.93380200	1.55945900
C	3.59420300	1.59851400	2.60214000
C	3.90340200	2.95468300	2.50112400
C	3.57249800	3.63288600	1.32986000
C	2.95965900	2.96306800	0.27672700
C	0.93094400	1.62903400	-1.48183200
H	5.85941300	-3.79676000	0.46723300
H	6.35272600	-1.62827800	-0.64077400
H	4.49747600	-0.10472400	-1.26582700
H	0.46758200	-2.06086100	-1.52410200
H	3.50296600	-4.42617400	0.94070100
H	1.65980000	-2.89278000	0.31719100
H	2.69103100	-0.11029100	1.68285600
H	3.82891200	1.05458500	3.51359900
H	4.39172000	3.47249800	3.32145300
H	3.80751900	4.68946500	1.22771700
H	2.74403000	3.49694600	-0.64555900
H	0.90964900	1.56447600	-2.57191800
H	0.57109400	2.58869200	-1.10831900
C	-2.96030600	0.12698200	-0.57944900
C	-3.88723000	-0.45117000	0.37671500
C	-3.37754600	1.27629200	-1.25875300
C	-3.54994800	-1.62427700	1.09428400
C	-5.15337600	0.15773800	0.61036800
C	-4.63103900	1.86349600	-1.00605800
H	-2.73686400	1.70618600	-2.02110000
C	-4.41951800	-2.18041700	2.00278600
H	-2.57888300	-2.06899100	0.90283700
C	-6.02973300	-0.43988900	1.55619800
C	-5.51387400	1.33557500	-0.09555000
H	-4.90064500	2.75988900	-1.56037000
C	-5.67709700	-1.57852800	2.23657200
H	-4.14263400	-3.08064900	2.54424000
H	-6.99577100	0.02910000	1.73134800
H	-6.48062200	1.79562300	0.09082700
H	-6.36315500	-2.01838600	2.95577400

IM-B2-R'

S	0.77908500	-0.91064500	-1.31432500
O	0.84755400	-1.27450900	-2.78931100
N	1.95483400	0.14009900	-0.82309300
C	-4.43798100	3.52202100	-0.56884500
C	-4.90129300	2.29631100	-1.04214000
C	-4.03871500	1.20853600	-1.12967800
C	-2.69447400	1.32800300	-0.75658500
C	-1.76957000	0.16522600	-0.87994200
C	-0.48494100	0.36017900	-1.21524500
C	-3.10612700	3.64894500	-0.18014200
C	-2.24399900	2.56098800	-0.26846500
C	-2.34452600	-1.19144400	-0.64324800
C	-3.12572700	-1.40038800	0.60674200
C	-2.76820100	-0.72562100	1.78024500
C	-3.47293200	-0.93152500	2.96155900
C	-4.55541400	-1.80795100	2.98882200
C	-4.92843700	-2.47566600	1.82431100
C	-4.22300300	-2.26903000	0.64322800
C	-2.15096800	-2.17791200	-1.52867800
H	-5.11234900	4.37030500	-0.49627700
H	-5.93884700	2.18622500	-1.34451500
H	-4.40270500	0.25478000	-1.50296900
H	-0.09836400	1.34851500	-1.45677700
H	-2.73787600	4.59612400	0.20355600
H	-1.21242300	2.65487800	0.05972500
H	-1.91995400	-0.04608400	1.76386200
H	-3.17508000	-0.40458900	3.86337400
H	-5.10866700	-1.96474600	3.91026900
H	-5.78005200	-3.15011600	1.83214100
H	-4.53473300	-2.77005700	-0.26961800
H	-1.57841200	-2.01127600	-2.43680800
H	-2.51644300	-3.18448600	-1.34059000
C	3.15785000	-0.42051800	-0.53024000
C	4.08191300	0.38460900	0.25917600
C	3.61431700	-1.68769700	-0.93048500
C	3.71718400	1.68458800	0.68051800
C	5.35891700	-0.12563700	0.62924600
C	4.87467900	-2.17320500	-0.54160800
H	2.99857900	-2.28249400	-1.59833400
C	4.57028800	2.46185100	1.42971600
H	2.73660300	2.04650800	0.38881700
C	6.21854900	0.69991600	1.40461600
C	5.74444400	-1.43097600	0.22350700

H	5.16999900	-3.16639800	-0.87516000
C	5.83869500	1.96039300	1.79649100
H	4.27072800	3.45857500	1.74178000
H	7.19457300	0.30823800	1.68524400
H	6.71920400	-1.81541200	0.51130400
H	6.51344200	2.57324200	2.38906200

IM-B3-R'

S	-0.89455500	-2.45776000	-0.63388700
O	-0.84953000	-3.96873000	-0.68058200
N	-1.65746100	-1.93871100	0.70342200
C	5.79906400	-0.84700500	-0.38432800
C	4.97103200	-1.26374800	-1.42419700
C	3.59290100	-1.32062100	-1.24260900
C	3.01405800	-0.97109900	-0.01635700
C	1.53667700	-1.05195700	0.20434200
C	0.85537900	-2.07167000	-0.35222400
C	5.23486300	-0.48205200	0.83592000
C	3.85653000	-0.53682200	1.01482700
C	0.95502200	0.00632900	1.07887200
C	1.25385000	1.42227400	0.72714200
C	1.45289200	1.80004000	-0.60715300
C	1.71063500	3.12429400	-0.94712400
C	1.78704900	4.10021900	0.04335300
C	1.60922300	3.73627400	1.37625800
C	1.35374200	2.41161700	1.71386400
C	0.21496000	-0.31536300	2.14763400
H	6.87476200	-0.79906900	-0.52688700
H	5.39805800	-1.53609700	-2.38542700
H	2.95004700	-1.62318200	-2.06487900
H	1.42679600	-2.81108300	-0.91279900
H	5.87046100	-0.15226900	1.65291600
H	3.41970100	-0.25091500	1.96821200
H	1.39533200	1.04463800	-1.38794500
H	1.85298100	3.39264600	-1.99034300
H	1.99242900	5.13384000	-0.21965800
H	1.68299700	4.48586100	2.15934500
H	1.24705400	2.13211200	2.75844400
H	0.02694400	-1.34979300	2.40494400
H	-0.26545000	0.44980300	2.75305200
C	-2.78033700	-1.16006300	0.57284300
C	-2.89127200	0.02775700	-0.25739900
C	-3.88705100	-1.46835000	1.37348500
C	-1.78673300	0.54286500	-0.98222600
C	-4.12806600	0.74422700	-0.32066200
C	-5.07988800	-0.73324100	1.32670600
H	-3.80107100	-2.33968500	2.01564600
C	-1.89854800	1.65375800	-1.78294500
H	-0.82231200	0.06056100	-0.87240900
C	-4.21942800	1.87854400	-1.17581800
C	-5.22544800	0.34205800	0.47831800
H	-5.91225700	-1.04031500	1.95668800
C	-3.14059600	2.31879700	-1.89789300
H	-1.02755300	2.03498600	-2.30836100
H	-5.17187300	2.40173200	-1.23450100
H	-6.15798200	0.89734000	0.42072900
H	-3.23076300	3.19149500	-2.53963000

TS2-R'

S	0.79583100	-2.73765900	0.07650000
O	0.77508800	-4.13364900	-0.49889100
N	1.53023700	-1.70863000	-0.95564600
C	-5.54064200	-0.26450600	1.06530400
C	-4.62080000	-0.86657100	1.92079300
C	-3.32667400	-1.13171700	1.48281300
C	-2.92802100	-0.80828100	0.17964500
C	-1.55213100	-1.10705500	-0.31212900
C	-0.93741100	-2.25372500	0.07971700
C	-5.15338100	0.07161100	-0.23096800
C	-3.86043800	-0.19517400	-0.66692000
C	-0.90464400	-0.21065700	-1.27114100
C	-0.98164700	1.25888000	-1.13027700
C	-1.25427300	1.87115400	0.10434400
C	-1.31613900	3.25487200	0.22769300
C	-1.10377900	4.07329300	-0.87927700
C	-0.83787700	3.48492300	-2.11434900
C	-0.78915900	2.10142400	-2.23829000
C	-0.05646700	-0.80463400	-2.16497400
H	-6.54969600	-0.05247400	1.40737100
H	-4.90757900	-1.12118800	2.93750000
H	-2.60141600	-1.57513800	2.16038700
H	-1.48853300	-2.98232600	0.67233000
H	-5.86248200	0.54301700	-0.90586100
H	-3.55740000	0.06533300	-1.67727900
H	-1.40956900	1.24832000	0.98107700
H	-1.52000100	3.69611400	1.19977900
H	-1.14824600	5.15424800	-0.78227900
H	-0.67947000	4.10704200	-2.99159700

H	-0.61626300	1.65705700	-3.21484200
H	-0.20105100	-1.84369500	-2.44101100
H	0.58441700	-0.21673500	-2.81638700
C	2.58694500	-0.91546200	-0.54190500
C	2.56223300	-0.01820000	0.59361000
C	3.72698100	-0.89762700	-1.34566300
C	1.40797100	0.14487300	1.40056400
C	3.71677800	0.76702500	0.90240900
C	4.84356100	-0.10010100	-1.04662900
H	3.73422500	-1.55622600	-2.20897000
C	1.39651800	1.00058700	2.47546200
H	0.50441500	-0.39341300	1.13750300
C	3.68086600	1.62791900	2.03455300
C	4.85840600	0.70833400	0.06639800
H	5.71267800	-0.14036100	-1.69917700
C	2.55470700	1.74061200	2.80749800
H	0.49147800	1.11748600	3.06535100
H	4.57011100	2.21007000	2.26700500
H	5.72634400	1.31488800	0.31199900
H	2.54347500	2.40934500	3.66406000

IM-B4-R'

S	0.80532600	-2.79128700	-0.18281900
O	0.95358600	-4.02169900	-1.03730400
N	1.33140900	-1.41719700	-1.01728700
C	-5.38303800	-0.41166300	1.22334400
C	-4.40838800	-1.01842600	2.01096900
C	-3.12999100	-1.23405700	1.50096900
C	-2.79795600	-0.84592000	0.19783700
C	-1.43545500	-1.10117200	-0.36458500
C	-0.89343700	-2.35847400	-0.10242700
C	-5.06677500	-0.02352500	-0.07841400
C	-3.79010300	-0.23973200	-0.58386700
C	-0.78563600	-0.15870200	-1.18559400
C	-0.96955300	1.28269800	-1.13602400
C	-1.47241800	1.95485100	0.00519900
C	-1.65138700	3.33099900	0.02879700
C	-1.31046100	4.11962900	-1.07101800
C	-0.78746300	3.48823300	-2.19880000
C	-0.62882700	2.10861400	-2.23421900
C	0.35061900	-0.76501700	-1.93875800
H	-6.38069400	-0.24254800	1.61916700
H	-4.64161800	-1.32097600	3.02851000
H	-2.36375900	-1.69661900	2.11792600
H	-1.48640600	-3.14384100	0.35546400
H	-5.82064800	0.44679300	-0.70418500
H	-3.54062800	0.05918800	-1.59786000
H	-1.70588400	1.37773300	0.89508600
H	-2.04239000	3.79709500	0.93055600
H	-1.44000900	5.19763800	-1.04562300
H	-0.51244000	4.07652500	-3.07156700
H	-0.25351400	1.64891700	-3.14501200
H	0.00909500	-1.58012800	-2.59463500
H	0.91406600	-0.04610100	-2.53870200
C	2.46265300	-0.69781000	-0.55386300
C	2.44699100	0.03330300	0.67843100
C	3.58770700	-0.66170000	-1.34999800
C	1.30581200	0.06904400	1.52312900
C	3.60932200	0.76612700	1.05984500
C	4.73062500	0.07791400	-0.97371600
H	3.57521600	-1.22772700	-2.27667500
C	1.32525500	0.78331500	2.69493000
H	0.41497800	-0.47259600	1.22113400
C	3.59645600	1.49012500	2.28203900
C	4.74602500	0.77004100	0.21005400
H	5.60142700	0.08302700	-1.62291600
C	2.48322500	1.49953000	3.08208000
H	0.44116600	0.80665600	3.32544800
H	4.48795200	2.04438500	2.56618400
H	5.62353100	1.33659000	0.51230300
H	2.48397800	2.06122400	4.01189900

TS1-R-a

P	-2.26767500	-1.99468000	-0.15037000
O	-0.76425400	2.80299900	1.40593500
N	-1.91609400	0.33210800	-2.54219700
H	-0.91201000	0.54811000	-2.50445900
H	-0.67409100	-0.38414200	4.98713800
O	-4.05564800	1.01441000	-2.20923700
O	-2.72357800	3.74203300	0.71789200
N	-1.39653000	2.54936900	-0.71427600
H	-0.44404200	2.17124100	-0.82019700
C	-2.23981100	-0.30693900	0.58982200
H	-1.26958800	0.10923000	0.29016500
H	-2.99949800	0.26244200	0.04380000
C	-2.09441300	-1.47962700	-4.29069700
C	-2.02740400	3.31244800	3.46598800
H	-2.85759700	3.89659800	3.07000000

H	-1.85136500	3.60063900	4.50905500
H	-2.29977100	2.25260700	3.44806900
C	-0.74254000	3.53313900	2.66956200
C	-1.71236600	3.09866900	0.49328800
C	-2.34427000	2.64755400	-1.81001300
H	-3.23131100	3.12556500	-1.38865300
C	-2.84744800	1.26001200	-2.22556400
C	-2.27776600	-1.05069800	-2.80808700
H	-3.35397500	-1.11321800	-2.62660100
C	-1.51774400	-1.95358500	-1.81598200
H	-0.47582000	-1.61478900	-1.72663600
H	-1.51203300	-2.99844100	-2.14441100
C	-2.44615400	-0.22935900	2.08683800
C	-1.33512900	-0.33775000	2.95071400
C	-1.53722800	-0.29842900	4.32944500
C	-1.33869700	-3.15771400	0.86491000
C	-1.80979500	-3.46955500	2.14824100
H	-2.74454600	-3.04978400	2.51143300
C	-1.06927900	-4.31835700	2.96305300
H	-1.42861200	-4.55260000	3.95999900
C	0.13082100	-4.85833700	2.50194900
H	0.71494100	-5.51111900	3.14382000
C	0.59246200	-4.56010100	1.22303500
H	1.53462500	-4.96997800	0.87044200
C	-0.13914300	-3.70904500	0.39924200
H	0.24805600	-3.46218800	-0.58495800
C	-3.95111400	-2.63784300	-0.33802600
C	-4.96513300	-1.81026800	-0.83999700
H	-4.78171300	-0.75830900	-1.05047800
C	-6.22587200	-2.34186300	-1.09441000
H	-7.00646100	-1.69548800	-1.48242600
C	-6.48109000	-3.68968900	-0.85274600
H	-7.46716200	-4.09909200	-1.05008500
C	-5.47279700	-4.51429500	-0.36055100
H	-5.66802400	-5.56532800	-0.17341800
C	-4.20746700	-3.99483200	-0.10544800
H	-3.42712500	-4.64728300	0.27394000
C	-1.86563300	3.53519200	-2.98821200
C	-2.97082700	3.57631600	-4.04833100
H	-3.91398400	3.94076500	-3.62553900
H	-3.15766200	2.58586100	-4.47920800
H	-2.68266400	4.24626800	-4.86637200
C	-1.65213700	4.94835300	-2.43332300
H	-1.29398400	5.61799800	-3.22371000
H	-0.91958400	4.95377500	-1.62023000
H	-2.58848000	5.35636400	-2.03622300
C	-0.56971600	3.01479000	-3.62014700
H	-0.73738900	2.09331800	-4.18891000
H	0.20620900	2.79489700	-2.87810600
H	-0.16895400	3.76137200	-4.31538400
C	0.43584200	2.91564400	3.41171600
H	0.20758300	1.88433800	3.69800300
H	0.63792200	3.48857900	4.32271100
H	1.33349400	2.92152900	2.78742000
C	-0.47999800	5.00968200	2.39460400
H	0.49944300	5.14356000	1.92510800
H	-0.48781800	5.56189300	3.34061200
H	-1.25246300	5.42968000	1.74513700
C	-2.79323400	-0.43547000	-5.16892100
H	-2.26134900	0.52031000	-5.14042900
H	-3.82417100	-0.26039200	-4.83881500
H	-2.81786300	-0.77464000	-6.21027200
C	-0.62253300	-1.57829600	-4.69500200
H	-0.55005800	-1.85796700	-5.75261800
H	-0.08563100	-2.33842200	-4.11471900
H	-0.10013100	-0.62809300	-4.55201200
C	-2.78879400	-2.83199200	-4.49904700
H	-3.83728600	-2.79477900	-4.17798100
H	-2.29613700	-3.64786300	-3.95895900
H	-2.77299700	-3.09864800	-5.56119400
C	-3.72623900	-0.00496900	2.62860400
C	-3.87936400	0.02855800	4.01771400
H	-4.87183700	0.20365700	4.42798700
C	-2.80439000	-0.13035000	4.88651200
C	0.07404000	-0.47172700	2.43353700
H	0.31297600	0.30537600	1.69437800
H	0.78951700	-0.36947000	3.25466300
H	0.24068000	-1.45561100	1.98020800
C	-4.95034400	0.23478900	1.77955600
H	-4.74485400	0.88175600	0.92011200
H	-5.37568000	-0.70020700	1.40221600
H	-5.72275700	0.72725100	2.37569100
C	-2.98796100	-0.04522800	6.37865800
H	-4.01522200	-0.28309500	6.66834100
H	-2.31444200	-0.72954000	6.90323200
H	-2.76900700	0.96799800	6.73557200
S	1.88617200	0.30769600	-1.48488300
O	0.86559500	0.20512800	-2.59888400

N	1.43818900	1.60471100	-0.65509000
C	4.06908500	-4.78760800	2.36046600
C	4.38509700	-5.00963700	1.02001600
C	4.11739300	-4.03245700	0.06563300
C	3.52138800	-2.82011800	0.43391800
C	3.22031100	-1.78910000	-0.59586900
C	1.98919100	-1.12812300	-0.53480500
C	3.48354100	-3.58117700	2.73820300
C	3.20616400	-2.60883600	1.78150400
C	4.15264600	-1.36276200	-1.56145500
C	5.61856500	-1.44846500	-1.41358300
C	6.26682100	-1.48045300	-0.16612600
C	7.65243300	-1.53972600	-0.07735100
C	8.43733900	-1.54861600	-1.22961600
C	7.81552400	-1.49976600	-2.47432600
C	6.42779500	-1.45826800	-2.56285900
C	3.58569400	-0.46303600	-2.51963400
H	4.28414700	-5.54804100	3.10561100
H	4.84679900	-5.94510200	0.71726000
H	4.37141700	-4.19880200	-0.97763700
H	1.30562100	-1.24145000	0.29676700
H	3.24033900	-3.39500800	3.78058400
H	2.75354400	-1.66693000	2.08248600
H	5.67817200	-1.43801400	0.74496800
H	8.12406600	-1.56100100	0.90133400
H	9.52005600	-1.58905800	-1.15648000
H	8.41200800	-1.50534100	-3.38240200
H	5.95050700	-1.44922000	-3.53986500
H	2.83270800	-0.84250700	-3.21391400
H	4.22880300	0.29442500	-2.96906700
C	2.32707000	2.18014900	0.26320200
C	2.35952000	3.62339500	0.31177900
C	3.14529000	1.48431000	1.14044700
C	1.59408500	4.41115300	-0.58414700
C	3.19470900	4.28547600	1.25608900
C	3.98428800	2.15297000	2.05927200
H	3.13800000	0.40016300	1.12938400
C	1.63922700	5.78348100	-0.54464500
H	0.97880800	3.90064900	-1.31467200
C	3.20924300	5.70629800	1.28430400
C	4.00728800	3.52091900	2.13580300
H	4.61014100	1.55914000	2.72041300
C	2.45269100	6.44142400	0.40638600
H	1.04890300	6.36618500	-1.24654000
H	3.84434400	6.20124400	2.01534800
H	4.64350100	4.03497100	2.85126500
H	2.47992400	7.52682700	0.43735700

TS1-R-b

P	3.30417800	0.62422200	-1.39442000
O	0.14880300	-0.28980200	2.84377200
N	1.96587000	-2.22565100	-0.87896000
H	1.07907900	-1.75967800	-0.67291700
H	1.39572800	4.91091300	1.56053300
O	3.66355200	-3.54040200	-0.14332500
O	2.18932300	-1.13351000	3.41111900
N	0.97483700	-1.99708800	1.67659700
C	2.60713200	0.57218100	0.31466100
H	1.55034300	0.31627900	0.17355800
H	3.07905300	-0.28238700	0.80888400
C	1.82276200	-2.88970600	-3.27816600
C	1.09760700	1.44693300	4.31521100
H	2.05552100	0.95630600	4.49011000
H	0.85130200	2.07051500	5.18198800
H	1.18496900	2.09859700	3.44031600
C	-0.02077600	0.43161400	4.10183500
C	1.18922300	-1.14152700	2.70703500
C	1.90965400	-3.07838700	1.42949400
H	2.72714500	-2.94873500	2.14512600
C	2.58727600	-2.97953700	0.05742600
C	2.52387400	-2.00740500	-2.20244600
H	3.57169200	-2.32585400	-2.15537300
C	2.46036200	-0.51051000	-2.55156800
H	1.41782700	-0.17943000	-2.59849400
H	2.94905300	-0.32445900	-3.51407300
C	2.78600200	1.84369200	1.11369600
C	1.89855300	2.92166900	0.94456300
C	2.08804000	4.08236700	1.70154300
C	3.13071400	2.28728300	-2.08083300
C	3.82455200	3.34785400	-1.48132900
H	4.49906400	3.16469500	-0.64885200
C	3.64062300	4.64498100	-1.94580800
H	4.17059000	5.46408400	-1.47067900
C	2.77266100	4.88998200	-3.00883500
H	2.62779200	5.90479700	-3.36651100
C	2.09310500	3.83694100	-3.61518600
H	1.41796300	4.02614700	-4.44338500
C	2.26687000	2.53583000	-3.15228800

H	1.71488200	1.72902500	-3.62281100	H	2.03055300	-3.31411400	-5.39139800
C	5.06500000	0.19402300	-1.39369500	C	3.82812400	1.93762100	2.06131100
C	5.48234500	-0.94855300	-0.69844300	C	3.97583500	3.11291400	2.79425900
H	4.78321600	-1.54636800	-0.11894500	H	4.77732600	3.17621300	3.52784600
C	6.81281500	-1.34926000	-0.76381900	C	3.11885500	4.20261100	2.62619000
H	7.12641700	-2.23807300	-0.22596400	C	0.73662600	2.91433700	-0.01981700
C	7.72839000	-0.61586500	-1.51523200	H	-0.18265300	3.20547500	0.49965000
H	8.76687600	-0.92936600	-1.55984100	H	0.90400400	3.64962900	-0.81713600
C	7.31304000	0.51491600	-2.21355700	H	0.54594100	1.94421000	-0.48348100
H	8.02356200	1.08531500	-2.80331100	C	4.76152200	0.78508900	2.33784600
C	5.98261900	0.92049100	-2.16037600	H	4.20302400	-0.09726900	2.67345100
H	5.66609800	1.79901900	-2.71403500	H	5.34632800	0.50431800	1.45584000
C	1.27728200	-4.47739700	1.70250100	H	5.46716800	1.05262000	3.12858300
C	2.32237400	-5.58336600	1.51868400	C	3.28194600	5.44604800	3.46040200
H	3.21657200	-5.39302600	2.12268300	H	2.83418500	5.30846900	4.45146000
H	2.64082400	-5.67719200	0.47767300	H	4.33814200	5.68917100	3.61133100
H	1.89369100	-6.54103200	1.83542100	H	2.79386200	6.30617100	2.99379900
C	0.79547100	-4.49534700	3.15786700	H	0.06135400	-1.95209800	1.18789400
H	0.37891600	-5.47918100	3.40138200	S	-1.51551800	-0.39625700	-0.68389500
H	0.01200600	-3.75048400	3.33116400	O	-0.06042200	-0.24676900	-1.10634300
H	1.62051100	-4.29339500	3.85129300	N	-1.51863600	-1.77180500	0.16176400
C	0.09059500	-4.73338700	0.77039000	C	-5.79190600	3.94013800	2.14806800
H	0.38887500	-4.68930600	-0.28404300	C	-5.43440000	4.37434300	0.87328400
H	-0.71248400	-4.00527800	0.92016000	C	-4.62326600	3.58249100	0.06670900
H	-0.32234400	-5.73141500	0.95708700	C	-4.15413700	2.34656900	0.52500200
C	-1.35270200	1.14762000	3.91380700	C	-3.27230900	1.51503700	-0.33552600
H	-1.28391900	1.88794600	3.11006300	C	-2.17919300	0.87409900	0.25320300
H	-1.63122300	1.66868500	4.83505400	C	-5.33388000	2.70818400	2.61108800
H	-2.14023000	0.42703900	3.66725500	C	-4.51829000	1.91747100	1.80681900
C	-0.11777800	-0.56860700	5.25080900	C	-3.51700600	1.27031000	-1.70310900
H	-0.92802400	-1.28331400	5.06611400	C	-4.84571500	1.27647800	-2.34126400
H	-0.34083900	-0.03643700	6.18116300	C	-6.03682400	1.05727600	-1.62608400
H	0.81883400	-1.11515300	5.37874400	C	-7.26641700	1.02166700	-2.27194600
C	2.14359900	-4.35480700	-2.96233200	C	-7.34755500	1.18671500	-3.65458000
H	1.79443600	-4.63771300	-1.96506700	C	-6.17865700	1.39375800	-4.38220500
H	3.22240700	-4.54178600	-3.00330300	C	-4.94882000	1.44660700	-3.73252700
H	1.65299100	-5.01135300	-3.68944300	C	-2.42641000	0.58281100	-2.32424500
C	0.30323400	-2.68615700	-3.26451000	H	-6.42758300	4.55725100	2.77626000
H	-0.16042400	-3.33889700	-4.01295600	H	-5.78944400	5.33250000	0.50510600
H	0.02453000	-1.65349100	-3.50398800	H	-4.34349500	3.91571600	-0.92884000
H	-0.13082200	-2.93025200	-2.28773800	H	-1.98347700	0.85900100	1.31880400
C	2.37432700	-2.56385300	-4.67127800	H	-5.61839600	2.35747700	3.59894600
H	3.47169800	-2.57266700	-4.68070600	H	-4.18068600	0.94476200	2.15641000
H	2.03317300	-1.58846700	-5.03528400	H	-5.99112000	0.87291600	-0.55708900

H	-8.16701800	0.83818200	-1.69201100
H	-8.30947000	1.15077500	-4.15758700
H	-6.22358300	1.52731900	-5.45978900
H	-4.04474000	1.63795900	-4.30591400
H	-1.47050600	1.10624800	-2.41201500
H	-2.63166000	-0.06339400	-3.17920400
C	-2.71120100	-2.06007900	0.85395500
C	-3.98772700	-2.21512700	0.20586600
C	-2.64385600	-2.22947000	2.22610800
C	-4.13306500	-2.15294500	-1.20506000
C	-5.14894400	-2.45304800	0.99875200
C	-3.79221600	-2.51830900	2.99683800
H	-1.68091600	-2.08889200	2.71007400
C	-5.36717700	-2.25179200	-1.79774700
H	-3.24420800	-2.05719300	-1.81892800
C	-6.41286100	-2.54471200	0.35582900
C	-5.02616100	-2.60529000	2.40503100
H	-3.69022500	-2.64201700	4.07198200
C	-6.52497400	-2.43242100	-1.00622000
H	-5.45902900	-2.18564100	-2.87817100
H	-7.29445900	-2.70950500	0.97103000
H	-5.91945300	-2.79860600	2.99305700
H	-7.49875100	-2.49487900	-1.48329400

10. NMR Spectra of New Compounds

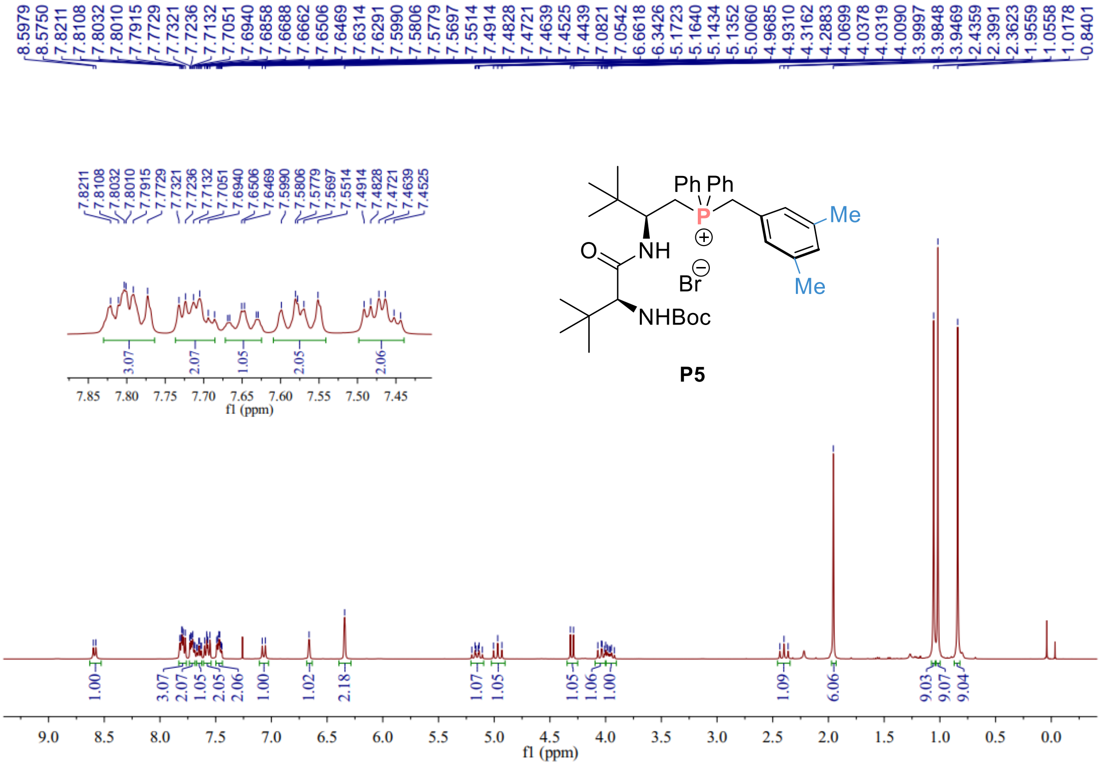

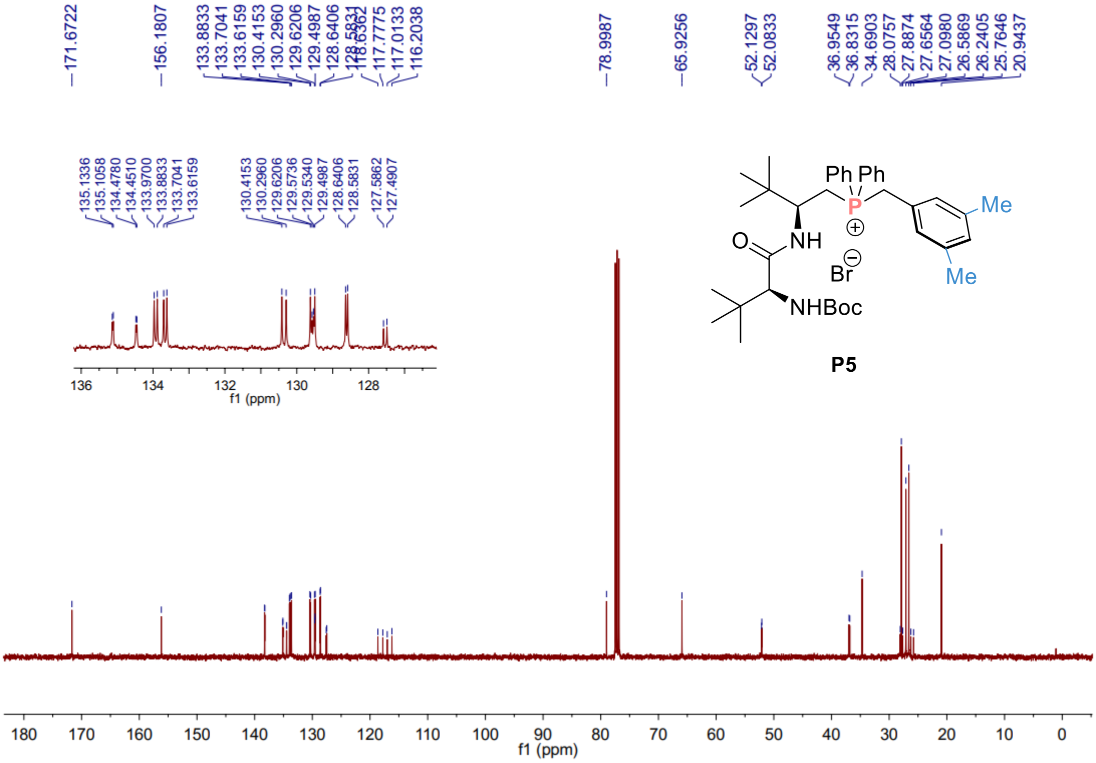

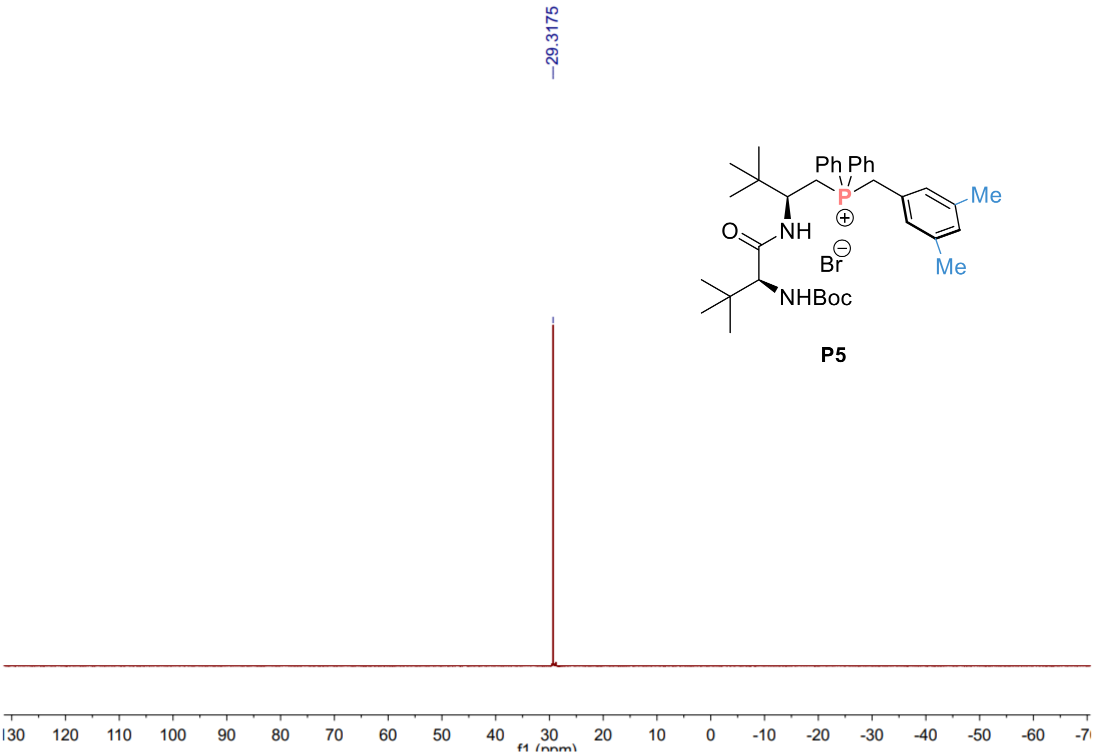

^{1}H-NMR and ^{13}C-NMR and ^{31}P-NMR of **P5**

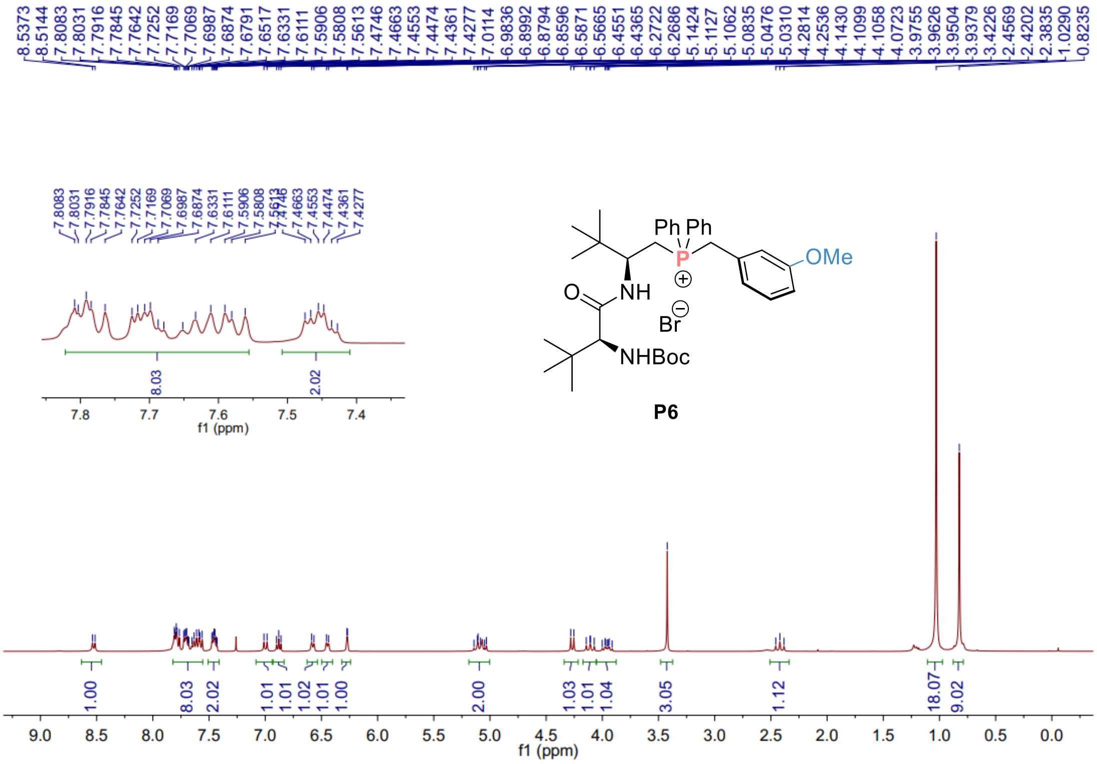

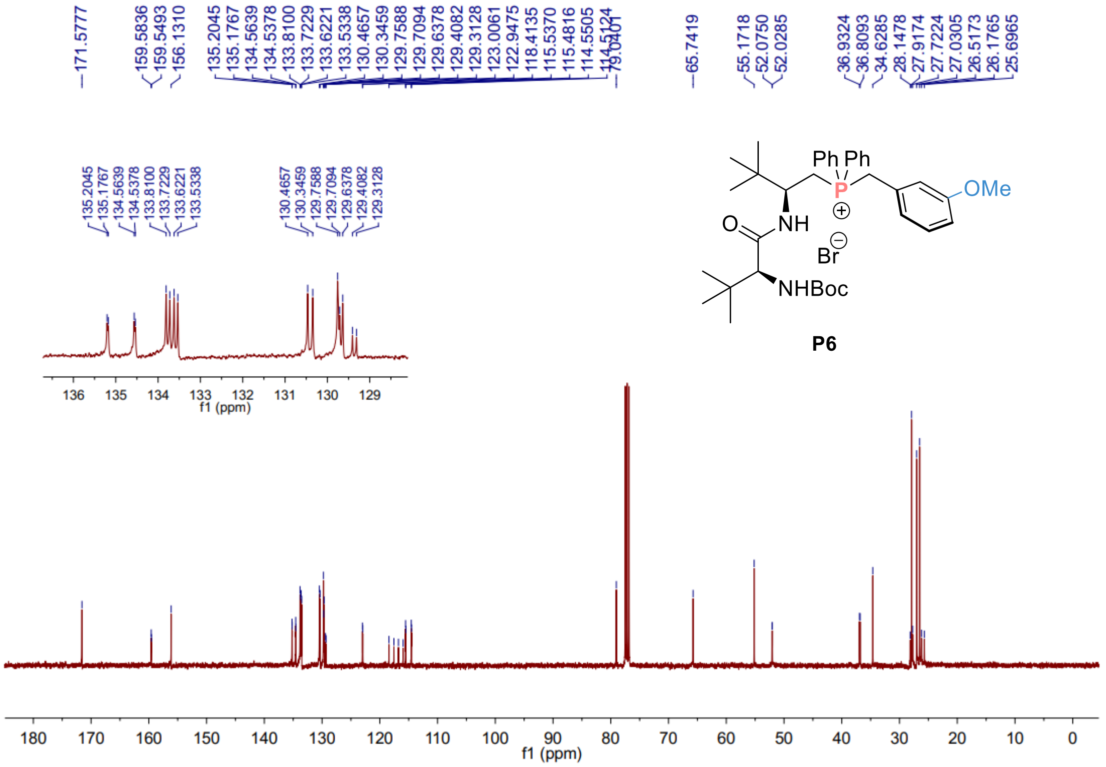

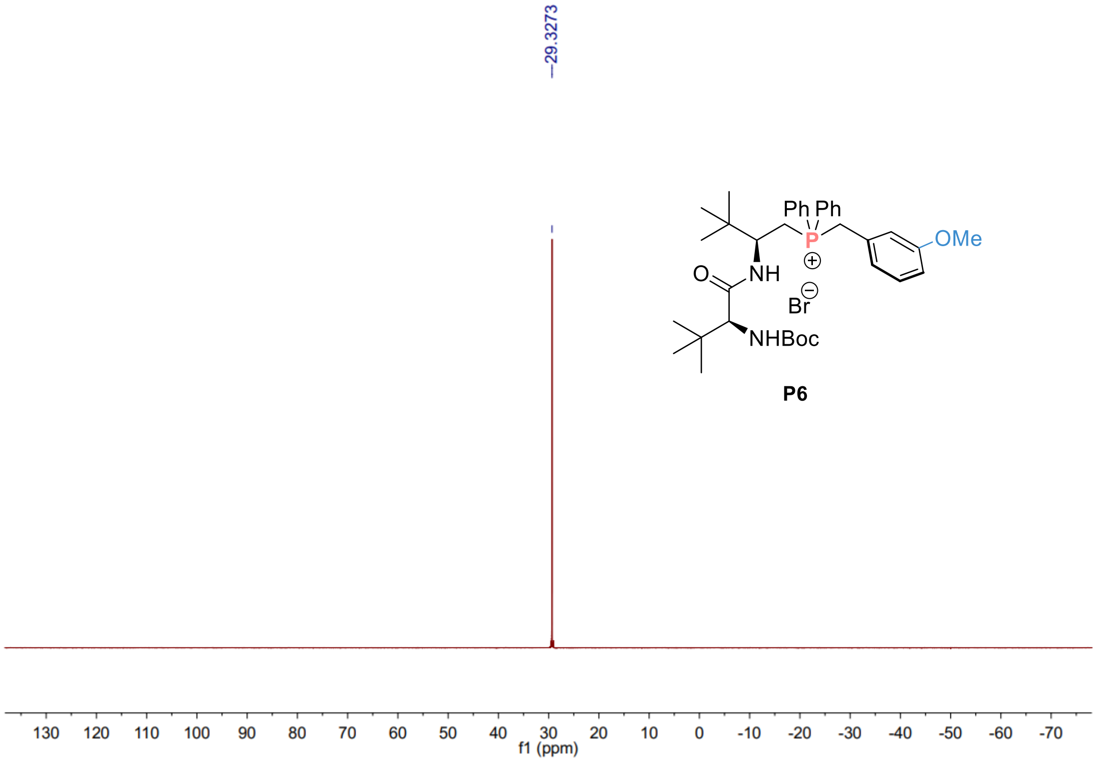

^{1}H-NMR and ^{13}C-NMR and ^{31}P-NMR of **P6**

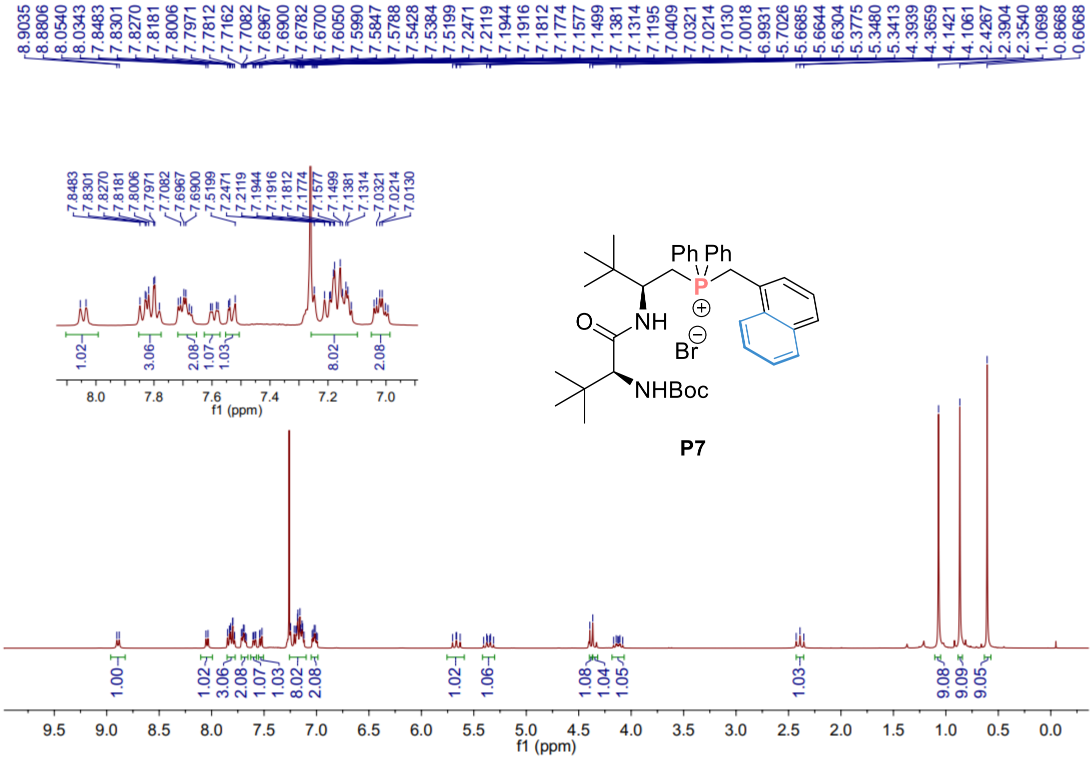

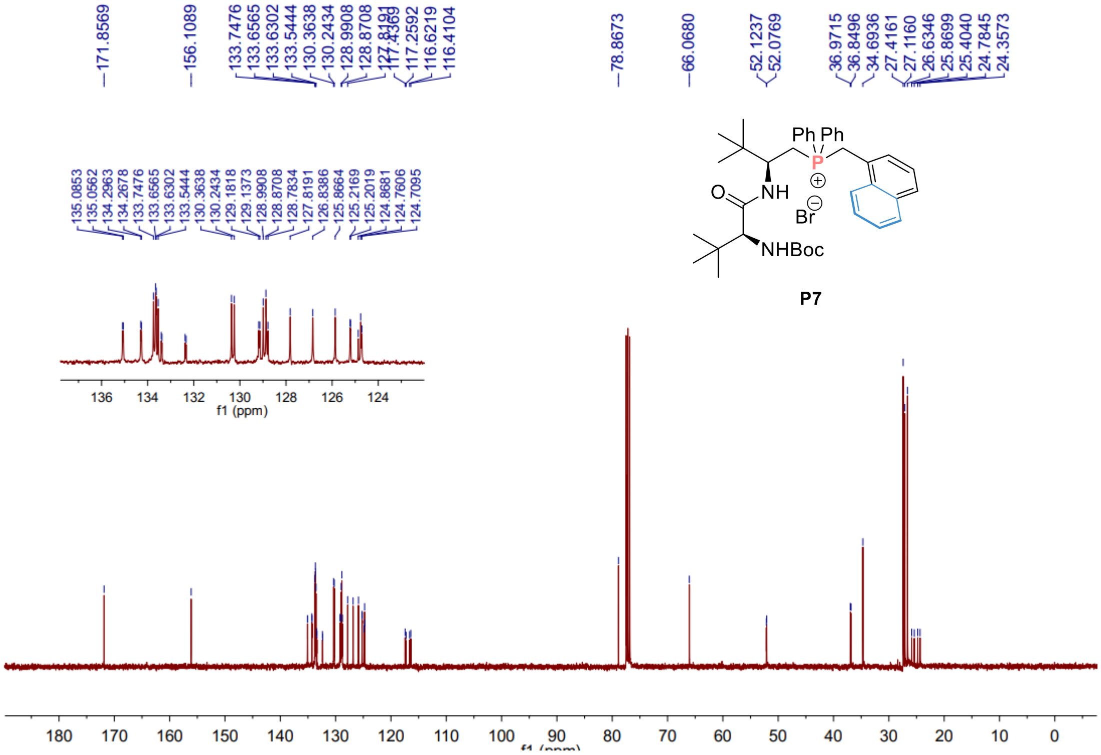

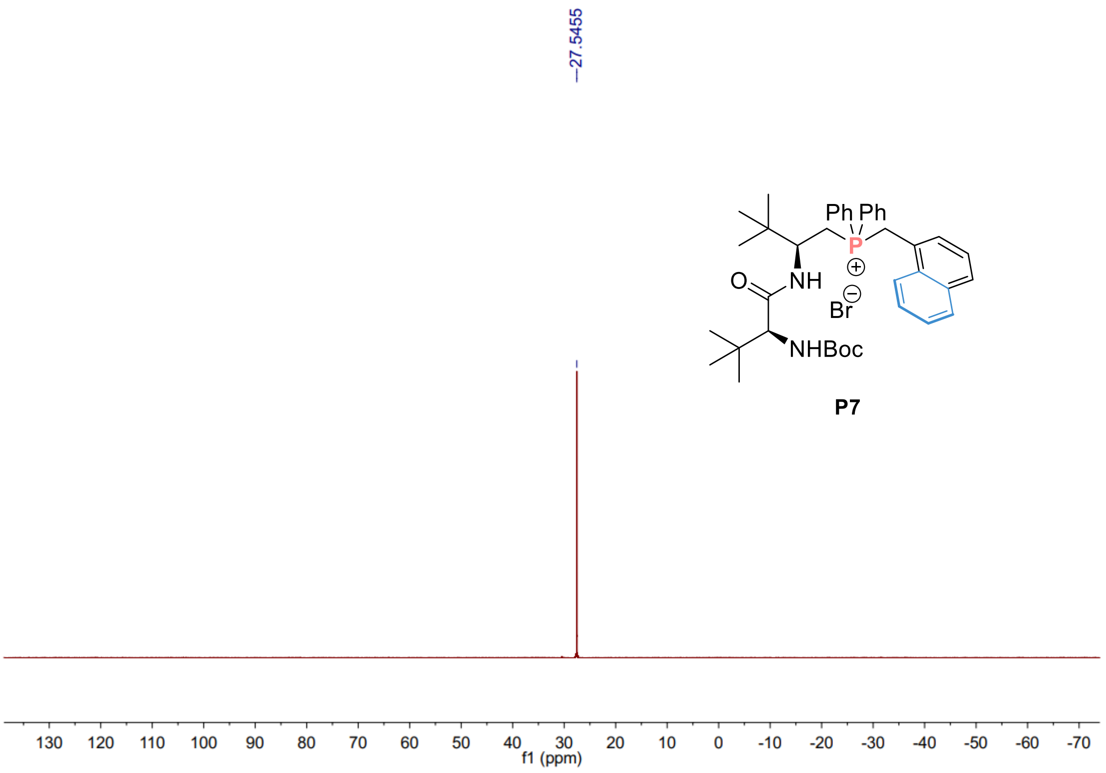

^{1}H-NMR and ^{13}C-NMR and ^{31}P-NMR of **P7**

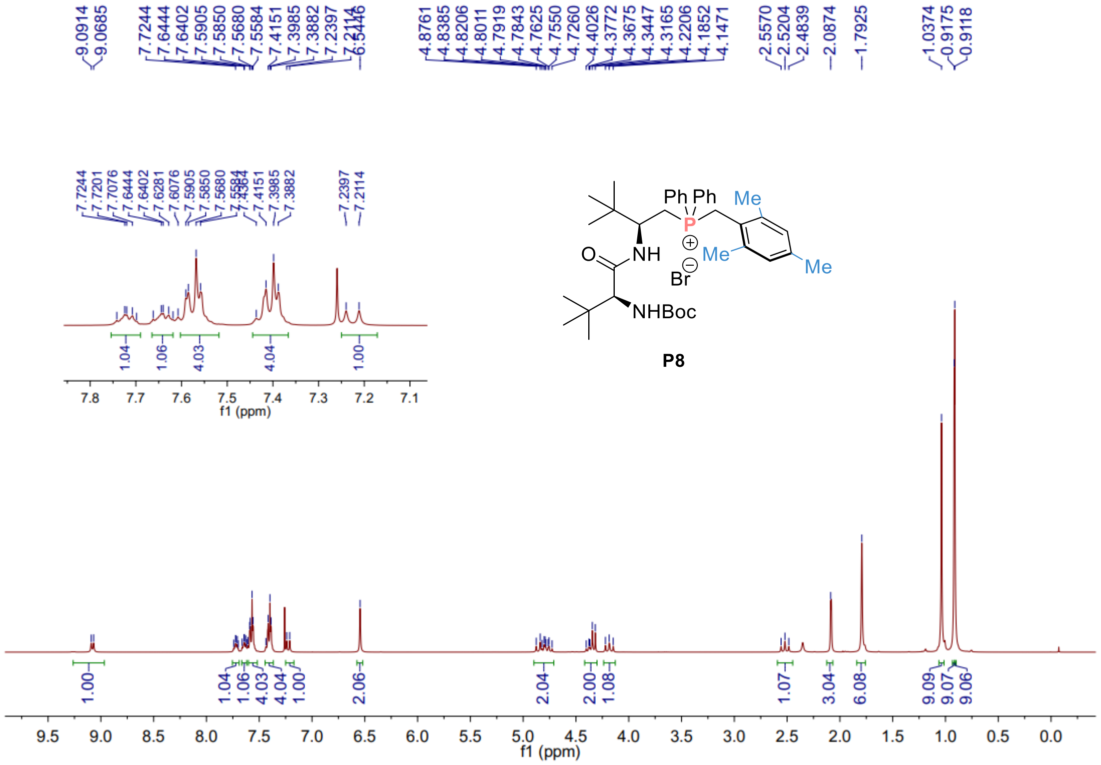

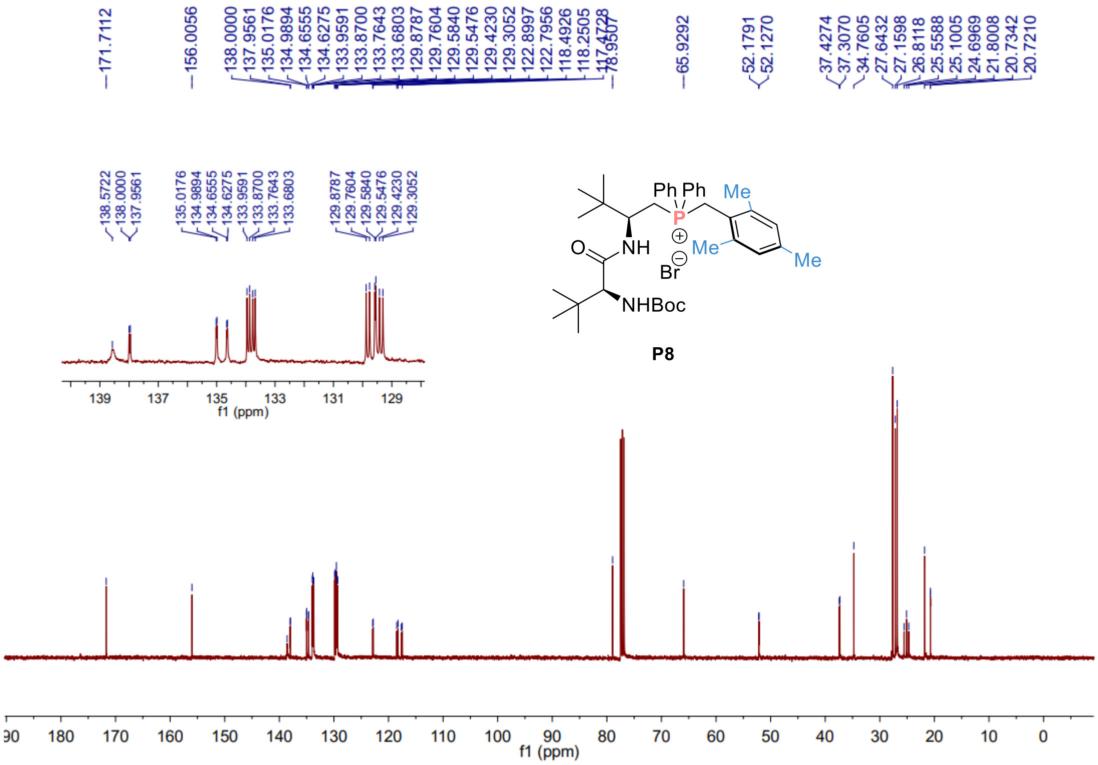

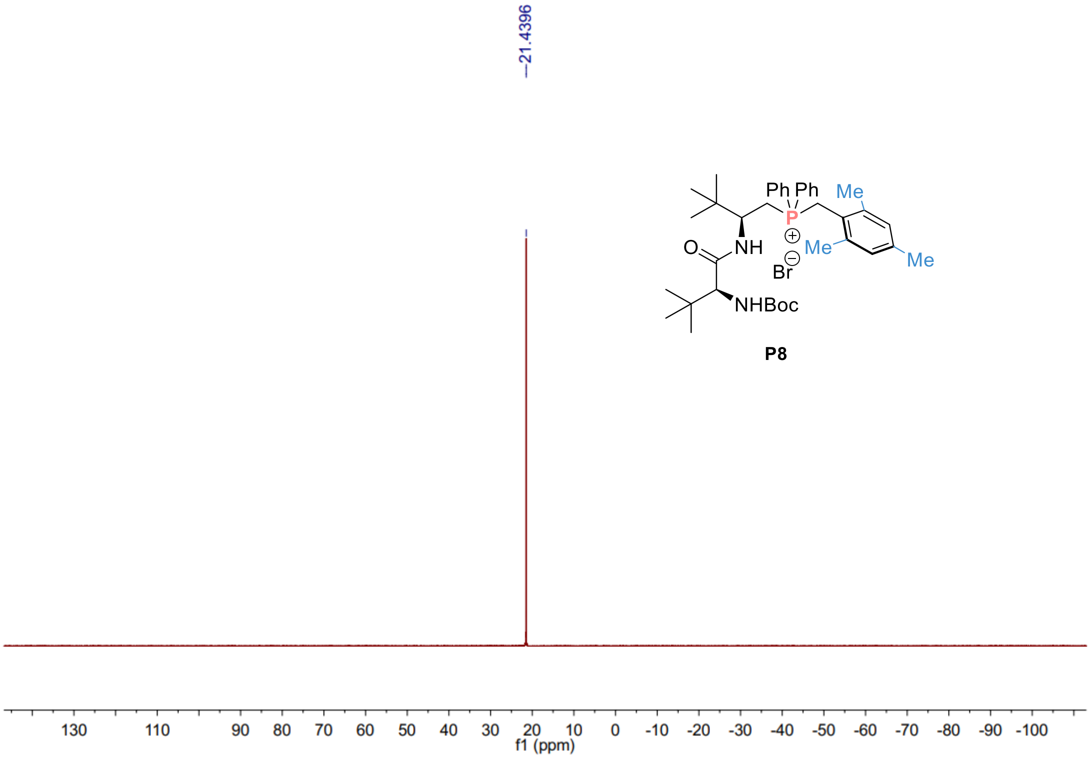

^{1}H-NMR and ^{13}C-NMR and ^{31}P-NMR of **P8**

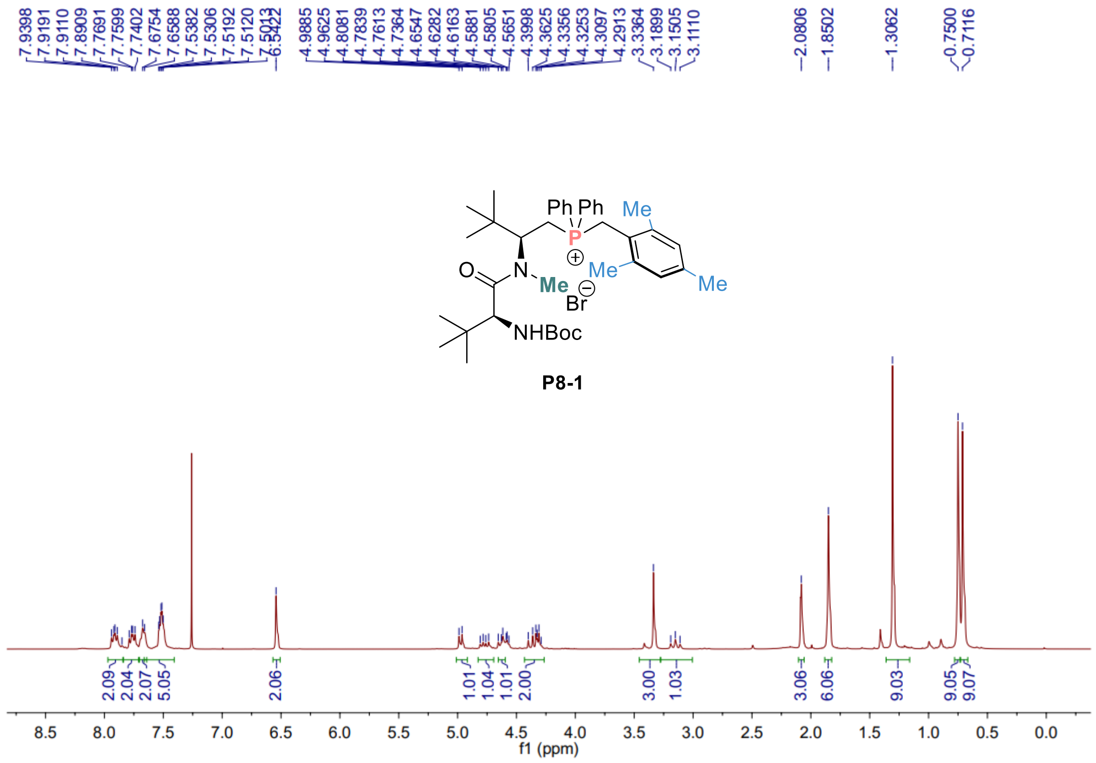

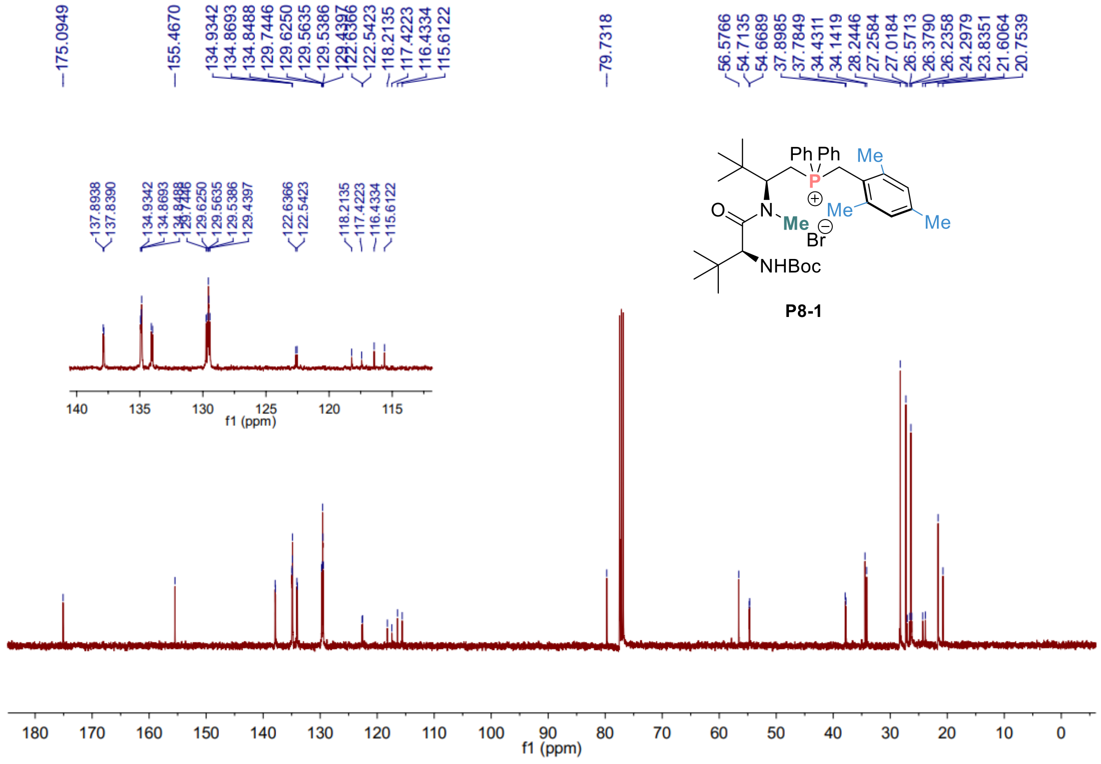

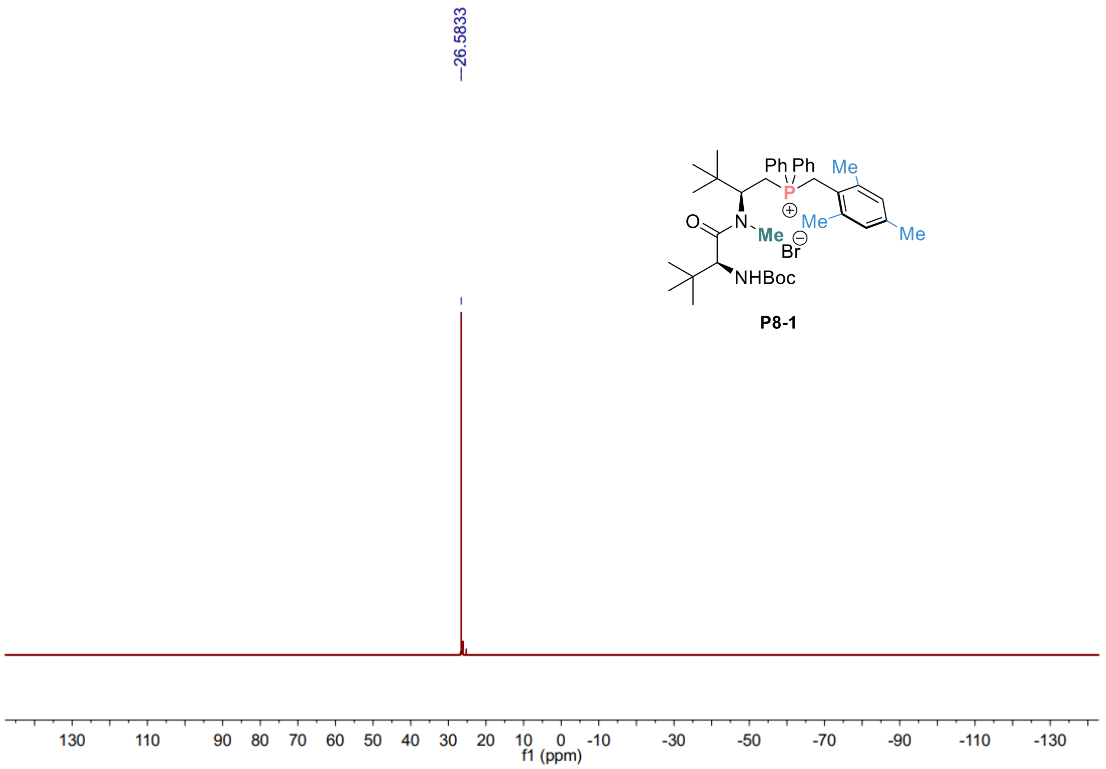

^{1}H-NMR and ^{13}C-NMR and ^{31}P-NMR of **P8-1**

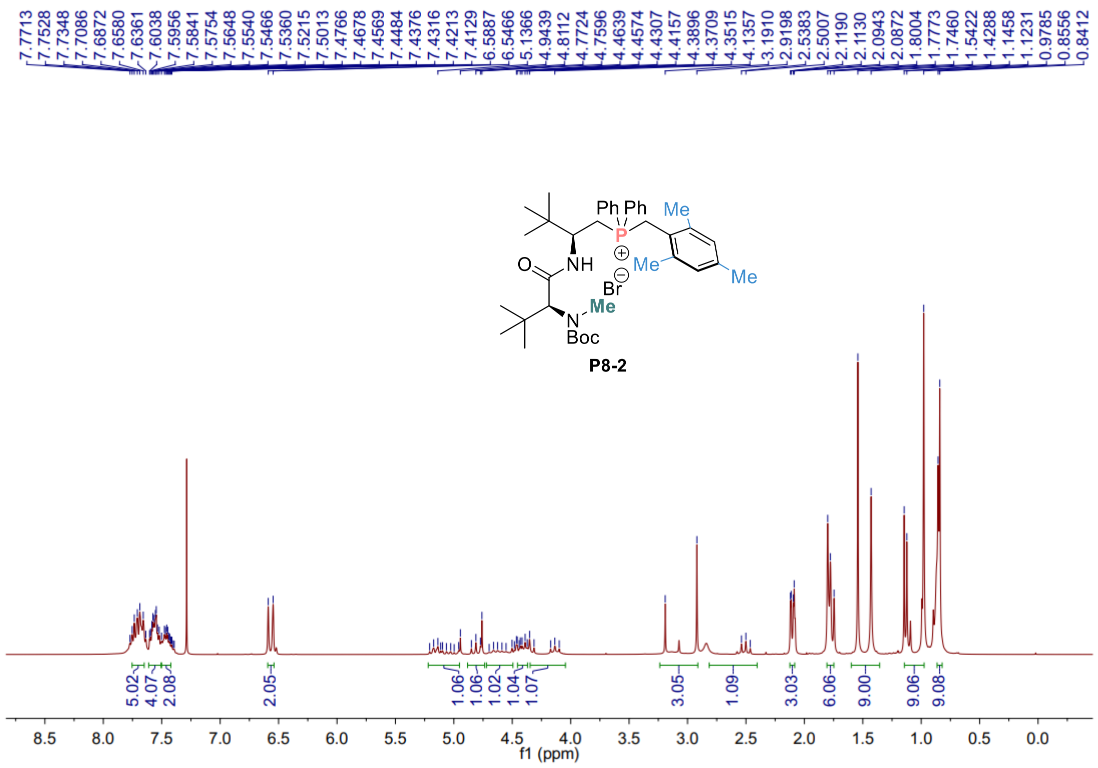

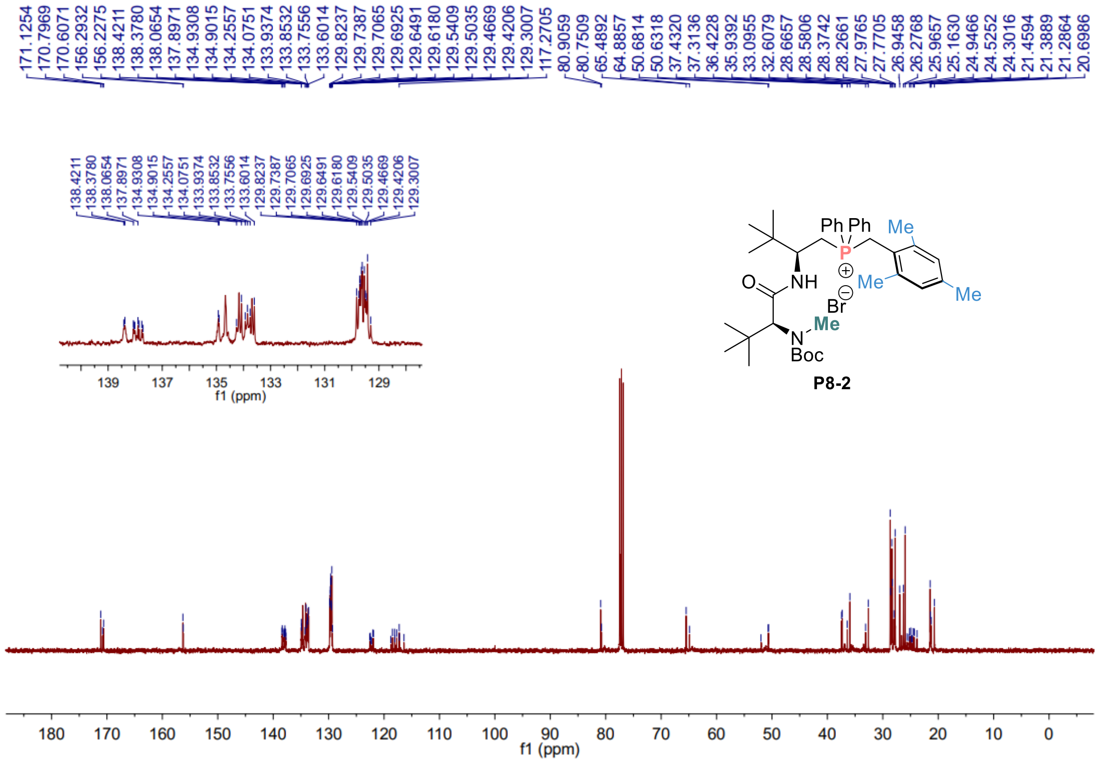

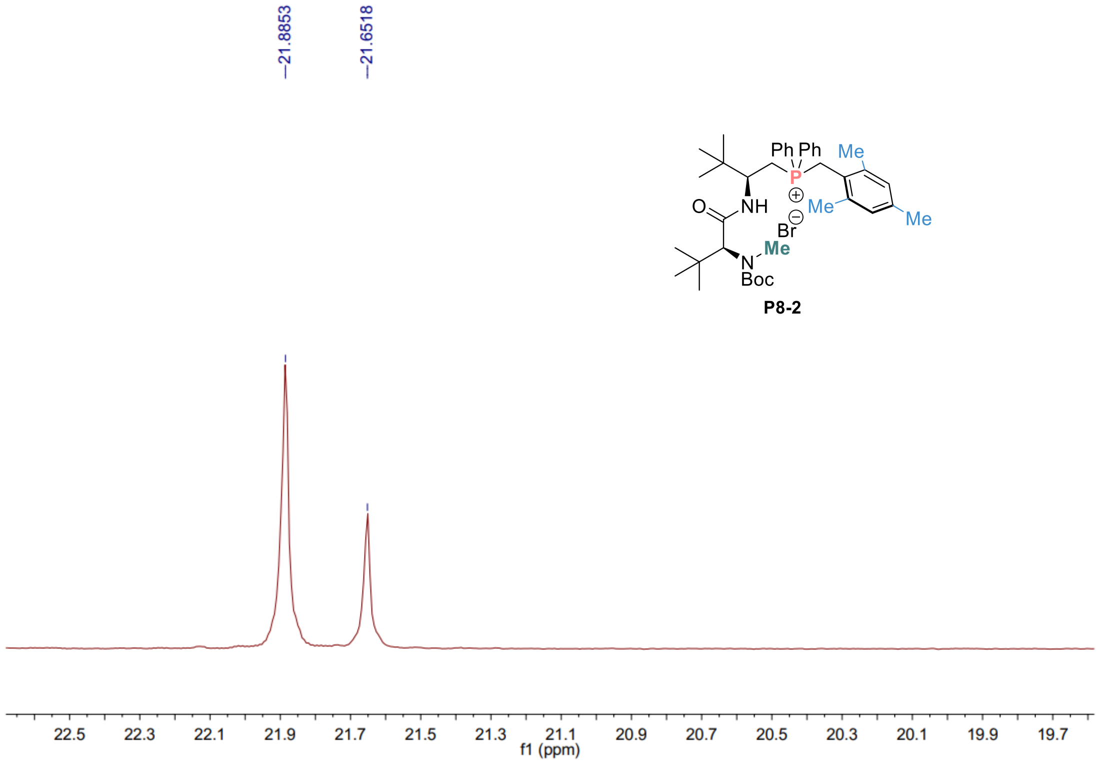

^{1}H-NMR and ^{13}C-NMR and ^{31}P-NMR of **P8-2**

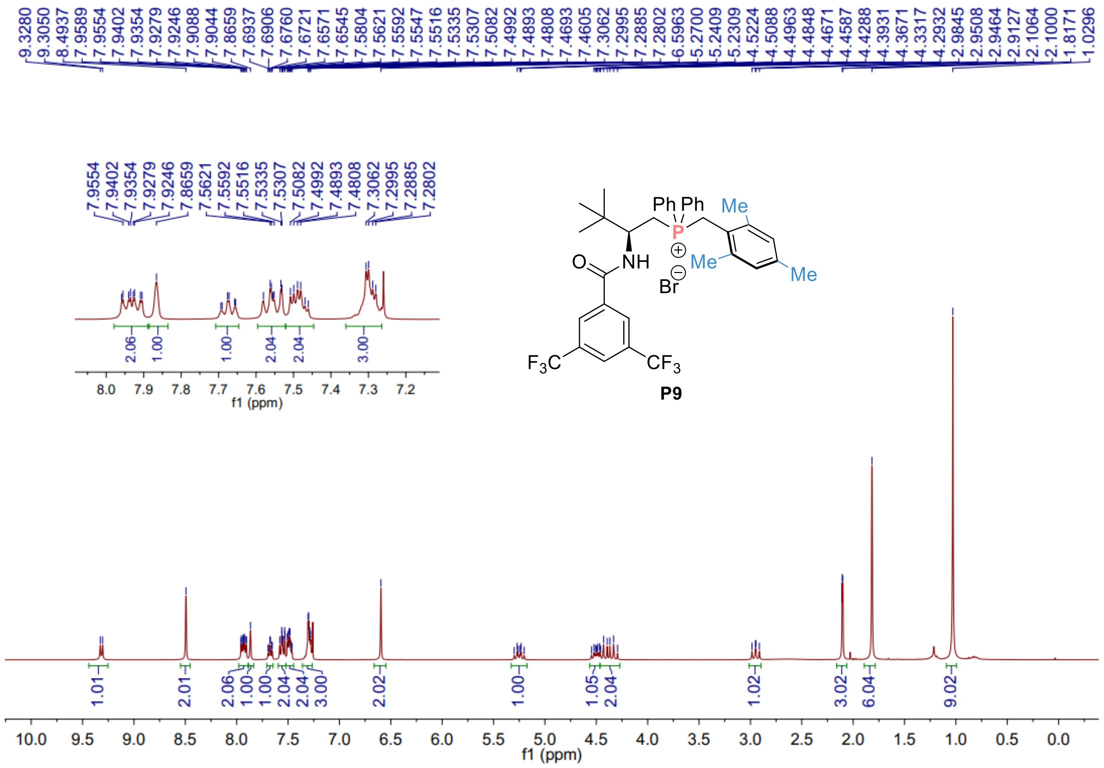
9.3280 9.3050 8.4937 7.9589 7.9554 7.9402 7.9354 7.9279 7.9246 7.9088 7.9044 7.8659 7.6937 7.6906 7.6760 7.6721 7.6571 7.6545 7.5804 7.5621 7.5592 7.5547 7.5516 7.5335 7.5307 7.5082 7.4992 7.4893 7.4808 7.4693 7.4605 7.3062 7.2995 7.2885 7.2802 6.5963 5.2700 5.2409 5.2309 4.5224 4.5088 4.4963 4.4848 4.4671 4.4587 4.4288 4.3931 4.3671 4.3317 4.2932 2.9845 2.9508 2.9464 2.9127 2.1064 2.1000 1.8171 1.0296
Ph Ph
Me
P
NH
Me
O
Br
Me
F3C
CF3
P9
1.01 2.01 2.06 1.00 1.00 2.04 2.04 3.00 2.02 1.00 1.05 2.04 1.02 3.02 6.04 9.02
f1 (ppm)

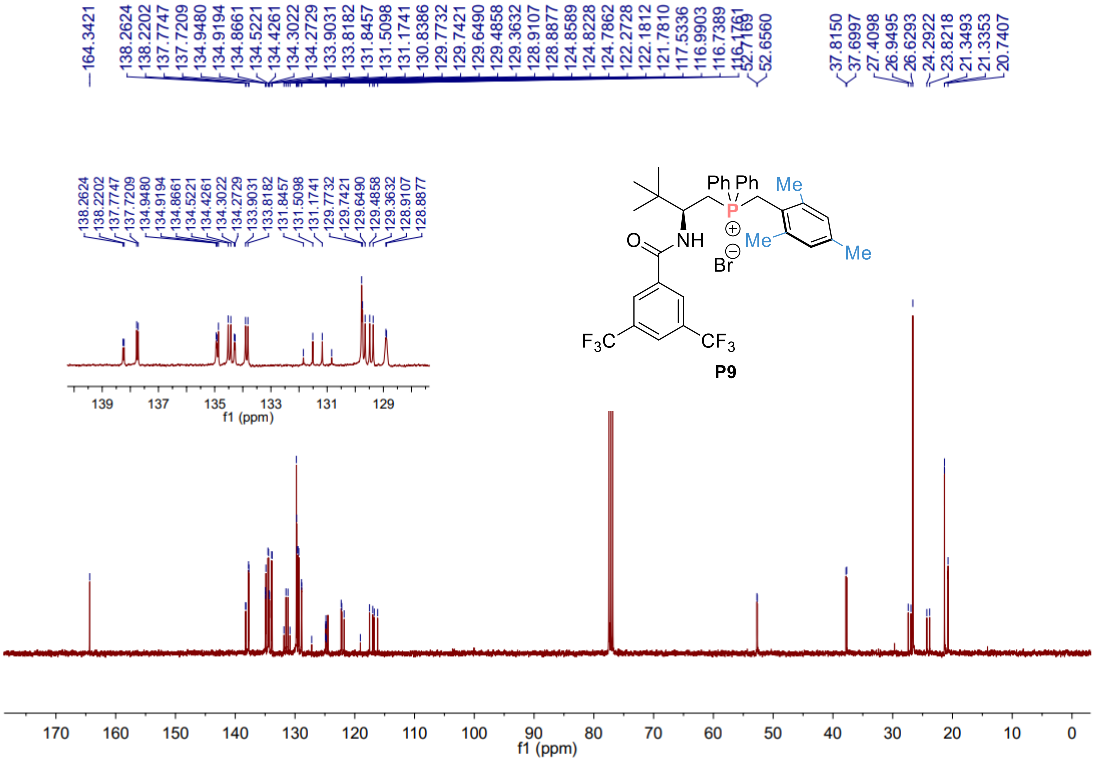
164.3421 138.2624 138.2202 137.7747 137.7209 134.9480 134.9194 134.8661 134.5221 134.4261 134.3022 134.2729 133.9031 133.8182 131.8457 131.5098 131.1741 130.8386 129.7732 129.7421 129.6490 129.4858 129.3632 128.9107 128.8877 124.8589 124.8228 124.7862 122.2728 122.1812 121.7810 117.5336 116.9903 116.7389 116.1761 52.7169 52.6560 37.8150 37.6997 27.4098 26.9495 26.6293 24.2922 23.8218 21.3493 21.3353 20.7407
Ph Ph
Me
P
NH
Me
O
Br
Me
F3C
CF3
P9
f1 (ppm)

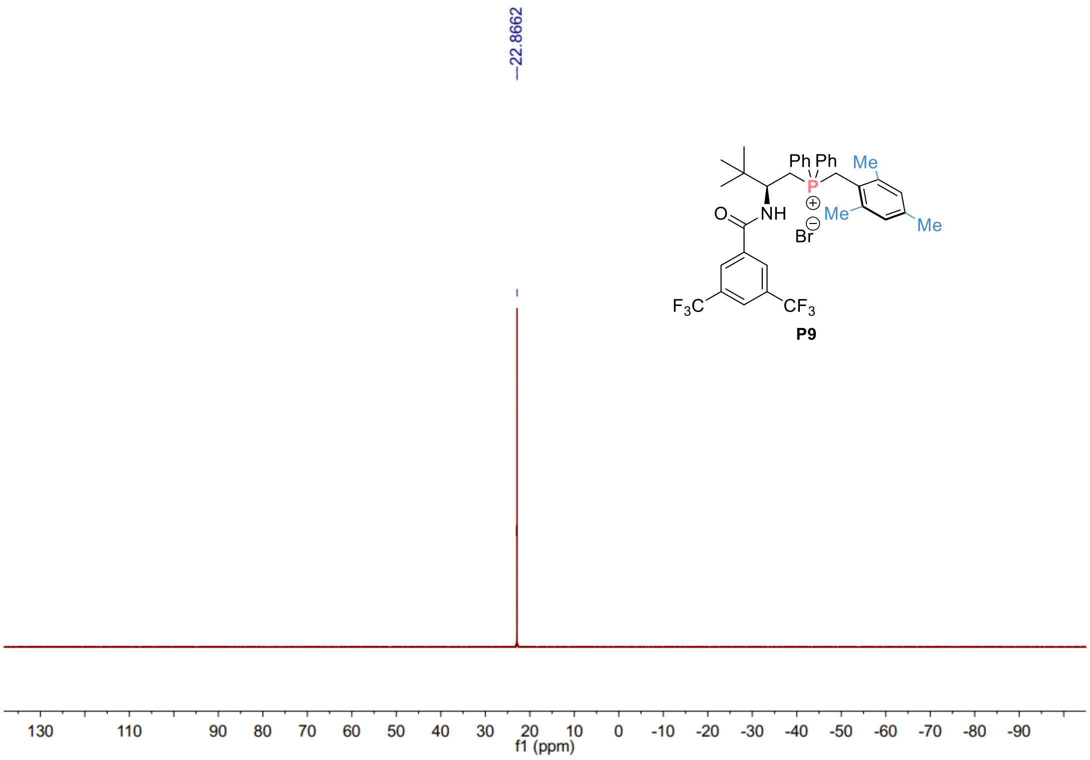

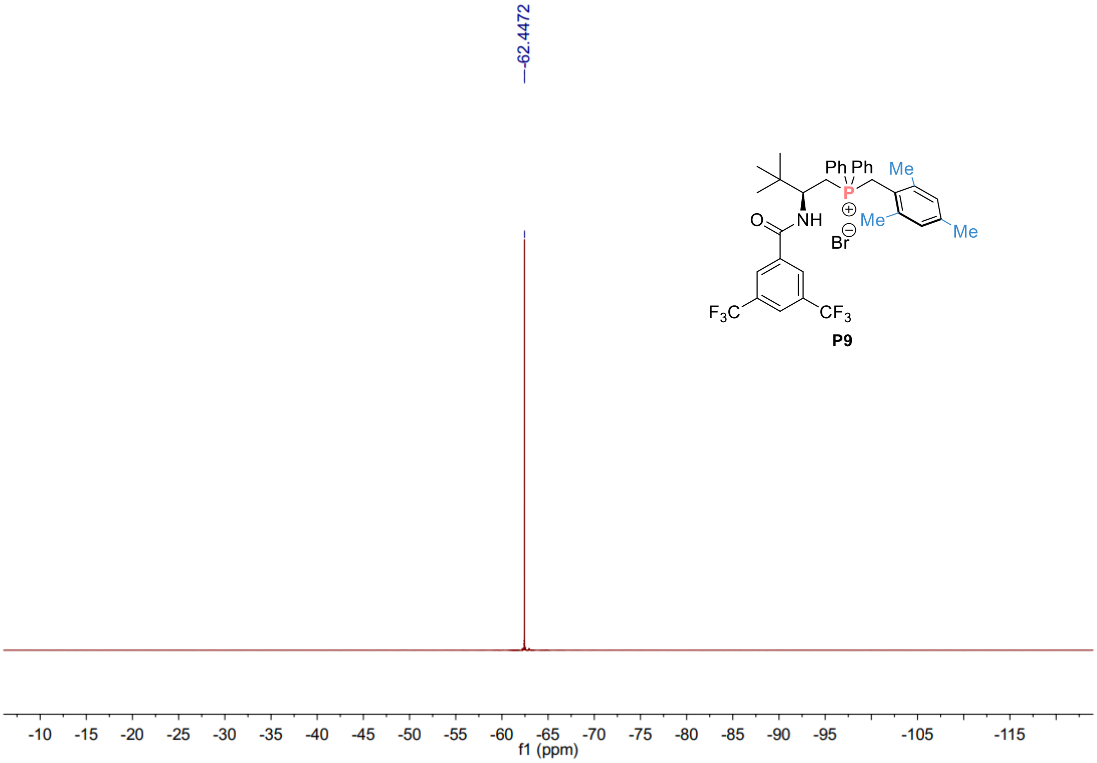

^{1}H-NMR and ^{13}C-NMR and ^{31}P-NMR and ^{19}F-NMR of **P9**

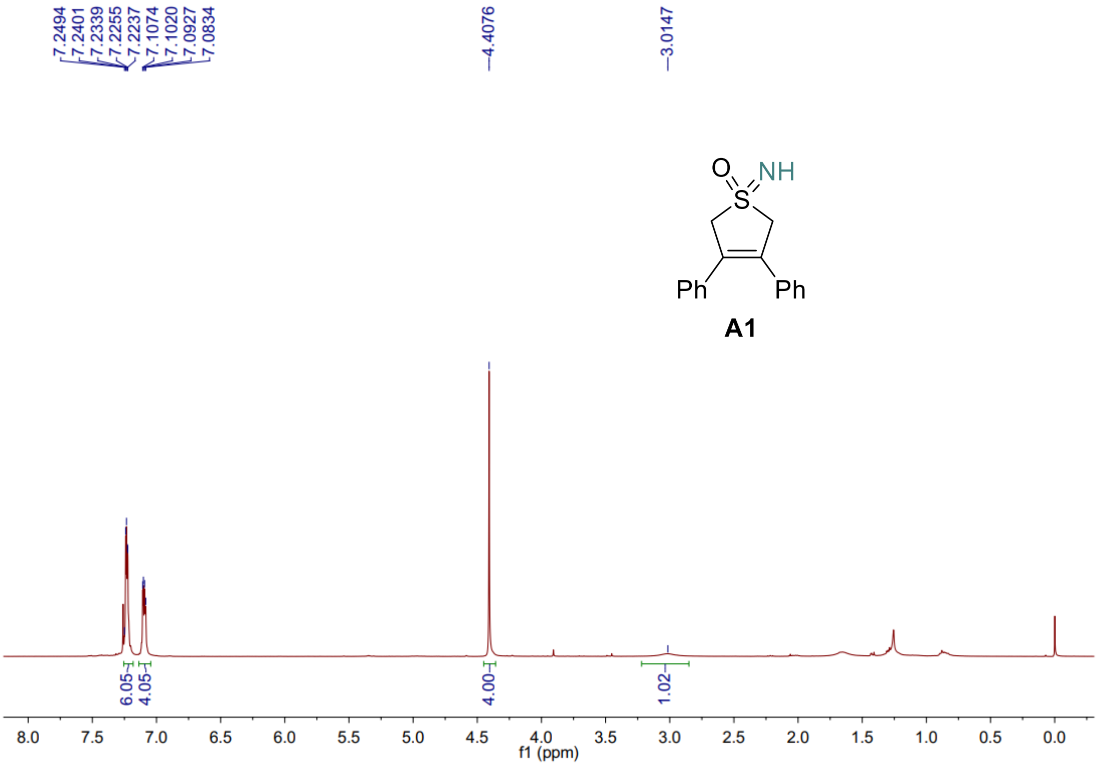

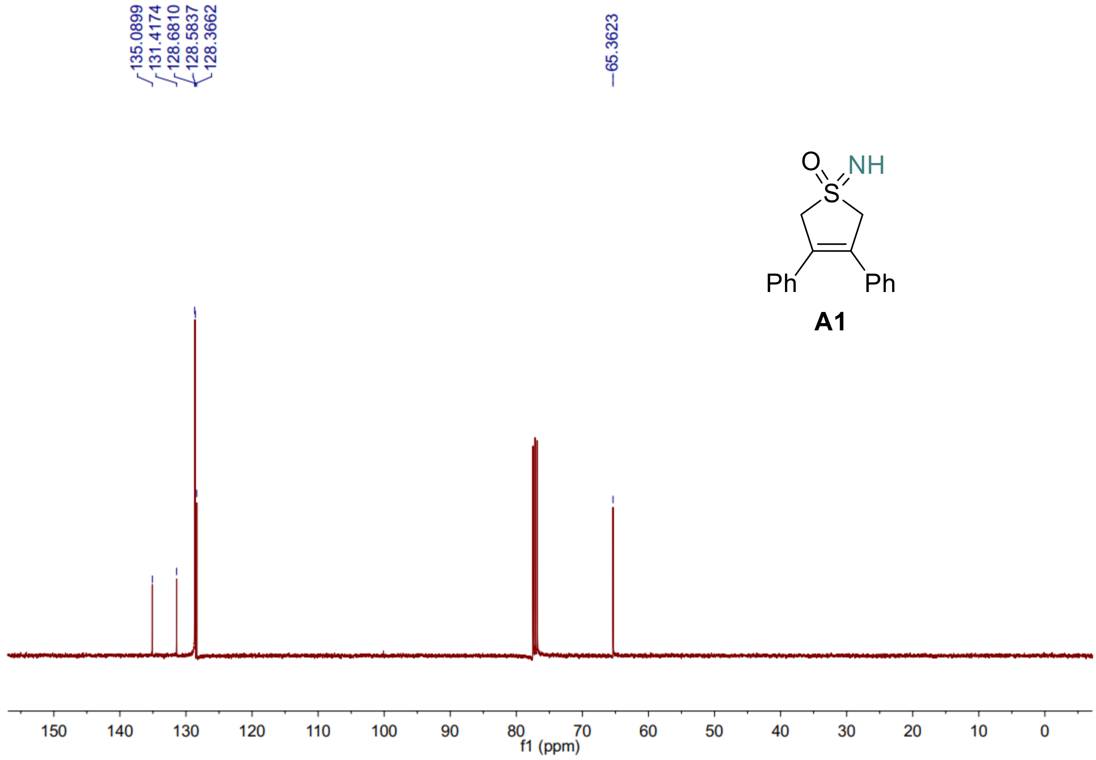

^{1}H-NMR and ^{13}C-NMR of **A1**

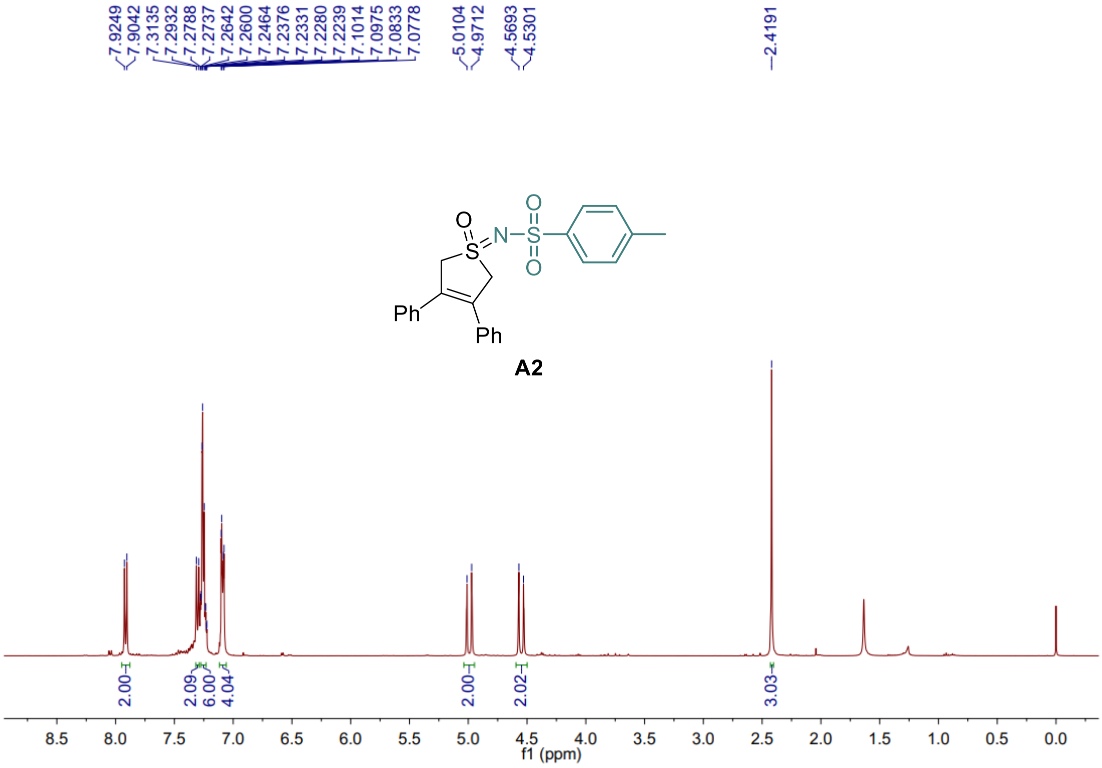

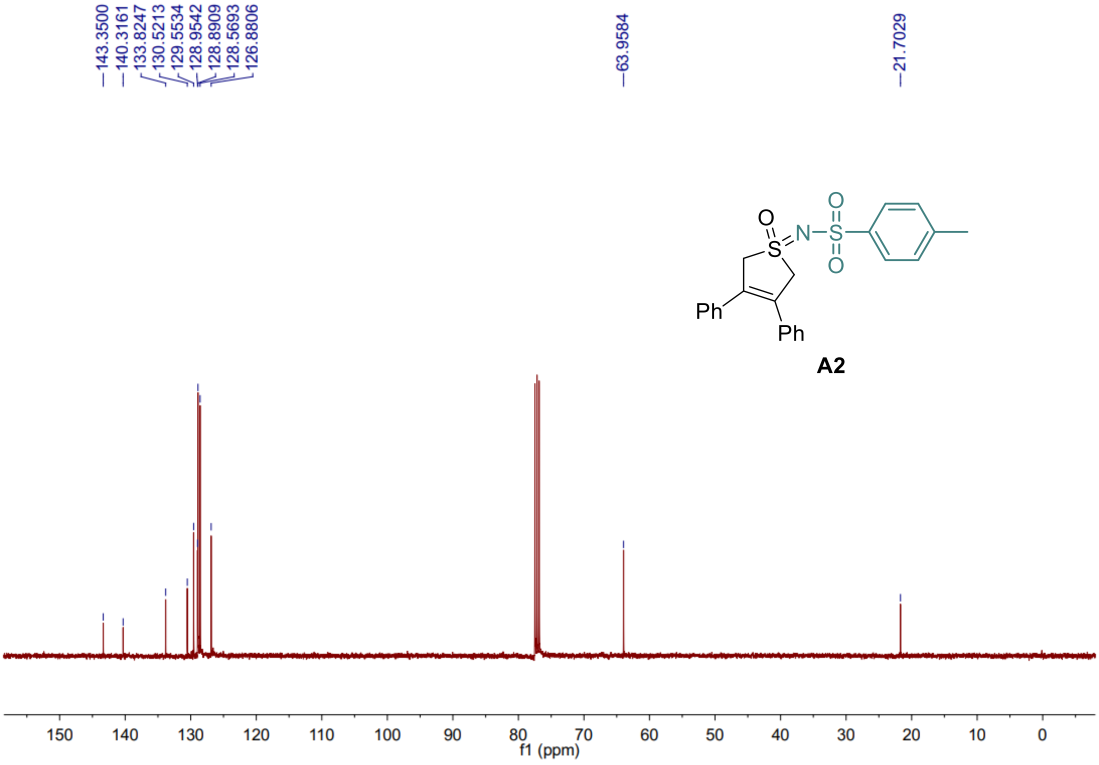

^{1}H-NMR and ^{13}C-NMR of **A2**

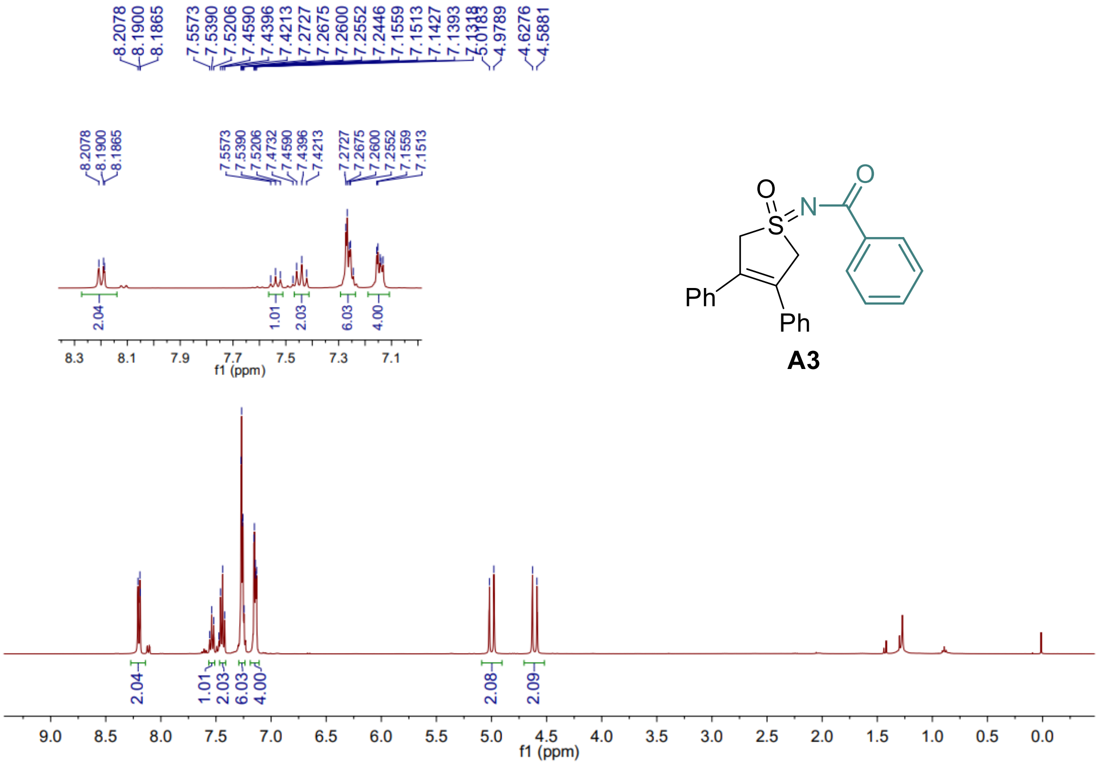

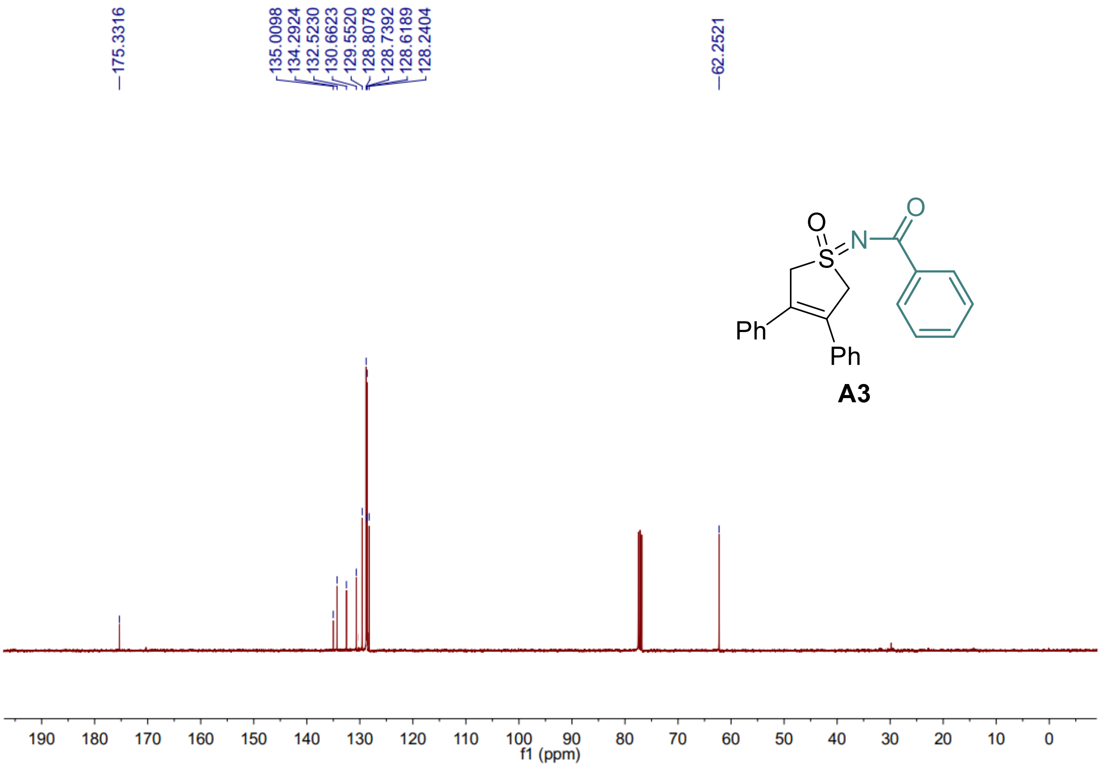

^{1}H-NMR and ^{13}C-NMR of **A3**

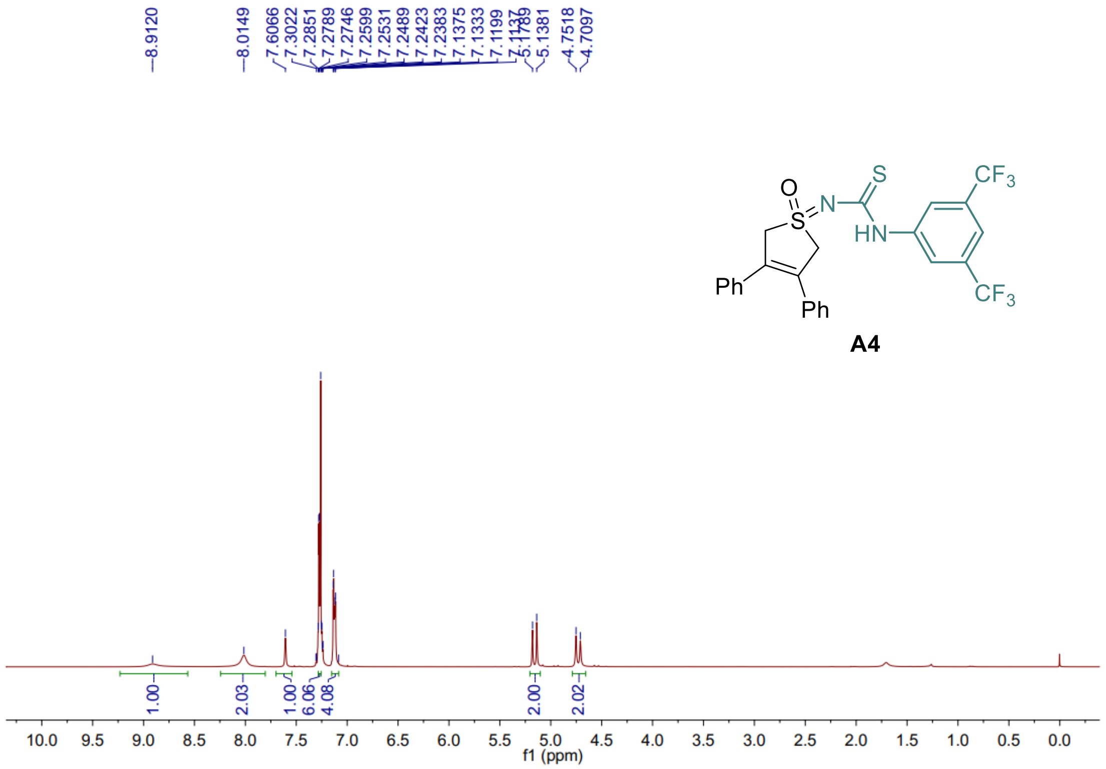
8.9120
8.0149
7.6066
7.3022
7.2851
7.2789
7.2746
7.2599
7.2531
7.2489
7.2423
7.2383
7.1375
7.1333
7.1199
7.1137
5.1789
5.1381
4.7518
4.7097
O
S
N
S
HN
CF_3
CF_3
Ph
Ph
A4
1.00
2.03
1.00
6.06
4.08
2.00
2.02
10.0 9.5 9.0 8.5 8.0 7.5 7.0 6.5 6.0 5.5 5.0 4.5 4.0 3.5 3.0 2.5 2.0 1.5 1.0 0.5 0.0
f1 (ppm)

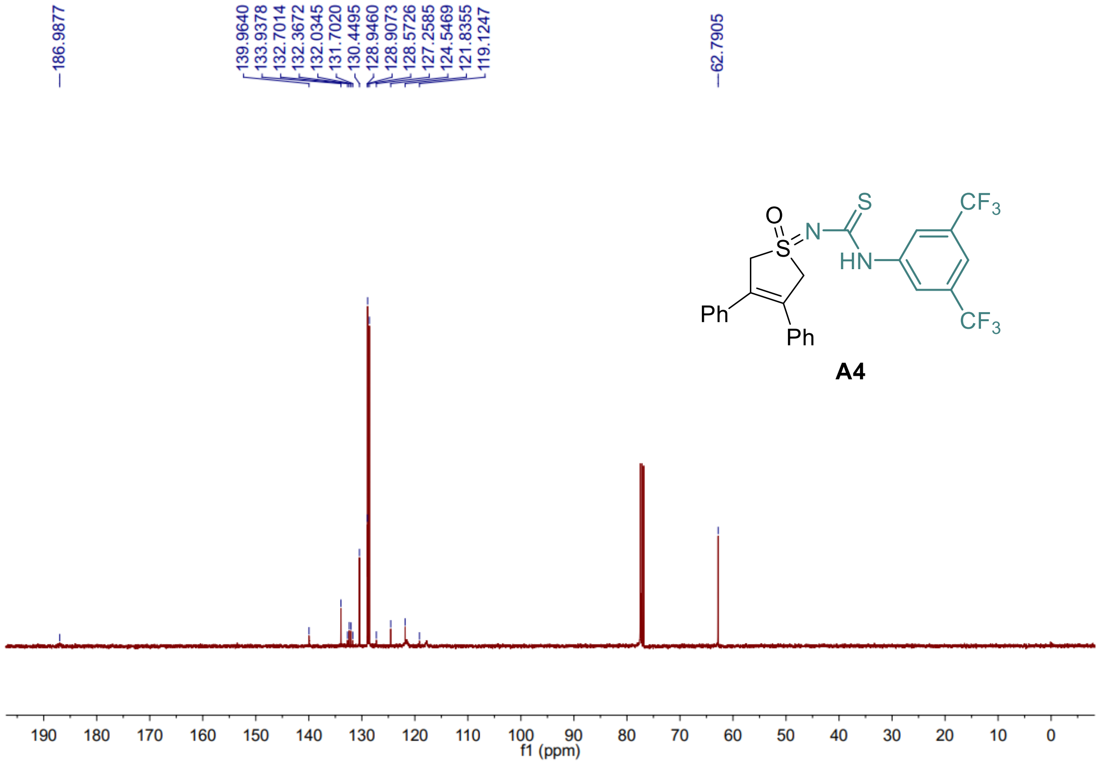
186.9877
139.9640
133.9378
132.7014
132.3672
132.0345
131.7020
130.4495
128.9460
128.9073
128.5726
127.2585
124.5469
121.8355
119.1247
62.7905
O
S
N
S
HN
CF_3
CF_3
Ph
Ph
A4
190 180 170 160 150 140 130 120 110 100 90 80 70 60 50 40 30 20 10 0
f1 (ppm)

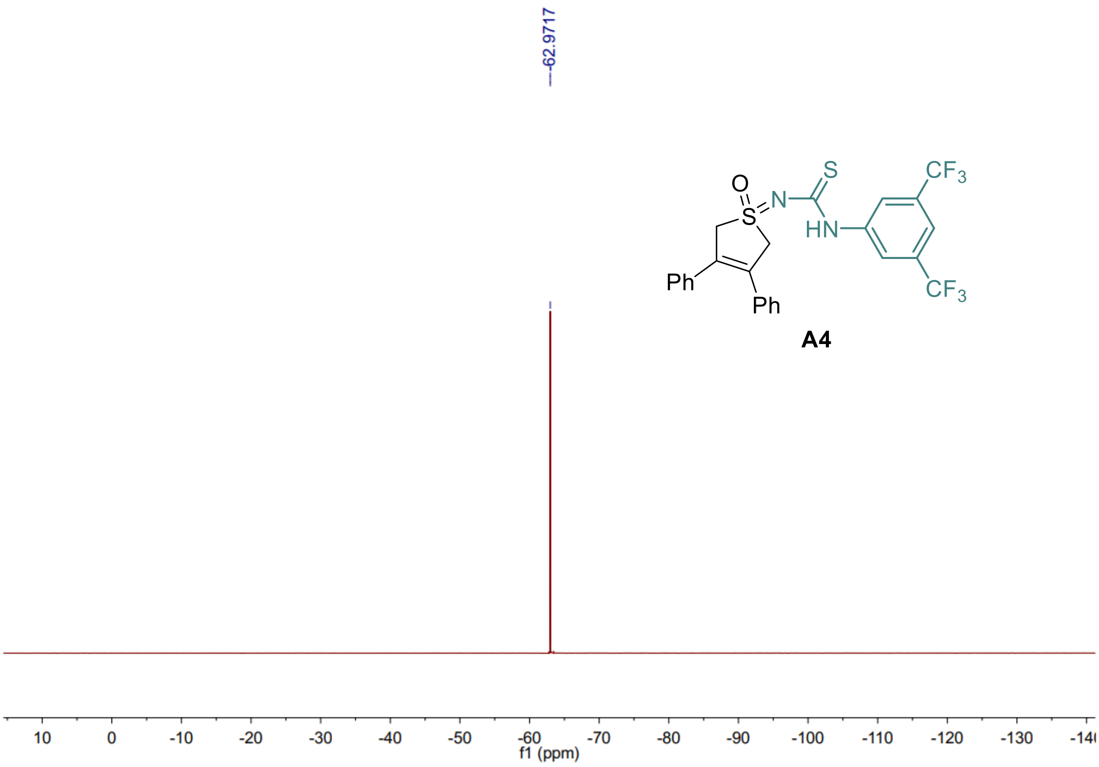

^{1}H-NMR and ^{13}C-NMR and ^{19}F-NMR of **A4**

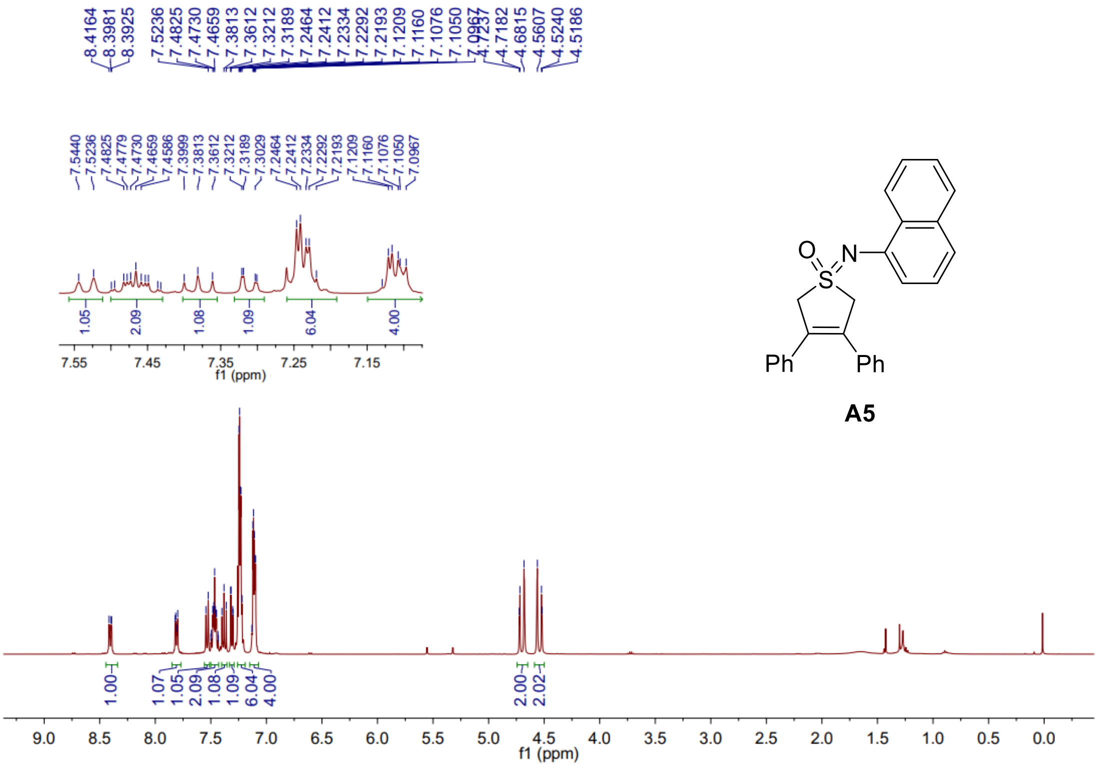

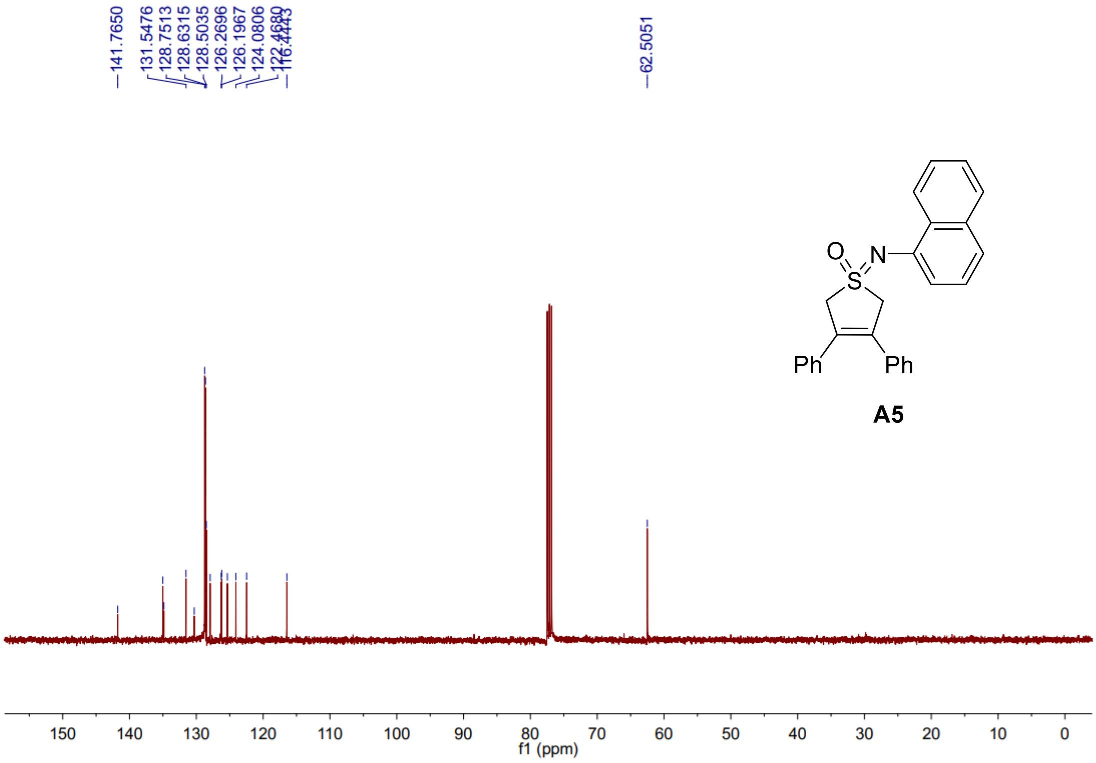

^{1}H-NMR and ^{13}C-NMR of **A5**

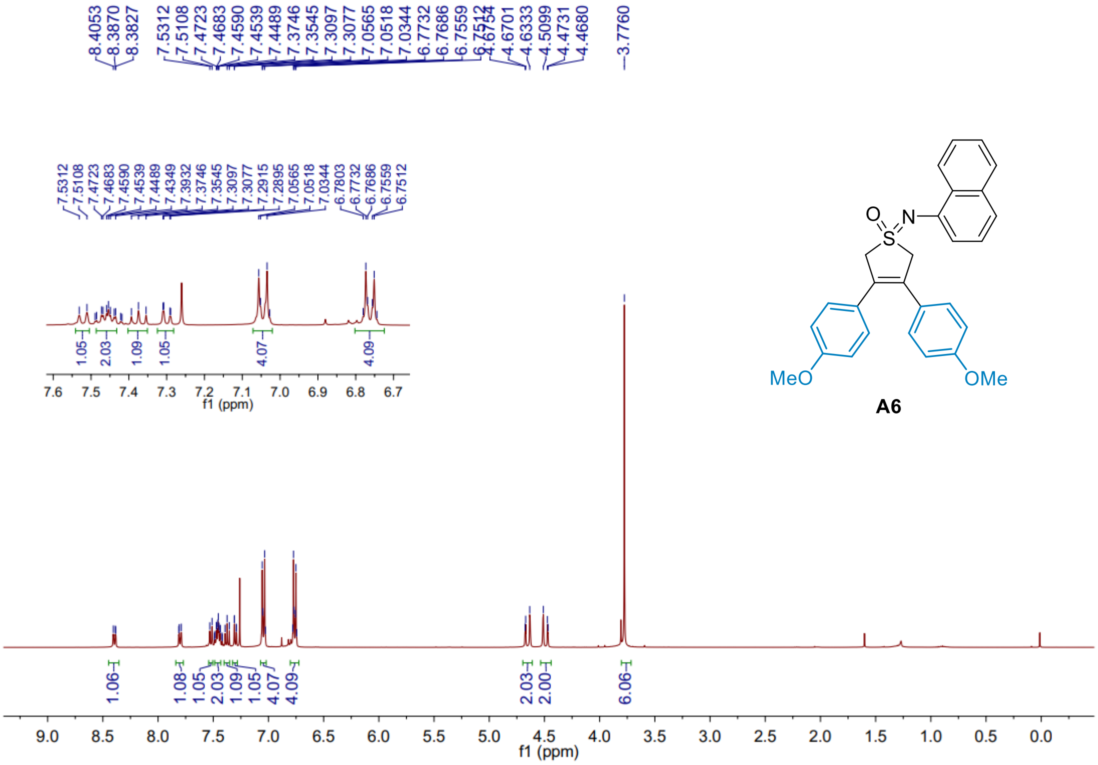

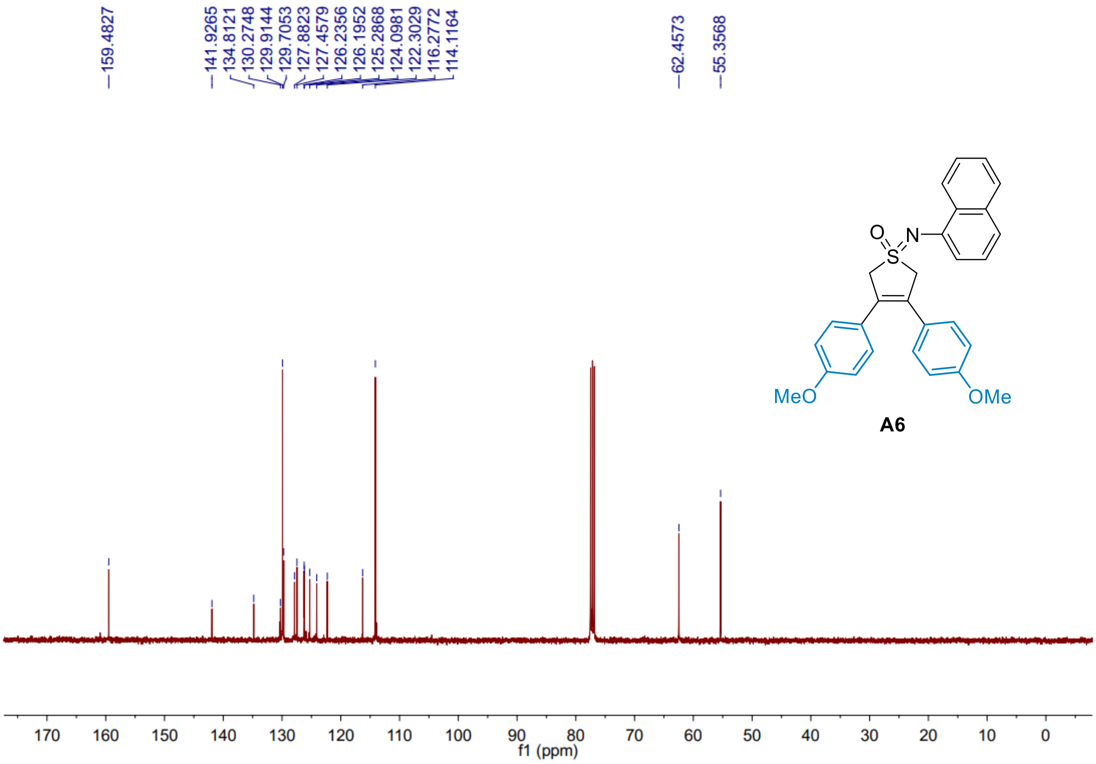

^{1}H-NMR and ^{13}C-NMR of **A6**

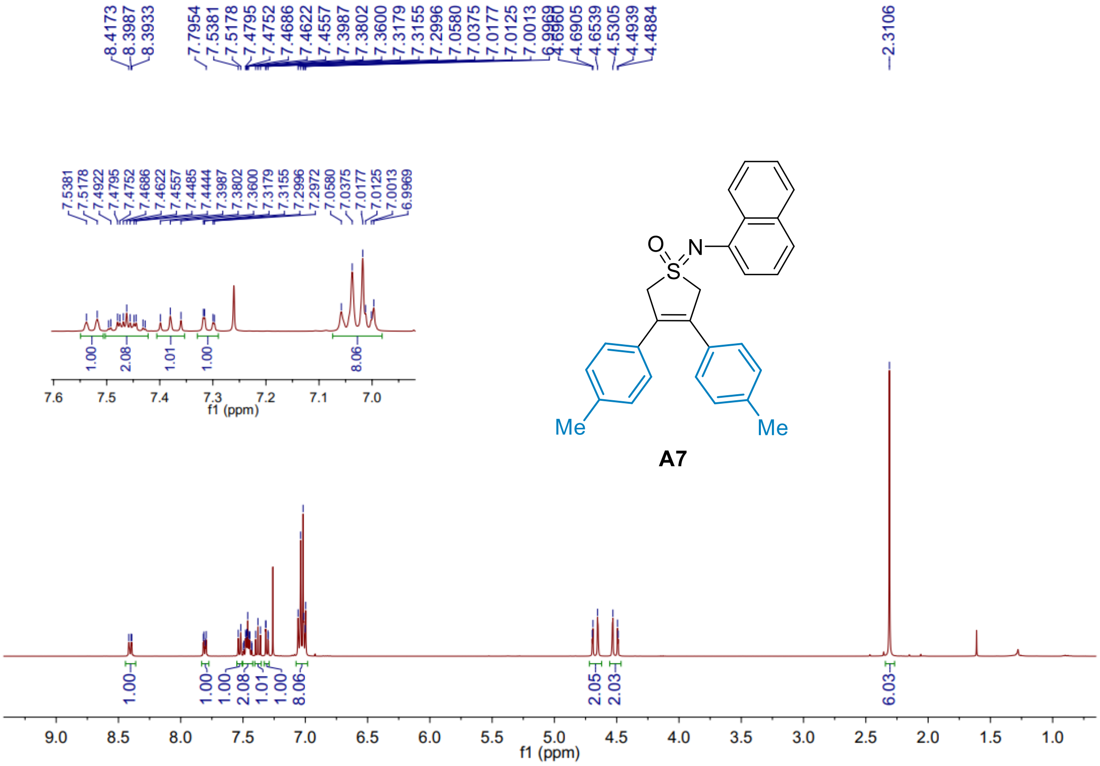

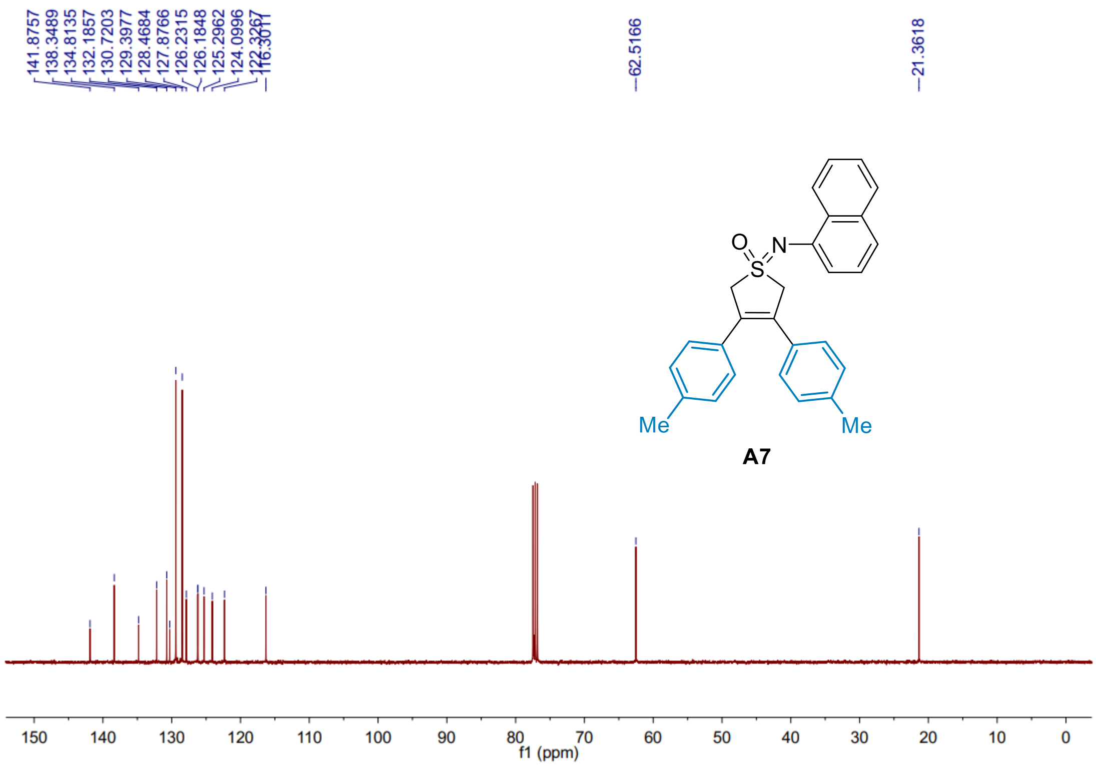

^{1}H-NMR and ^{13}C-NMR of **A7**

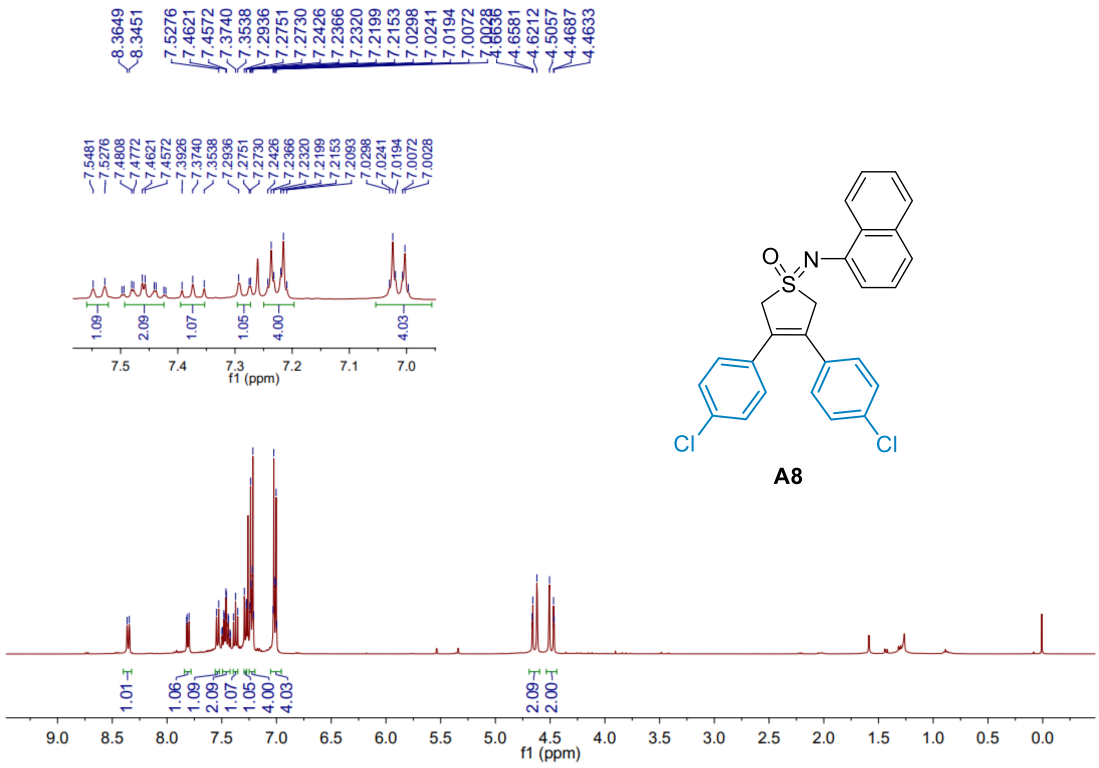

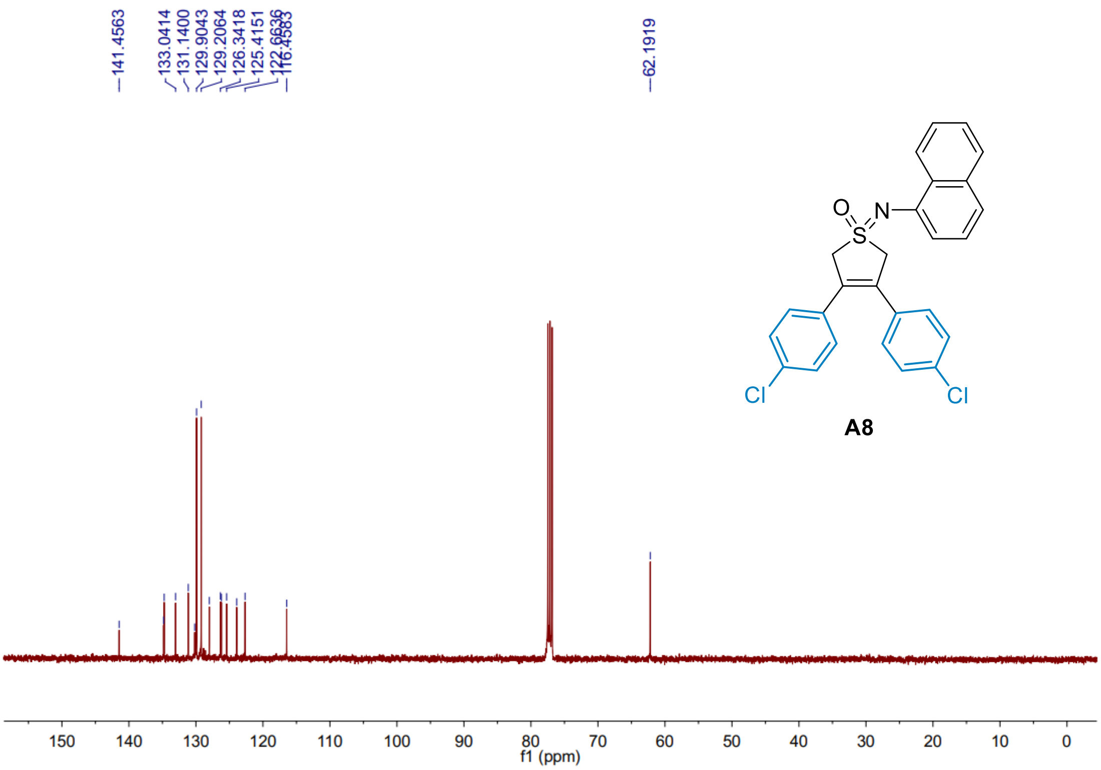

^{1}H-NMR and ^{13}C-NMR of **A8**

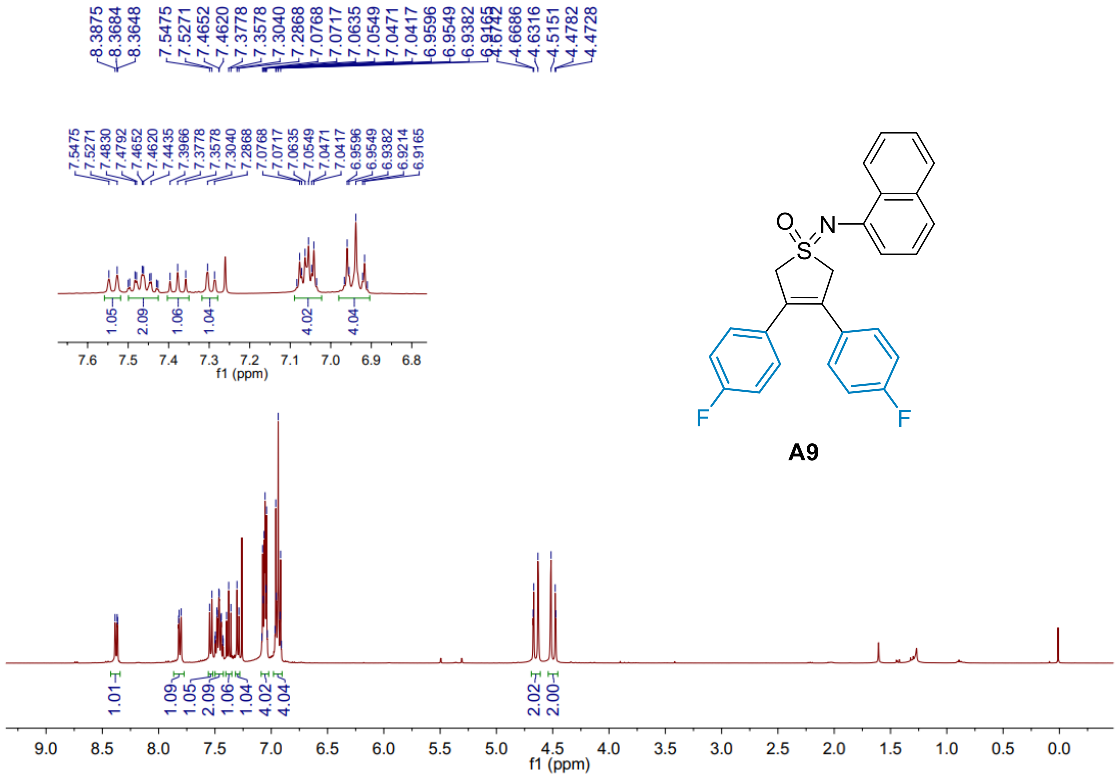

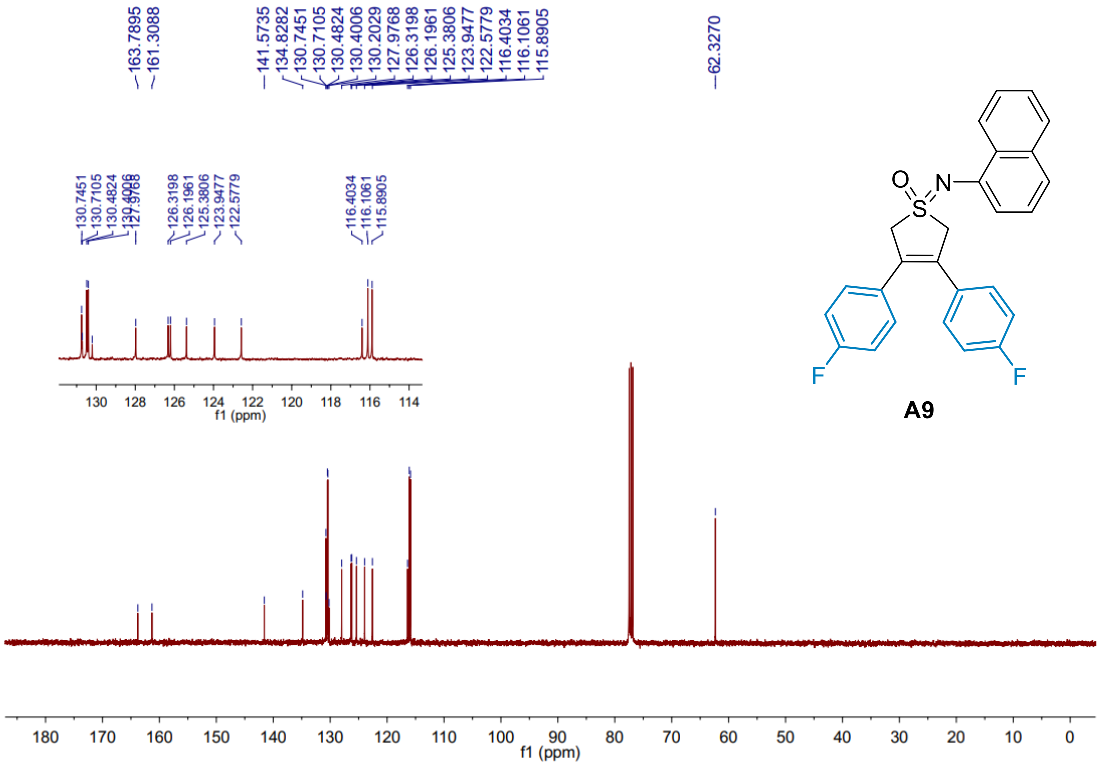

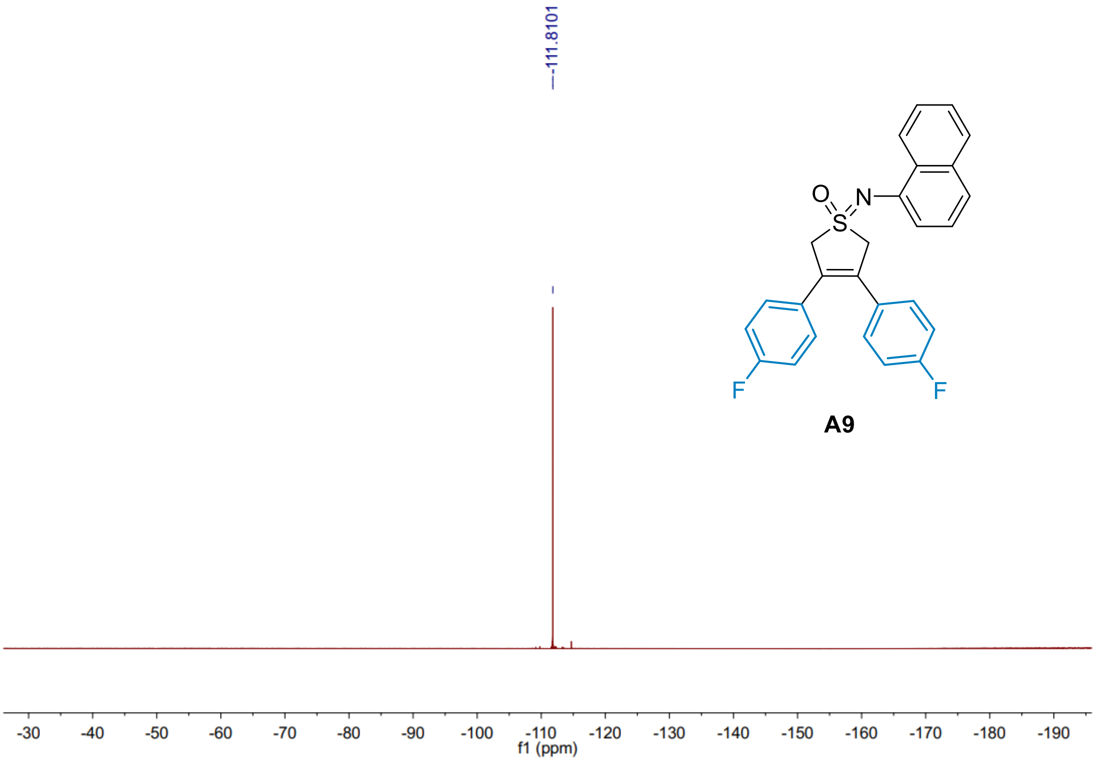

^{1}H-NMR and ^{13}C-NMR and ^{19}F-NMR of **A9**

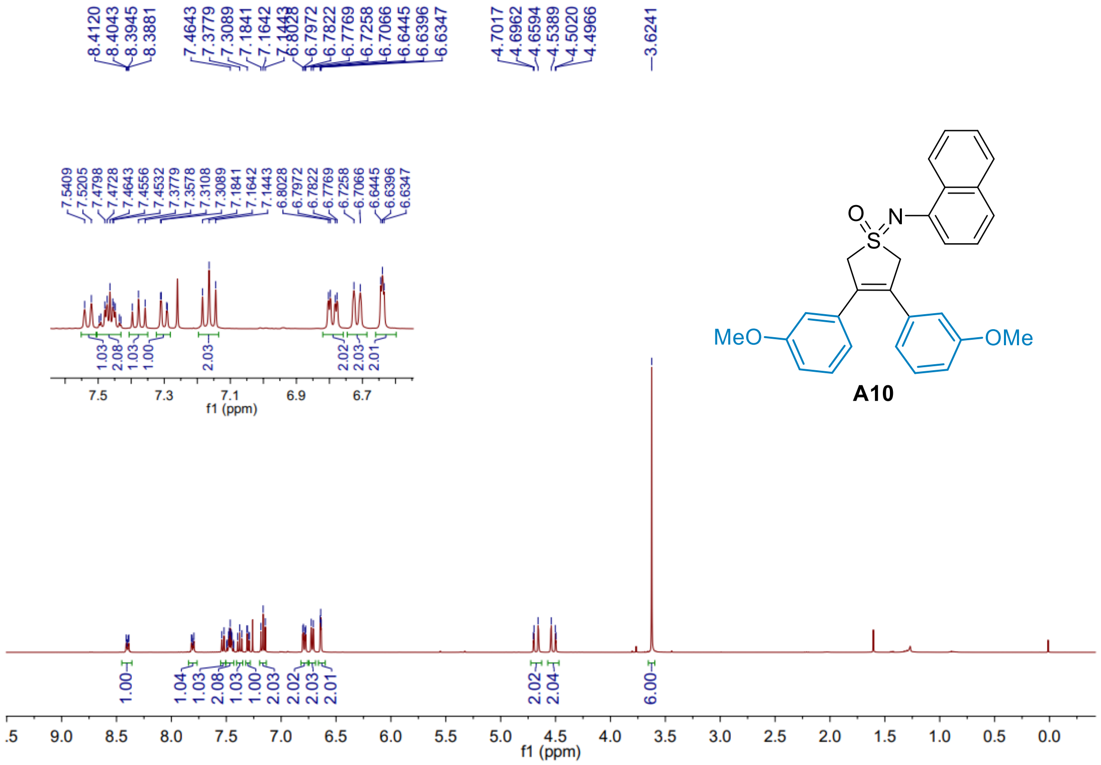

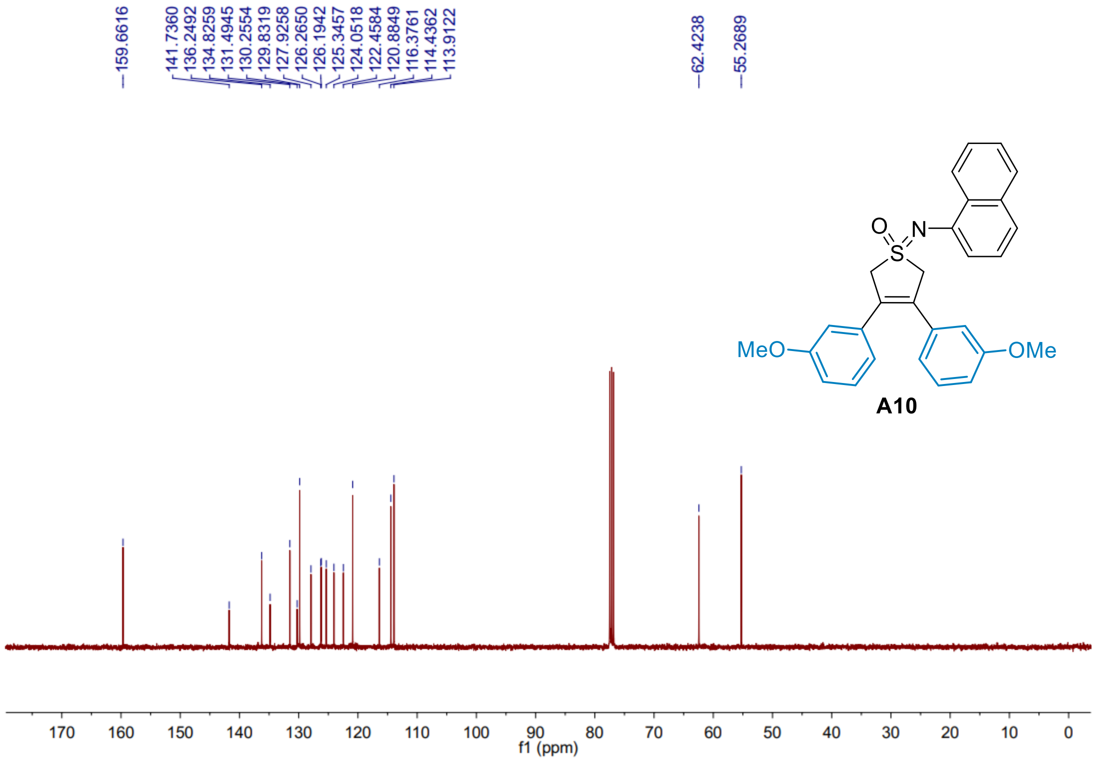

^{1}H-NMR and ^{13}C-NMR of **A10**

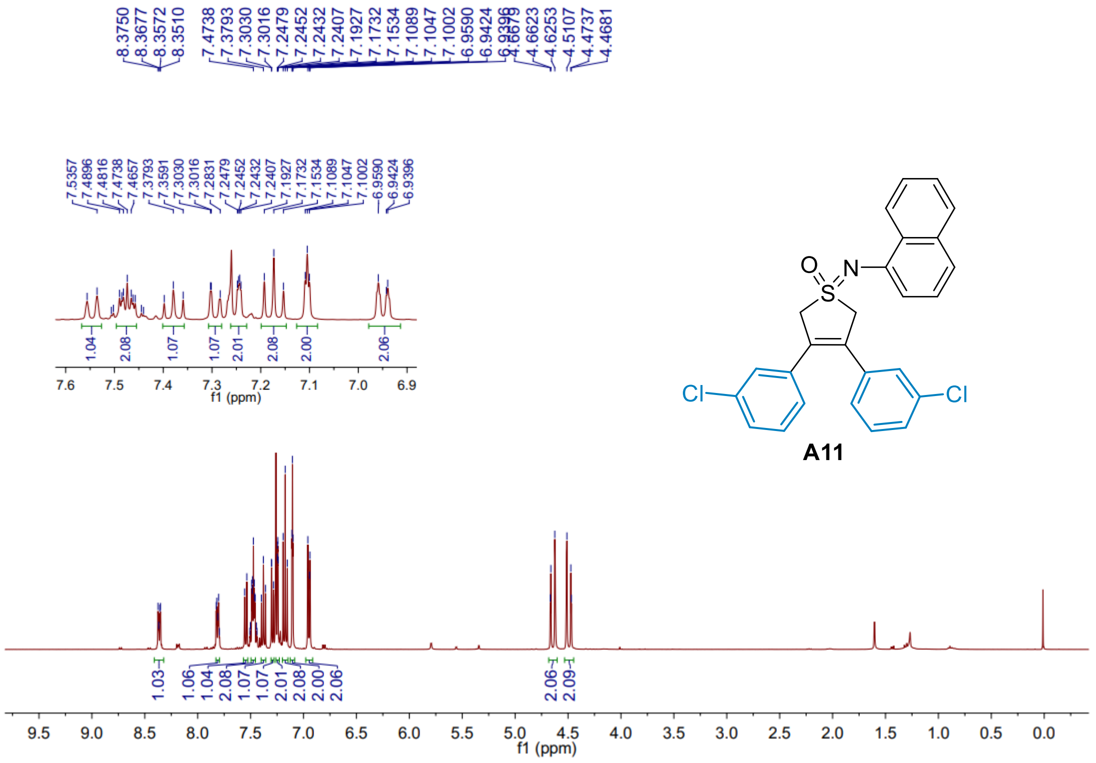

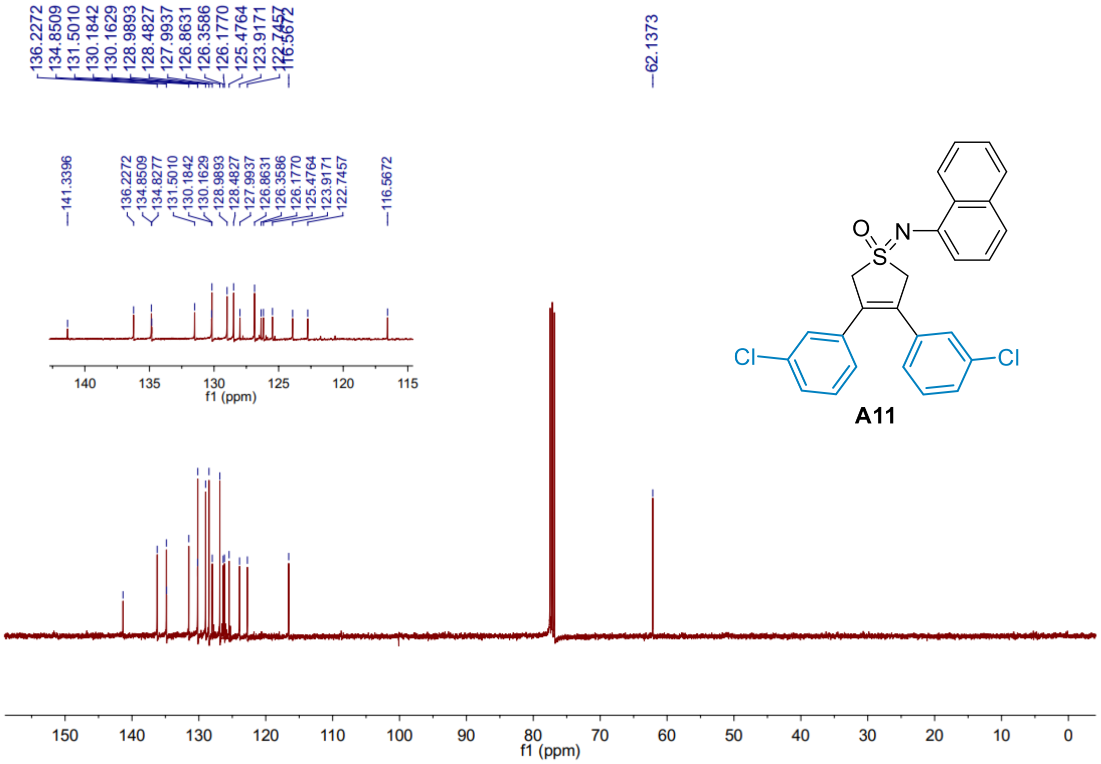

^{1}H-NMR and ^{13}C-NMR of **A11**

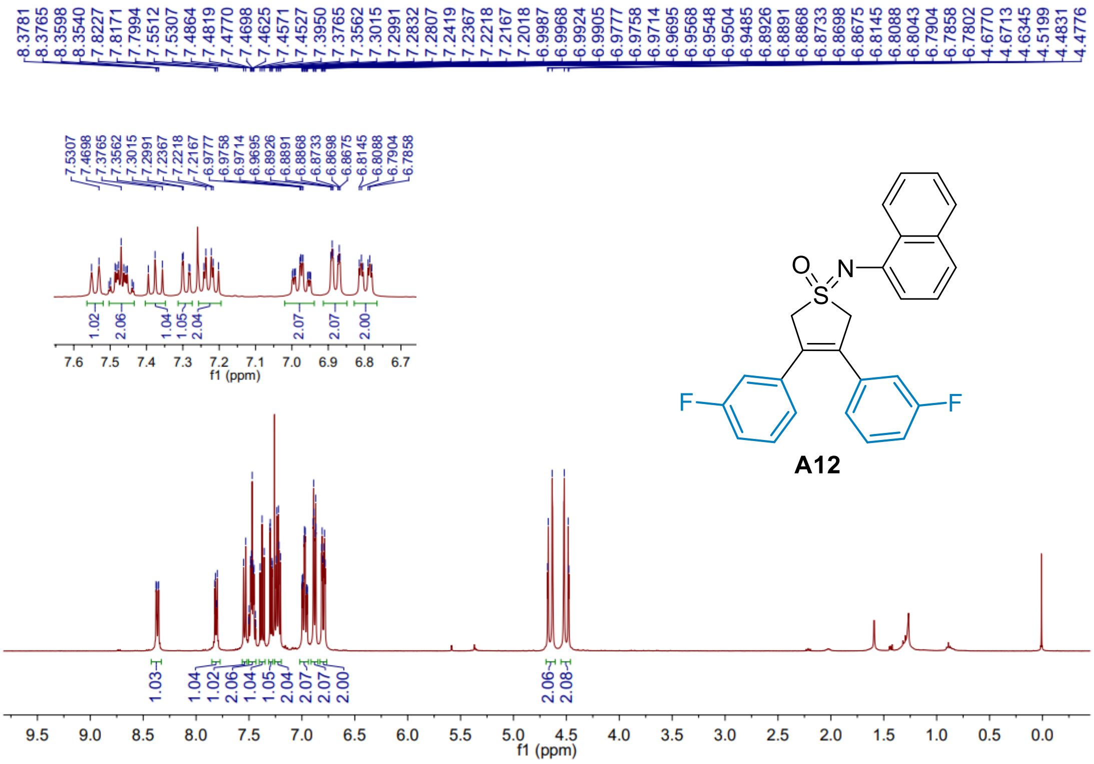

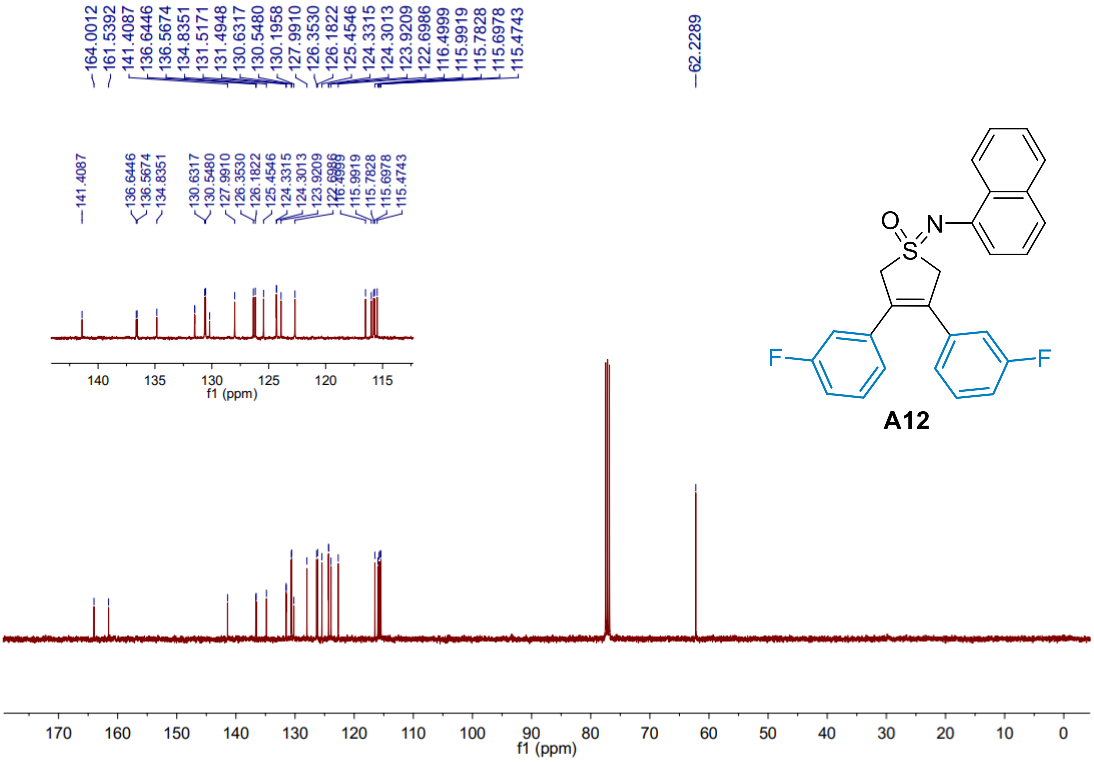

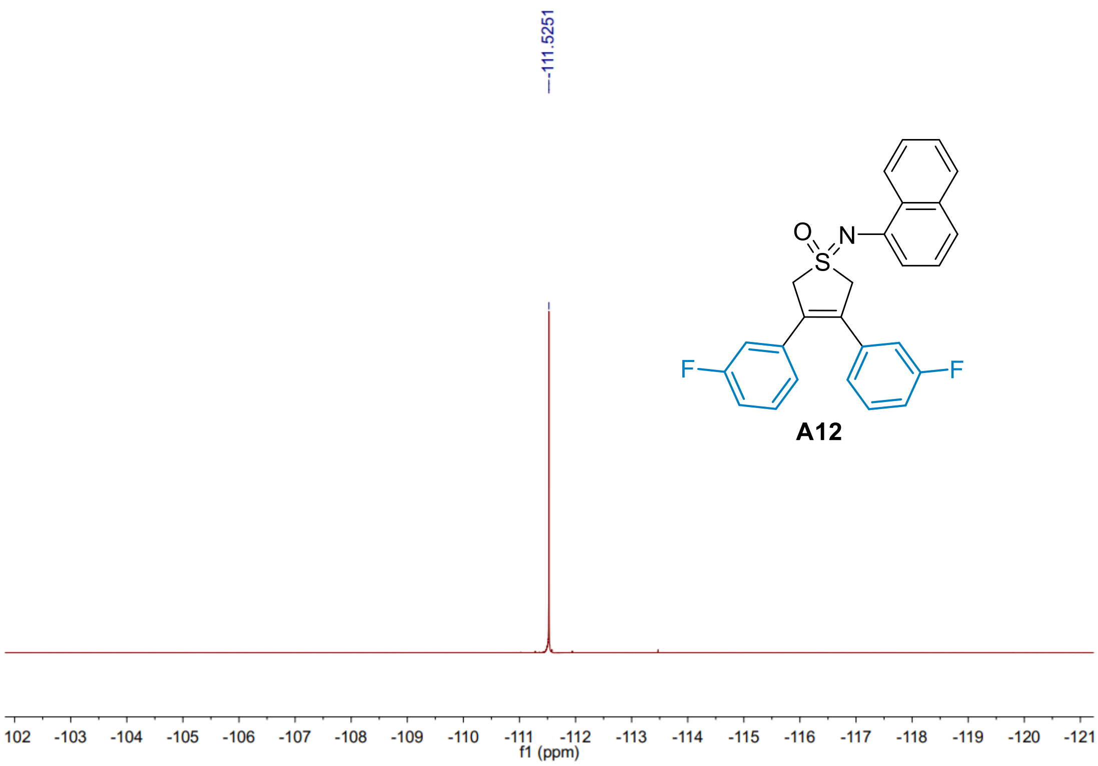

^{1}H-NMR and ^{13}C-NMR and ^{19}F-NMR of **A12**

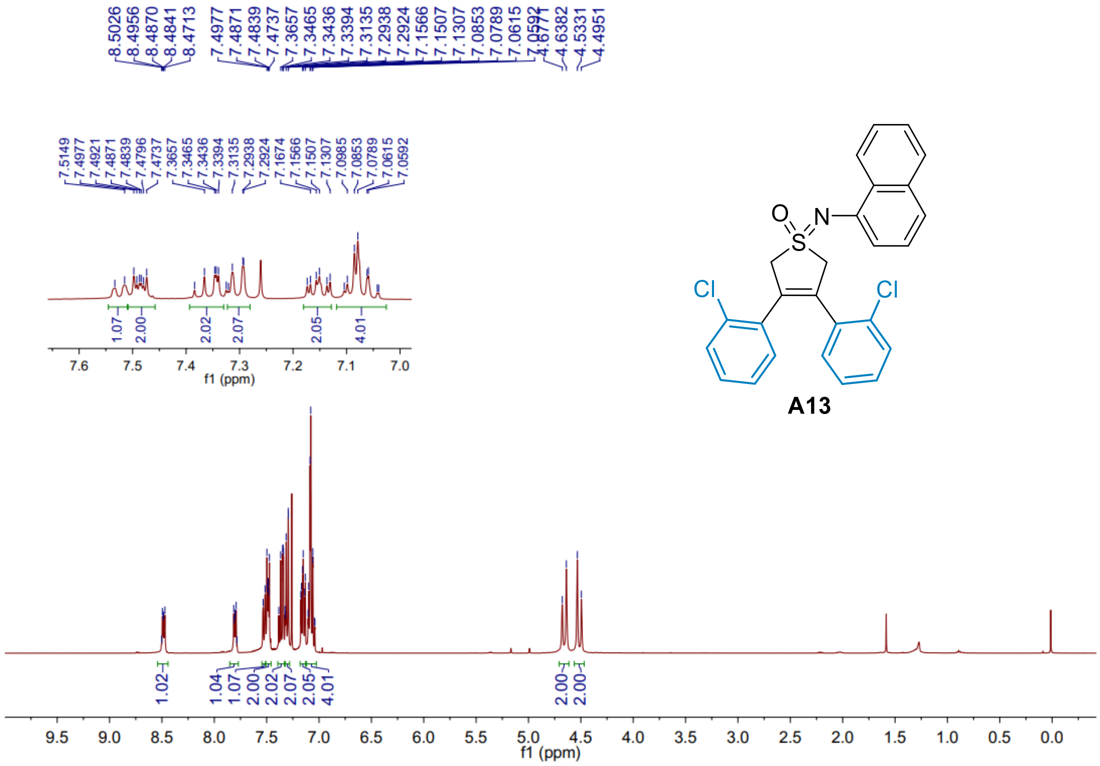

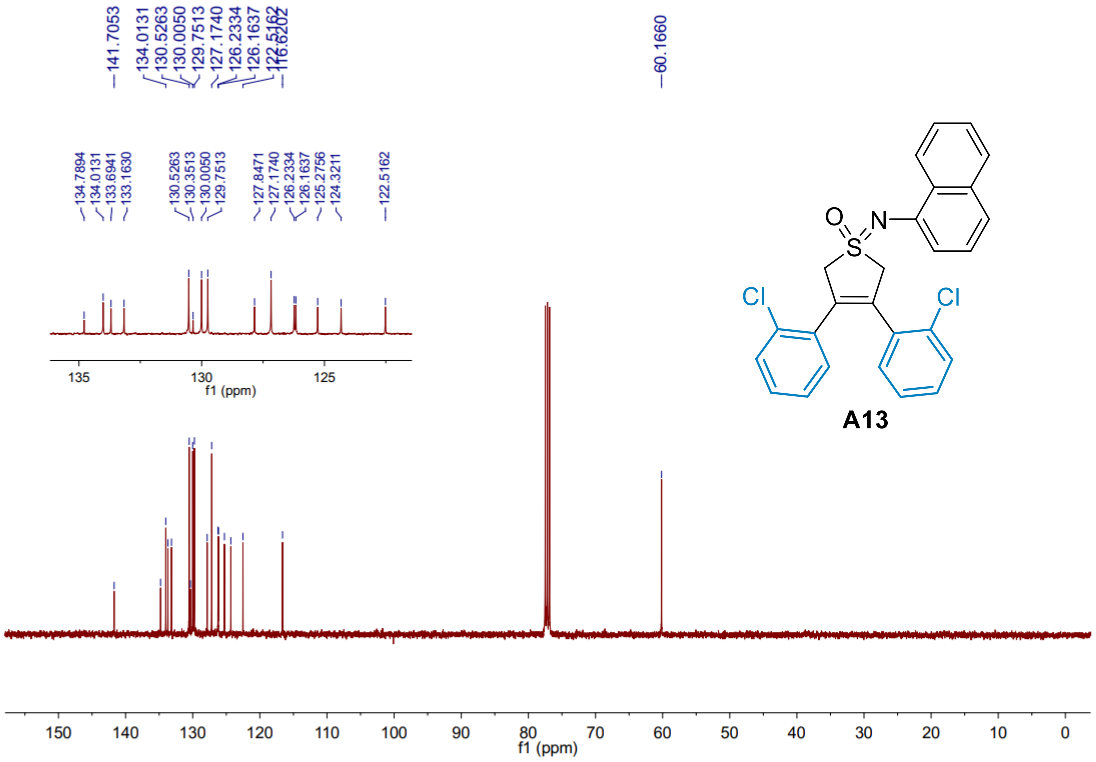

^{1}H-NMR and ^{13}C-NMR of **A13**

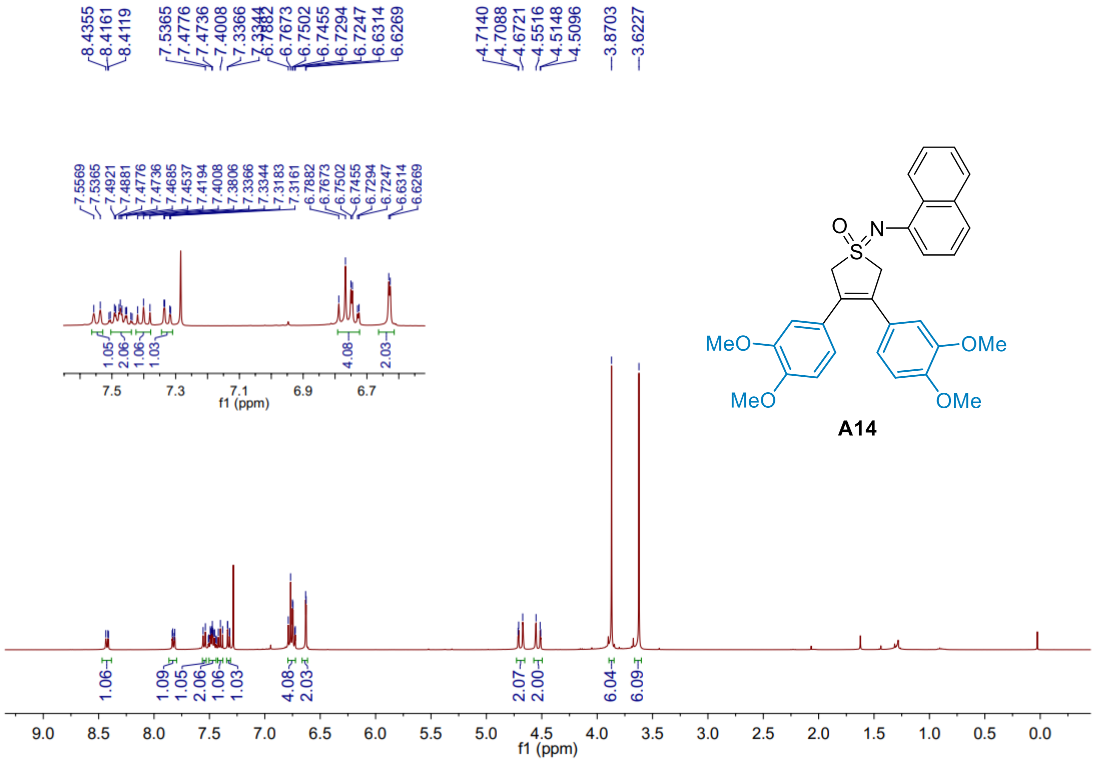

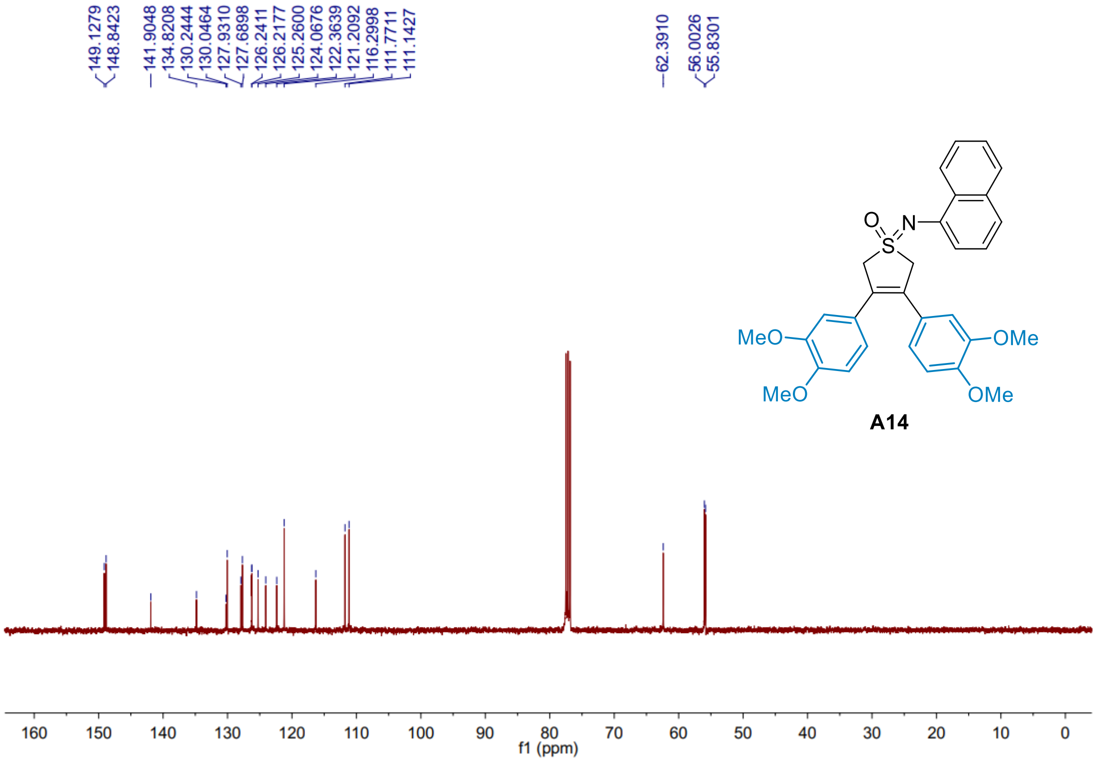

^{1}H-NMR and ^{13}C-NMR of **A14**

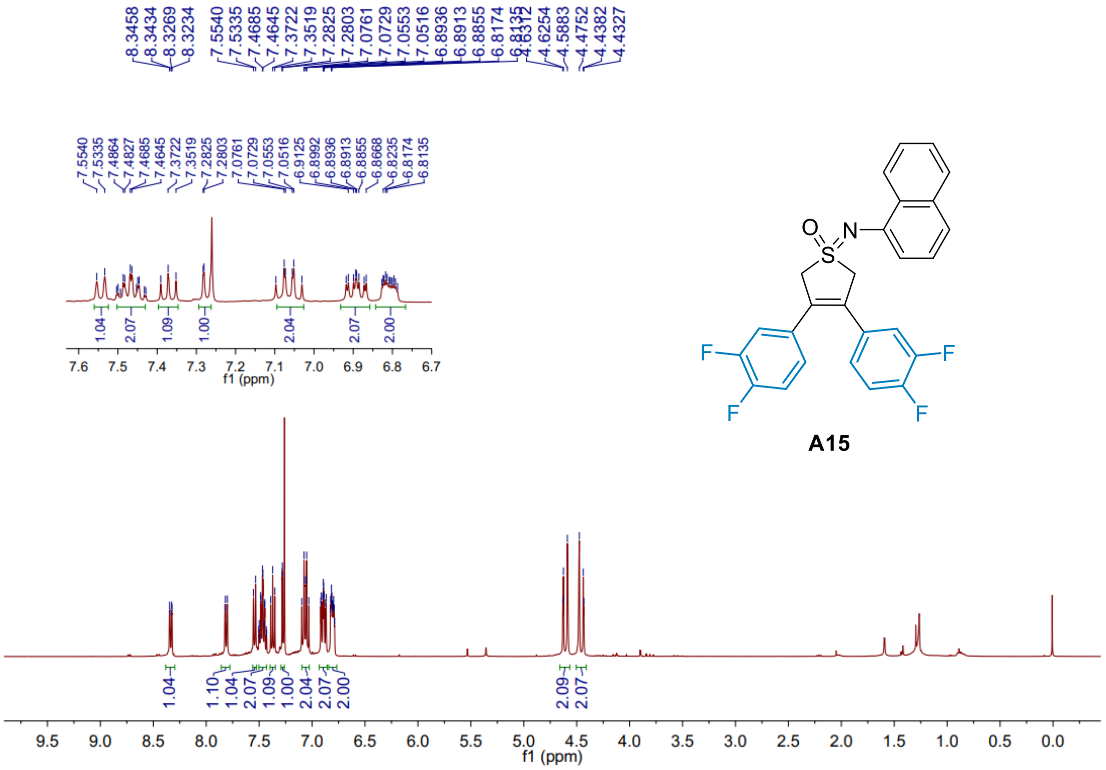

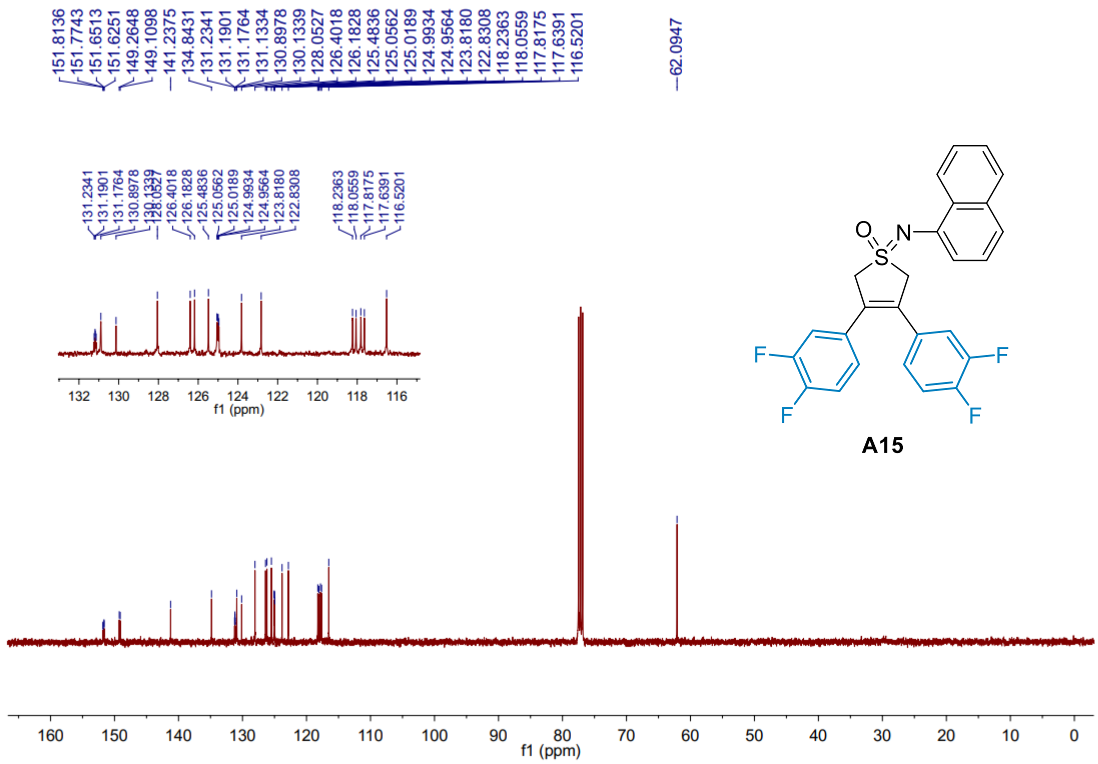

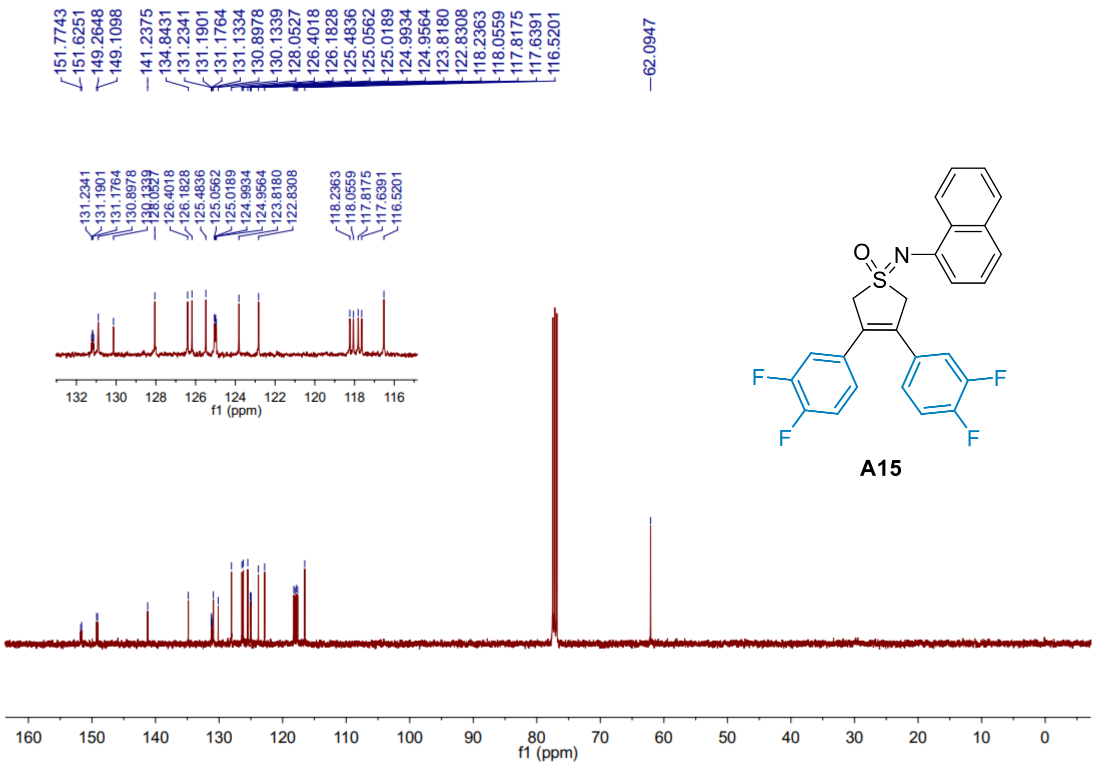

^{1}H-NMR and ^{13}C-NMR and ^{19}F-NMR of **A15**

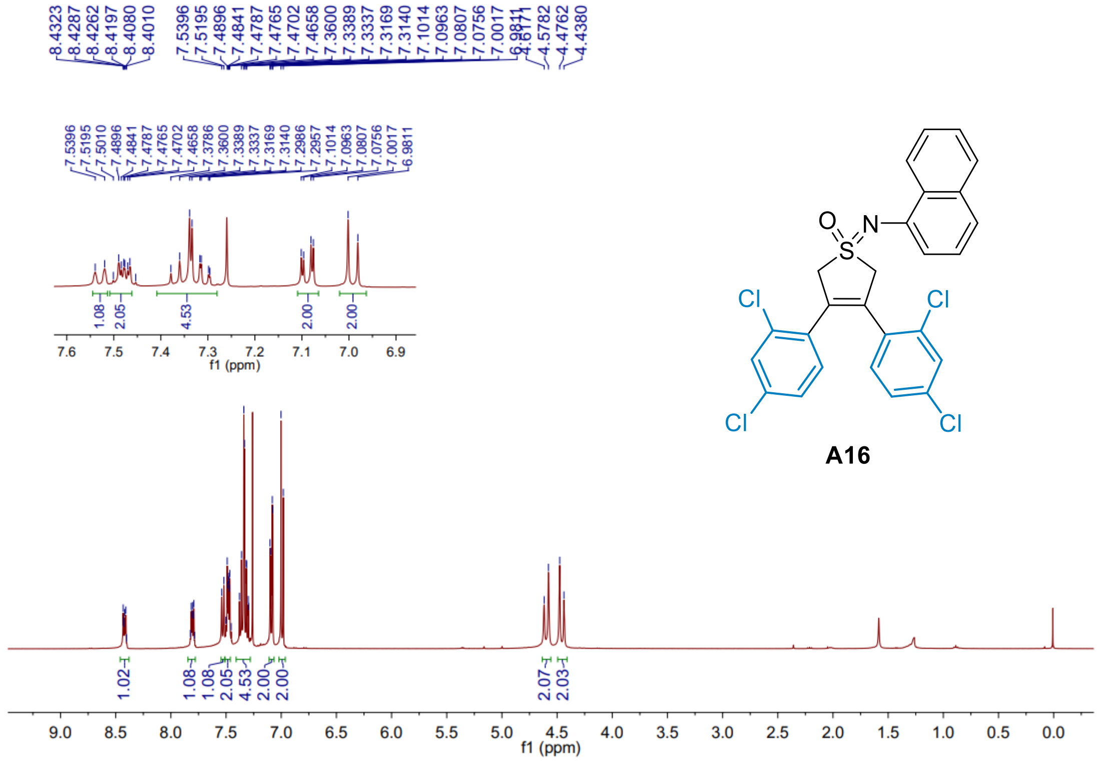

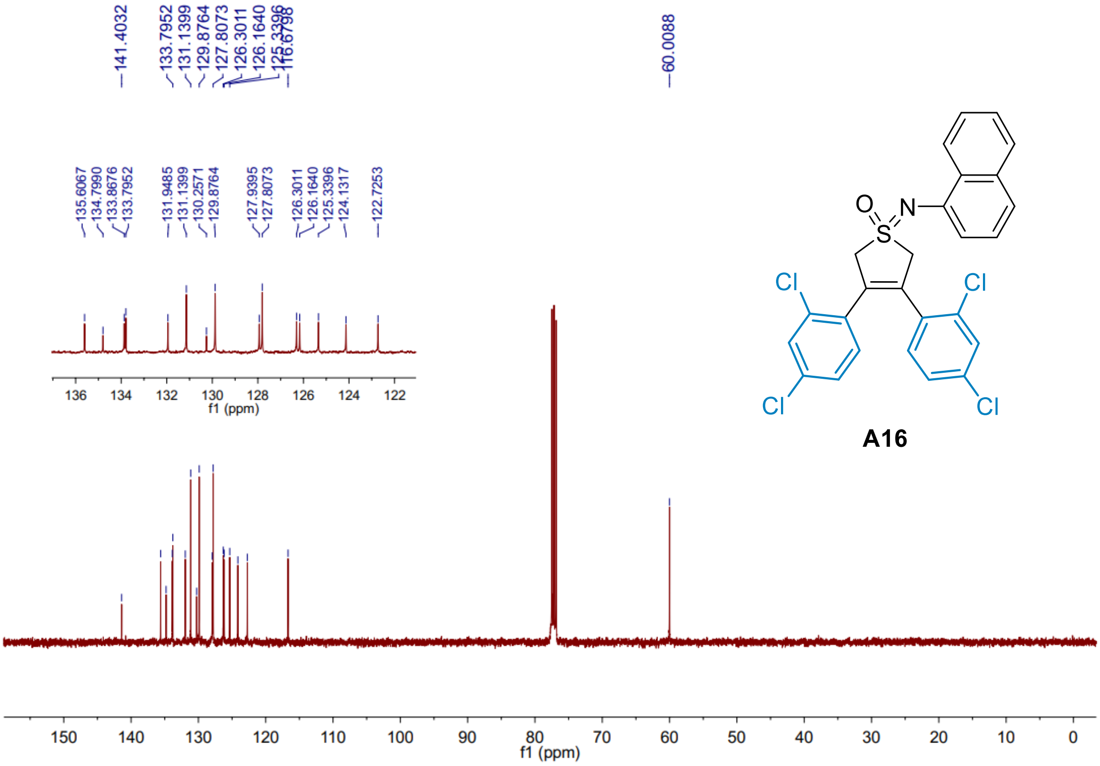

^{1}H-NMR and ^{13}C-NMR of **A16**

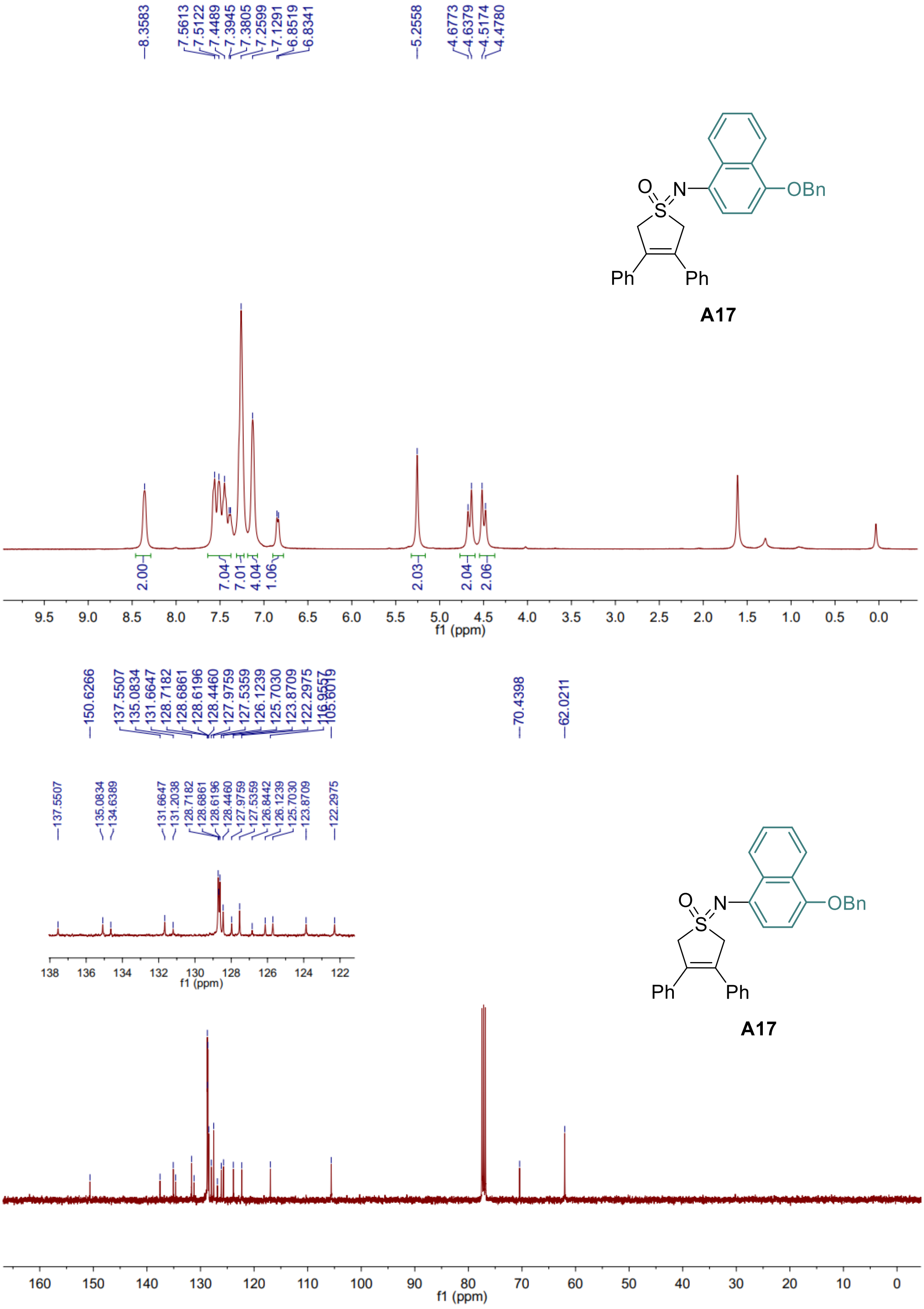

^{1}H-NMR and ^{13}C-NMR of **A17**

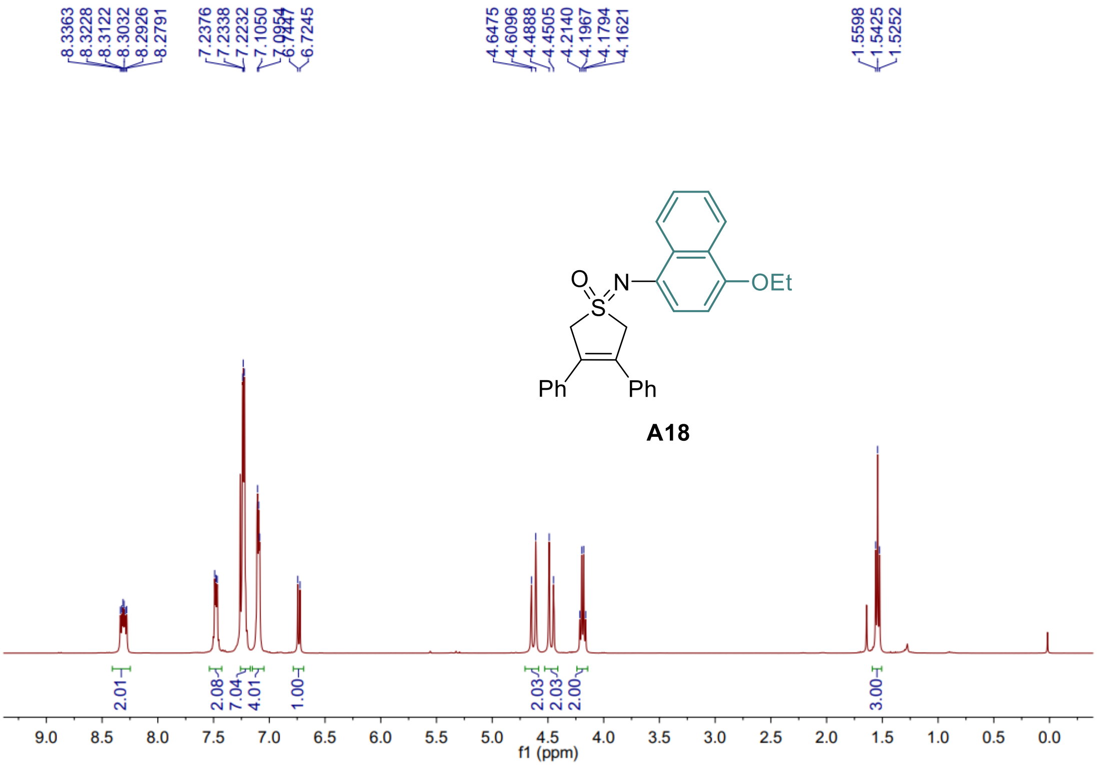

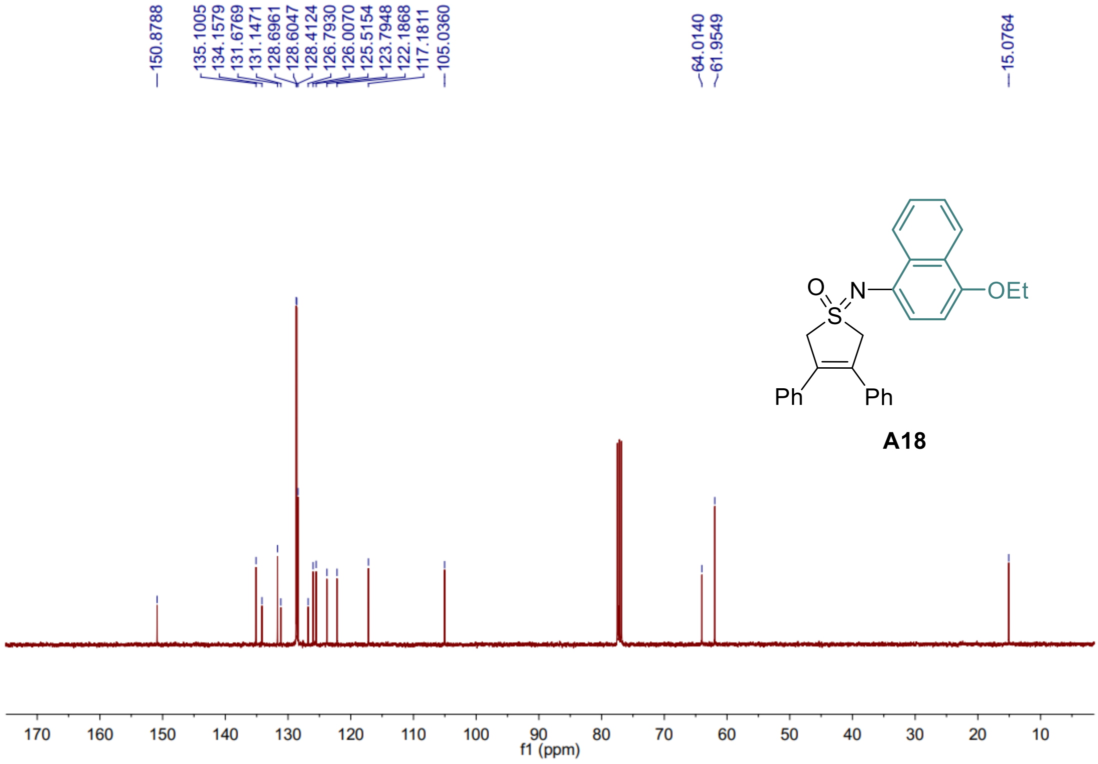

^{1}H-NMR and ^{13}C-NMR of **A18**

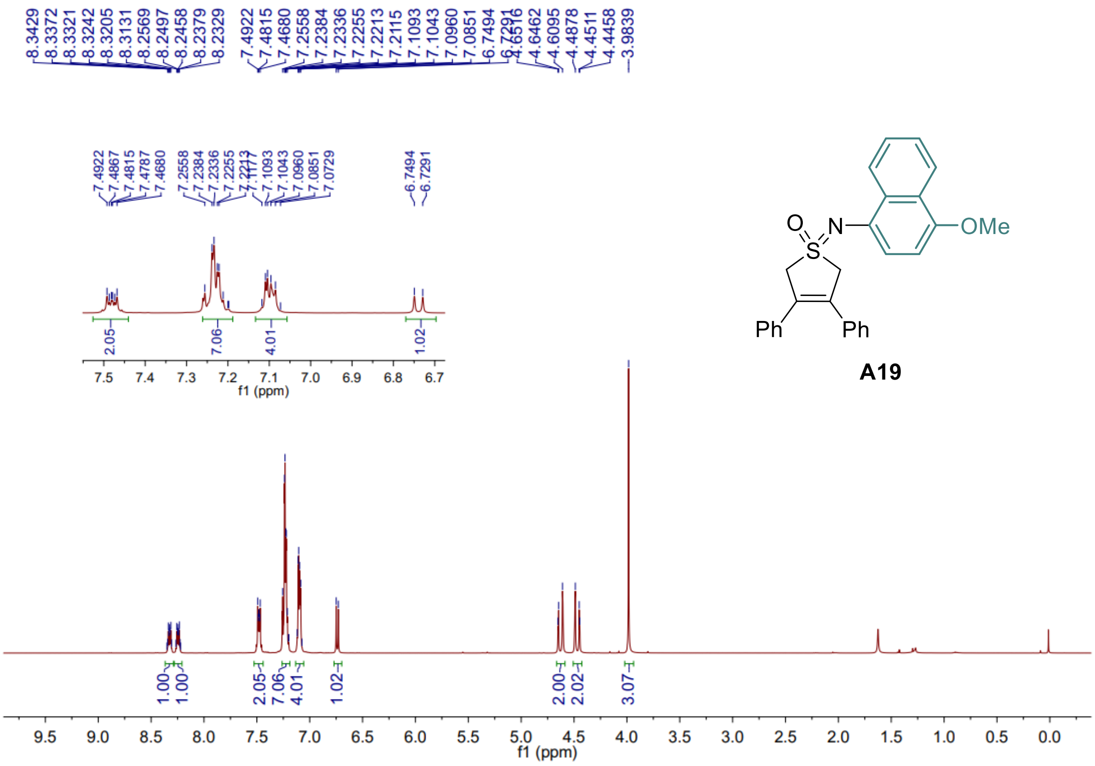

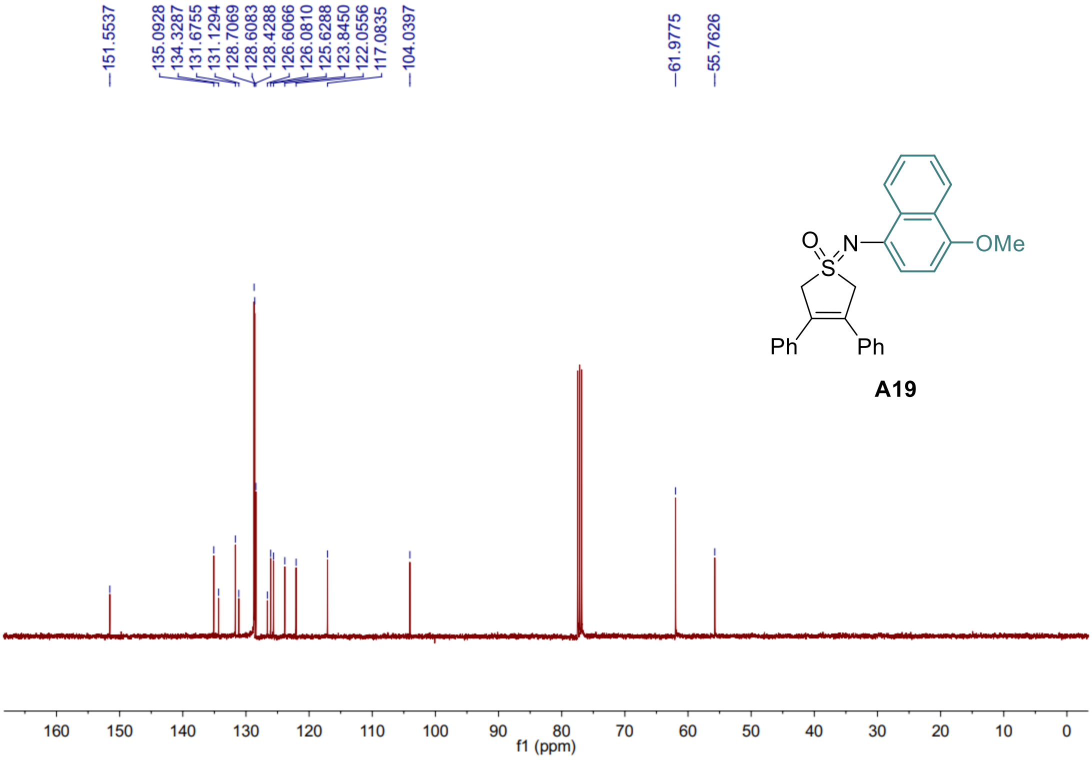

^{1}H-NMR and ^{13}C-NMR of **A19**

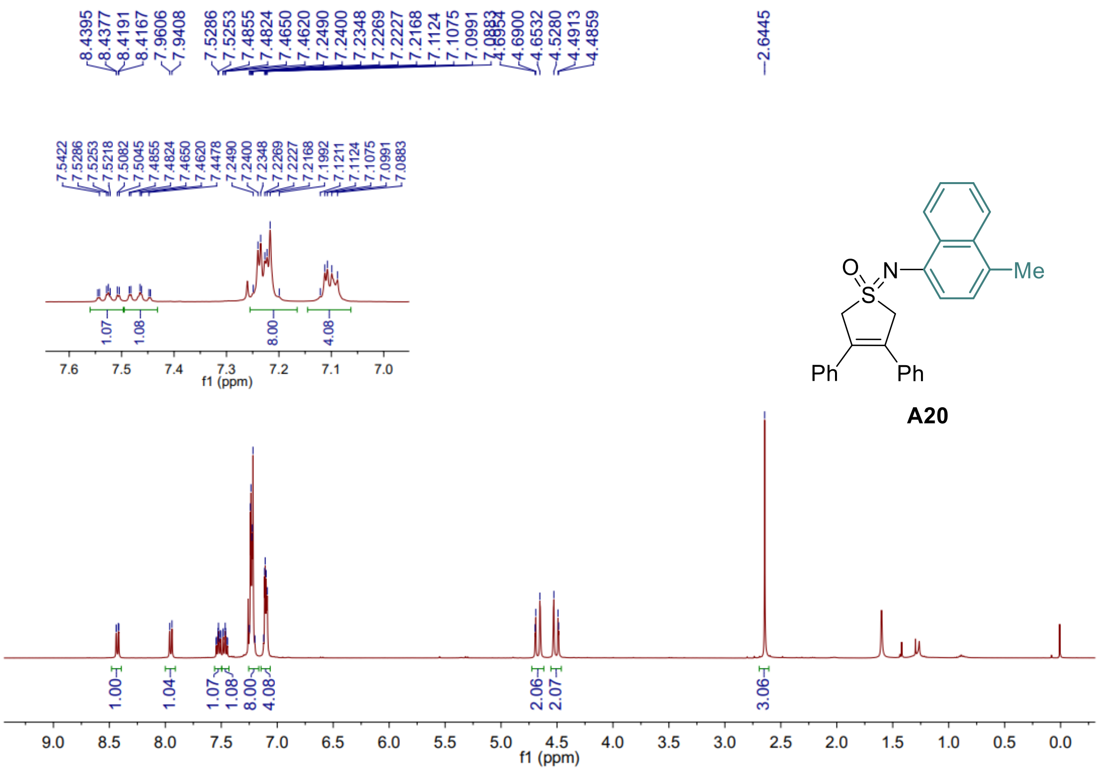

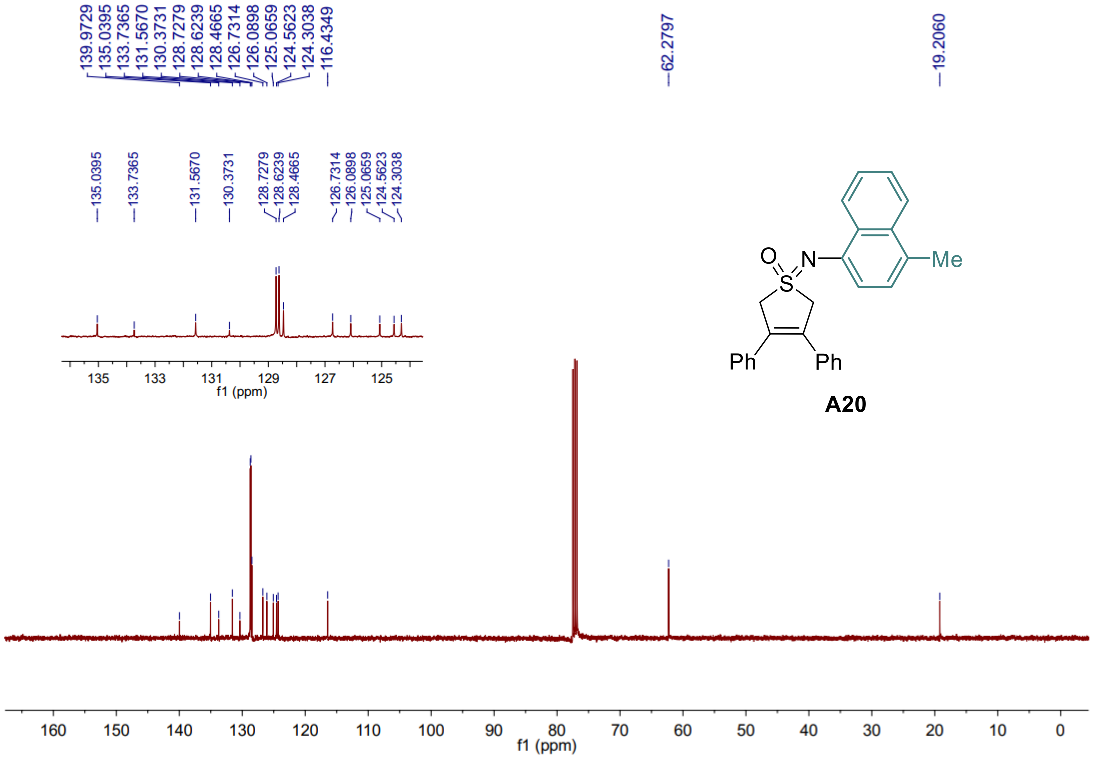

^{1}H-NMR and ^{13}C-NMR of **A20**

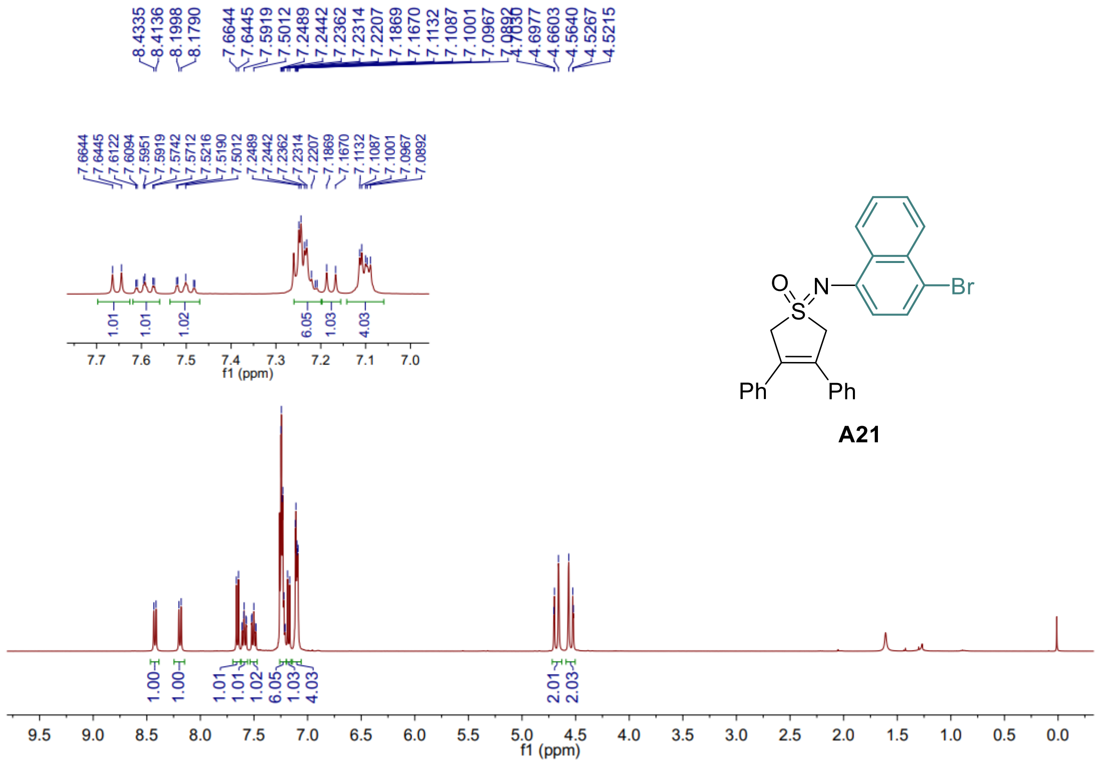

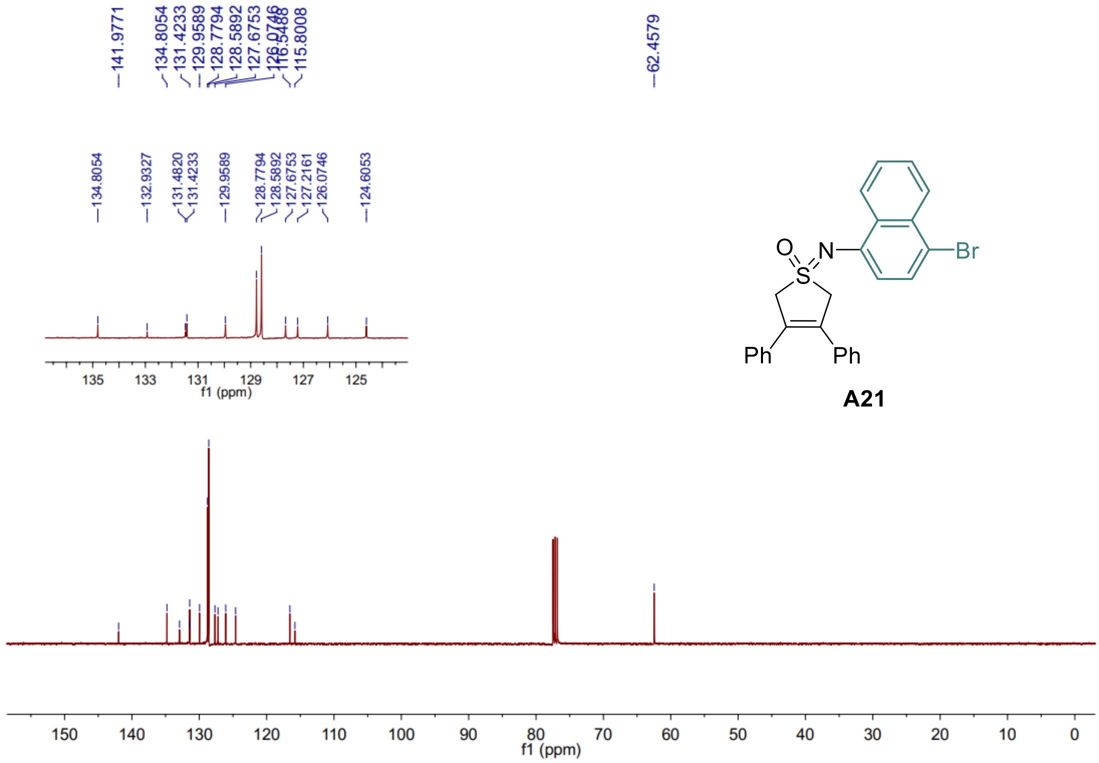

^{1}H-NMR and ^{13}C-NMR of **A21**

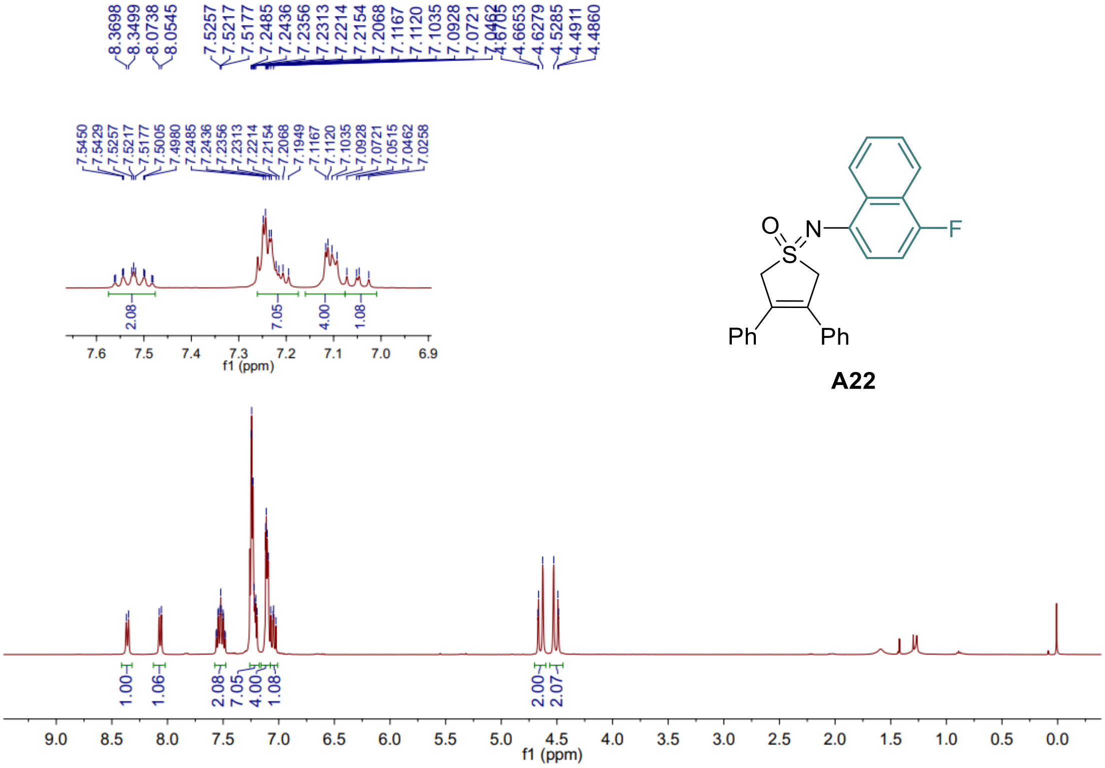
8.3698
8.3499
8.0738
8.0545
7.5257
7.5217
7.5177
7.2485
7.2436
7.2356
7.2313
7.2214
7.2154
7.2068
7.1167
7.1120
7.1035
7.0928
7.0721
7.0462
4.6705
4.6653
4.6279
4.5285
4.4911
4.4860
7.5450
7.5429
7.5257
7.5217
7.5177
7.5005
7.4980
7.2485
7.2436
7.2356
7.2313
7.2214
7.2154
7.2068
7.1949
7.1167
7.1120
7.1035
7.0928
7.0721
7.0515
7.0462
7.0258
2.08
7.05
4.00
1.08
f1 (ppm)
1.00
1.06
2.08
7.05
4.00
1.08
2.00
2.07
f1 (ppm)
O
N
S
F
Ph
Ph

A22

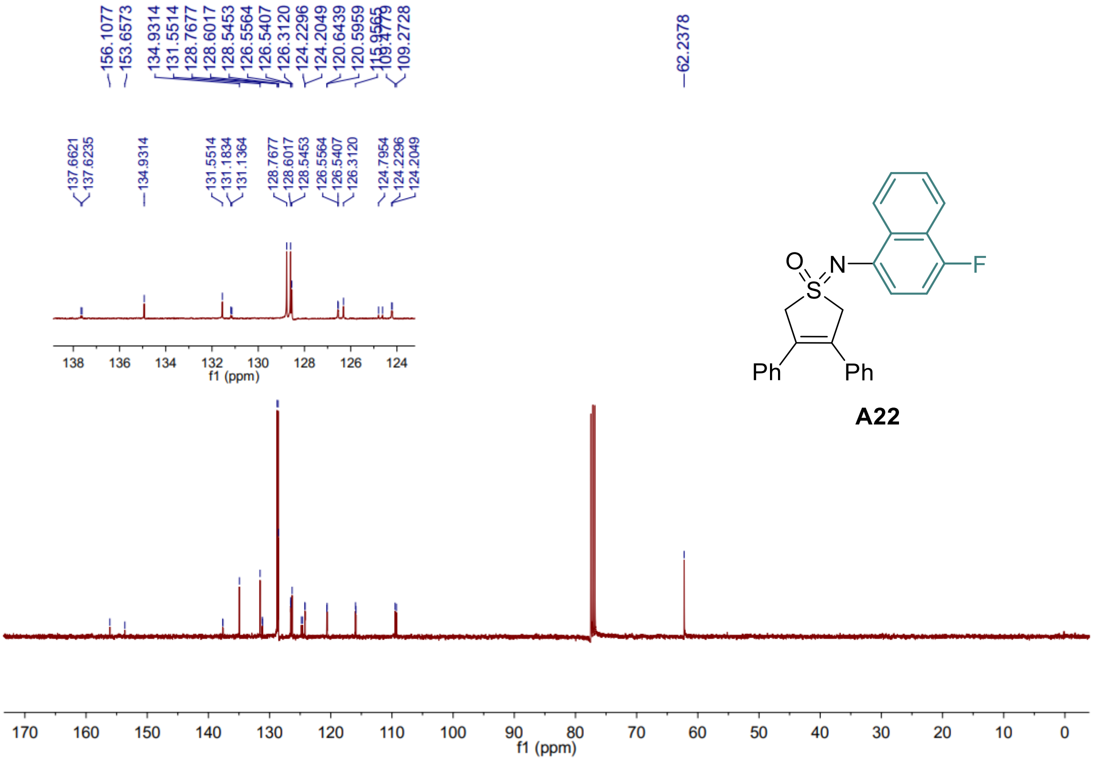
156.1077
153.6573
134.9314
131.5514
128.7677
128.6017
128.5453
126.5564
126.5407
126.3120
124.2296
124.2049
120.6439
120.5959
115.9565
109.4779
109.2728
62.2378
137.6621
137.6235
134.9314
131.5514
131.1834
131.1364
128.7677
128.6017
128.5453
126.5564
126.5407
126.3120
124.7954
124.2296
124.2049
f1 (ppm)
f1 (ppm)
O
N
S
F
Ph
Ph

A22

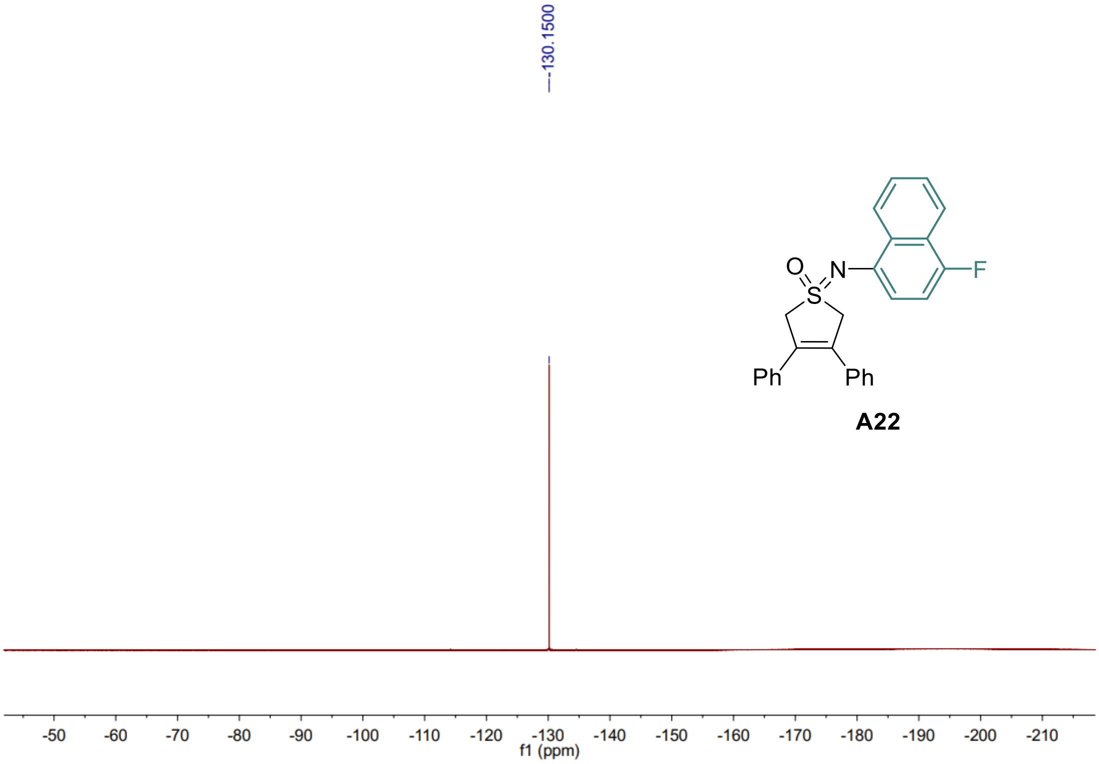

^{1}H-NMR and ^{13}C-NMR and ^{19}F-NMR of **A22**

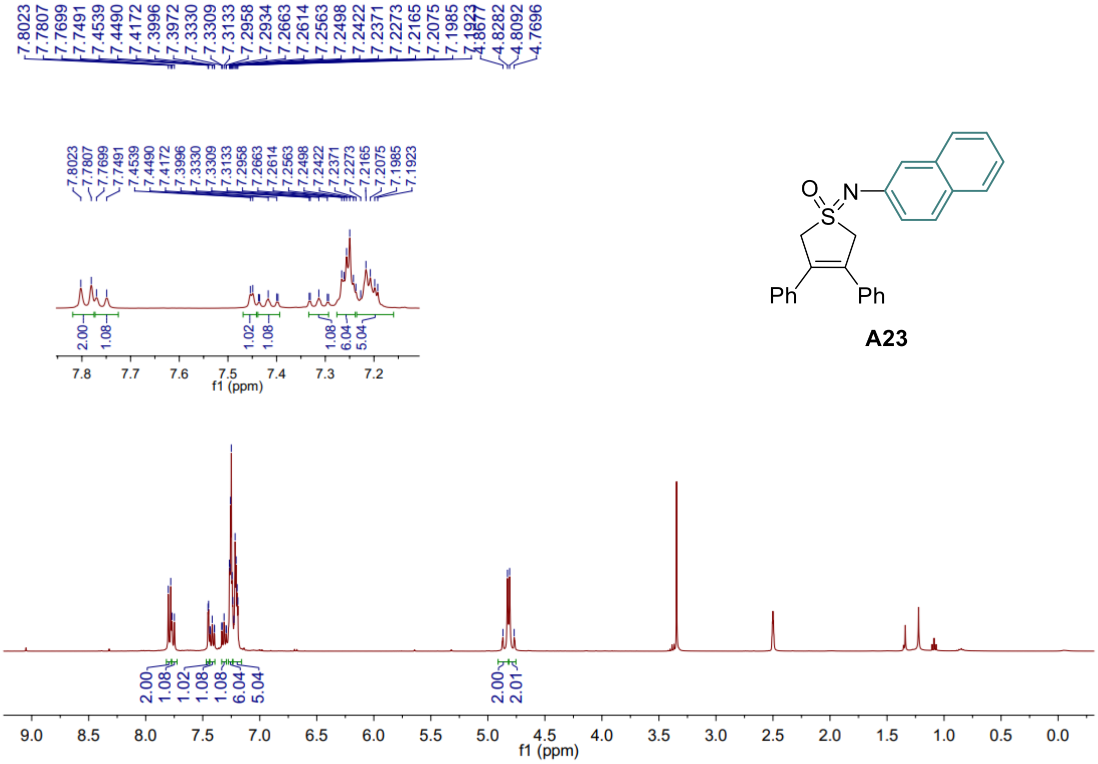

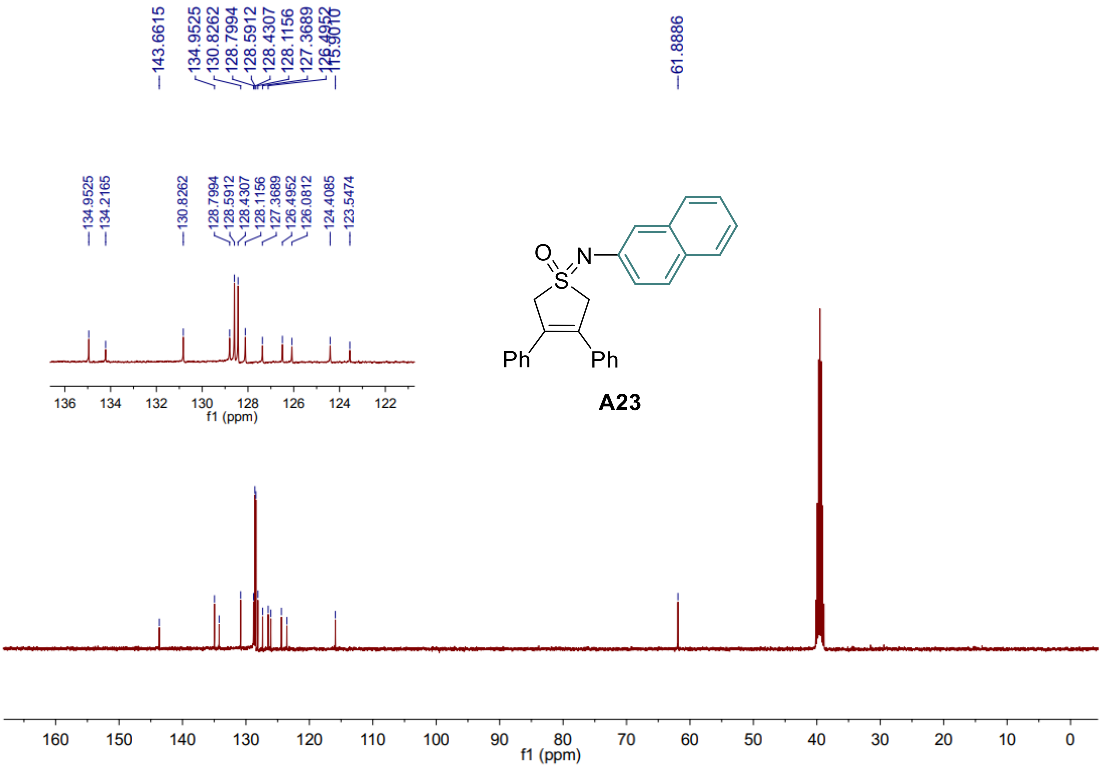

^{1}H-NMR and ^{13}C-NMR of **A23**

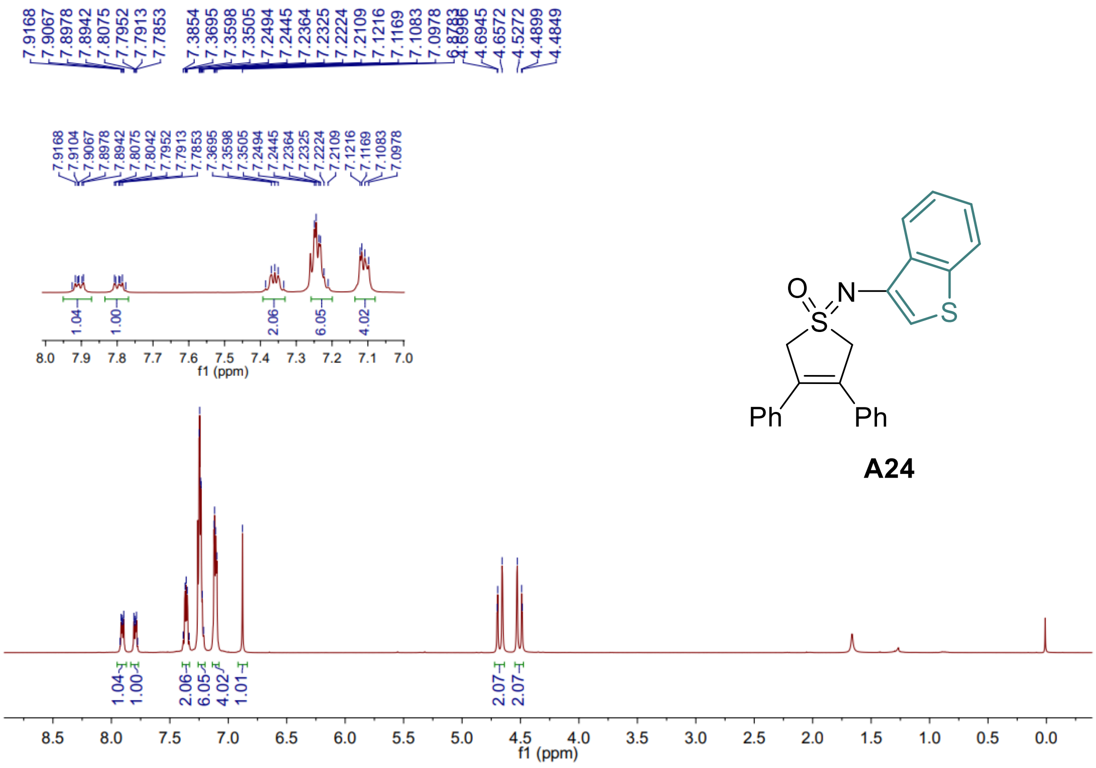

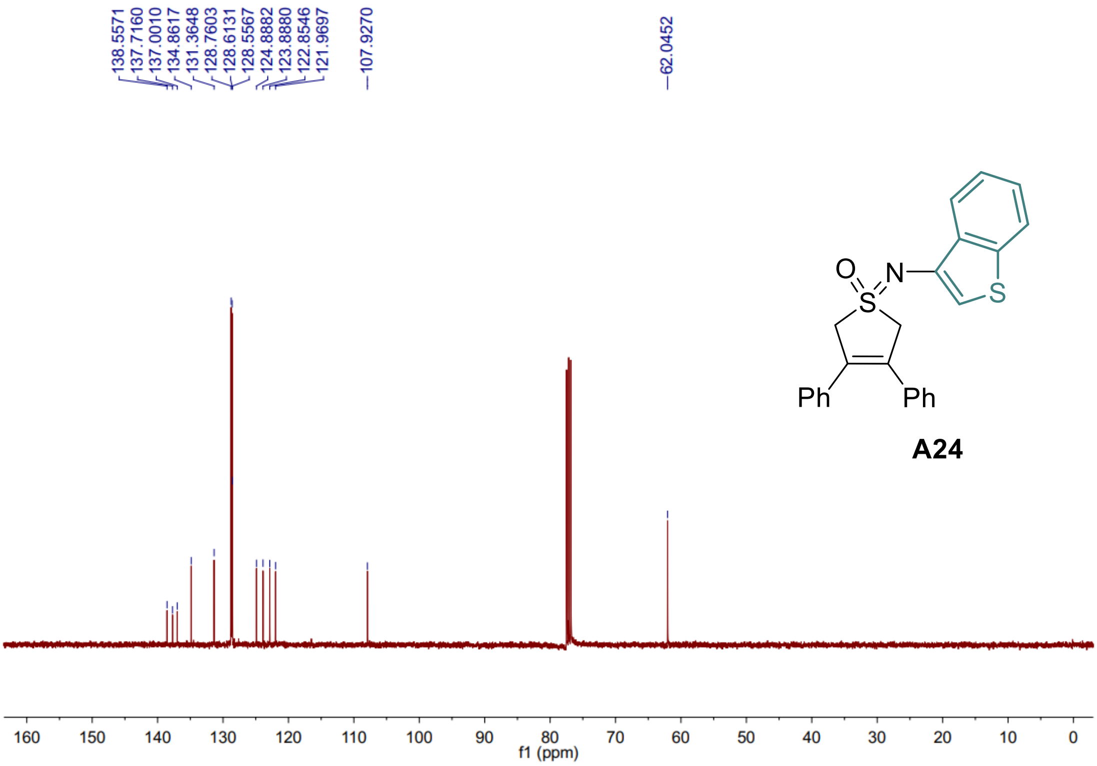

^{1}H-NMR and ^{13}C-NMR of **A24**

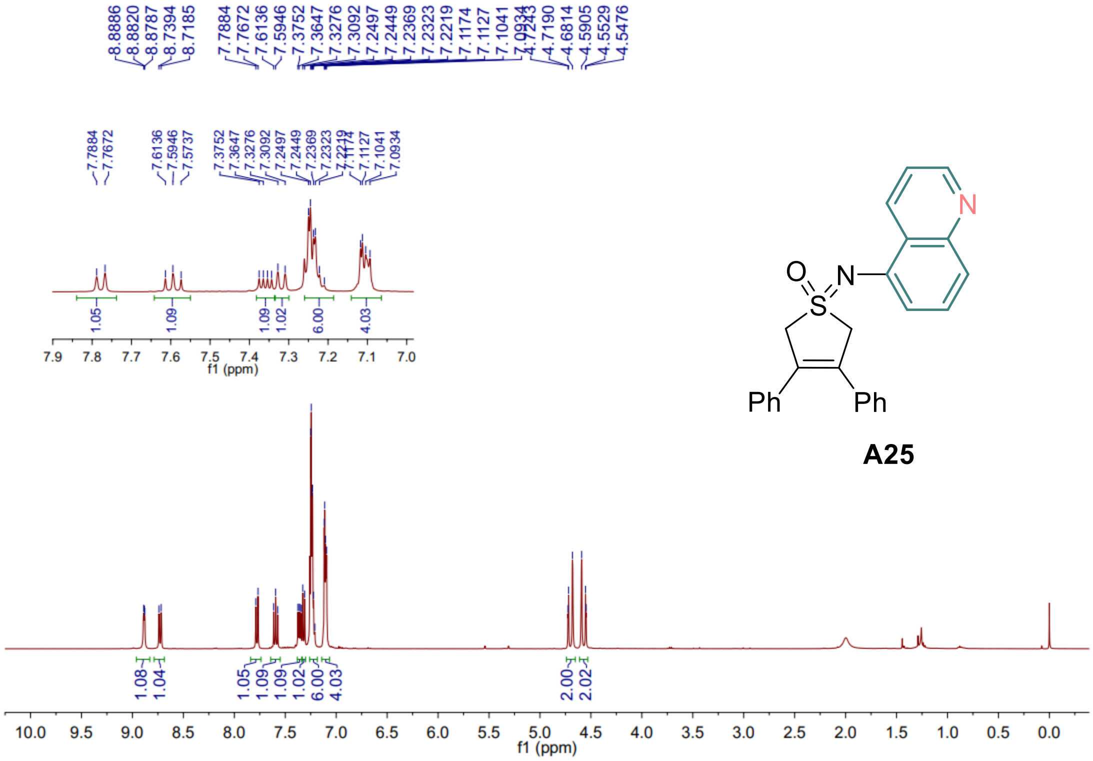

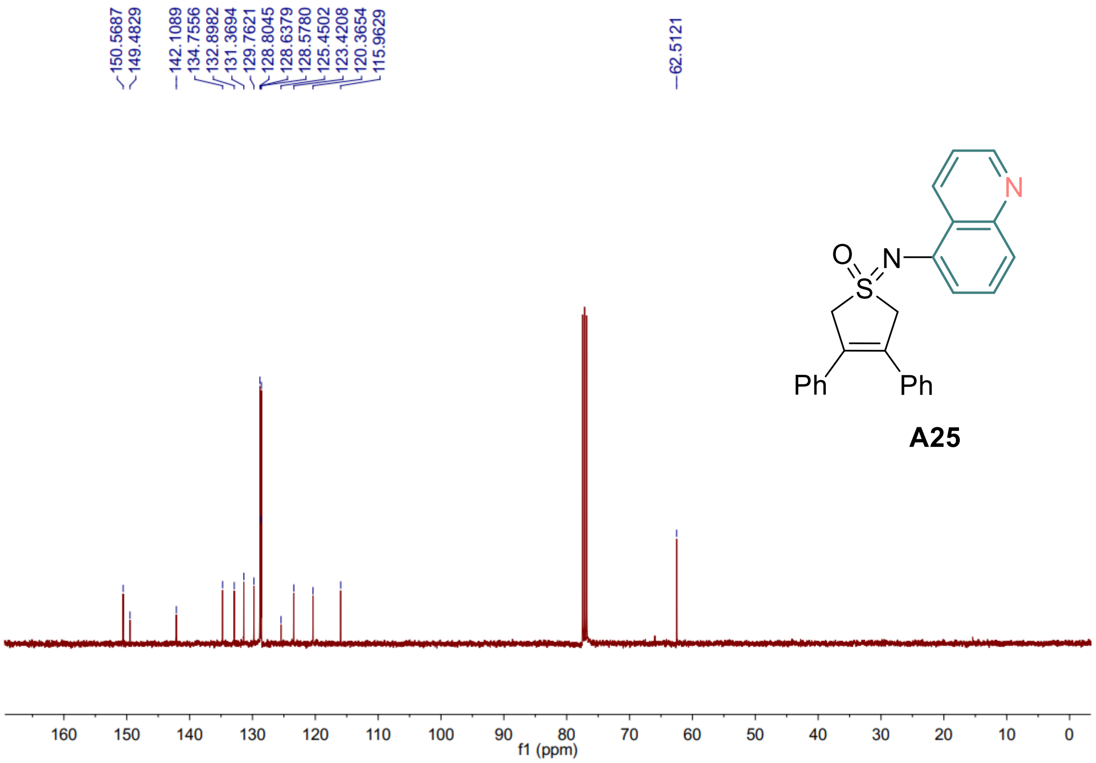

^{1}H-NMR and ^{13}C-NMR of **A25**

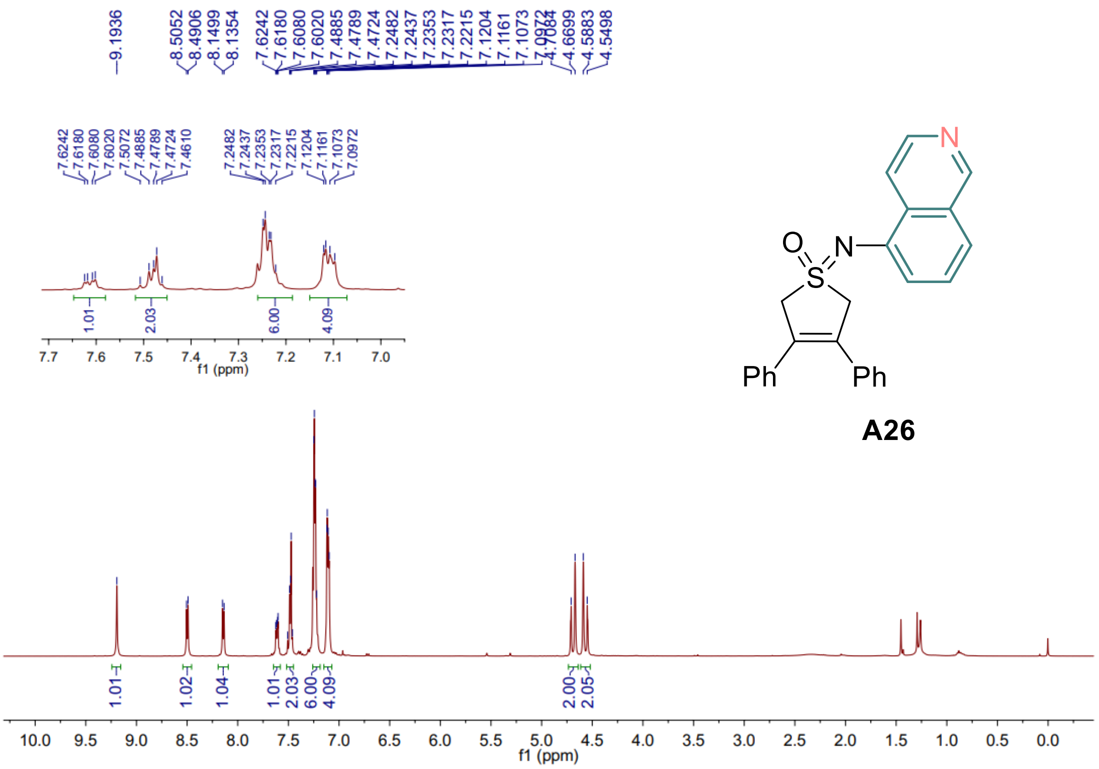

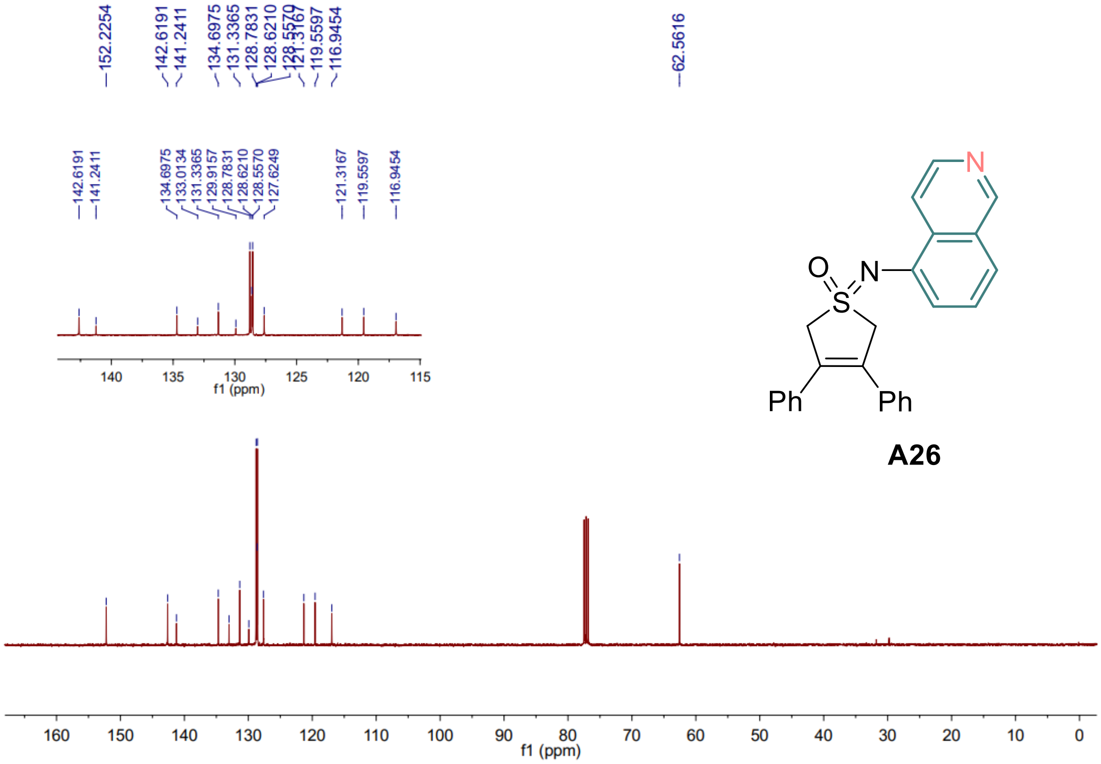

^{1}H-NMR and ^{13}C-NMR of **A26**

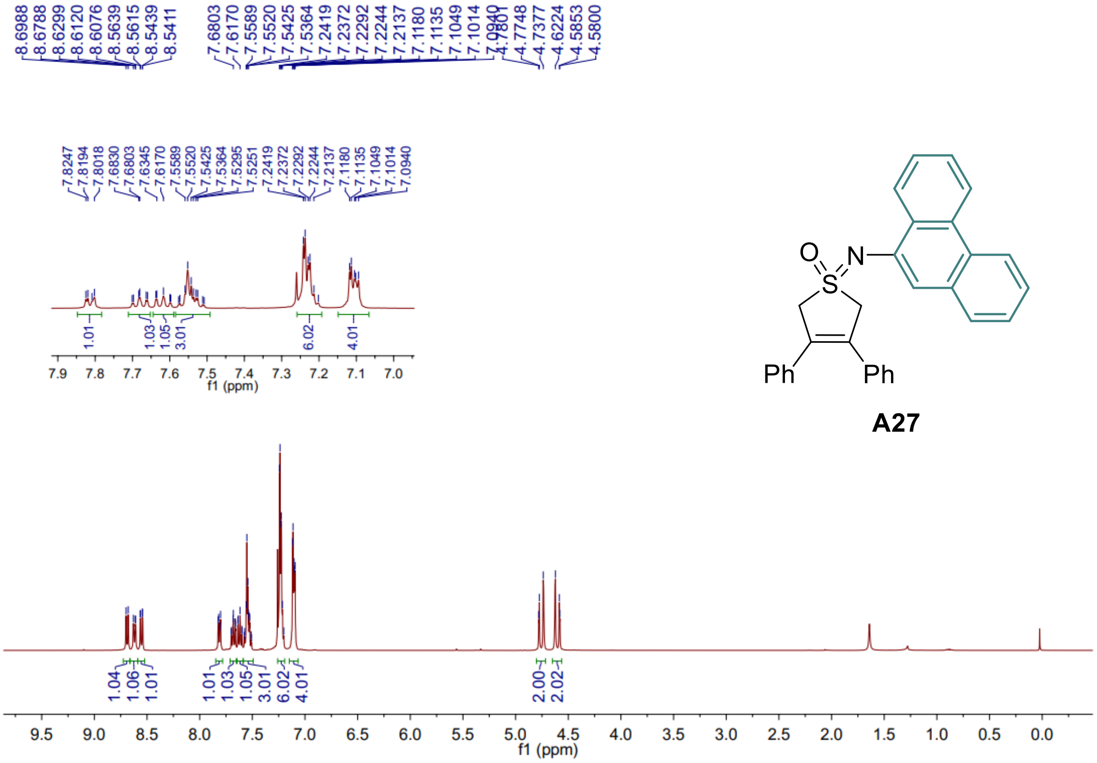

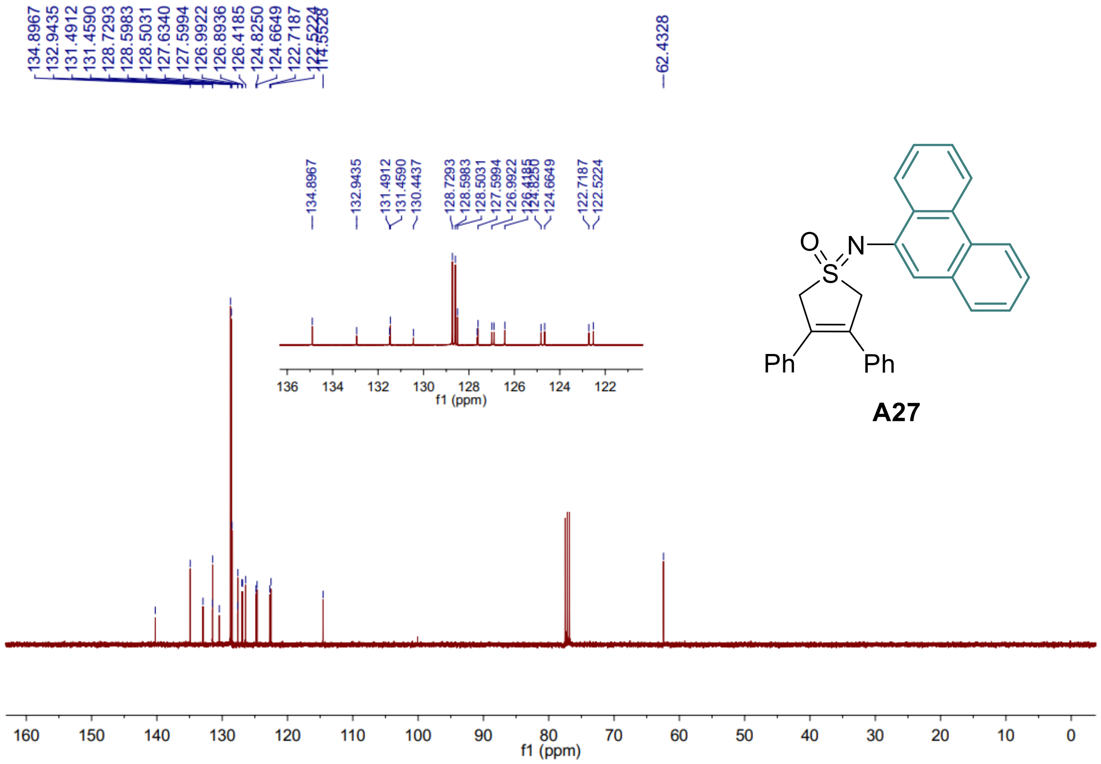

^{1}H-NMR and ^{13}C-NMR of **A27**

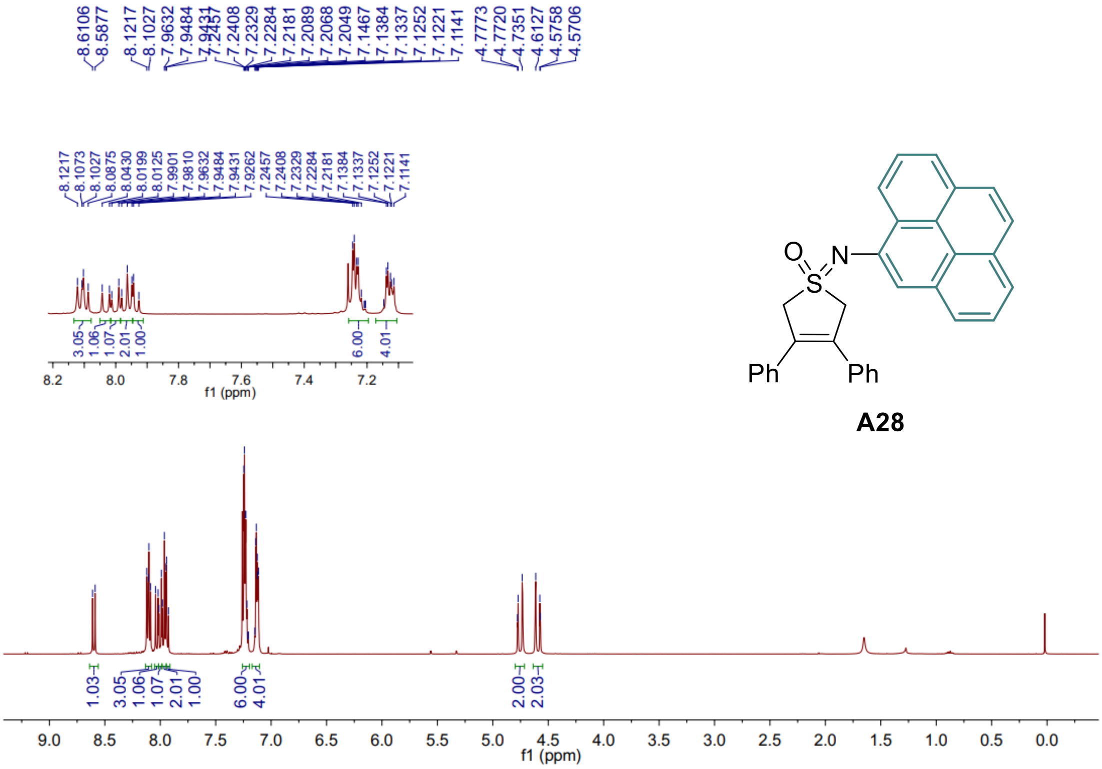

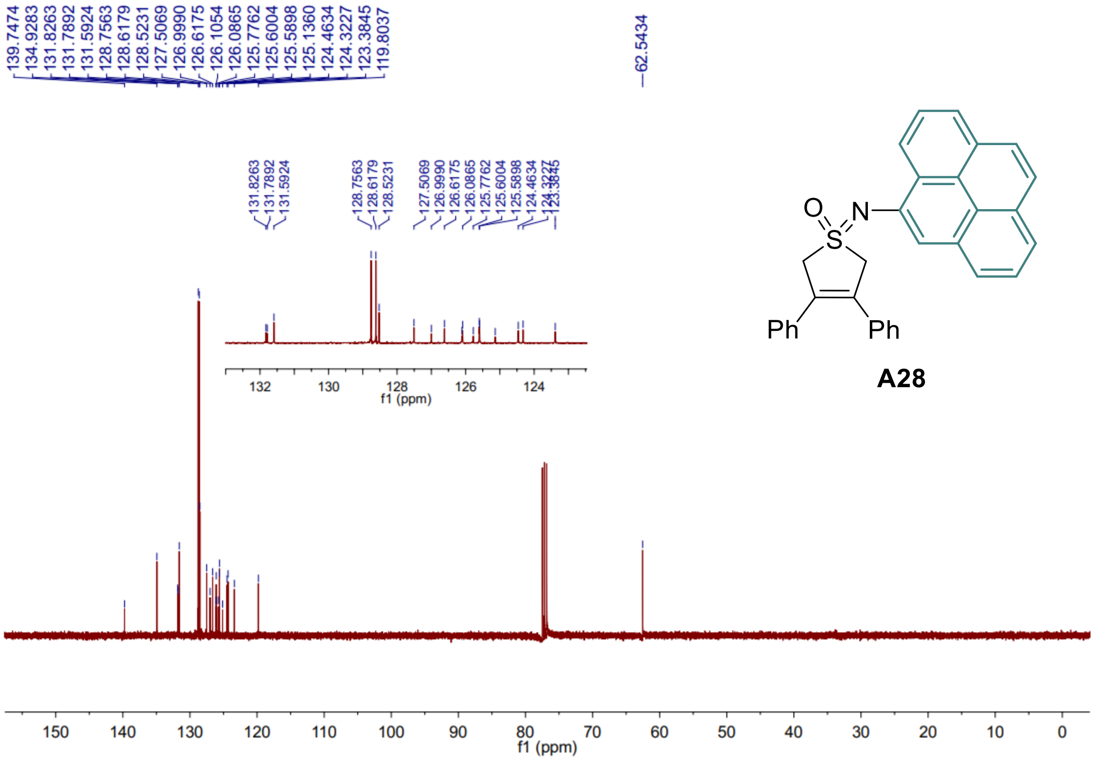

^{1}H-NMR and ^{13}C-NMR of **A28**

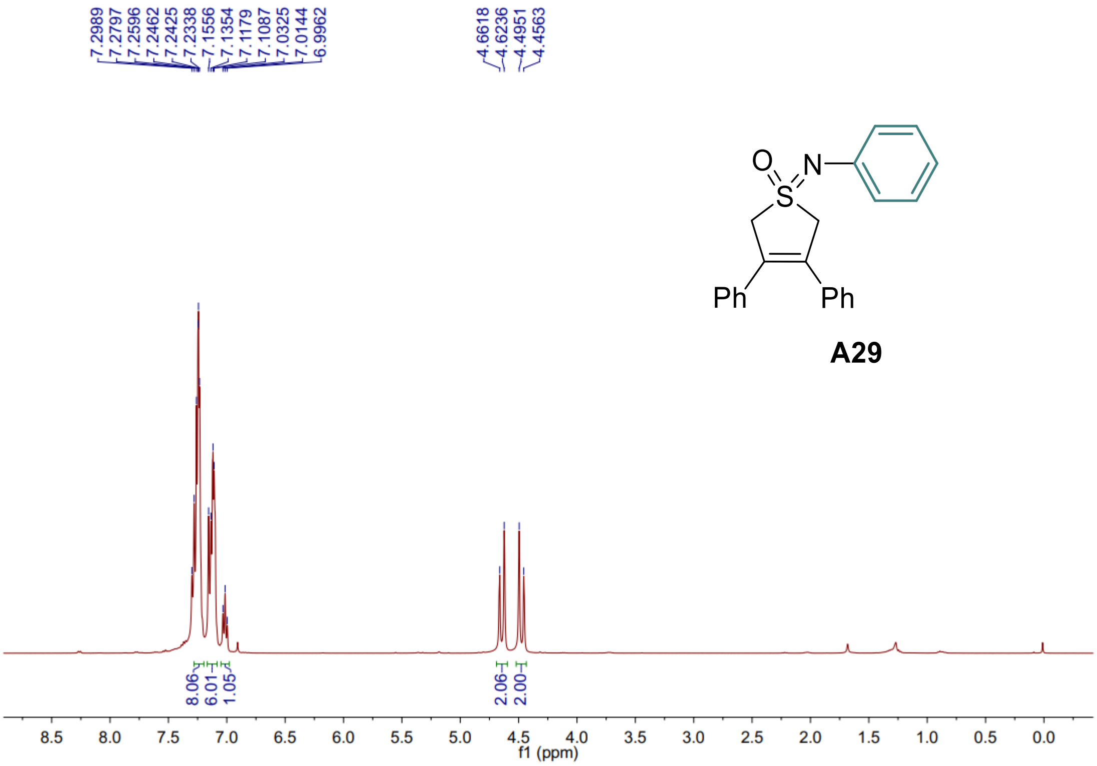

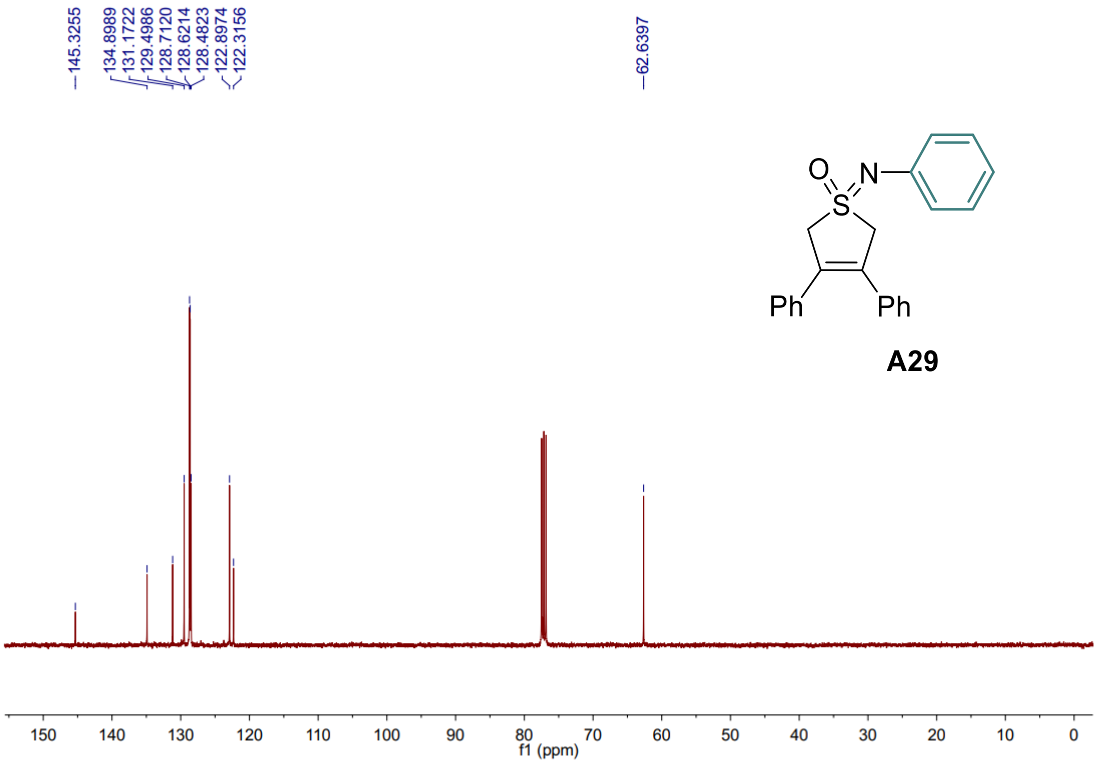

^{1}H-NMR and ^{13}C-NMR of **A29**

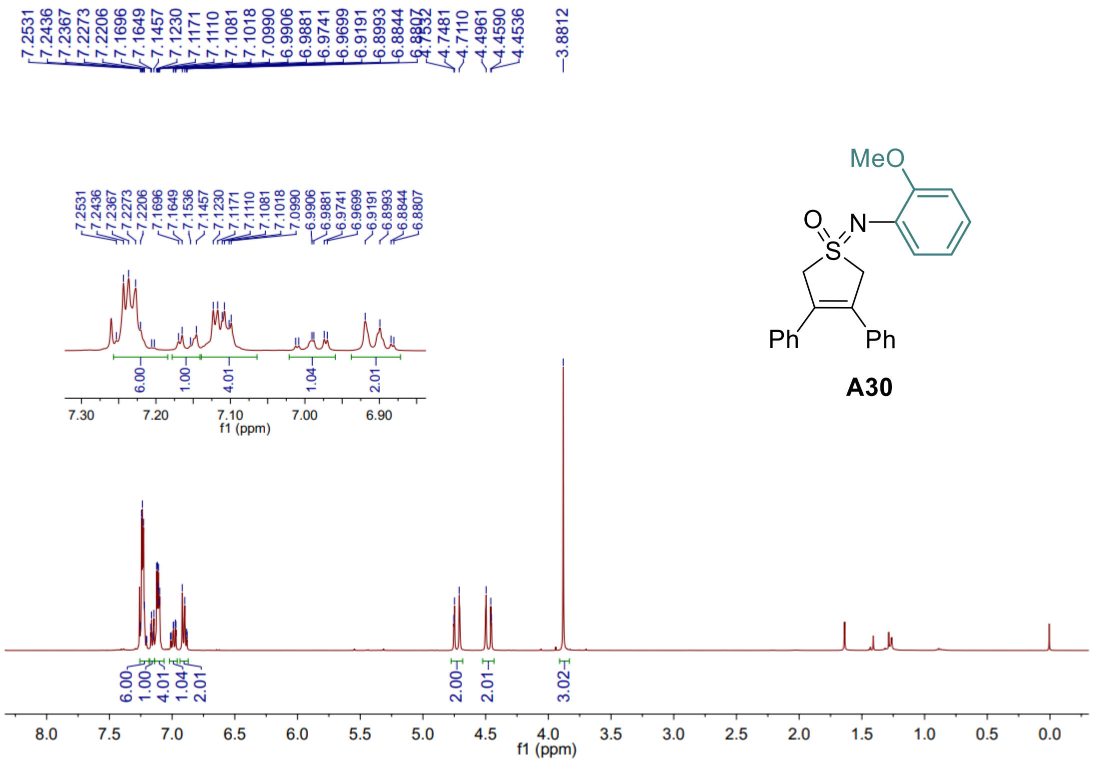

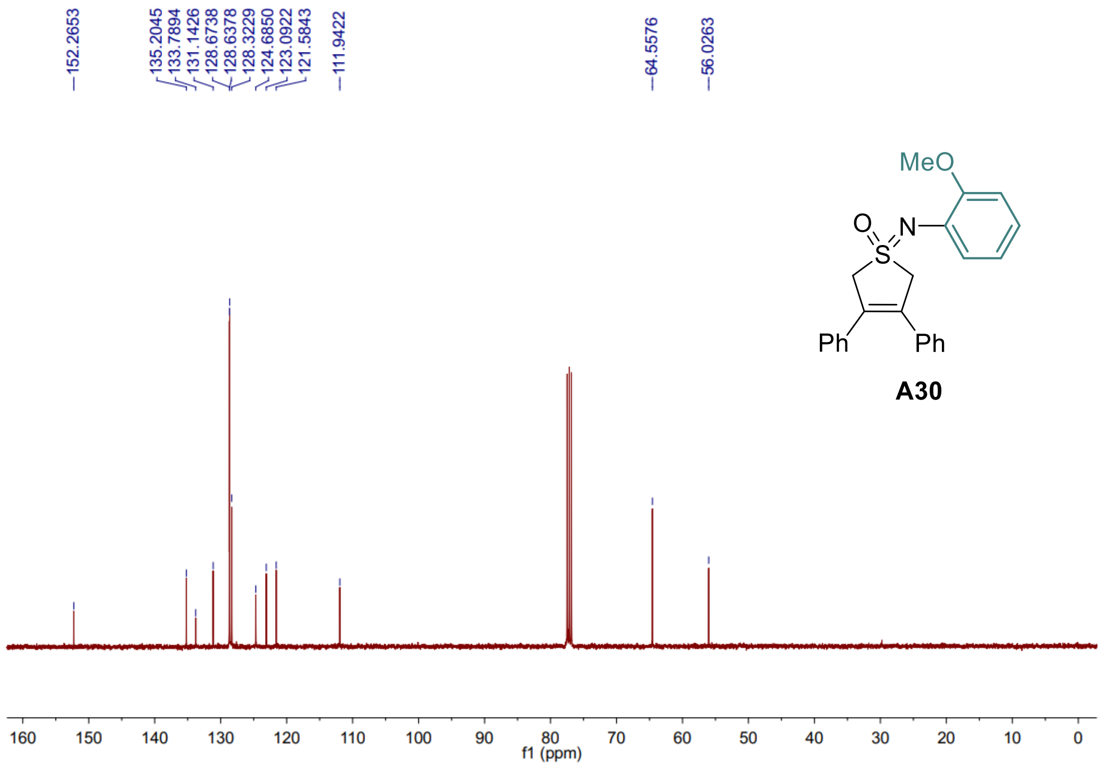

^{1}H-NMR and ^{13}C-NMR of **A30**

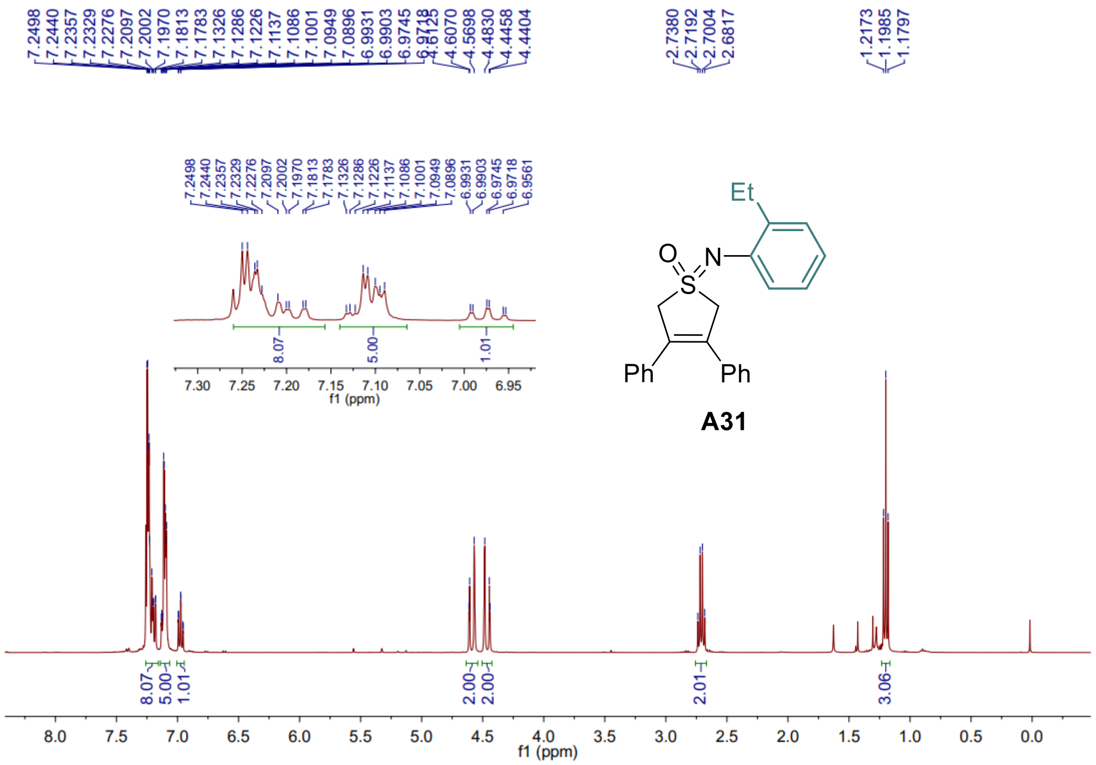

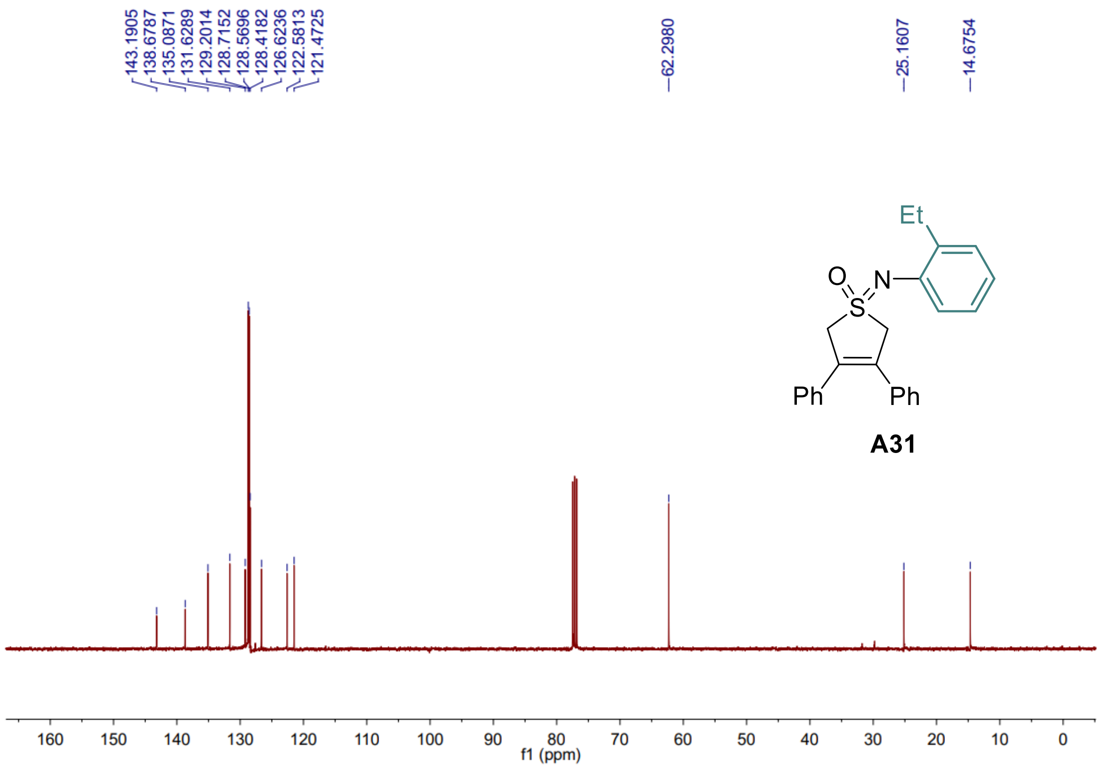

^{1}H-NMR and ^{13}C-NMR of **A31**

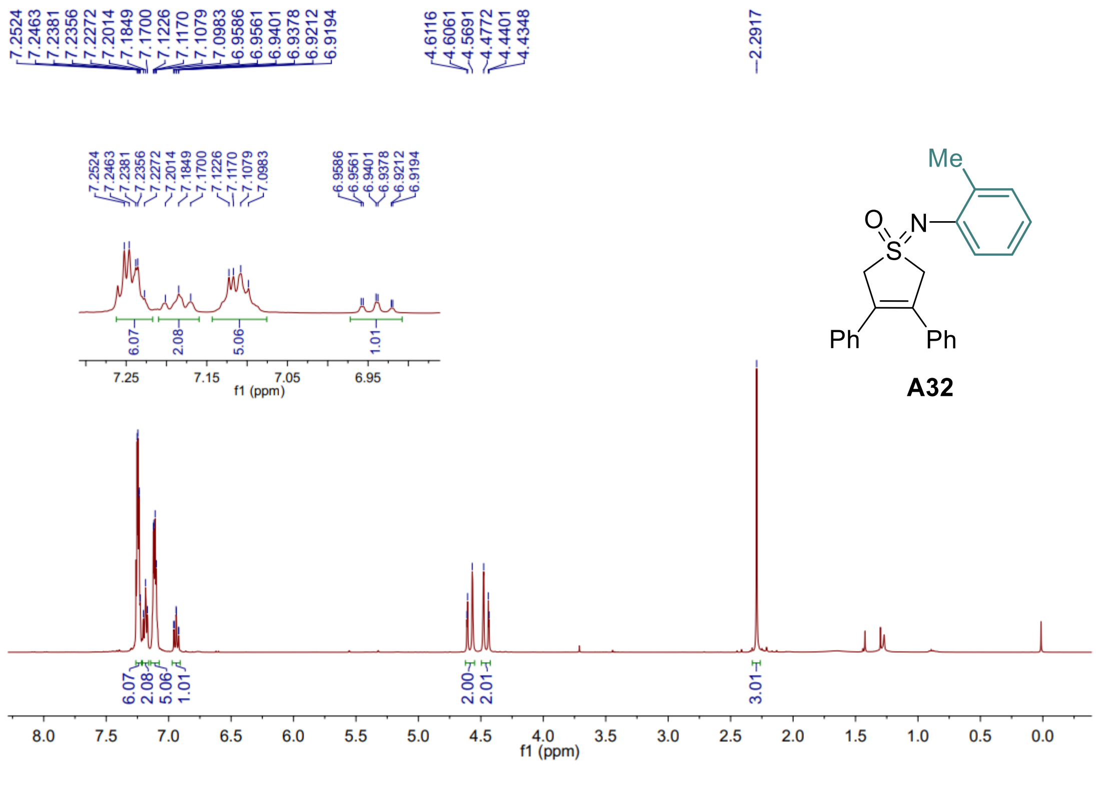

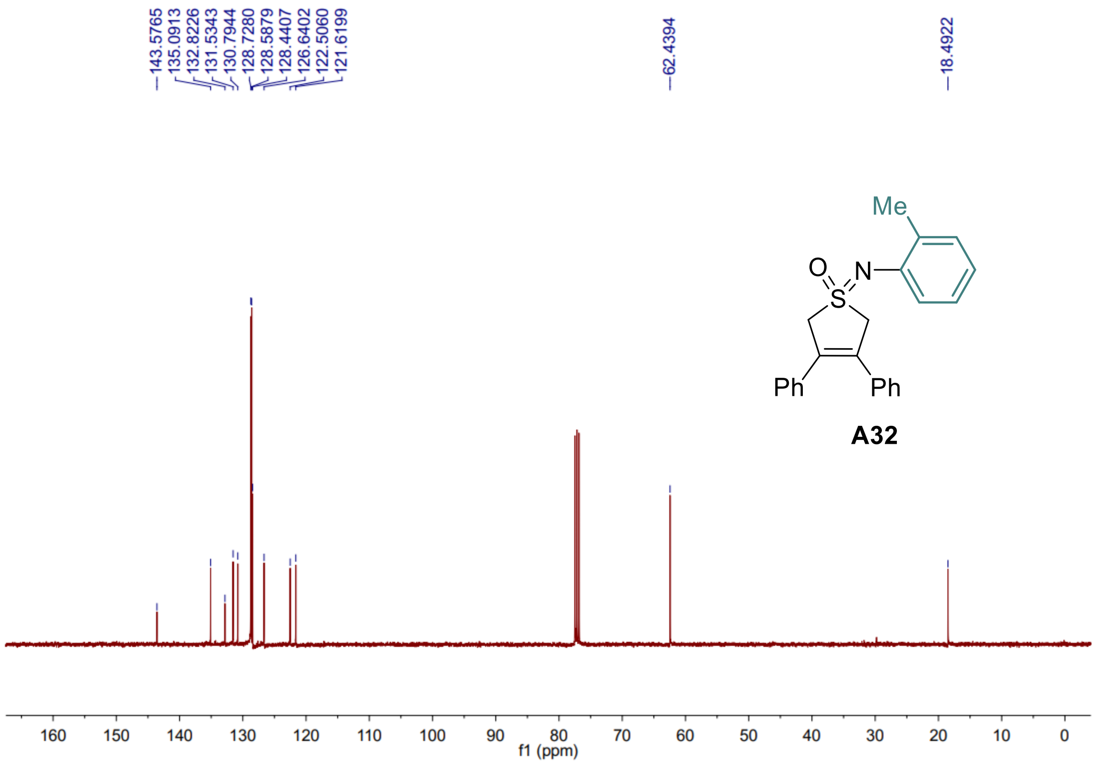

^{1}H-NMR and ^{13}C-NMR of **A32**

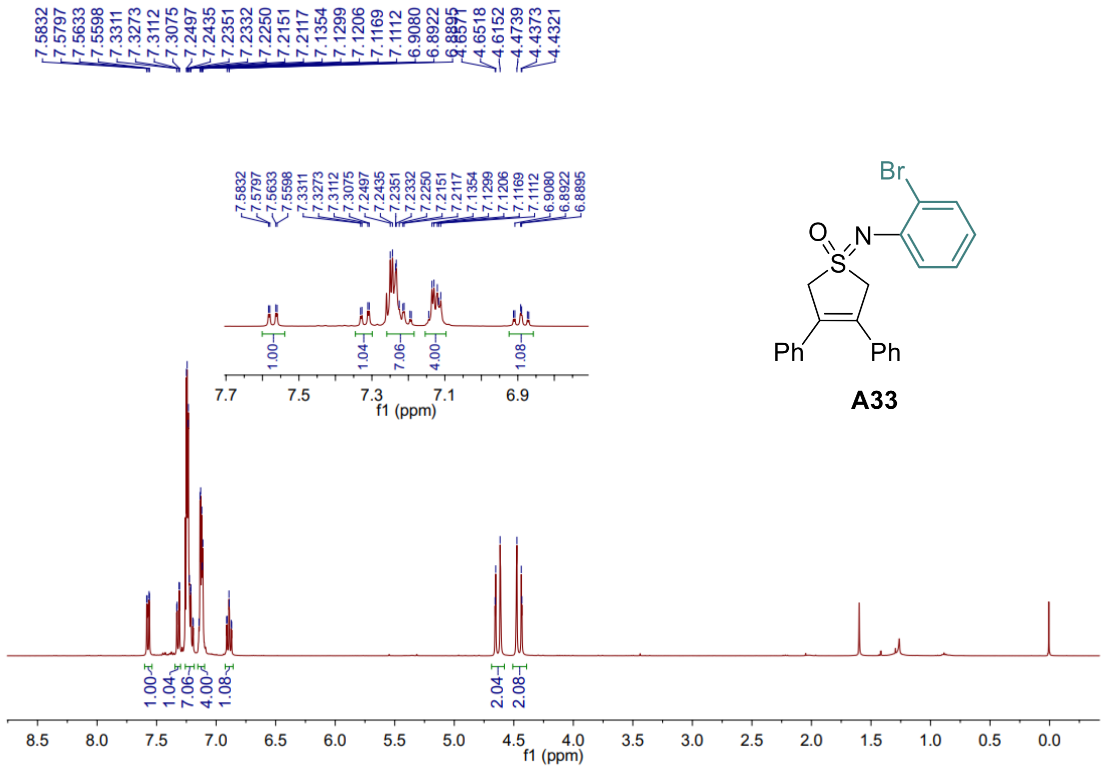

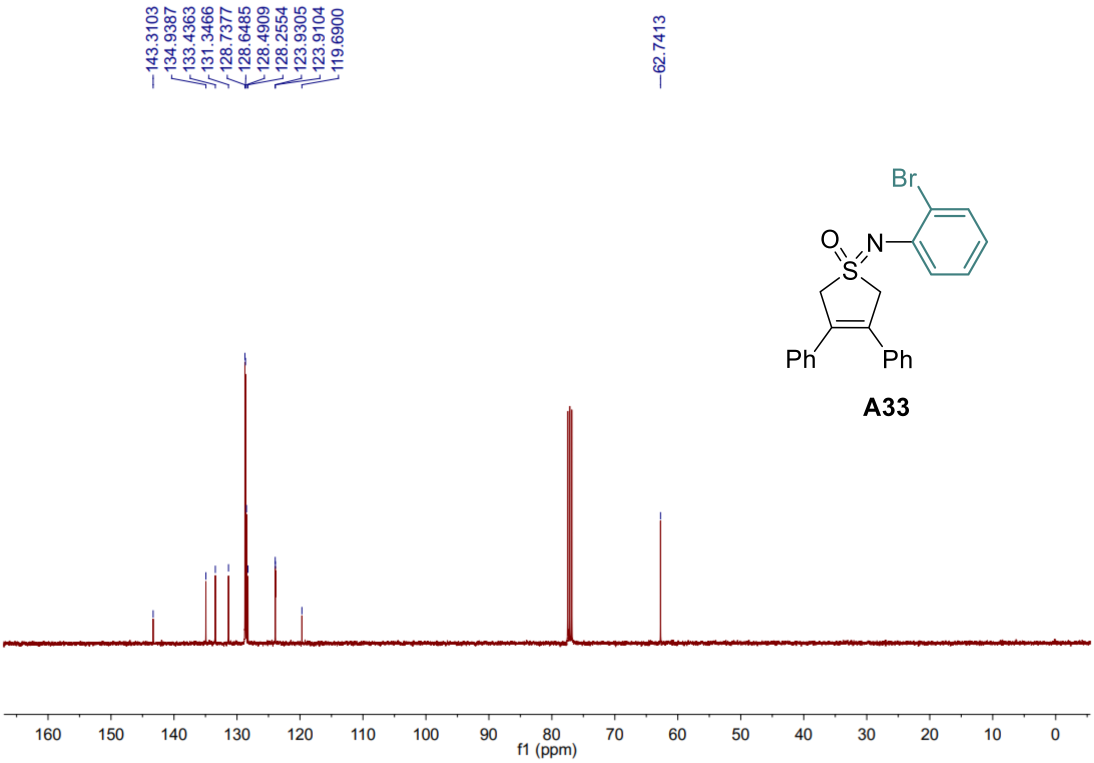

^{1}H-NMR and ^{13}C-NMR of **A33**

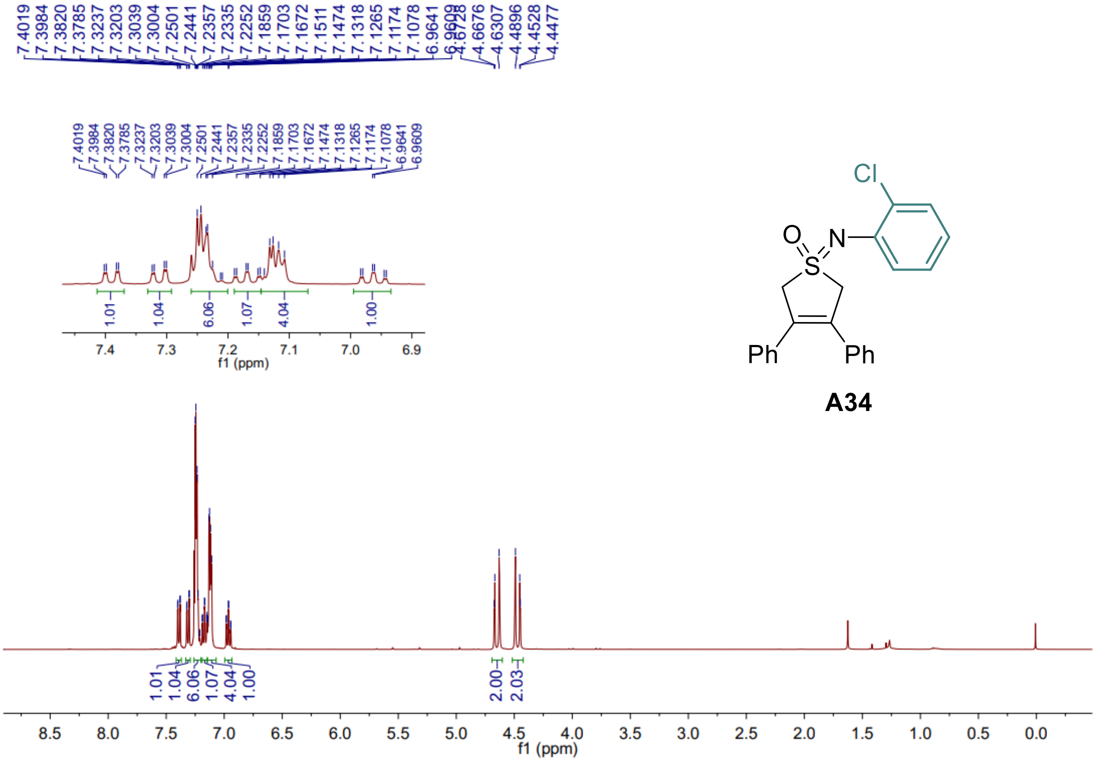

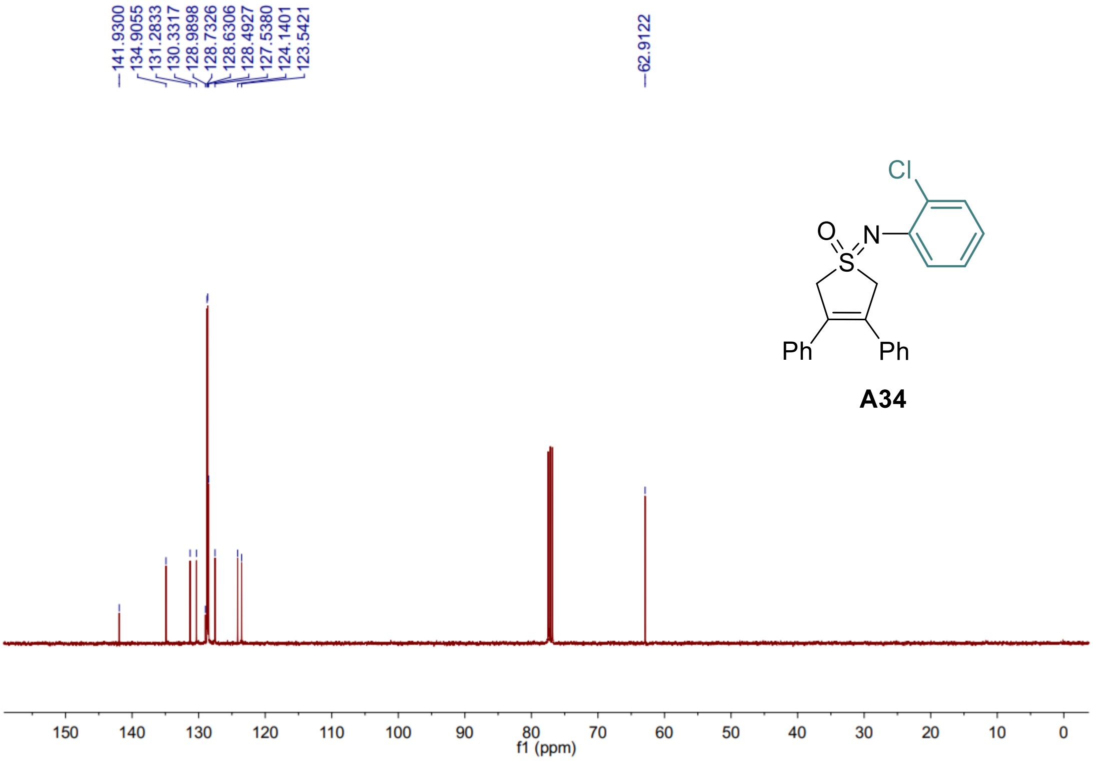

A34

^{1}H-NMR and ^{13}C-NMR of **A34**

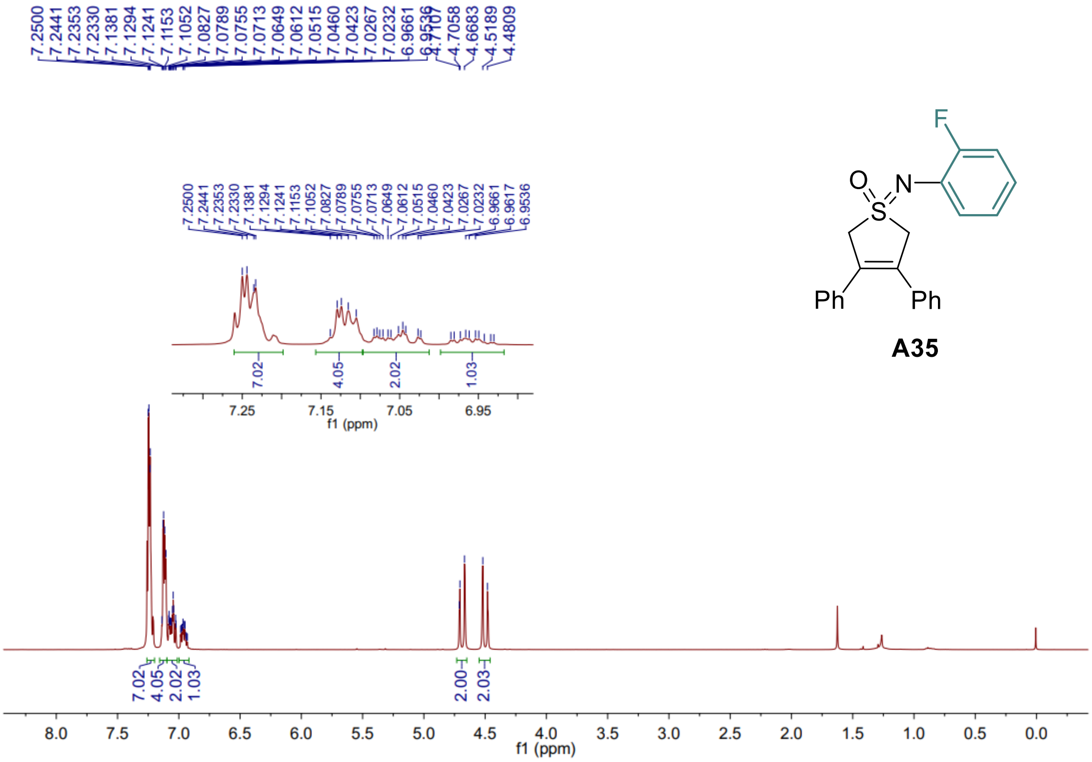

A35

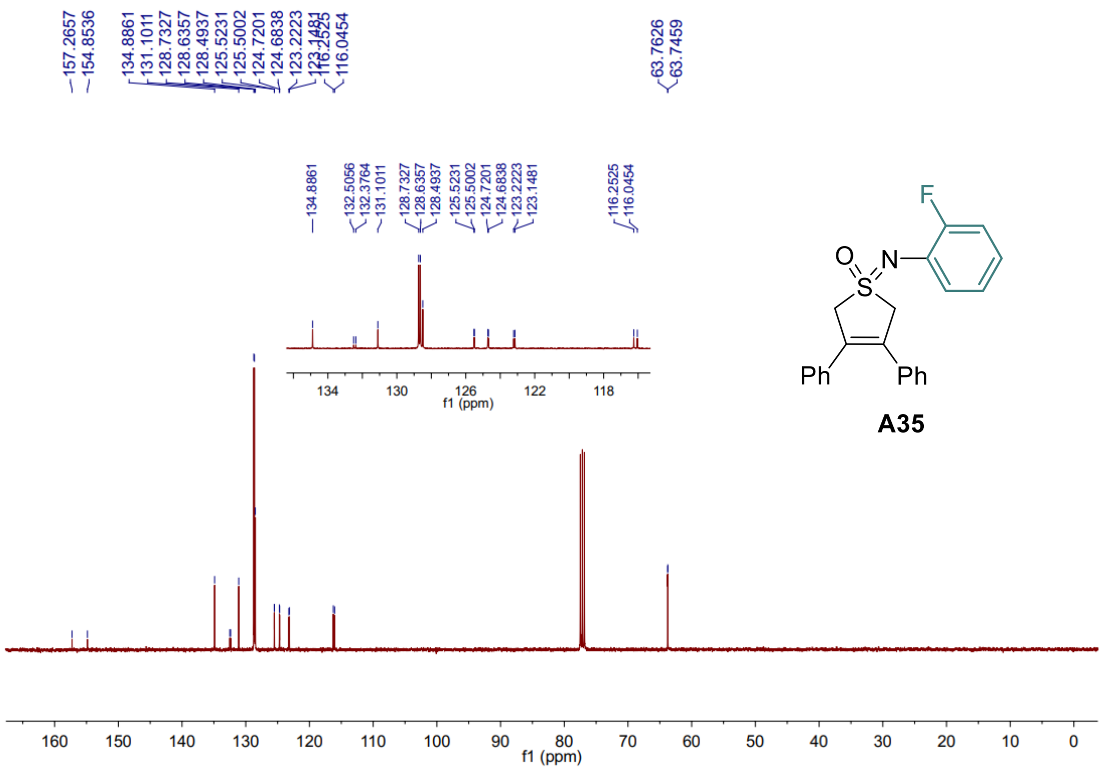

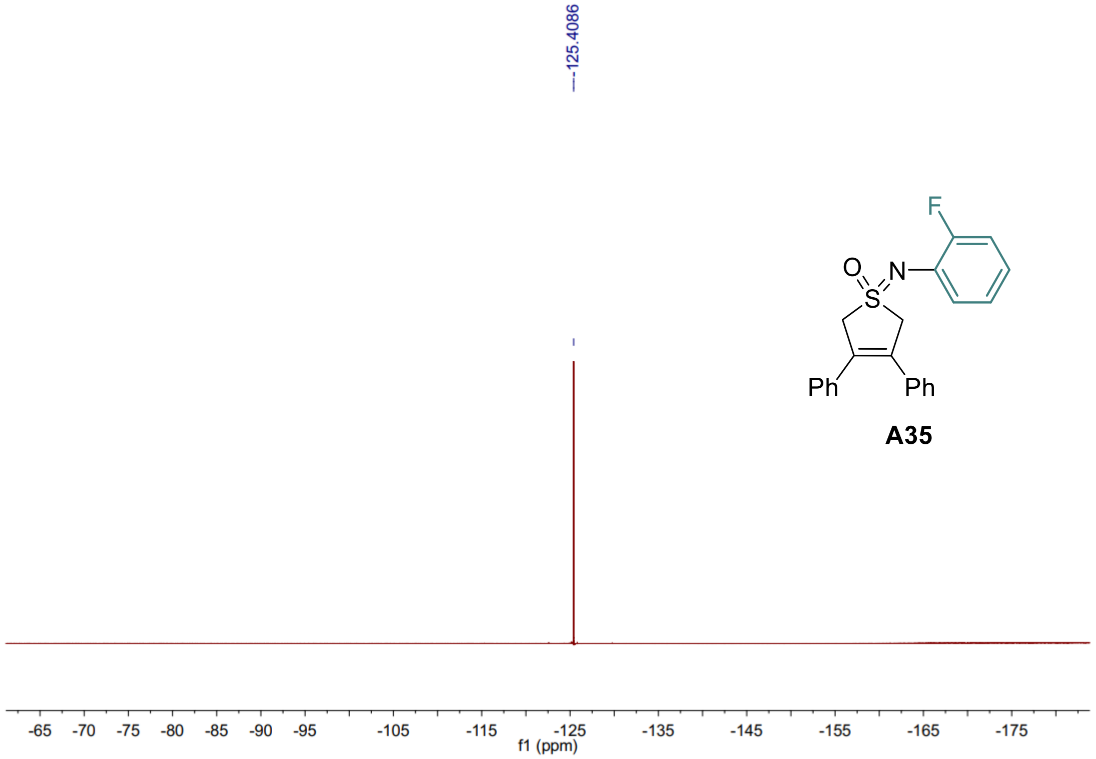

^{1}H-NMR and ^{13}C-NMR and ^{19}F-NMR of **A35**

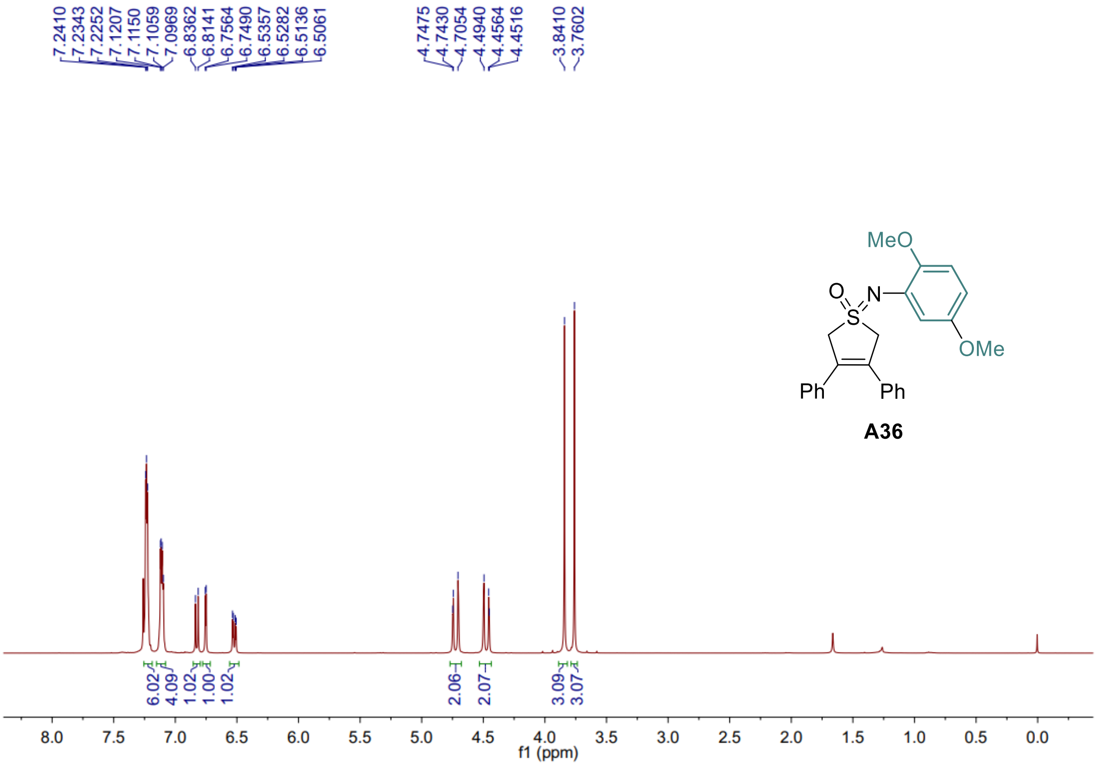

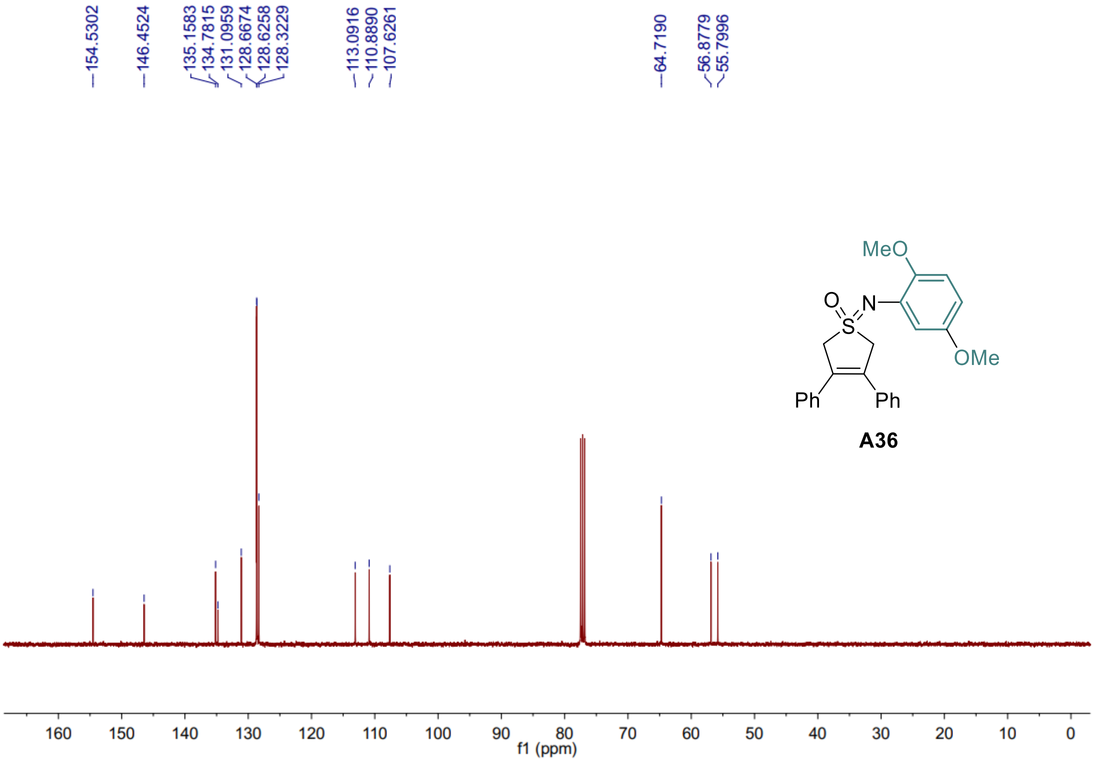

^{1}H-NMR and ^{13}C-NMR of **A36**

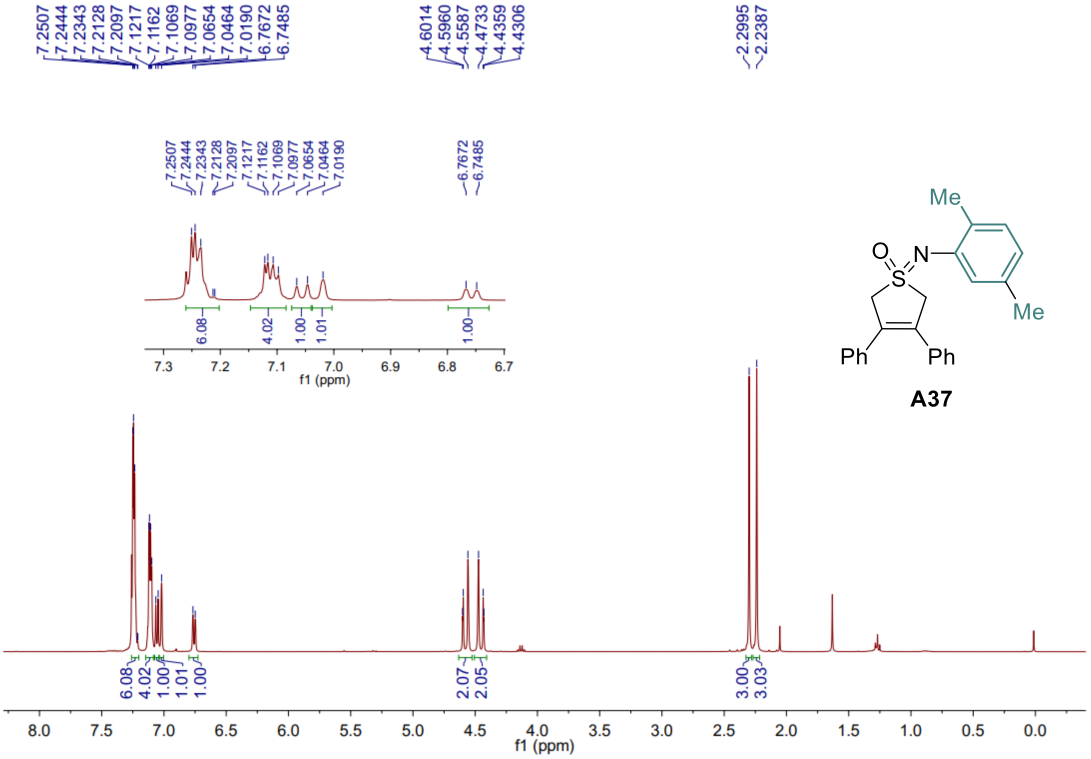

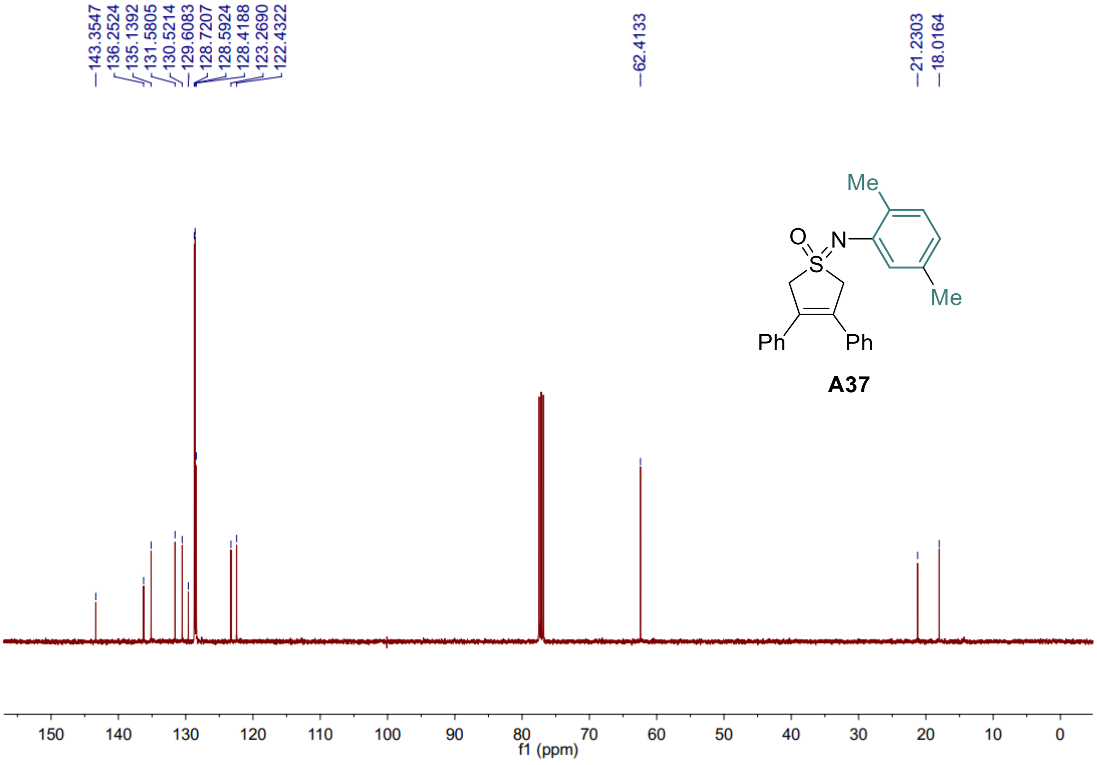

^{1}H-NMR and ^{13}C-NMR of **A37**

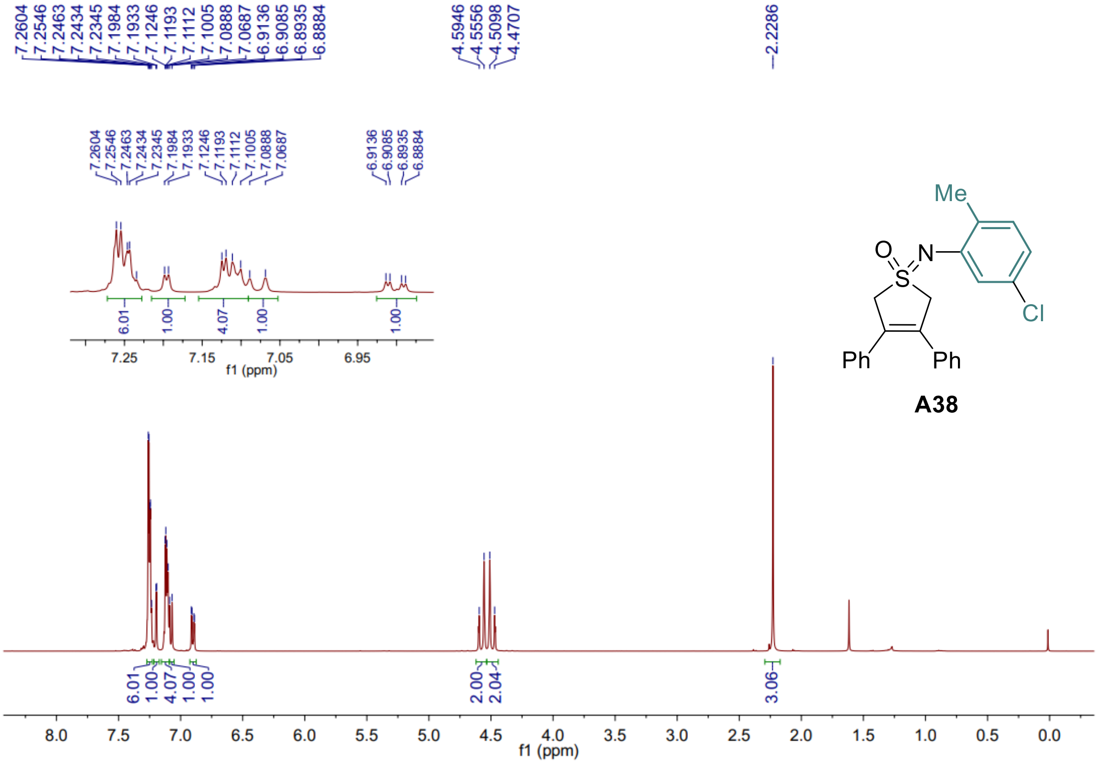

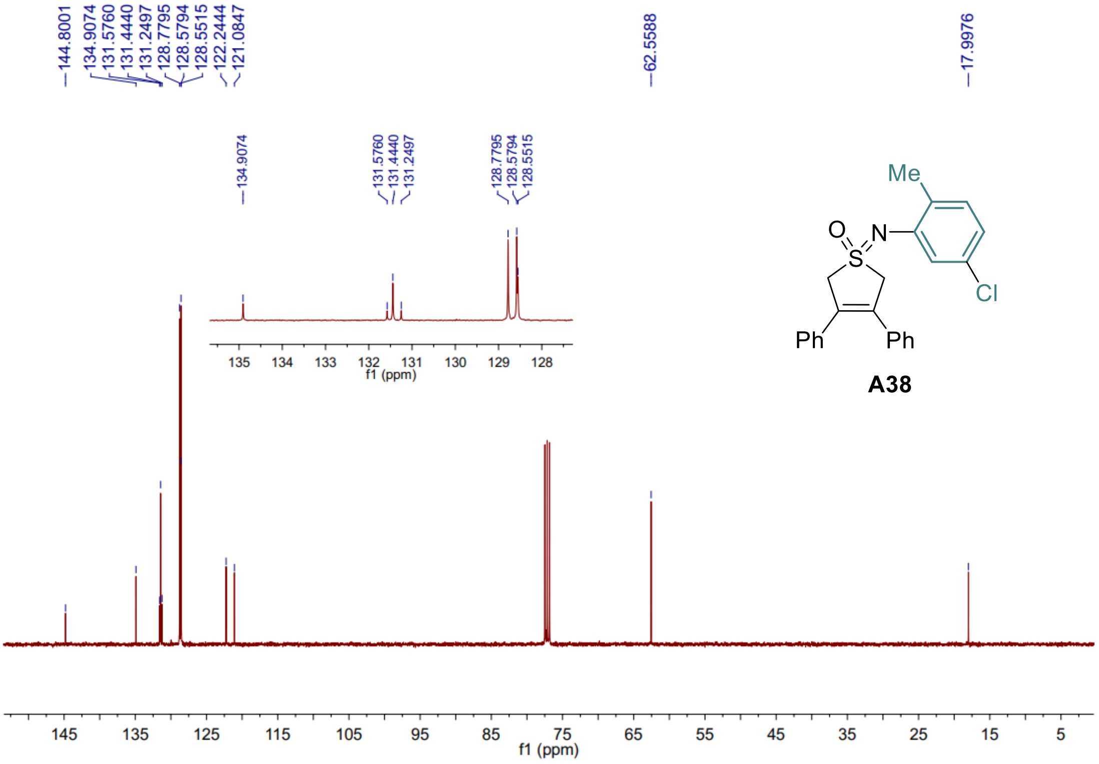

^{1}H-NMR and ^{13}C-NMR of **A38**

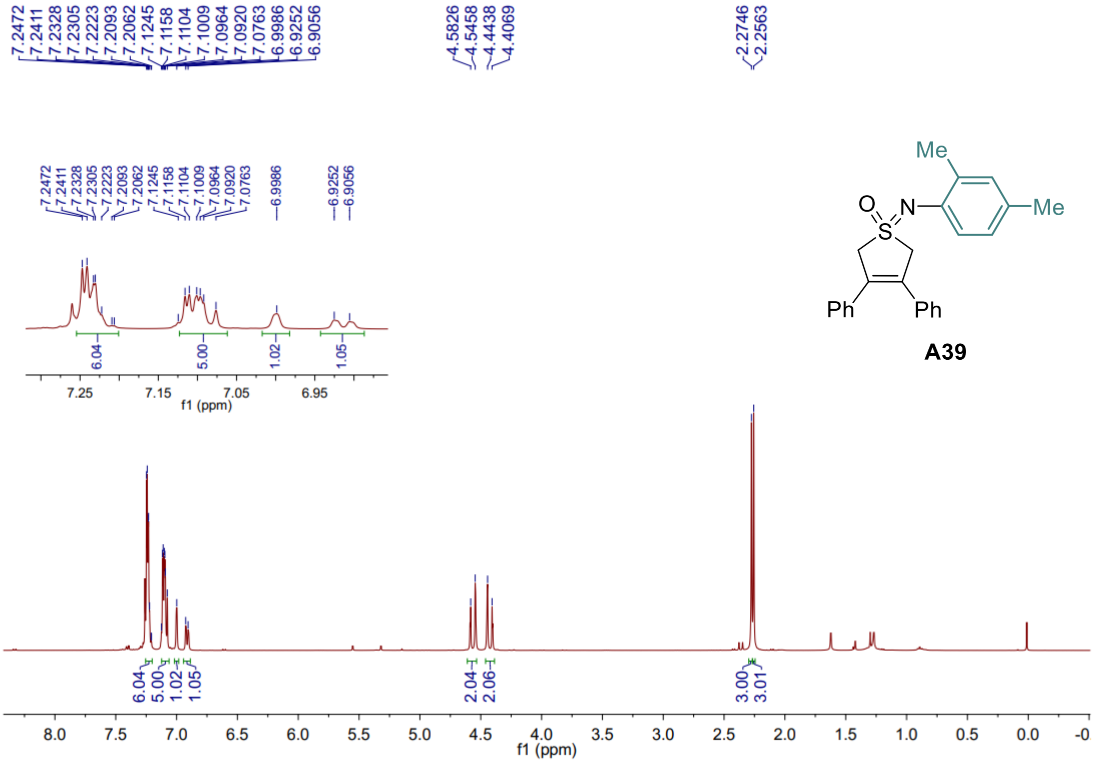

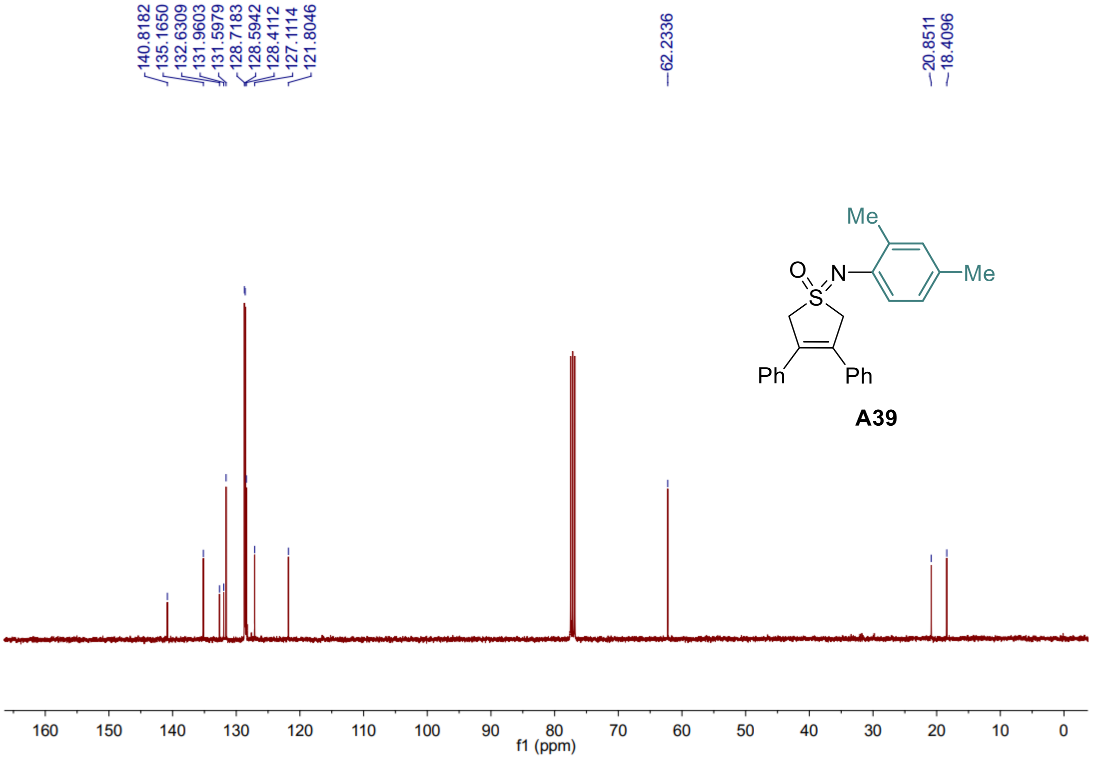

^{1}H-NMR and ^{13}C-NMR of **A39**

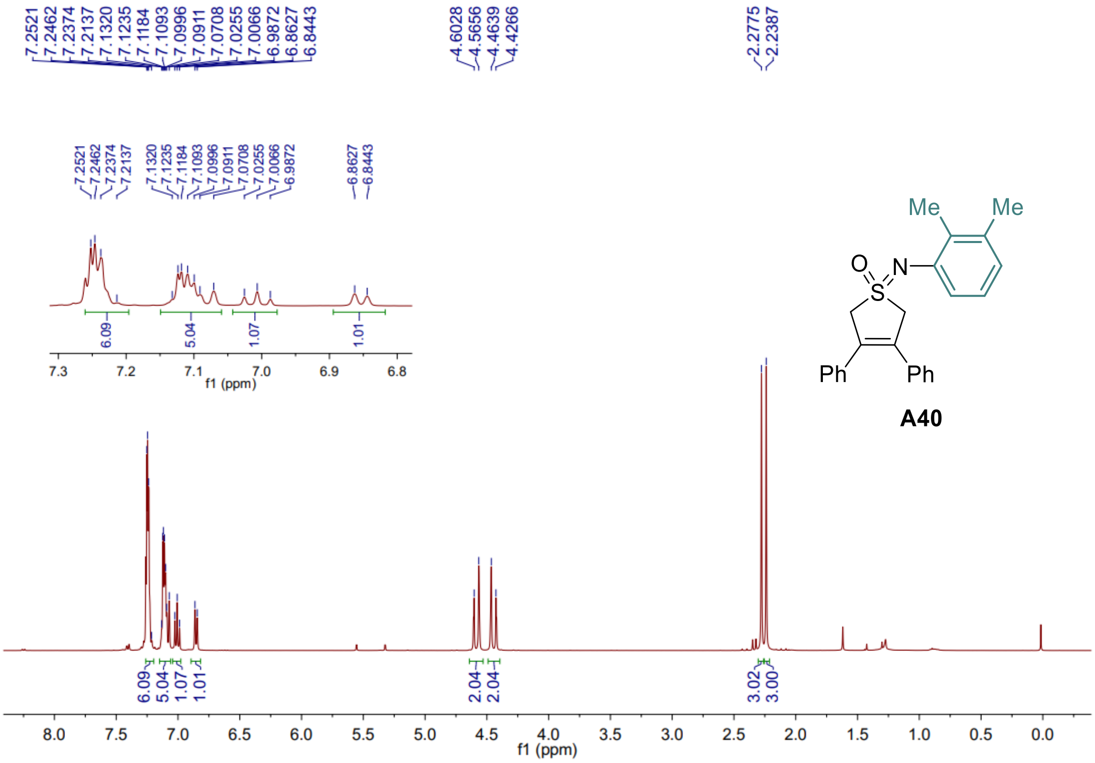

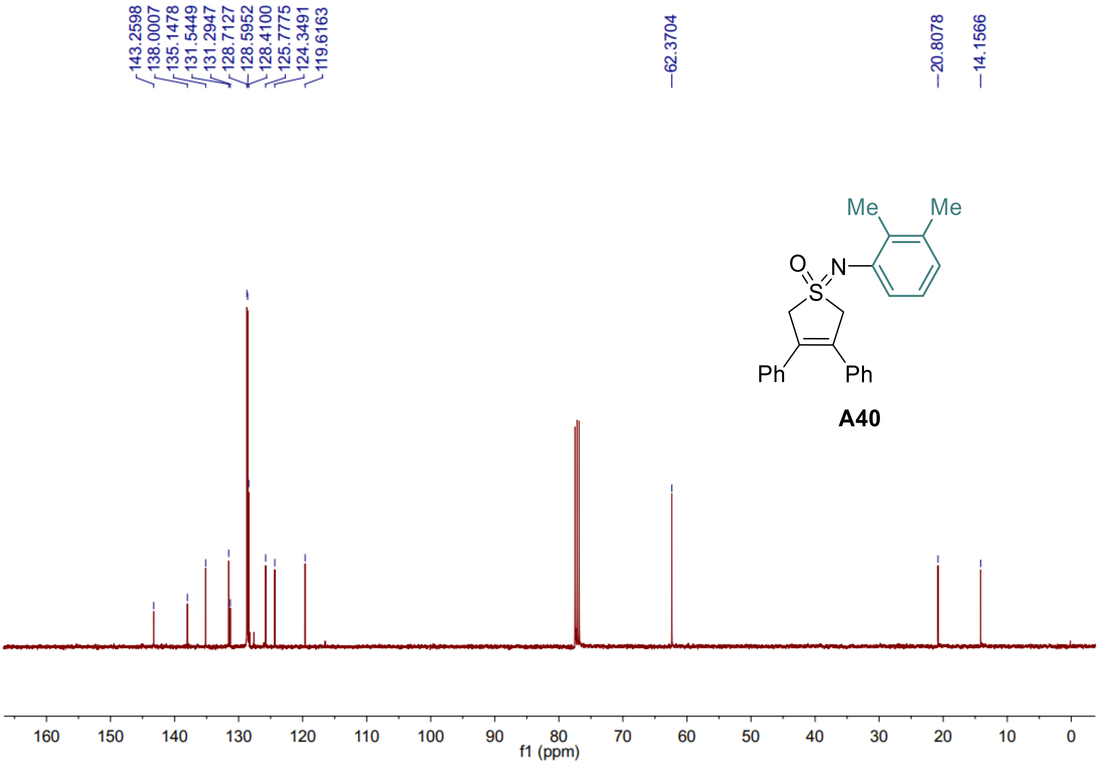

^{1}H-NMR and ^{13}C-NMR of **A40**

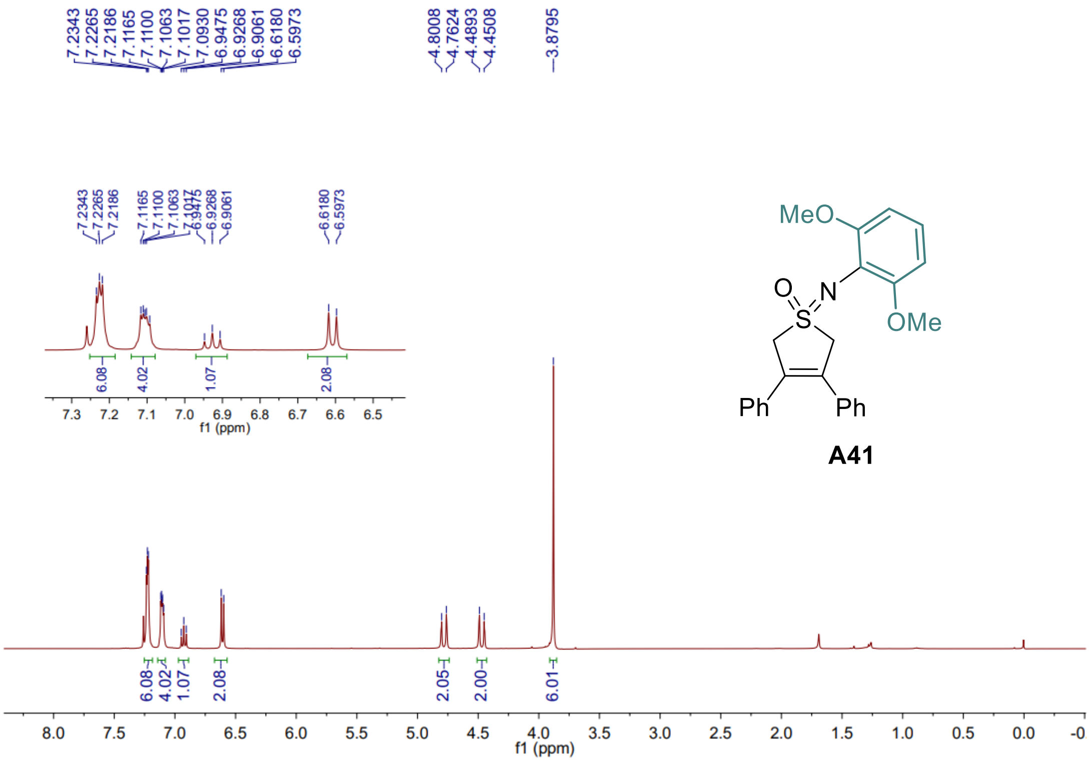

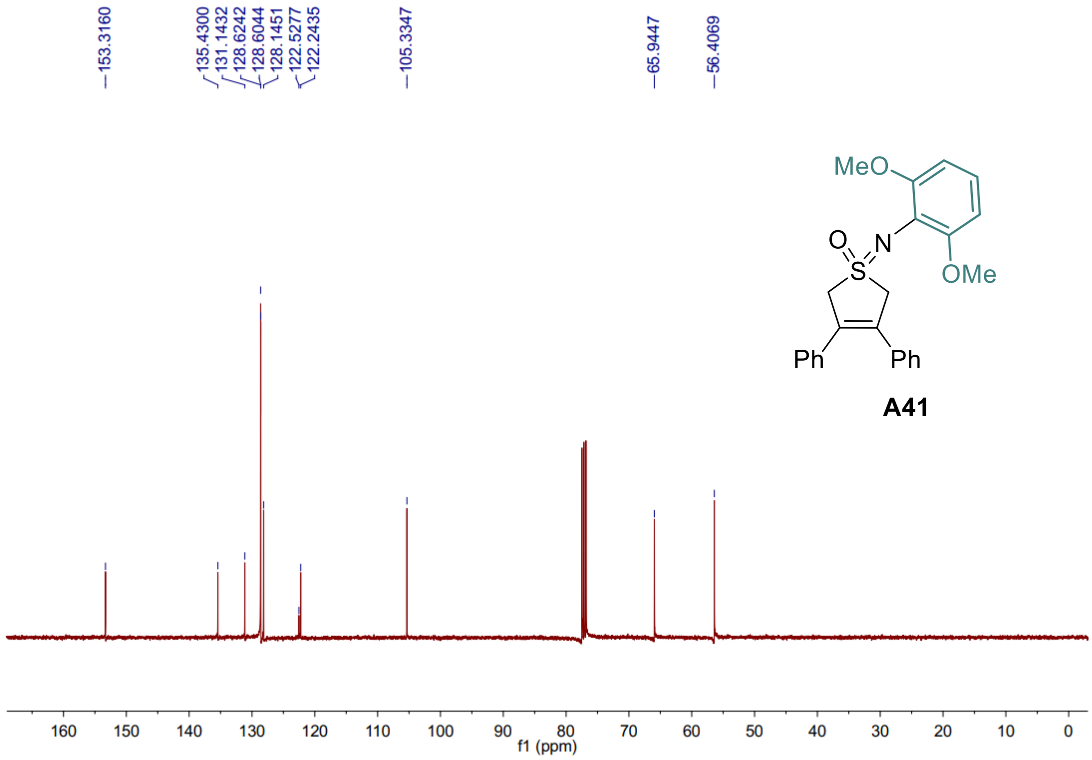

^{1}H-NMR and ^{13}C-NMR of **A41**

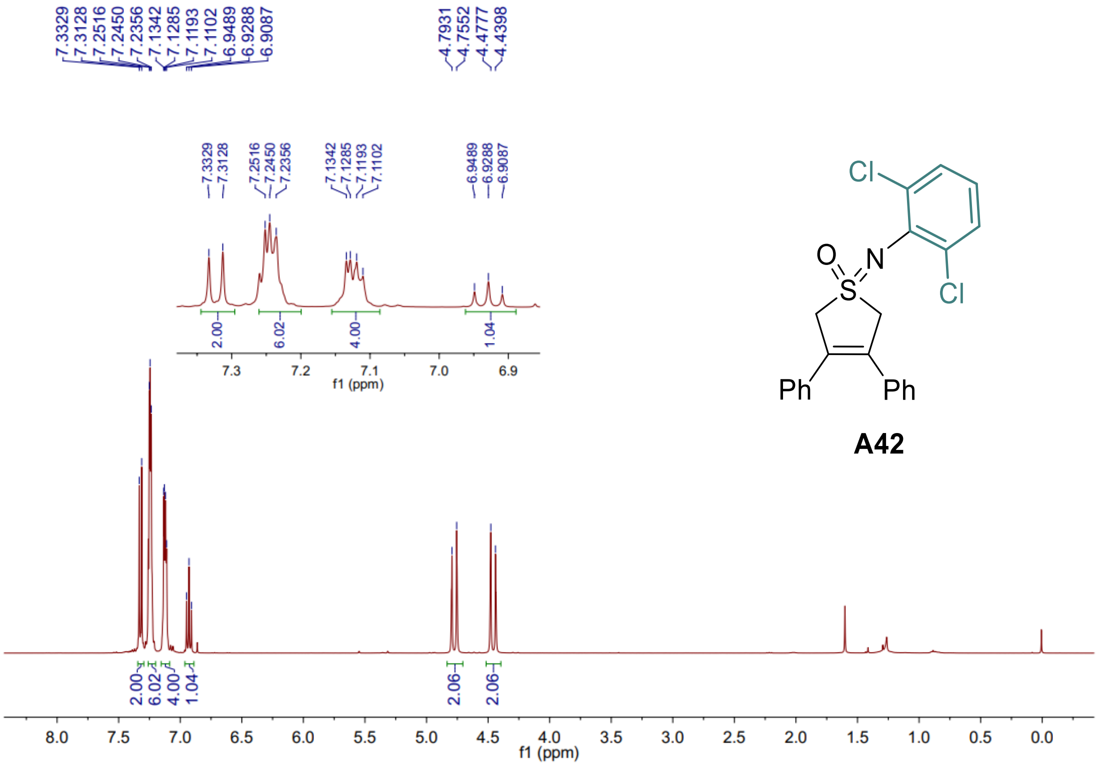

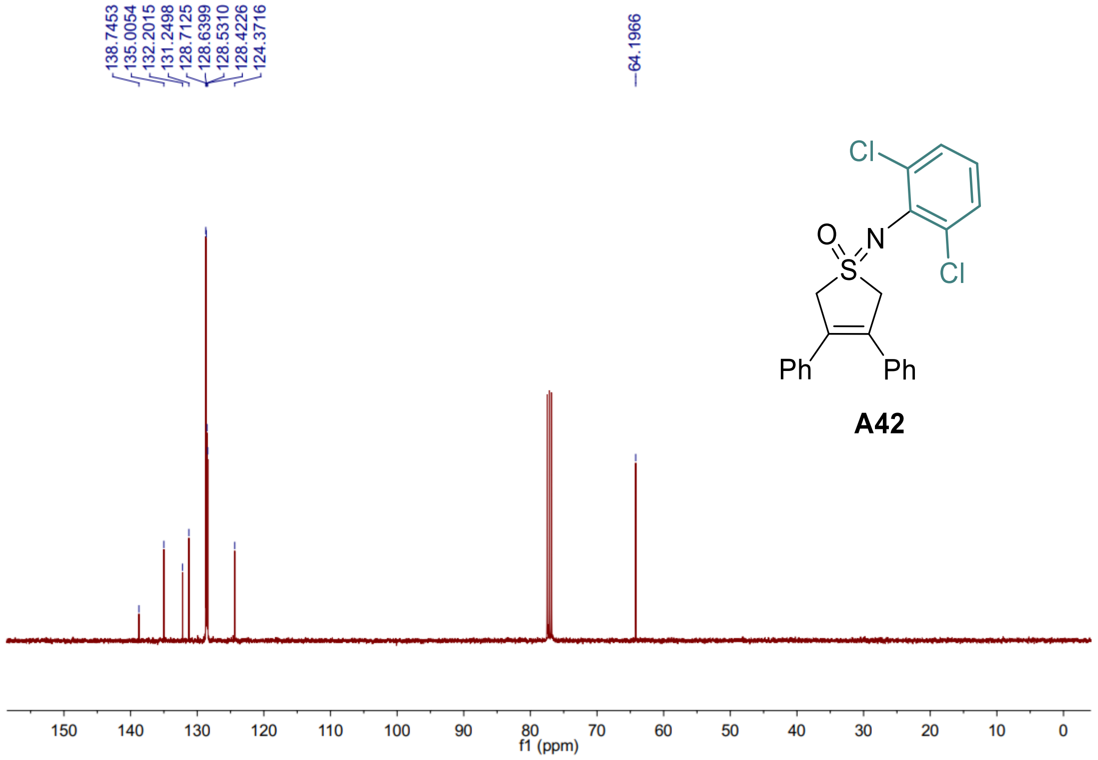

^{1}H-NMR and ^{13}C-NMR of **A42**

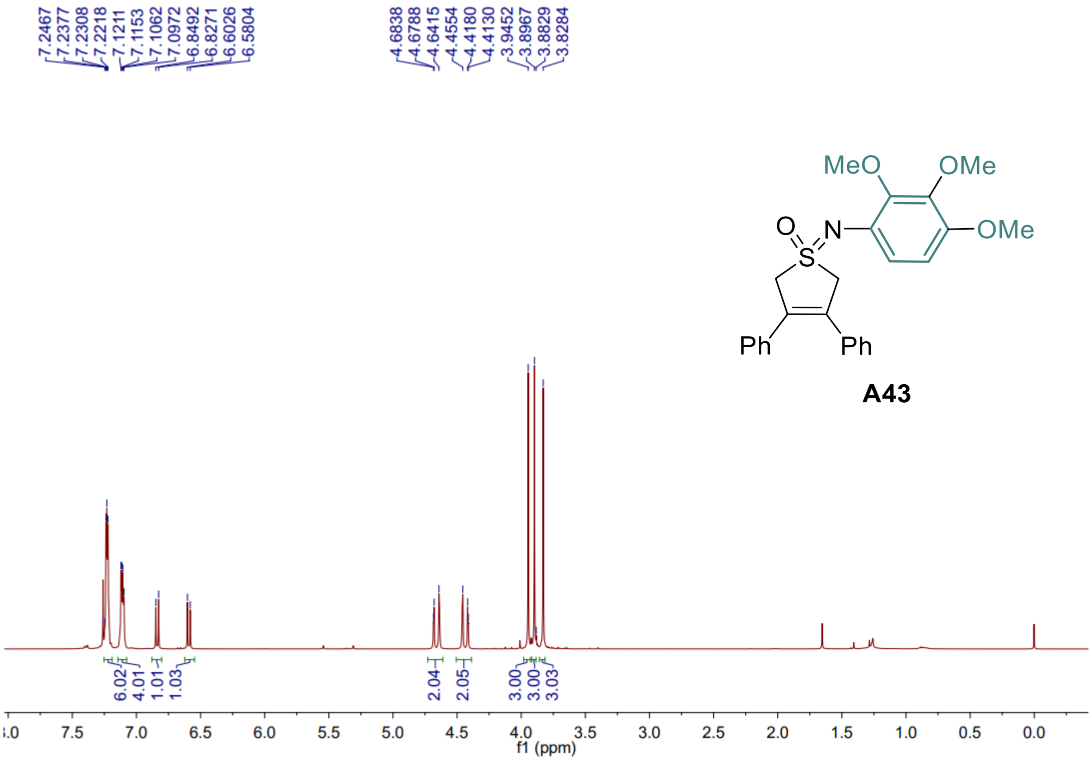

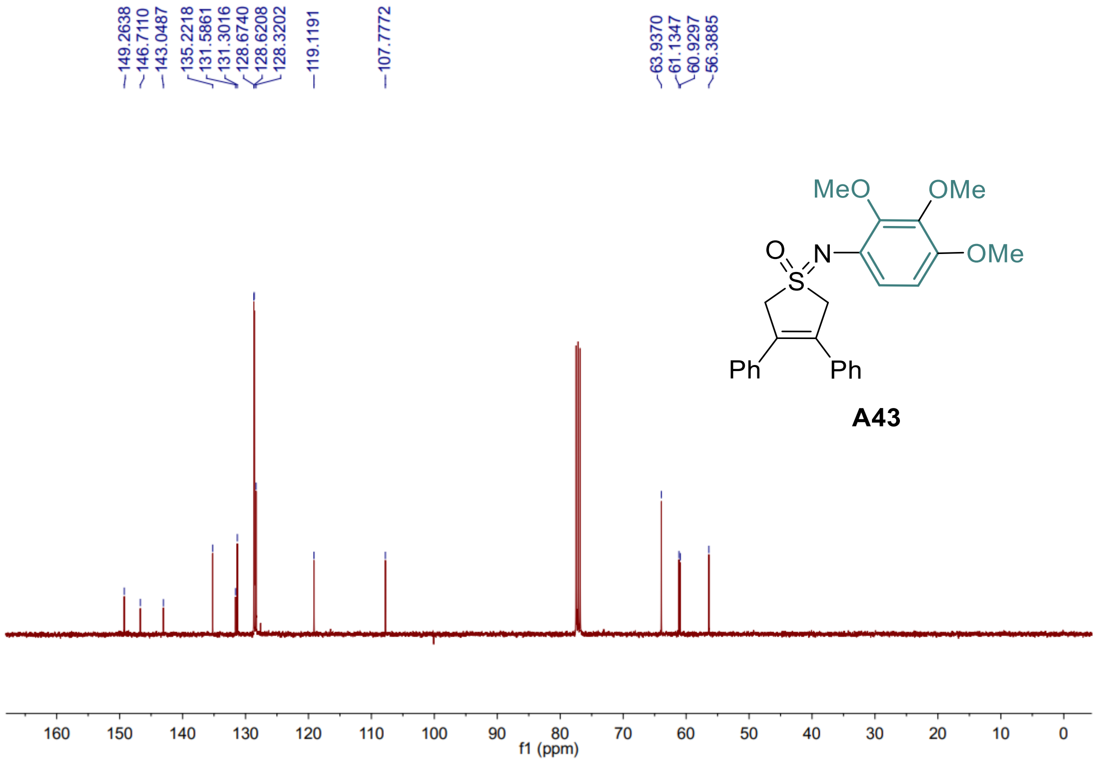

^{1}H-NMR and ^{13}C-NMR of **A43**

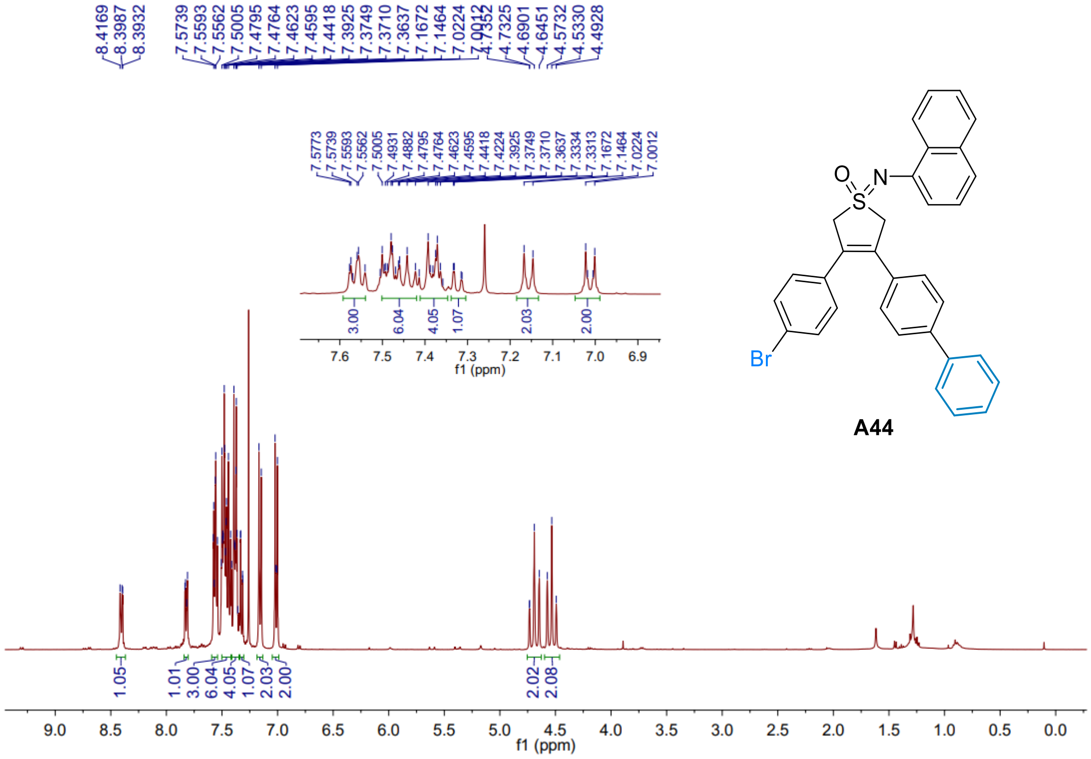

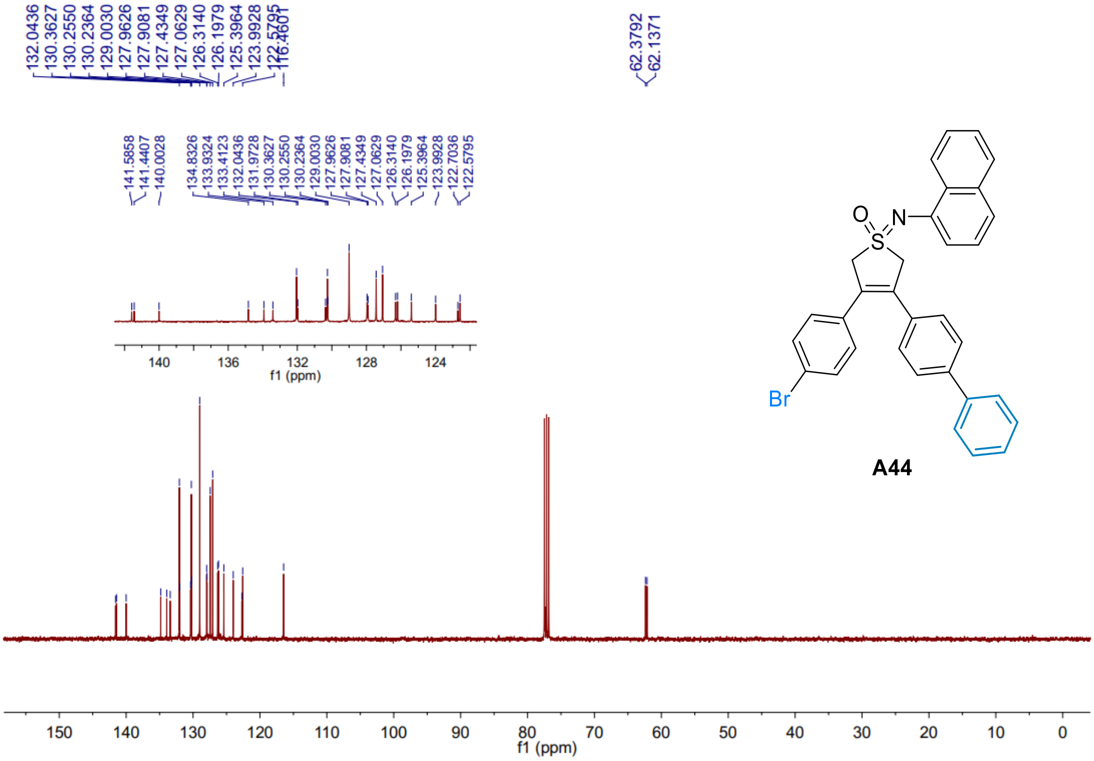

^{1}H-NMR and ^{13}C-NMR of **A44**

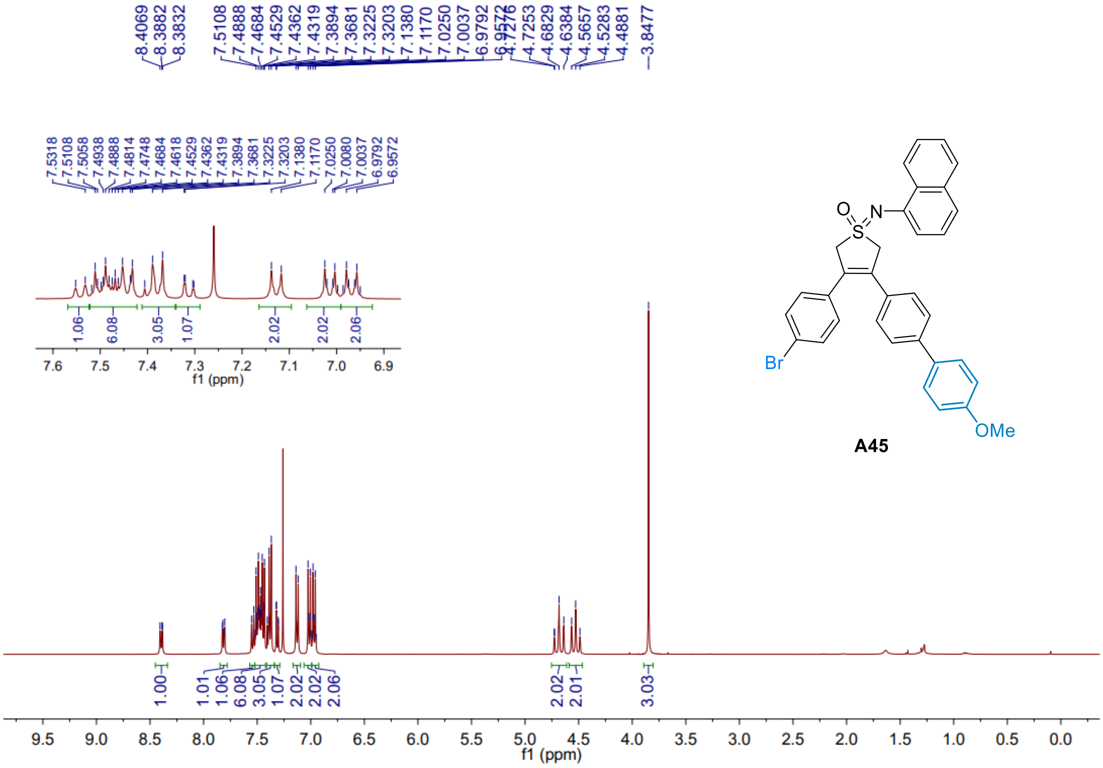

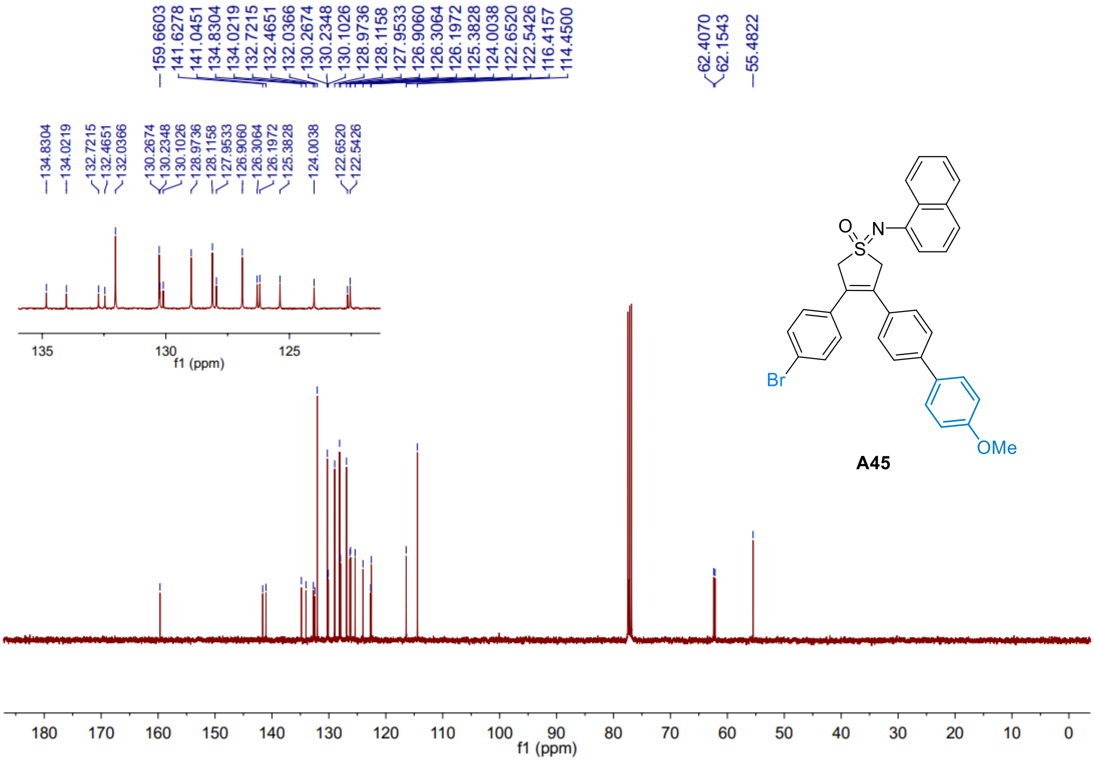

^{1}H-NMR and ^{13}C-NMR of **A45**

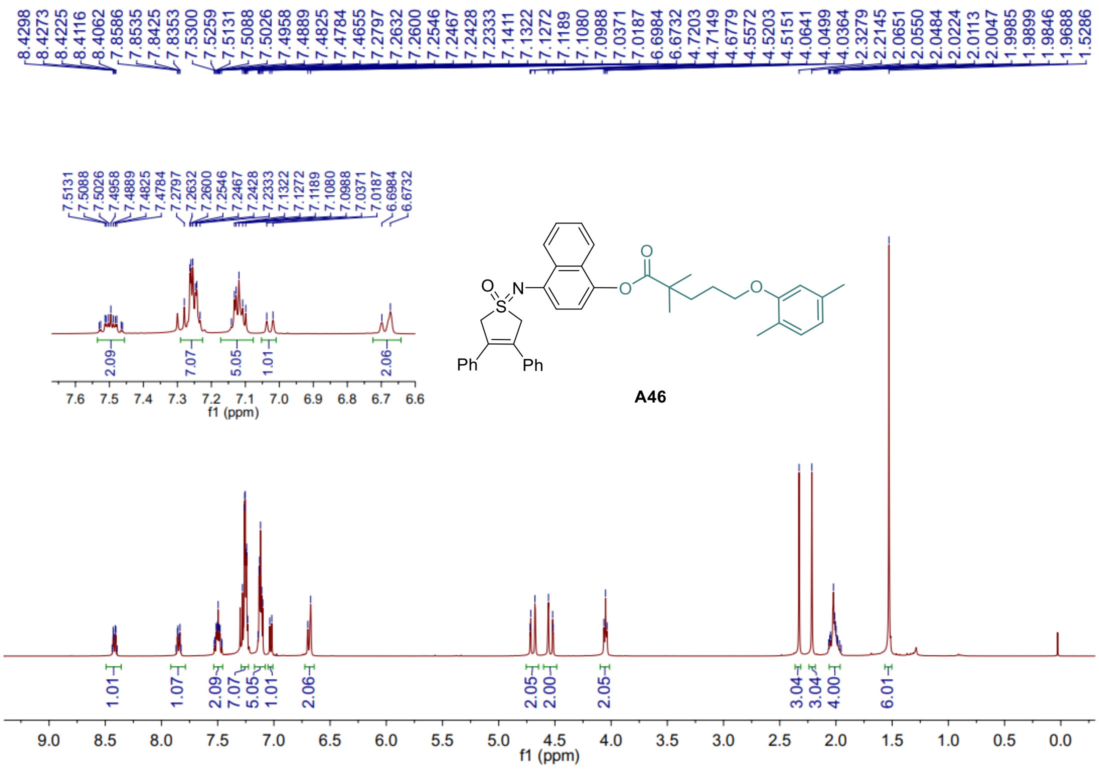

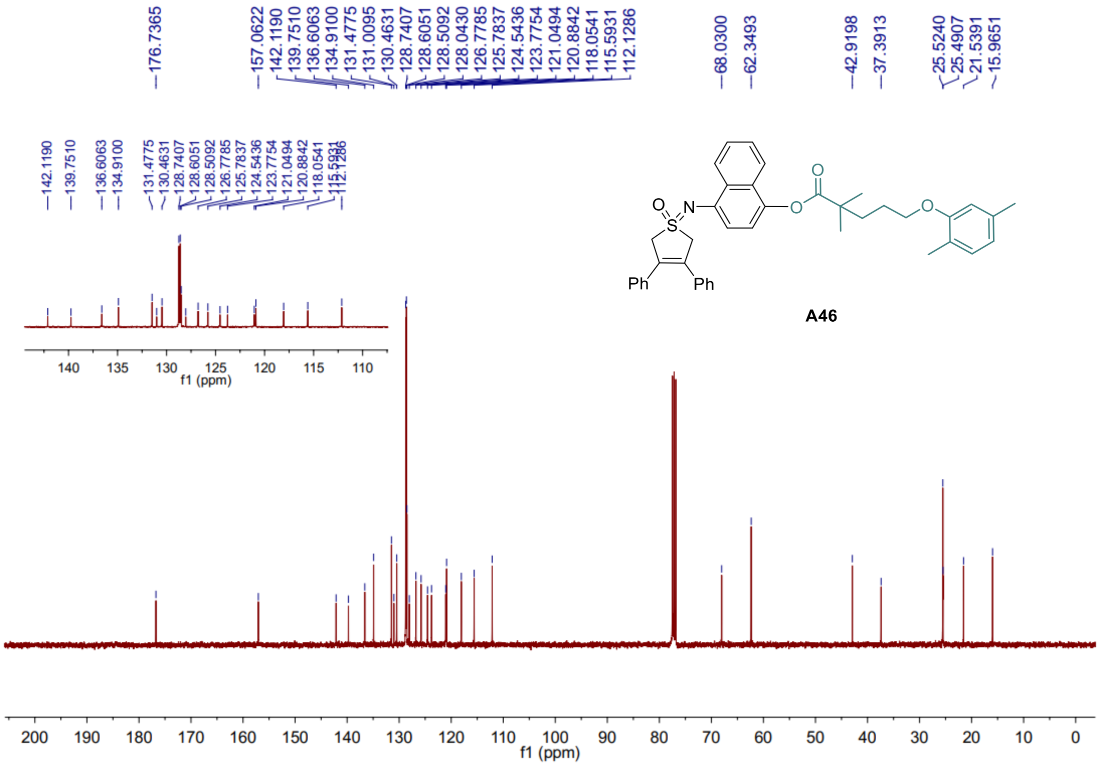

^{1}H-NMR and ^{13}C-NMR of **A46**

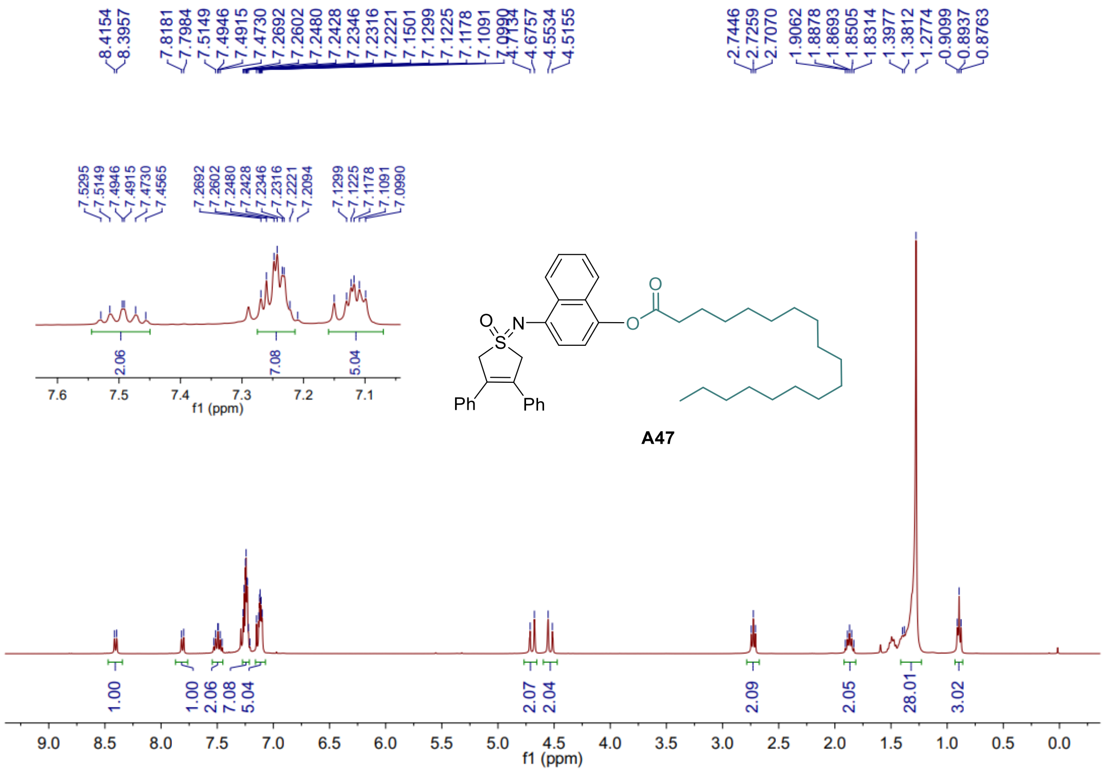

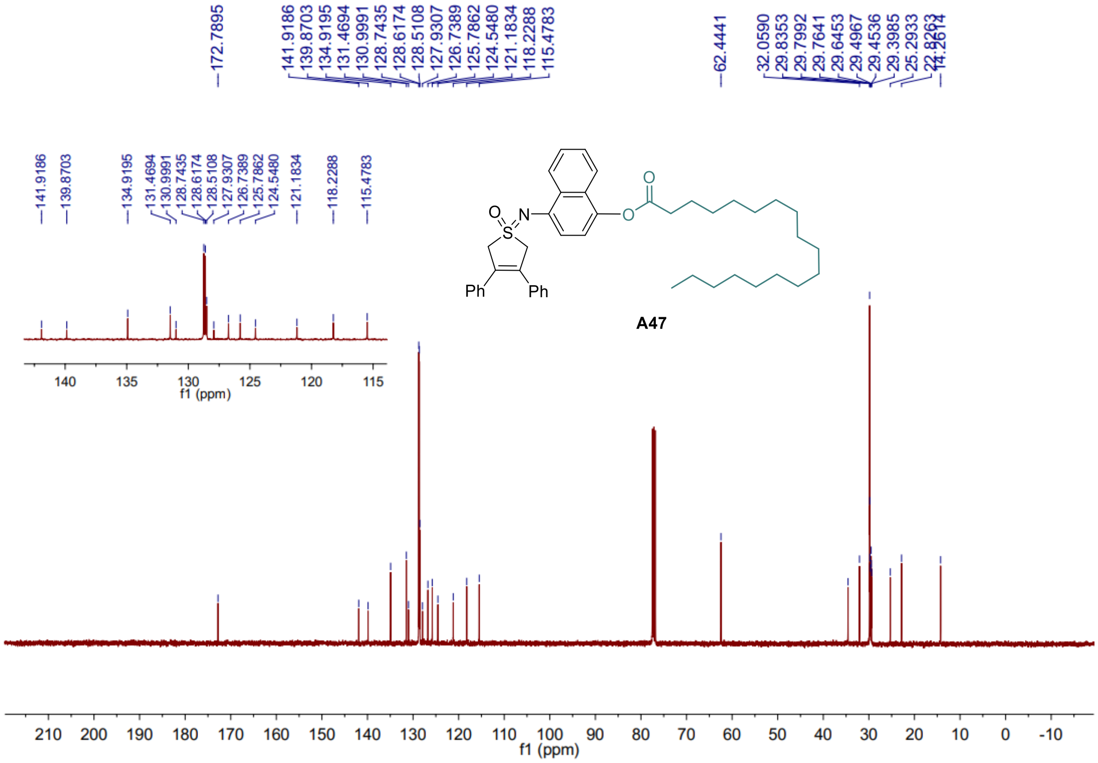

^{1}H-NMR and ^{13}C-NMR of **A47**

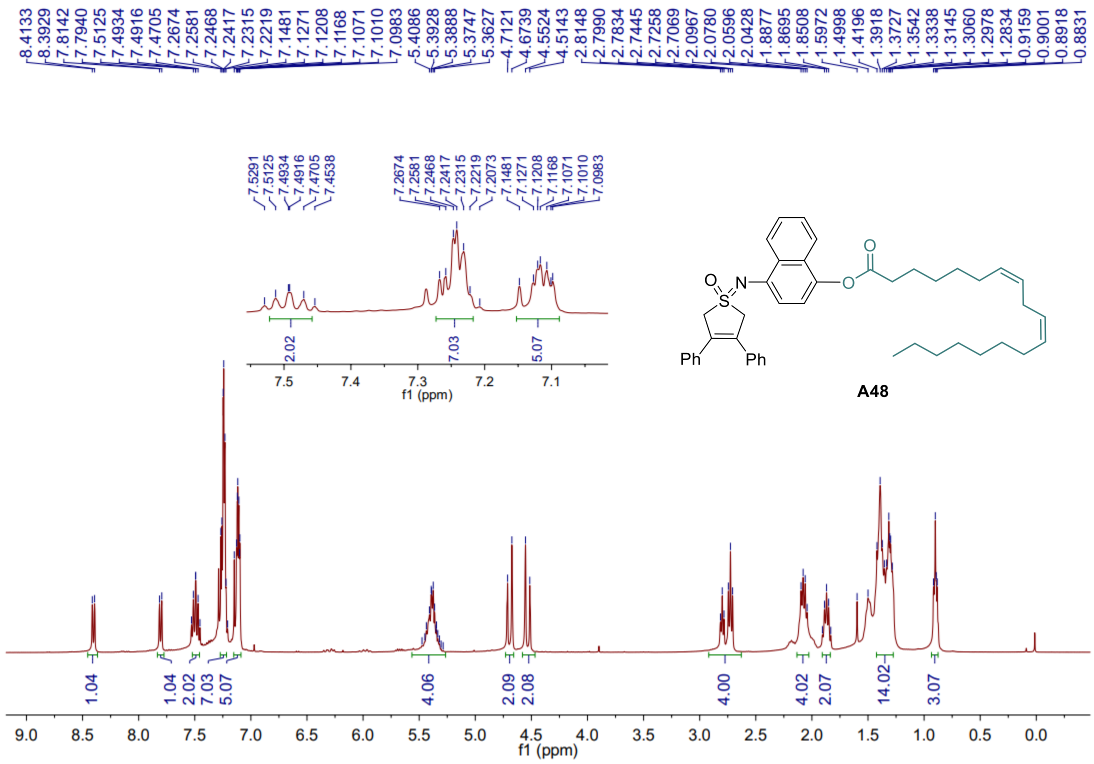

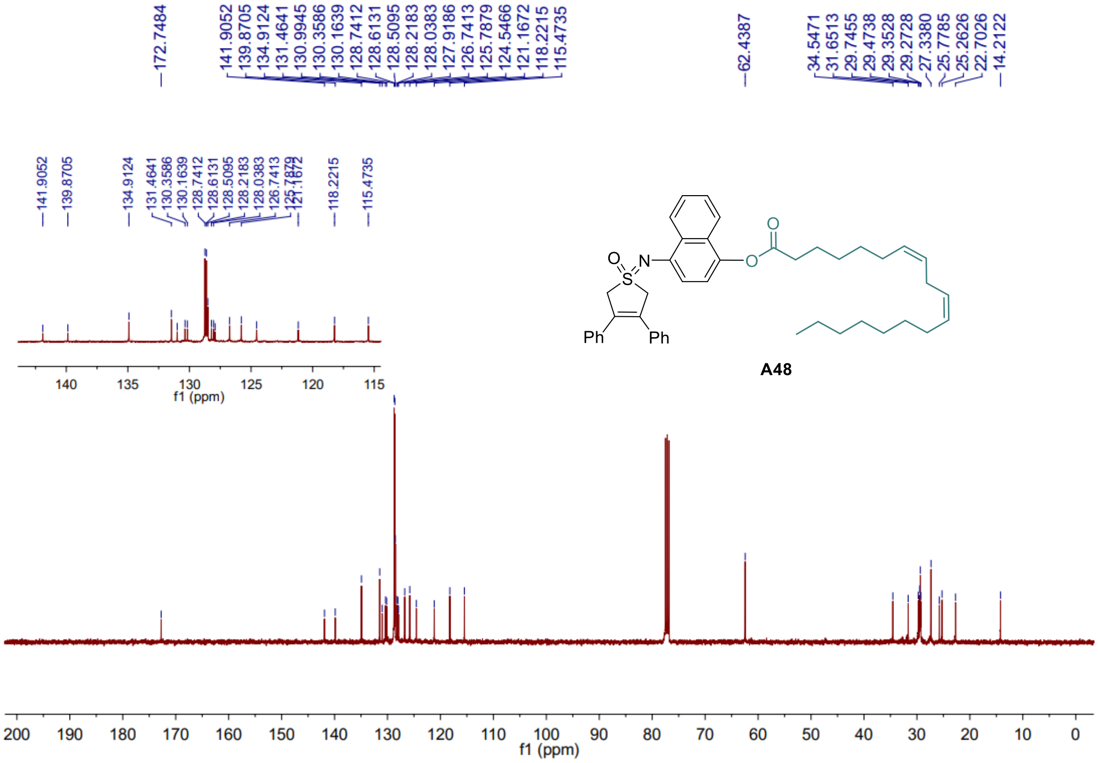

^{1}H-NMR and ^{13}C-NMR of **A48**

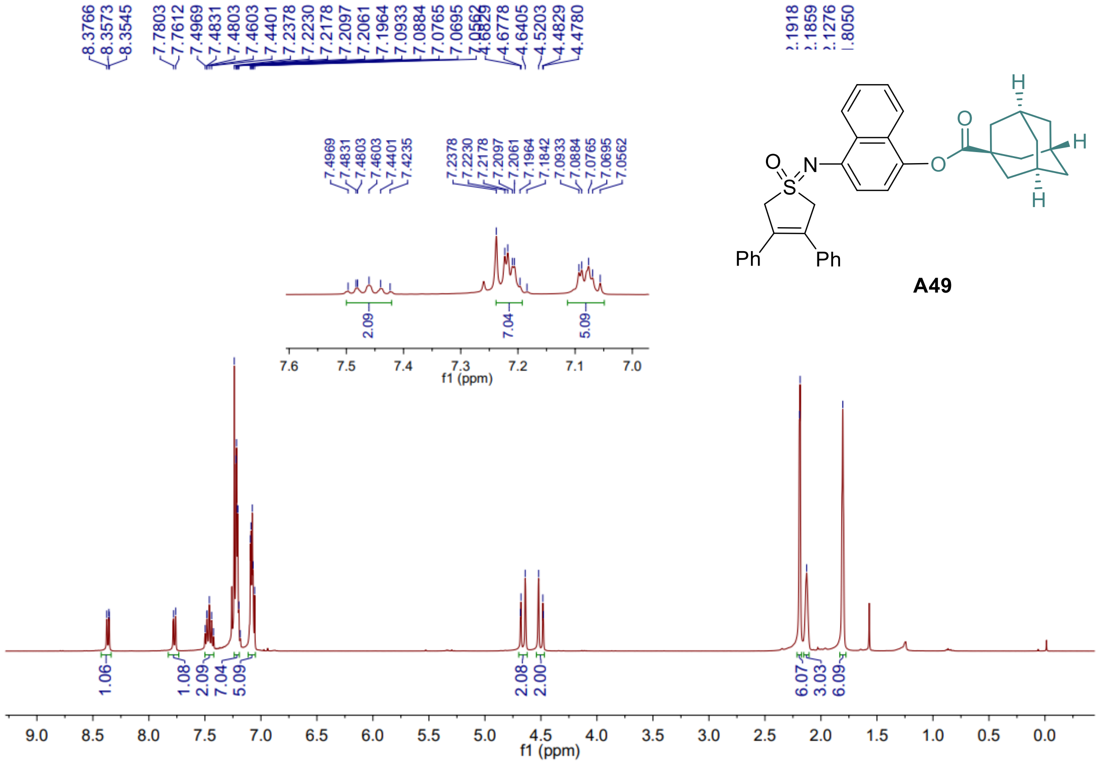

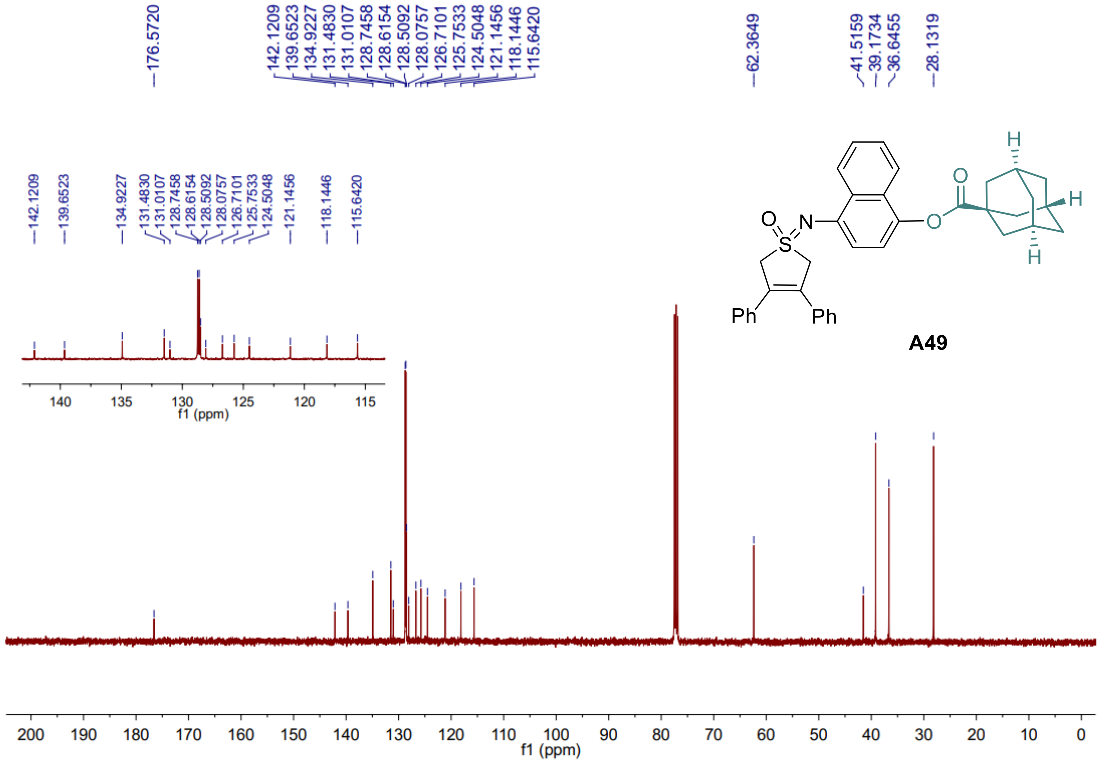

^{1}H-NMR and ^{13}C-NMR of **A49**

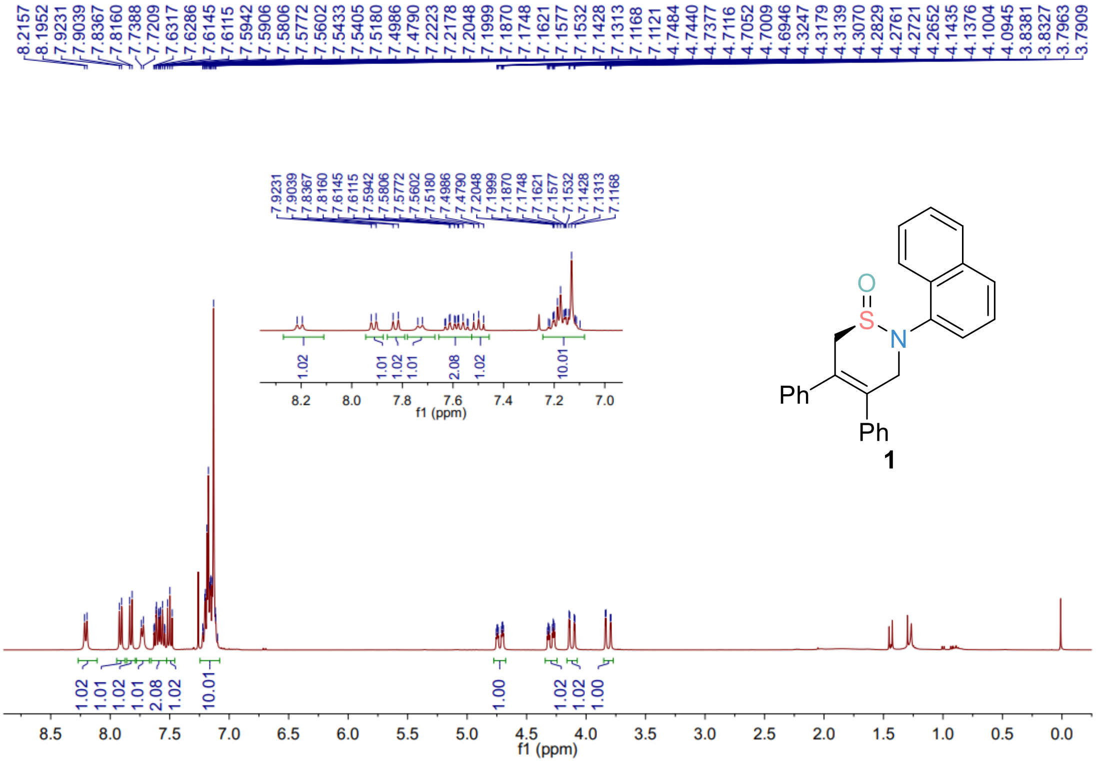

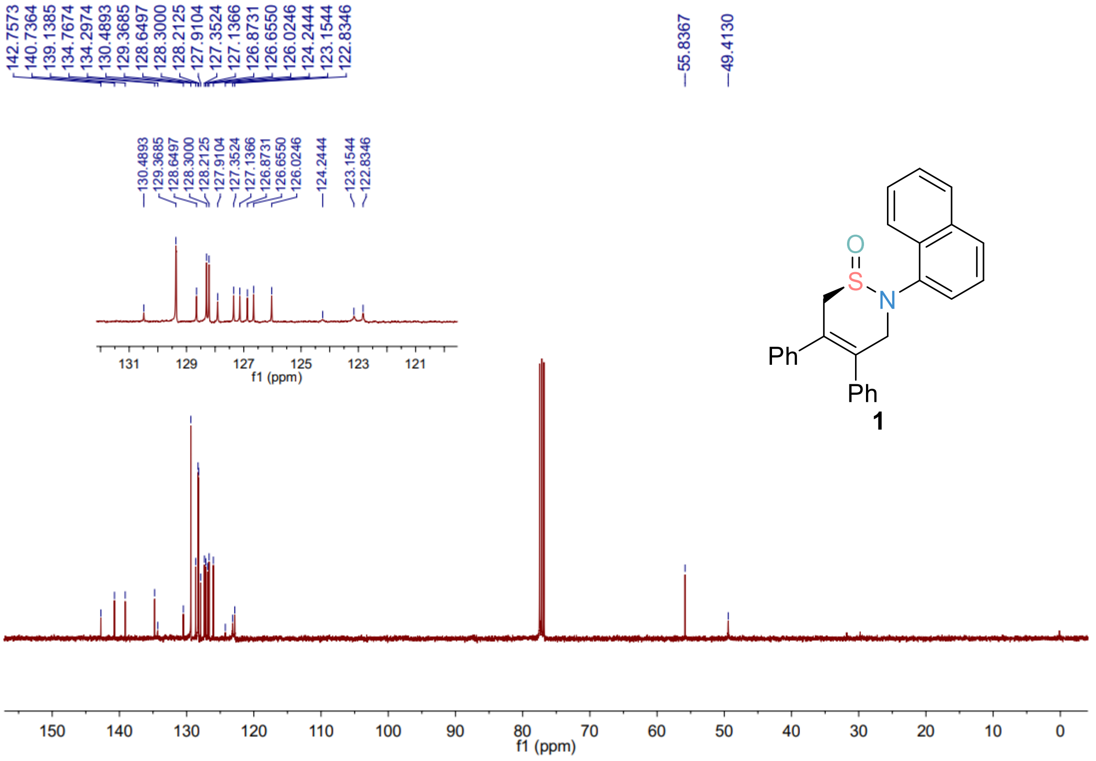

^{1}H-NMR and ^{13}C-NMR of **1**

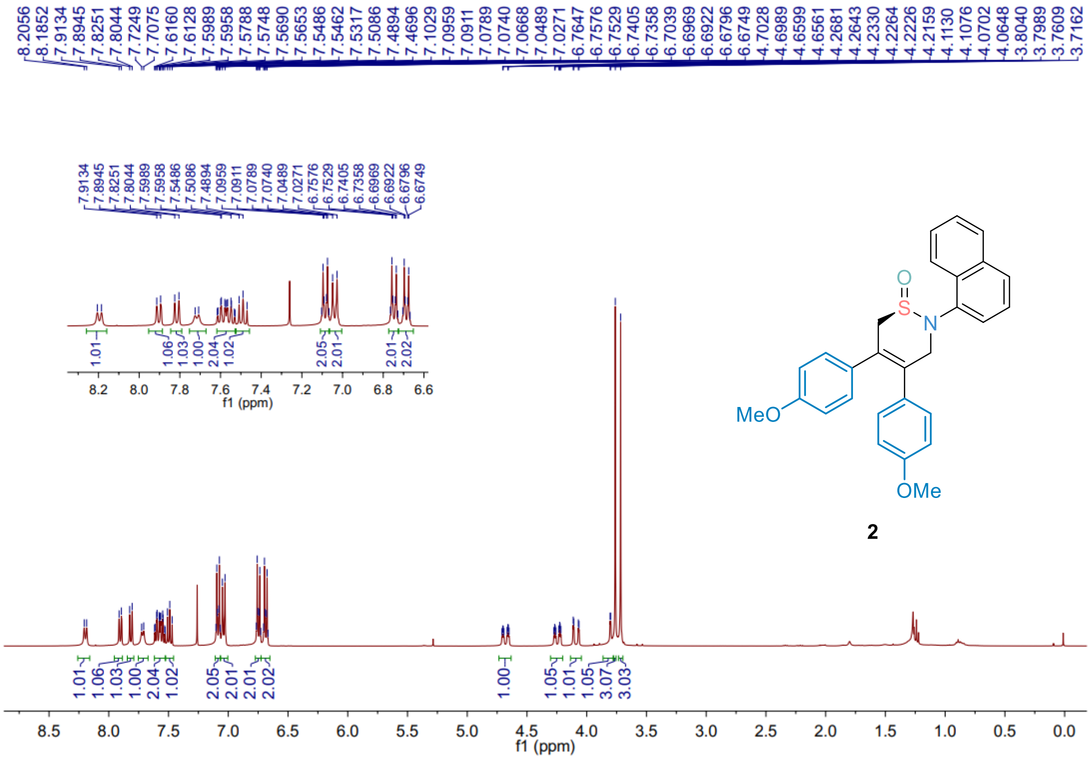

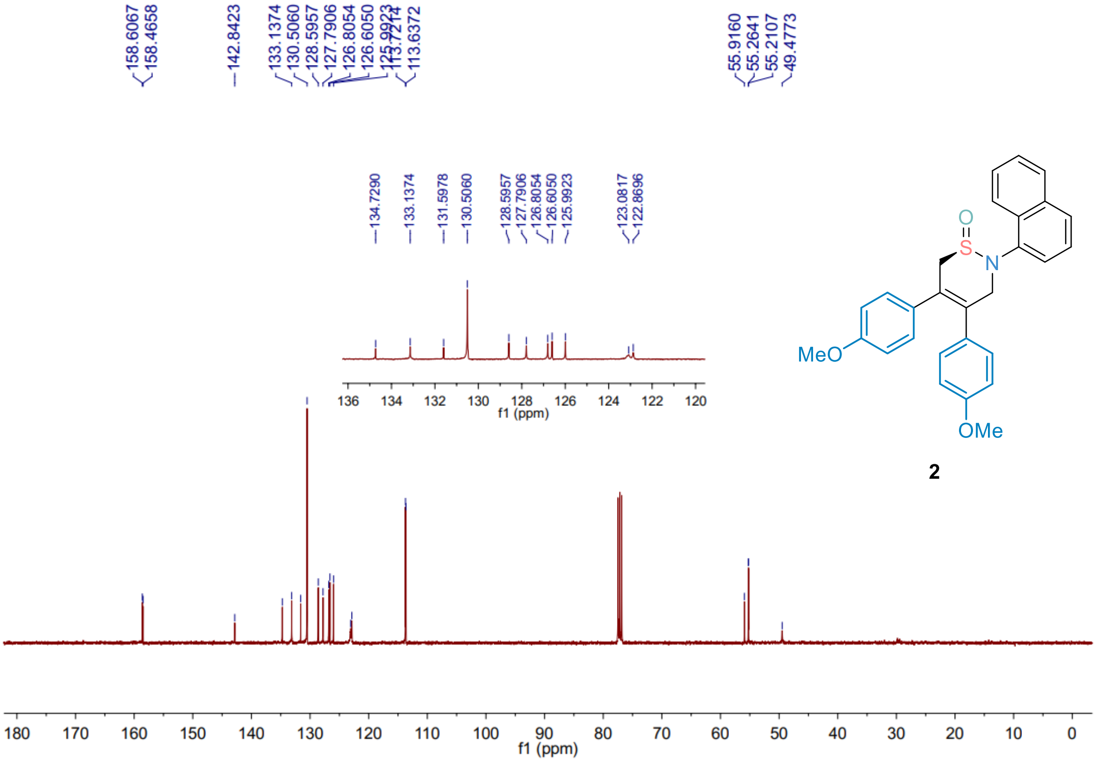

^{1}H-NMR and ^{13}C-NMR of **2**

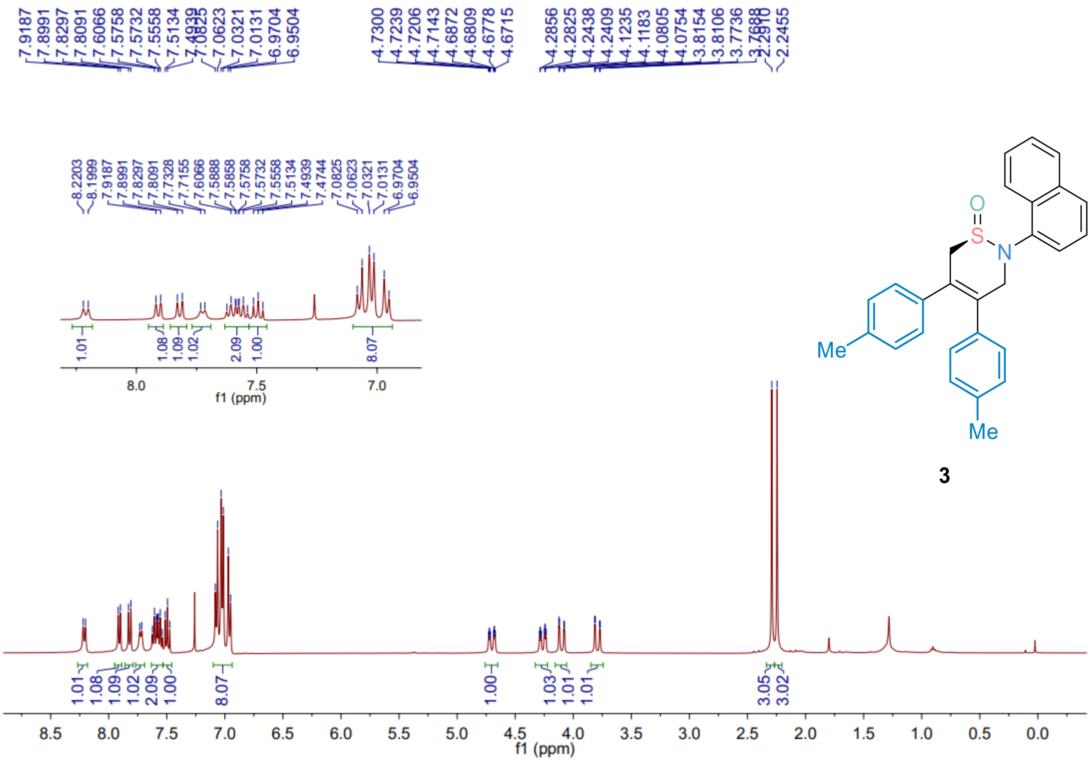

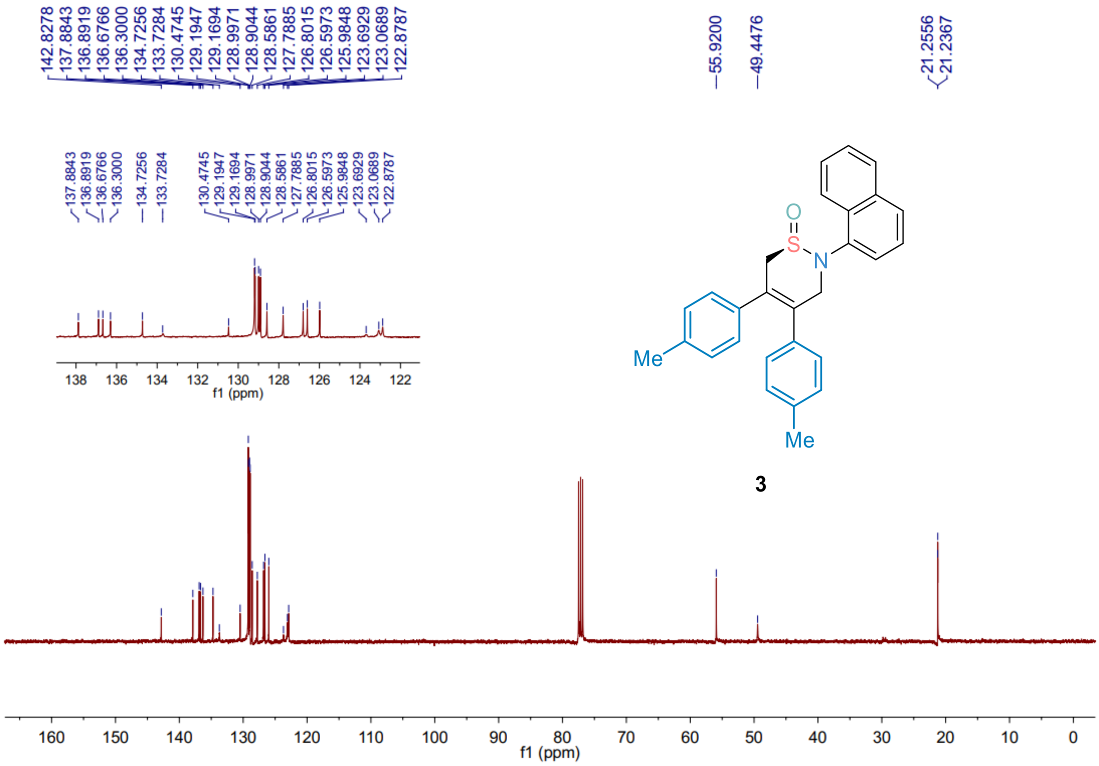

^{1}H-NMR and ^{13}C-NMR of **3**

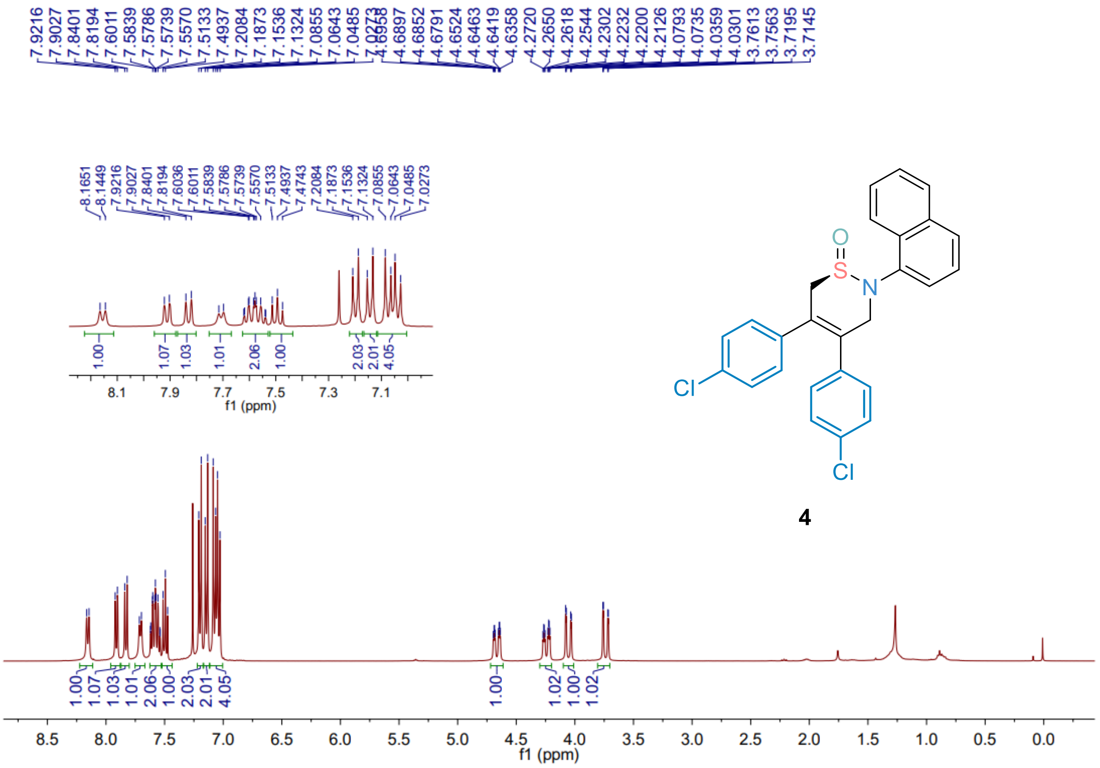

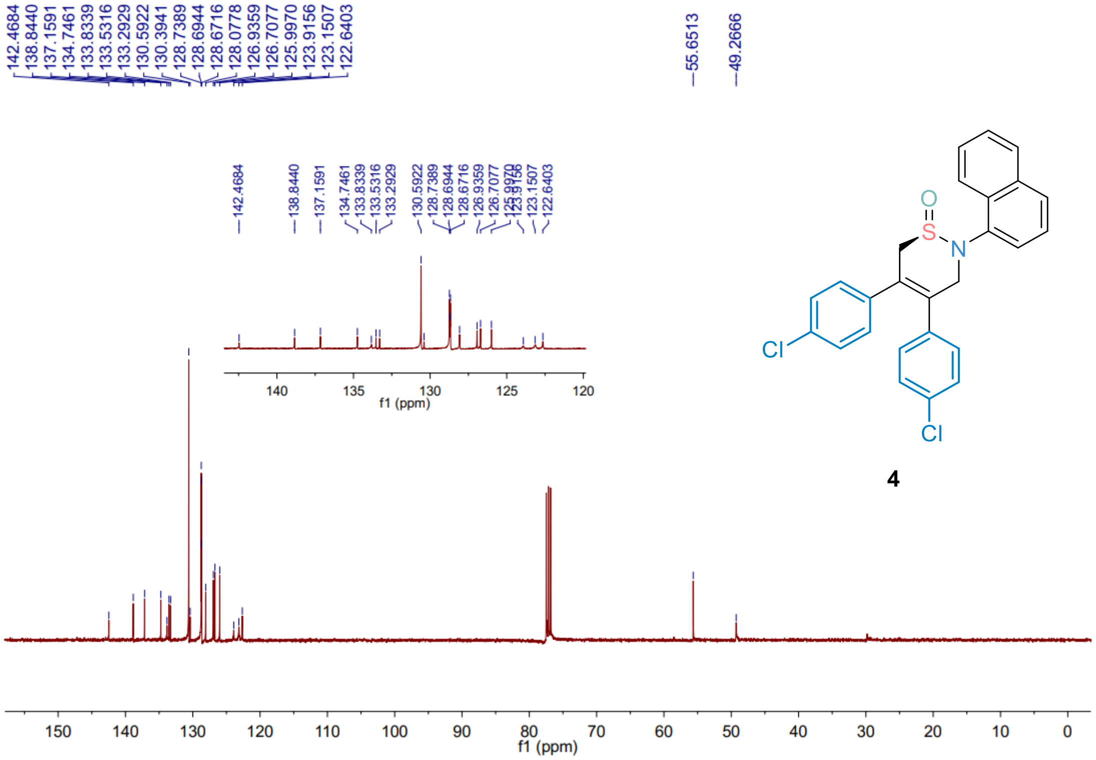

^{1}H-NMR and ^{13}C-NMR of **4**

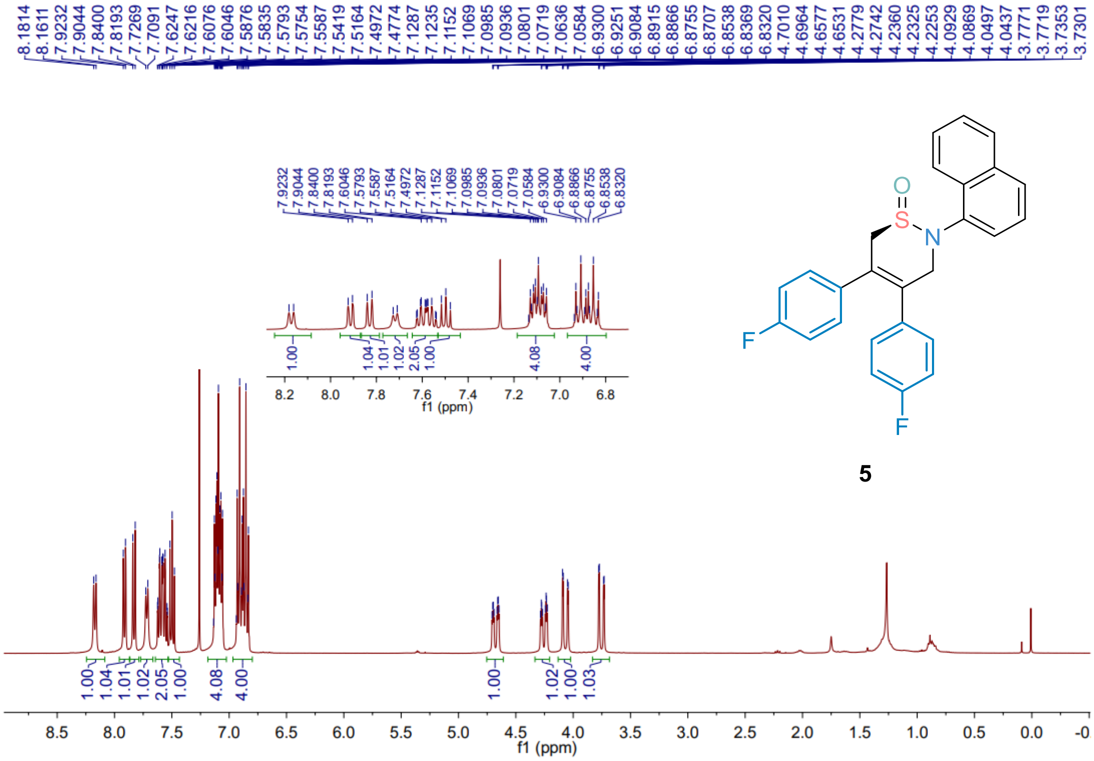

5

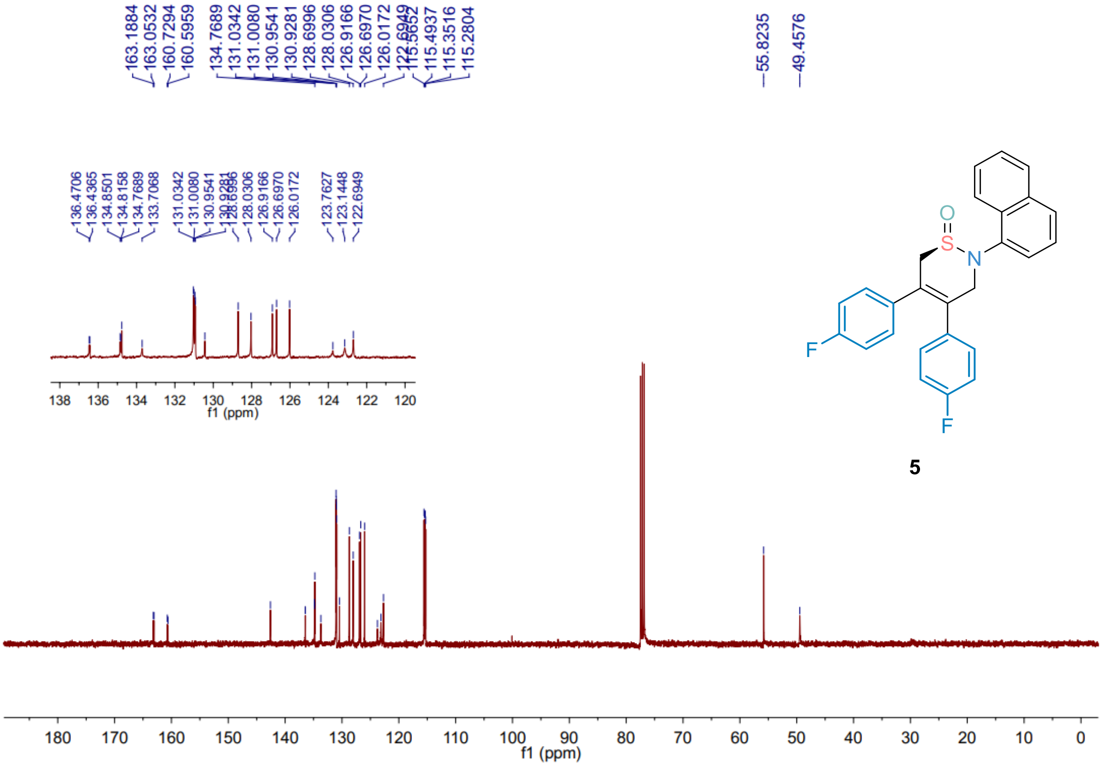

5

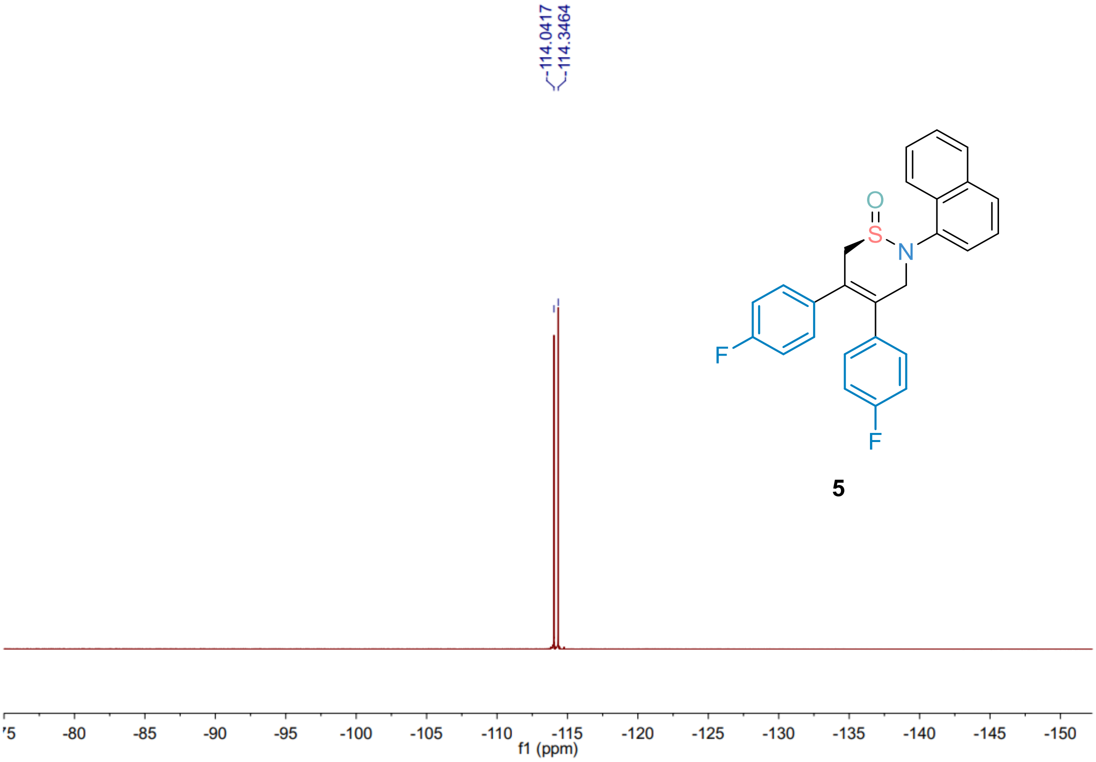

^{1}H-NMR and ^{13}C-NMR of **5**

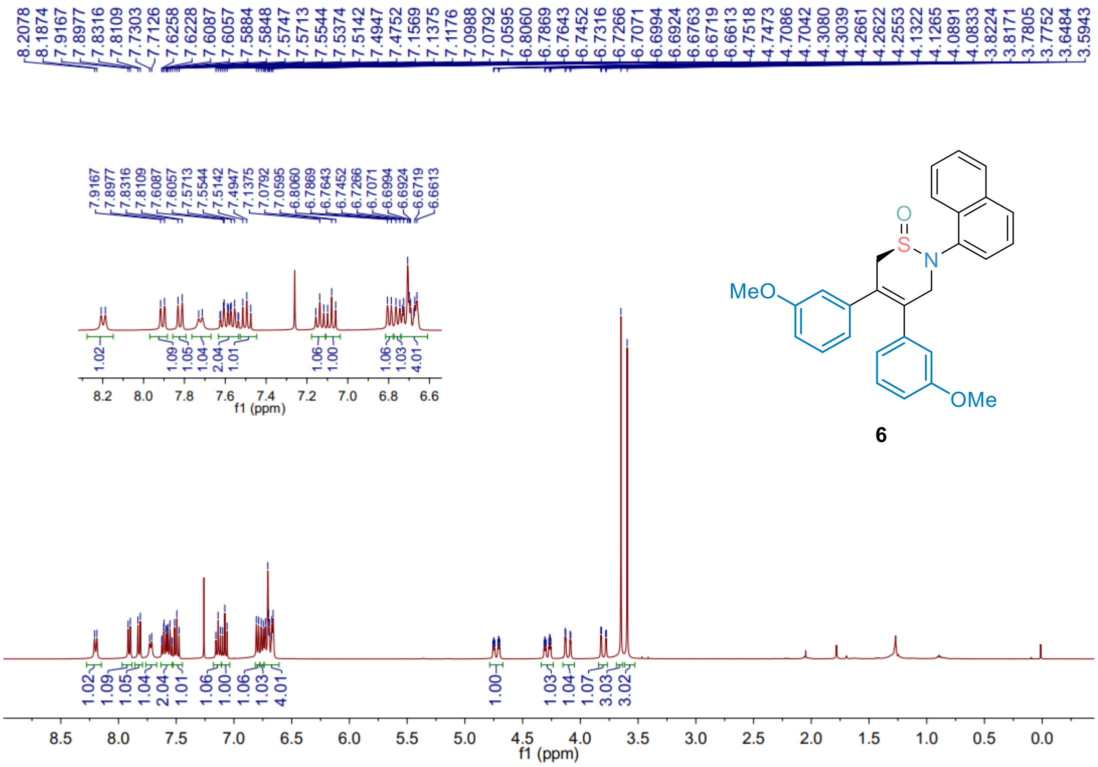

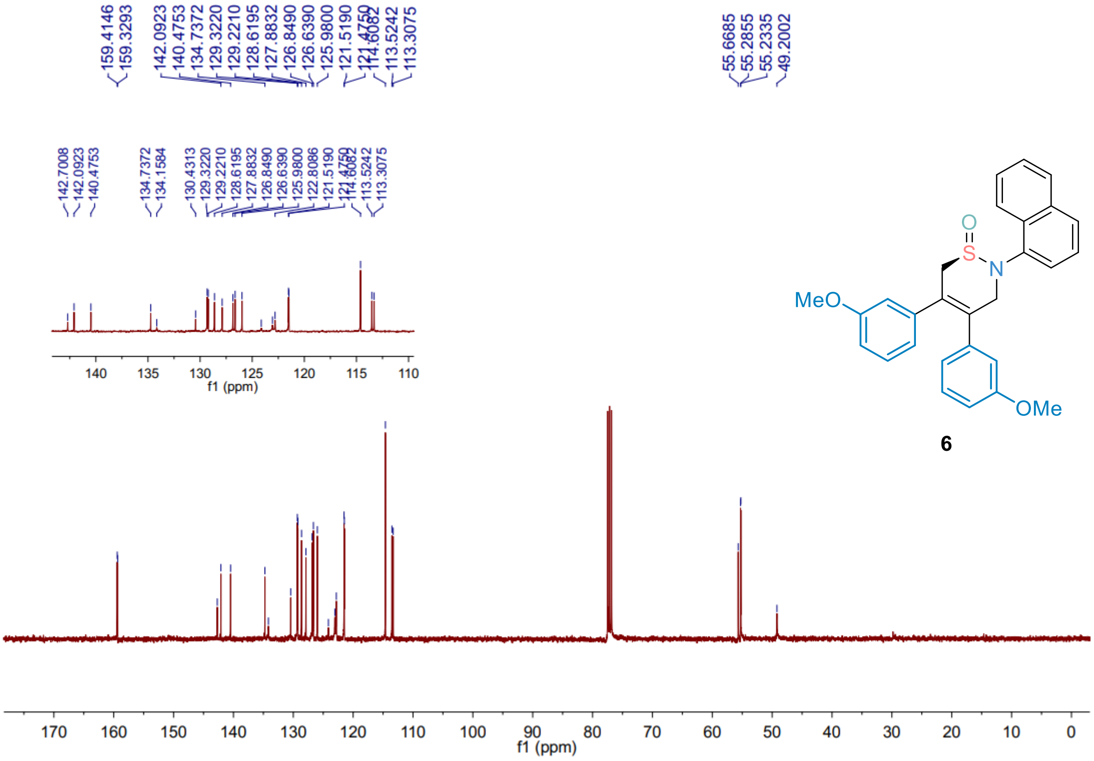

^{1}H-NMR and ^{13}C-NMR of **6**

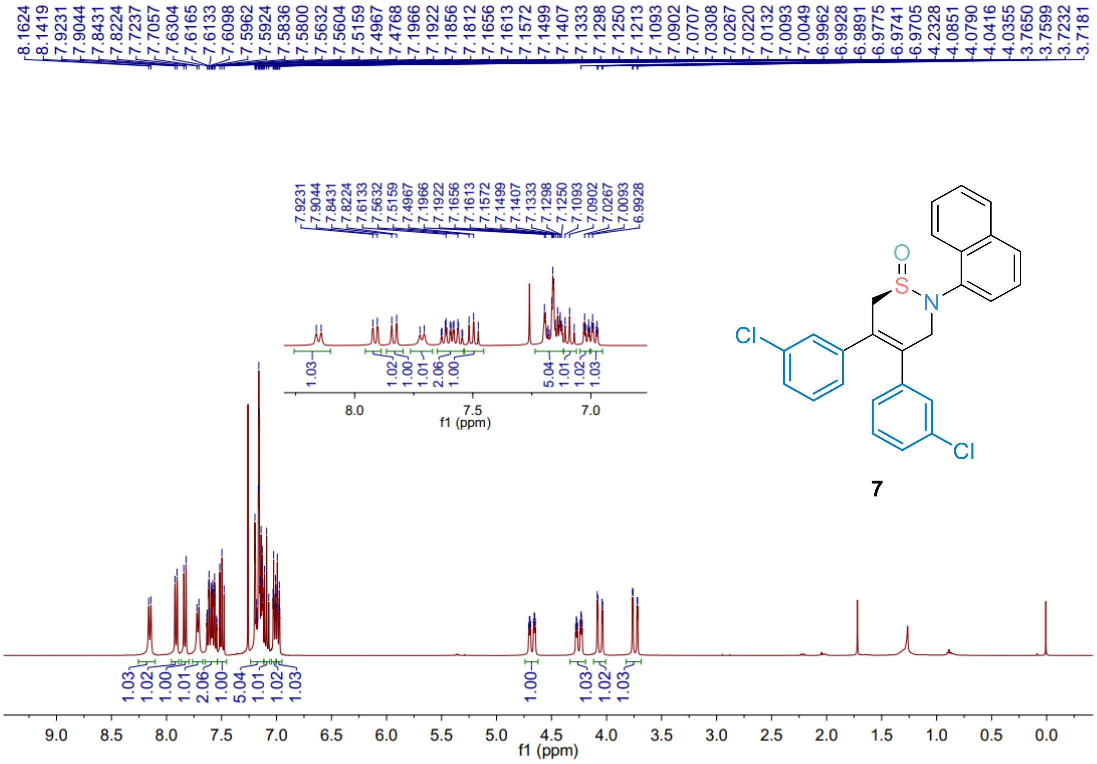

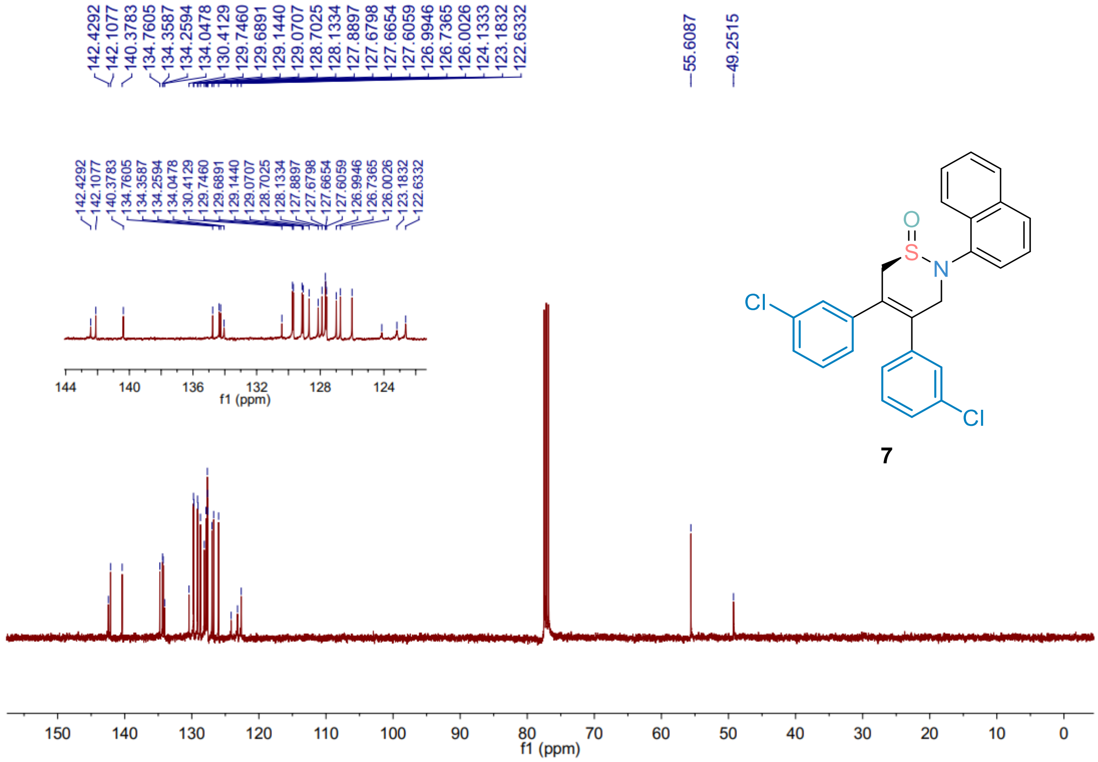

^{1}H-NMR and ^{13}C-NMR of **7**

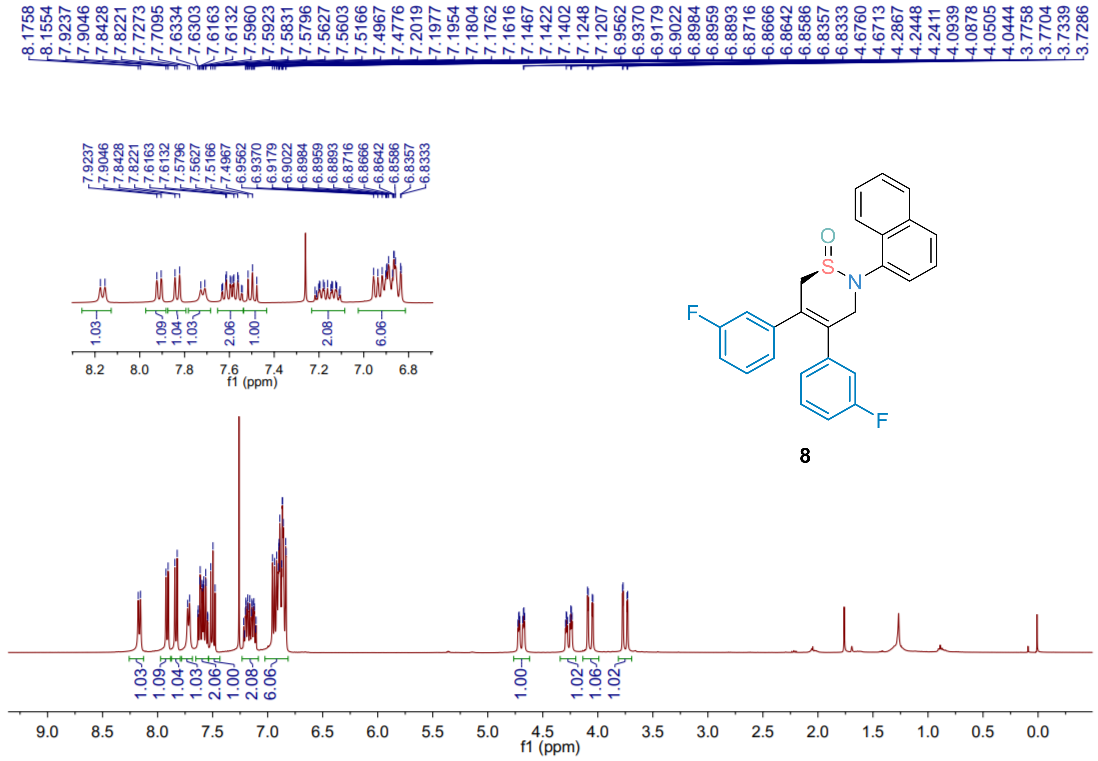

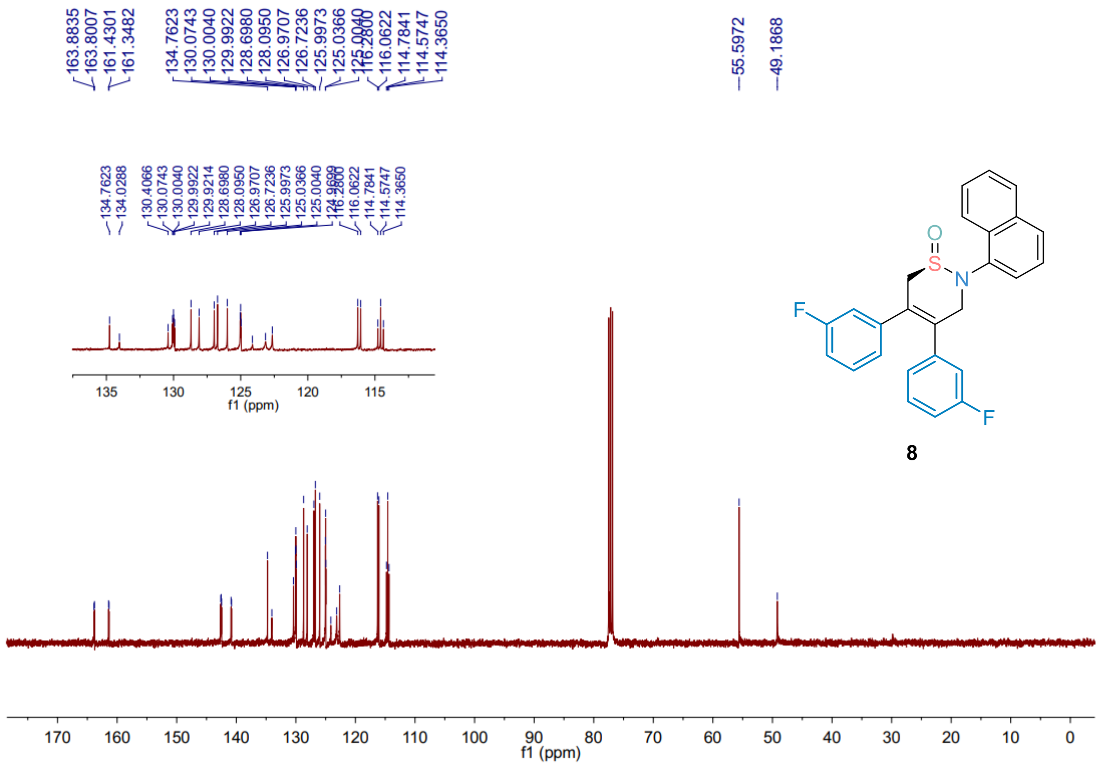

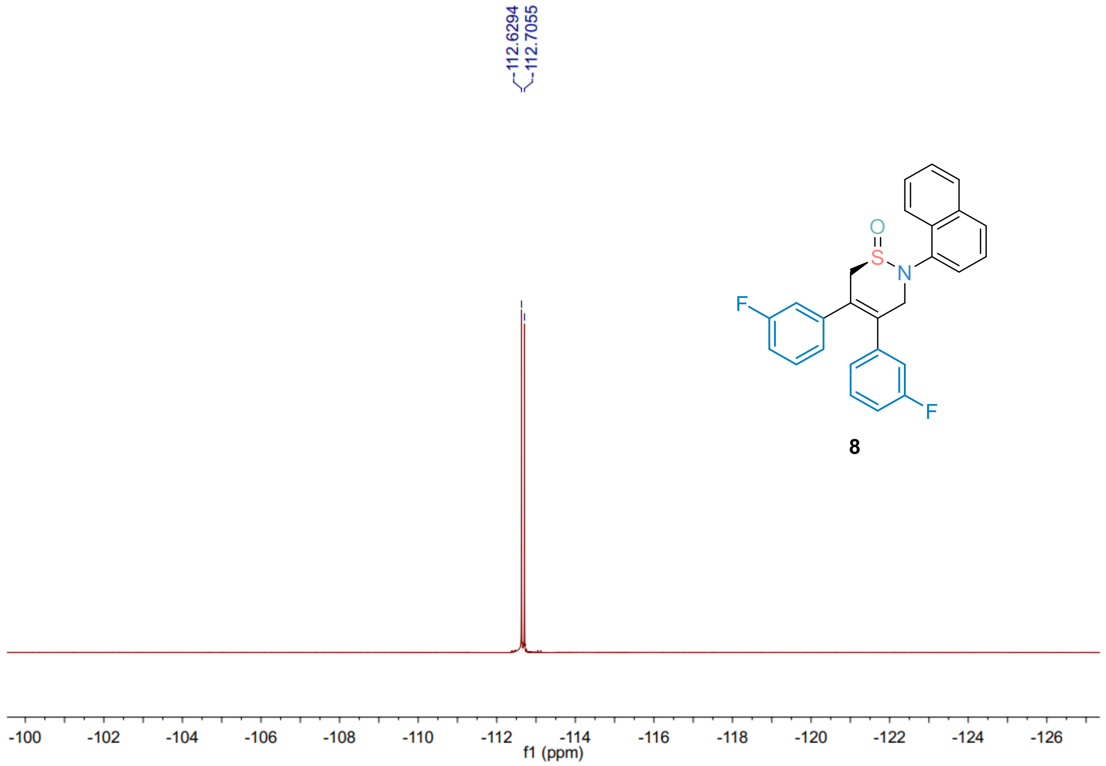

^{1}H-NMR and ^{13}C-NMR and ^{19}F-NMR of **8**

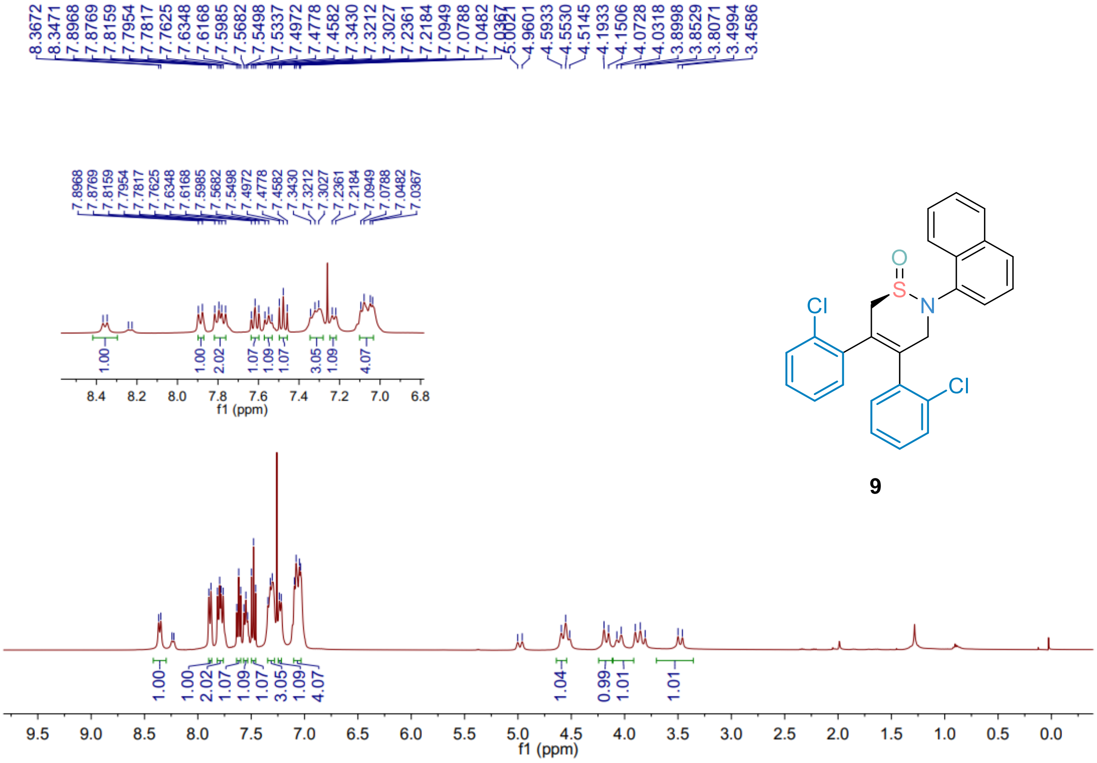

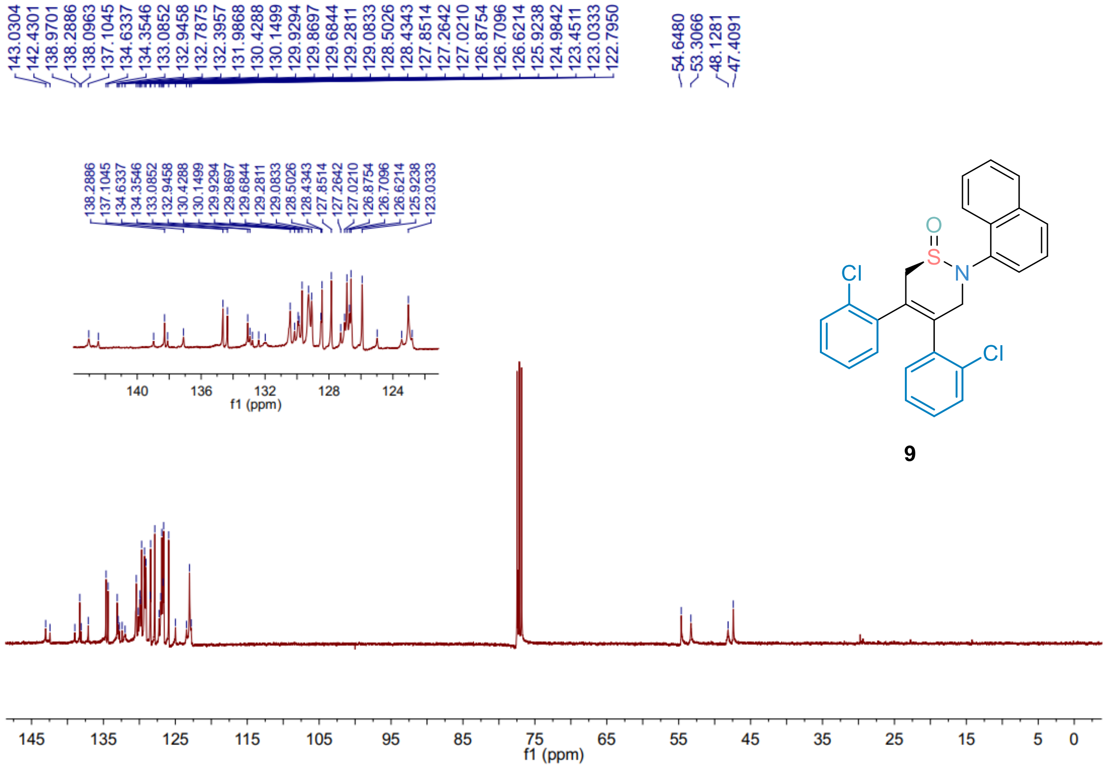

^{1}H-NMR and ^{13}C-NMR of **9**

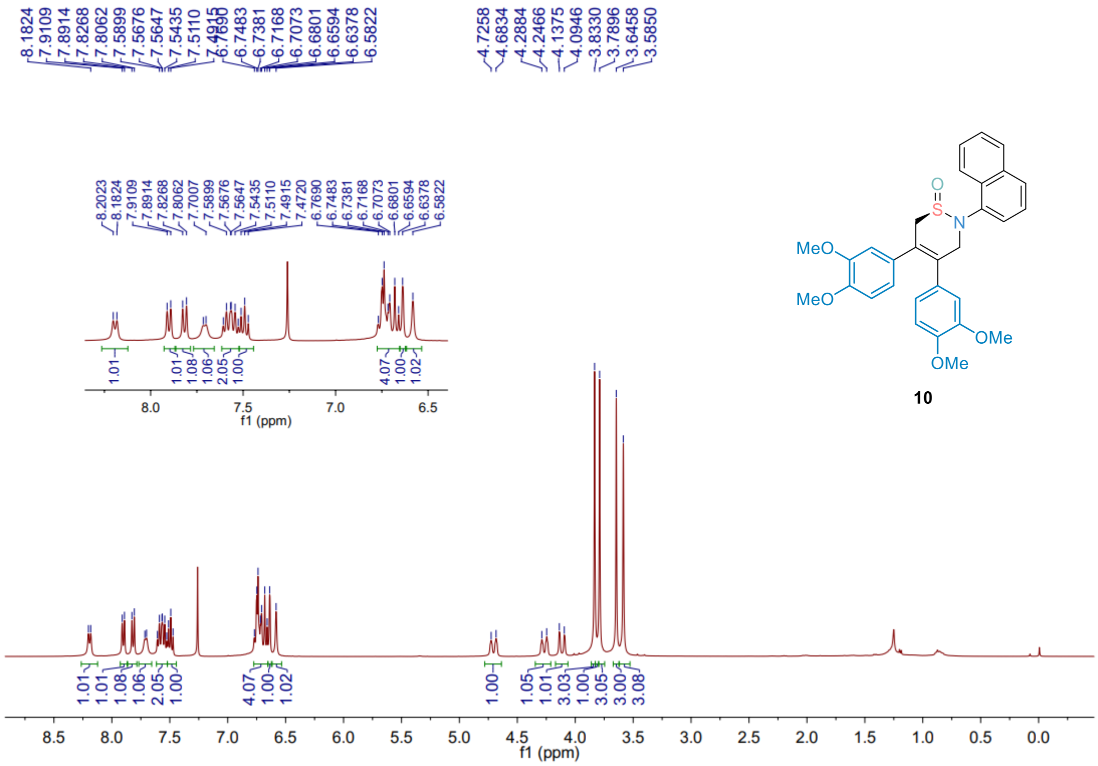

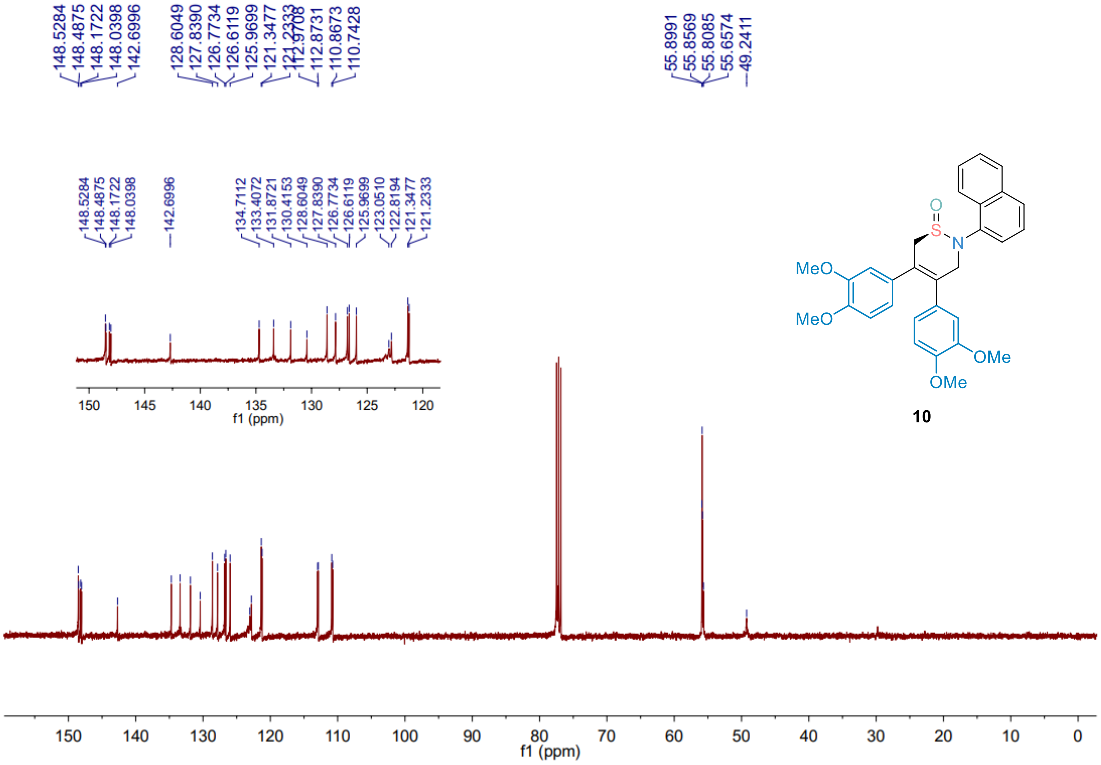

^{1}H-NMR and ^{13}C-NMR of **10**

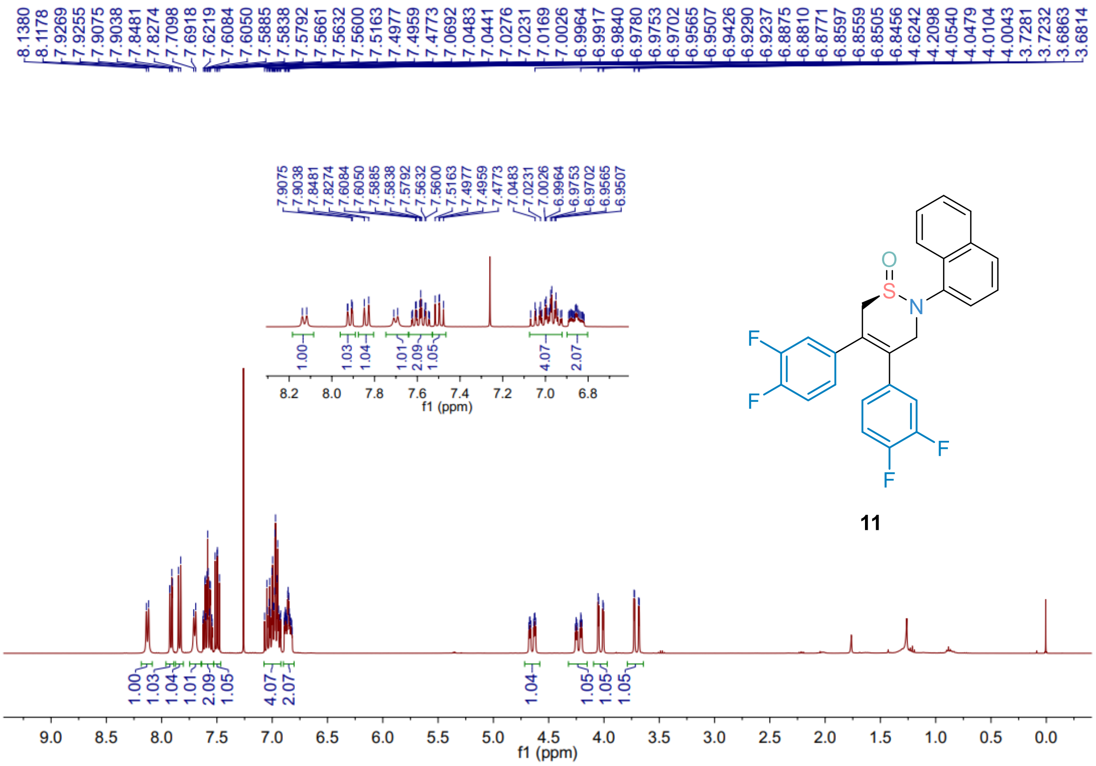

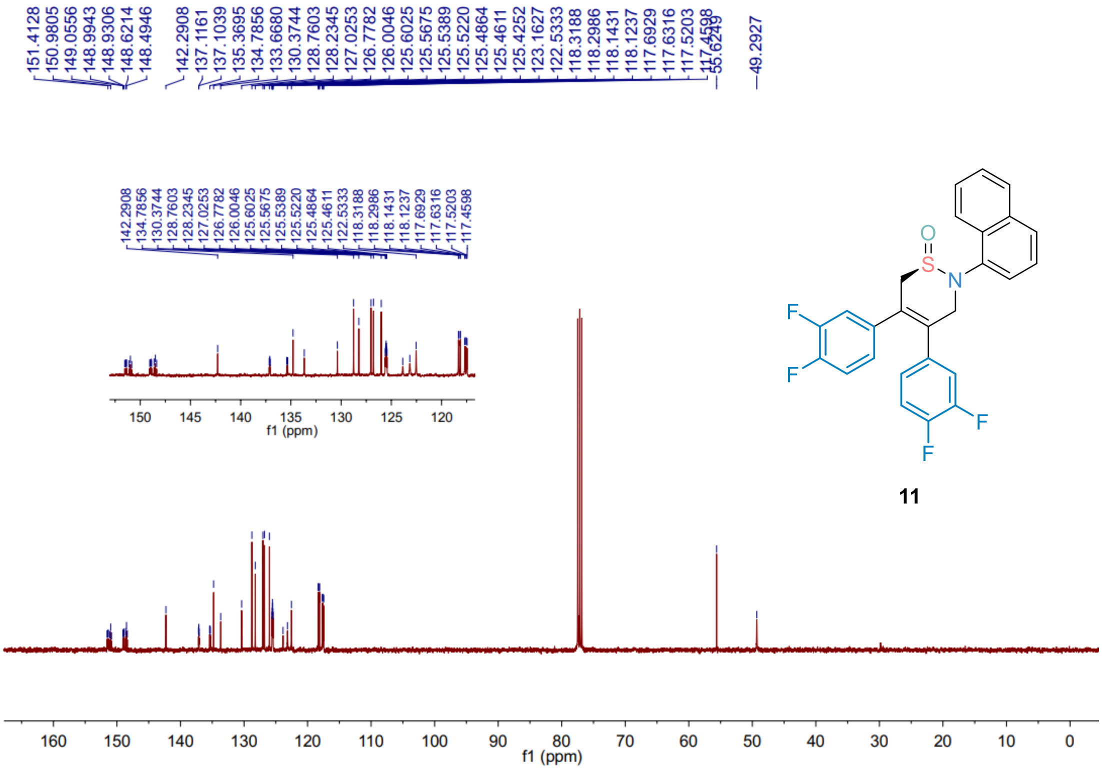

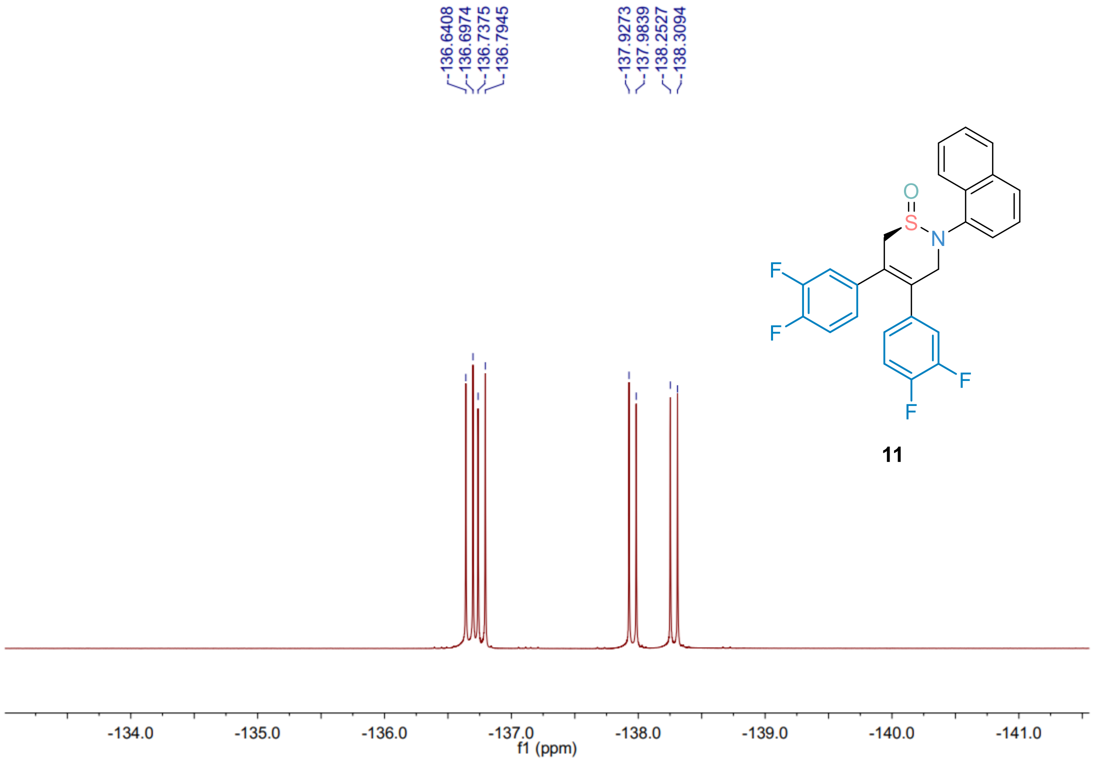

^{1}H-NMR and ^{13}C-NMR and ^{19}F-NMR of **11**

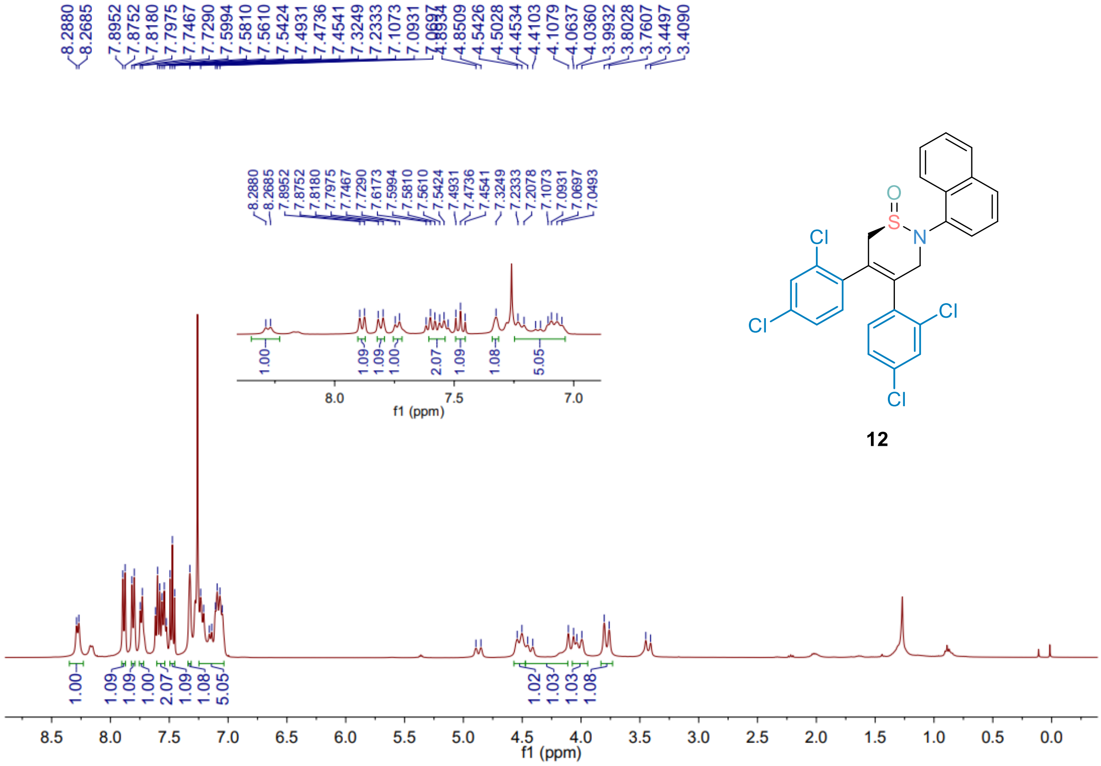

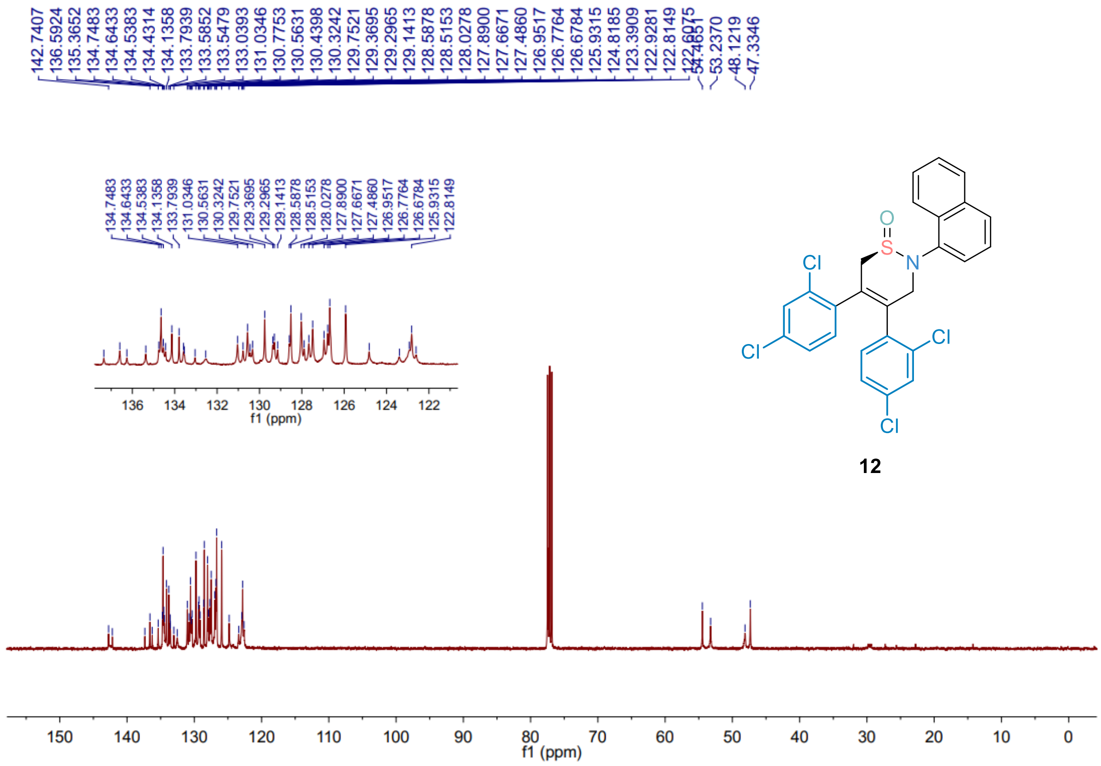

^{1}H-NMR and ^{13}C-NMR of **12**

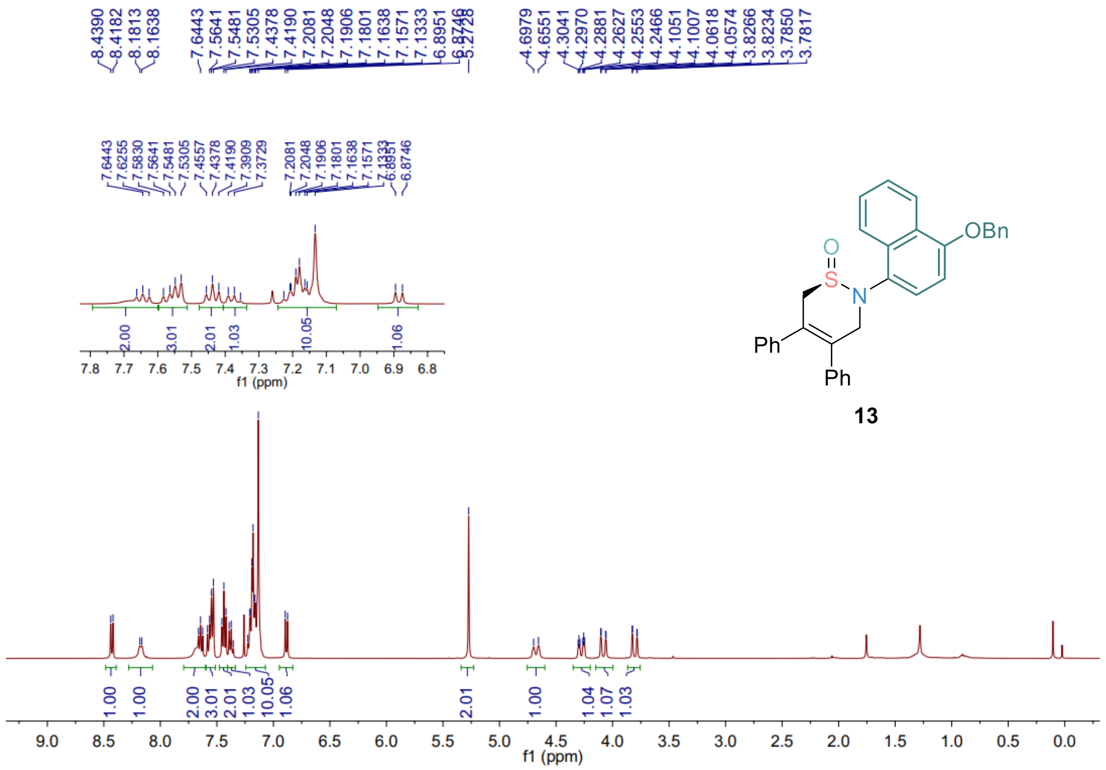

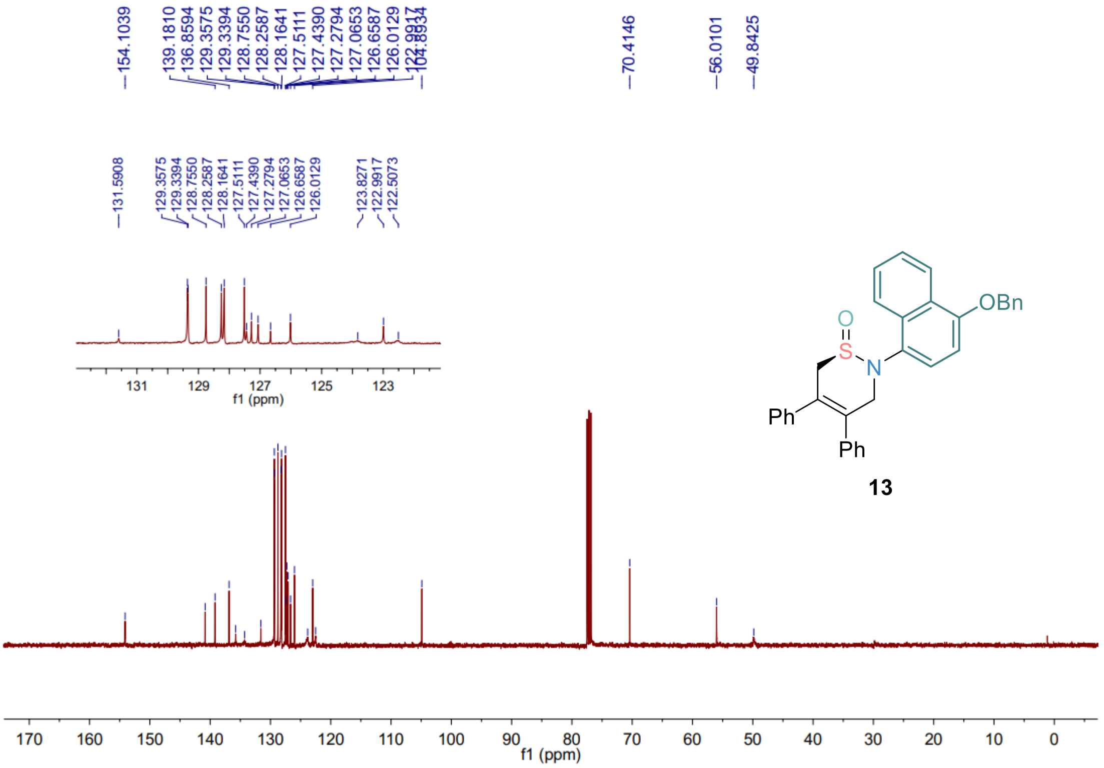

^{1}H-NMR and ^{13}C-NMR of **13**

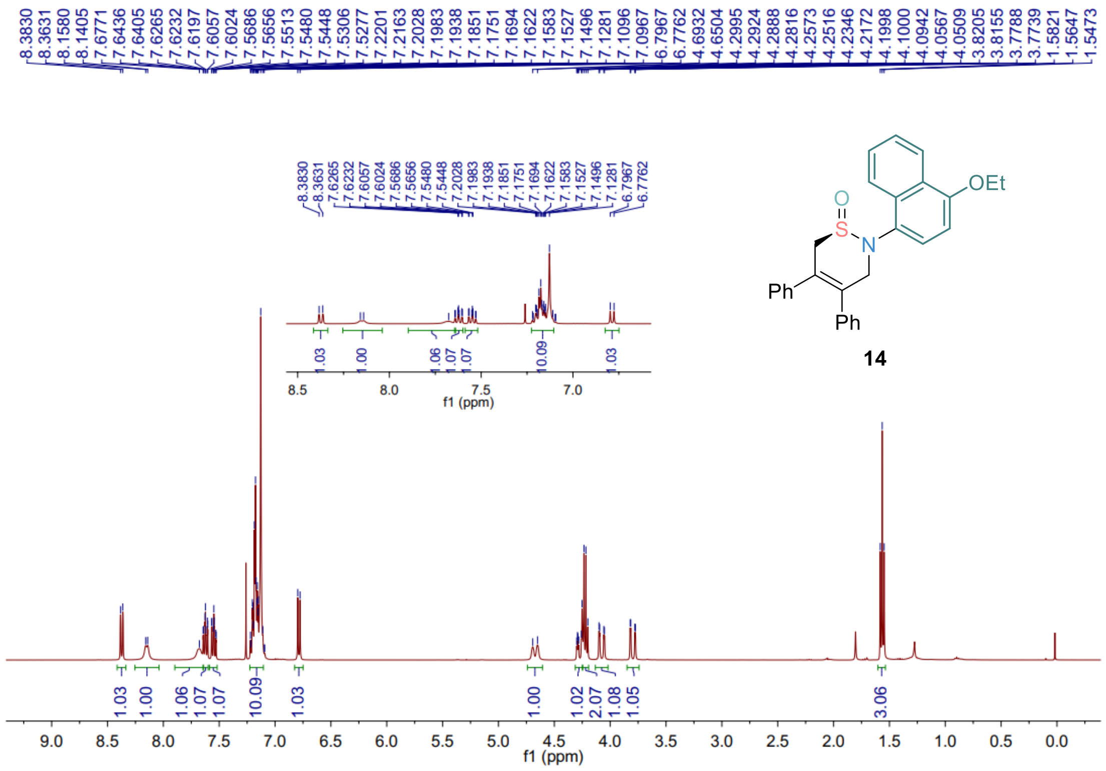

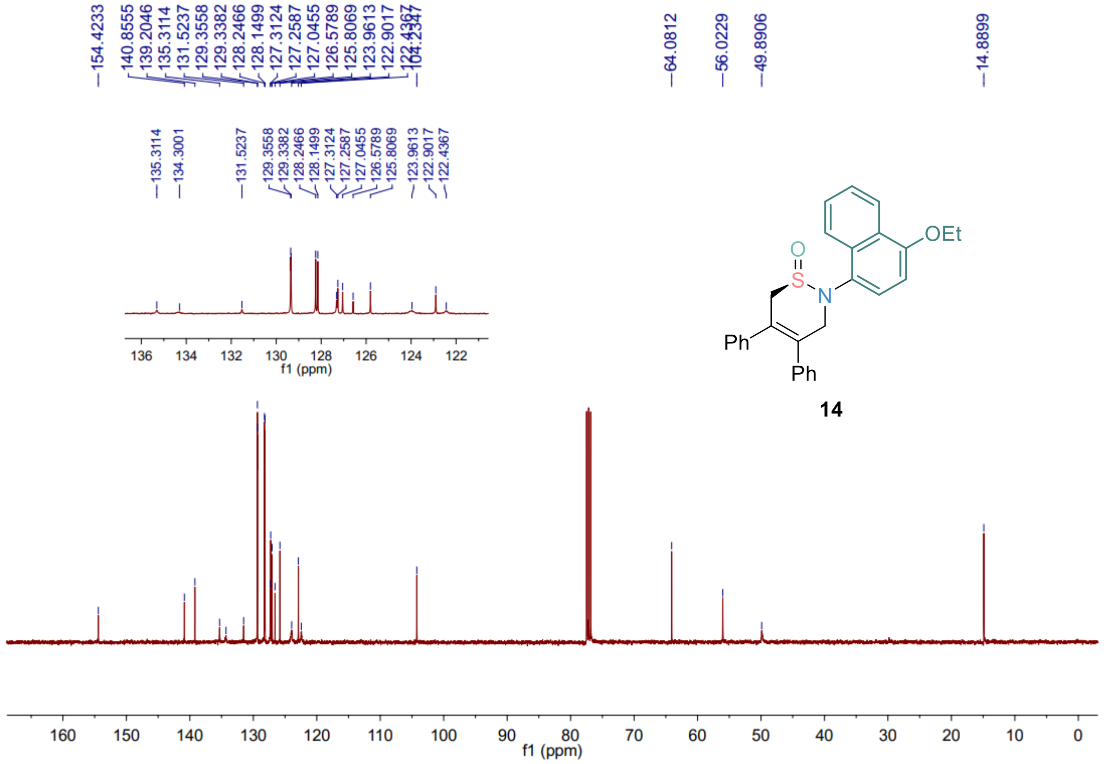

14

^{1}H-NMR and ^{13}C-NMR of **14**

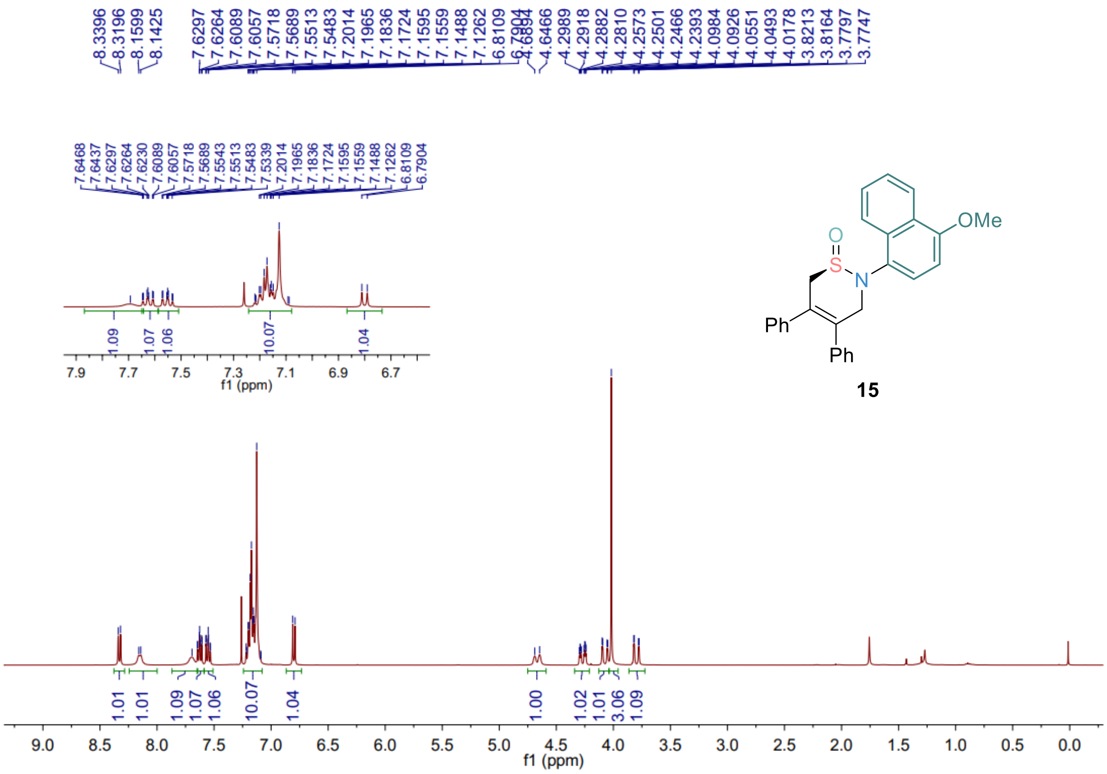

15

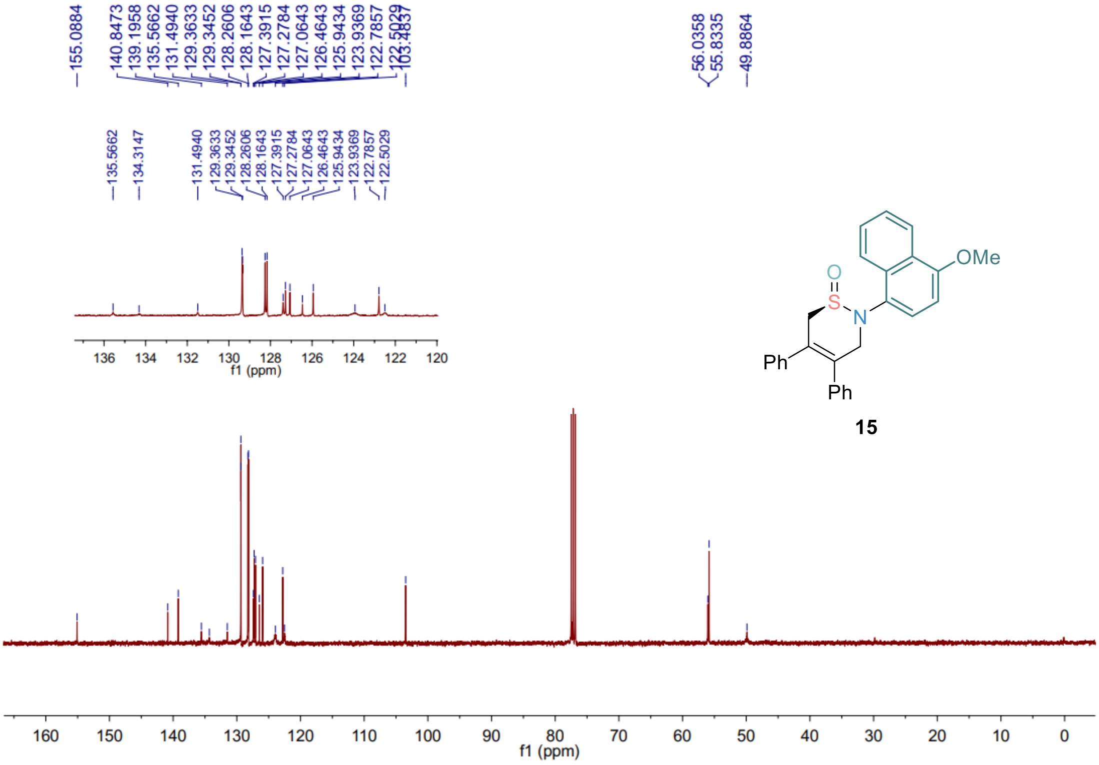

^{1}H-NMR and ^{13}C-NMR of **15**

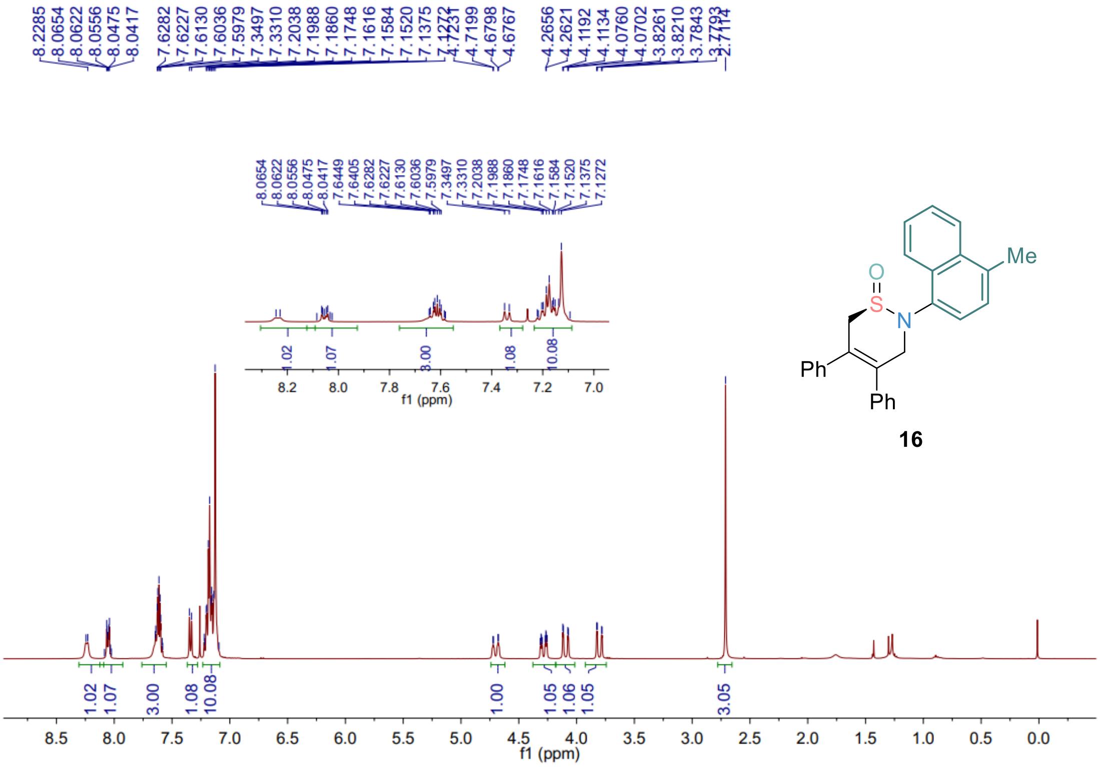

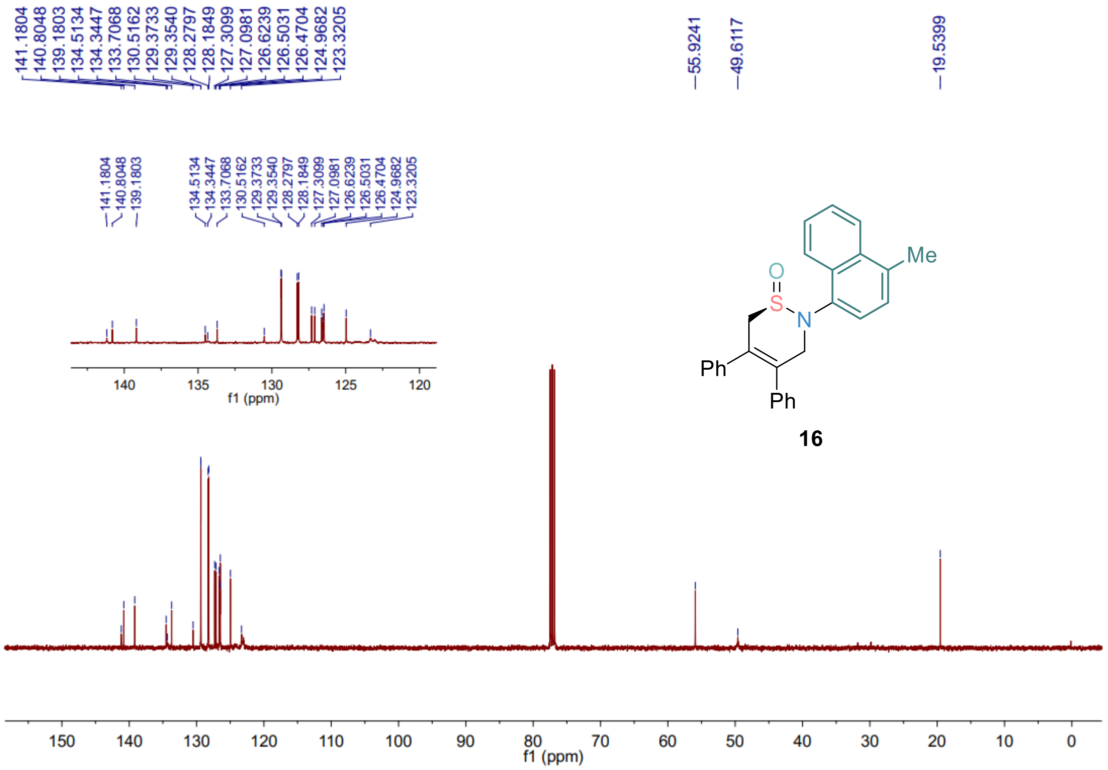

^{1}H-NMR and ^{13}C-NMR of **16**

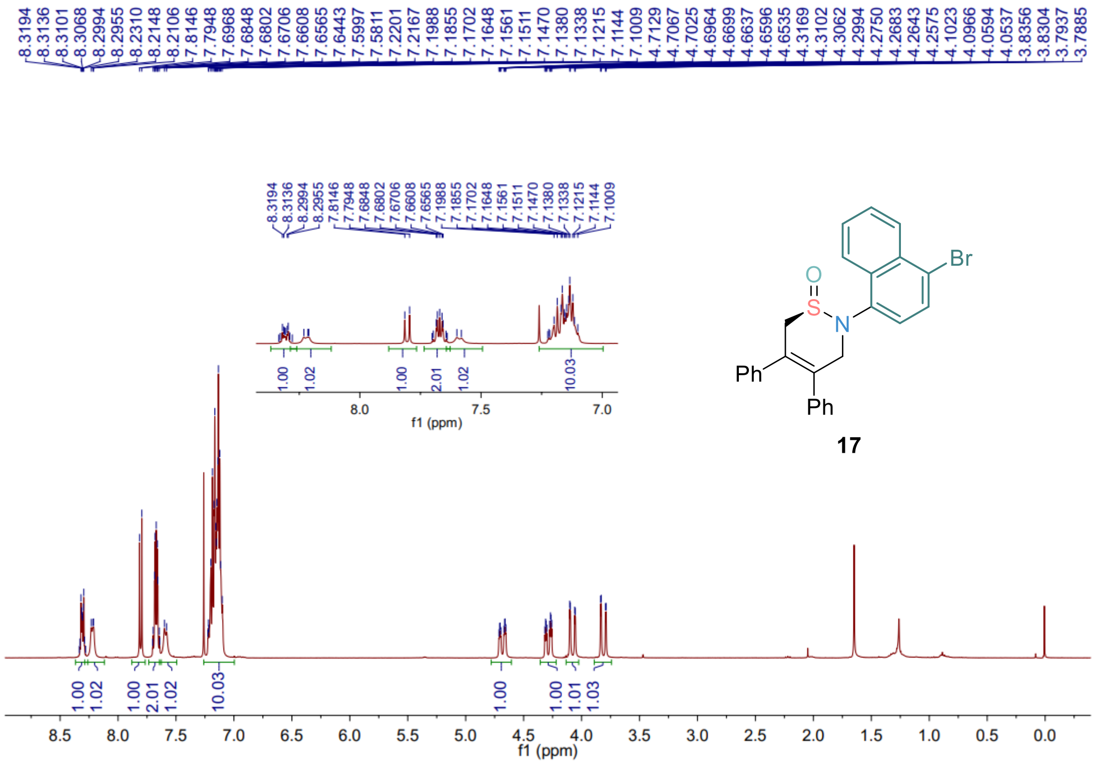

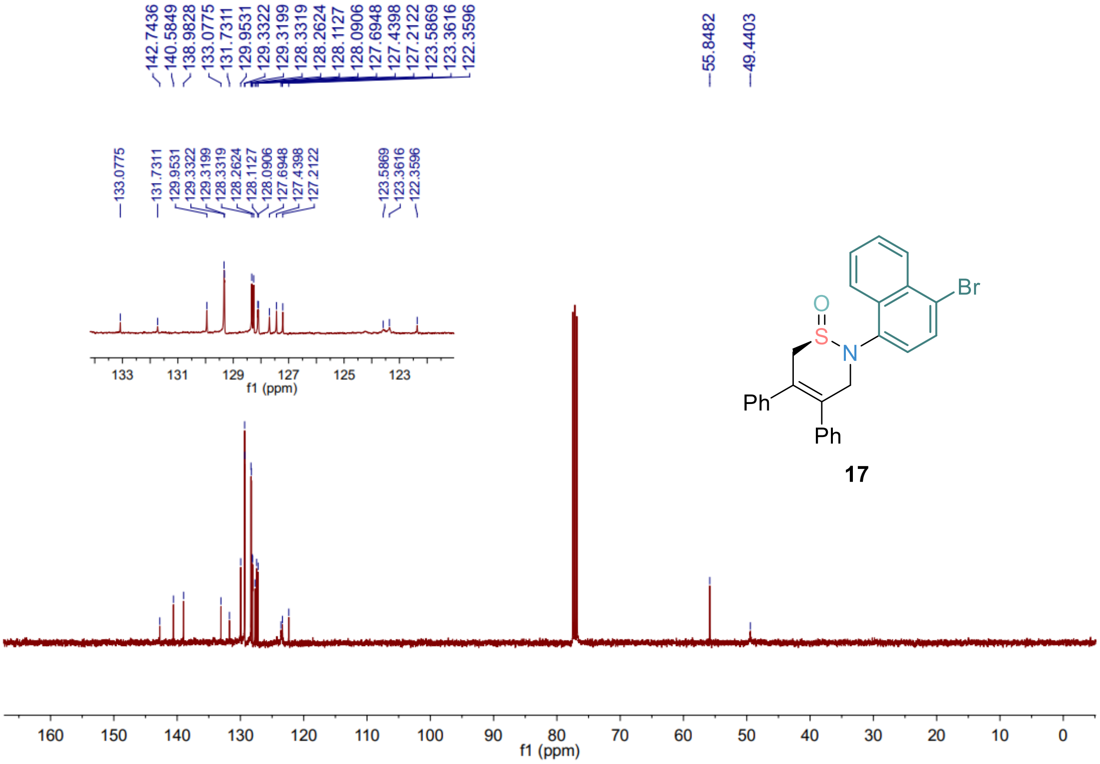

^{1}H-NMR and ^{13}C-NMR of **17**

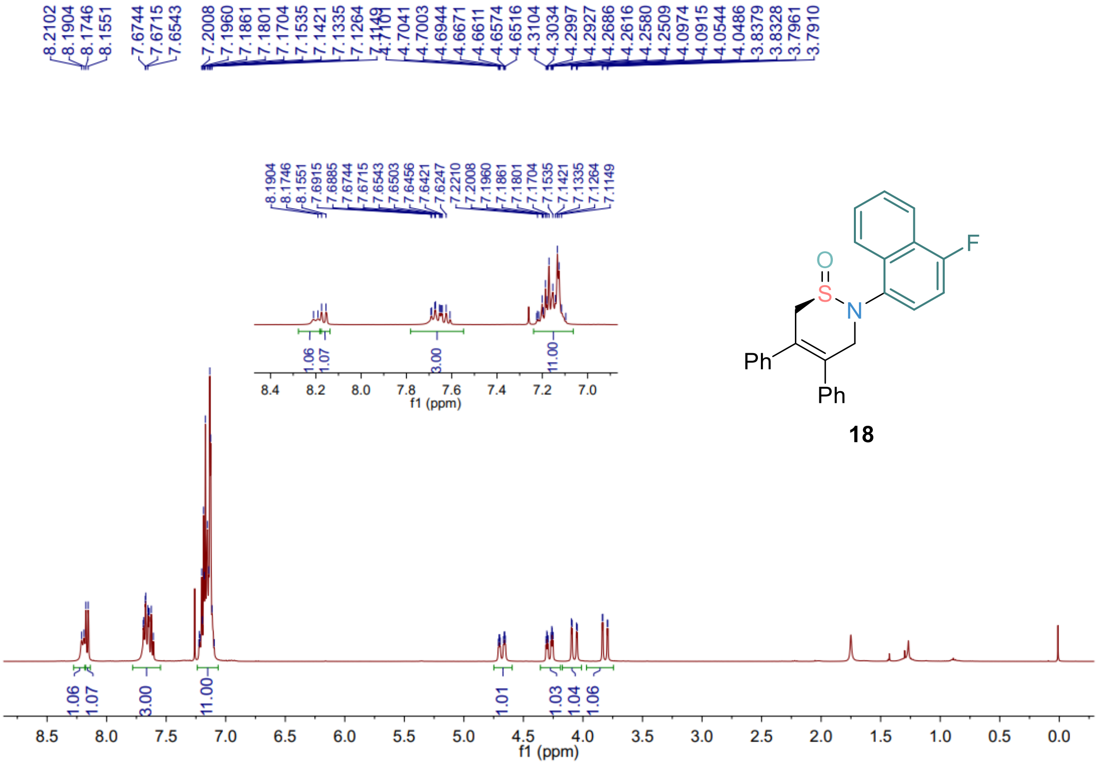

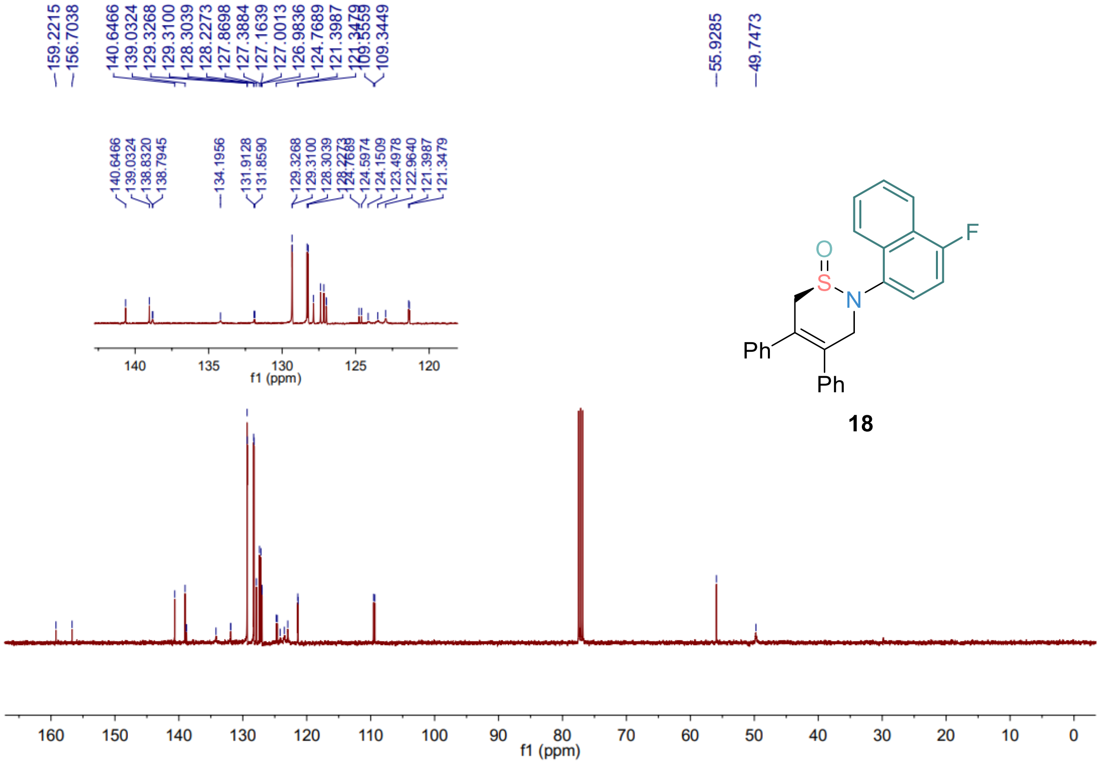

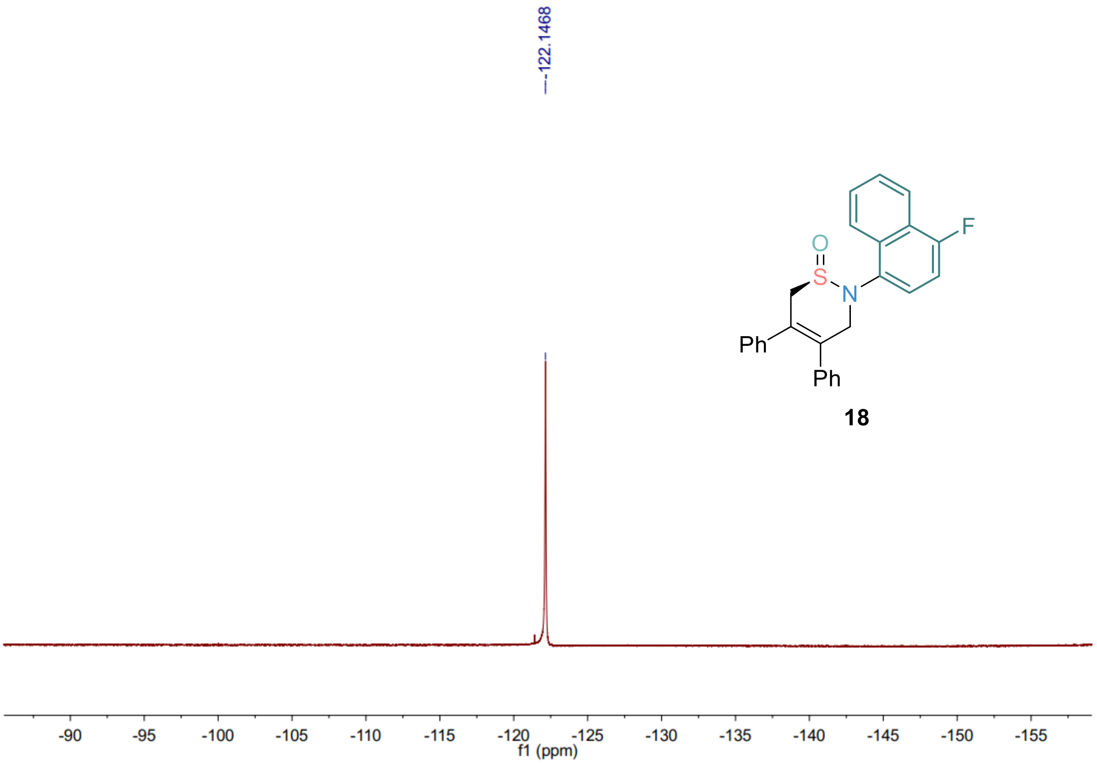

^{1}H-NMR and ^{13}C-NMR and ^{19}F-NMR of **18**

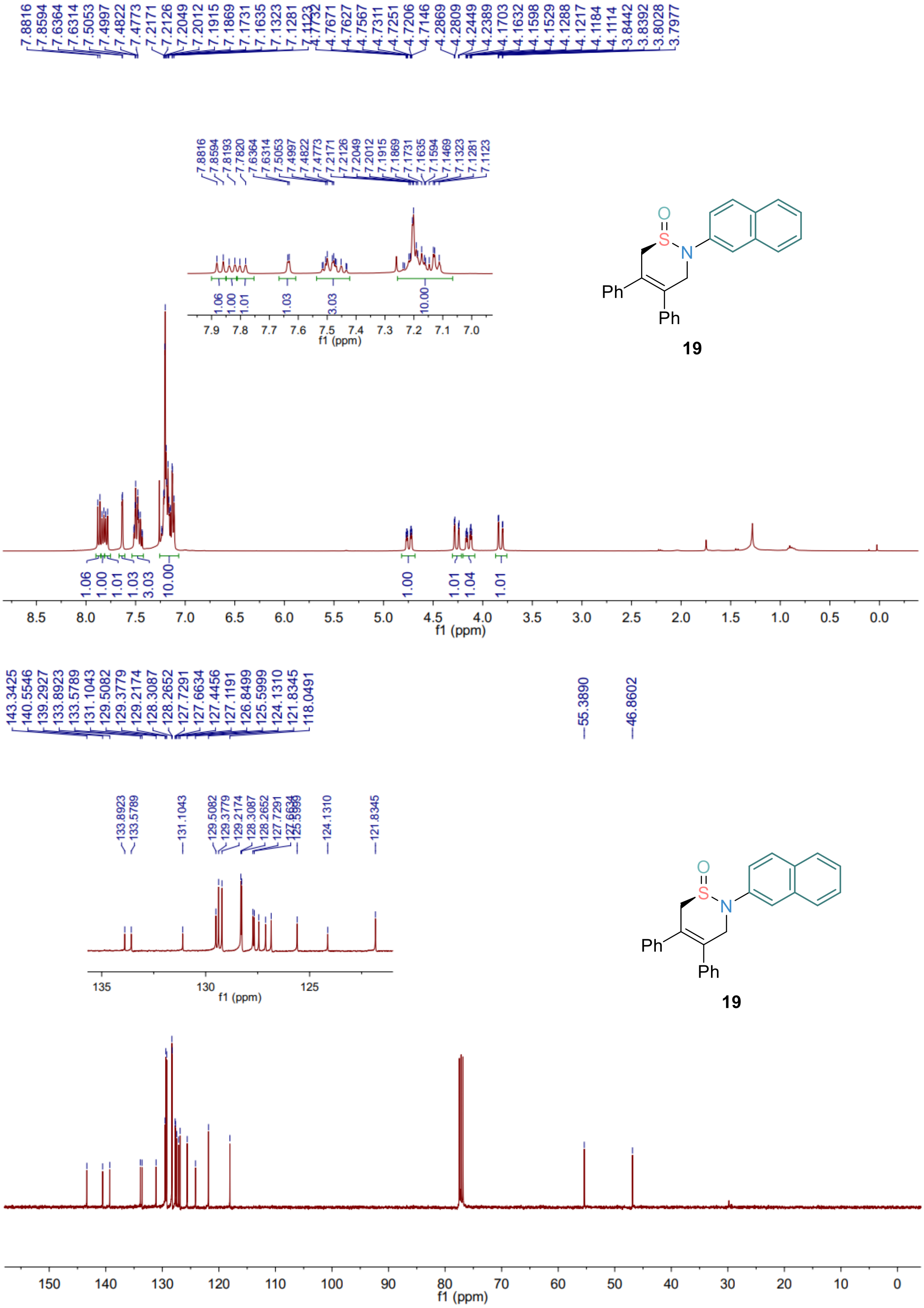

^{1}H-NMR and ^{13}C-NMR of **19**

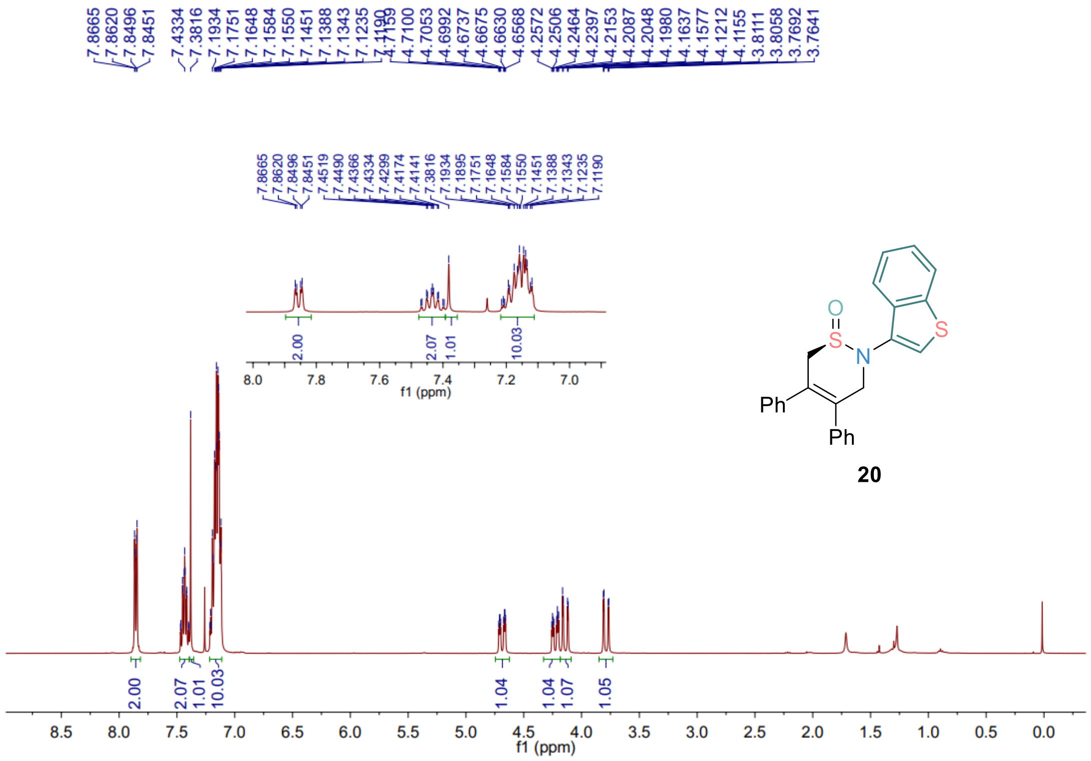

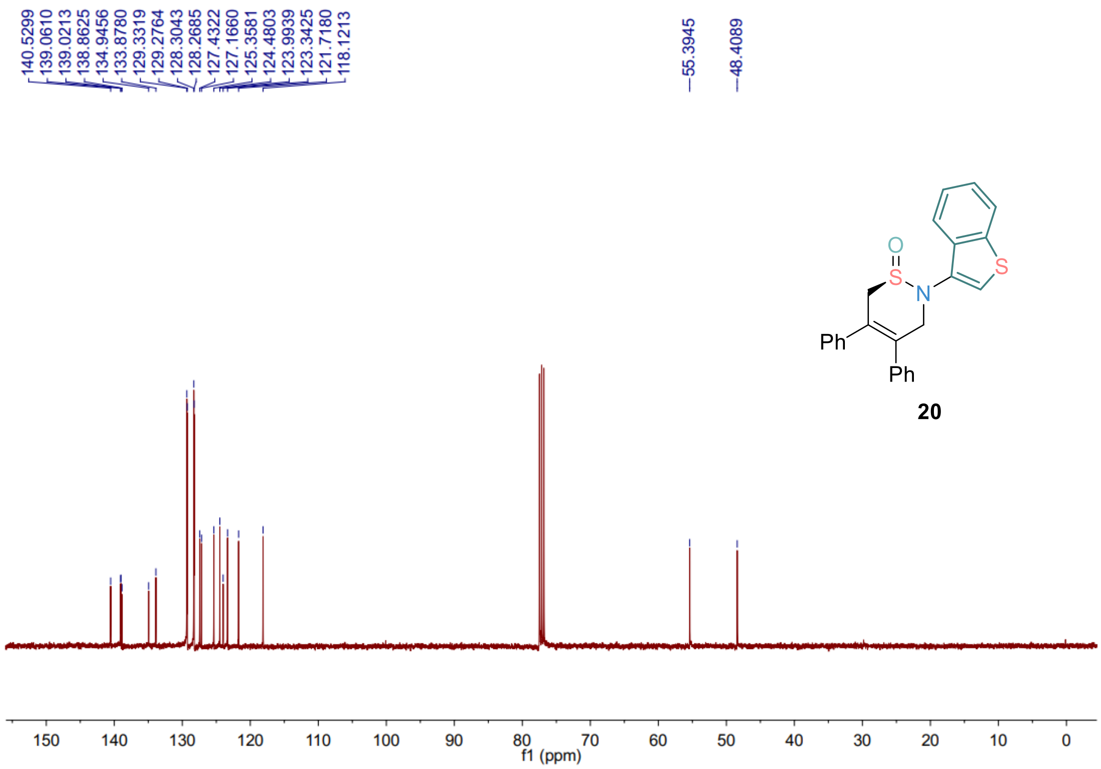

^{1}H-NMR and ^{13}C-NMR of **20**

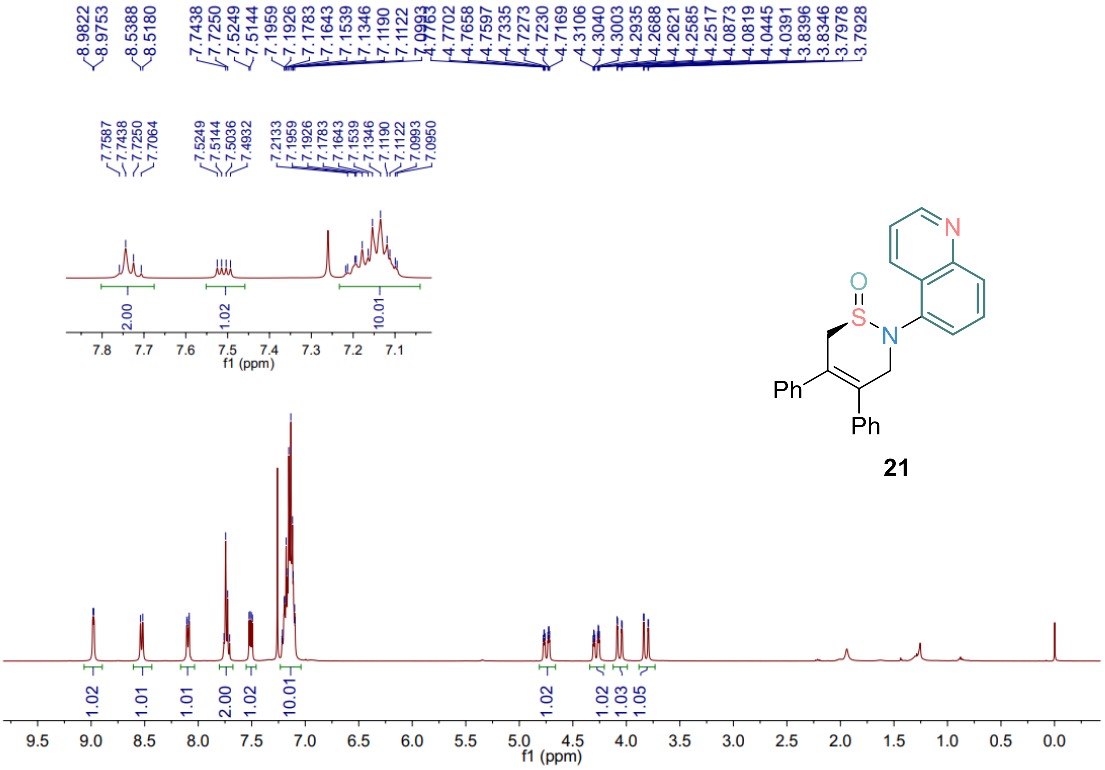

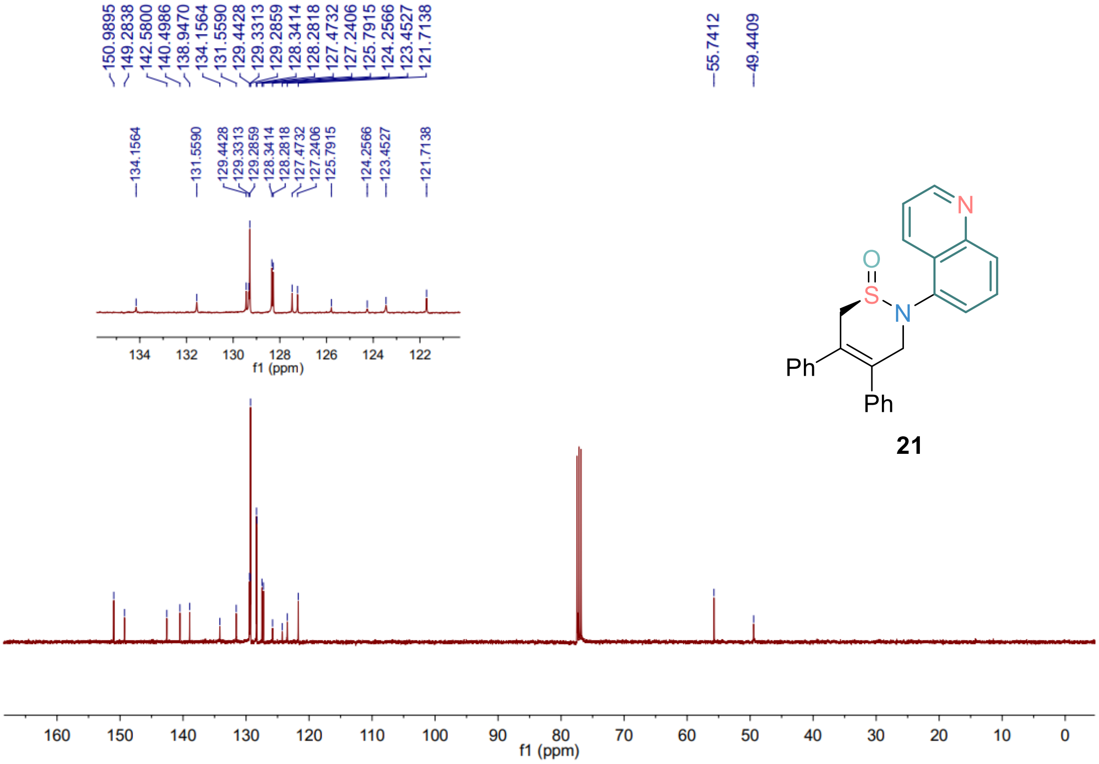

^{1}H-NMR and ^{13}C-NMR of **21**

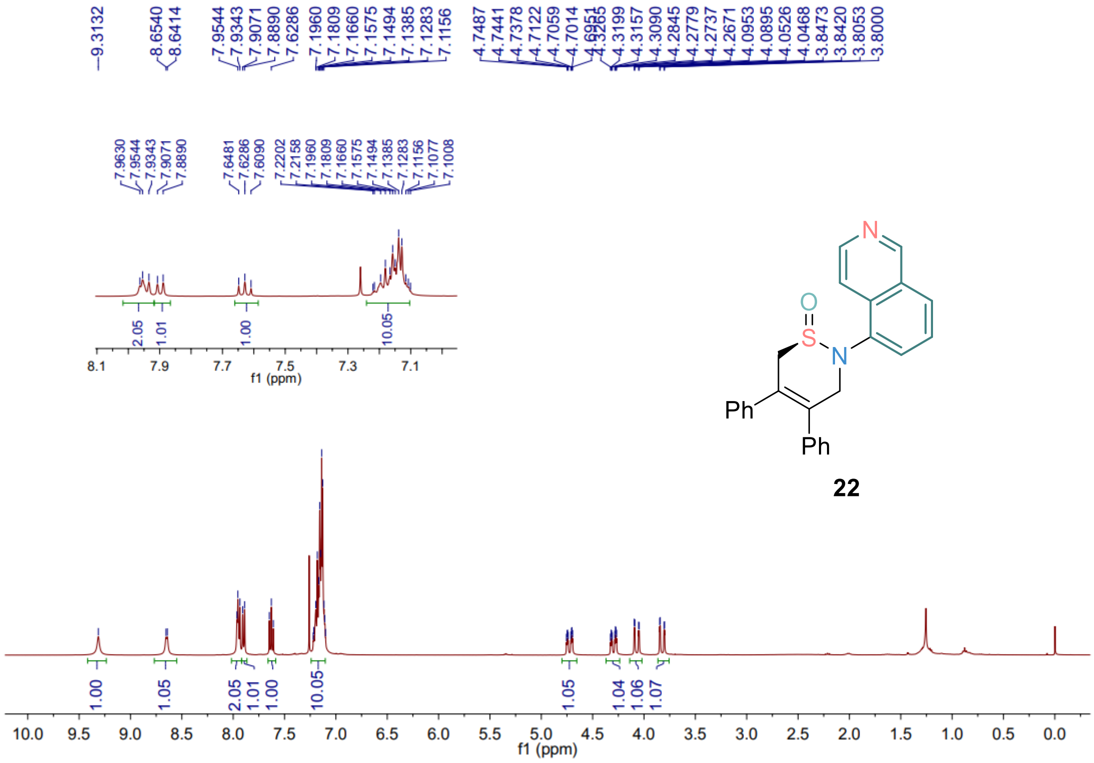

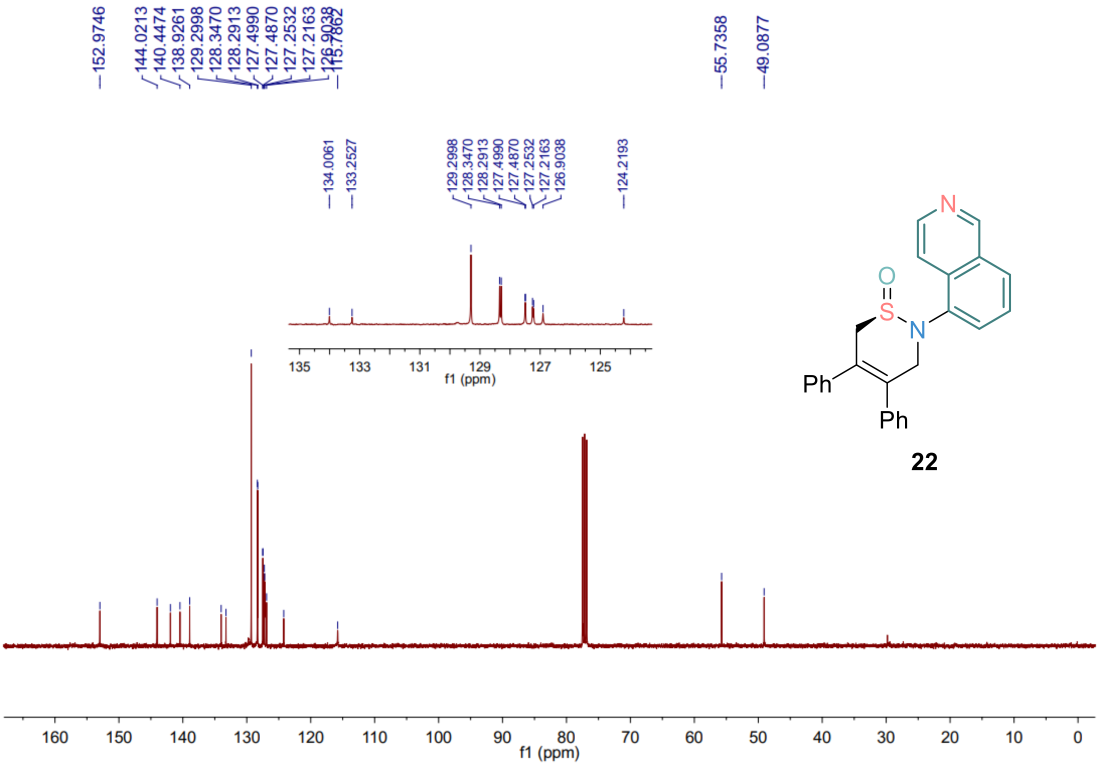

^{1}H-NMR and ^{13}C-NMR of **22**

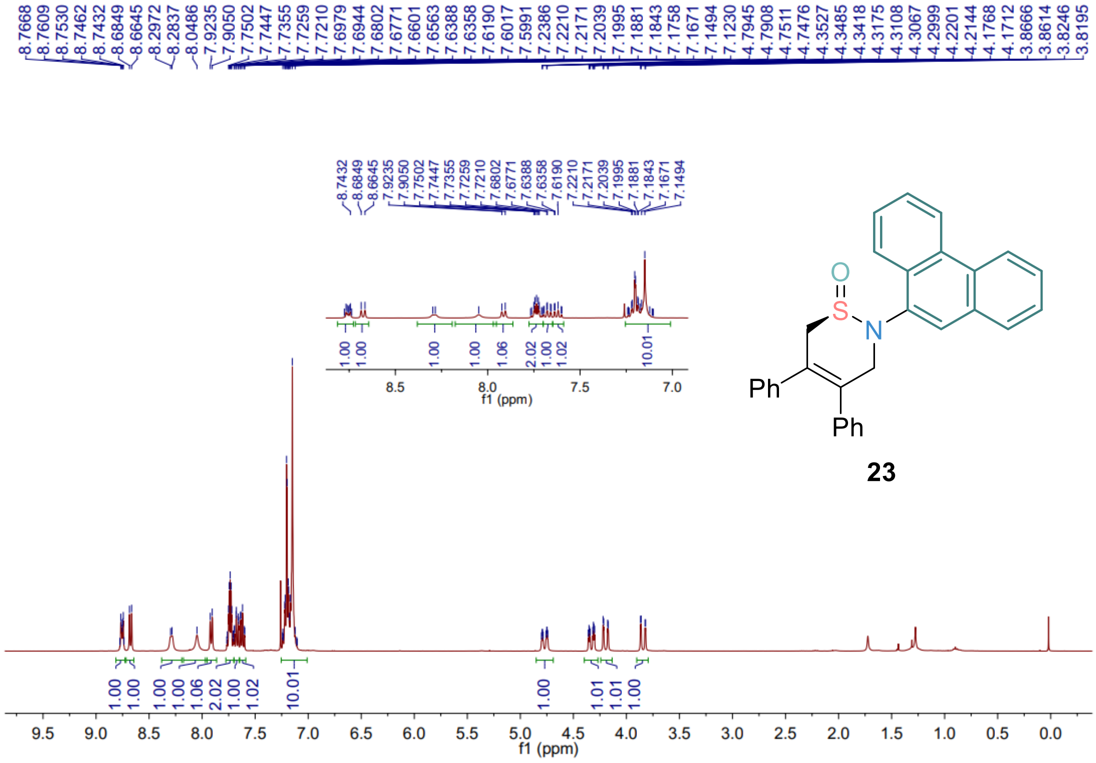

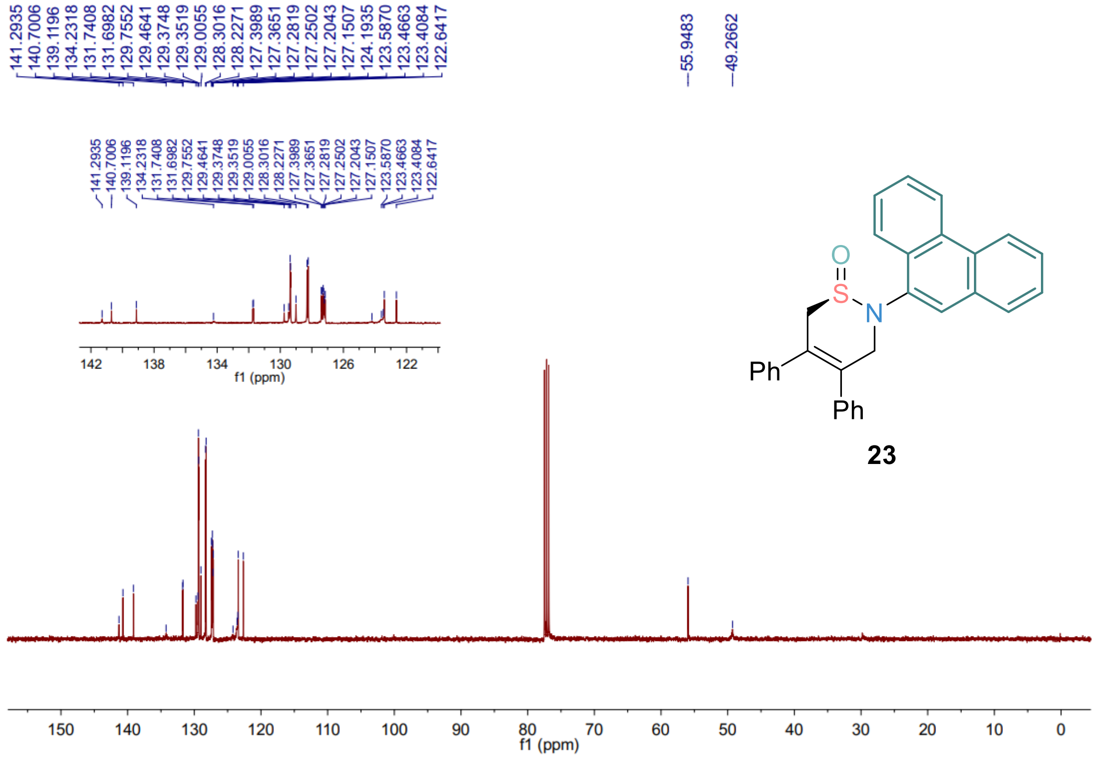

^{1}H-NMR and ^{13}C-NMR of **23**

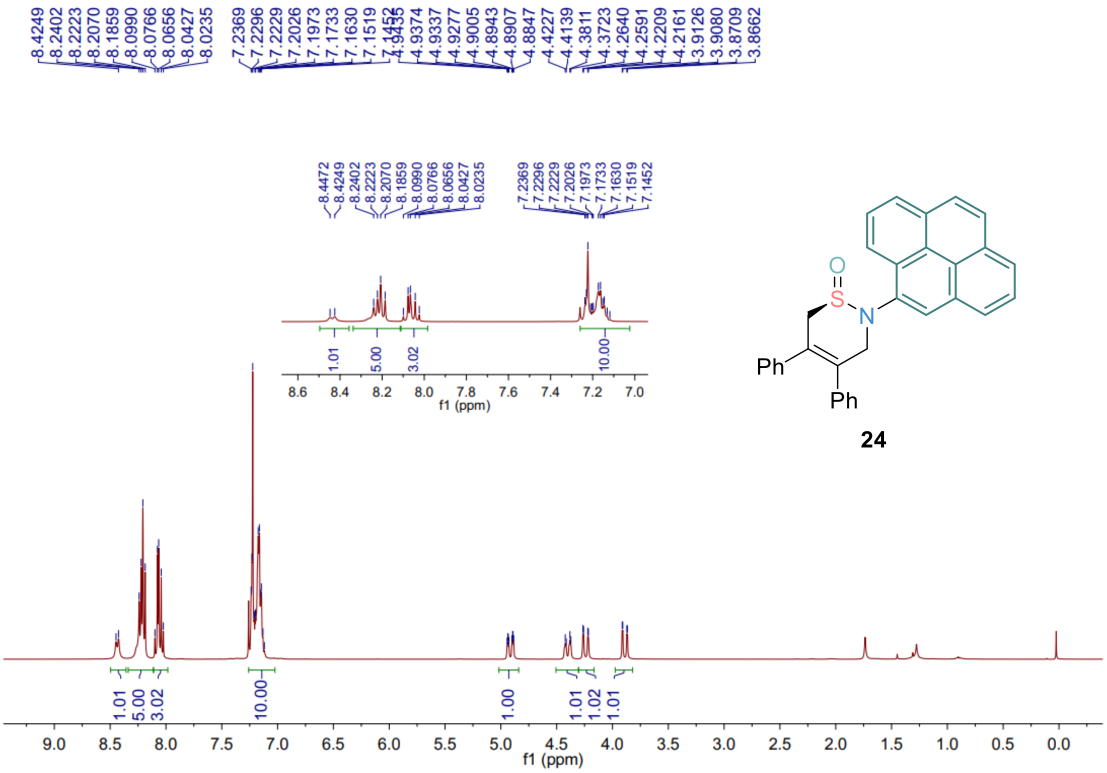

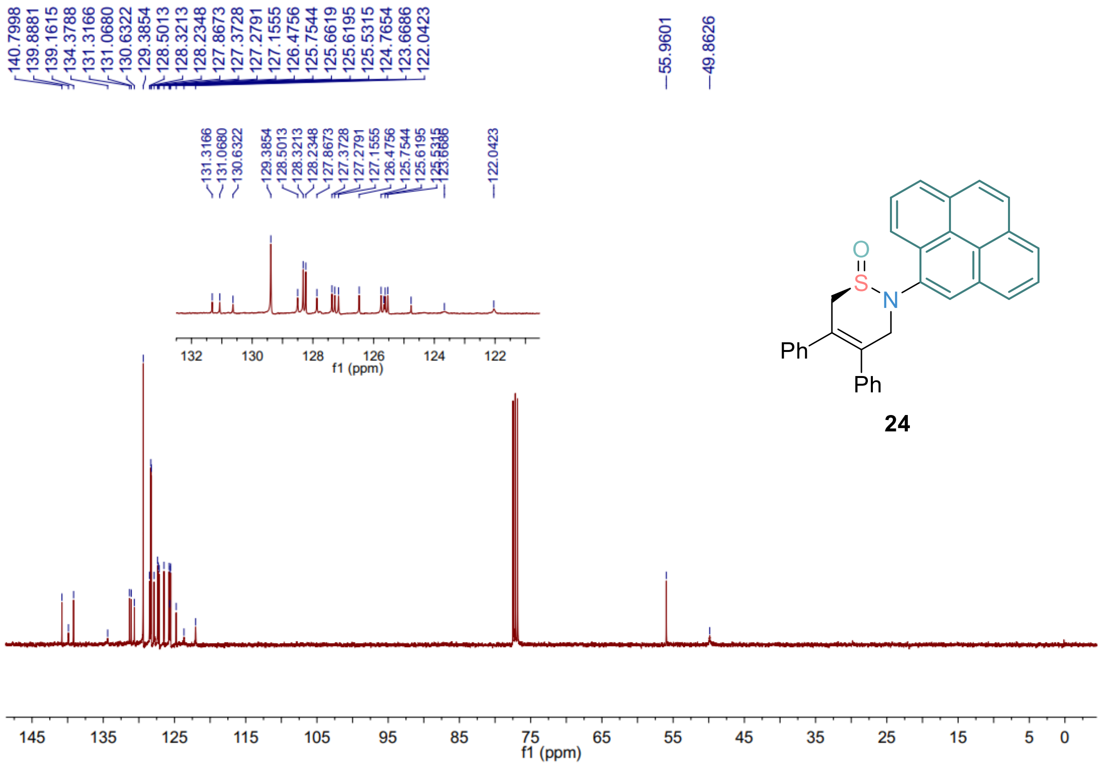

^{1}H-NMR and ^{13}C-NMR of **24**

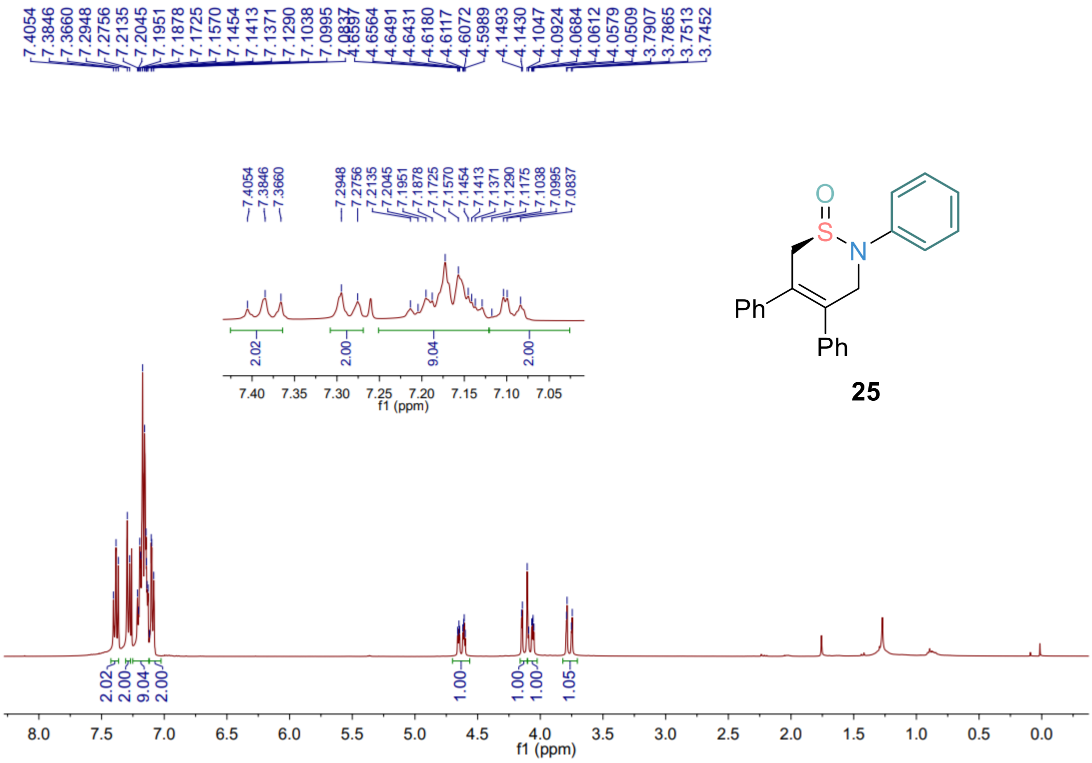

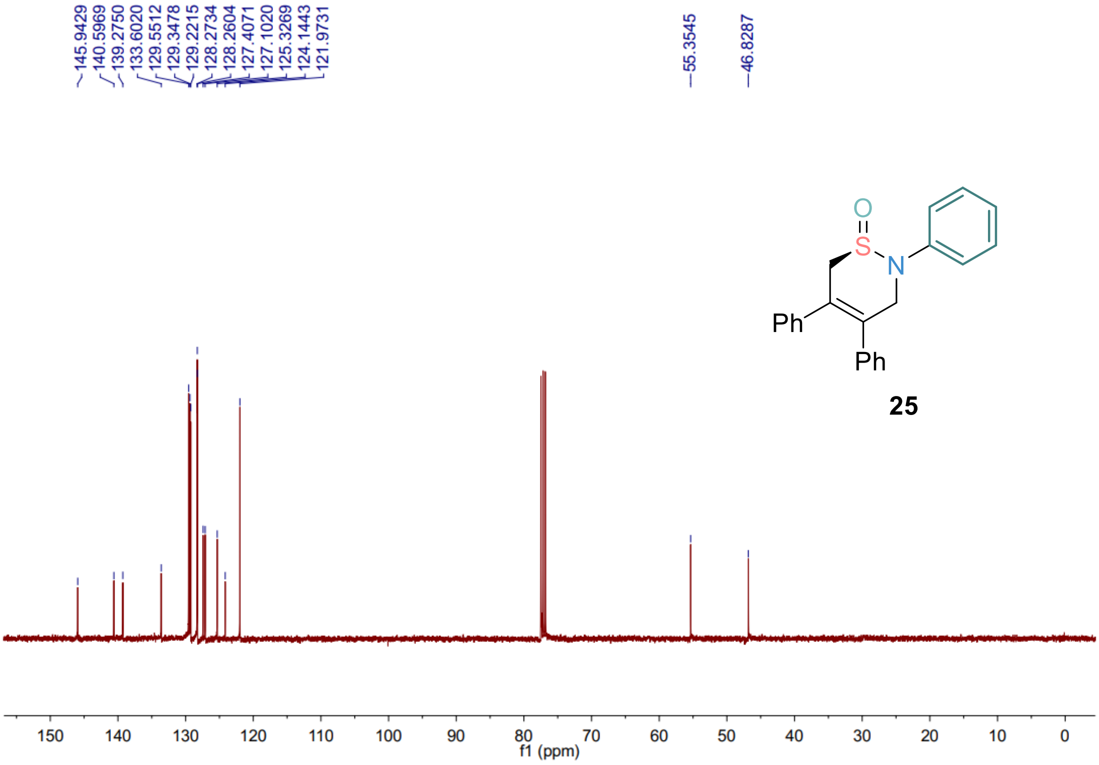

^{1}H-NMR and ^{13}C-NMR of **25**

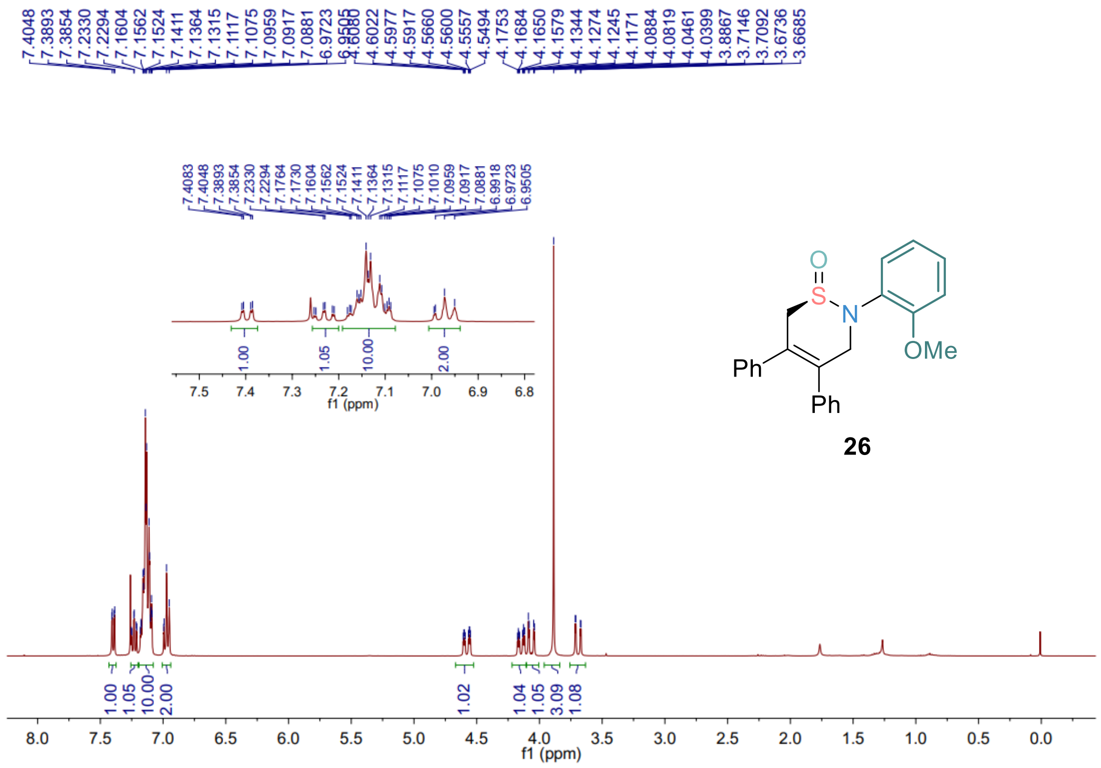

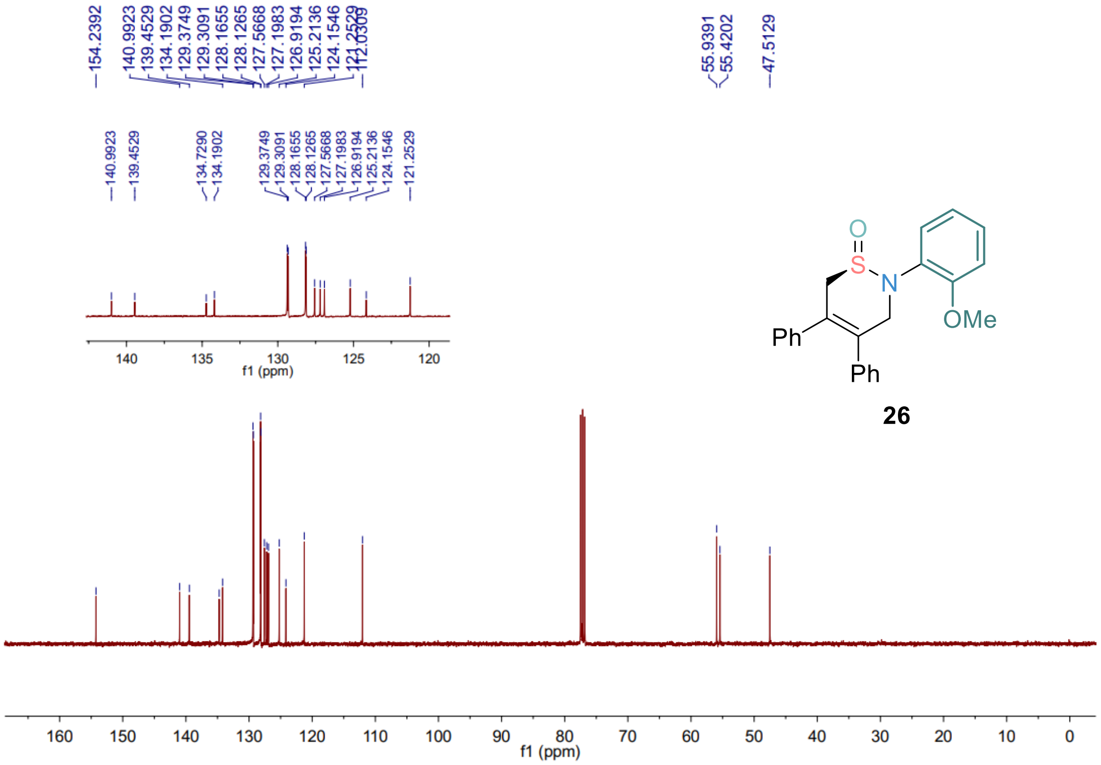

^{1}H-NMR and ^{13}C-NMR of **26**

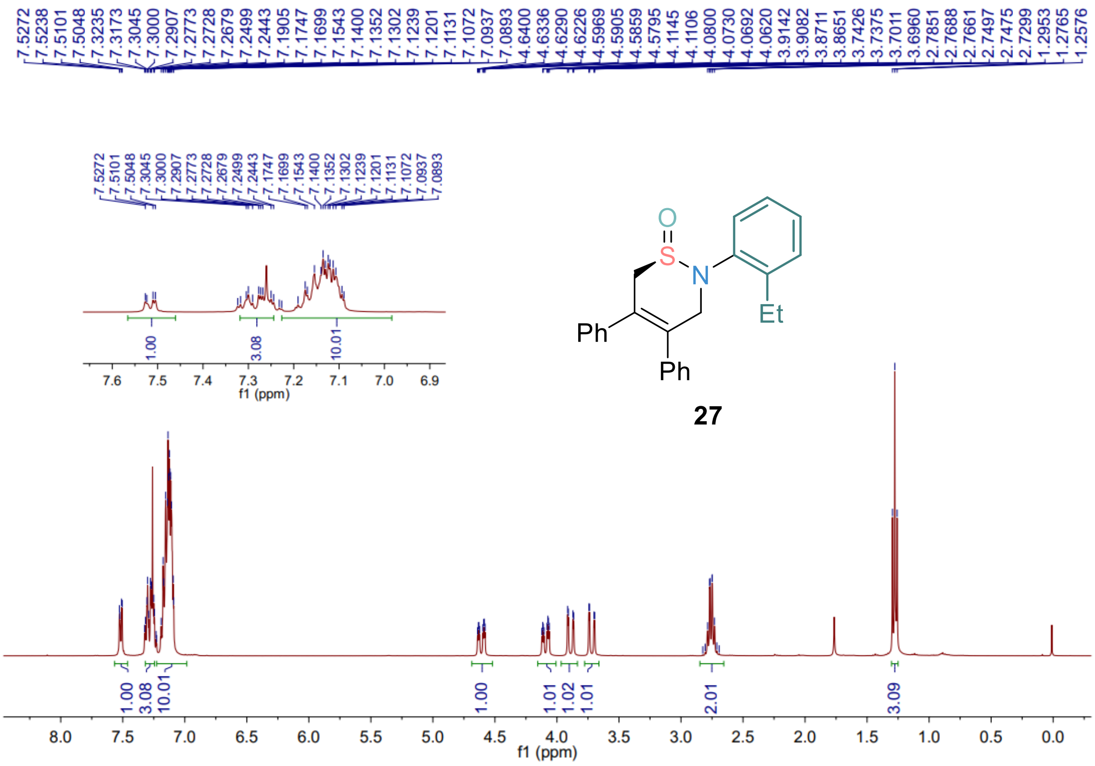

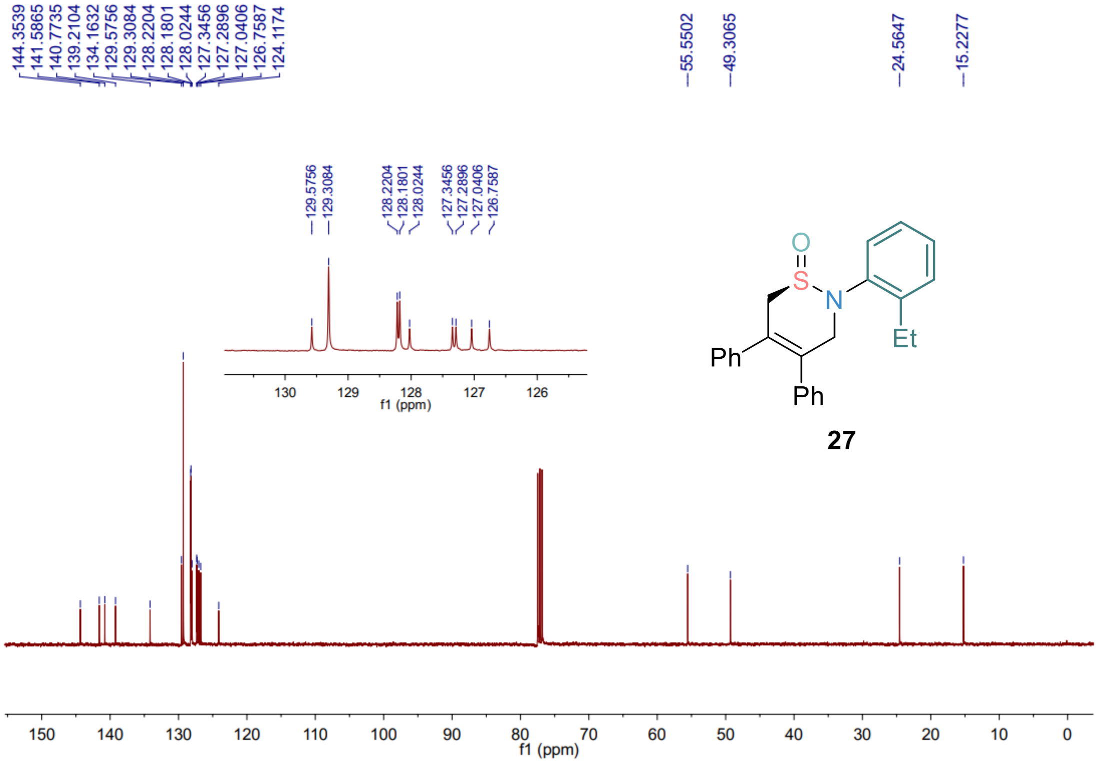

^{1}H-NMR and ^{13}C-NMR of **27**

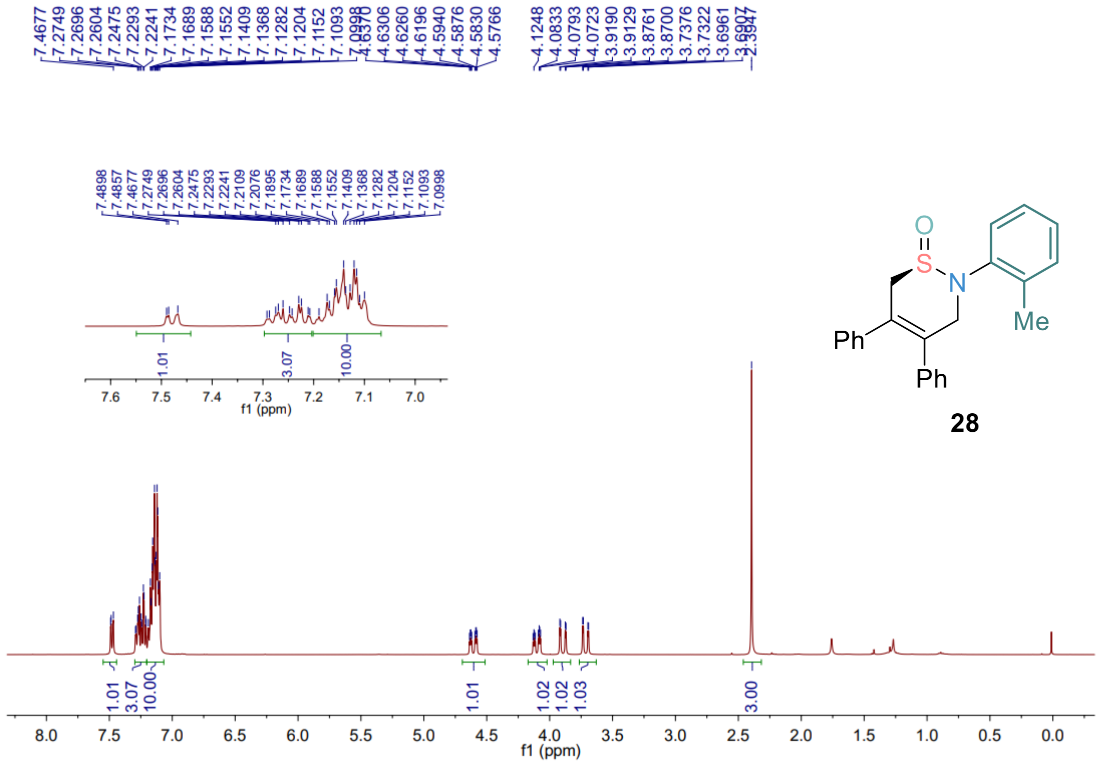

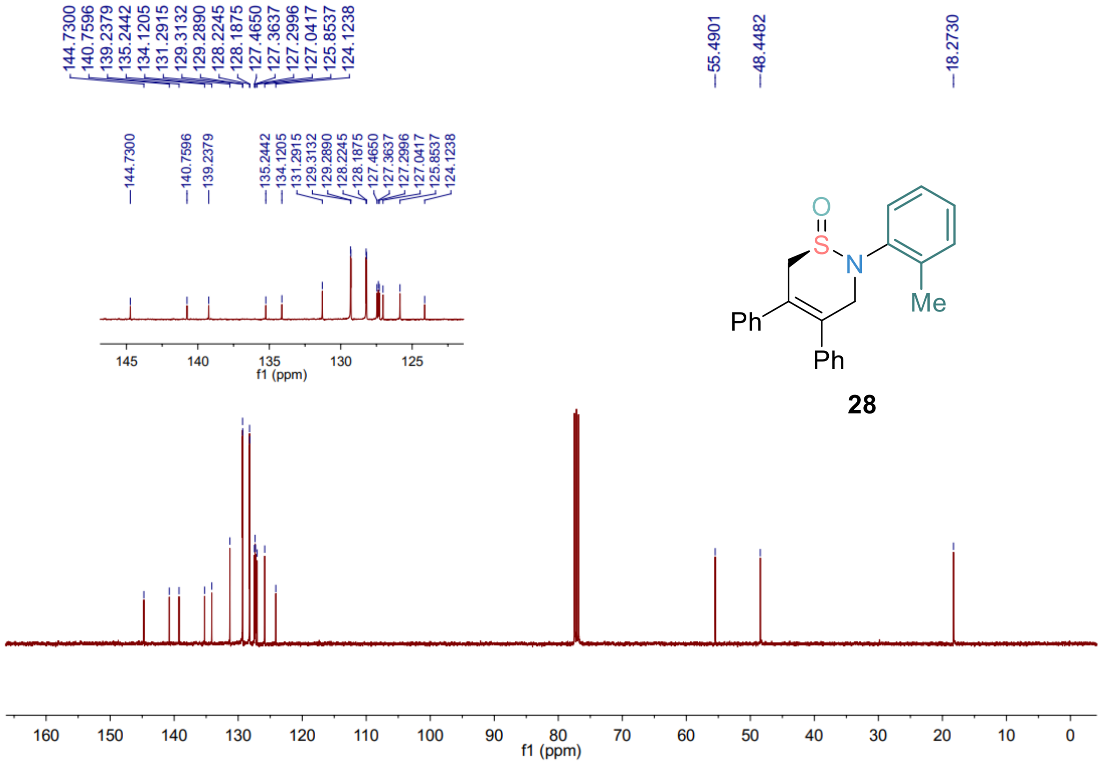

^{1}H-NMR and ^{13}C-NMR of **28**

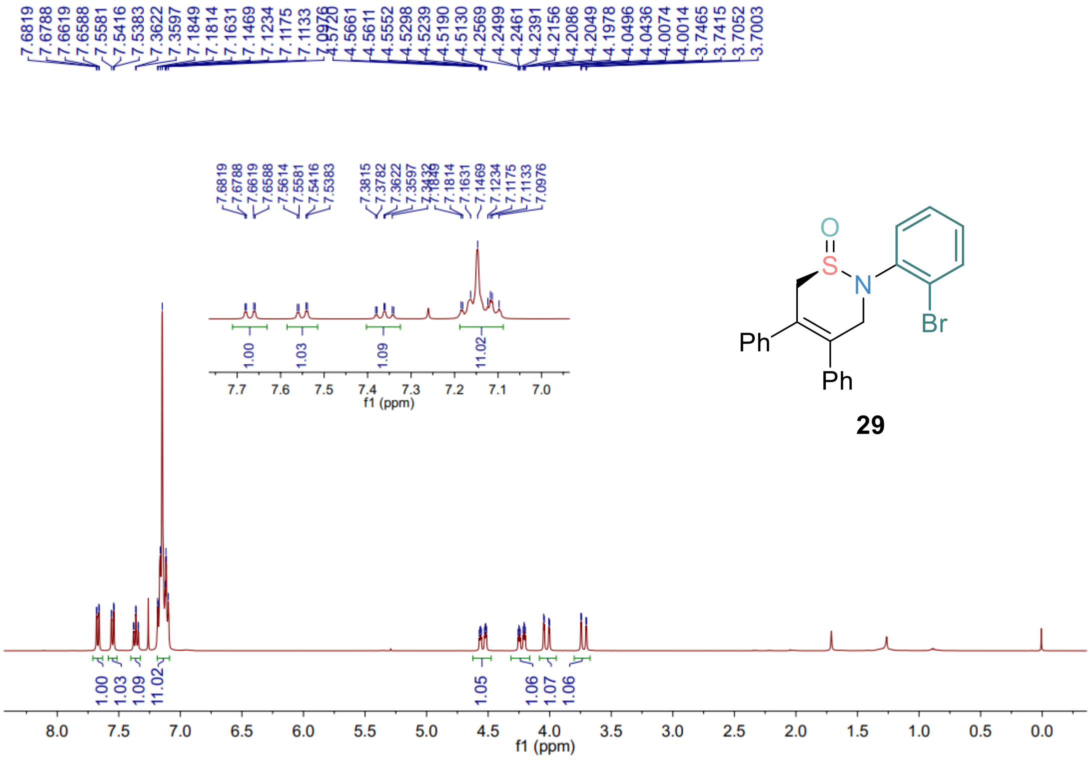

29

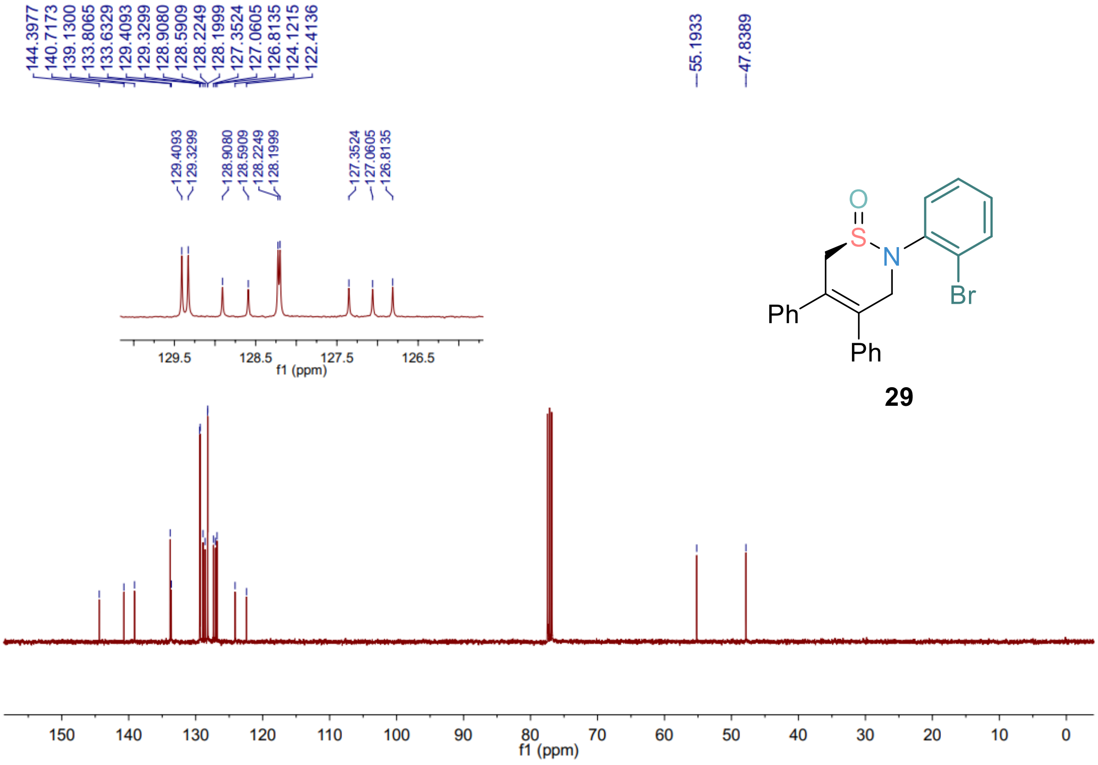

29

^{1}H-NMR and ^{13}C-NMR of **29**

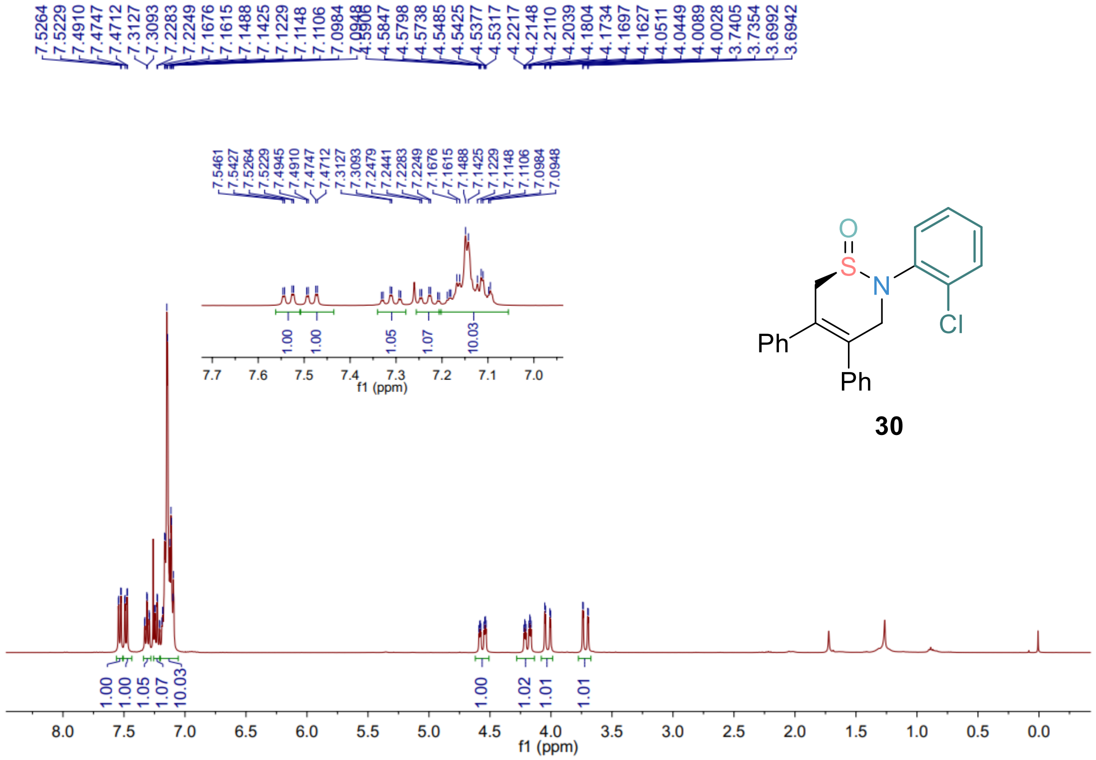

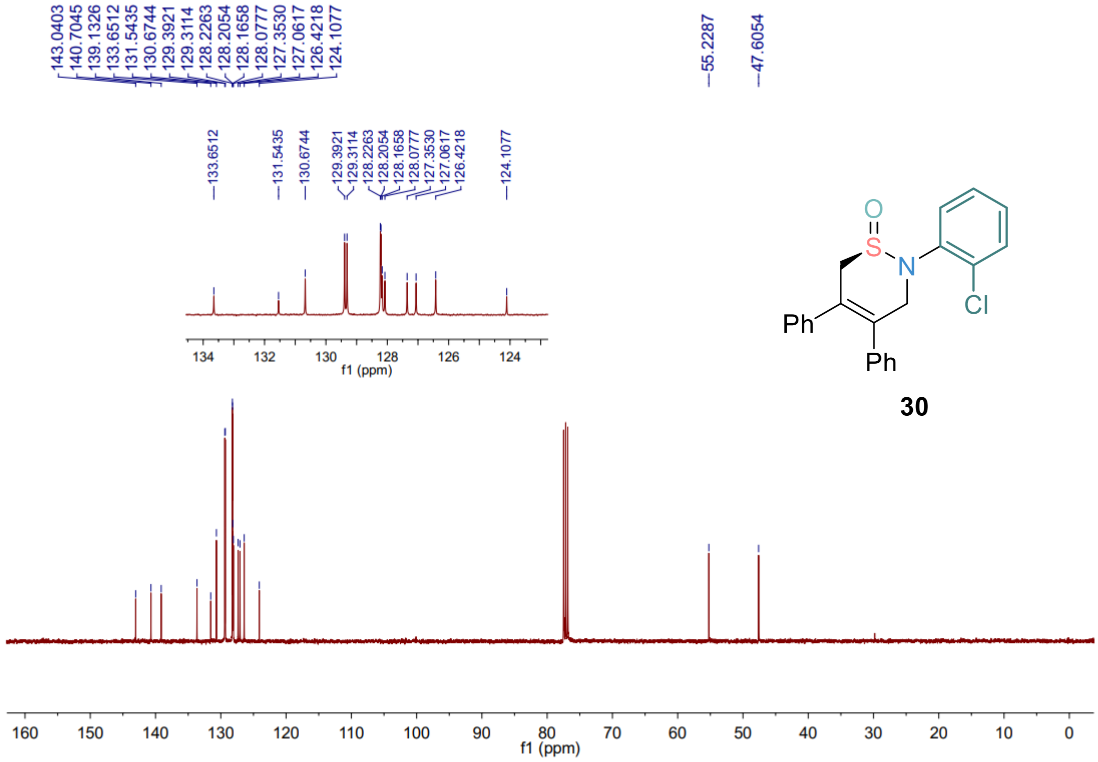

^{1}H-NMR and ^{13}C-NMR of **30**

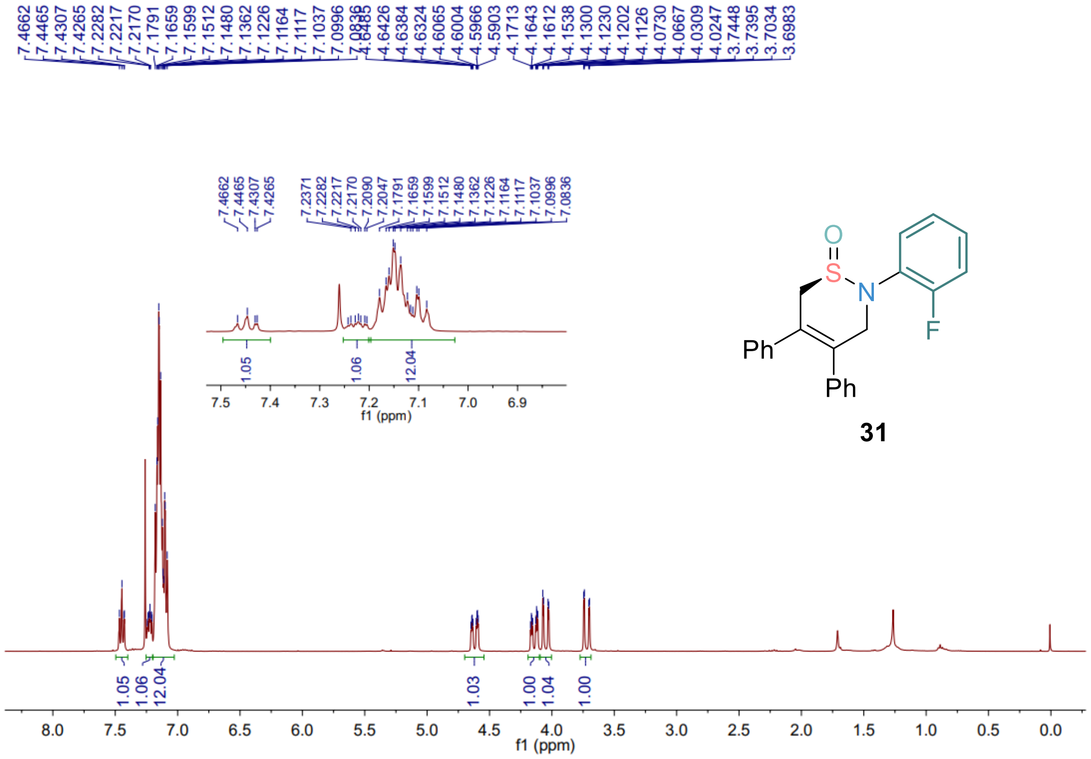

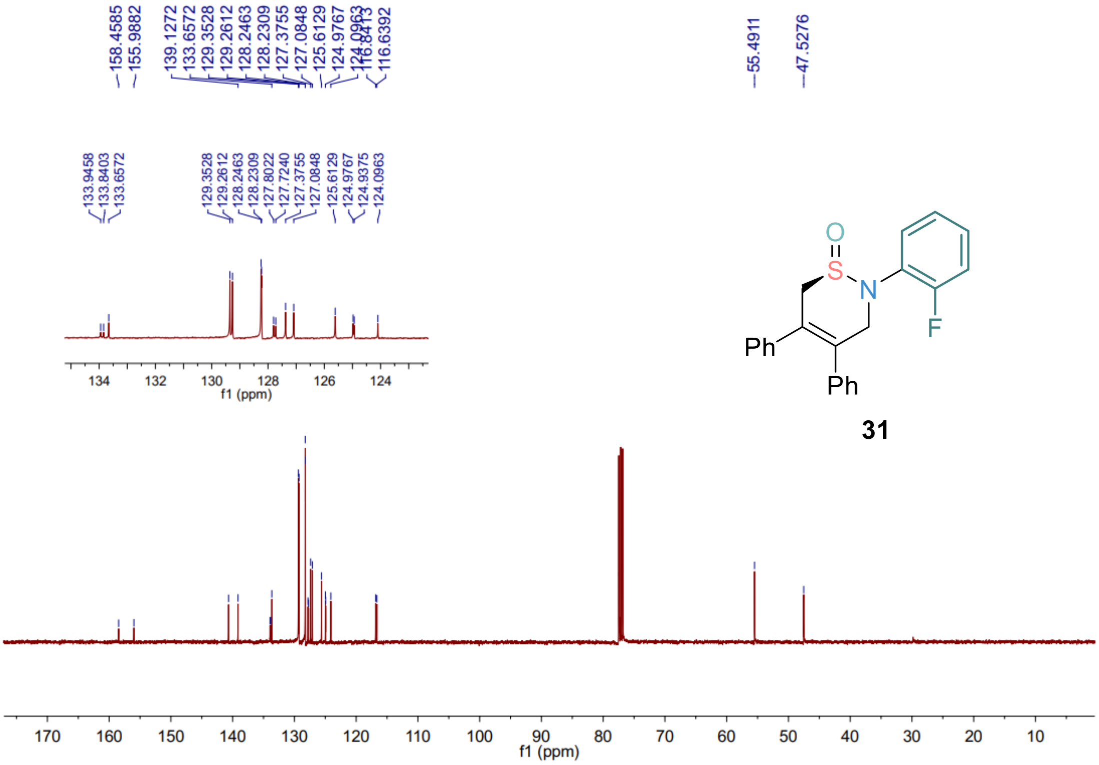

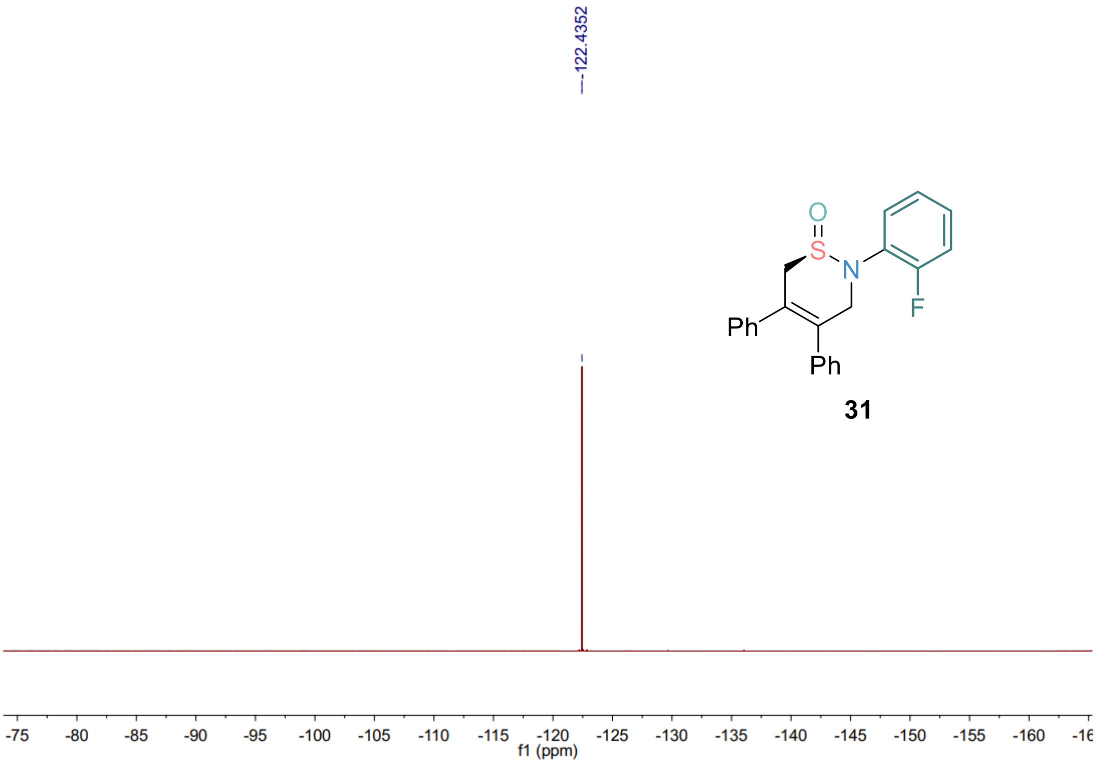

^{1}H-NMR and ^{13}C-NMR and ^{19}F-NMR of **31**

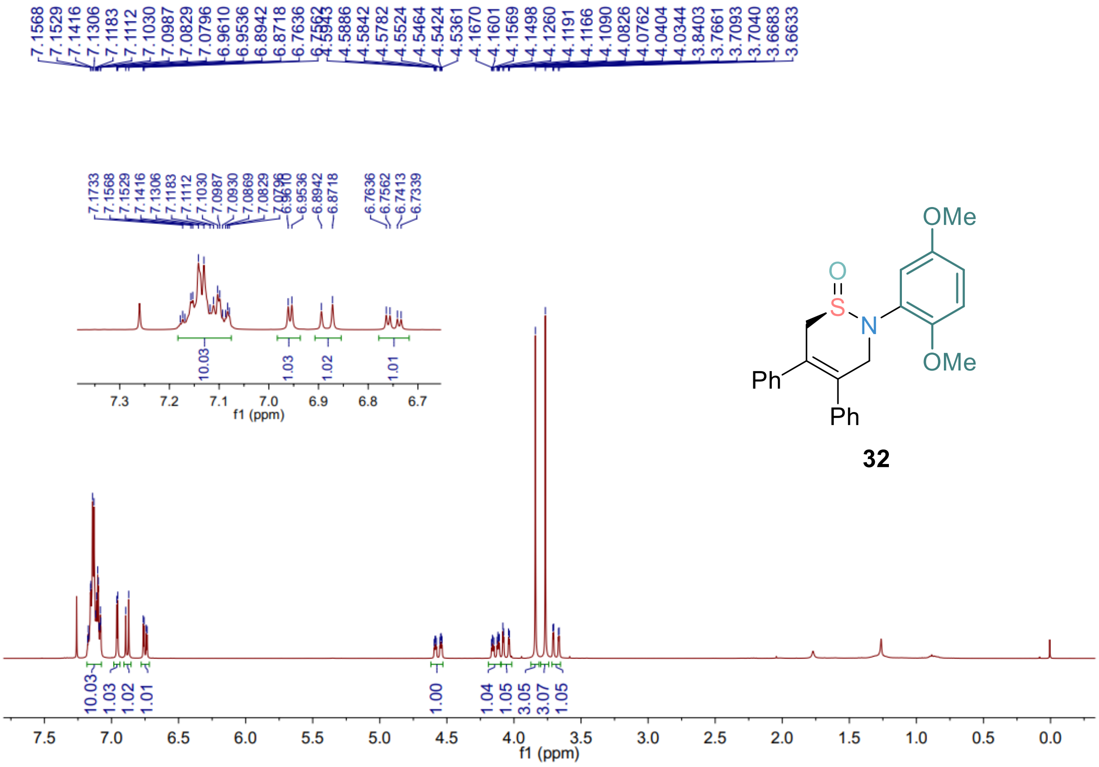

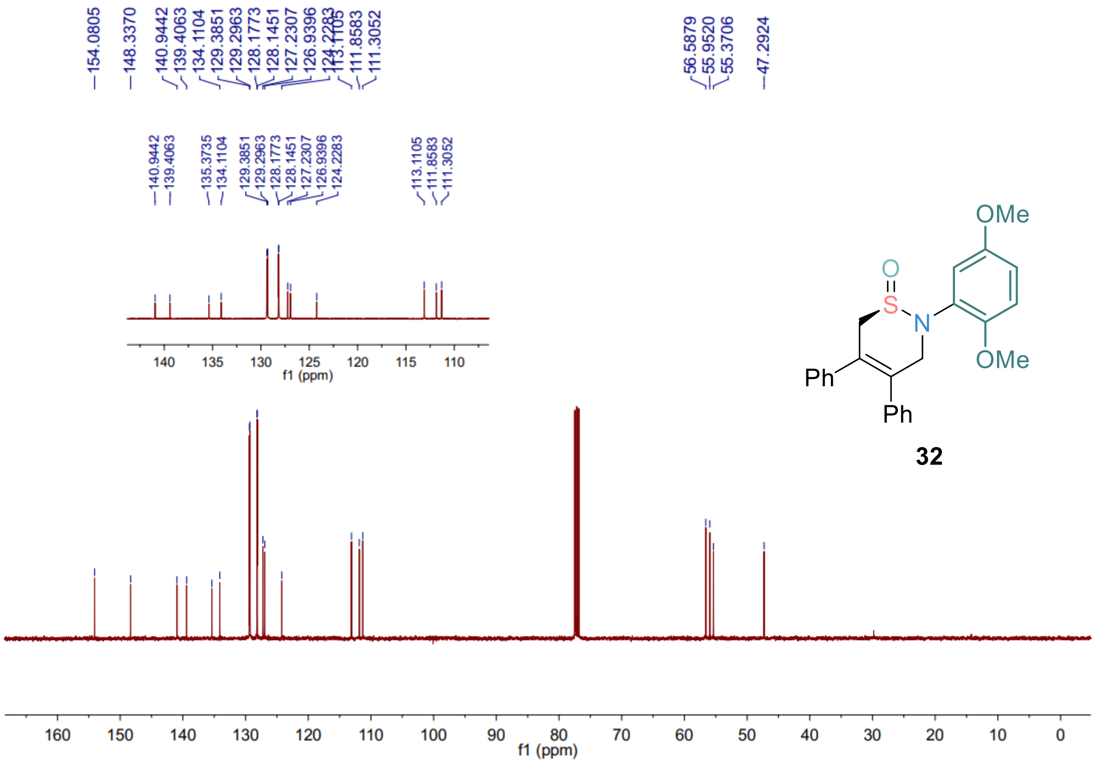

32

^{1}H-NMR and ^{13}C-NMR of **32**

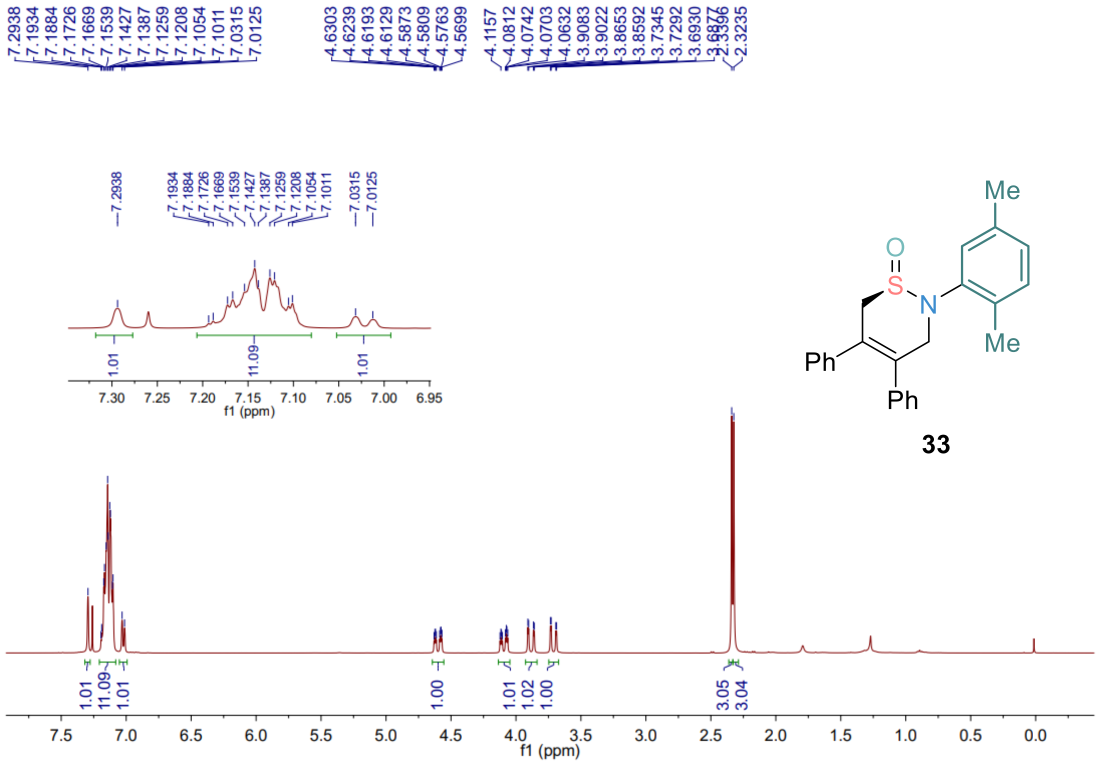

33

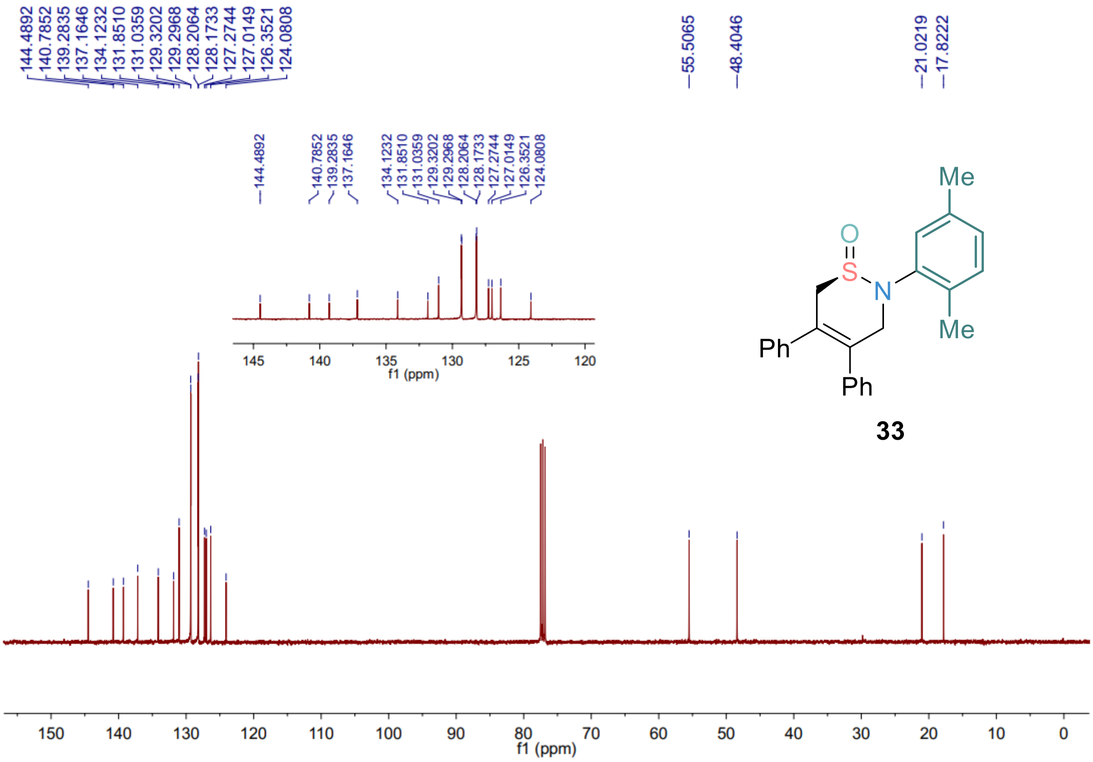

^{1}H-NMR and ^{13}C-NMR of **33**

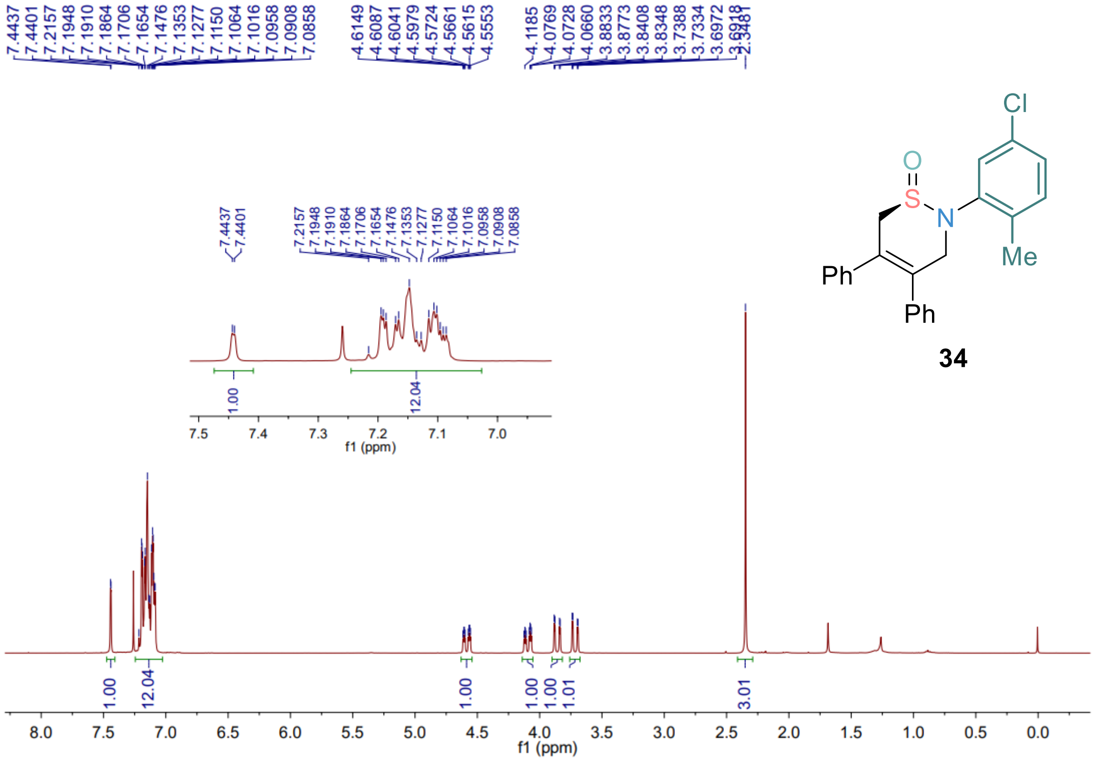

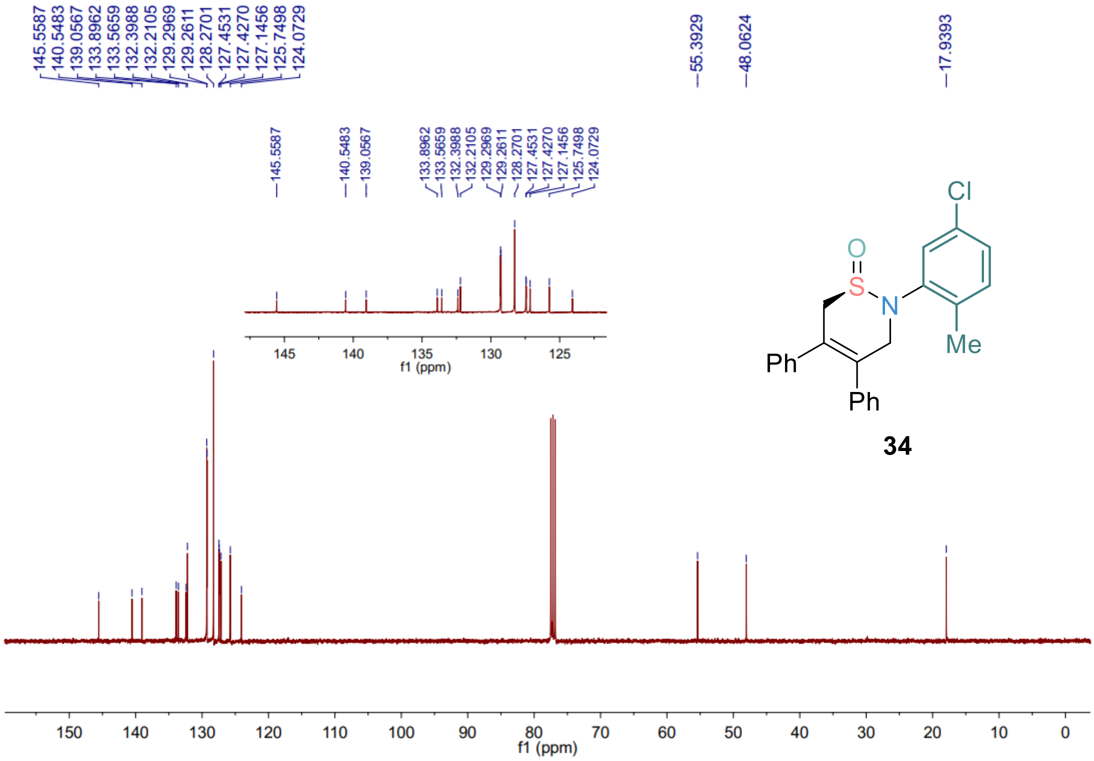

^{1}H-NMR and ^{13}C-NMR of **34**

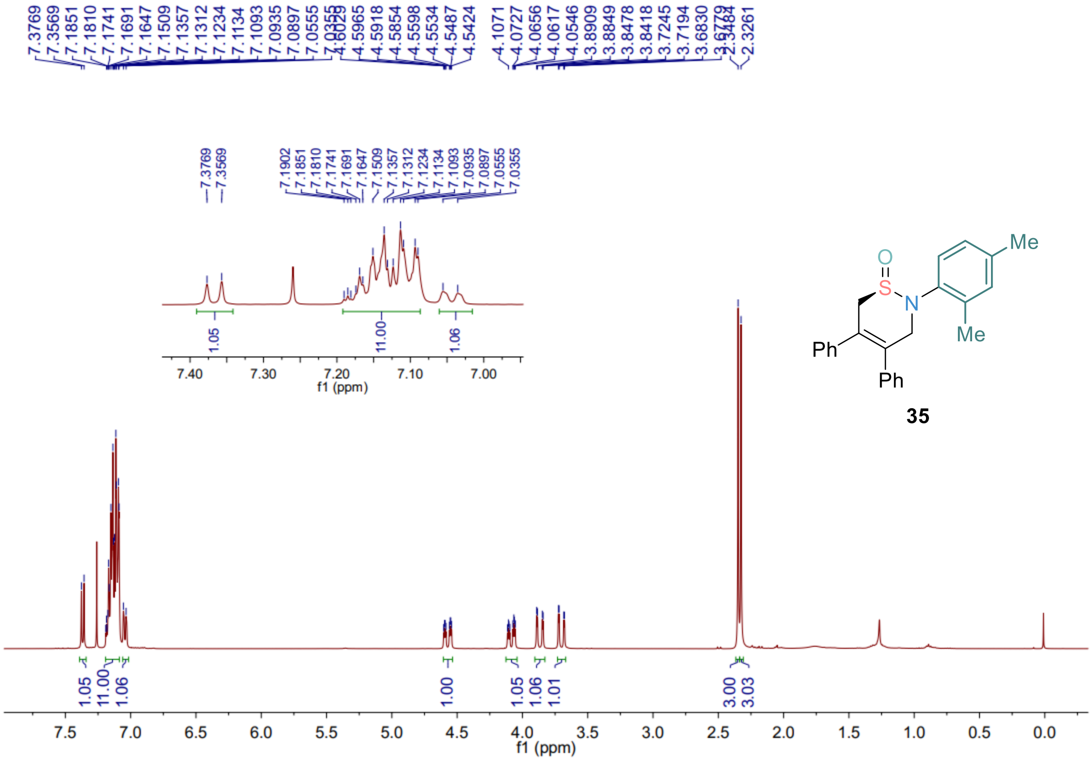

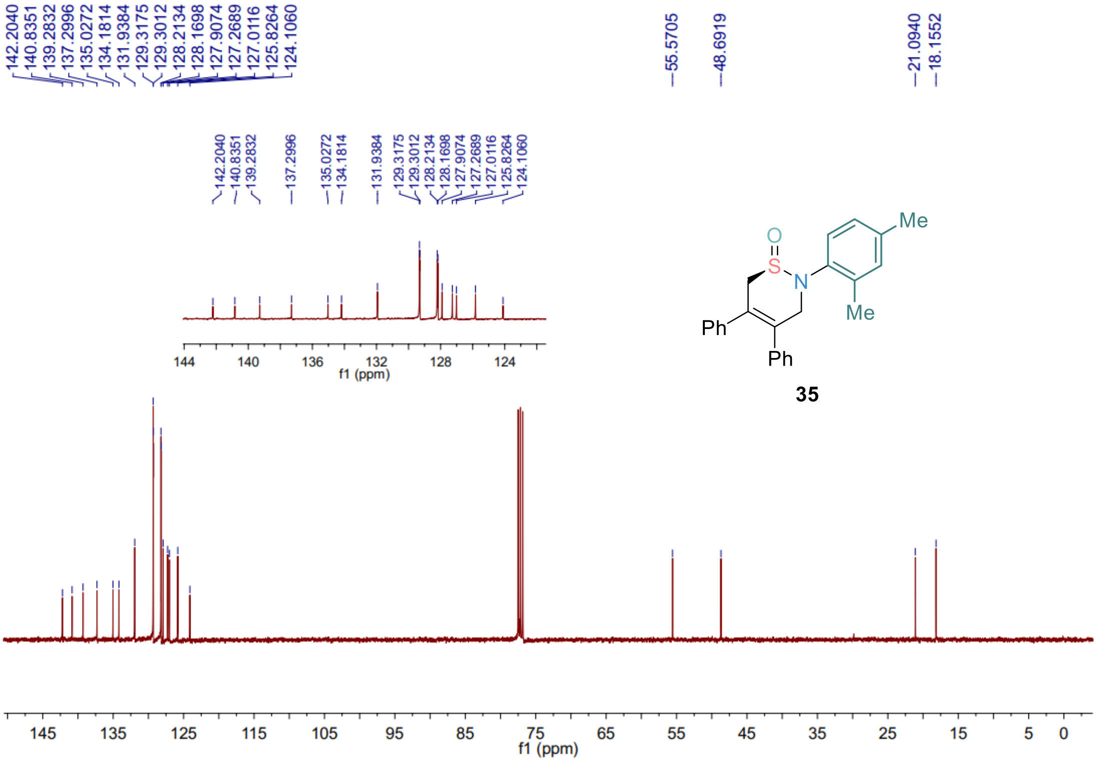

35

^{1}H-NMR and ^{13}C-NMR of **35**

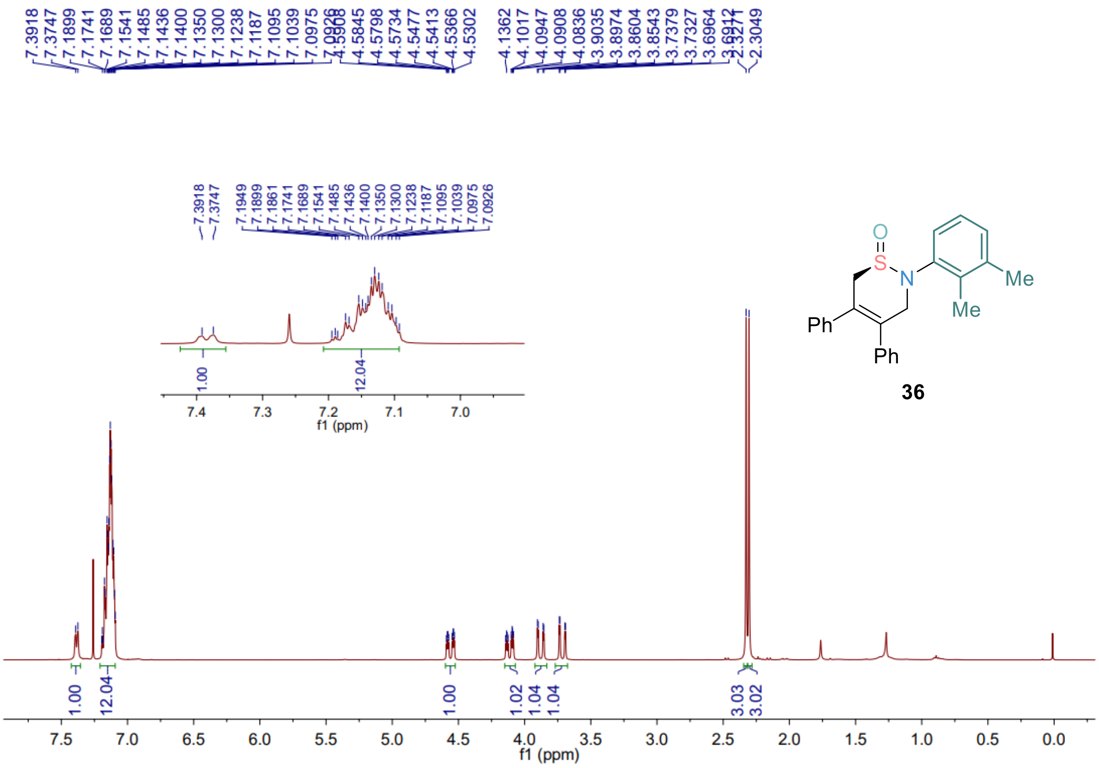

36

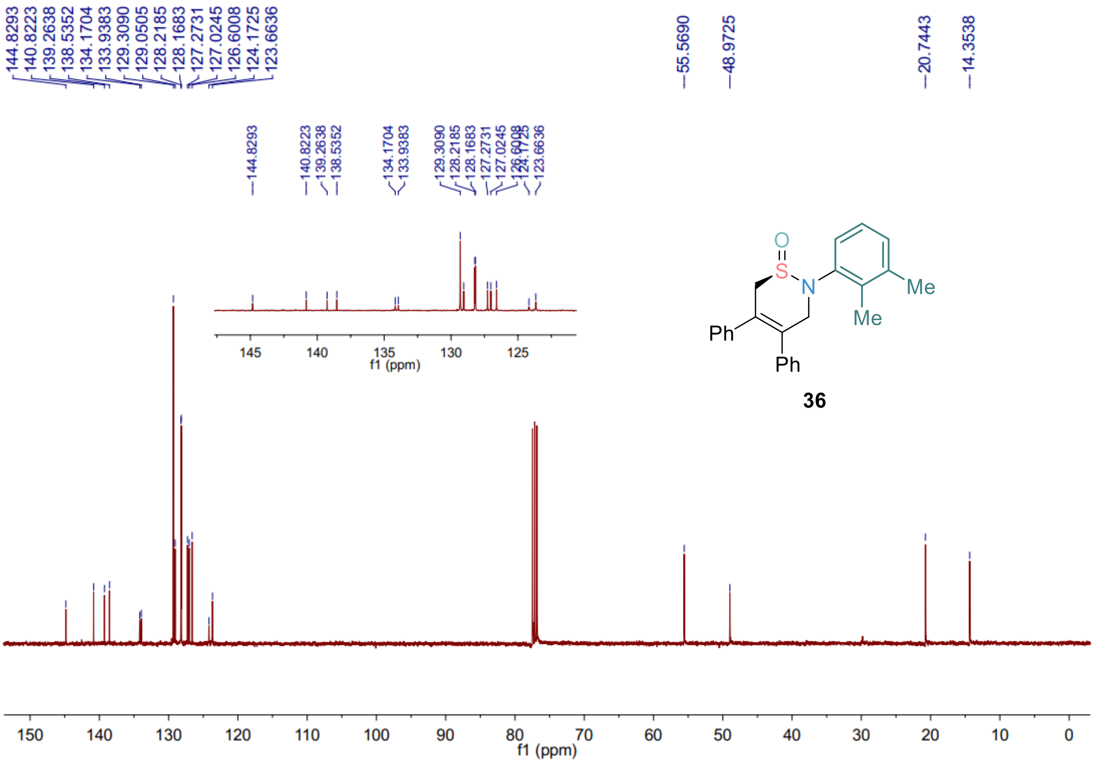

^{1}H-NMR and ^{13}C-NMR of **36**

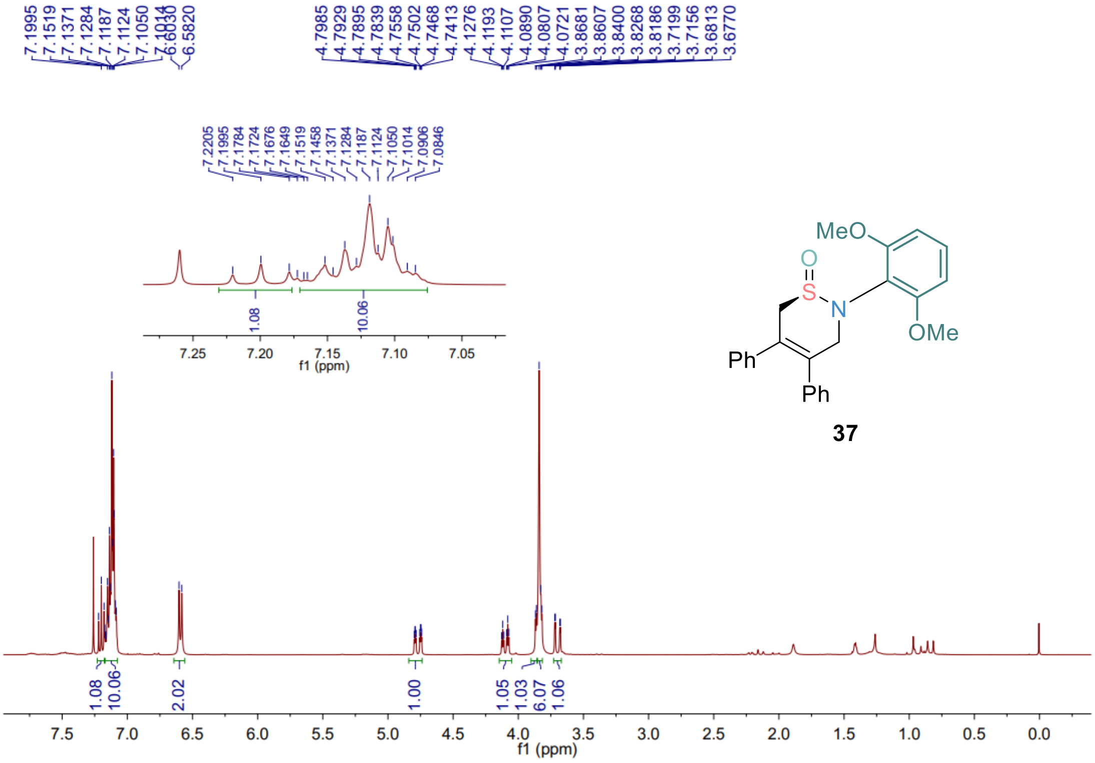

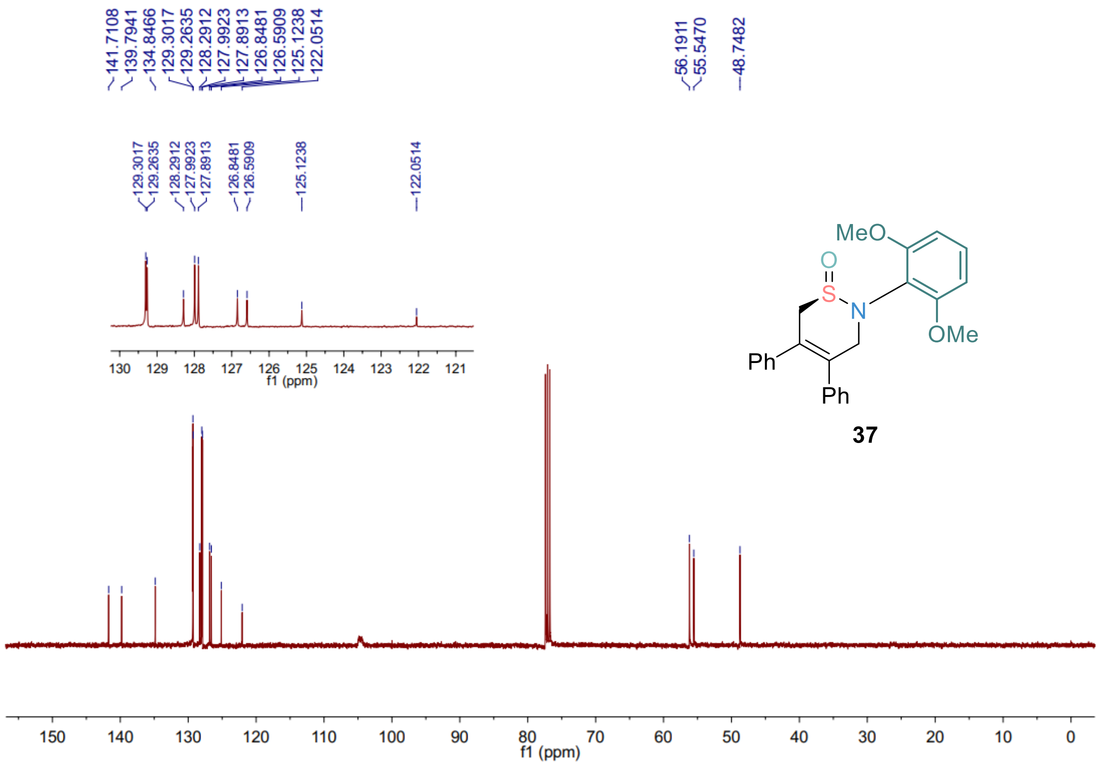

^{1}H-NMR and ^{13}C-NMR of **37**

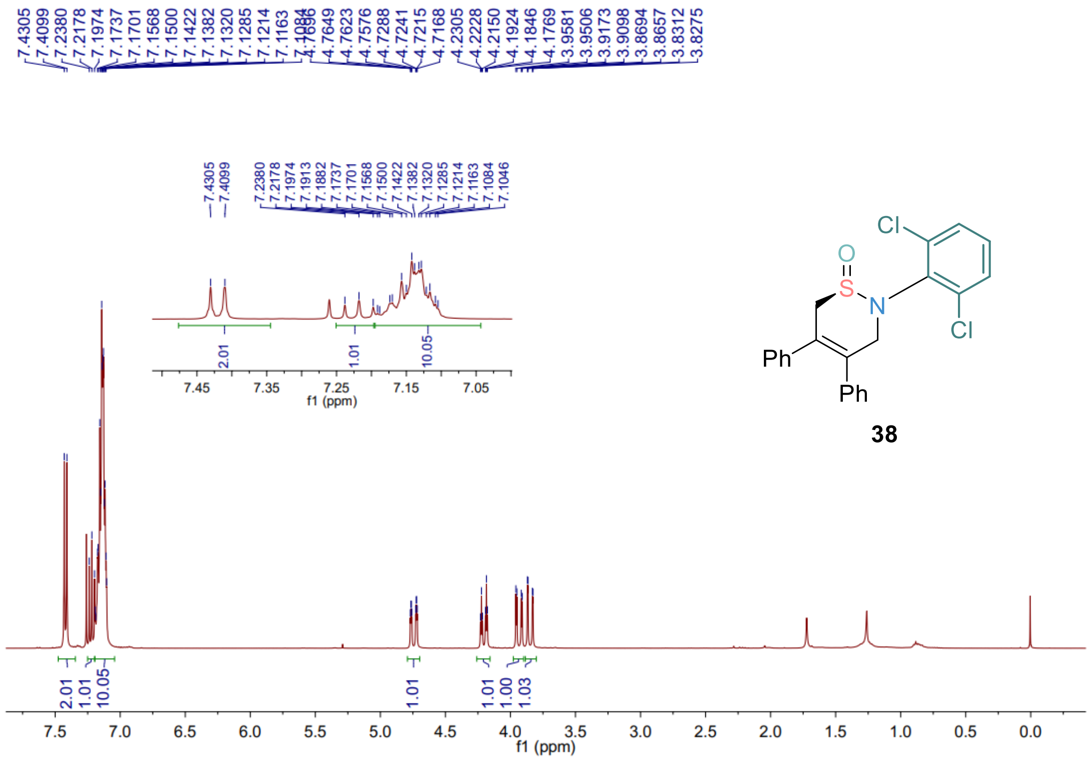

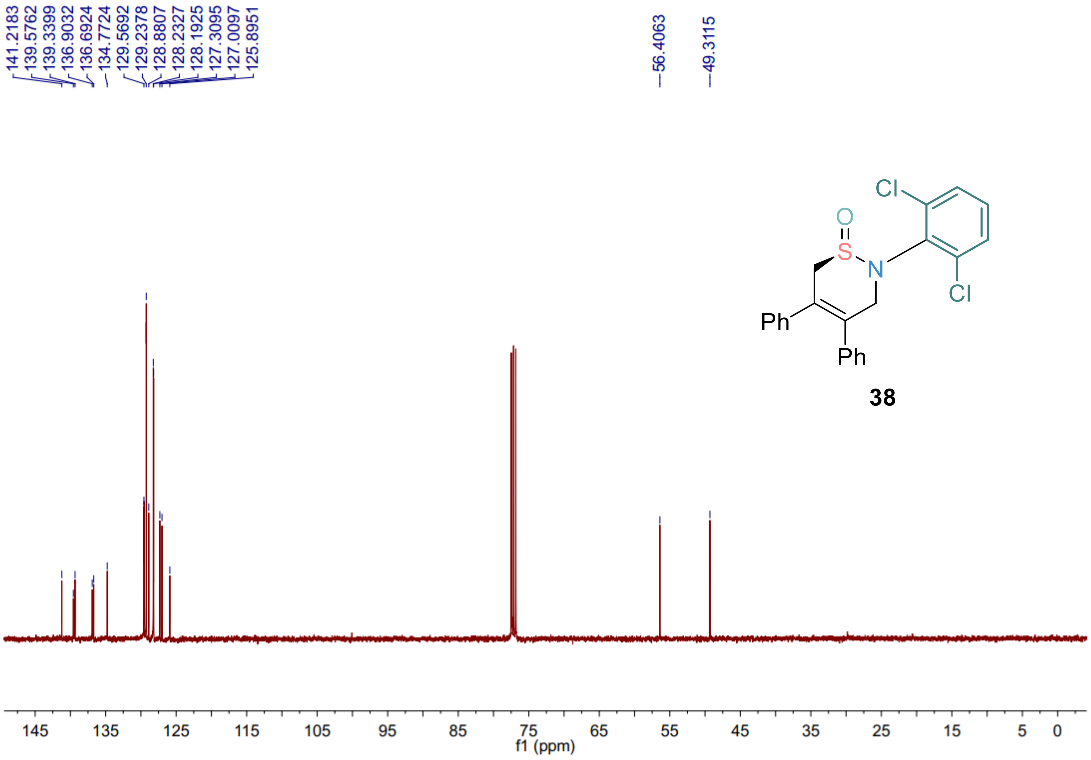

^{1}H-NMR and ^{13}C-NMR of **38**

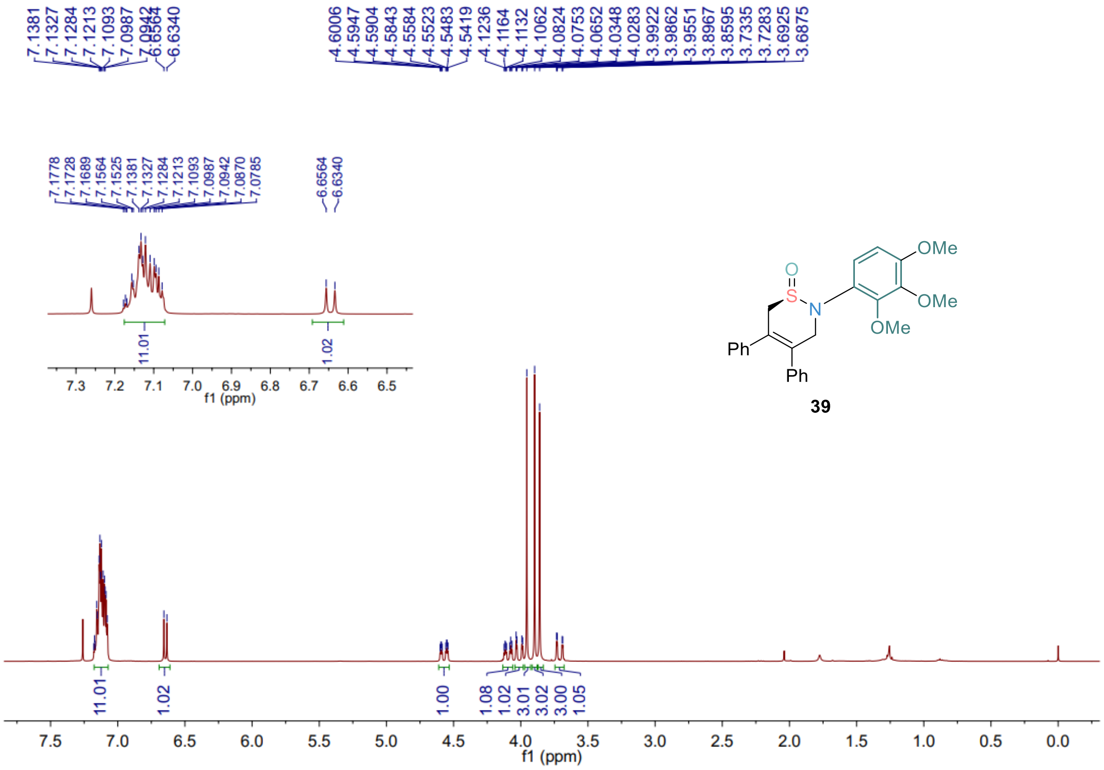

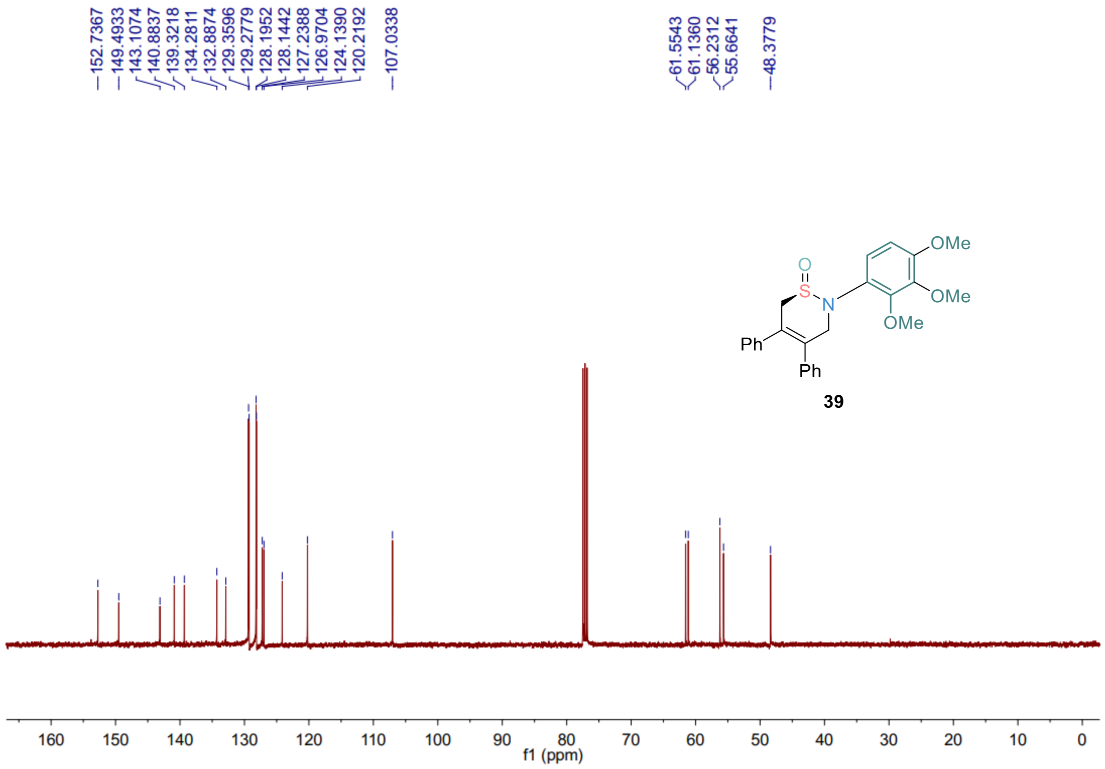

^{1}H-NMR and ^{13}C-NMR of **39**

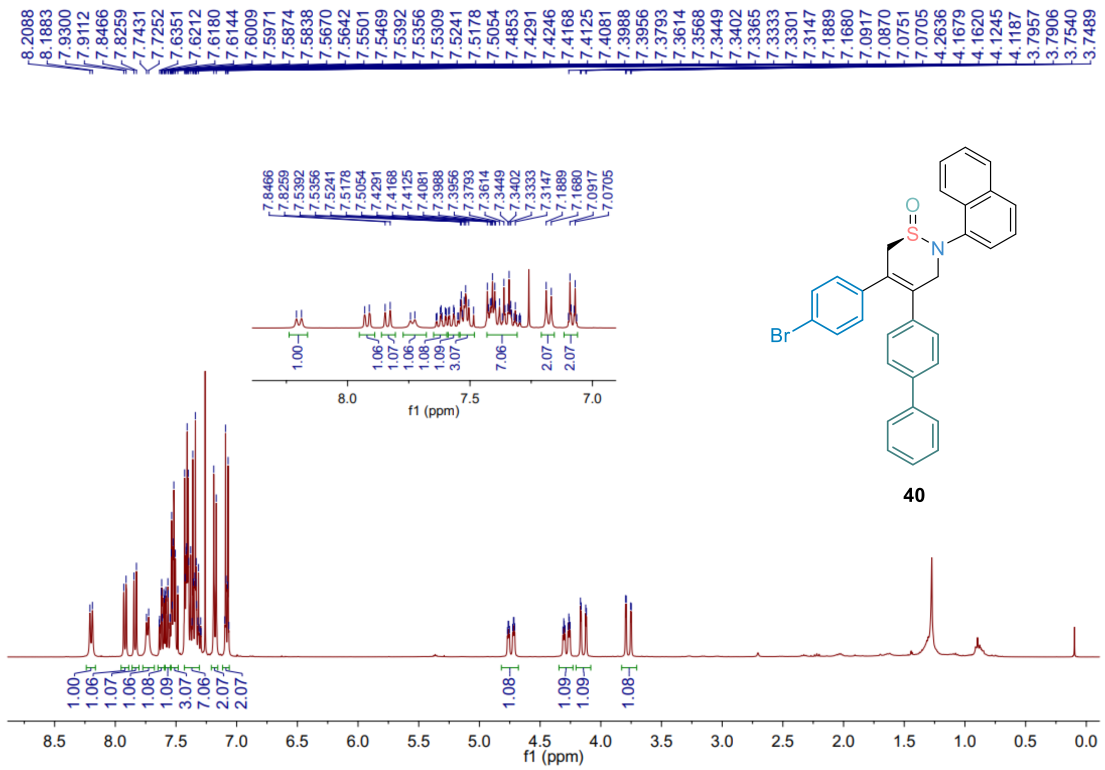

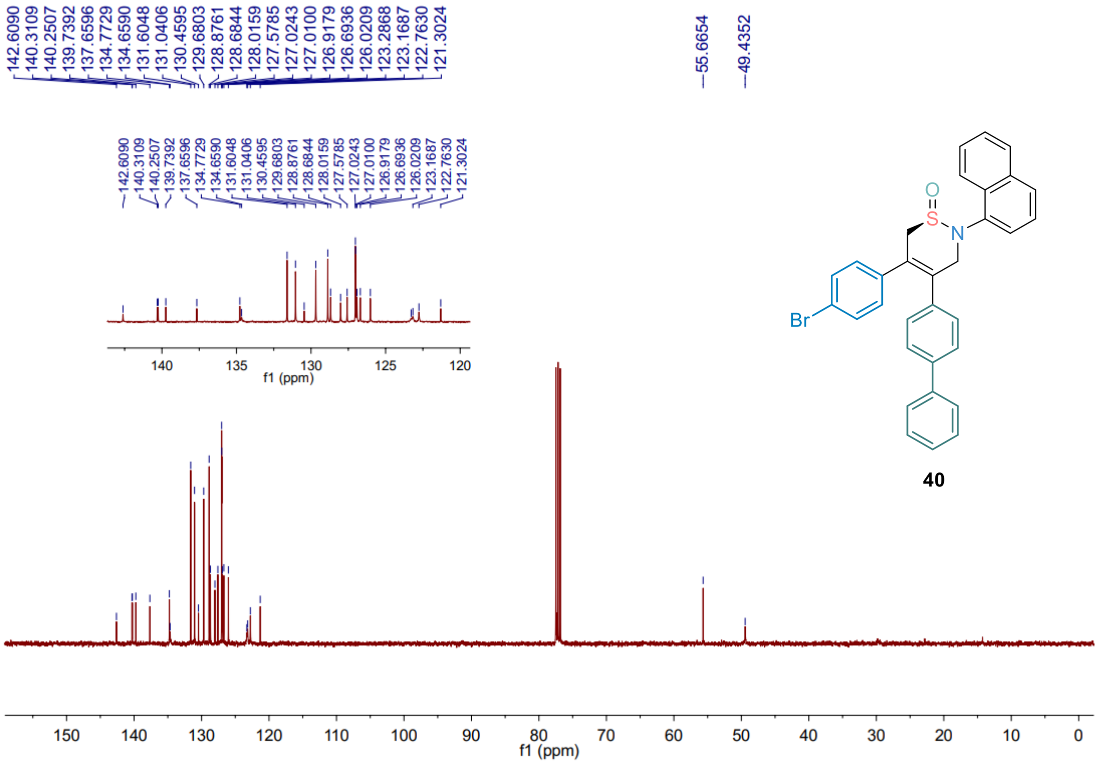

^{1}H-NMR and ^{13}C-NMR of **40**

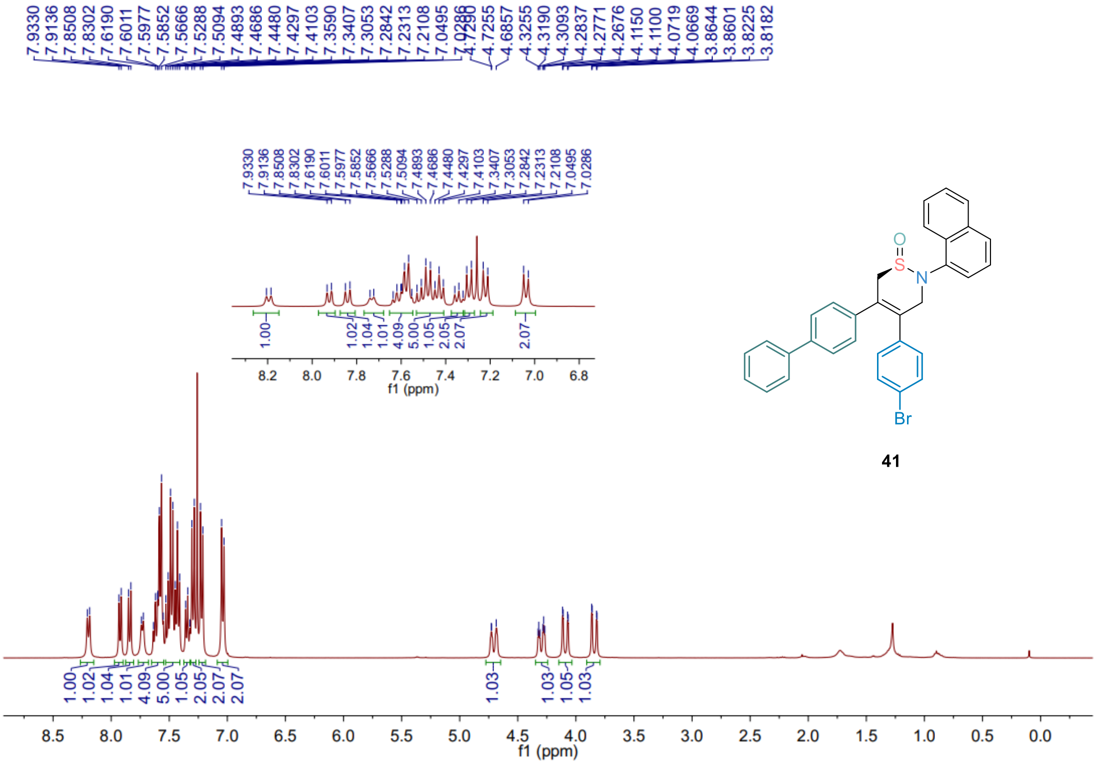

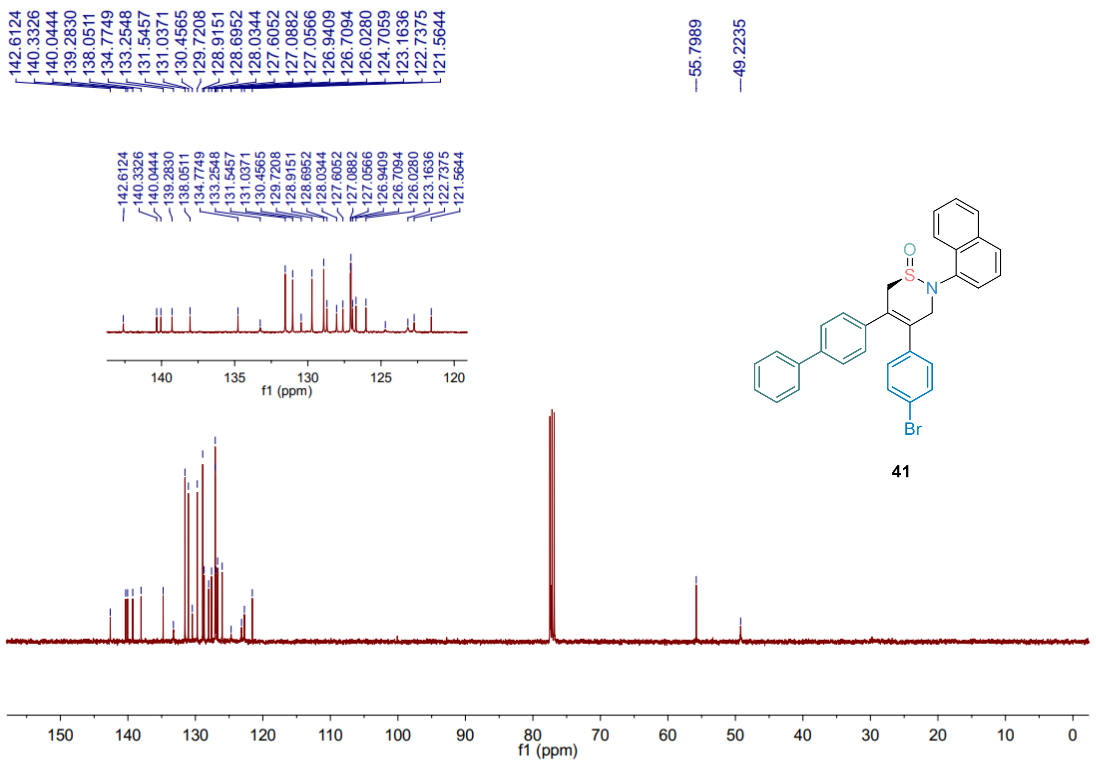

^{1}H-NMR and ^{13}C-NMR of **41**

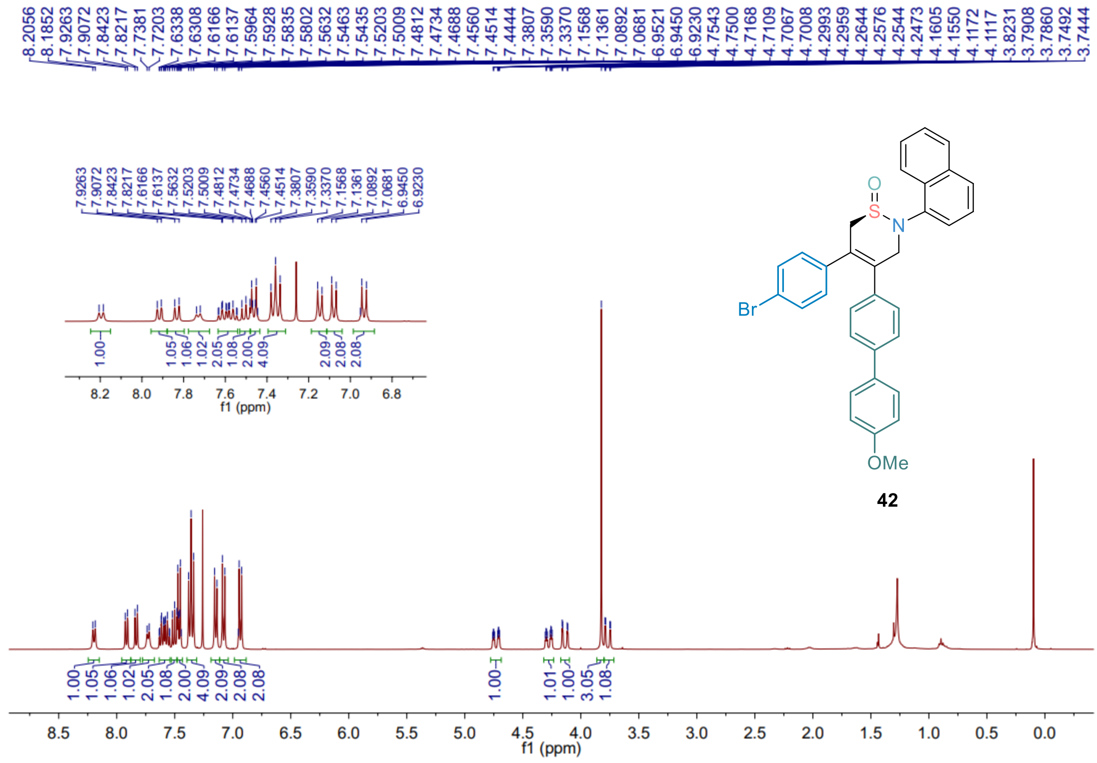

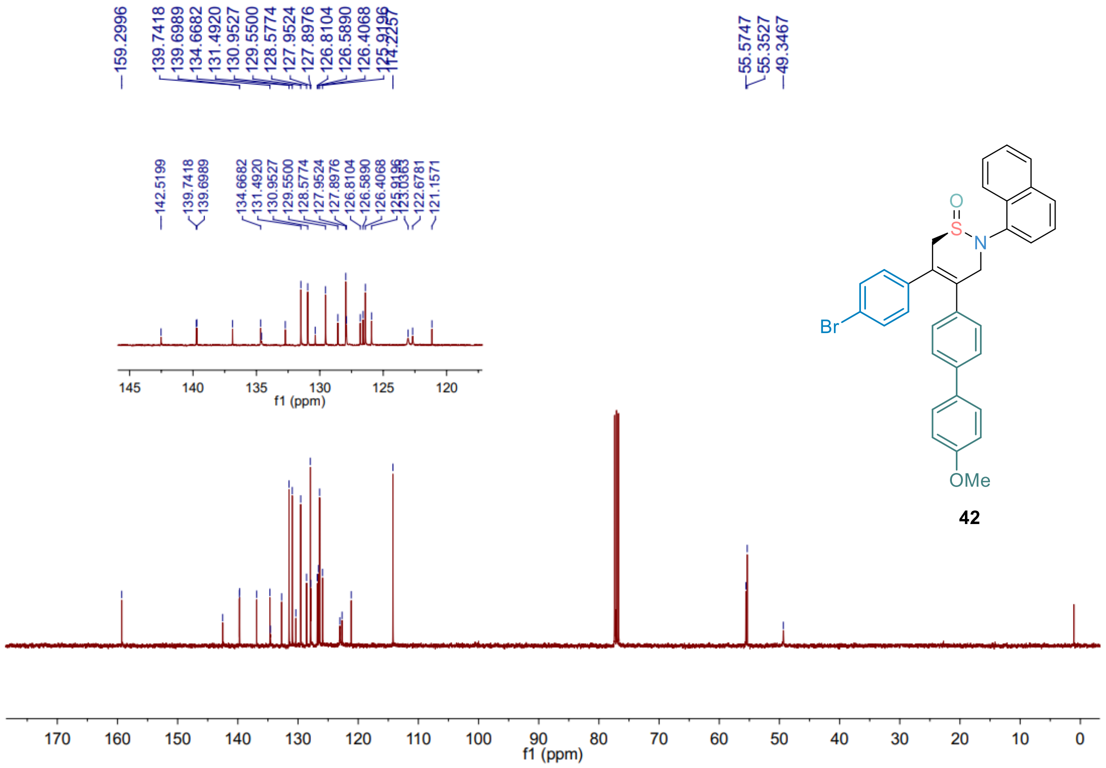

^{1}H-NMR and ^{13}C-NMR of **42**

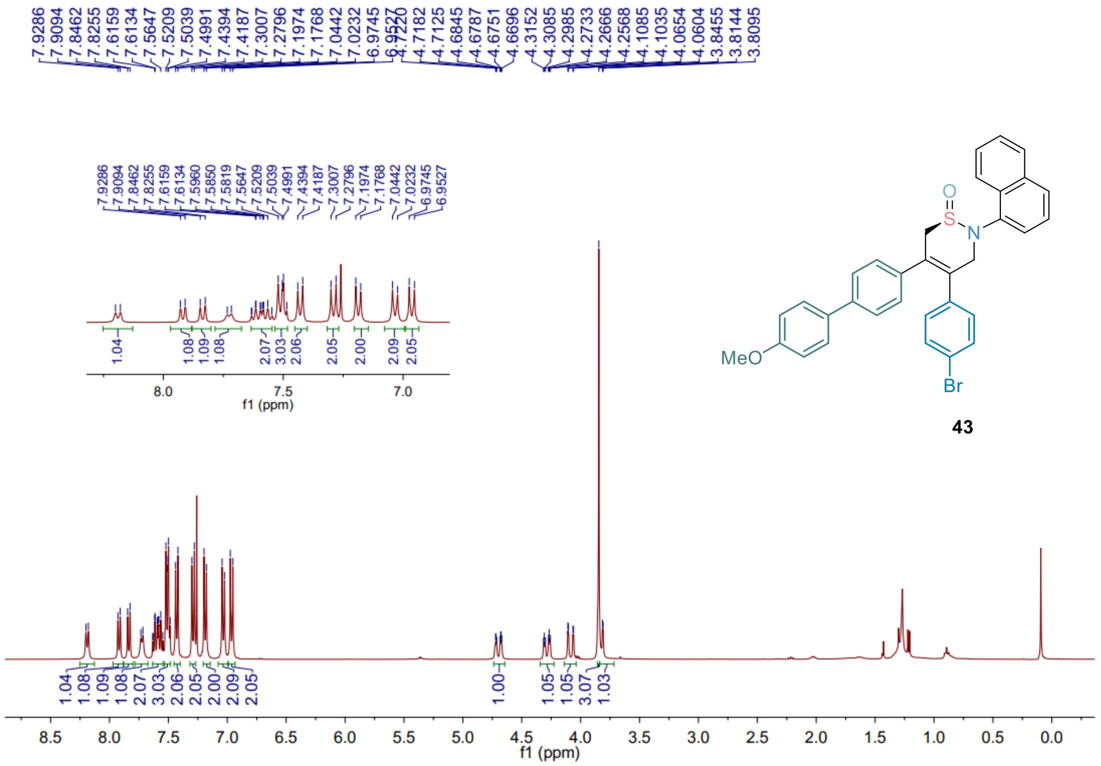

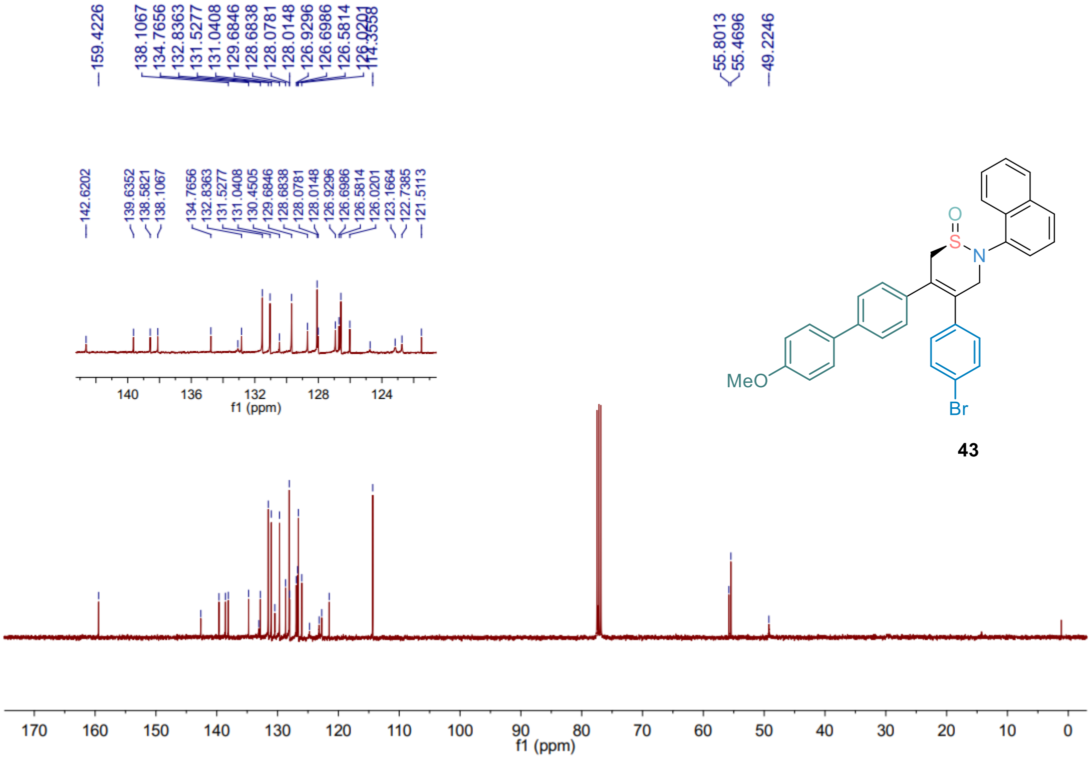

43

^{1}H-NMR and ^{13}C-NMR of **43**

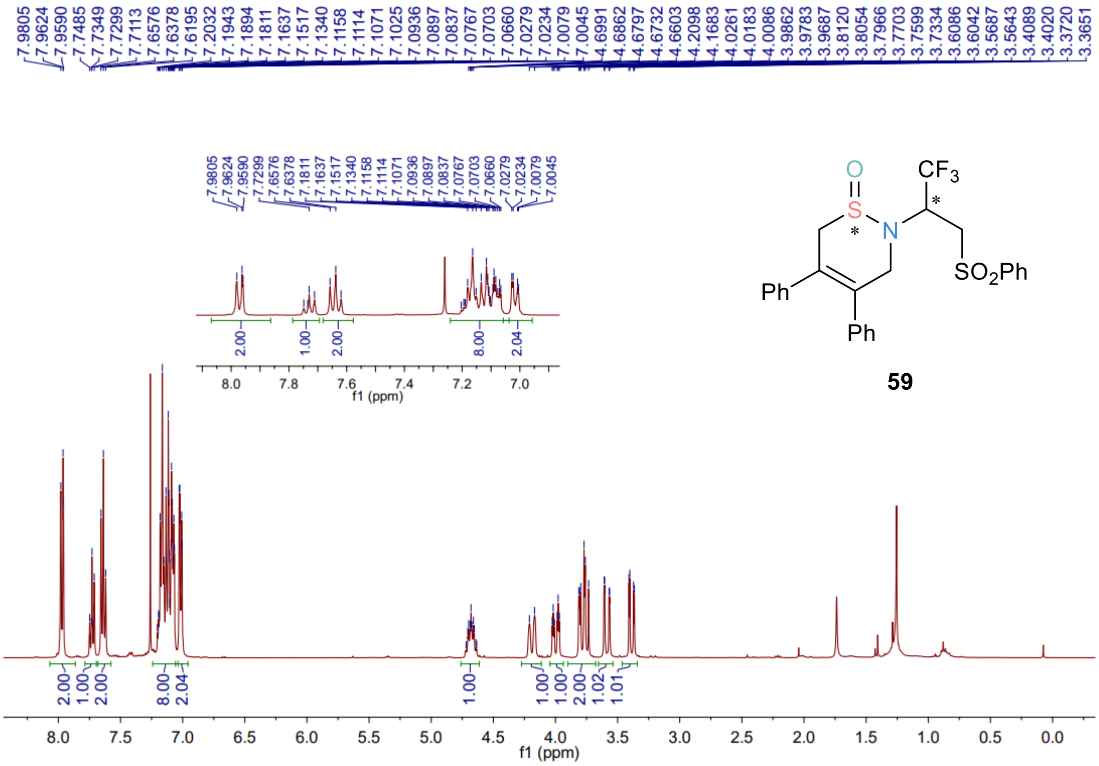

59

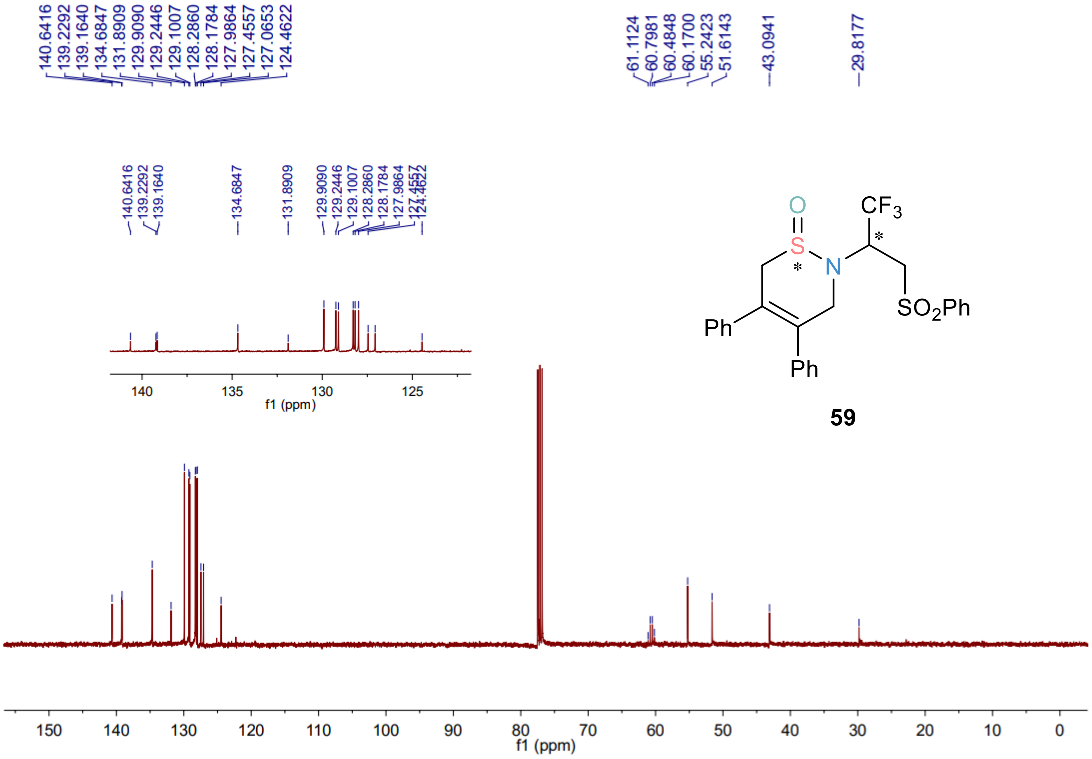

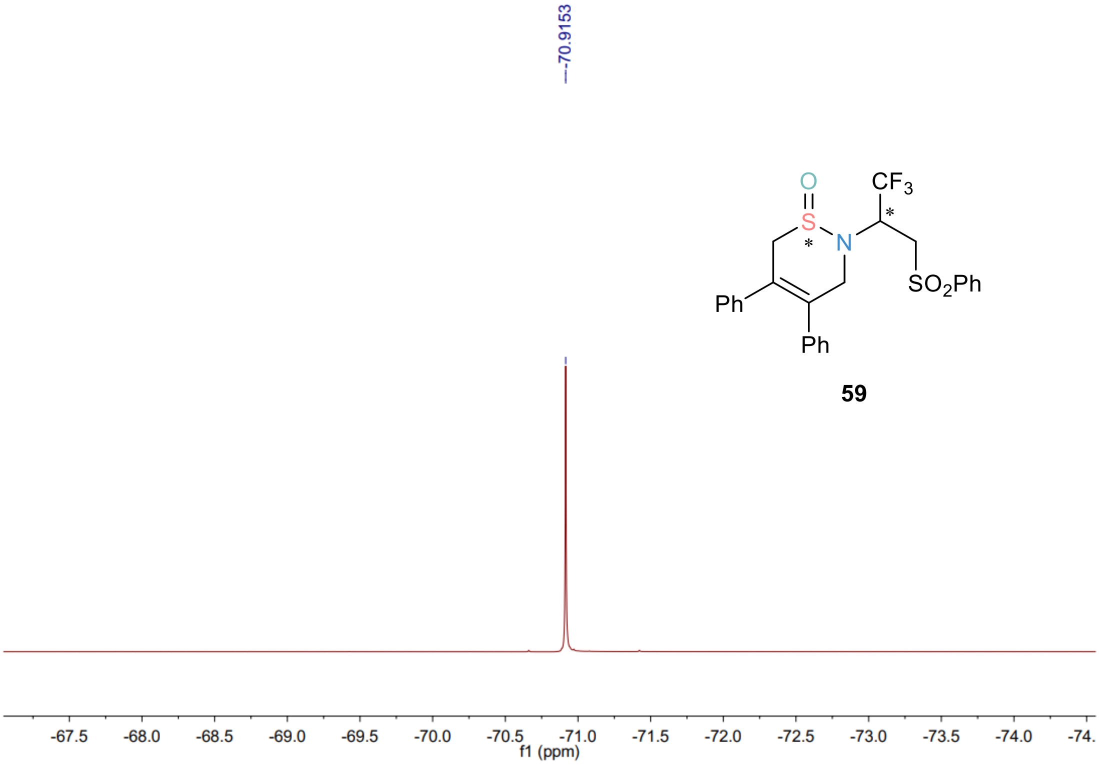

^{1}H-NMR and ^{13}C-NMR and ^{19}F-NMR of **59**

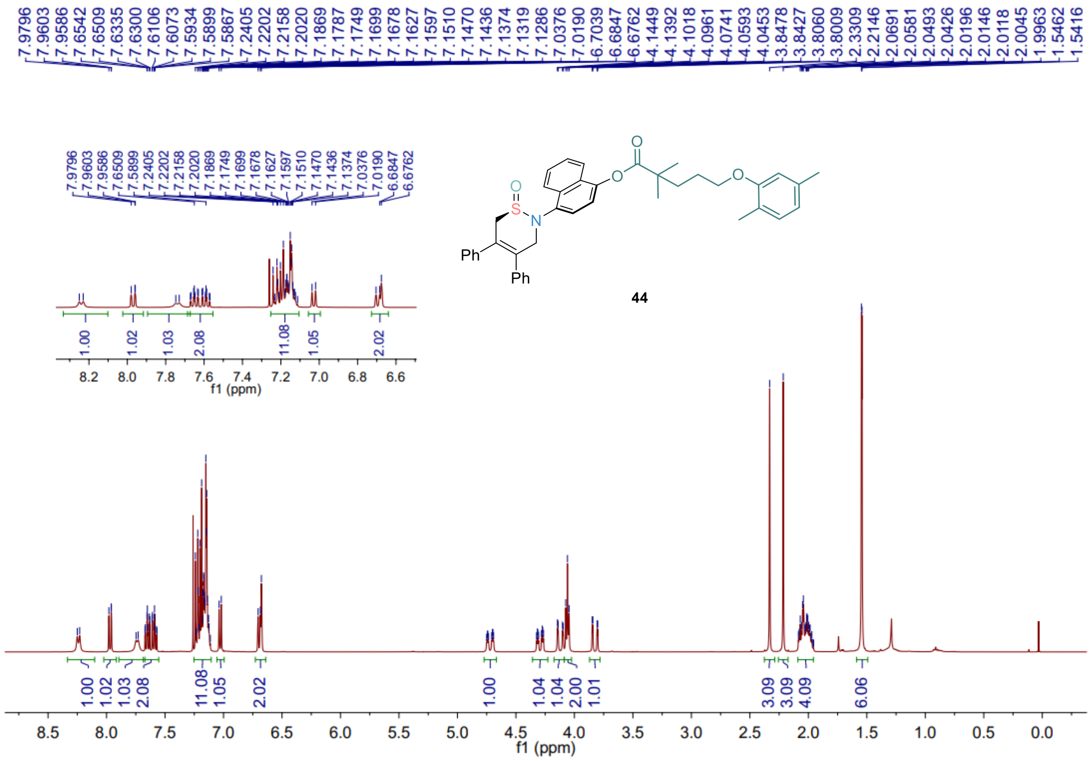

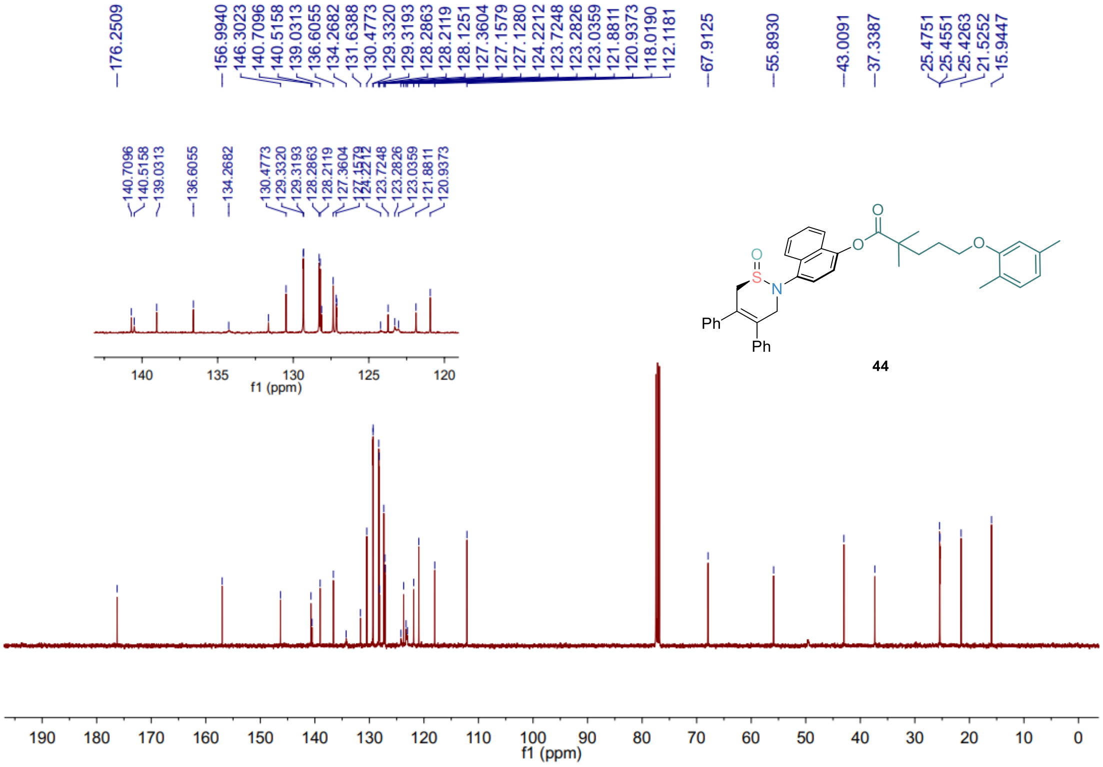

^{1}H-NMR and ^{13}C-NMR of **44**

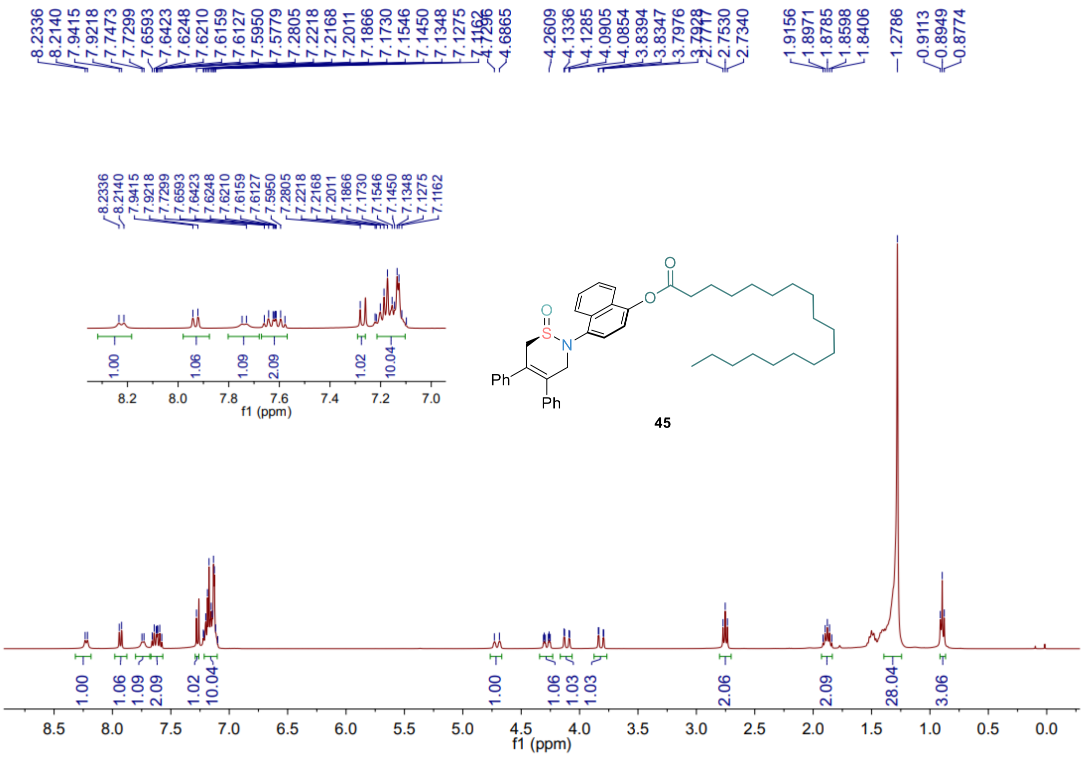

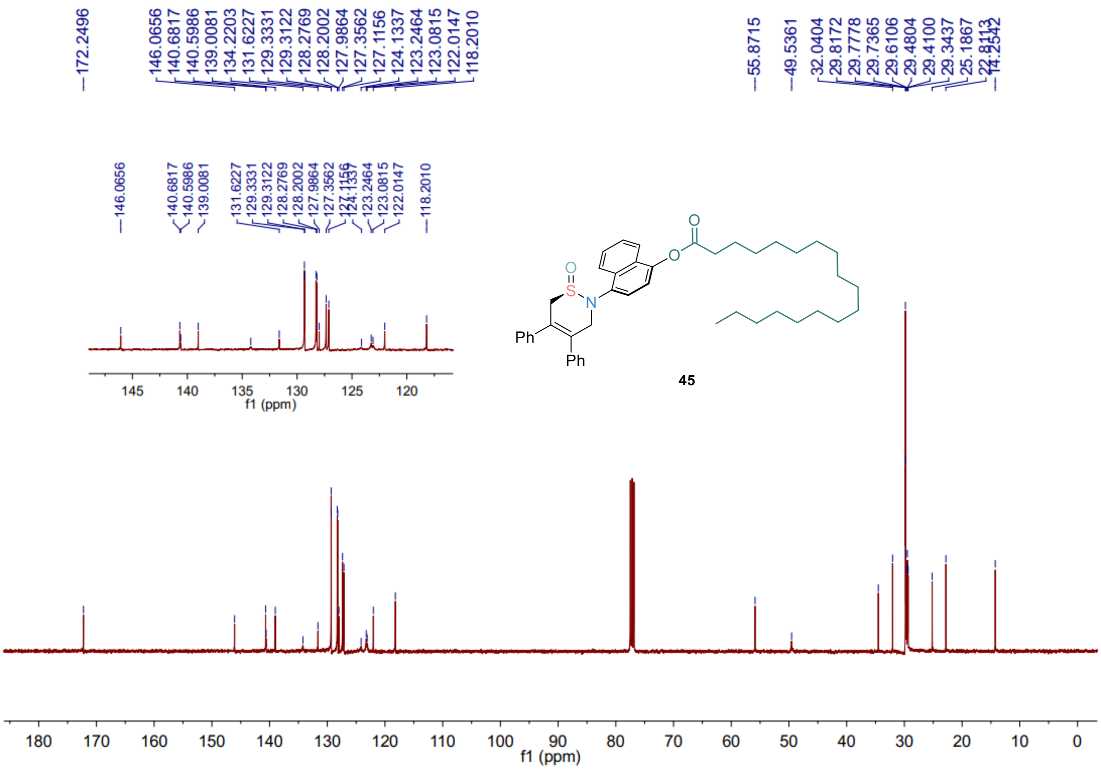

^{1}H-NMR and ^{13}C-NMR of **45**

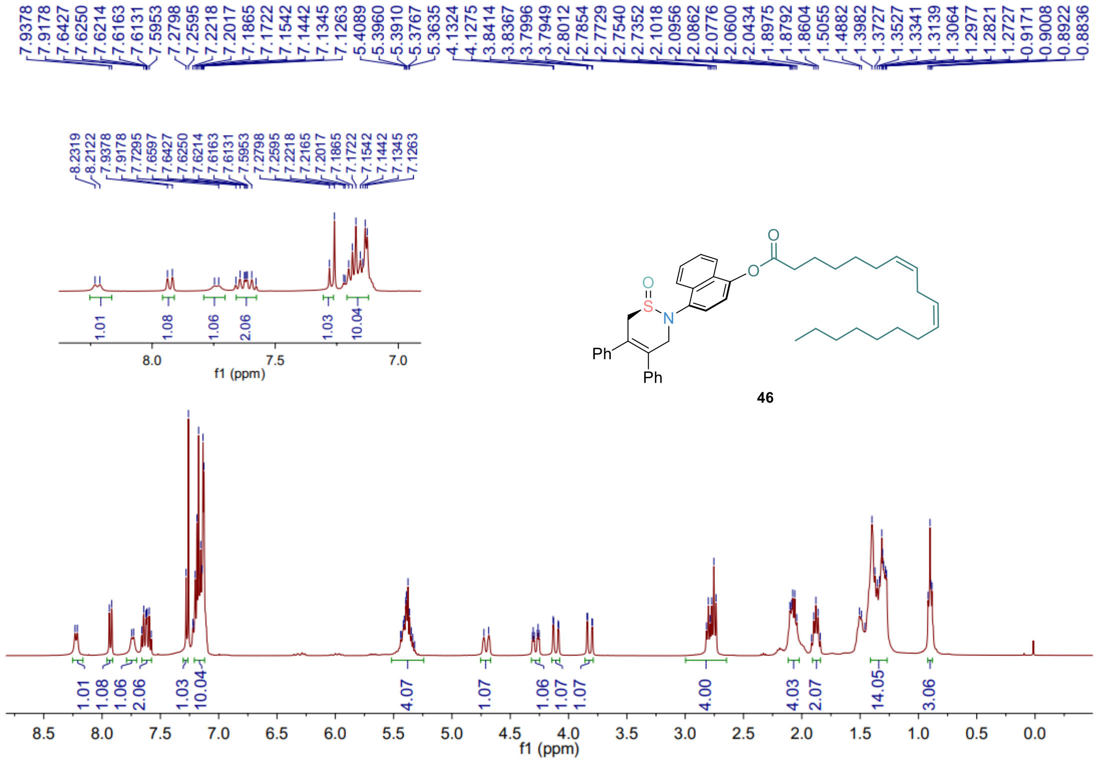

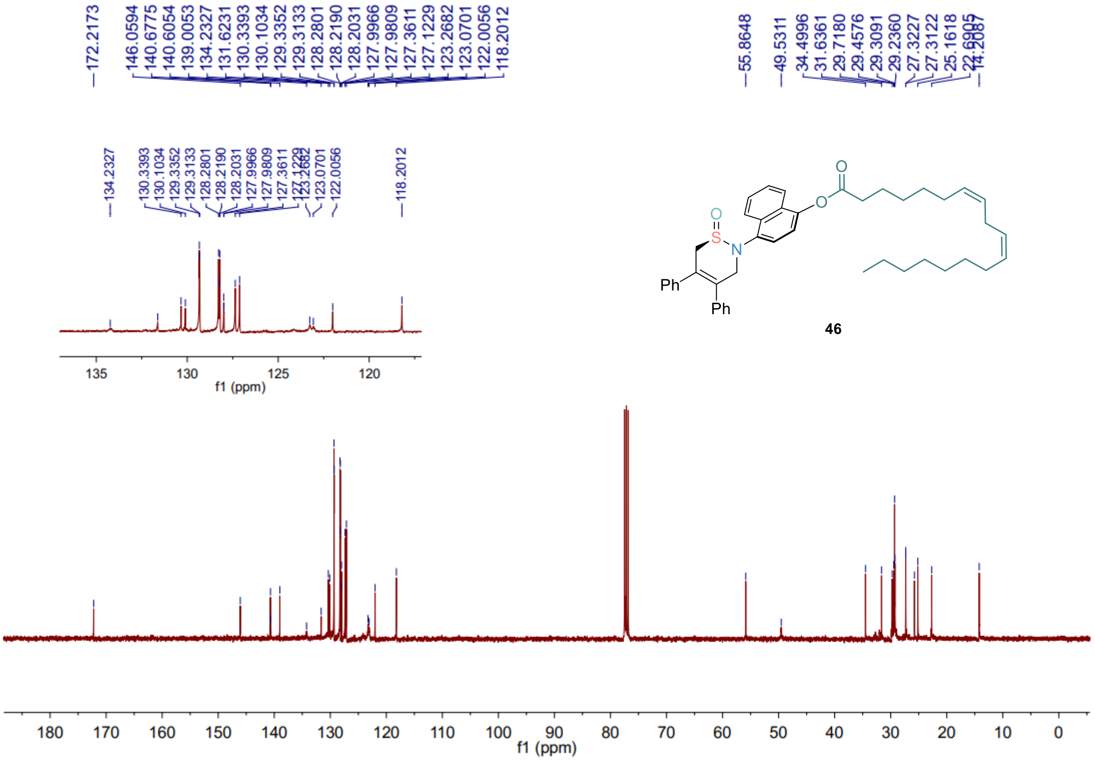

^{1}H-NMR and ^{13}C-NMR of **46**

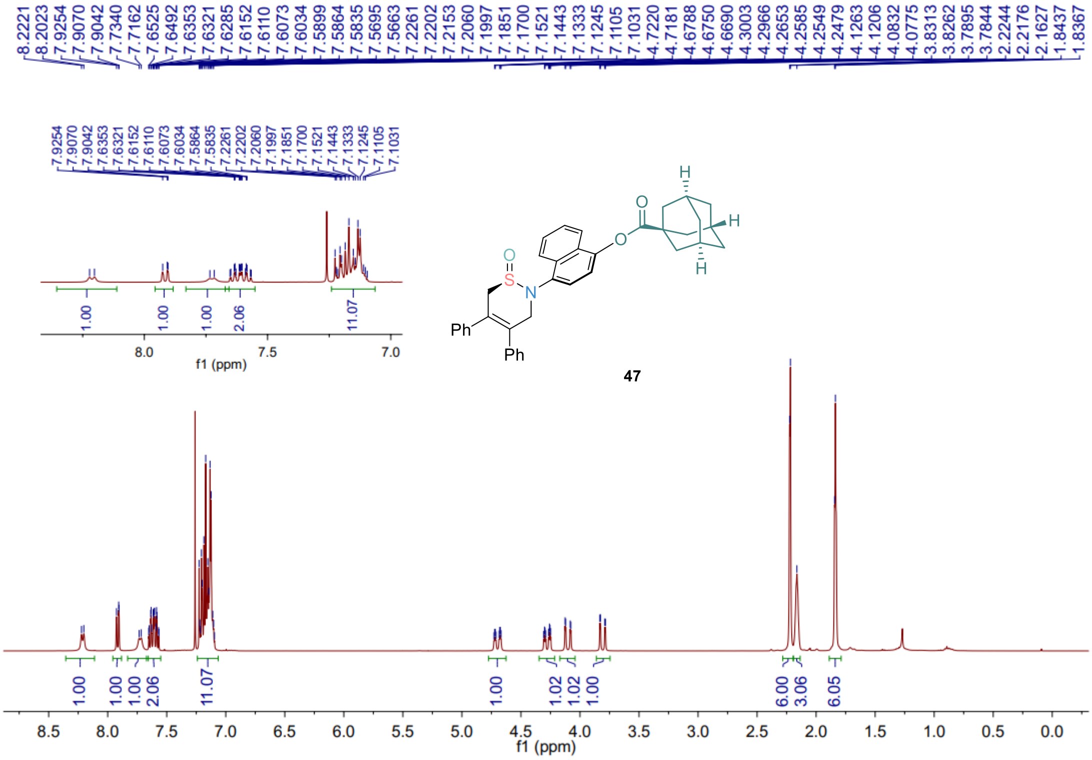

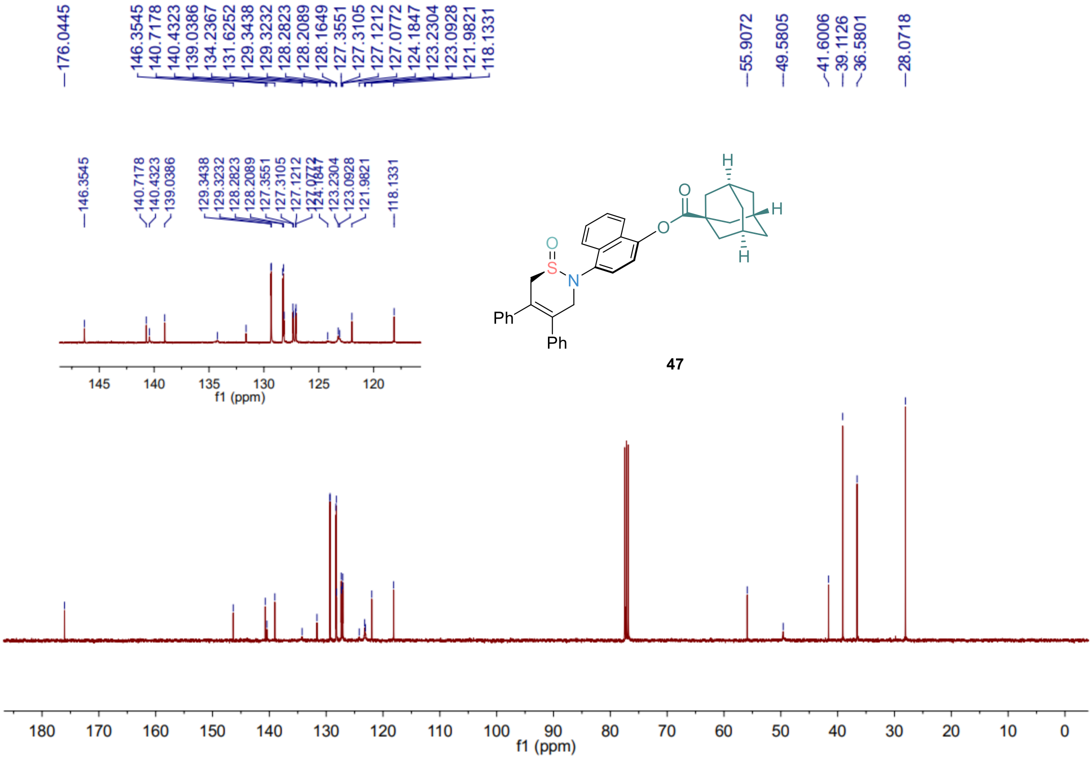

^{1}H-NMR and ^{13}C-NMR of **47**

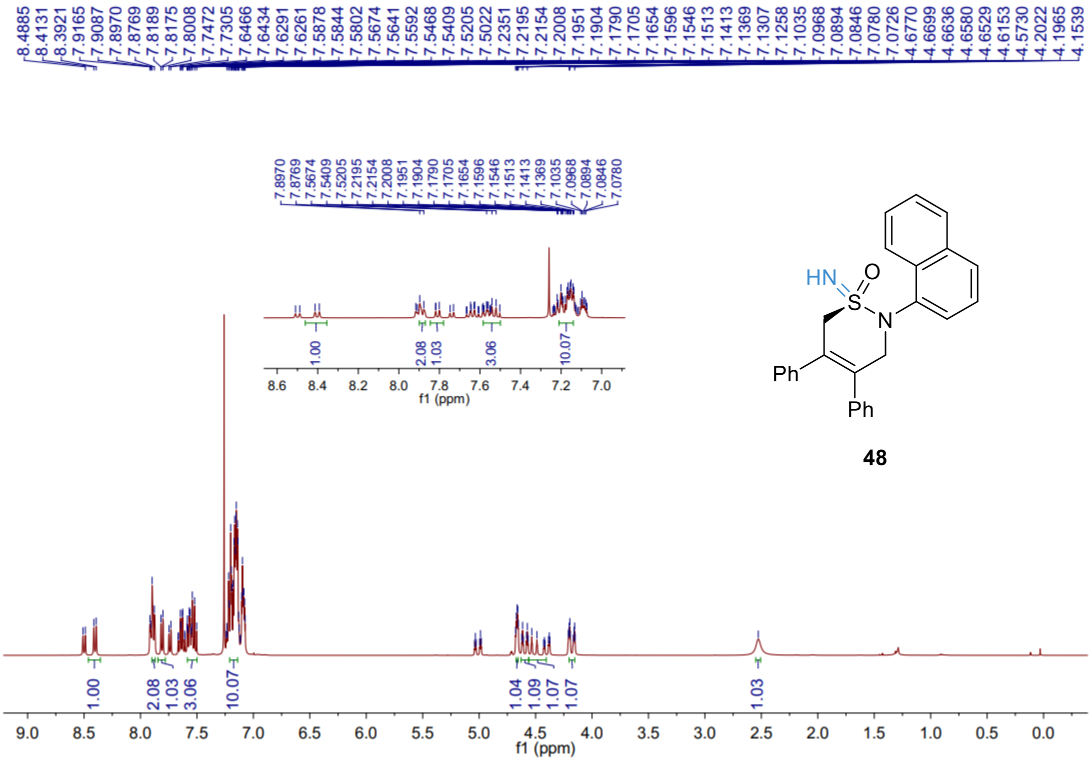

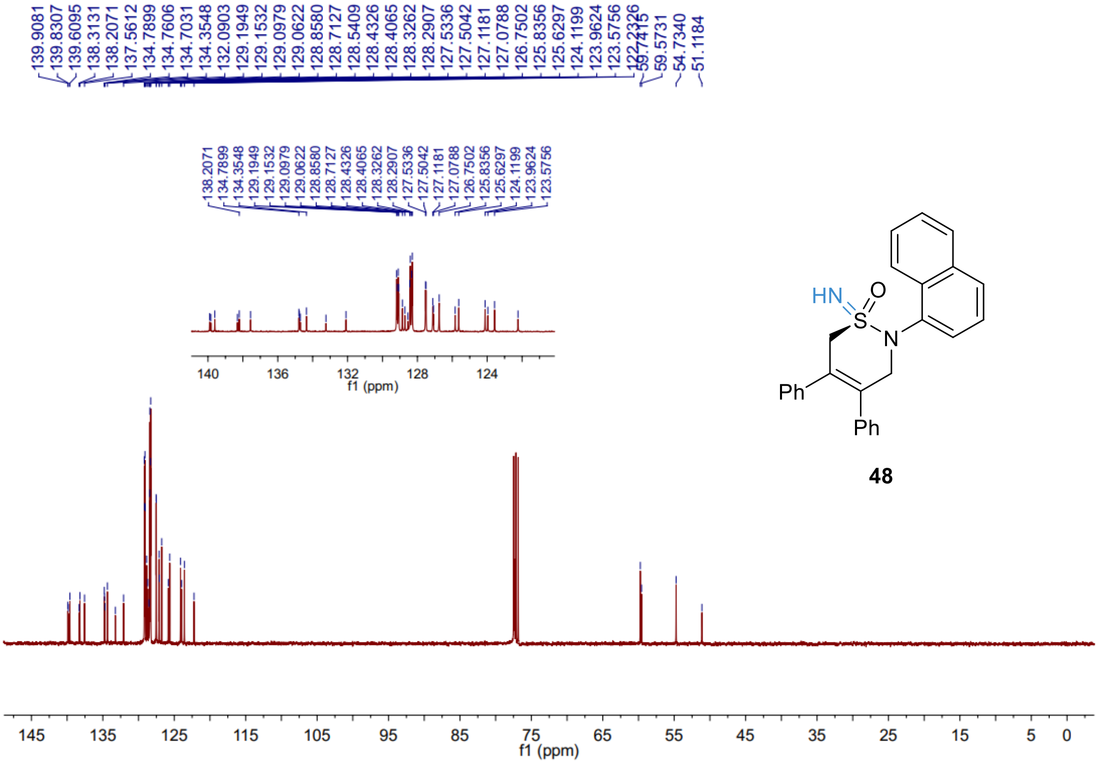

^{1}H-NMR and ^{13}C-NMR of **48**

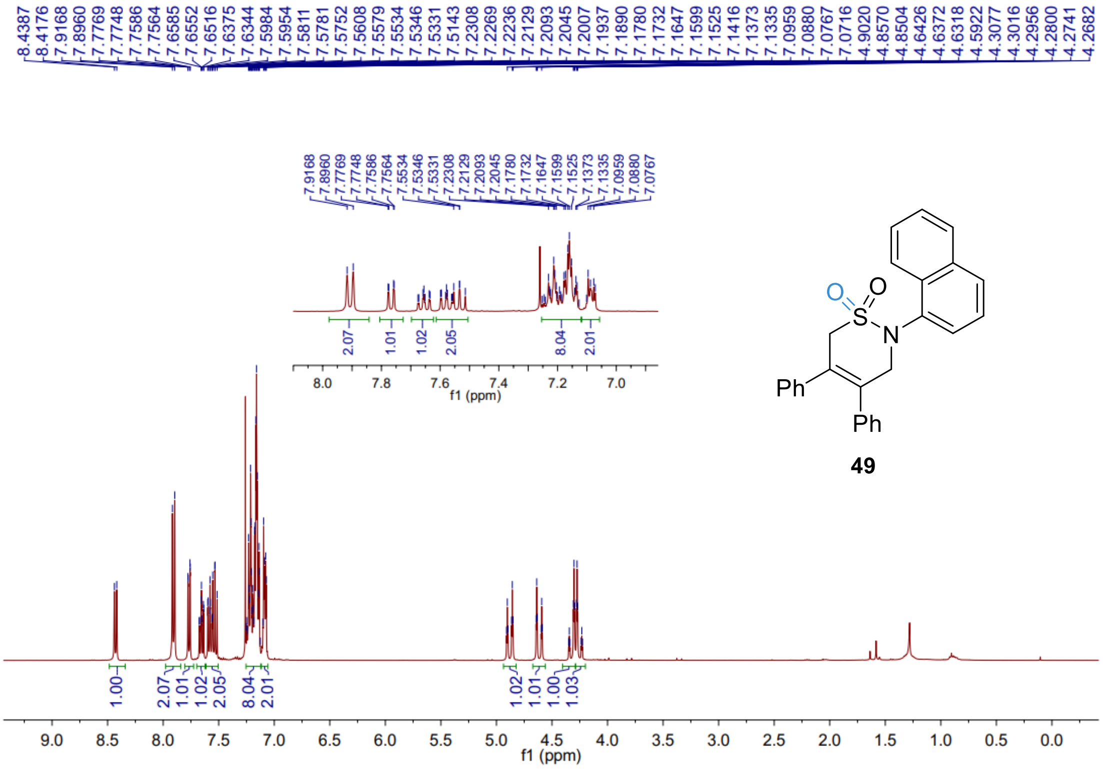

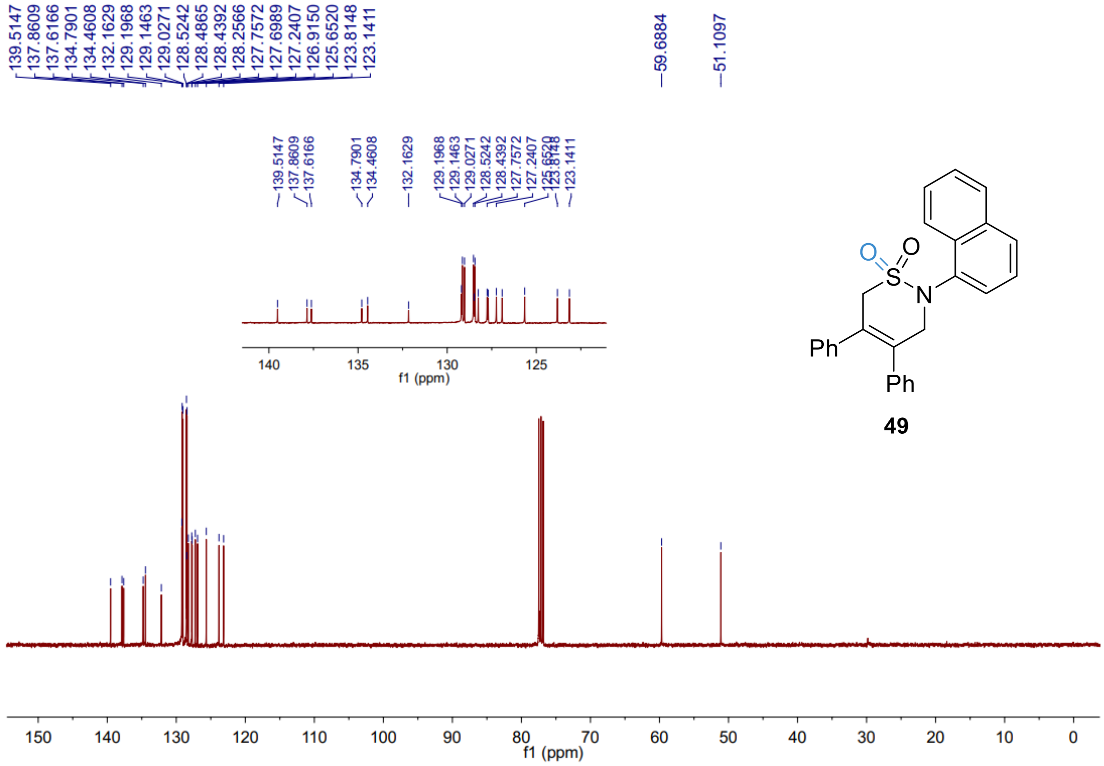

^{1}H-NMR and ^{13}C-NMR of **49**

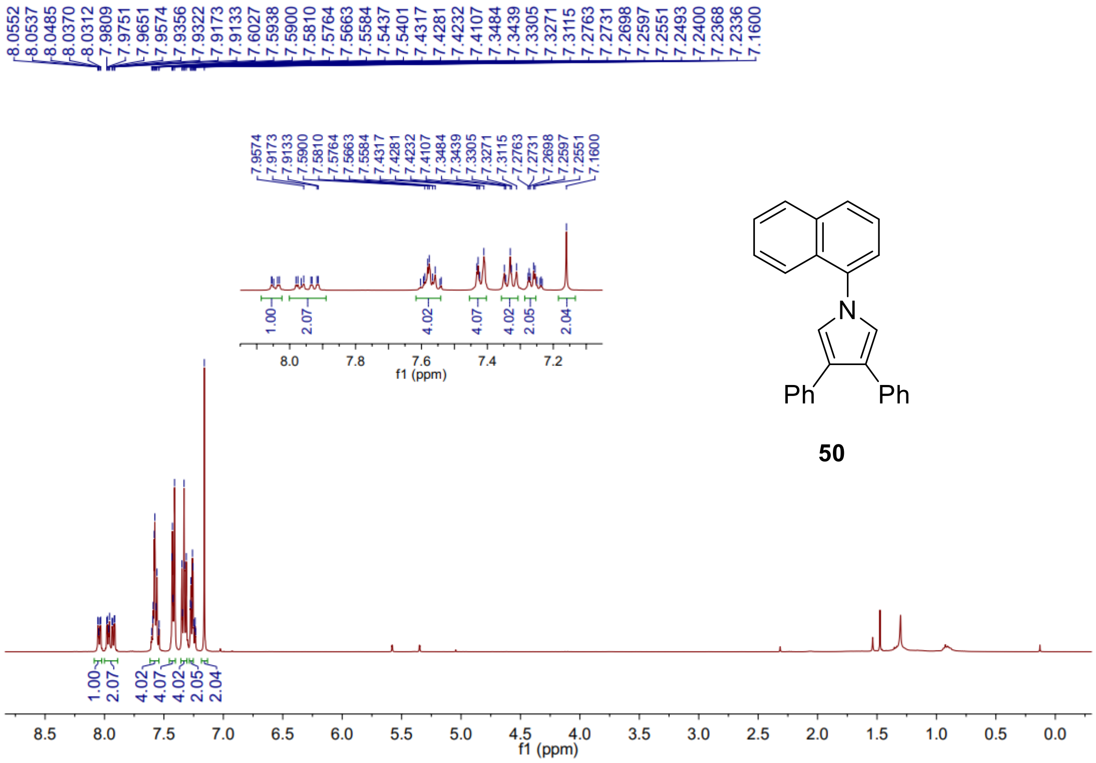

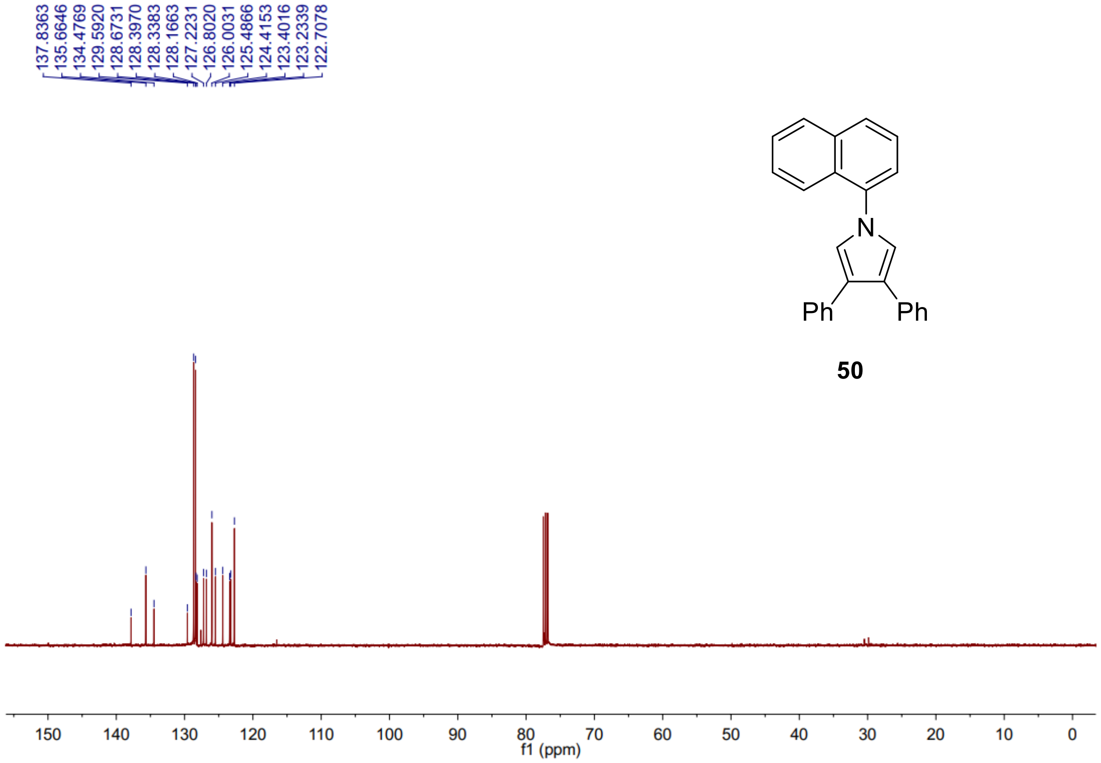

^{1}H-NMR and ^{13}C-NMR of **50**

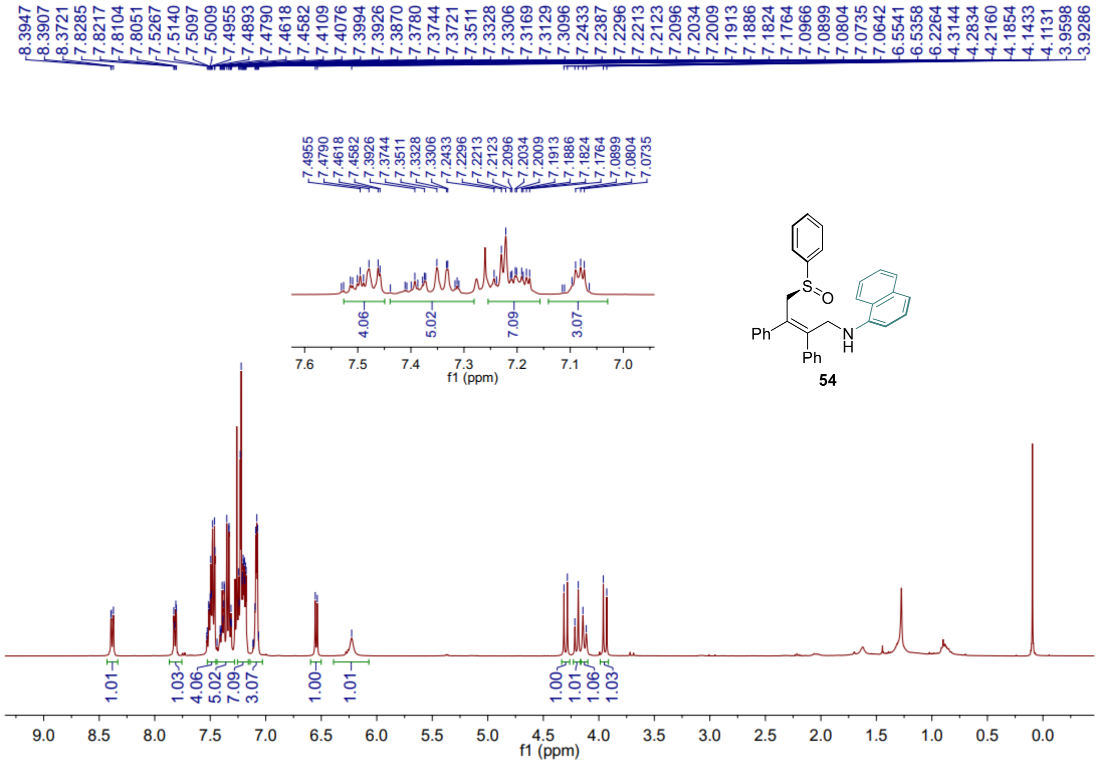

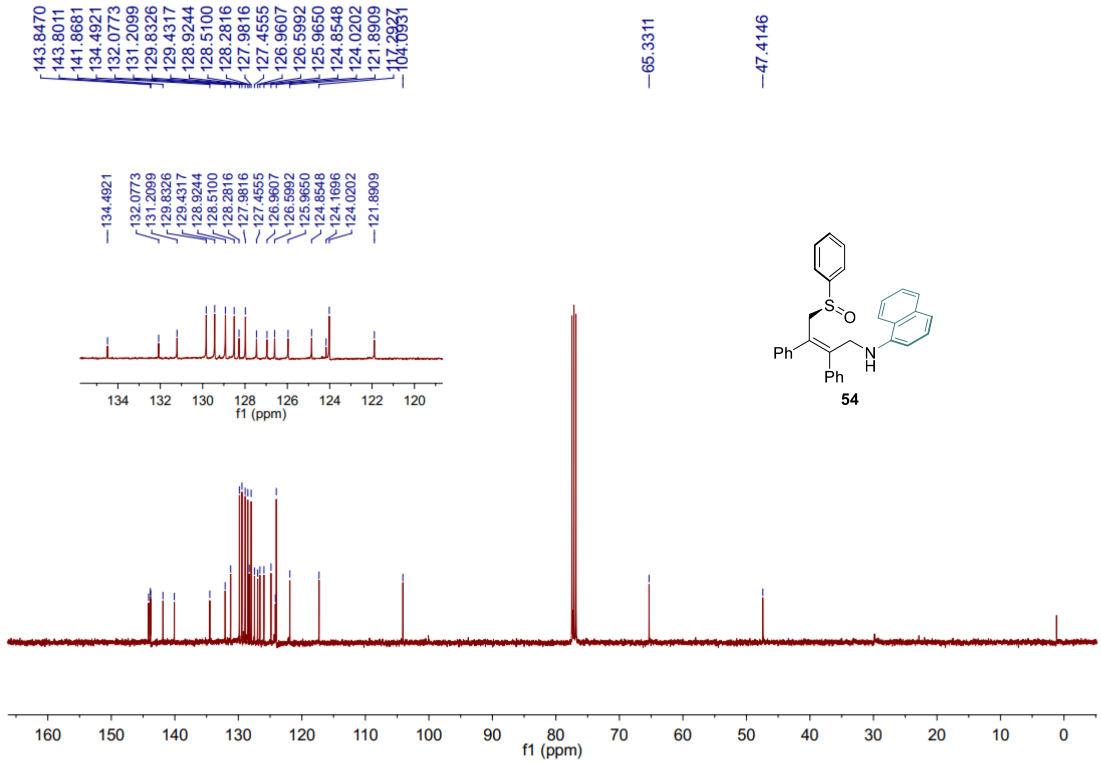

^{1}H-NMR and ^{13}C-NMR of **54**

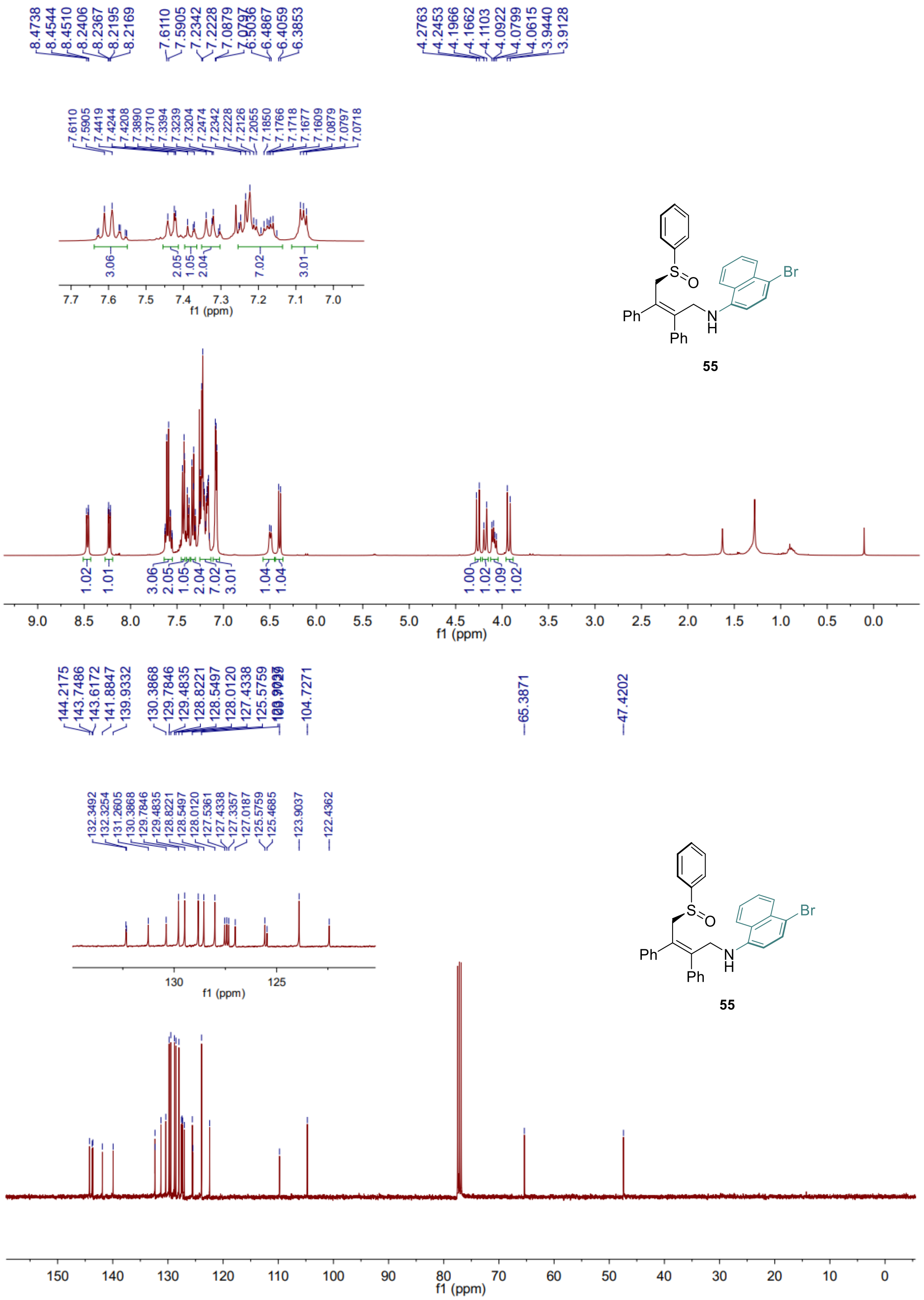

^{1}H-NMR and ^{13}C-NMR of **55**

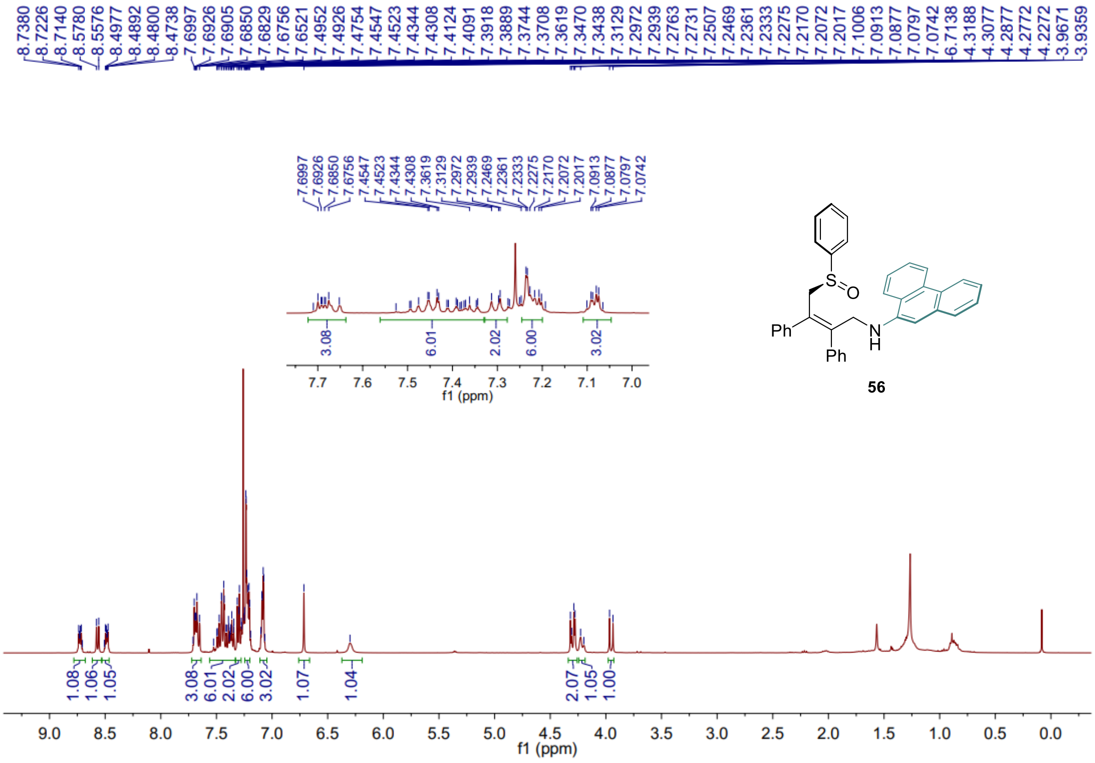

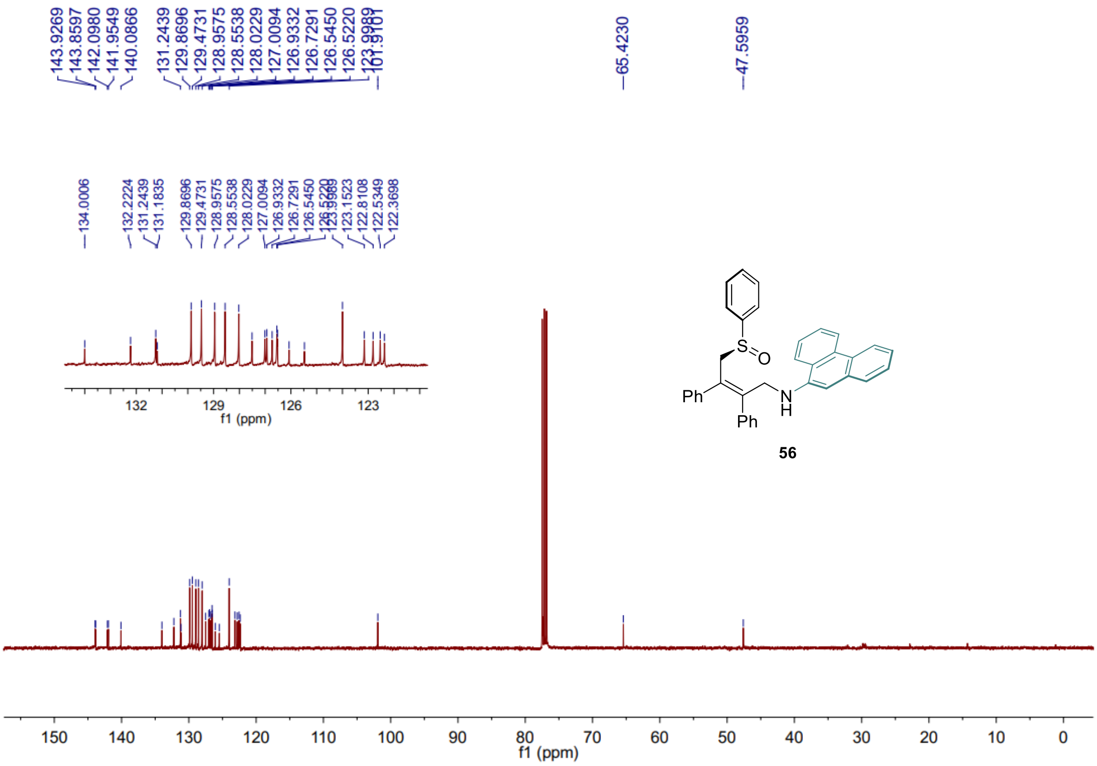

^{1}H-NMR and ^{13}C-NMR of **56**

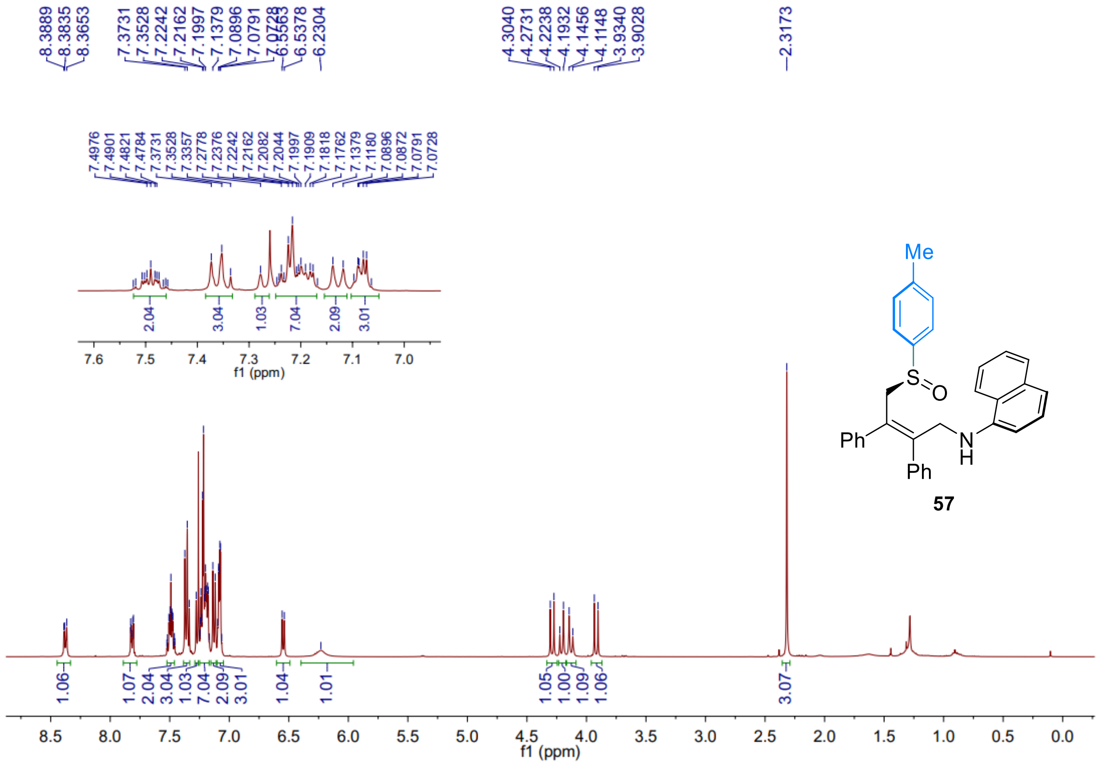

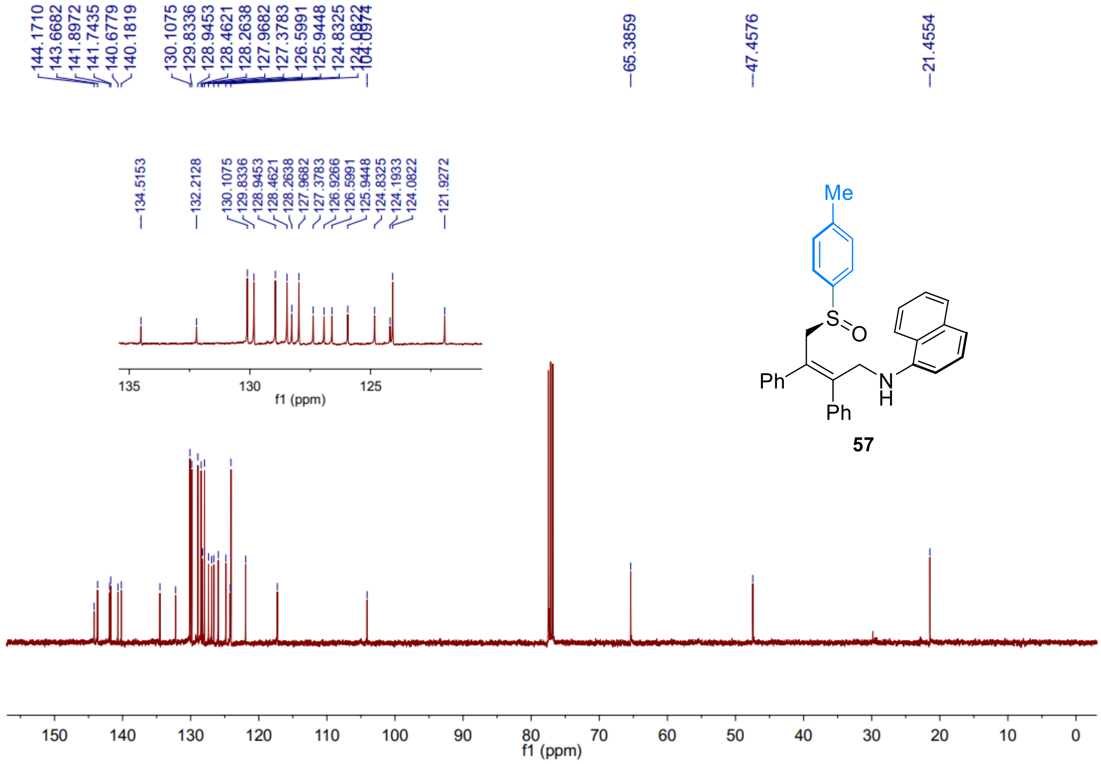

^{1}H-NMR and ^{13}C-NMR of **57**

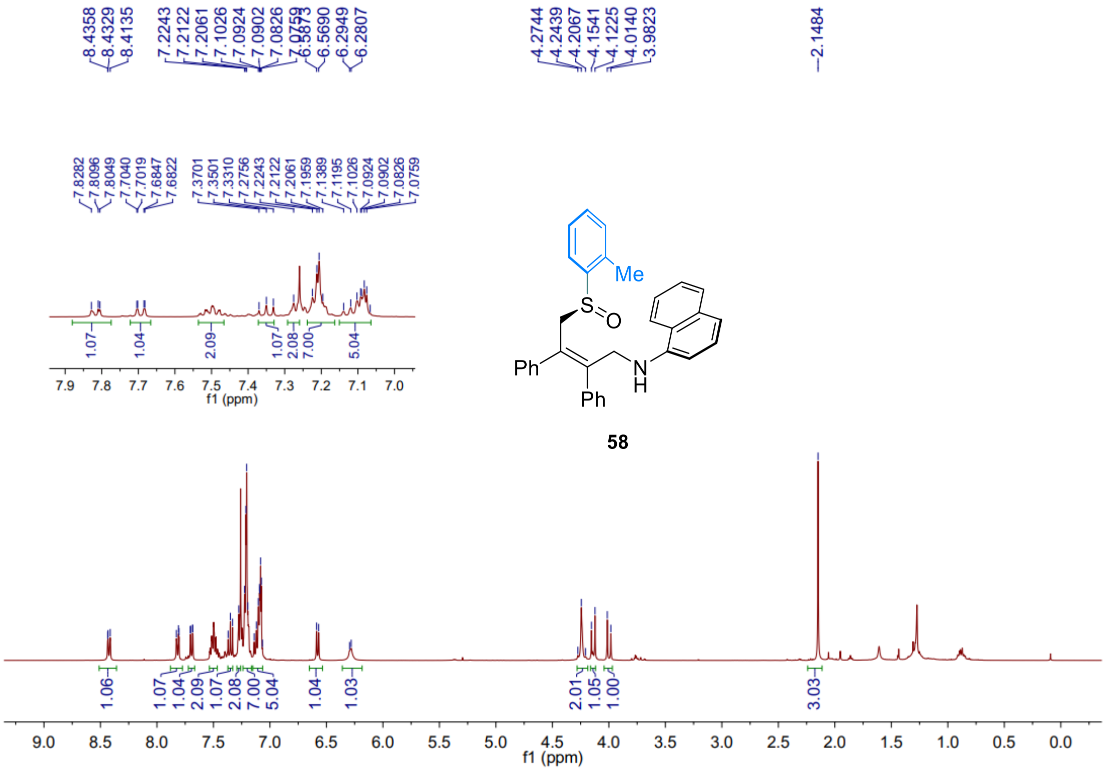

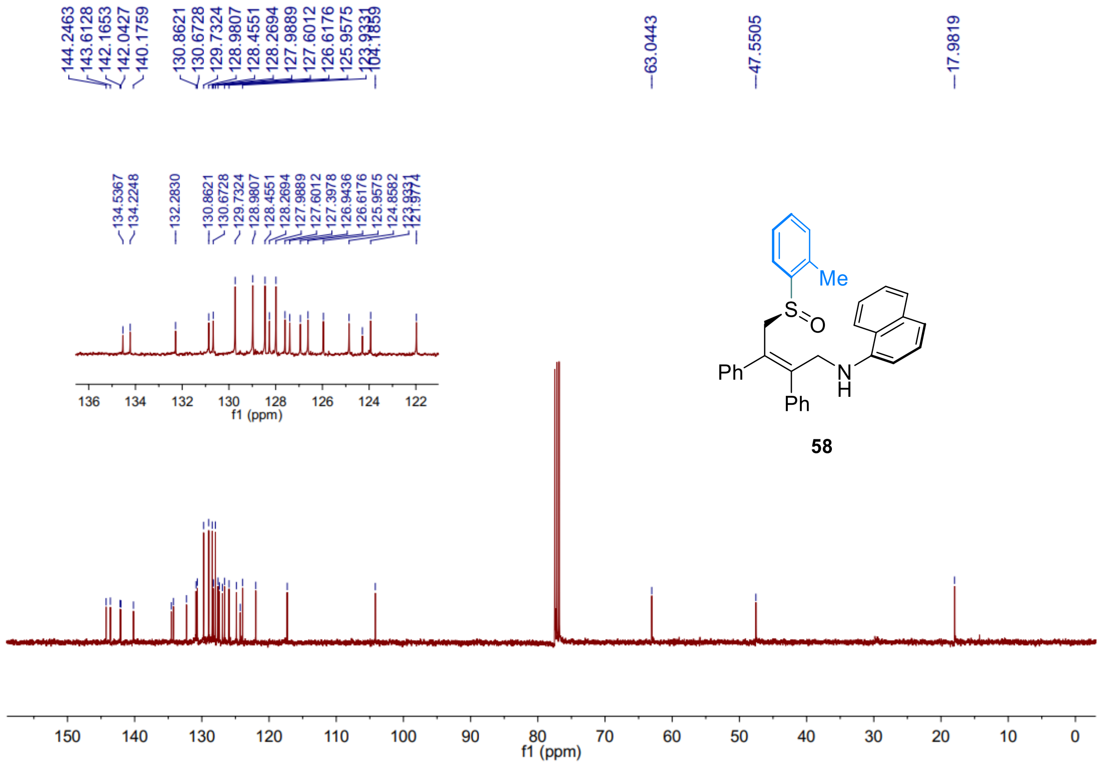

^{1}H-NMR and ^{13}C-NMR of **58**

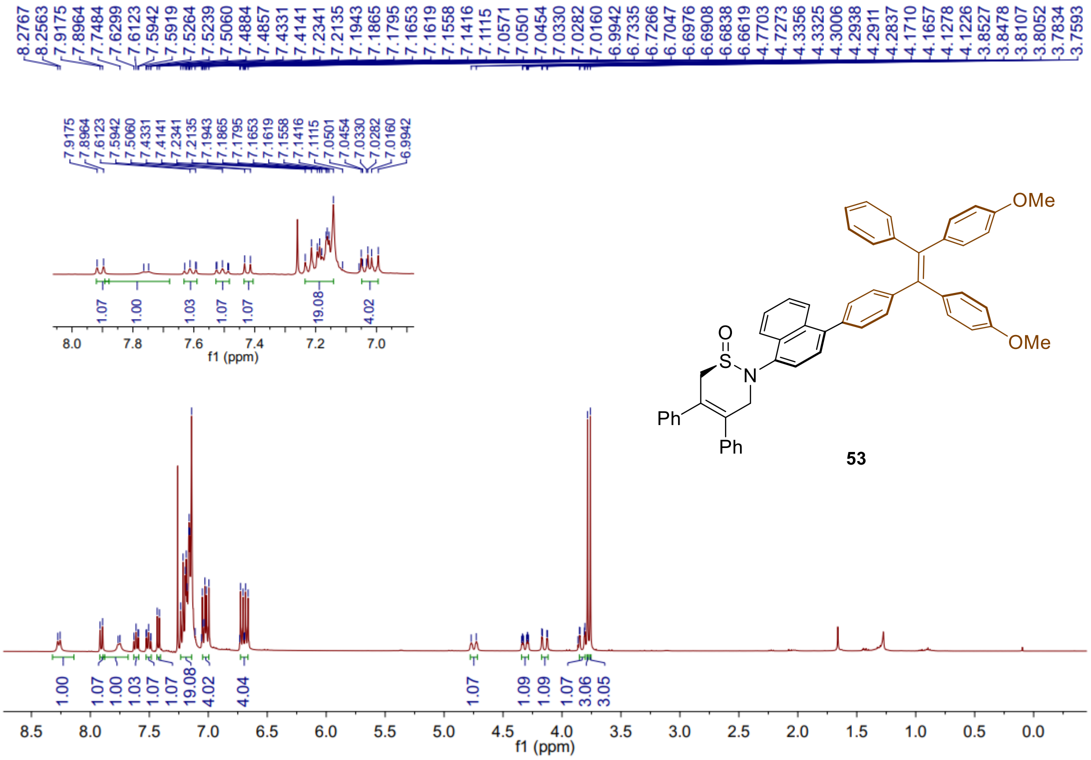

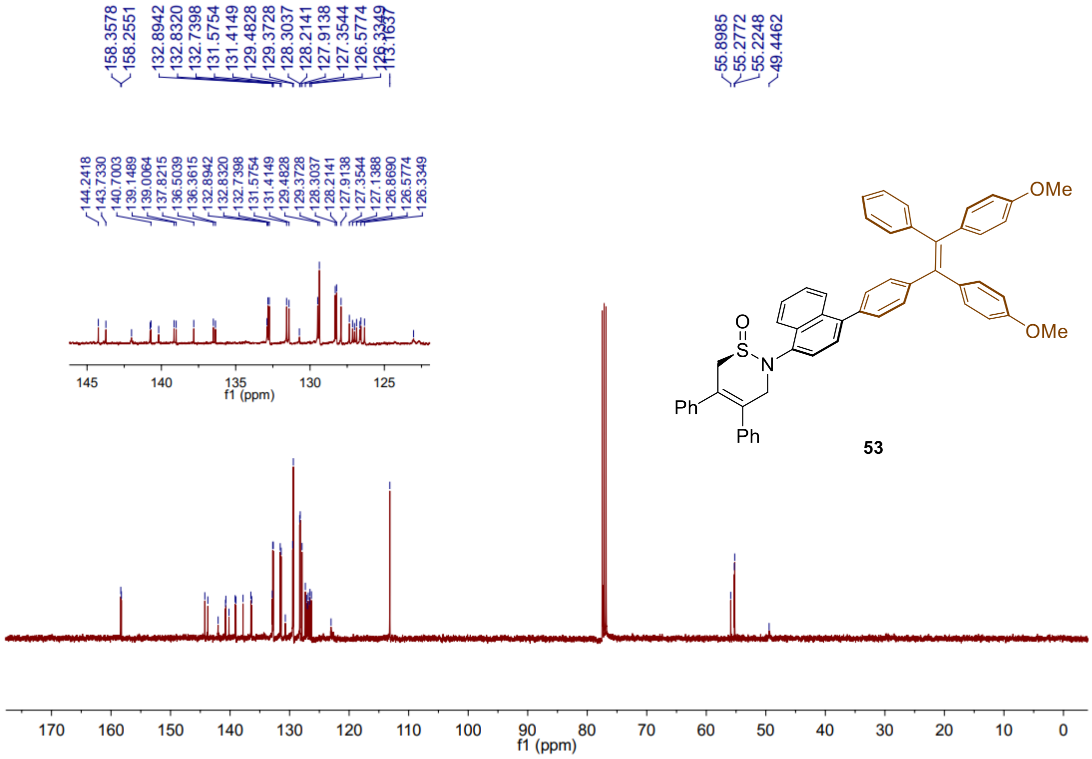

^{1}H-NMR and ^{13}C-NMR of **53**

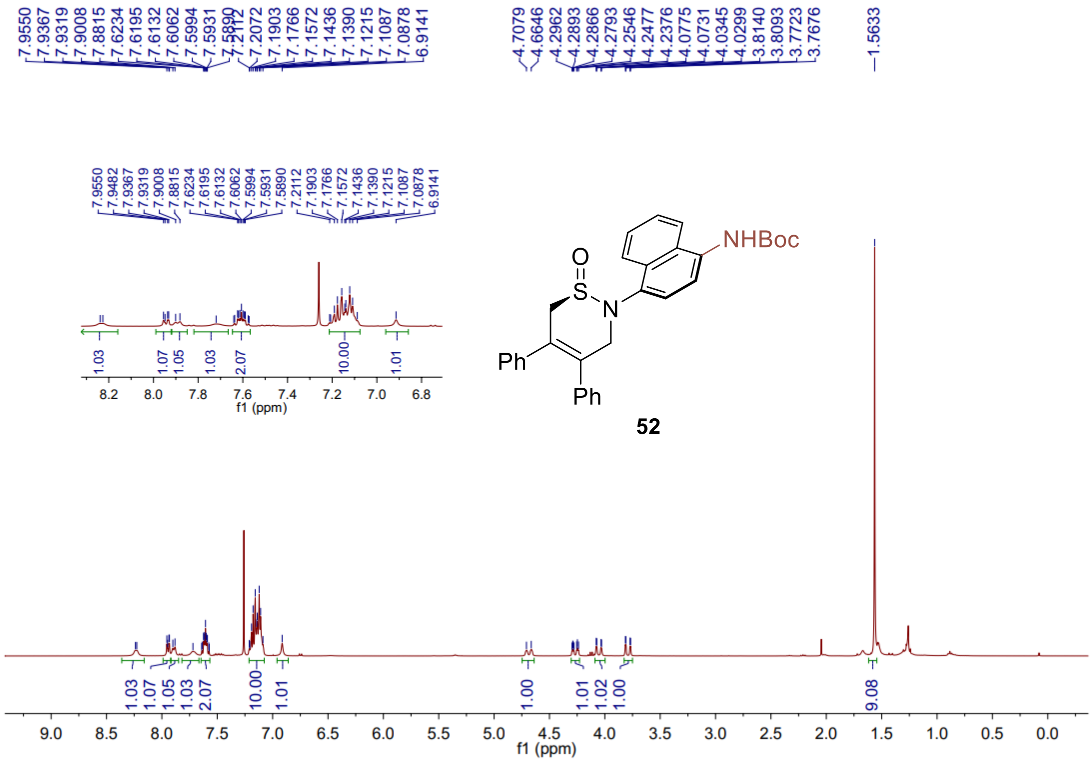

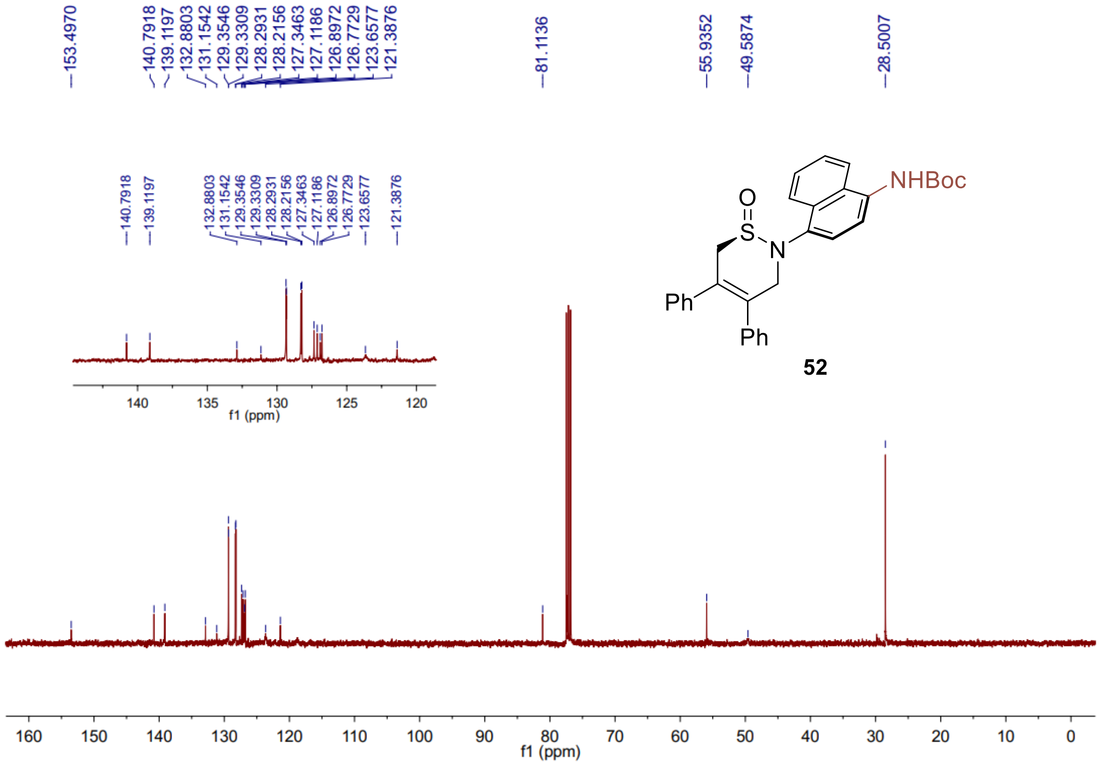

^{1}H-NMR and ^{13}C-NMR of **52**

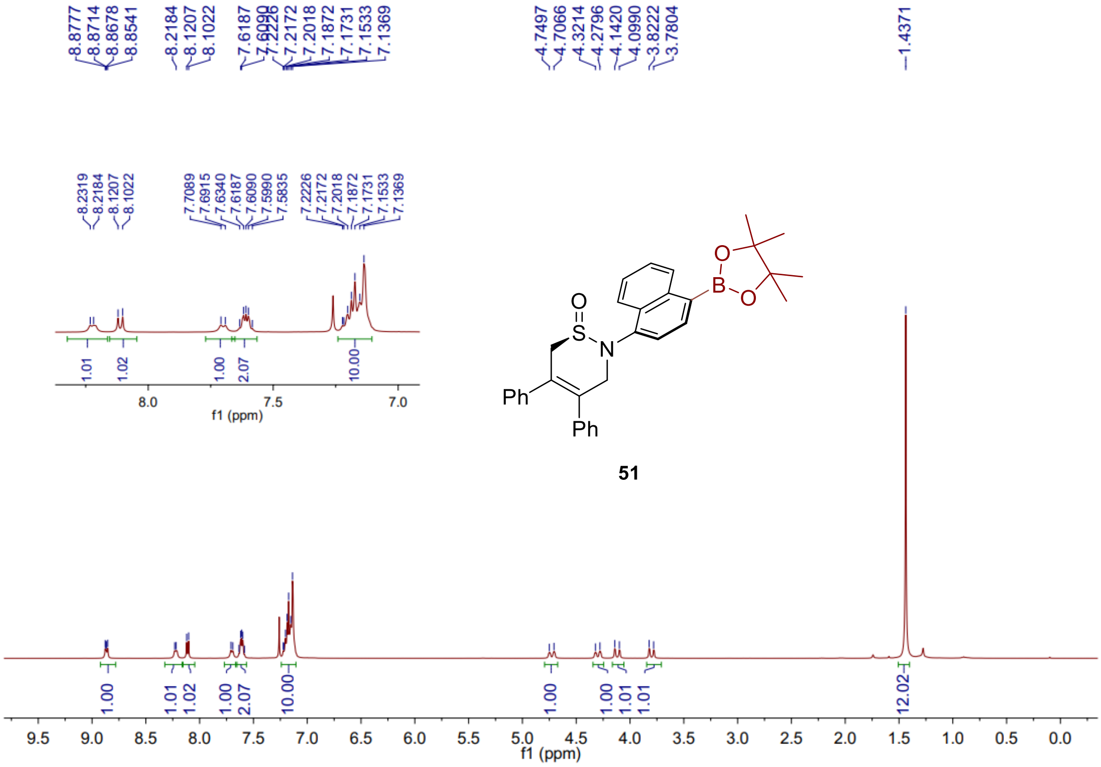

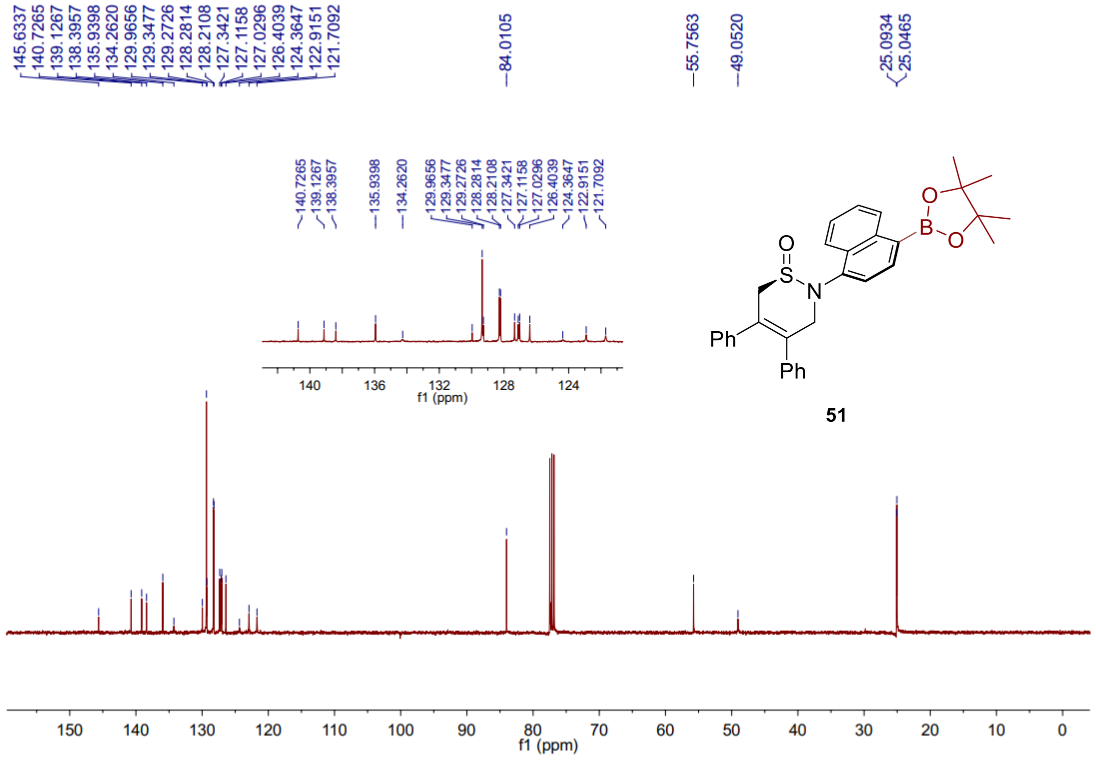

^{1}H-NMR and ^{13}C-NMR of **51**

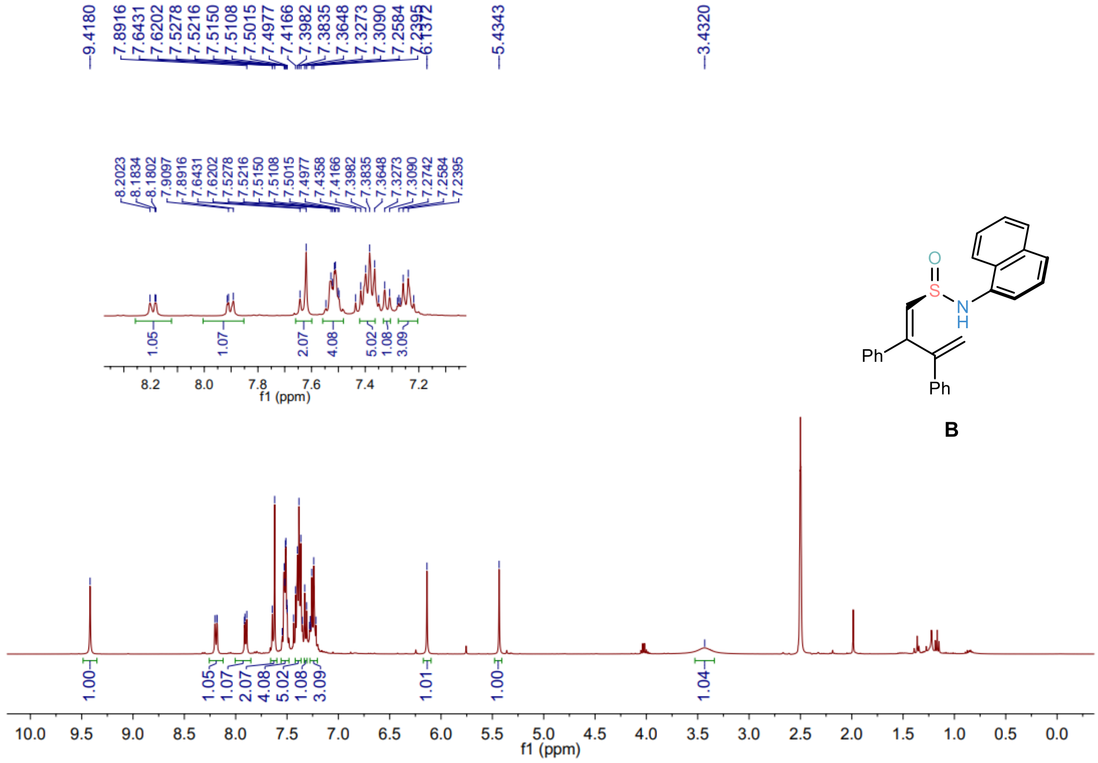

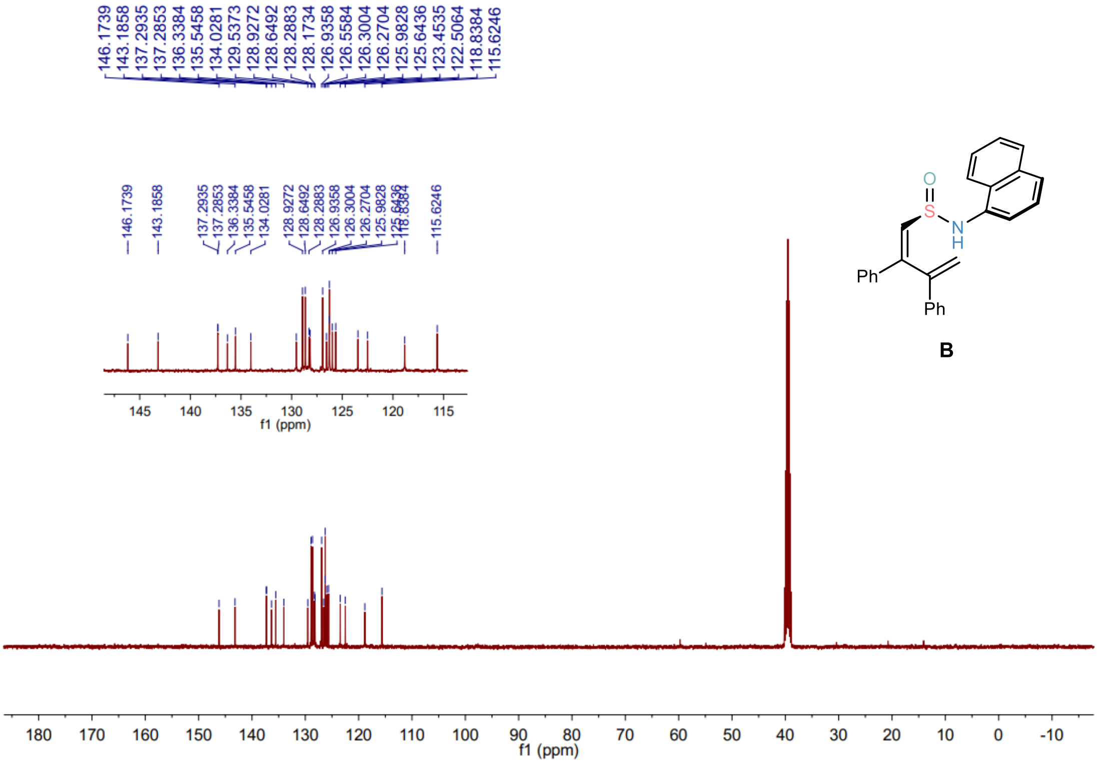

^{1}H-NMR and ^{13}C-NMR of **B**

Supplementary References

1. Han, X., Wang, Y., Zhong, F. & Lu, Y. Enantioselective [3+2] Cycloaddition of Allenes to Acrylates Catalyzed by Dipeptide-Derived Phosphines: Facile Creation of Functionalized Cyclopentenes Containing Quaternary Stereogenic Centers. *J. Am. Chem. Soc.* **133**, 1726-1729 (2011).
2. Zhong, F., Han, X., Wang, Y. & Lu, Y. Highly Enantioselective [3+2] Annulation of Morita-Baylis-Hillman Adducts Mediated by L-Threonine-Derived Phosphines: Synthesis of 3-Spirocyclopentene-2-oxindoles having Two Contiguous Quaternary Centers. *Angew. Chem. Int. Ed.* **50**, 7837-7841 (2011).
3. Han, X., Zhong, F., Wang, Y. & Lu, Y. Versatile Enantioselective [3+2] Cyclization between Imines and Allenoates Catalyzed by Dipeptide-Based Phosphines. *Angew. Chem. Int. Ed*. **51**, 767-770 (2012).
4. Zhong, F., Han, X., Wang, Y. & Lu, Y. Highly enantioselective [4+2] annulations catalyzed by amino acid-based phosphines: Synthesis of functionalized cyclohexenes and 3-spirocyclohexene-2-oxindoles. *Chem. Sci.* **3**, 1231-1234 (2012).
5. Zhong, F., Luo, J., Chen, G.-Y., Dou, X. & Lu, Y. Highly Enantioselective Regiodivergent Allylic Alkylations of MBH Carbonates with Phthalides. *J. Am. Chem. Soc*. **134**, 10222-10227 (2012).
6. Zhong, F. et al. Chiral Phosphine Catalyzed Asymmetric Michael Addition of Oxindoles. *Angew. Chem. Int. Ed.* **52**, 943-947 (2013).
7. Che, J. et al. Asymmetric Mannich/Radical Debromination Cascade of α-Bromoketones by Dipeptide-Phosphonium Salt Catalysis: Enantioselective Synthesis of β-Amino Ketone-Pyrazolinones. *Asian J. Org. Chem.* **12**, e202300079 (2023).
8. Hu, H.-L. et al. Stereodivergently asymmetric synthesis of chiral phosphorus compounds by synergistic combination of ion-pair catalyst and base. *Sci. China Chem*. **65**, 2500-2511 (2022).
9. Chen, Y. et al. Synergistic Catalysis between a Dipeptide Phosphonium Saltanda Metal-Based Lewis Acid for Asymmetric Synthesis of *N*-Bridged [3.2.1] Ring Systems. *Angew. Chem. Int. Ed*. **61**, e202207334 (2022).
10. Ohta, K. et al. Design and Synthesis of Novel Breast Cancer Therapeutic Drug Candidates Based upon the Hydrophobic Feedback Approach of Antiestrogens. *Molecules.* **24**, 3966-3978 (2019).
11. Fang, S. et al. Access to S-Stereogenic Free Sulfoximines via Bifunctional Phosphonium Salt-Catalyzed Desymmetrization of Bisphenols. *ACS Catal.* **11**, 13902-13912 (2021).
12. Gupta, S. Baranwal, S. Muniyappan, N. Sabiah, S. & Kandasamy, J. Copper-Catalyzed *N*-Arylation of Sulfoximines with Arylboronic Acids under Mild Conditions. Synthesis, **51**, 2171-2182 (2019).
13. Molander, G. A., Cavalcanti, L. N. & García-García, C. Nickel-Catalyzed Borylation of Halides and Pseudohalides with Tetrahydroxydiboron [$B_2(OH)_4$]. *J. Org. Chem*. 78, 6427-6439 (2013).
14. Frisch, M. J. et al. Fox, Gaussian 09, Revision D.01, Gaussian, Inc. Wallingford, CT (2013).
15. Zhao, Y. & Truhlar, D.G. The Mo6 suite of density functionals for main group thermochemistry, thermochemical kinetics, noncovalent interactions, excited states, and transition elements: two new functionals and systematic testing of four Mo6-class functionals and 12 other functionals. *Theor. Chem. Acc*. **120**, 215-241 (2008).

16. Tomasi, J., Mennucci, B. & Cammi, R. Quantum Mechanical Continuum Solvation Models. *Chem. Rev.* **105**, 2999-3094 (2005).
17. Grimme, S., Antony, J., Ehrlich, S. & Krieg, H. A consistent and accurate *ab initio* parametrization of density functional dispersion correction (DFT-D) for the 94 elements H-Pu. *J. Chem. Phys.* **132**, 154104-154119 (2010).
18. Grimme, S., Ehrlich, S. & Goerigk, L. Effect of the damping function in dispersion corrected density functional theory. *J. Comput. Chem.* **32**, 1456-1465 (2011).
19. Gonzalez, C. & Schlegel, H. B. An improved algorithm for reaction path following. *J. Chem. Phys*. **90**, 2154-2161 (1989).
20. CYLview20; Legault, C. Y., Université de Sherbrooke (2020) (http://www.cylview.org)
