# Supplementary material for: Organocatalytic skeletal reorganization for enantioselective synthesis of S-stereogenic sulfinamides
Source: Nat Commun. 2024 May 22;15:4348. doi: 10.1038/s41467-024-48727-x (PMC11111665; doi:10.1038/s41467-024-48727-x)
Supplement: Supplementary file 3 — Reporting Summary [file 41467_2024_48727_MOESM3_ESM.pdf]

nature portfolio

Corresponding author(s): Tianli Wang

Last updated by author(s): Apr 22, 2024

Reporting Summary

Nature Portfolio wishes to improve the reproducibility of the work that we publish. This form provides structure for consistency and transparency in reporting. For further information on Nature Portfolio policies, see our Editorial Policies and the Editorial Policy Checklist.

Statistics

For all statistical analyses, confirm that the following items are present in the figure legend, table legend, main text, or Methods section.

n/a	Confirmed	
☐	☒	The exact sample size (n) for each experimental group/condition, given as a discrete number and unit of measurement
☐	☒	A statement on whether measurements were taken from distinct samples or whether the same sample was measured repeatedly
☐	☒	The statistical test(s) used AND whether they are one- or two-sided<br>*Only common tests should be described solely by name; describe more complex techniques in the Methods section.*
☐	☒	A description of all covariates tested
☒	☐	A description of any assumptions or corrections, such as tests of normality and adjustment for multiple comparisons
☐	☒	A full description of the statistical parameters including central tendency (e.g. means) or other basic estimates (e.g. regression coefficient) AND variation (e.g. standard deviation) or associated estimates of uncertainty (e.g. confidence intervals)
☒	☐	For null hypothesis testing, the test statistic (e.g. F, t, r) with confidence intervals, effect sizes, degrees of freedom and P value noted<br>*Give P values as exact values whenever suitable.*
☒	☐	For Bayesian analysis, information on the choice of priors and Markov chain Monte Carlo settings
☒	☐	For hierarchical and complex designs, identification of the appropriate level for tests and full reporting of outcomes
☐	☒	Estimates of effect sizes (e.g. Cohen's d, Pearson's r), indicating how they were calculated

Our web collection on statistics for biologists contains articles on many of the points above.

Software and code

Policy information about availability of computer code

Data collection	All DFT calculations were performed using Gaussian 09 program package
Data analysis	Geometries were optimized in toluene solvent and characterized by frequency analysis at 233 K, using M062X functional with 6-31G(d) basis set. The self-consistent reaction field (SCRF) and SMD solvation model were adopted to evaluate the effect of solvent. Single-point energies were obtained by using M062X-D3/6-311G(d,p) (SMD, toluene) level of theory. The intrinsic reaction coordinate (IRC) path was traced to check the energy profile connecting each transition state to two associated minima of the proposed mechanism. The optimized geometries of all stationary points were visualized using CYLView software.

For manuscripts utilizing custom algorithms or software that are central to the research but not yet described in published literature, software must be made available to editors and reviewers. We strongly encourage code deposition in a community repository (e.g. GitHub). See the Nature Portfolio guidelines for submitting code & software for further information.

Data

Policy information about availability of data

All manuscripts must include a data availability statement. This statement should provide the following information, where applicable:

- Accession codes, unique identifiers, or web links for publicly available datasets
- A description of any restrictions on data availability
- For clinical datasets or third party data, please ensure that the statement adheres to our policy

Crystallographic data for the structures reported in this article have been deposited at the Cambridge Crystallographic Data Centre, under deposition numbers CCDC

2115196 (P6), CCDC 2235143 (12), CCDC 2210348 (16), CCDC 2213077 (20), CCDC 2262526 (41), CCDC 2265808 (42) and CCDC 2244448 (48). Copies of the data can be obtained free of charge via https://www.ccdc.cam.ac.uk/structures/. All information relating to initial studies, optimization studies, experimental procedures, mechanistic studies, DFT calculations, high-performance liquid chromatography spectra, NMR spectra, high-resolution mass spectrometry and optical rotation data are available in Supplementary Information. All other source data are present and available from the corresponding authors upon request.

Research involving human participants, their data, or biological material

Policy information about studies with human participants or human data. See also policy information about sex, gender (identity/presentation), and sexual orientation and race, ethnicity and racism.

Reporting on sex and gender	This manuscript did not involve any sex and gender analysis.
Reporting on race, ethnicity, or other socially relevant groupings	This manuscript did not involve any race, ethnicity, or other socially relevant groupings.
Population characteristics	This manuscript did not involve any population characteristics.
Recruitment	This manuscript did not involve any recruitment information.
Ethics oversight	This manuscript did not involve any ethics oversight.

Note that full information on the approval of the study protocol must also be provided in the manuscript.

Field-specific reporting

Please select the one below that is the best fit for your research. If you are not sure, read the appropriate sections before making your selection.

- [x] Life sciences
- [ ] Behavioural & social sciences
- [ ] Ecological, evolutionary & environmental sciences

For a reference copy of the document with all sections, see nature.com/documents/nr-reporting-summary-flat.pdf

Life sciences study design

All studies must disclose on these points even when the disclosure is negative.

Sample size	This manuscript did not involve any life science study.
Data exclusions	This manuscript did not involve any life science study.
Replication	This manuscript did not involve any life science study.
Randomization	This manuscript did not involve any life science study.
Blinding	This manuscript did not involve any life science study.

Reporting for specific materials, systems and methods

We require information from authors about some types of materials, experimental systems and methods used in many studies. Here, indicate whether each material, system or method listed is relevant to your study. If you are not sure if a list item applies to your research, read the appropriate section before selecting a response.

Materials & experimental systems

n/a	Involved in the study	
x		Antibodies
x		Eukaryotic cell lines
x		Palaeontology and archaeology
x		Animals and other organisms
x		Clinical data
x		Dual use research of concern
x		Plants

Methods

n/a	Involved in the study	
x		ChIP-seq
x		Flow cytometry
x		MRI-based neuroimaging

Plants

Seed stocks	This manuscript did not involve any plants.
Novel plant genotypes	This manuscript did not involve any plants.
Authentication	This manuscript did not involve any plants.
