# Supplementary material for: Organocatalytic skeletal reorganization for enantioselective synthesis of S-stereogenic sulfinamides
Source: Nat Commun. 2024 May 22;15:4348. doi: 10.1038/s41467-024-48727-x (PMC11111665; doi:10.1038/s41467-024-48727-x)
Supplement: Supplementary file 2 — Peer Review File [file 41467_2024_48727_MOESM2_ESM.pdf]

nature portfolio

Peer Review File

Organocatalytic skeletal reorganization for enantioselective synthesis of S-stereogenic sulfinamides

Open Access This file is licensed under a Creative Commons Attribution 4.0 International License, which permits use, sharing, adaptation, distribution and reproduction in any medium or format, as long as you give appropriate credit to the original author(s) and the source, provide a link to the Creative Commons license, and indicate if changes were made. In the cases where the authors are anonymous, such as is the case for the reports of anonymous peer reviewers, author attribution should be to 'Anonymous Referee' followed by a clear attribution to the source work. The images or other third party material in this file are included in the article's Creative Commons license, unless indicated otherwise in a credit line to the material. If material is not included in the article's Creative Commons license and your intended use is not permitted by statutory regulation or exceeds the permitted use, you will need to obtain permission directly from the copyright holder. To view a copy of this license, visit http://creativecommons.org/licenses/by/4.0/.

Editorial Note: This manuscript has been previously reviewed at another journal that is not operating a transparent peer review scheme. This document only contains reviewer comments and rebuttal letters for versions considered at *Nature Communications.*

REVIEWERS' COMMENTS

Reviewer #2 (Remarks to the Author):

In this manuscript, Su, Wang, and coworkers reported a phosphonium salt-catalyzed desymmetrization of cyclic sulfoximines into chiral sulfinamides. The transformation proceeds via a rearrangement/skeletal reorganization of the sulfoximine nitrogen inserted into the parent five-membered ring to generate the corresponding six-membered sulfinamide with an S-stereogenic center. The authors have appropriately addressed the main concerns raised during the previous submission by carrying out additional experiments and revising the manuscript accordingly. Considering the high significance of the reported reaction in both synthetic utility and intriguing mechanism, this reviewer recommends its publication in Nature Communications in the current form.
